# ﻿A revision of the parasitoid wasp genus *Dolichogenidea* Viereck (Hymenoptera, Braconidae) in the Neotropical region, with the description of 102 new species

**DOI:** 10.3897/zookeys.1237.141007

**Published:** 2025-05-07

**Authors:** Jose L. Fernandez-Triana, Caroline Boudreault, James B. Whitfield, Amelie Höcherl, M. Alex Smith, Winnifred Hallwachs, Daniel H. Janzen

**Affiliations:** 1 Canadian National Collection of Insects, Arachnids and Nematodes, AAFC, Ottawa, Canada Canadian National Collection of Insects, Arachnids and Nematodes Ottawa Canada; 2 University of Illinois, Urbana, USA University of Illinois Urbana United States of America; 3 Stuttgart State Museum of Natural History, Stuttgart, Germany Stuttgart State Museum of Natural History Stuttgart Germany; 4 Department of Integrative Biology, University of Guelph, Guelph, Ontario, Canada University of Guelph Guelph Canada; 5 University of Pennsylvania, Philadelphia, USA University of Pennsylvania Philadelphia United States of America

**Keywords:** DNA barcoding, host data, Microgastrinae, morphology, Neotropics, turbo taxonomy

## Abstract

The parasitoid wasp genus *Dolichogenidea* is currently the second most speciose within the subfamily Microgastrinae (Hymenoptera: Braconidae), with 366 world species known so far, but with hundreds awaiting to be described. Here, the fauna of the Neotropical region is revised, with an emphasis in the Area de Conservación Guanacaste (ACG), Costa Rica. In addition to 23 species previously recorded from the Neotropics, 102 additional species are described as new, increasing the regional and world richness to 125 and 468 species, respectively. All species are diagnosed and described by using a combination of basic morphology (dichotomous key and brief diagnostic descriptions) and, when available DNA COI barcodes, biology (host data and wasp cocoon strategy), and distribution data. Neither morphology, biology, nor molecular data alone were sufficient to unambiguously separate all taxa, as all approaches were found to have limitations, but the combination of all three approaches provided stronger support to species delimitation. Morphology allowed the inclusion of all known species, therefore building a foundation upon which to improve as more molecular and biological data become available and new species are discovered; however, it was not sufficient (or it was very difficult to use) to separate at least 15% of all species keyed out in the dichotomous key. DNA barcoding was better able to separate species, and it is likely to become the most efficient way to identify species in the near future; however, DNA failed to identify 8.3% of the species with molecular data available, in addition to one third of the described species currently lacking molecular data. Biological data is currently the most incomplete, with only 42% of the species having associated host information, with a strong data availability bias towards ACG specimens. A total of 11 Lepidoptera families are here recorded to be parasitized by Neotropical *Dolichogenidea*, mainly Depressariidae (34% of all host data available), Gelechiidae (17%), Crambidae (14%), Tortricidae (10%), Thyrididae (8%) and Pyralidae (7%). Most of the wasps seem to be monophagous or at most oligophagous, as 56% are known to only parasitize a single host species, whereas 23% parasitize two host species and 10% parasitize three hosts; in almost all cases, the hosts species belong to one genus (or related genera) in the same Lepidoptera family. Most species of *Dolichogenidea* are found between 400–1,500 m, but a few have been found at higher elevations, including a few examples higher than 3,000 m (Costa Rica) and 4,000–4,100 m in the Andes (South America). The following nomenclatural acts are proposed: 1) the genus *Exoryza* is synonymized under *Dolichogenidea*, **syn. nov.**; 2) a total of 16 species are transferred to *Dolichogenidea* as **comb. nov.**, one species formerly in the genus *Apanteles*: *Dolichogenideacroceicornis* (Muesebeck, 1958) and all 15 species formerly placed within *Exoryza* (six of them from the Neotropics): *Dolichogenideaasotae* (Watanabe, 1932), *Dolichogenideabelippicola* (Liu & You, 1988), *Dolichogenideahylas* (Wilkinson, 1932), *Dolichogenideamariabustosae* (Fernandez-Triana, 2016), *Dolichogenideamegagaster* (de Saeger, 1944), *Dolichogenideaminnesota* (Mason, 1981), *Dolichogenideamonocavus* (Valerio & Whitfield, 2004), *Dolichogenideaoryzae* Walker, 1994, *Dolichogenideareticarina* (Song & Chen, 2003), *Dolichogenidearichardashleyi* (Fernandez-Triana, 2016), *Dolichogenidearitaashleyae* (Fernandez-Triana, 2016), *Dolichogenidearosamatarritae* (Fernandez-Triana, 2016), *Dolichogenideasafranum* (Rousse & Gupta, 2013), *Dolichogenideaschoenobii* (Wilkinson, 1932) and *Dolichogenideayeimycedenoae* (Fernandez-Triana, 2016); 3) *Dolichogenideayeimycedenoae* (Fernandez-Triana, 2016) becomes a senior secondary homonym of *Dolichogenideayeimycedenoae* Fernandez-Triana & Boudreault, 2019; therefore, *Dolichogenideacedenoae* Fernandez-Triana & Boudreault, 2025 is a replacement name for *Dolichogenideayeimycedenoae* Fernandez-Triana & Boudreault, 2019; 4) the following 102 species, all authored by Fernandez-Triana & Boudreault, are described as **sp. nov.**: *D.aceituno*, *D.alanflemingi*, *D.alejandromarini*, *D.alerce*, *D.alexamasisae*, *D.alexandrei*, *D.alixhamiltonae*, *D.amazonas*, *D.anacamposae*, *D.andreamezae*, *D.angelsolisi*, *D.anikenpalolae*, *D.anniapicadoae*, *D.annlisterudae*, *D.annychaverae*, *D.antioquia*, *D.antjevirkusae*, *D.arenal*, *D.bernardoespinozai*, *D.beryllacosteae*, *D.bradzlotnicki*, *D.caldas*, *D.carlosalvaradoi*, *D.carlosviquezi*, *D.chichicastenango*, *D.christinaagapakisae*, *D.claudiadoblesae*, *D.dole*, *D.encruzilhada*, *D.ericpalolai*, *D.ericsimoni*, *D.escobarae*, *D.felipechavarriai*, *D.frankjoycei*, *D.fredhicksi*, *D.helenedumasae*, *D.heredia*, *D.ingredolsonae*, *D.isabelleae*, *D.isidrochaconi*, *D.jaimelewisi*, *D.jasonkelleyi*, *D.jennyphillipsae*, *D.jessiehillae*, *D.johnrobinsoni*, *D.jorgecarvajali*, *D.jorgecortesi*, *D.josephfridmani*, *D.joshdarfleri*, *D.juanmatai*, *D.junhyongkimi*, *D.kasiiya*, *D.katiemccluskeyae*, *D.kenzabaddouae*, *D.lacochaparamo*, *D.leahdennisae*, *D.limoncocha*, *D.luishamiltoni*, *D.luzmariaromeroae*, *D.machupichu*, *D.mehdirheljari*, *D.moniqueae*, *D.moniquegilbertae*, *D.ninamasisae*, *D.nothofagus*, *D.oiketicus*, *D.palenque*, *D.papallacta*, *D.paulfryi*, *D.pedroleoni*, *D.puschendorfi*, *D.putumayo*, *D.puyo*, *D.rexhamiltoni*, *D.robertofernandezi*, *D.robinsherwoodae*, *D.robmacewani*, *D.robpringlei*, *D.rociocordobae*, *D.rodrigogamezi*, *D.ronaldzunigai*, *D.rubymacpearsae*, *D.rudyamadori*, *D.sallydaleyae*, *D.sarahoconnorae*, *D.scottmilleri*, *D.shelleymcsweeneyae*, *D.sigifredomarini*, *D.stephmae*, *D.stevestroudi*, *D.susanabramsae*, *D.teremariae*, *D.tiboshartae*, *D.timrichi*, *D.tomdaleyi*, *D.tristanpalolai*, *D.tucuman*, *D.verobrondexae*, *D.virgendelparamo*, *D.weaversway*, *D.yungas*, *D.yvesbraeti*.

## ﻿﻿Introduction

With 366 described species, the genus *Dolichogenidea* Viereck, 1911 is the second most speciose taxon within Microgastrinae parasitoid wasps (Hymenoptera: Braconidae), only surpassed by *Apanteles* (Fernandez-Triana et al. 2020). *Dolichogenidea* was originally described as a subgenus of *Apanteles*, but it was later elevated to generic rank by Mason (1981). Since then, its recognition has been at times controversial (e.g., Mason 1981; van Achterberg 2003; Fernandez-Triana et al. 2014a, 2020), but in the past decade it has been widely accepted as a valid genus by most of the taxonomists working with the group worldwide.

This paper aims to describe a significant number of new species from the Neotropical region. We provide a key, illustrations for all known species, and comments on natural history and DNA barcoding data. Special emphasis is given to the Area de Conservación de Guanacaste, northwestern Costa Rica (ACG), as the present study contributes taxonomically to its ongoing insect biodiversity inventory based on rearing and Malaise trapping (Janzen and Hallwachs 2016). Other areas beyond ACG are also dealt with, although they have not been as comprehensively studied.

## ﻿﻿Materials and methods

In this paper we diagnose and describe all the species of *Dolichogenidea* known from the Neotropical region by using a combination of basic morphology (dichotomous key and brief diagnostic descriptions), DNA barcodes (COI gene, when available), biology (host data and wasp cocoon strategy, when available), and distribution data, following suggestions outlined in Fernandez-Triana (2022).

Morphological terms and measurements mostly follow Huber and Sharkey (1993), Whitfield (1997), Karlsson and Ronquist (2012) and Fernandez-Triana et al. (2014a). The abbreviations T1, T2, and T3 are used for metasomal mediotergites 1, 2, and 3 respectively, and F1–16 refer to flagellomeres 1 to 16.

Species are diagnosed to species groups following some simple characters and then within each group a few additional characters are provided to separate species in that specific group. The resulting diagnostic descriptions are thus based on a relatively small set of morphological characters which vary depending on the species group.

Complete and verbatim label details are provided only for holotypes. Paratypes and other specimens examined are listed only with basic information (country, repository, sex, and voucher codes). All information associated with those specimens can be accessed in the publicly available CNC database (https://www.cnc.agr.gc.ca/taxonomy/TaxonMain.php). A dataset with all details for specimens with available DNA COI sequences in BOLD is available at https://dx.doi.org/10.5883/DS-NEODOLIC.

DNA barcoding was also used to characterize and recognize species. DNA extracts were obtained from single legs using a glass fiber protocol (Ivanova et al. 2006), and total genomic DNA was re-suspended in 30 μl of distilled water. The barcode region, a 658 base pairs (bp) region near the 5’ terminus of the COI gene, was amplified using standard primers following established protocols (e.g., see references in Fernandez-Triana et al. 2014a).

The Barcode Index Number (BIN) was considered to approximately characterize species limits, following the BIN concept detailed in Ratnasingham and Hebert (2013). A Neighbor Joining (NJ) tree with all available sequences of Neotropical *Dolichogenidea* (Suppl. material 1) and with single sequences per BIN were generated using BOLD capabilities.

For additional analyses, best-fit models were identified using ModelFinder (Kalyaanamoorthy et al. 2017), Maximum Likelihood analysis was performed using IQ-TREE 2 (Minh et al. 2020) including ultrafast Bootstrap (Hoang et al. 2018). Bayesian analysis was performed using BEAST X v. 1.10.4 (Suchard et al. 2018) with BEAGLE (Ayres et al. 2012). For those BINs that showed potential BIN-discrepancies, TCS haplotype network analysis was performed using PopART (Snell et al. 2002; Leigh and Bryant 2015). The sequences used were selected based on sequence length which is indicated in the description of the figures depicting the haplotype networks. In the figures, each hatch mark in the network represents a single mutational change; small black dots at nodes indicate missing haplotypes. The diameter of the circles is proportional to the number of specimens sampled per haplotype. We also performed clustering using ASAP (Puillandre et al. 2021) for some selected species

All information for the sequences associated with each specimen barcoded (including primers and trace files) is available on the Barcode of Life Data System (BOLD) (Ratnasingham and Hebert 2007) under the dataset “DS-NEODOLI Neotropical Dolichogenidea species” (https://www.boldsystems.org/index.php/MAS_Management_DataConsole?codes=DS-NEODOLI).

Host data (Lepidoptera species) as well as wasp cocoon strategy (solitary/gregarious) were mostly taken from the website “Dynamic database for an inventory of the macrocaterpillar fauna, and its food plants and parasitoids in ACG databases” (http://janzen.sas.upenn.edu/caterpillars/database.lasso). We caution that considering a wasp species “solitary” when there are only one or two rearing records may be an artifact of their having been just one surviving wasp larva of a gregarious species; e.g., see record DHJPAR0042935, for wasp species *Dolichogenideaalejandromasisi*, where there is just a single cocoon of a species that is notoriously gregarious (https://bench.boldsystems.org/index.php/MAS_DataRetrieval_OpenSpecimen?selectedrecordid=ASHYH693-11).

Although many hosts are identified to species level in that database, for many others the available information only included the genus of Lepidoptera with an interim or provisional species name code (supported by biology or DNA barcoding to be one biological unit); these conventions to record Lepidoptera hosts from ACG have been used in many scientific papers published during the past 10+ years (see Fernandez-Triana et al. 2023 for an explanation on how to interpret those codes).

We also note that, in contrast to parasitoids of macrolepidopterans (e.g., Nymphalidae, Saturniidae, Sphingidae, Geometridae) whose larvae are distinctive and easy to identify in the field, many hosts of *Dolichogenidea* have small and poorly identified larvae often hidden in leaf rolls or nests and therefore subject to misidentification other than when there are so many that some survive to adult status, thereby ensuring the host identification (e.g., 93 rearings of *Dolichochenideaalejandromasisi* from the single species *Antaeotricharenselariana* (Depressariidae) eating 32 species of Fabaceae in ten genera in all ACG habitats).

The majority of the photos were taken with a Keyence VHX-1000 Digital Microscope (Keyence Corporation, Japan), using a lens with a range of 10–130×; multiple images were taken of the structures through the focal plane and then combined to produce a single in-focus image using the software associated with the Keyence System. Some photographs were taken with a Canon EOS-7D Mark 2 (G) (Canon Inc., Japan) using a super-macro lens Canon MP-65 with a Yongnuo professional flash speedlight flashlight installed on a modified microscope stand; multiple images (in raw format .CR2) were taken of a structure through the focal plane, converted to .dng with Adobe DNG converter, then corrected (brightness and contrast) in Adobe Bridge CS4, converted to .tiff images with Adobe Photoshop CS4 and finally combined to produce a single in-focus image using Zerene Stacker (http://zerenesystems.com/cms/stacker). Final images were corrected using GIMP 2.10.12. Images of wasp cocoons and larvae were taken by parataxonomists at ACG with a Canon camera. All plates were prepared using Microsoft PowerPoint 2010 and saved as .TIF files.

Images of one holotype (of a previously described species), deposited in the Natural History Museum (Smithsonian Institution, Washington DC, USA) were accessed through the Primary Type Specimens Catalog of the Department of Entomology Collections (https://collections.nmnh.si.edu/search/ento/). The downloaded images were later combined into plates. Those images were classified in that website as CC0, therefore making them available under the Creative Commons license CC0 1.0 license granting the right to share for personal and educational purposes under the fair use doctrine; in any case, we acknowledge the source of those images here.

All ACG specimens were collected, exported, and DNA barcoded under Costa Rican government permits issued to BioAlfa (R-054-2022-OT-CONAGEBIO; R-019-2019-CONAGEBIO; National Published Decree #41767), JICA-SAPI #0328497 (2014) and DHJ and WH (ACG-PI-036-2013; R-SINAC-ACG-PI-061-2021; Resolución N°001-2004 SINAC; PI-028-2021).

## ﻿﻿Results

This paper deals with 127 species (Table 1). This total includes 125 Neotropical species (102 of them described as new) as well as the only two Nearctic species of *Dolichogenidea* recorded from southern areas in the United States around the Gulf of Mexico (Alabama, Florida, Louisiana, Mississippi, and Texas): *D.acrobasidis* (Muesebeck, 1921) and *D.bushnelli* (Muesebeck, 1933). The Nearctic species were included in the key below in case they are later found to also occur in the Neotropics, especially Mexico or on some Caribbean islands, although at present there is no evidence they occur there.

**Table 1.** List of all known Neotropical *Dolichogenidea* species with associated molecular data (BIN code, when available, or details of partial barcodes), host families and host species, wasp cocoon strategy (S- solitary; G- gregarious), and known distribution by country. [See explanation in Materials and methods about few cases where considering a wasp cocoon as “solitary” may not be accurate].

Species name	Molecular data	Host Family	Host species	S/G	Distribution
*Dolichogenideaaceituno*					Chile
*D.acrobasidis*		Pyralidae, Tortricidae	*Acrobasiscaryae*, *Gretchenabolliana*	S	United States
*D.alanflemingi*	BOLD:AAN2497	Depressariidae	*Antaeotricha* Janzen49, *Antaeotricha* Janzen146	S	Costa Rica, Saint Vincent, Trinidad & Tobago
*D.alejandromarini*	BOLD:AAI9746				Costa Rica
*D.alejandromasisi*	BOLD:ABX6174	Depressariidae	*Antaeotricharenselariana*	G	Costa Rica
*D.alerce*	BOLD:AAH1316				Chile
*D.alexamasisae*	BOLD:AAF5364	Crambidae	*Herpetogramma* Janzen04, *Rhectocraspeda* Solis05	S	Costa Rica, Ecuador, Venezuela
*D.alexandrei*					Guatemala
*D.alixhamiltonae*	BOLD:AAL2285	Thyrididae	*Banisia myrsusalisDHJ01*	S	Costa Rica
*D.amazonas*					Peru
*D.anacamposae*	BOLD:AAE8612	Tortricidae	*Olethreutes* Brown22, *Olethreutes* Janzen323	S	Costa Rica
*D.andreamezae*	BOLD:ABA7252	Erebidae	*Rivula* Poole03	S	Costa Rica
*D.angelagonzalezae*	BOLD:AAL2298	Choreutidae	*Brenthia* Janzen12	S	Costa Rica
*D.angelsolisi*	BOLD:ACI3413	Immidae	immidJanzen01 Janzen26	S	Costa Rica
*D.anikenpalolae*	BOLD:ABY1812	Crambidae	spiloBioLep01 BioLep379	S	Costa Rica
*D.anniapicadoae*	BOLD:ABY7999	Crambidae	*Ategumialotanalis*	S	Costa Rica
*D.annlisterudae*	BOLD:AAB5549, BOLD:ACF0272				Costa Rica
*D.annychaverae*	BOLD:AAD5258				Costa Rica
*D.antioquia*					Colombia
*D.antjevirkusae*	BOLD:AAM5849				Costa Rica
*D.arenal*					Costa Rica
*D.bernardoespinozai*	BOLD:AAE8596				Costa Rica
*D.beryllacosteae*	BOLD:AAM1098	Thyrididae	*Microscahedialis*	G	Costa Rica
*D.bradzlotnicki*	BOLD:ACC1295	Depressariidae	*Chlamydastisvividella*, *Stenoma* Janzen199	S	Costa Rica
*D.bushnelli*		Pyralidae, Tortricidae	*Dioryctriaabietella*, *Dioryctriaclarioralis*, *Dioryctriadisclusa*, *Rhyacioniabushnelli*, *Rhyacioniapasadenana*	S	United States
*D.caldas*					Colombia
*D.carlosalvaradoi*	BOLD:AAM5848				Costa Rica
*D.carlosmanuelrodriguezi*	BOLD:ABZ4155	Depressariidae	*Antaeotrichaspurca*	S	Costa Rica
*D.carlosviquezi*					Costa Rica
*D.cedenoae*	BOLD:ABY3724	Depressariidae	elachJanzen01 Janzen196, elachJanzen01 Janzen397, *Antaeotricha* Janzen126	G	Costa Rica
*D.chichicastenango*					Guatemala
*D.christinaagapakisae*	BOLD:AAI9755	Depressariidae, Gelechiidae	*Gonionota* Janzen22, gelJanzen01 Janzen23	S	Costa Rica
*D.claudiadoblesae*	BOLD:AAD2236				Costa Rica
*D.croceicornis*		Crambidae	*Microthyrisanormalis*	S	Peru
*D.dole*	BOLD:AAM5739				Costa Rica
*D.encruzilhada*					Brazil
*D.ensiger*	BOLD:AAA3764	Crambidae, Tortricidae	*Fissicrambusmutabilis*, *Neodactriazeellus*, *Choristoneurafreemani*, *Epiblemastrenuana*	?S	Canada, Costa Rica, United States
*D.ericpalolai*	BOLD:AAF7717				Costa Rica
*D.ericsimoni*					Chile
*D.escobarae*					Brazil
*D.evadne*					Juan Fernández Islands
*D.felipechavarriai*	BOLD:ACC4119	Depressariidae	*Gonionota* Janzen116	S	Costa Rica
*D.frankjoycei*	BOLD:ABA3469	Tortricidae	*Platynota* rostranaDHJ01, *Platynota* rostranaDHJ02	S	Costa Rica
*D.fredhicksi*	BOLD:AAK2061	Depressariidae	*Anadasmus* Janzen25, *Stenoma* Janzen27	G	Costa Rica
*D.gelechiidivoris*	BOLD:AAM4042	Gelechiidae	*Phthorimaeaoperculella*, *Keiferialycopersicella*, *Tutaabsoluta*	S	Algeria, Chile, Colombia, Peru, Spain, Venezuela
*D.genuarnunezi*	BOLD:ACC1300	Depressariidae	*Antaeotrichaphaeoneura*	G	Costa Rica
*D.hedyleptae*		Crambidae	*Marucavitrata*, *Omiodesindicata*	?G	Puerto Rico
*D.helenedumasae*	BOLD:ABZ4155				Brazil, French Guiana
*D.heredia*					Costa Rica
*D.homoeosomae*	Partial sequences (425 bp)	Pyralidae	*Homoeosomaelectellum*	S	Cuba, Canada, United States
*D.ingredolsonae*	BOLD:AAM5850				Costa Rica
*D.isabelleae*					Ecuador
*D.isidrochaconi*	BOLD:AAB9372				Costa Rica
*D.jaimelewisi*	BOLD:AAM5738	Crambidae	*Herpetogrammasalbialis*, *Herpetogramma* Dapkey27, spiloBioLep01 BioLep617	G	Costa Rica
*D.jasonkelleyi*	BOLD:AAI9747				Costa Rica
*D.jennyphillipsae*	BOLD:AAM5088				Costa Rica
*D.jessiehillae*	BOLD:AAM5851				Costa Rica
*D.johnrobinsoni*	BOLD:ACF0267				Costa Rica
*D.jorgecarvajali*	BOLD:AAM5847				Costa Rica
*D.jorgecortesi*	BOLD:AAM5846				Costa Rica
*D.josealfredohernandezi*	BOLD:ABA9255	Depressariidae	*Stenoma* Janzen99	S	Costa Rica
*D.josephfridmani*	BOLD:AAE8602				Costa Rica
*D.joshdarfleri*	BOLD:AAC7481				Costa Rica
*D.juanmatai*	BOLD:AAM5740				Costa Rica
*D.junhyongkimi*	BOLD:AAD6850				Costa Rica
*D.kasiiya*	BOLD:AAM5750				Costa Rica
*D.katiemccluskeyae*	BOLD:ACE8228				Costa Rica, Honduras
*D.kenzabaddouae*	BOLD:AAY4695	Depressariidae	*Antaeotricha* Janzen221	S	Costa Rica
*D.lacochaparamo*					Colombia
*D.leahdennisae*	BOLD:AAJ1396				Costa Rica
*D.limoncocha*					Ecuador
*D.luishamiltoni*	BOLD:AAT8840	Gelechiidae	gelJanzen01 Janzen394	S	Costa Rica
*D.luzmariaromeroae*	BOLD:ABX5620	Pyralidae	phyBioLep01 BioLep758	S	Costa Rica
*D.machupichu*					Peru
*D.mariabustosae*	BOLD:AAN2496	Gelechiidae	gelJanzen01 Janzen319	S	Costa Rica
*D.mehdirheljari*	BOLD:AAM5852				Costa Rica
*D.melaniamunozae*	BOLD:AAB5701	Depressariidae	*Cerconotarecurvella*	S	Costa Rica
*D.moniqueae*					Chile
*D.moniquegilbertae*	BOLD:AAX8653				Costa Rica, Mexico
*D.monocavus*					Costa Rica
*D.ninamasisae*	BOLD:AAY4690	Tortricidae	*Megalotacrassana*, *Megalotaspinulosa*	S	Costa Rica
*D.nothofagus*					Chile
*D.oiketicus*		Psychidae	*Oiketicuskirbyi*	S	Trinidad & Tobago
*D.palenque*					Ecuador
*D.papallacta*					Ecuador
*D.parallelis*					Saint Vincent
*D.paulfryi*	BOLD:ACF2929				Costa Rica
*D.pedroleoni*	BOLD:AAB4946	Mimallonidae	*Eadmuna* Janzen01	G	Costa Rica
*D.phthorimaeae*		Gelechiidae	*Keiferiaglochinella*, *Keiferiainconspicuella*, *Keiferialycopersicella*	S	Canada, Honduras, United States
*D.politiventris*					Colombia, Dominican Republic, Puerto Rico, Saint Vincent, Trinidad & Tobago
*D.puschendorfi*	BOLD:AAM5853				Costa Rica
*D.putumayo*					Colombia
*D.puyo*					Ecuador
*D.rexhamiltoni*	BOLD:AAL2287				Costa Rica
*D.richardashleyi*	BOLD:ABX6267	Gelechiidae	gelJanzen01 Janzen349	S	Costa Rica
*D.ritaashleyae*	BOLD:ABX6267				Costa Rica
*D.robertofernandezi*	BOLD:AAC8392				Costa Rica
*D.robinsherwoodae*	BOLD:ABX5195	Depressariidae	*Antaeotricha* Janzen221	S	Costa Rica
*D.robmacewani*					Brazil
*D.robpringlei*		Thyrididae	*Collinsaferreiceps*	G	Costa Rica
*D.rociocordobae*	BOLD:ACJ2777	Gelechiidae	*Dichomeris* Janzen273, *Dichomeris* Janzen703	S	Costa Rica
*D.rodrigogamezi*	BOLD:AAM5843				Costa Rica
*D.rogerblancoi*	BOLD:AAL2325	Depressariidae	*Antaeotricha* radicalisDHJ01	G	Costa Rica
*D.ronaldzunigai*	BOLD:AAT8860	Depressariidae	*Chlamydastismontywoodi*, *Chlamydastistryphon*, *Chlamydastisvividella*, *Stenoma* Janzen199, elachJanzen01 Janzen693	S	Costa Rica
*D.rosamatarritae*	BOLD:ABY5258	Choreutidae, Gelechiidae, Depressariidae	*Brenthia* Janzen05, *Stenoma* Phillips543, gelJanzen01 Janzen16	S	Costa Rica
*D.rubymacpearsae*					Ecuador, Peru
*D.rudyamadori*	BOLD:AAX8664				Costa Rica
*D.sallydaleyae*	BOLD:ACM2280	Depressariidae	elachBioLep01 BioLep286	S	Costa Rica
*D.sarahoconnorae*	BOLD:ABX6008	Thyrididae	*Microscahedialis*, *Microscapolychloralis*, siculoJanzen01 biolep03, siculoJanzen01 Janzen05	G	Costa Rica
*D.scottmilleri*	BOLD:AAC2174, BOLD:ACE8823	Thyrididae	*Microscapaullula*	G	Costa Rica
*D.shelleymcsweeneyae*	BOLD:AAM5736				Costa Rica
*D.sigifredomarini*	BOLD:ACI3397	Depressariidae		S	Costa Rica, French Guiana
*D.stephmae*					Brazil
*D.stevestroudi*	BOLD:ACB1629	Gelechiidae	gelJanzen01 Janzen22	S	Costa Rica
*D.susanabramsae*	BOLD:AAI6323	Gelechiidae	*Dichomeris* designatellaDHJ04	S	Costa Rica
*D.teremariae*	BOLD:AAM5842				Costa Rica, Honduras
*D.tiboshartae*	BOLD:AAC5949	Depressariidae, Gelechiidae	*Dichomeris* Janzen76, elachJanzen01 Janzen409, gelJanzen01 Janzen116	S	Costa Rica
*D.timrichi*	BOLD:AAJ1390				Costa Rica
*D.tomdaleyi*	BOLD:AAD8952	Depressariidae, Crambidae	*Antaeotrichaincrassata*, *Antaeotrichacirrhoxantha*DHJ02, *Antaeotrichasimilis*EPR01, *Antaeotrichasimilis*EPR02, *Antaeotricha* BioLep46, *Antaeotricha* Janzen23, *Antaeotricha* Janzen31, *Antaeotricha* Janzen77, *Antaeotricha* Janzen106, *Antaeotricha* Janzen146, *Antaeotricha* Janzen290, *Antaeotricha* Janzen292DHJ0, *Antaeotricha* Janzen364, *Antaeotricha* Philips01, *Chlamydastis* Janzen04, *Stenoma* Janzen18, *Stenoma* Janzen58, *Stenoma* Janzen199, *Stenoma* Janzen699	S	Costa Rica
*D.tristanpalolai*	BOLD:AAI9740				Costa Rica
*D.tucuman*					Argentina
*D.verobrondexae*					Venezuela
*D.virgendelparamo*					Ecuador
*D.weaversway*		Gelechiidae	*Telphusa* BioLep476		Costa Rica
*D.yeimycedenoae*	BOLD:ABX6267				Costa Rica
*D.yungas*					Bolivia
*D.yvesbraeti*					French Guiana

Of the 25 species previously described, seven had been previously placed in different genera but are here transferred to *Dolichogenidea* as new combinations: *D.croceicornis* (Muesebeck, 1958) which was described within *Apanteles* (based on holotype having vannal lobe entirely setose); and species formerly placed within *Exoryza* (see below for rationale to synonymize this genus within *Dolichogenidea*): *D.mariabustosae* (Fernandez-Triana, 2016), *D.monocavus* (Valerio & Whitfield, 2004), *D.richardashleyi* (Fernandez-Triana, 2016), *D.ritaashleyae* (Fernandez-Triana, 2016), *D.rosamatarritae* (Fernandez-Triana, 2016) and *D.yeimycedenoae* (Fernandez-Triana, 2016). A special situation arises with the species *Exoryzayeimycedenoae* Fernandez-Triana, 2016 which, when here transferred to *Dolichogenideayeimycedenoae* (Fernandez-Triana, 2016), becomes a senior secondary homonym of *Dolichogenideayeimycedenoae* Fernandez-Triana & Boudreault, 2019; because the latter name now becomes a junior secondary homonym, that species would need a replacement name. Therefore, here we propose *Dolichogenideacedenoae* Fernandez-Triana & Boudreault, nom. nov. as a replacement name for *Dolichogenideayeimycedenoae* Fernandez-Triana & Boudreault, 2019.

The status of *Exoryza* as a valid genus, separate from *Dolichogenidea*, has been questioned by many authors (Valerio et al. 2004; Rousse and Gupta 2013; Fernandez-Triana et al. 2014a, 2016, 2020). The distinction may be particularly difficult because many species of *Dolichogenidea* have T1 and T2 strongly sculptured and T2 with a similar shape as that described for *Exoryza* (Mason 1981). Molecular data (DNA barcodes) do not support the separation of these two genera, but retrieve *Exoryza* as one group within *Dolichogenidea* (e.g., Fig. 2; see also Suppl. material 1). Here we formally synonymize *Exoryza* under *Dolichogenidea*. This new synonymy affects the six Neotropical species mentioned above as well as nine other species from other biogeographical regions formerly placed within *Exoryza* which are here also transferred as new combinations: *Dolichogenideaasotae* (Watanabe, 1932), *D.belippicola* (Liu & You, 1988), *D.hylas* (Wilkinson, 1932), *D.megagaster* (de Saeger, 1944), *D.minnesota* (Mason, 1981), *D.oryzae* Walker, 1994, *D.reticarina* (Song & Chen, 2003), *D.safranum* (Rousse & Gupta, 2013), and *D.schoenobii* (Wilkinson, 1932).

A total of 73 species are here described as new from Costa Rica, 71 of them from ACG. When accounting for additional species previously described (Valerio et al. 2004; Fernandez-Triana et al. 2016, 2019), there are now 87 *Dolichogenidea* species recorded from Costa Rica, 84 of them from ACG alone.

The rest of the Neotropical region has barely been studied, as we record here only a few species from other countries, Ecuador (8 species), Chile (7, including one species from Juan Fernandez Islands), Brazil and Peru (5), French Guiana, Honduras, Saint Vincent, Trinidad & Tobago, and Venezuela (3), Guatemala and Puerto Rico (2), Argentina, Bolivia, Cuba, Dominican Republic, and Mexico (1). Therefore, the 124 Neotropical species of *Dolichogenidea* accounted for within this paper represent only a small fraction of the actual species richness in the region. We assume that there are hundreds of species remaining undescribed in collections and in nature, especially from South America.

Including the 102 new taxa described here, *Dolichogenidea* now comprises 468 species. The actual diversity of the genus was recently estimated to be at least 700 species (Fernandez-Triana et al. 2020) but this now seems like a rather severe underestimate.

### ﻿﻿Morphology and species concepts used in this paper

Rather few taxonomic publications specify the species concept used (Williams and Ebach 2020), therefore we would like to spend a bit of time explaining how the different data sources were used to generate species hypotheses for Neotropical *Dolichogenidea* and why it is important to incorporate as many sources of information as possible. We used an integrative taxonomy approach where species were recognized, delimited and diagnosed using a combination of morphological, molecular and biological traits – insofar as these data were available. Morphological data are included in a dichotomous key, species diagnostic descriptions, and comprehensive illustrations for all 127 species dealt with in this review. Molecular data included DNA barcodes (complete or partial sequences), which were available for 67.7% of all Neotropical species. Biological data included parasitoid strategy (solitary or gregarious) and host-parasitoid associations, which were available for 41.9% of all Neotropical species. Geographical distribution was not used as a criterion to separate species but it was informative, particularly in deciding which described species should be considered and whether species were conspecific with existing types (it may also be that two or more “conspecifics” are found later to be reliably distinguished by geography, e.g., upper elevations versus adjacent lowlands, as it has been the case with other ACG groups, e.g., Smith et al. 2023).

Morphological data, especially in the dichotomous key and species diagnoses, were intended to provide a foundation for dealing with and incorporating species described in the future. As more species are discovered and described, they can be added to the key, and some of the species diagnoses may need to be modified to accommodate additional species. Morphology also allows us to include all known species in our taxonomic framework, including those lacking molecular and biological data at present.

**Figure 1.**
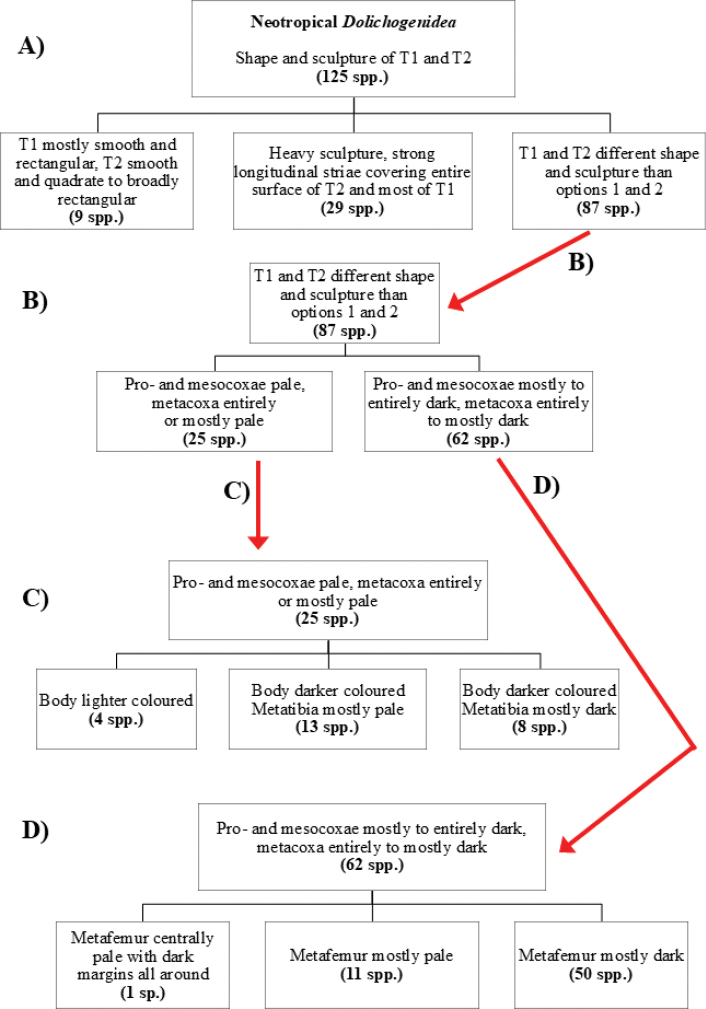
Simplified depiction of characters used to separate all Neotropical species of *Dolichogenidea***A** separation of species in three main groups **B** further separation of one of the main groups in two subgroups **C, D** additional separation of some subgroups into smaller units. The characters shown are simplified and abbreviated, for complete details the dichotomous key must be consulted.

At the beginning of the work for this revision, a number of morphological characters were selected, and we started scoring them for several species. Some species were very similar morphologically, suggesting potential complexes of cryptic species, as has been the case with many other ACG sympatric species. Evaluating those with more detailed analyses (e.g., morphometrics) was not possible because most of the species were not represented by enough specimens to properly assess morphological variability. Additionally, some characters were difficult to measure properly (e.g., the length of T1, the width of the posterior margin of T1, the length of the setose part of the ovipositor sheath), and thus are liable to different interpretations, with resulting measurements not being accurate enough to be useful. Therefore, we decided to use a simplified approach, where species were first grouped based on characters which were simple to assess and score and then, within each of those groups, additional characters were used to recognize individual species. It must be stressed that many of the morphological groupings are not thought to be monophyletic but are only used as a way to organize the work of recognizing species. For example, just by assessing the shape and sculpture of T1 and T2 it is possible to separate all Neotropical species of *Dolichogenidea* in to three groups (Fig. 1A). The first group is characterized by having T1 mostly smooth and rectangular, and T2 smooth and quadrate to broadly rectangular; it includes nine species so far recorded from the Neotropics and it certainly appears to represent a monophyletic group, supported by morphology, DNA and biology (Fernandez-Triana et al. 2019). The second group includes 29 species characterized by having T1 and T2 strongly sculptured with longitudinal striae and with T2 subquadrate to rectangular (or, in a few cases, transverse); it can be divided further into two distinctive subgroups, one of them certainly monophyletic and comprising what until now was considered to be the genus *Exoryza* (Fernandez-Triana et al. 2016) while the other subgroup is probably not monophyletic but still recognizable because of the distinctive shape and relatively coarse sculpture of T1 and T2 (see details in the dichotomous key below). The third group is the largest with 87 species and it is almost certainly not monophyletic. The third group can be separate into two subgroups based on the color of the coxae (Fig. 1B). And these two subgroups can be separated further based on body color, leg color, T1 shape, ovipositor sheath length, or other unique characters (e.g., Fig. 1C, D). The resulting key uses a relatively small set of characters which separate several groups, and then all species within each group are further diagnosed by adding additional characters (which work at that level but may not work for another group).

This “modular approach” considerably sped up the work of diagnosing and describing the taxa, but it also meant that the diagnostic descriptions provided do not include the same set of characters assessed for every species: they only include characters needed for a specific group. It can be argued that, when future studies find additional new species of Neotropical *Dolichogenidea*, it is likely that both the dichotomous key and the diagnostic descriptions will need to be revised, modified or expanded accordingly, perhaps in some cases significantly. But the same will happen when more DNA barcodes are obtained, or new host-parasitoid associations are discovered. Therefore, the present work must be taken as a first approximation, which can/should be improved as knowledge advances.

For 11 species (8.7%) morphology alone was insufficient or very subjective, and DNA barcodes were incorporated into the key as the only available or the most reliable way to separate two species (e.g., see couplets 5, 20, 21, 38, 64, and 68 in the key below). Additionally, for at least another eight species (6.3%) the morphological characters provided in the key are either difficult to assess or interpret and may not be distinctive enough to clearly and easily differentiate species (e.g., see couplets 9, 60, 77 and 86 in the key below). Therefore, morphology alone is not sufficient (or it is very difficult to use) for at least 15% of all species keyed out in the dichotomous key we provide.

Despite its shortcomings for species diagnosis, morphology allows us to include all Neotropical species of *Dolichogenidea* known at present into a common framework, building a foundation upon which to improve as more molecular and biological data become available and new species are discovered.

### ﻿﻿DNA barcoding and the molecular identification of Neotropical Dolichogenidea

**Figure 2.**
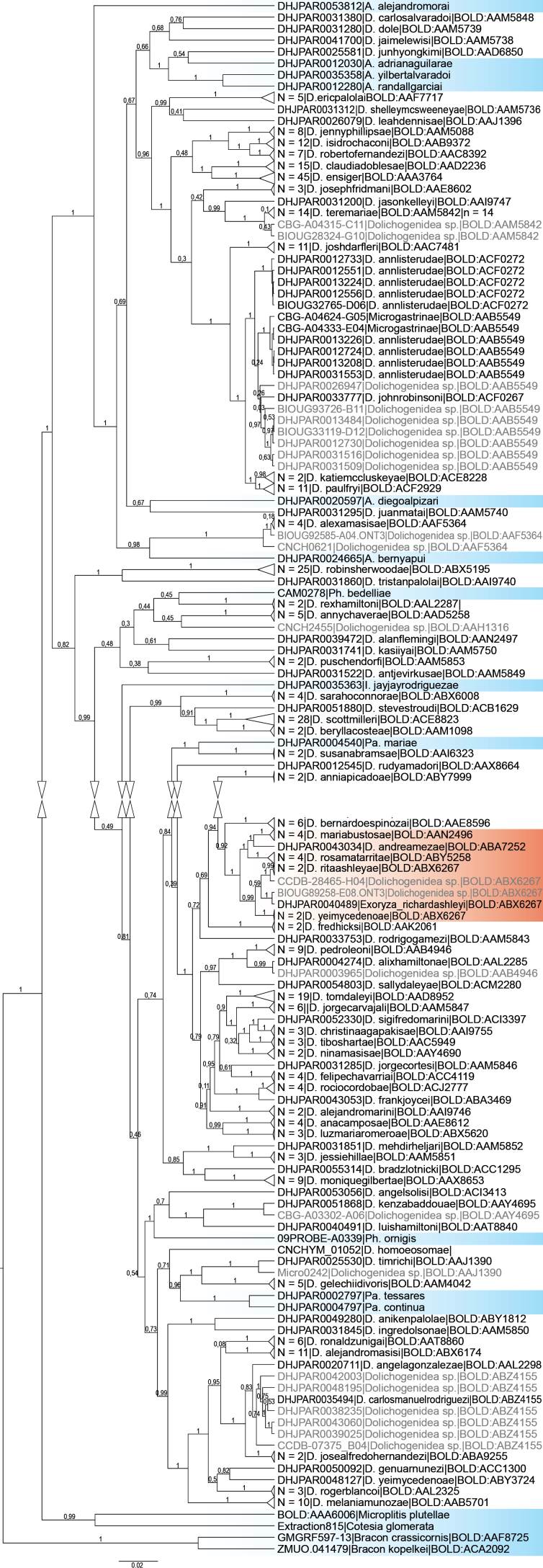
Bayesian inference consensus topology of the COI barcoding region of the *Dolichogenidea* species dataset, including additional outgroups. Triangles indicate collapsed branches. Branch labels represent p-values. Pale blue coloration indicates species from other genera and outgroups, orange coloration indicates the cluster with species that were formerly part of *Exoryza*. The sequences and BEAST settings used can be retrieved from Suppl. material 1.

Bayesian (Fig. 2; see also Suppl. material 1) and Maximum Likelihood (Suppl. material 2) analyses did not recover *Dolichogenidea* as monophyletic. While our analyses have the limitation of including only a single gene, more comprehensive molecular analyses (e.g., Parks 2018’s five-gene molecular phylogeny; ongoing research based on data from Jasso-Martinez et al. 2022’s ultra-conserved elements) have also found the genus to be polyphyletic. The difficulties to characterize *Dolichogenidea*, both morphologically and molecularly, and to separate it from putatively related genera (such as some *Pholetesor*, *Apanteles*, and *Parapanteles*) have long been recognized and extensively discussed (e.g., Fernandez-Triana et al. 2014a, 2016, 2020; Parks 2018). Regardless of the current challenges to unambiguously recognize the genus, most species with molecular data available can be reliably identified using DNA barcodes.

The current coverage of DNA barcodes for Neotropical *Dolichogenidea* is fairly comprehensive, with 68.5% of the species being represented in BOLD as of April 2024 by at least 83 BINs. A total of 84 species (67.7%) have barcode compliant sequences while another one, *D.homoeosomae*, has two almost complete sequences of 425 bp and it is also included in the discussion below.

While most species had a unique correspondence with a single BIN, seven cases included either multiple BINs per single species or more than one species within a BIN, they are briefly discussed below.

**Figure 3.**
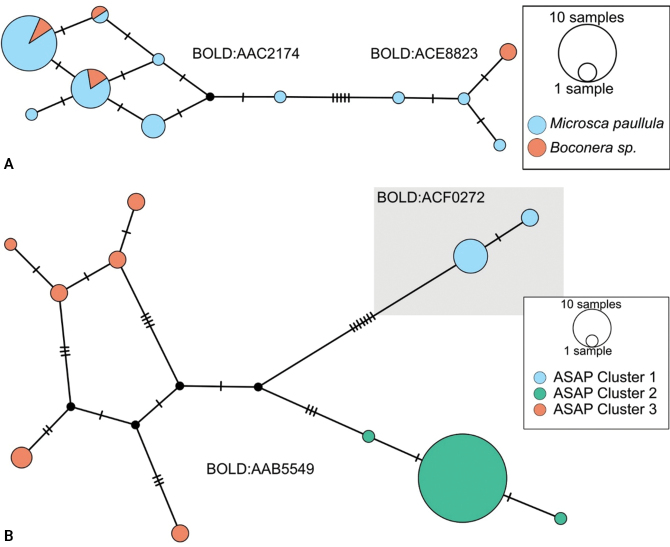
**A** TCS haplotype network of BINs BOLD:AAC2174 and BOLD:ACE8823, sequence length for analysis: 561 bp. Host species are indicated via coloration **B** TCS haplotype network of BINs BOLD:AAB5549 and BOLD:ACF2930, sequence length for analysis: 626 bp. The different molecular clusters resulting from ASAP analysis are indicated via coloration.

*Dolichogenideascottmilleri* sequences are included in BINs BOLD:AAC2174 and BOLD:ACE8823 (Fig. 3A), with a minimum p-distance between them of 1.05% (5 bp). Specimens from both BINs were reared from what appears to be the same Lepidoptera hosts, and we could not find any morphological difference to separate them, therefore we consider these two BINs to represent the same species.

Sequences of *D.annlisterudae* are included in BINs BOLD:AAB5549 and BOLD:ACF0272 (Fig. 3B), with a minimum p-distance of 1.08% (~ 7 bp). BOLD:AAB5549 showed some indicators of not being well separated from neighboring BINs with the maximum within-BIN p-distance of 1.77% being higher than the minimum p-distance to the Nearest Neighbor BIN (BOLD:ACF0267) of 1.28%. We performed ASAP clustering and retrieved three different clusters which are also represented in the TCS haplotype network analysis (Fig. 3B). However, we could not find strong morphological traits to separate them, only minor differences between the two BINs (see detailed discussion of those characters under the species description below). And there is no biological data available, as all specimens known from this species have only been collected with Malaise traps. That combined with the molecular differences being rather small (1.28%), does not provide any support to separate the specimens, therefore we here consider the two BINs/ three ASAP clusters to represent the same species.

**Figure 4.**
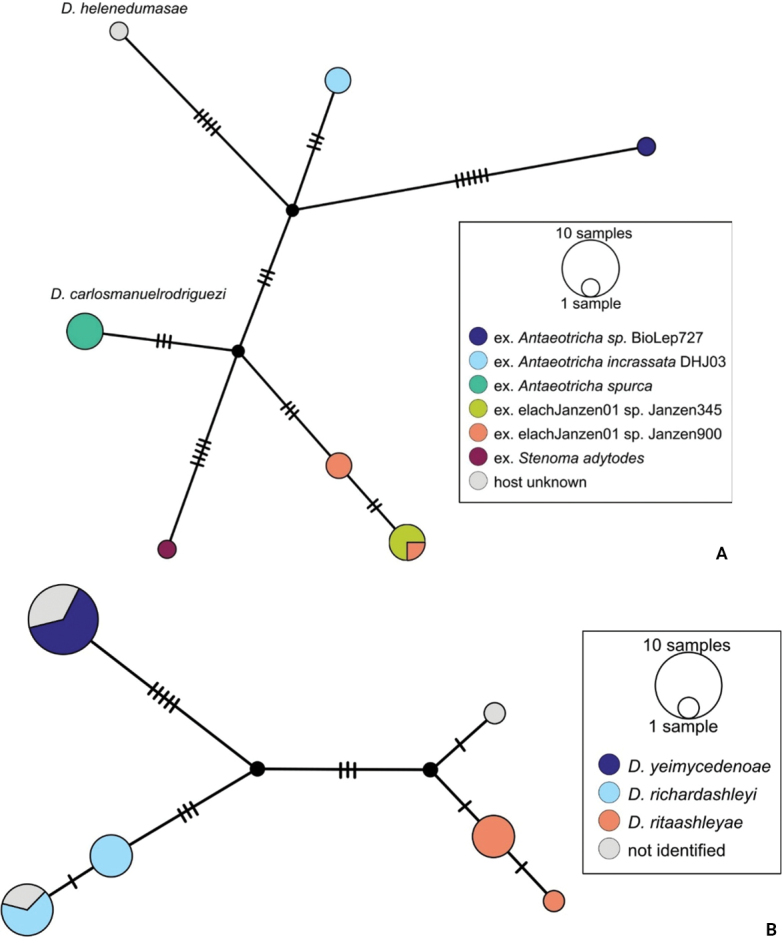
**A** TCS haplotype network of BINBOLD:ABZ4155, sequence length for analysis: 632 bp. Host species are indicated via coloration **B** TCS haplotype network of BINBOLD:ABX6267, sequence length for analysis: 651 bp. The different molecular BINs are indicated via coloration.

BINBOLD:ABZ4155 is shared by two species, *D.carlosmanuelrodriguezi* (Costa Rica) and *D.helenedumasae* (Brazil, French Guiana). It has been discussed before (Fernandez-Triana et al. 2019: 100) that this BIN contains more than one species, including some still undescribed from Costa Rica, ACG (Fig. 4A). The full barcode sequence of a paratype of *helenedumasae* (only specimen with available sequence for this species) is > 1.5% (~ 10 bp) different from the available sequences of *carlosmanuelrodriguezi* and the remaining (undescribed) Costa Rican species within this BIN. There are also morphological differences between these two species (see details in key below), which we consider to be distinct.

BINBOLD:ABX6267 is shared by three *Dolichogenidea* species, all of them from ACG, *richardashleyi*, *ritaashleyae* and *yeimycedenoae* (Fig. 4B). The differences between these three species ranges between 1.05 and 1.20% (7–8 bp) and they all group in three distinct clusters in the NJ tree. This BIN is also comparatively very close to BOLD:ABY5258, which only contains *D.rosamatarritae*, another ACG species. The difference between these two BINs is 1.93% (~ 12 bp) compared to a within-BIN maximum p-distance of 1.61% for BOLD:ABX6267 and 0.77% for BOLD:ABY5258. However, there are consistent morphological differences between these four species (Fernandez-Triana et al. 2016, see also key below), and we consider all four to be distinct.

**Figure 5.**
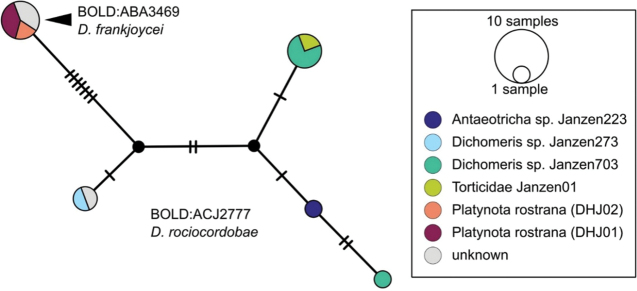
TCS haplotype network of BINs BOLD:ABA3469 and BOLD:ACJ2777, sequence length for analysis: 654 bp. Host species are indicated via coloration.

In addition to the cases discussed above, several other BINs may or may not represent a single species and more comprehensive studies would be needed to conclude, ideally including other molecular markers, more morphological analyses, and/or additional biological information. For example, *Dolichogenidearociocordobae* and *D.frankjoycei*, are here considered as distinct because they have been reared from different host species, but the morphological differences are very small and subtle (see couplet 21 in the key below) and DNA barcodes, considered in BOLD as two separate BINs are only 1.01% different (~ 6 bp) (Fig. 5).

It could be argued that, in the near future, DNA barcoding data would be the most efficient way to identify species. However, at present DNA barcoding alone is neither sufficient (as one third of the *Dolichogenieda* here treated do not have available barcodes) nor entirely accurate (as 8.3% of the species with molecular data cannot be unambiguously identified by using BINs). Therefore, morphological and biological data must also be considered to differentiate species.

### ﻿﻿Parasitoid biology data

Biological data for Neotropical *Dolichogenidea* are currently incomplete. Only 52 of the species (41.9%) have some sort of host information associated; therefore, the comments below can only be seen as preliminary and may change when biological data are known for more species and more rearings in the region.

There seems to be a prevalence of wasp species with solitary cocoons (76.9%), more than three times the species with gregarious cocoons (23.1%). This is in stark contrast with data for the related genera *Apanteles* (which had 42% of its Mesoamerican species with solitary cocoons versus 58% gregarious, see Fernandez-Triana et al. 2014a) and *Alphomelon* (48% of New World species solitary, 52% gregarious, see Fernandez-Triana et al. 2023).

A total of 11 Lepidoptera families were parasitized by Neotropical *Dolichogenidea*. The most represented group was the microlepidopteran Depressariidae (33.9% of all host data available), followed by Gelechiidae (16.9%), Crambidae (13.6%), Tortricidae (10.2%), Thyrididae (8.5%) and Pyralidae (6.7%). Again, this is in strong contrast with the (morphologically closely related) genus *Apanteles*, which in Mesoamerica has been recorded from at least 14 families of Lepidoptera but with a completely different arrangement of groups (Fernandez-Triana et al. 2014a): Hesperiidae (33%), Elachistidae (26%), Crambidae (21%), Pyralidae (4%), Choreutidae (3%) and Gelechiidae (3%). The main two groups parasitized by *Apanteles* (hesperiids and elachistids) are not even found among the known host records of *Dolichogenidea* in the Neotropics. Although it must be noted that some of the “Gelechiidae” from the *Apanteles* paper could now be placed in Depressariidae, that still would not make any major difference as that is the family with the smallest percentage of parasitism by *Apanteles*, whereas it is the largest group attacked by *Dolichogenidea*. Although more host data would be useful, especially for *Dolichogenidea*, there seems to be a very distinctive separation between the two parasitoid genera regarding host families used and solitary/gregarious wasp cocoons.

With respect to parasitoid biology, the only similarity between Neotropical species of *Dolichogenidea* and *Apanteles* seems to be the fact that both genera are mostly monophagous or at most oligophagous. Out of the 52 *Dolichogenidea* species with host data available, 29 (55.8%) are only known to parasitize a single host species, whereas 12 (23.1%) parasitize two host species and five (9.6%) parasitize three hosts. Another five wasp species (9.6%) attack either four or five different hosts. Only a single species, *D.tomdaleyi*, has been recorded parasitizing up to 19 different hosts, but even there that list includes 14 putative species of *Antaeotricha* and four putative species of *Stenoma* (both moth genera belonging to the family Depressariidae). In almost all cases, the hosts species belong to one genus (or related genera) in the same Lepidoptera family. Only six *Dolichogenidea* have host records that include two Lepidoptera families and *D.rosamatarritae* hosts include three different families. One caveat is that most of the biological information discussed above is based on the extensive rearing data available from ACG material, while data from other Neotropical areas are neither complete nor comprehensive.

### ﻿﻿Preliminary data on elevational and ecosystem distribution of species

Neotropical *Dolichogenidea* are here recorded from sea level up to 4,100 m. Although the data available at present are insufficient to draw strong conclusions, the distribution of species appears to be elevationally segregated. Most of the species dealt with in this paper are found between 400–1,500 m; however, a few species have only been found at higher elevations, including a few examples found at more than 3,000 m (Costa Rica) and 4,000–4,100 m in the Andes (South America). Among the species recorded from ACG (the only area where more comprehensive data is available), most species seem to be restricted to one specific ecosystem (dry forest, rain forest or cloud forest).

### ﻿﻿Key to *Dolichogenidea* from the Neotropical region

After morphological characters, basic data on species distribution (countries), and, if available or known, biology (solitary/gregarious parasitoid and general info on host), and DNA barcodes (BIN data) are included. The additional data are presented between brackets, and are meant to support and complement (but not supplant) the morphological identification of species. More comprehensive details on species distribution, biology and molecular data are discussed under each species treatment.

**Table:** 

1	T2 shape ranging from almost quadrate to broadly rectangular, its width at posterior margin 1.3–2.2× its length centrally, rarely 2.3–2.4×; ***and*** T2 smooth; ***and*** ovipositor sheath 1.8–2.5× as long as metatibia length (*carlosmanuelrodriguezi* group)	**2**
–	T2 shape ranging from transverse to (rarely) broadly rectangular; its width at posterior margin usually 3.0–5.0× its length centrally, ***if*** rarely 2.4–2.9×, ***then*** T2 strongly sculptured with longitudinal striae ***or*** ovipositor sheaths < 1.2× metatibia length; ovipositor sheath usually 0.5–1.5× as long as metatibia length, ***if*** very rarely up to 2.0×, ***then*** T2 strongly sculptured	**10**
2(1)	Comparatively larger size, body length 4.80–4.90 mm, fore wing length 4.70–4.80 mm, ovipositor sheath length 3.00–3.40 mm; ***and*** metatrochanter and metatrochantellus entirely black [Costa Rica. Solitary, host: *Cerconotarecurvella* (Depressariidae). BINBOLD:AAB5701]	***D.melaniamunozae* Fernandez-Triana & Boudreault, 2019**
–	Comparatively smaller size, body length < 4.00 mm (rarely 4.20 mm), fore wing length < 3.70 mm (rarely 4.20 mm), ovipositor sheath length, 3.10 mm (rarely 3.20 mm); ***and*** metatrochanter and/or metatrochantellus at least partially yellow	**3**
3(2)	Mesofemur entirely yellow, metafemur almost entirely yellow (except for small, dark spot on anterior 0.1–0.2)	**4**
–	Mesofemur partially dark brown (rarely mostly yellow with small, dark spots), metafemur mostly to entirely dark brown to black	**6**
4(3)	Comparatively larger size, body length 4.10–4.20 mm, fore wing length 4.20–4.30 mm, ovipositor sheath length 3.00–3.20 mm [Brazil, French Guiana. BINBOLD:ABZ4155]	***D.helenedumasae* Fernandez-Triana & Boudreault, sp. nov.**
–	Comparatively smaller size, body length 3.70–3.90 mm, fore wing length 3.70 mm, ovipositor sheath length 2.60–2.90 mm [Costa Rica]	**5**
5(4)	Slightly smaller specimens (body length 3.70 mm; ovipositor sheath 2.60 mm); T2 posterior width 2.0 × T2 length medially; F2 1.6× as long as F14 [21 DNA barcode diagnostic characters: 31T, 37A, 55G, 67A, 70A, 79A, 88A, 109A, 139A, 190C, 277A, 290G, 316G, 322G, 370T, 386T, 418T, 436T, 460A, 496G, 556A] [Costa Rica. Solitary, host: *Antaeotrichaspurca* (Depressariidae). BINBOLD:ABZ4155]	***D.carlosmanuelrodriguezi* Fernandez-Triana & Boudreault, 2019**
–	Slightly larger specimens (body length 3.90 mm; ovipositor sheath 2.90 mm); T2 posterior width 1.8 × T2 length medially; F2 1.7–1.9× as long as F14 [21 DNA barcode diagnostic characters: 31A, 37G, 55A, 67T, 70G, 79T, 88T, 109G, 139T, 190A, 277T, 290A, 316A, 322T, 370A, 386A, 418A, 436A, 460G, 496A, 556GA] [Costa Rica. Solitary, host: *Stenoma* Janzen99 (Depressariidae). BINBOLD:ABA9255]	***D.josealfredohernandezi* Fernandez-Triana & Boudreault, 2019**
6(3)	T1 comparatively broader, T1 length 1.5 × T1 anterior width, and T1 length 1.5 × T1 posterior width; T2 comparatively less quadrate (T2 posterior width 2.3 × T2 length medially) [Costa Rica. Solitary, host: *Brenthia* Janzen12 (Choreutidae). BINBOLD:AAL2298]	***D.angelagonzalezae* Fernandez-Triana & Boudreault, 2019**
–	T1 comparatively narrower, T1 length 1.9–2.4× (very rarely 1.6×) T1 anterior width, and T1 length 1.9–3.0× (very rarely 1.6–1.7× in small specimens) T1 posterior width; T2 comparatively more quadrate, T2 posterior width 1.3–1.9× (very rarely 2.0–2.1× in small specimens) T2 length medially	**7**
7(6)	Mesofemur mostly yellow, at most with small, dark spots (if mesofemur mostly brown, then specimens significantly smaller, with body length 2.50–3.00 mm)	**8**
–	Mesofemur mostly dark brown (body length ≥ 3.30 mm, usually more)	**9**
8(7)	F2 2.1–2.5× (average: 2.3×) as long as F14; T2 posterior width 1.3–1.9× (average: 1.6×) T2 length medially; pterostigma length/width 3.0–3.5× (average 3.2×) [Costa Rica. Gregarious, host: *Antaeotricharenselariana* (Depressariidae). BINBOLD:ABX6174]	***D.alejandromasisi* Fernandez-Triana & Boudreault, 2019**
–	F2 1.8–2.1× (average: 1.9×) as long as F14; T2 posterior width 1.6–2.1× (average: 1.8×) T2 length medially; pterostigma length/width 2.7–3.0× (average 2.8×) [Costa Rica, Ecuador. Gregarious, host: *Antaeotricha* Janzen107 (Depressariidae). BINBOLD:AAL2325]	***D.rogerblancoi* Fernandez-Triana & Boudreault, 2019**
9(7)	Pterostigma length/width 3.0×; posterior ocellar line 1.6× lateral ocellus diameter; ocular ocellar line 1.2× posterior ocellar line [Costa Rica. Gregarious, host: *Antaeotrichaphaeoneura* (Depressariidae). BINBOLD:ACC1300]	***D.genuarnunezi* Fernandez-Triana & Boudreault, 2019**
–	Pterostigma length/width 3.1–3.3× (average 3.2×); posterior ocellar line 1.8–2.0× lateral ocellus diameter; ocular ocellar line 1.2–1.5× posterior ocellar line [Costa Rica. Gregarious, host: *Antaeotricha* Janzen126 (Depressariidae). BINBOLD:ABY3724]	***D.cedenoae* Fernandez-Triana & Boudreault, 2025**
10(1)	T1 and T2 heavily sculptured with strong longitudinal striae (rarely strong reticulated sculpture) covering entire surface of T2 and most of T1 (at least posterior 0.6–0.9, although centrally there may be a narrow smoother area); T2 often with strong, crenulated sulcus along posterior margin; T2 broadly rectangular or trapezoidal in shape, its width at posterior margin 2.4–2.8× (rarely ~ 3.0×) its length medially ***or*** T2 more or less transverse but with anterior and/or posterior margins centrally arcuate (thus increasing T2 length medially, so that width at posterior margin is < or ~ 3.0× its length medially); T1 usually slightly broadening towards posterior margin or parallel-sided, ***if*** very rarely T1 mostly parallel-sided but slightly narrowing towards posterior margin near apex ***then*** T2 always broadly rectangular or trapezoidal	**11**
–	T1 and T2 much less sculptured, if sculptured rarely with strong longitudinal striae and then only covering margins of posterior half or less on T1 and almost never entire surface of T2 (***if*** very rarely T2 more or less almost entirely sculptured, ***then*** T2 always very transverse, its width at posterior margin > 3.5× its length medially); T2 rarely with crenulated sulcus along posterior margin (***if*** sulcus present, less strongly impressed and much narrower than above); T2 shape variable but usually transverse (its width at posterior margin > 3.0× its length medially, usually 3.5–4.0× or more); ***if*** rarely T2 broadly rectangular or trapezoidal ***then*** T1 comparatively thin and narrowing towards posterior margin	**39**
11(10)	T1 comparatively broader, evenly broadening towards posterior margin or mostly parallel-sided but slightly widening towards posterior margin thus T1 length < 1.5× its width at posterior margin; T2 usually broadly rectangular and large, covering most surface of tergum	**12**
–	T1 comparatively thinner, mostly parallel-sided but posterior 0.1–0.3 slightly narrowing towards posterior margin thus T1 length ≥ 2.0× (rarely 1.7×) its width at posterior margin; T2 usually trapezoidal and rather small, not covering entire surface of tergum (rarely T2 more or less transverse)	**30**
12(11)	Anteromesoscutum entirely to mostly covered by coarse and deep punctures, face, anterior half of mesopleuron, scutellar disc with coarse punctures; F1–F4 (sometimes F1–F6) yellow to yellow brown, clearly much paler colored than dark brown scape and rest of flagellomeres; T1 and T2 usually black and T4+ usually brown, but metasoma dorsally with some paler coloration always including T3 (which varies from dark brown centrally with orange-yellow laterally, to mostly or entirely yellow or orange-yellow), sometimes also T4 and T5 orange-yellow and rarely even T2 reddish brown; legs mostly yellow or orange-yellow, except for metafemur posterior 0.3–0.5, metatibia 0.3 and entire metatarsus brown to dark brown [Canada, Costa Rica, United States. Solitary (?), hosts: *Choristoneurafreemani*, *Epiblemastrenuana* (Tortricidae), *Fissicrambusmutabilis*, *Neodactriazeellus* (Crambidae) BINBOLD:AAA3764]	***D.ensiger* (Say, 1836)**
–	Anteromesoscutum, face, mesopleuron and scutellar disc with variable sculpture but never as coarse and deep as above; flagellomeres of approx. same coloration and usually brown to dark brown or black; metasoma usually entirely dark brown to black, without extensive pale coloration; legs with more extensive dark coloration	**13**
13(12)	Ovipositor sheath spatula-shaped and 0.8× as long as metatibia length; distinctive leg color with coxae dark brown to black and rest of legs mostly dark brown (except for bright yellow trochanters and trochantellus, dorsal margin of profemur, anterior 0.1 of pro- and mesotibiae and anterior 0.3 of metatibia; and all tibial spurs white-yellow); tegula dark brown to black, humeral complex mostly dark brown; pterostigma bright yellow-white but with thin brown margins, most of wing veins pale yellow-brown; F15 sub-cubic (1.1× as long as high); propodeum areola mostly defined, but open on anterior ~ 0.3, and without defined transverse carinae; body length 3.80 mm, fore wing length 4.20 mm [Colombia]	***D.caldas* Fernandez-Triana & Boudreault, sp. nov.**
–	Ovipositor sheath not spatula-shaped; different leg coloration, usually neither as dark nor as contrasting; tegula and humeral complex variable but not as dark as above; pterostigma and wing venation color not as above; F15 usually not cubic, often > 1.3× as long as high; propodeum areola variable but not as above; body and fore wing lengths variable but usually much smaller	**14**
14(13)	Metatibia dark brown to black on posterior 0.5–0.8; body length comparatively smaller, usually 2.20–2.70 mm	**15**
–	Metatibia mostly yellow with only dark brown to black on posterior 0.1–0.2 (in addition there can be a dark brown band dorsally on entire length of metatibia); body length comparatively larger, usually 3.10–3.80 mm	**24**
15(14)	Metatibia dark brown to black on posterior 0.5; ocular ocellar line 1.4× posterior ocellar line	**16**
–	Metatibia dark brown to black on posterior 0.6–0.8; ocular ocellar line 1.0–1.1× posterior ocellar line	**17**
16(15)	F15 length/width 1.5×; size comparatively smaller, body and fore wing lengths 2.30–2.40 mm; pterostigma brown [Costa Rica, 1,000 m]	***D.monocavus* (Valerio & Whitfield, 2004)**
–	F15 length/width 1.1×; size comparatively larger, body length 2.70 mm, fore wing length 3.00 mm; pterostigma brown with small pale spot at base [Venezuela, 1,900 m]	***D.verobrondexae* Fernandez-Triana & Boudreault, sp. nov.**
17(15)	T3 sculptured on anterior 0.2–0.5 (but there mostly centrally); T1 and T2 mostly with reticulate sculpture; tarsal claws of hind legs simple [Costa Rica. Solitary, hosts: *Brenthia* Janzen05, *Stenoma* Phillips543 (Choreutidae, Gelechiidae, Depressariidae). BINBOLD:ABY5258]	***D.rosamatarritae* (Fernandez-Triana, 2016)**
–	T3 entirely smooth; T1 and T2 mostly with longitudinally striated sculpture; tarsal claws of hind legs with single spine-like basally	**18**
18(17)	Metafemur yellow on anterior half and brown on posterior half; **and** ovipositor sinuate; **and** all laterotergites, sternites and hypopygium yellow; **and** T3–T6 centrally brown, laterally with yellow spots [Ecuador]	***D.palenque* Fernandez-Triana & Boudreault, sp. nov.**
–	Metafemur color different, either mostly brown or entirely to almost entirely yellow; and ovipositor not sinuate; metasoma color variable but not as above	**19**
19(18)	Metafemur mostly brown (only anterior 0.1 and posterior 0.1 yellow)	**20**
–	Metafemur entirely yellow, at most with brown spot dorsally on posterior 0.2 or less	**22**
20(19)	F15 2.0× as long as wide; DNA barcodes 5.9% different from closest BIN and > 7.0% different from species in second half of couplet [Costa Rica. BINBOLD:AAM5843]	***D.rodrigogamezi* Fernandez-Triana & Boudreault, sp. nov.**
–	F15 1.5× as long as wide; DNA barcodes > 7.0% different from species in first half of couplet [BINBOLD:ACJ2777, BINBOLD:ABA3469]	**21**
21(20)	Ovipositor sheath almost as long as metatibia length (0.95×); F15 comparatively shorter, 1.2× as long as wide [Costa Rica. Solitary, hosts: *Dichomeris* spp. (Gelechiidae). BINBOLD:ACJ2777]	***D.rociocordobae* Fernandez-Triana & Boudreault, sp. nov.**
–	Ovipositor sheath clearly shorter than metatibia length (0.80×); F15 comparatively longer, 1.4× as long as wide [Costa Rica. Solitary, hosts: *Platynota* spp. (Tortricidae). BINBOLD:ABA3469]	***D.frankjoycei* Fernandez-Triana & Boudreault, sp. nov.**
22(19)	Tegula and humeral complex of same color, white-yellow [Costa Rica. Solitary, host: undetermined species of Gelechiidae with interim name ‘gelJanzen01 Janzen319’. BINBOLD:AAN2496]	***D.mariabustosae* (Fernandez-Triana, 2016)**
–	Tegula white-yellow, clearly paler than brown humeral complex	**23**
23(22)	Pro- and mesocoxae yellow; laterotergites 1–4 and at least sternites 1 and 2 entirely to mostly yellow; pterostigma with very small pale spot on anterior 0.1 or less; F15 length 1.4–1.5× its height [Costa Rica, 600–700 m. Solitary, host: *Gonionota* Janzen116 (Depressariidae). BINBOLD:ACC4119]	***D.felipechavarriai* Fernandez-Triana & Boudreault, sp. nov.**
–	Pro- and mesocoxae dark reddish brown; all laterotergites and sternites pale brown to dark brown; pterostigma with pale spot on anterior 0.25; F15 length 1.2× its height [Guatemala, 2,000 m]	***D.chichicastenango* Fernandez-Triana & Boudreault, sp. nov.**
24(14)	Pterostigma with pale yellow spot on proximal 0.5; smaller species (body length 3.10 mm and fore wing length 3.40 mm) [Costa Rica. Solitary, host: undetermined species of Gelechiidae with interim name ‘gelJanzen01 Janzen349’. BINBOLD:ABX6267]	***D.richardashleyi* (Fernandez-Triana, 2016)**
–	Pterostigma mostly brown, at most with small pale spot on proximal 0.1–0.2; usually, but not always, larger species (body length 3.30–3.80 mm and fore wing length 3.50–4.10 mm)	**25**
25(24)	Metatibial spurs with brown tips; F2 length 2.1 × F14 length [Costa Rica. BINBOLD:ABX6267]	***D.yeimycedenoae* (Fernandez-Triana, 2016)**
–	Metatibial spurs entirely yellow; F2 length 2.5 × F14 length	**26**
26(25)	T2 more or less transverse, with anterior margin centrally arcuate, so that its width at posterior margin is ~ 3.0× its length medially; **and** metacoxa almost entirely dark brown (very small yellow spot on posterior 0.1); **and** body and fore wing lengths 3.50–3.70 mm [Colombia]	***D.antioquia* Fernandez-Triana & Boudreault, sp. nov.**
–	T2 broader, its width at posterior margin ≤ 2.8× its length medially (usually less); ***either*** metacoxa dark brown on anterior half and yellow on posterior half, ***or*** metacoxa almost entirely dark brown ***and*** body and fore wing lengths ≤ 3.30 mm	**27**
27(26)	Hind legs tarsal claws simple; metacoxa dark brown on anterior half and yellow on posterior half; comparatively larger species, body length 3.50–3.80 mm	**28**
–	Hind legs tarsal claws with single spine; metacoxa almost entirely dark brown (very small yellow spot on posterior 0.1); comparatively smaller species, body length ≤ 3.30 mm	**29**
28(27)	Metatibia with dorsal dark brown band on entire length of metatibia; pterostigma mostly pale yellow-brown but with thin brown margins; T1 comparatively narrower, ~ 2.0× as long as wide at posterior margin [Costa Rica]	***D.heredia* Fernandez-Triana & Boudreault, sp. nov.**
–	Metatibia without dorsal dark band; pterostigma mostly brown, with pale spot at base; T1 comparatively broader, < 1.6× as long as wide at posterior margin [Costa Rica. BINBOLD:ABX6267]	***D.ritaashleyae* (Fernandez-Triana, 2016)**
29(27)	Metatibia with dark brown spot on posterior 0.2; metatarsus mostly brown; T2 more transverse, its width at posterior margin > 3.0× its central length; comparatively smaller species, body length 2.70–2.90 mm; ovipositor sheath 1.0–1.1× metatibia length [Costa Rica. Solitary, hosts: Depressariidae: elachJanzen01 Janzen409; Gelechiidae: *Dichomeris* Janzen76, gelJanzen01 Janzen116. BINBOLD:AAC5949]	***D.tiboshartae* Fernandez-Triana & Boudreault, sp. nov.**
–	Metatibia almost entirely yellow, with pale brown spot on posterior 0.1; metatarsus mostly yellow to yellow-pale brown; T2 subrectangular its width at posterior margin < 2.7× its central length; comparatively larger species, body length 3.10–3.30 mm; ovipositor sheath 1.2–1.3× metatibia length [Costa Rica. Solitary, hosts: *Megalota* spp. (Tortricidae). BINBOLD:AAY4690]	***D.ninamasisae* Fernandez-Triana & Boudreault, sp. nov.**
30(11)	Ovipositor sheath 1.6× metatibia length; **and** T1 length 1.8× its width at posterior margin; **and** legs almost entirely brown to dark brown (except for yellow protibial and protarsus and very small, paler spots on posterior 0.1 of pro- and mesofemora and anterior 0.1–0.2 of meso- and metatibiae); **and** tegula and humeral complex dark brown; **and** pterostigma mostly yellow-white but with thin brown margins [Guatemala]	***D.alexandrei* Fernandez-Triana & Boudreault, sp. nov.**
–	Ovipositor sheath much shorter or much longer than above; **and/or** T1 length almost always > 2.0× its width at posterior margin; **and/or** legs with at least some segments pale colored (yellow to yellow-white or yellow-orange); **and/or** tegula and/or humeral complex usually yellow; **and/or** pterostigma color variable but usually not as above	**31**
31(30)	Ovipositor sheath ~ 2.0× metatibia length; body length 3.20–3.50 mm	**32**
–	Ovipositor sheath ≤ 1.2× metatibia length (usually much less); usually body length 2.30–2.40 mm (rarely 2.70–3.20 mm)	**33**
32(31)	Hypopygium and most sternites dark brown; metacoxa partially yellow partially dark brown; comparatively larger ovipositor sheath (1.70–2.00 mm) in spite of slightly smaller body and fore wing (2.60–3.10 mm) than below, with ratio of ovipositor sheath length/body length of 0.60–0.65 [Costa Rica. Solitary, hosts: *Antaeotricha* spp. *Chlamydastis* Janzen04, *Stenoma* spp. (Depressariidae and Crambidae). BINBOLD:AAD8952]	***D.tomdaleyi* Fernandez-Triana & Boudreault, sp. nov.**
–	Hypopygium and all sternites yellow; metacoxa entirely brown; comparatively shorter ovipositor sheath (1.50 mm) despite slightly larger sized body and fore wing (3.20 mm) than above, with ratio of ovipositor sheath length/body length of 0.47 [Costa Rica. Solitary, host: undetermined species of Depressariidae with interim name elachBioLep01 BioLep286]	***D.sallydaleyae* Fernandez-Triana & Boudreault, sp. nov.**
33(31)	All legs almost entirely brown to dark brown (except for very small, paler spots, on posterior 0.1 of pro- and mesofemora and anterior 0.1–0.2 of tibiae); wings slightly infumated; tegula and humeral complex brown; F15 cubic (around same length than width); T2 shape trapezoidal but rather narrow, barely wider than T1; pterostigma mostly yellow-white but with thin brown margins [Bolivia]	***D.yungas* Fernandez-Triana & Boudreault, sp. nov.**
–	Legs with at least some segments pale colored (yellow to yellow-white or yellow-orange); wings not infumated; tegula and/or humeral complex usually yellow; F15 not cubic (longer than wide); ***if*** T2 trapezoidal not as narrow as above; pterostigma color variable but usually not as above	**34**
34(33)	Tegula brown; ovipositor strongly sinuate; propodeum areola narrower (its height > 1.5× its central width) and open anteriorly [Costa Rica. BINBOLD:AAM5848]	***D.carlosalvaradoi* Fernandez-Triana & Boudreault, sp. nov.**
–	Tegula yellow; ovipositor not sinuate; propodeum areola broader (its height ~ 1.2× its central width) and close anteriorly	**35**
35(34)	Scutellar disc with coarse punctures; anterior half of mesopleuron and anteromesoscutum with relatively coarse punctures; pterostigma pale brown but centrally paler than margins; tegula and humeral complex yellow; metatibia entirely brown to dark brown [Costa Rica. BINBOLD:AAD2236]	***D.claudiadoblesae* Fernandez-Triana & Boudreault, sp. nov.**
–	Scutellar disc smooth; anterior half of mesopleuron smooth, anteromesoscutum with sparse and relatively shallow punctures; pterostigma mostly pale brown with small, paler spot anteriorly; tegula white-yellow, humeral complex mostly brown; metatibia yellow at least on anterior 0.4	**36**
36(35)	T3 with yellow spots laterally, centrally pale brown, T4+ pale brown to brown; laterotergites 1–5 yellow, most sternites at least partially yellow, hypopygium partially yellow and partially pale brown; pro- and mesocoxae entirely yellow, metacoxa reddish brown; propodeum with carinae defining areola strongly risen and sharp; F15 1.3× as long as high [Ecuador]	***D.isabelleae* Fernandez-Triana & Boudreault, sp. nov.**
–	Metasoma dorsally entirely dark brown to black; all laterotergites, sternites and hypopygium entirely brown to dark brown; pro- and mesocoxae partially reddish brown to brown, metacoxa black or dark brown; propodeum with carinae defining areola not as strongly risen as above; F15 ≥ 1.5× as long as high [Costa Rica]	**37**
37(36)	Ovipositor comparatively very thin, much thinner than half flagellomeres width; T2 comparatively less transverse, its width at posterior margin 2.8× its central length; metatibia yellow on anterior 0.4–0.5 [Costa Rica. BINBOLD:AAM5846]	***D.jorgecortesi* Fernandez-Triana & Boudreault, sp. nov.**
–	Ovipositor comparatively thicker, at least as thick as 0.8× flagellomeres width; T2 comparatively more transverse, its width at posterior margin > 3.1× its central length; metatibia mostly yellow, only posterior 0.2 or less dark brown	**38**
38(37)	Comparatively smaller species, body length 2.30 mm; parasitizing Erebidae; wasp cocoons solitary; DNA barcodes 3.7% different from closest BIN and 5.3% different from species in second half of couplet [Costa Rica. Solitary, host: *Rivula* Poole03BIN (Erebidae). BINBOLD:ABA7252]	***D.andreamezae* Fernandez-Triana & Boudreault, sp. nov.**
–	Comparatively larger species, body length 2.70–3.00 mm; parasitizing Mimallonidae; wasp cocoons gregarious; DNA barcodes 2.1% different from closest BIN and 5.3% different from species in first half of couplet [Costa Rica. Gregarious, host: *Eadmuna* Janzen01 (Mimallonidae). BINBOLD:AAB4946]	***D.pedroleoni* Fernandez-Triana & Boudreault, sp. nov.**
39(10)	Pro- and mesocoxae pale (white, yellow, orange, yellow-pale brown); metacoxa pale entirely or mostly pale (anterior 0.3–0.4 dark), very rarely metacoxa mostly dark brown	**40**
–	Pro- and mesocoxae mostly to entirely dark (dark orange-brown, brown, dark brown, black); **and** metacoxa entirely (often) or mostly (rarely) dark, at most with small pale area on 0.2 or less of metacoxa	**65**
40(39)	Comparatively paler colored species; T3 (sometimes also T4 and part of T5) entirely yellow to yellow-white, rest of tergites pale brown (except for dark brown T1); **and** hypopygium and all or most laterotergites and sternites yellow to yellow-white; **and** all coxae yellow-white, rest of legs mostly yellow to yellow-white (except for part of metafemur and metatibia); **and** antenna pale brown; **and** tegula and humeral complex yellow; **and** mesosoma with some spots pale brown to orange-brown; **and** fore wing veins r and 2RS strongly angulate; **and** flagellomeres with comparatively long setae, as long as half flagellomere width	**41**
–	Comparatively darker colored species, usually all or most tergites brown to black, **if** rarely with T1 or T2 yellow or pale brown-yellow **then** T3 always brown, **or** T3 laterally yellow but centrally brown; laterotergites, sternites and hypopygium variable but very rarely all pale; **and/or** legs usually with some segments dark; antenna color usually dark brown to black, rarely pale brown; tegula and humeral complex color variable; fore wing veins r and 2RS variable but rarely strongly angulate; flagellomeres usually with shorter setae	**43**
41(40)	T1 more or less parallel-sided, only very slightly narrowing near posterior margin; T4 (mostly) and T5 (partially) yellow to yellow-white; metafemur mostly yellow-white with only posterior 0.1 brown [Ecuador]	***D.limoncocha* Fernandez-Triana & Boudreault, sp. nov.**
–	T1 strongly narrowing towards posterior margin so that width at anterior margin is clearly longer than width at posterior margin; most tergites brown to reddish brown (except for yellow T3); metafemur either entirely yellow or yellow on anterior 0.5 and dark brown on posterior 0.5	**42**
42(41)	Flagellomeres orange-yellow; metafemur entirely yellow; T1 very strongly narrowing posteriorly, its width at anterior margin ~ 2.0× its width at posterior margin [Peru. Solitary, host: *Microthyrisanormalis* (Crambidae)]	***D.croceicornis* (Muesebeck, 1958)**
–	Flagellomeres brown; metafemur yellow on anterior 0.5 and dark brown on posterior 0.5; T1 not as strongly narrowing posteriorly, its width at anterior margin ~ 1.5× its width at posterior margin [Costa Rica, Ecuador, Venezuela. Solitary, hosts: *Herpetogramma* Janzen04, *Rhectocraspeda* Solis05 (Crambidae). BINBOLD:AAF5364]	***D.alexamasisae* Fernandez-Triana & Boudreault, sp. nov.**
43(40)	T3 mostly yellow (only pale brown on central part); **and** first and second pair of legs almost entirely yellow (except for pale brown mesocoxa), third pair of legs mostly brown to dark brown (except for yellow trochanter, trochantellus, anterior 0.1 and posterior 0.1 of metafemur, and anterior 0.5–0.6 of metatibia); T1 mostly strongly sculptured, T2 entirely smooth; T2 comparatively narrow and sub-quadrate, its width at posterior margin 2.0× its central length; fore wing veins r and 2RS strongly angulate; pterostigma mostly yellow-white with thin brown margins [Peru, 2,000 m]	***D.amazonas* Fernandez-Triana & Boudreault, sp. nov.**
–	T3 entirely pale brown, dark brown or black; leg coloration different than above; **and/or** T1 and T2 sculptured different than above; **and/or** T2 more transverse; **and/or** fore wing veins r and 2RS variable but rarely strongly angulate; **and/or** pterostigma coloration different than above	**44**
44(43)	Posterior 0.1–0.2 of T1 (centrally) and entire T2 yellow to pale brown-yellow; T3+ pale brown; metacoxa entirely to almost entirely dark brown; anteromesoscutum with coarse, deep and dense punctures (separation between punctures less than individual puncture diameter); face, propleuron, pronotum, most of mesopleuron, scutellar disc, and most of outer side of metacoxa mostly to entirely covered by relatively coarse punctures [Costa Rica. BINBOLD:AAF7717]	***D.ericpalolai* Fernandez-Triana & Boudreault, sp. nov.**
–	Different coloration pattern of T1 and T2 (usually mostly to entirely brown, dark brown or black); T3+ almost always dark brown to black; metacoxa usually entirely to partially pale colored (white, yellow-white, yellow, orange-yellow); anteromesoscutum rarely as strongly sculptured; face, propleuron, pronotum, most of mesopleuron, scutellar disc and most of outer side of metacoxa with variable sculpture, but usually mostly smooth or with shallow and/or sparse punctures	**45**
45(44)	Metatibia entirely to mostly pale (at most with darker area on posterior 0.3 or less), **if** metatibia with brown margin dorsally **then** rest of legs yellow or white-yellow	**46**
–	Metatibia entirely to mostly dark (at most with paler area on anterior 0.3 or less) ***and*** at least some other leg segments dark	**58**
46(45)	Extensive orange coloration, including clypeus, anteromesoscutum (almost entirely, except for small dark spot near scutellar disc), antero-dorsal spot on mesopleuron, propleuron (partially), mesosternum (mostly) and two faint spots postero-laterally on T1 [Costa Rica. BINBOLD:AAM5736]	***D.shelleymcsweeneyae* Fernandez-Triana & Boudreault, sp. nov.**
–	If there is any orange or orange-yellow coloration, it is very limited to small, isolated spots	**47**
47(46)	Metafemur and metatibia mostly yellow-white but with brown to dark brown margins dorsally (dark dorsal margin only partially defined on metafermur); pterostigma mostly white-yellow on anterior 0.5–0.7, with darker (pale brown) margins [Costa Rica. Solitary, host: undetermined Crambidae. BINBOLD:ABY1812]	***D.anikenpalolae* Fernandez-Triana & Boudreault, sp. nov.**
–	Metafemur and metatibia mostly yellow or yellow-white (at most with brown spot on posterior 0.3 or less), without dark margins dorsally; pterostigma uniformly colored, mostly brown to brown, or brown with small pale spot on anterior 0.2	**48**
48(47)	T1 strongly narrowing towards posterior margin, its length medially ~ 4.0–5.0× its width at posterior margin and its width at anterior margin 2.0× its width at posterior margin	**49**
–	T1 either parallel-sided or mostly parallel-sided and only narrowing towards posterior margin near its posterior half, its length medially ≤ 3.0× its width at posterior margin (usually less) and its width at anterior margin < 1.5× its width at posterior margin (usually less)	**51**
49(48)	Humeral complex, all laterotergites, sternites and hypopygium brown to dark brown [Costa Rica. BINBOLD:AAI9740]	***D.tristanpalolai* Fernandez-Triana & Boudreault, sp. nov.**
–	Humeral complex, at least some laterotergites, all sternites and hypopygium yellow	**50**
50(49)	Ovipositor sheath almost as long as metatibia length (0.9×); tegula yellow, same color than humeral complex [Costa Rica. BINBOLD:AAD6850]	***D.junhyongkimi* Fernandez-Triana & Boudreault, sp. nov.**
–	Ovipositor sheath clearly shorter than metatibia length (0.7×); tegula brown, darker than yellow humeral complex [Costa Rica. BINBOLD:AAM5850]	***D.ingredolsonae* Fernandez-Triana & Boudreault, sp. nov.**
51(48)	Ovipositor comparatively thicker, as wide or wider than flagellomeres width; **and** F15 comparatively very long, 2.0× as long as its width; **and** fore wing vein r longer than pterostigma height and ~ 2.0× as long as vein 2RS; **and** all legs yellow or orange yellow, except for metacoxa mostly dark brown to black but with posterior 0.2 yellow; **and** pterostigma mostly brown but with pale spot at anterior 0.2 [French Guiana]	***D.yvesbraeti* Fernandez-Triana & Boudreault, sp. nov.**
–	Ovipositor comparatively thinner, its width much less than flagellomeres width; **and/or** F15 comparatively shorter, < 1.5× as long as its width; **and/or** fore wing vein r not approx. same length or shorter than pterostigma height and < 1.5× as long as vein 2RS; **and/or** legs with different coloration pattern; **and** pterostigma mostly brown to pale brown	**52**
52(51)	T2 comparatively less transverse, its length 3.0× its width at posterior margin	**53**
–	T2 comparatively more transverse, its length 4.0–5.0× its width at posterior margin	**54**
53(52)	Ovipositor sheath 1.0× as long as metatibia; fore wing veins r and 2RS not meeting at strong angle; comparatively smaller species, body length 2.00 mm, fore wing length 2.20 mm [Costa Rica, Honduras. BINBOLD:ACE8228]	***D.katiemccluskeyae* Fernandez-Triana & Boudreault, sp. nov.**
–	Ovipositor sheath 0.75× as long as metatibia; fore wing veins r and 2RS meeting at a strong angle; comparatively larger species, body length 2.60 mm, fore wing length 2.70 mm [Costa Rica]	***D.arenal* Fernandez-Triana & Boudreault, sp. nov.**
54(52)	Tegula and humeral complex dark brown [Costa Rica. BINBOLD:AAJ1396]	***D.leahdennisae* Fernandez-Triana & Boudreault, sp. nov.**
–	Tegula yellow or pale brown, humeral complex yellow	**55**
55(54)	Scutellar disc mostly with punctures; anteromesoscutum with relatively coarse punctures; T1 length medially ~ 3.0× its width at posterior margin; anterior 0.3 of mesopleuron and posterior 0.4–0.5 of metapleuron sculptured [Costa Rica. BINBOLD:AAC7481]	***D.joshdarfleri* Fernandez-Triana & Boudreault, sp. nov.**
–	Scutellar disc usually smooth and shiny, without punctures (at most with very shallow punctures along margins); anteromesoscutum mostly smooth or with relatively shallow punctures; T1 length medially ≤ 2.5× its width at posterior margin (usually less); mesopleuron and metapleuron entirely to almost entirely smooth or with few, shallow punctures	**56**
56(55)	T2 mostly smooth; T1 often (but not always) with anterior 0.4–0.5 yellow-brown, clearly paler colored than rest of tergite (which is dark brown or reddish brown) [Costa Rica. Solitary, host: *Antaeotricha* Janzen221 (Depressariidae). BINBOLD:ABX5195]	***D.robinsherwoodae* Fernandez-Triana & Boudreault, sp. nov.**
–	T2 entirely sculptured (rarely weakly sculptured but then sculpture covering most of tergite); T1 entirely dark brown or black	**57**
57(56)	Scape yellow, much paler than brown flagellomeres; ovipositor sheath 0.7× as long as metatibia; pterostigma entirely pale brown [Canada, Honduras, United States. Solitary, hosts: *Keiferia* spp. (Gelechiidae)]	***D.phthorimaeae* (Muesebeck, 1921)**
–	Scape brown, same color than flagellomeres; ovipositor sheath 0.9× as long as metatibia; pterostigma mostly bright yellow-white (but with central, darker spot which is pale brown) [Argentina]	***D.tucuman* Fernandez-Triana & Boudreault, sp. nov.**
58(45)	T1 comparatively narrow, its length medially ~ 3.0× its width at posterior margin; **and** tegula brown, clearly darker than yellow humeral complex; **and** all trochanters, pro- and mesocoxae white; **and** metacoxa mostly white-yellow, with dark brown spot on anterior 0.2–0.3 [Costa Rica. BINBOLD:ACF2929]	***D.paulfryi* Fernandez-Triana & Boudreault, sp. nov.**
–	T1 usually comparatively broader, its length medially usually 2.0–2.5× its width at posterior margin (rarely up to 3.0×); **and/or** tegula white or yellow, similar color or paler than humeral complex; **and/or** all trochanters, pro- and mesocoxae yellow; **and** metacoxa entirely to mostly dark brown (very rarely mostly yellow with dark brown spot on anterior 0.3)	**59**
59(58)	Metafemur entirely to mostly yellow (at most thin brown area dorsally on posterior 0.3 or less); T2 with large, smooth area centrally, sculpture limited to margins	**60**
–	Metafemur mostly dark brown (except for yellow-white spot on anterior 0.2–0.3 or posterior 0.2); T2 mostly sculptured, at most with small central area smooth	**61**
60(59)	T1 comparatively narrower, its length medially ~ 3.0× its width at posterior margin [Costa Rica. BINs BOLD:AAB5549, BOLD:ACF0272]	***D.annlisterudae* Fernandez-Triana & Boudreault, sp. nov.**
–	T1 comparatively broader, its length medially ~ 2.2× its width at posterior margin [Costa Rica. BINBOLD:ACF0267]	***D.johnrobinsoni* Fernandez-Triana & Boudreault, sp. nov.**
61(59)	Pterostigma entirely yellow-white or mostly yellow-white but with thin brown margins; metacoxa entirely dark brown	**62**
–	Pterostigma brown; metacoxa half brown and half pale (yellow or yellow-white)	**63**
62(61)	Much larger body size (body length 4.00 mm, fore wing length 4.50 mm); all sternites yellow, hypopygium yellow on anterior 0.5, dark brown on posterior 0.5; pterostigma mostly yellow-white but with thin brown margins; T1 slightly broadening towards posterior margin, its length 1.2× its width at posterior margin; T1 almost entirely covered by coriaceous sculpture; T2 comparatively less transverse, its width at posterior margin 3.0× its central length; T2 mostly covered by longitudinal sculpture, but with two smoother, small areas near anterior margin centrally [Ecuador]	***D.papallacta* Fernandez-Triana & Boudreault, sp. nov.**
–	Much smaller body size (body length and fore wing length 2.20–2.50 mm); all sternites and hypopygium brown; pterostigma entirely yellow-white; T1 distinctively narrowing from posterior 0.3 towards posterior margin, its length 2.0× its width at posterior margin; T2 comparatively more transverse, its width at posterior margin > 3.5× its central length, its length 2.0× its width at posterior margin; T1 posterior 0.6 and T2 (mostly) sculptured [Costa Rica. BINBOLD:AAM5842]	***D.teremariae* Fernandez-Triana & Boudreault, sp. nov.**
63(61)	T2 with central area smooth; tegula yellow, same color than humeral complex; most of metafemur and metatibia brown [Costa Rica. BINBOLD:AAM5088]	***D.jennyphillipsae* Fernandez-Triana & Boudreault, sp. nov.**
–	T2 mostly sculptured (if there is a central area smooth, it is very small); tegula white-yellow, clearly paler in color than yellow humeral complex; most of metafemur and metatibia black	**64**
64(63)	DNA barcodes with 14 diagnostic characters: 100G, 115C, 127A, 197T, 202T, 313A, 316G, 322A, 340C, 343C, 388G, 397C, 457C, 484C [Costa Rica. BINBOLD:AAC8392]	***D.robertofernandezi* Fernandez-Triana & Boudreault, sp. nov.**
–	DNA barcodes with 14 diagnostic characters: 100A, 115T, 127T, 197C, 202C, 313G, 316A, 322G, 340T, 343T, 388A, 397T, 457T, 484T [Costa Rica. BINBOLD:AAB9372]	***D.isidrochaconi* Fernandez-Triana & Boudreault, sp. nov.**
65(39)	Metafemur brown on anterior half and yellow on posterior half; **and** ovipositor sheath 0.6× as long as metatibia length; **and** propodeum mostly smooth with only two carinae partially defining an areola on posterior 0.4; **and** T1 mostly sculptured on posterior 0.6 **and** T2 mostly smooth [Chile]	***D.ericsimoni* Fernandez-Triana & Boudreault, sp. nov.**
–	Metafemur mostly pale ***or*** mostly dark; ovipositor sheath variable but often longer than 0.6× metatibia length; propodeum variable but often with some sculpture in addition to propodeal carinae; sculpture of T1 and T2 variable but often not as above	**66**
66(65)	Metafemur entirely to mostly pale (at most with darker spot on posterior 0.3 or less, or narrow dark borders around margins but still mostly pale)	**67**
–	Metafemur entirely to mostly dark (at most with pale spots on anterior 0.2 or less, or posterior 0.2 or less, or with very small area centrally paler colored)	**78**
67(66)	Ovipositor sheath < 0.5× metatibia length	**68**
–	Ovipositor sheath > 0.7× metatibia length (usually much more)	**69**
68(67)	DNA barcodes with 64 diagnostic characters: 56T, 67T, 73A, 88A, 127A, 133T, 139A, 172A, 181G, 184A, 190T, 205A, 211G, 217A, 220A, 223T, 229A, 232A, 241G, 244G, 256A, 274T, 278T, 281T, 283T, 293T, 298T, 311A, 312C, 319G, 322G, 325A, 343A, 352T, 356A, 357T, 358T, 359T, 368G, 376A, 386T, 400T, 412T, 421A, 433T, 434G, 436T, 439T, 445T, 448T, 449A, 454A, 455A, 482A, 484A, 494A, 526T, 528C, 529A, 544G, 547T, 553T, 568T, 574T [Costa Rica. BINBOLD:AAE8596]	***D.bernardoespinozai* Fernandez-Triana & Boudreault, sp. nov.**
–	DNA barcodes with 64 diagnostic characters: 56A, 67A, 73G, 88T, 127T, 133C, 139T, 172T, 181A, 184G, 190A, 205T, 211A, 217T, 220T, 223A, 229G, 232T, 241A, 244A, 256T, 274A, 278G, 281G, 283A, 293A, 298A, 311G, 312T, 319A, 322T, 325T, 343T, 352A, 356T, 357C, 358G, 359A, 368A, 376G, 386A, 400A, 412A, 421T, 433G, 434T, 436A, 439A, 445C, 448A, 449T, 454T, 455G, 482T, 484G, 494T, 526A, 528T, 529T, 544A, 547A, 553A, 568A, 574A [Costa Rica. Solitary, host: Immidae. BINBOLD:ACI3413]	***D.angelsolisi* Fernandez-Triana & Boudreault, sp. nov.**
69(67)	Posterior 0.5 of T1 and entire T2 sculptured with strong, longitudinal striae; **and** T1 comparatively thinner, its length 2.0× its width at posterior margin; **and** T2 width at posterior margin 3.7× its length medially; **and** body color mostly pale reddish brown; **and** pterostigma with small pale (pale yellow) with white spots on anterior 0.1 and posterior 0.1; **and** propodeum mostly smooth, with comparatively tall and thin areola that occupies the entire length of propodeum and it is completely defined by carinae; **and** ovipositor sheath 1.5× metatibia length [Colombia]	***D.putumayo* Fernandez-Triana & Boudreault, sp. nov.**
–	Different combination of characters, **either** with posterior 0.5–0.6 of T1 and/or T2 mostly smooth **or** T2 sculptured but with small polished area centrally; **and/or** T2 width at posterior margin < 3.0× its length medially; **and** body color mostly dark brown to black; **and** pterostigma with different color pattern; and/or propodeum with areola variously defined and not as thin; **and/or** ovipositor sheath length either shorter or longer than 1.5× metatibia length	**70**
70(69)	T1 strongly narrowing towards posterior margin (width at anterior margin 1.8× width at posterior margin), T1 length 4.5× its width at posterior margin; **and** T2 weakly sculptured and trapezoidal, its width at posterior margin 2.5× its central length [Brazil]	***D.escobarae* Fernandez-Triana & Boudreault, sp. nov.**
–	T1 broader, its width at anterior margin similar or only very slightly broader (1.2× or less) than width at posterior margin, T1 length < 3.0× its width at posterior margin; T2 sculpture and shape variable but not as above	**71**
71(70)	Posterior 0.5–0.6 of T1 and/or T2 mostly smooth, if sculptured, without strong longitudinal striae mostly covering terga surface; **and/or** ovipositor sheath < 1.2× as metatibia length; **and/or** T2 transverse and comparatively narrow, its width at posterior margin > 3.0× its length medially (usually more)	**72**
–	Posterior 0.5–0.6 of T1 and T2 mostly with strong sculpture, usually longitudinal striae mostly covering terga surface (but T2 with small polished area centrally); **and** ovipositor comparatively very long, ≥ 1.5× as metatibia length (usually much more, rarely 1.3–1.4×); T2 more or less transverse but with anterior and/or posterior margins strongly arcuate, so that T2 length is longer medially than laterally and thus T2 width at posterior margin is usually < 3.0× its length medially	**75**
72(71)	Propodeum without areola, with only small carinae near nucha; metatarsus yellow-brown, similar color than metatibia [Saint Vincent]	***D.parallelis* (Ashmead, 1900)**
–	Propodeum with areola, areola delimited by carina posteriorly and laterally; metatarsus dark brown to black, clearly much darker than yellow-brown metatibia [Costa Rica]	**73**
73(72)	T1 shiny and almost entirely smooth (at most with few shallow punctures along lateral margins on posterior 0.3); T1 broader, its length medially 2.0× its width at posterior margin [Costa Rica. Solitary, hosts: *Chlamydastis* spp., *Stenoma* Janzen199 (Depressariidae). BINBOLD:AAT8860]	***D.ronaldzunigai* Fernandez-Triana & Boudreault, sp. nov.**
–	T1 with some sculpture on posterior half; T1 narrower, length medially ≥ 2.5× its width at posterior margin	**74**
74(73)	Scape entirely dark brown to black; pro- and mesocoxae brown, metacoxa black; tegula yellow, much paler than brown humeral complex; anteromesoscutum punctures near end of notauli well separated and similar to puntures on rest of anteromesoscutum [Costa Rica. BINBOLD:AAM5740]	***D.juanmatai* Fernandez-Triana & Boudreault, sp. nov.**
–	Scape ventrally yellow-brown, distinctly paler colored than dorsal side; pro- and mesocoxae partially pale brown partially yellow, metacoxa mostly dark brown to black but with posterior 0.1–0.2 yellow; tegula brown, same color than humeral complex; anteromesoscutum punctures near end of notauli fused, unlike punctures on rest of anteromesoscutum [Costa Rica. Gregarious, hosts: *Herpetogramma* spp. (Crambidae)]	***D.jaimelewisi* Fernandez-Triana & Boudreault, sp. nov.**
75(71)	Vannal lobe straight and with very few, very small setae [United States. Solitary, hosts: *Acrobasiscaryae*, *Gretchenabolliana* (Pyralidae, Tortricidae)]	***D.acrobasidis* (Muesebeck), 1921**
–	Vannal lobe slightly convex and uniformly bordered by setae	**76**
76(75)	Ovipositor very long, 2.0× as long as metatibia length; pterostigma with relatively large pale (yellow-white) spot at base that occupies 0.3–0.4 pterostigma length [Costa Rica. Solitary, host: *Ategumialotanalis* (Crambidae). BINBOLD:ABY7999]	***D.anniapicadoae* Fernandez-Triana & Boudreault, sp. nov.**
–	Ovipositor much shorter, ≤ 1.4× as long as metatibia length; pterostigma usually without pale spot at base or with small pale spot occupying < 0.1 pterostigma length	**77**
77(76)	Tegula and humeral complex yellow; most laterotergites, some sternites and sometimes hypopygium yellow to yellow-brown [Costa Rica. BINBOLD:AAM5847]	***D.jorgecarvajali* Fernandez-Triana & Boudreault, sp. nov.**
–	Tegula dark brown, humeral complex partially dark brown partially yellow; laterotergites, sternites and hypopygium brown to dark brown [Costa Rica. BINBOLD:AAL2287]	***D.rexhamiltoni* Fernandez-Triana & Boudreault, sp. nov.**
78(66)	T1 comparatively broader, covering most of dorsal surface of tergum, T1 median length < 1.2× (rarely 1.3×) its width at posterior margin	**79**
–	T1 comparatively narrower, almost always covering only part of dorsal surface of tergum, T1 median length > 1.5× its width at posterior margin (usually much more)	**88**
79(78)	Face rostriformis (malar space longer than mandible width) and with slightly elongate mouth parts; T1 and T2 entirely smooth and shiny; T1 very broad at posterior margin, almost quadrate, its length 1.05× its width at posterior margin; ovipositor sheath 1.5× metatibia length; fore wing veins mostly transparent; **and** pterostigma mostly yellow but with brown margins; **and** palpi, tegula and humeral complex dark brown [Chile]	***D.aceituno* Fernandez-Triana & Boudreault, sp. nov.**
–	Face almost always not rostriformis (malar space approx. same length than mandible width) and almost always without elongate mouth parts; T1 and T2 from entire to partially sculptured; T1 width variable but usually its length > 1.1× its width at posterior margin; ovipositor sheath length variable but usually < 1.5× metatibia length; fore wing veins color variable; pterostigma usually brown or mostly brown with pale spot at base (rarely strongly yellow-white with very thin brown margins); palpi, tegula and humeral complex variously colored but never the three dark brown (**if** very rarely pterostigma mostly yellow but with brown margins and palpi, tegula and humeral complex dark brown **then** T1 and T2 mostly sculptured)	**80**
80(79)	Comparatively darker colored species, with all legs entirely dark brown to black (except for yellow-brown on anterior 0.1 and/or posterior 0.1 of femur, anterior 0.1–0.3 of metatibia, and sometimes part of trochantellus); pterostigma mostly yellow or strong yellow-white, with brown margins	**81**
–	Paler colored species, legs always with some segments pale (yellow); pterostigma entirely brown or mostly brown with pale spot at base	**82**
81(80)	Palpi, tegula and humeral complex dark brown; most veins in fore wing brown; pterostigma mostly yellow with brown margins; comparatively larger species, body length 3.20–3.50 mm, fore wing length 3.50–3.80 mm [Ecuador, 4,000–4,100 m]	***D.virgendelparamo* Fernandez-Triana & Boudreault, sp. nov.**
–	Palpi yellow, tegula pale yellow-brown, humeral complex half yellow and half brown; most veins in fore wing yellow-white; pterostigma strong yellow-white with very thin brown margins; body length 2.80 mm, fore wing length 3.10 mm [Colombia, 2,900 m]	***D.lacochaparamo* Fernandez-Triana & Boudreault, sp. nov.**
82(80)	Ovipositor sheath slightly shorter than metatibia length	**83**
–	Ovipositor sheath clearly longer (1.15–1.25×) than metatibia length	**84**
83(82)	F15 cubic, its length 1.1× its width; T2 mostly sculptured, except for central area; tegula yellow; mesosternum entire black [Costa Rica. Host: *Telphusa* BioLep476 (Gelechiidae)]	***D.weaversway* Fernandez-Triana & Boudreault, sp. nov.**
–	F15 rectangular, its length 1.6× its width; T2 mostly smooth, except along posterior margin; tegula dark brown; mesosternum black but with contrasting pale brown stripe [Costa Rica. Gregarious, host: *Microscahedialis* (Thyrididae). BINBOLD:AAM1098]	***D.beryllacosteae* Fernandez-Triana & Boudreault, sp. nov.**
84(82)	T1 mostly smooth and shiny (sculpture limited along lateral margins on posterior 0.5); overall body comparatively mostly smooth and shiny; body color comparatively paler colored, mostly yellow-brown or reddish brown, including pale brown antenna; trochantelli and tegula yellow, humeral complex partially yellow and partially brown [Costa Rica. Solitary, host: *Banisia* myrsusalisDHJ01 (Thyrididae). BINBOLD:AAL2285]	***D.alixhamiltonae* Fernandez-Triana & Boudreault, sp. nov.**
–	T1 mostly sculptured on posterior 0.5 or more; overall body comparatively less smooth and shiny; body color comparatively darker colored, mostly dark brown to black, including dark brown to black antenna; trochantelli, tegula and humeral complex variable but not as above	**85**
85(84)	Tegula and humeral complex yellow; meso- and metatrochantelli dark brown to black	**86**
–	Tegula and humeral complex dark brown; trochantelli mostly yellow to yellow-brown	**87**
86(85)	Body entirely black; T1 slightly broadening towards posterior margin, 1.1–1.2× as long as its width at posterior margin; comparatively larger species, body length 2.80–2.90 mm, fore wing length 3.10–3.30 mm [Costa Rica. Solitary, host: Gelechiidae. BINBOLD:AAT8840]	***D.luishamiltoni* Fernandez-Triana & Boudreault, sp. nov.**
–	Body mostly dark brown to pale brown; T1 parallel-sided, 1.3× as long as its width at posterior margin; comparatively smaller species, body length 2.30 mm, fore wing length 2.60 mm [Brazil]	***D.encruzilhada* Fernandez-Triana & Boudreault, sp. nov.**
87(85)	Fore wing venation mostly dark brown; pterostigma with small pale yellow-brown spot on anterior 0.1 which is poorly defined; posterior 0.5 of propodeum (beyond transverse carinae of areola) mostly smooth; T2 mostly smooth [Costa Rica. BINBOLD:AAM5849]	***D.antjevirkusae* Fernandez-Triana & Boudreault, sp. nov.**
–	Fore wing venation mostly pale brown to yellow-brown; pterostigma with comparatively large bright yellow-white spot on anterior 0.2 which is clearly defined; posterior 0.5 of propodeum (beyond transverse carinae of areola) mostly striated; T2 mostly sculptured [Costa Rica. Gregarious, hosts: *Anadasmus* Janzen25, *Stenoma* Janzen27 (Depressariidae). BINBOLD:AAK2061]	***D.fredhicksi* Fernandez-Triana & Boudreault, sp. nov.**
88(78)	Comparatively darker colored species, with all legs entirely dark brown to black (except for yellow-brown on posterior 0.1 of pro- and mesofemora and anterior 0.1–0.2 of tibiae); palpi, tegula and humeral complex dark brown	**89**
–	Paler colored species, legs always with some segments pale (yellow); coloration of palpi, tegula and humeral complex variable but never the three dark brown	**90**
89(88)	T1 very strongly narrowing near posterior margin (its length 4.5× its width at posterior margin, and width at anterior margin 3.0× width at posterior margin); propodeum with almost no traces of areola, with only some small, poorly defined carinae from nucha; vein R1 shorter than pterostigma length and approx. as long as than distance between its end and end of vein 3RSb; pterostigma comparatively narrow (3.0× as long as wide) and often with lower anterior margin angulated so that it looks as having four sides; vein r arising from apical 0.7 of pterostigma [Male specimens with some variation in venation, shape of T1 and shape and sculpture of T2] [Ecuador, Peru]	***D.rubymacpearsae* Fernandez-Triana & Boudreault, sp. nov.**
–	T1 narrowing near posterior margin but not as strongly (its length 3.0× its width at posterior margin, and width at anterior margin 2.0× width at posterior margin); propodeum with areola defined on posterior 0.5; vein R1 longer than pterostigma length and much longer than distance between its end and end of vein 3RSb; pterostigma broader and not angulated at lower anterior margin; vein r usually arising at ~ 0.5 of pterostigma length [Chile. BINBOLD:AAH1316]	***D.alerce* Fernandez-Triana & Boudreault, sp. nov.**
90(88)	T1 strongly narrowing near posterior margin (T1 length ~ 4.0× its width at posterior margin); ***and*** following areas entirely to mostly smooth: scutellar disc, most of propodeum, T1 and T2	**91**
–	T1 not strongly narrowing near posterior margin (usually T1 length < 2.5× its width at posterior margin, often much less), **if** rarely T1 ~ 3.0× its width at posterior margin **then** T1 evenly narrowing from anterior to posterior margin or narrowing on posterior 0.3 only; **and** some or all of the following areas not smooth but variably sculptured: scutellar disc, propodeum, T1 and T2	**93**
91(90)	Propodeum with carinae clearly defining a more or less complete areola; ovipositor sheath comparatively much shorter, its length 0.5× metatibia length; T1 evenly narrowing towards posterior margin (T1 width at anterior margin 2.2× T1 width at posterior margin); legs mostly yellow or pale brown [Costa Rica]	***D.carlosviquezi* Fernandez-Triana & Boudreault, sp. nov.**
–	Propodeum with short carinae weakly defining an areola only on posterior half or less; ovipositor sheath comparatively much longer, ≥ 0.75× metatibia length; T1 strongly to very strongly narrowing near posterior margin; most legs dark brown to black	**92**
92(91)	F15 comparatively much longer, length/width 1.65–1.75×; ovipositor sheath comparatively shorter, its length 0.75–0.80× metatibia length; T1 comparatively thinner, its length 3.0× or more its maximum width, and T1 width at anterior margin 3.0 × T1 width at posterior margin [Costa Rica, Saint Vincent, Trinidad & Tobago. Solitary, hosts: *Antaeotricha* spp. (Depressariidae). BINBOLD:AAN2497]	***D.alanflemingi* Fernandez-Triana & Boudreault, sp. nov.**
–	F15 comparatively much shorter, sub-cubic, length/width 1.15×; ovipositor sheath comparatively longer, its length 1.15× metatibia length; T1 comparatively thicker, its length 2.0× its maximum width, and T1 width at anterior margin 2.0× T1 width at posterior margin [Costa Rica. BINBOLD:AAD5258]	***D.annychaverae* Fernandez-Triana & Boudreault, sp. nov.**
93(90)	T1 evenly narrowing from anterior to posterior margin and ~ 3.0× its width at posterior margin; ***and*** ovipositor sheath very short, < 0.55× metatibia length [Costa Rica. BINBOLD:AAM5852]	***D.mehdirheljari* Fernandez-Triana & Boudreault, sp. nov.**
–	T1 more or less parallel-sided or only narrowing on posterior 0.3 and 2.0× or less its width at posterior margin **and/or** ovipositor sheath longer than 0.6× metatibia length	**94**
94(93)	Comparatively darker colored species, with tegula and humeral complex dark brown, all legs brown to dark brown (except sometimes for yellow protibia), tergites dark brown to black, all sternites and hypopygium dark brown; **and** T2 mostly covered by weak sculptured; **and** ovipositor sheath length ~ 1.2× metatibia length; **and** T1 mostly sculptured and parallel-sided but narrowing on posterior 0.3 [Chile]	***D.nothofagus* Fernandez-Triana & Boudreault, sp. nov.**
–	Comparatively paler colored species, with at least some body areas yellow or yellow-orange; **and/or** T2 smooth or mostly sculptured with longitudinal striae; **and/or** ovipositor sheath length different **and/or** T1 shape and sculpture often different than above	**95**
95(94)	Ovipositor sheath clearly longer than metatibia length (≥ 1.25×, usually more)	**96**
–	Ovipositor sheath clearly shorter or approx. same length than metatibia (0.60–1.10×)	**106**
96(95)	Sculpture of T1 and T2 different, T1 mostly sculptured with longitudinal striae on posterior 0.5–0.6 (but sometimes with central smooth area near posterior margin) **and** T2 entirely smooth [Brazil]	***D.robmacewani* Fernandez-Triana & Boudreault, sp. nov.**
–	Sculpture of T1 and T2 similar, ***either*** T1 and T2 mostly to entirely smooth ***or*** T1 and T2 mostly sculptured	**97**
97(96)	T1 mostly smooth, at most with weak sculpture on posterior 0.3; T2 smooth	**98**
–	T1 mostly sculptured with longitudinal striae; T2 entirely to mostly sculptured (at most with small, smooth area centrally)	**101**
98(97)	Ovipositor tip apically sinuate; T2 smooth [Costa Rica. BINBOLD:AAM5750]	***D.kasiiya* Fernandez-Triana & Boudreault, sp. nov.**
–	Ovipositor tip not sinuate, if with tip weakly sinuate, then T2 strongly sculptured	**99**
99(98)	Propodeum with complete areola; tegula and humeral complex white or yellow; pterostigma much paler colored, either mostly yellow-white with thin brown margins, or very pale brown (almost transparent) with small, paler spot at base	**100**
–	Propodeum without areola, with some striae on posterior margin near nucha; tegula and humeral complex dark brown; pterostigma mostly dark brown with pale spot at base [Canada, Cuba, United States. Solitary, host: *Homoeosomaelectellum* (Pyralidae). Partial sequences available]	***D.homoeosomae* (Muesebeck, 1933)**
100(99)	Ovipositor sheath shorter than metasoma length and 1.3× as long metatibia length; anteromesoscutum coarsely punctured; T1 with some weak sculpture on posterior 0.5, especially along margins [Costa Rica. Solitary, host: *Antaeotricha* Janzen221 (Depressariidae). BINBOLD:AAY4695]	***D.kenzabaddouae* Fernandez-Triana & Boudreault, sp. nov.**
–	Ovipositor sheath longer than metasoma length and 1.6–1.7× as long as metatibia length; anteromesoscutum very finely punctate anteriorly, smooth and shiny posteriorly; T1 smooth [Colombia, Dominican Republic, Puerto Rico, Saint Vincent, Trinidad & Tobago]	***D.politiventris* Muesebeck, 1958**
101(97)	T2 with central smooth area; T2 width at posterior margin < 3.0× its central length	**102**
–	T2 entirely sculptured; T2 width at posterior margin > 3.5× (usually > 4.0×) its central length	**103**
102(101)	Tegula black, humeral complex dark brown; profemur and protibia mostly brown to dark brown; all trochantelli dark brown to black; pterostigma entirely brown [Costa Rica. BINBOLD:AAM5739]	***D.dole* Fernandez-Triana & Boudreault, sp. nov.**
–	Tegula yellow, humeral complex half yellow half brown; profemur and protibia yellow; pro- and mesotrochantelli yellow; pterostigma with pale spot at base [Ecuador]	***D.puyo* Fernandez-Triana & Boudreault, sp. nov.**
103(101)	Ovipositor tip weakly sinuate; pterostigma with pale spot very small, 0.1 pterostigma length [Costa Rica. BINBOLD:AAX8664]	***D.rudyamadori* Fernandez-Triana & Boudreault, sp. nov.**
–	Ovipositor tip slightly and evenly curving downwards but not sinuate; pterostigma pale spot 0.2–0.3 pterostigma length	**104**
104(103)	All trochantelli yellow; profemur entirely and mesofemur mostly (except for longitudinal brown bands on margins) yellow [Costa Rica. Solitary, host: *Dichomeris* designatellaDHJ04 (Gelechiidae). BINBOLD:AAI6323]	***D.susanabramsae* Fernandez-Triana & Boudreault, sp. nov.**
–	All trochantelli dark brown; anterior 0.3–0.4 of profemur and entire mesofemur brown	**105**
105(104)	Most veins transparent to yellow white; pterostigma comparatively elongate, its length > 3.0× its maximum height [Costa Rica, Mexico. BINBOLD:AAX8653]	***D.moniquegilbertae* Fernandez-Triana & Boudreault, sp. nov.**
–	Most veins yellow-brown or brown; pterostigma comparatively thicker, its length ~ 2.0× its maximum width [United States. Solitary, hosts: *Dioryctria* spp, *Rhyacionia* spp. (Pyralidae, Tortricidae)]	***D.bushnelli* (Muesebeck, 1933)**
106(95)	Comparatively darker colored species, with tegula and humeral complex dark brown, all legs brown to dark brown (except for yellow protibia), tergites dark brown to black, all sternites and hypopygium dark brown [Peru]	***D.machupichu* Fernandez-Triana & Boudreault, sp. nov.**
–	Comparatively paler colored species, with at least some body areas yellow or yellow-orange	**107**
107(106)	Scutellar disc mostly with shallow punctures; **and** T1 relatively thinner, with median length 2.0–2.2× its width at posterior margin; **and** T1 with central hump; **and** tegula and humeral complex yellow; **and** mesofemur, metatibia (except for small pale spot on anterior 0.1) and metatarsus dark brown [Costa Rica. BINBOLD:AAI9747]	***D.jasonkelleyi* Fernandez-Triana & Boudreault, sp. nov.**
–	Scutellar disc smooth and shiny, without punctures; **either** with T1 relatively thicker, with median length < 2.0× its width at posterior margin; **and/or** T1 without central hump; **and/or** tegula and humeral complex darker; **and/or** mesofemur, metatibia and metatarsus partially or entirely pale colored	**108**
108(107)	Pterostigma pale (yellow or white-yellow), at most with thin margins which are slightly darker	**109**
–	Pterostigma mostly dark (brown), at most with small pale spot on anterior 0.1 or, very rarely, with small spot centrally which is slightly paler than rest of pterostigma	**111**
109(108)	Tegula and humeral complex yellow; anteromesoscutum with punctures on most of its surface and scutellar disc with some punctures [Costa Rica. BINBOLD:AAE8602]	***D.josephfridmani* Fernandez-Triana & Boudreault, sp. nov.**
–	Tegula and humeral complex dark brown; anteromesoscutum and scutellar disc mostly smooth and shiny	**110**
110(109)	Pterostigma bright yellow-white; T2 entirely smooth; T1 mostly smooth, with weak punctures centrally; fore wing vein R1 longer than pterostigma and > 3.5× as long as the space between its end and end of vein 3RSb [Chile]	***D.moniqueae* Fernandez-Triana & Boudreault, sp. nov.**
–	Pterostigma pale yellow-brown but with thin brown margins; T2 mostly sculptured, with small area smooth along posterior margin; T1 rugose on posterior half; fore wing vein R1 around same length (sometimes shorter) than pterostigma and 3.0× or less as long as the space between its end and end of vein 3RSb [Algeria, Chile, Colombia, Peru, Spain, Venezuela. Solitary, hosts: *Phthorimaeaoperculella*, *Keiferialycopersicella*, *Tutaabsoluta* (Gelechiidae)]	***D.gelechiidivoris* (Marsh, 1975)**
111(108)	Metatibia mostly yellow (except for dark brown spot on posterior 0.1–0.2); part of metafemur (anterior 0.1–0.2 and posterior 0.1) yellow	**112**
–	Metatibia ≥ 0.5–0.8 dark brown; metafemur usually almost entirely dark brown to black	**113**
112(111)	T1 comparatively wider, its median length 1.25× its width at posterior margin; humeral complex yellow, same color than tegula; fore wing vein 1CU medially raised or arched in a sharp angle [Costa Rica. Solitary, hosts: *Gonionota* Janzen22 (Depressariidae), unidentified Gelechiidae with interim name ‘gelJanzen01 Janzen23’. BINBOLD:AAI9755]	***D.christinaagapakisae* Fernandez-Triana & Boudreault, sp. nov.**
–	T1 comparatively thinner, its median length 1.65× its width at posterior margin; humeral complex almost entirely brown, clearly darker than yellow tegula; fore wing vein 1CU straight [Costa Rica, French Guiana. Solitary, host: unidentified Depressariidae. BINBOLD:ACI3397]	***D.sigifredomarini* Fernandez-Triana & Boudreault, sp. nov.**
113(111)	Tegula yellow	**114**
–	Tegula dark brown to black	**119**
114(113)	Humeral complex yellow; T2 mostly to entirely smooth and comparatively very transverse, its width at posterior margin 4.0× its central length; T1 mostly smooth (only with fine sculpture laterally near posterior margin) [Caribbean species]	**115**
–	Humeral complex mostly to entirely brown; T2 mostly sculptured (at least around margins) and comparatively less transverse, its width at posterior margin 2.2–3.0× its central length; T1 with some sculpture, sometimes strong on posterior half [Central and South America species]	**116**
115(114)	Propodeum mostly smooth and without defined areola; pterostigma entirely brown; mesoscutum shiny and with extremely shallow and separate punctures; lunules semicircular and comparatively short; fore wing with vein r arising well beyond middle of pterostigma; metafemur mostly yellow-brown (sometimes darker on upper and lower margins), metatibia darker on posterior 0.2 or less; body color, including coxae, mostly black [Puerto Rico. Probably gregarious, hosts: *Marucavitrata*, *Omiodesindicata* (Pyralidae)]	***D.hedyleptae* (Muesebeck, 1958)**
–	Propodeum mostly with punctures and rugulosities and with complete and strongly defined areola; pterostigma with pale spot on anterior 0.3; lunules triangular and comparatively very high; fore wing with vein r arising around middle of pterostigma; metafemur and posterior 0.5 of metatibia dark reddish brown; body color, including coxae, mostly dark reddish brown [Trinidad & Tobago. Solitary, host: *Oiketicuskirbyi* (Psychidae)]	***D.oiketicus* Fernandez-Triana & Boudreault, sp. nov.**
116(114)	T1 2.2× as long as wide at posterior margin [Costa Rica. BINBOLD:AAI9746]	***D.alejandromarini* Fernandez-Triana & Boudreault, sp. nov.**
–	T1 1.3–1.7× as long as wide at posterior margin	**117**
117(116)	Ovipositor sheath clearly shorter than metatibia length (0.7×) [Costa Rica. Solitary, host: unidentified Pyralidae. BINBOLD:ABX5620]	***D.luzmariaromeroae* Fernandez-Triana & Boudreault, sp. nov.**
–	Ovipositor sheath as long as metatibia or longer	**118**
118(117)	Anteromesoscutum mostly with rather coarse punctures; propodeum with almost complete areola (open anteriorly); ocelli comparatively smaller, ocular ocellar line > 3.0× diameter of posterior ocellus; pterostigma mostly dark brown (with pale spot on anterior 0.1 or less) [Costa Rica. Solitary, hosts: *Olethreutes* spp. (Tortricidae). BINBOLD:AAE8612]	***D.anacamposae* Fernandez-Triana & Boudreault, sp. nov.**
–	Anteromesoscutum mostly shiny, punctures sparse, superficial and fading towards posterior margin; propodeum without defined areola but with faint U-shaped depression medially; ocelli comparatively larger, ocular ocellar line ~ 2.0× diameter of posterior ocellus; pterostigma mostly pale brown (with pale spot anteriorly) [Chile, Juan Fernández islands]	***D.evadne* (Nixon, 1955)**
119(113)	T2 entirely to mostly smooth (if there is some sculpture, it is limited to margins of tergite)	**120**
–	T2 entirely to mostly sculptured	**122**
120(119)	Gena with white spot; smaller size, body length and fore wing length 1.80 mm; propodeum areola open anteriorly, propodeum generally with relatively few sculptures or carinae [Costa Rica. BINBOLD:AAM5853]	***D.puschendorfi* Fernandez-Triana & Boudreault, sp. nov.**
–	Gena without white spot; larger size, body length and fore wing length 2.40–2.60 mm; propodeum areola complete, propodeum generally sculptured and with well-defined carinae	**121**
121(120)	Comparatively darker colored specimen, with antenna pedicel, most of mesofemur and entire mesosternum dark brown to black; propodeum mostly sculptured, with lateral striation on most areas between carinae; anteromesoscutum mostly covered with relatively coarse punctures; T1 slightly narrowing near posterior margin; T2 smooth [Costa Rica. BINBOLD:AAJ1390]	***D.timrichi* Fernandez-Triana & Boudreault, sp. nov.**
–	Comparatively paler colored specimen, with pedicel, most of mesofemur and longitudinal band on mesosternum yellow to yellow-brown; propodeum mostly smooth, with few areas between carinae with some striation; anteromesoscutum punctures less coarse on posterior 0.3–0.4; T1 slightly widening near posterior margin; T2 usually with some sculpture [Costa Rica. Gregarious, hosts: *Microsca* spp. (Thyrididae). BINBOLD:ABX6008]	***D.sarahoconnorae* Fernandez-Triana & Boudreault, sp. nov.**
122(119)	Ovipositor sheath distinctly shorter than metatibia length (< 0.7×)	**123**
–	Ovipositor sheath slightly longer (1.1–1.2×) than metatibia length	**124**
123(122)	T1 clearly narrowing towards posterior margin (T1 width at anterior margin 1.2 × T1 width at posterior margin) and comparatively less broad (T1 length 2.2 × T1 width at posterior margin); T2 trapezoidal, its width at posterior margin ~ 2.0× its central length; propodeum areola less defined anteriorly; profemur brown on anterior ~ 0.5; metatibia with anterior 0.2 yellow-white, posterior 0.8 dark brown [Costa Rica. BINBOLD:AAM5851]	***D.jessiehillae* Fernandez-Triana & Boudreault, sp. nov.**
–	T1 parallel-sided (same T1 width at anterior and posterior margins) and comparatively much broader (T1 length 1.2 × T1 width at posterior margin); T2 transverse, its width at posterior margin > 3.0× its central length; propodeum areola well defined and bounded by carinae; profemur entirely yellow; metatibia with anterior 0.4 yellow, posterior 0.6 brown [Costa Rica. Solitary, host: unidentified Gelechiidae. BINBOLD:ACB1629]	***D.stevestroudi* Fernandez-Triana & Boudreault, sp. nov.**
124(122)	Anterior 0.5 of profemur and entire mesofemur brown; **and** antenna shorter than body length; and comparatively smaller size, body length 2.10 mm, fore wing length 2.30 mm [Brazil]	***D.stephmae* Fernandez-Triana & Boudreault, sp. nov.**
–	Pro- and mesofemora usually entirely yellow (**if** rarely femora darker, **then** antenna much longer than body and comparatively larger size, body length 2.60 mm, fore wing length 2.75 mm); antenna usually as long as body length or longer; usually species larger than 2.00 mm	**125**
125(124)	Anterior 0.5 of profemur and entire mesofemur brown; ovipositor sheath distinctly (> 1.2×) longer than metatibia length [Costa Rica. Solitary, hosts: *Chlamydastisvividella*, *Stenoma* Janzen199 (Depressariidae). BINBOLD:ACC1295]	***D.bradzlotnicki* Fernandez-Triana & Boudreault, sp. nov.**
–	Pro- and mesofemora entirely yellow; ovipositor sheath from slightly longer (1.1× or less) to distinctly shorter (< 0.8×) than metatibia length	**126**
126(125)	Comparatively longer F15, its length > 1.5× its width; usually body comparatively less shiny and less smooth; sculpture on anteromesoscutum and propodeum coarser and more deeply indicated; comparatively darker colored, with paler areas (such as longitudinal strip on metasternum and most of first pair of legs) yellow to yellow-brown [Costa Rica. Solitary, host: *Microscapaullula* (Thyrididae). BINBOLD:AAC2174]	***D.scottmilleri* Fernandez-Triana & Boudreault, sp. nov.**
–	Comparatively shorter, cubic, F15, its length ≤ 1.1× its width; body comparatively shinier and smoother; sculpture on anteromesoscutum and propodeum shallower; comparatively paler colored with some areas (such as longitudinal strip on metasternum and most of first pair of legs) bright yellow to white-yellow [Costa Rica. Gregarious, host: *Collinsaferreiceps* (Thyrididae)]	***D.robpringlei* Fernandez-Triana & Boudreault, sp. nov.**

### ﻿﻿Taxonomic treatment of species, in alphabetical order

#### ﻿ Dolichogenidea aceituno

Taxon classificationAnimaliaHymenopteraBraconidae

Fernandez-Triana & Boudreault sp. nov.

A78795FC-F69B-5098-8DB0-21EDD0665D06

https://zoobank.org/1930F0BD-E5B4-4F6E-823C-52166B8B060D

Fig. 6A–H

##### Type material.

***Holotype*.** Chile • Female, CNC; Atacama, Chanaral de Aceituno; 12.x.1958; L. E. Pena leg.; Voucher code: CNC1196525. ***Paratypes*.** Chile • 3 Males, CNC; CNC1196544, CNC1196545, CNC1196567.

**Figure 6.**
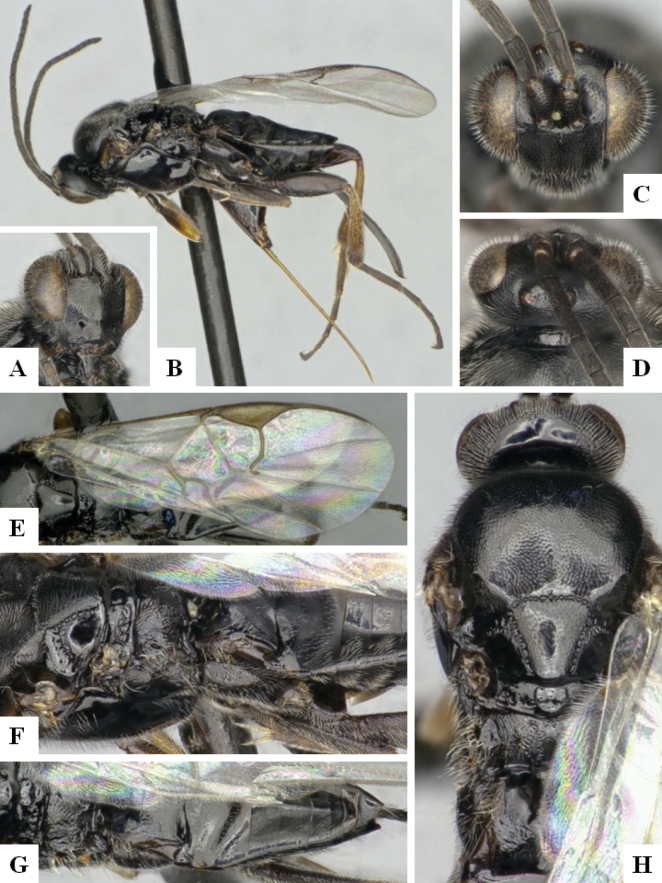
*Dolichogenideaaceituno* Fernandez-Triana & Boudreault holotype female CNC1196525 **A** head, fronto-lateral **B** habitus, lateral **C** head, frontal **D** head, dorsal **E** wings **F** propodeum & T1–T3, dorsal **G** metasoma, dorsal **H** mesosoma, dorsal.

##### Diagnostic description.

Face rostriformis (malar space longer than mandible width) and with slightly elongate mouth parts; T1 and T2 entirely smooth and shiny; T1 very broad at posterior margin, almost quadrate, its length 1.05× its width at posterior margin; ovipositor sheath 1.5× metatibia length; fore wing veins mostly transparent; pterostigma mostly yellow but with brown margins; palpi, tegula and humeral complex dark brown; all coxae black to dark brown; profemur dark brown on posterior half, meso- and metafemora entirely dark brown; body length: 3.70 mm; fore wing length: 3.90 mm. Among all species with smooth T1 and T2, *D.aceituno* is very distinctive because of its elongate face and mouth parts, dark coloration of coxae and femora and broad T1.

##### Distribution.

Chile.

##### Biology.

No host data available.

##### DNA barcoding data.

No data.

##### Etymology.

Named after the type locality, the Chañaral de Aceituno, in the Chanaral Island (Isla Chanaral) where the Pingüino de Humboldt National Reserve, an important nature reserve for marine wildlife, is located.

#### ﻿ Dolichogenidea acrobasidis

Taxon classificationAnimaliaHymenopteraBraconidae

(Muesebeck), 1921

3EAE2BF1-5511-5F42-9039-B4DD6A4D8024

Figs 7A–H , 8A, B

##### Notes.

This species has not been found in the Neotropical region, but it is included in the key above because of its occurrence in southern USA states (Florida and Mississippi).

**Figure 7.**
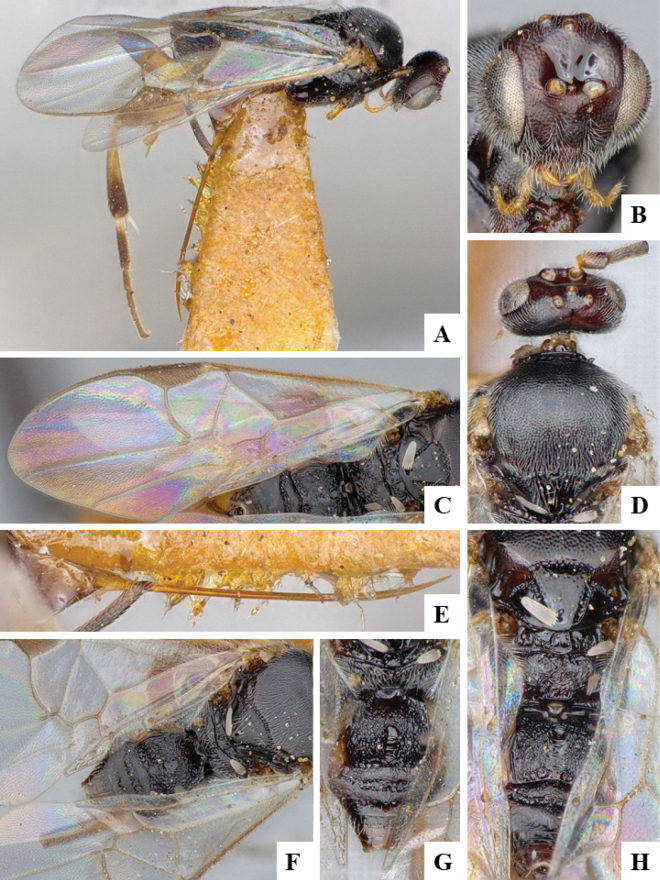
*Dolichogenideaacrobasidis* (Muesebeck) female CNCHYM00971 **A** habitus, lateral **B** head, frontal **C** wings **D** head & mesoscutum, dorsal **E** ovipositor **F** propodeum & metasoma, dorsal **G** metasoma, dorsal **H** propodeum & T1–T3, dorsal.

**Figure 8.**
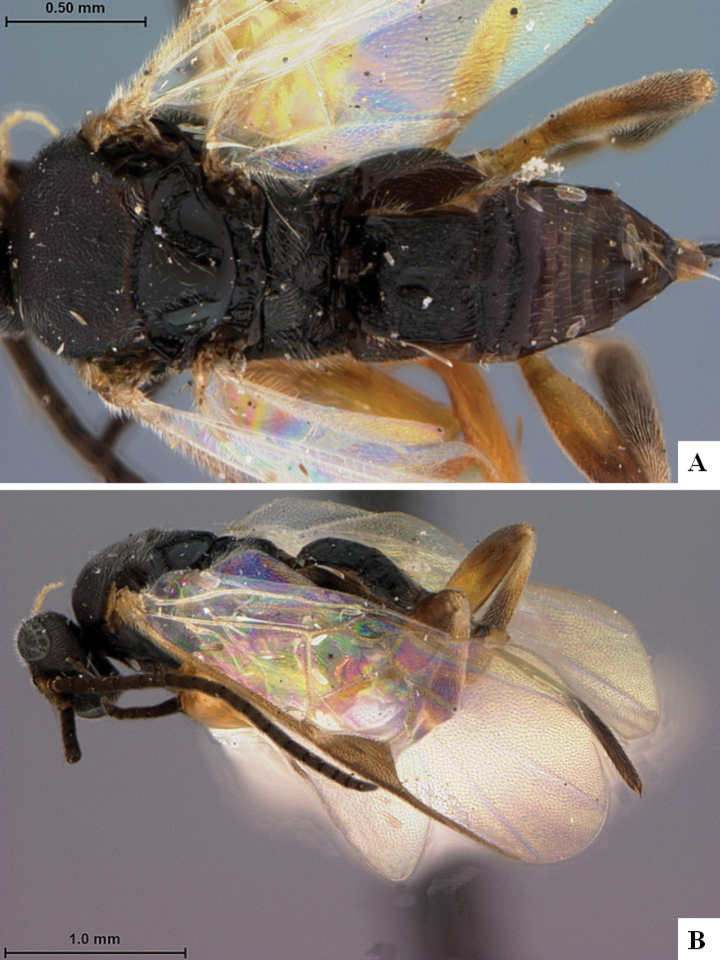
*Dolichogenideaacrobasidis* (Muesebeck) holotype female **A** habitus, dorsal **B** habitus, lateral.

#### ﻿ Dolichogenidea alanflemingi

Taxon classificationAnimaliaHymenopteraBraconidae

Fernandez-Triana & Boudreault sp. nov.

45E67219-794B-5380-B6EF-42A920D35AE7

https://zoobank.org/658B69AE-01C9-4F50-A6E0-05FE0A69618D

Figs 9A–E , 151A

##### Type material.

***Holotype*.** Costa Rica • Female, CNC; Guanacaste, Area de Conservación Guanacaste, Pasmompa, Sector Pitilla; 11°1'8.40"N, 85°24'36"W; 440 m; 17.iii.2010; Calixto Moraga leg.; Host: *Antaeotricha* Janzen146; Voucher code: DHJPAR0039472; Host voucher code: 10-SRNP-30807.

**Figure 9.**
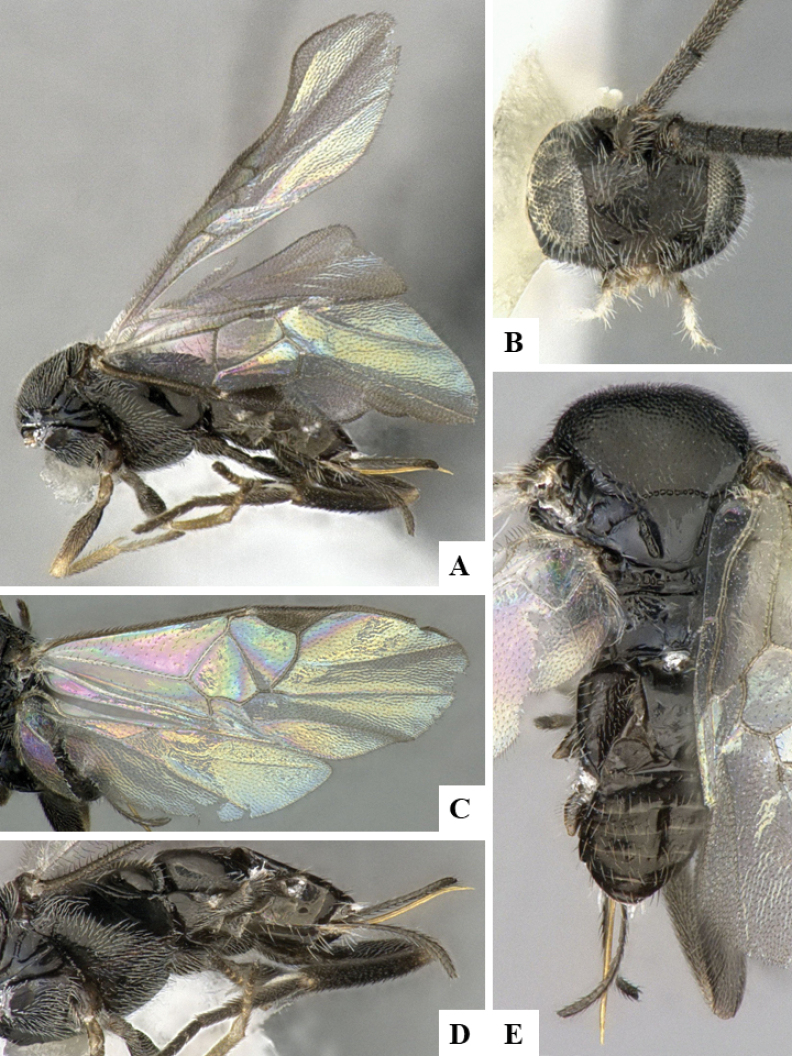
*Dolichogenideaalanflemingi* Fernandez-Triana & Boudreault holotype female DHJPAR0039472 **A** habitus, lateral **B** head, frontal **C** wings **D** metasoma, lateral **E** habitus, dorsal.

##### Other material.

Saint Vincent • 1 Female, CNC; CNC1196962; Trinidad & Tobago • 2 Females, CNC; CNC1179943, CNC1179701.

##### Diagnostic description.

Overall body mostly shiny and smooth, including scutellar disc and most of propodeum (except for weak carinae defining a partial areola); T1 strongly narrowing near posterior margin (T1 length > 3.0× its width at posterior margin); T1 and T2 smooth; ovipositor sheath 0.8× as long as metatibia length; legs mostly dark brown (except for tibiae and tarsi of first two pairs of legs); body length: 1.70 mm; fore wing length: 2.15 mm. Among all species with dark coxae and smooth T1 and T2, *D.alanflemingi* is distinguished by shape of T1, overall body sculpture, small body size and ovipositor sheath length. *Dolichogenideaannychaverae* is morphologically similar but has F15 comparatively much shorter, ovipositor sheath comparatively longer, T1 comparatively thicker and with different shape (see key for better diagnosis of these two species). They also have different DNA barcodes.

##### Distribution.

Costa Rica, Saint Vincent, Trinidad & Tobago.

##### Biology.

Solitary. Depressariidae: *Antaeotricha* Janzen49, *Antaeotricha* Janzen146.

##### DNA barcoding data.

BINBOLD:AAN2497 (2 sequences, 2 barcode compliant).

##### Etymology.

Named in honor of Mr. Alan Fleming of the Diptera Division of the Canadian National Collection in Ottawa in recognition of his solid full-time 10 years, and ongoing, taxonomizing of the Tachinidae of Area de Conservación Guanacaste in northwestern Costa Rica.

##### Notes.

The specimens from Trinidad & Tobago and Saint Vincent are very similar but body coloration is paler, more dark reddish brown than black, therefore they are not included as paratypes.

#### ﻿ Dolichogenidea alejandromarini

Taxon classificationAnimaliaHymenopteraBraconidae

Fernandez-Triana & Boudreault sp. nov.

377E7356-440B-5055-8DA4-2BE087018636

https://zoobank.org/21DB0B77-6609-40A1-BE53-BC723268903E

Fig. 10A–H

##### Type material.

***Holotype*.** Costa Rica • Female, CNC; Guanacaste, Area de Conservación Guanacaste, Sector Santa Rosa, Area Administrativa; 10.83764, -85.61871; 295 m; 25.xii.2008; D. H. Janzen & W. Hallwachs leg.; Malaise trap; Voucher code: DHJPAR0031574. ***Paratype*.** Costa Rica • 1 Male, CNC; DHJPAR0012560.

**Figure 10.**
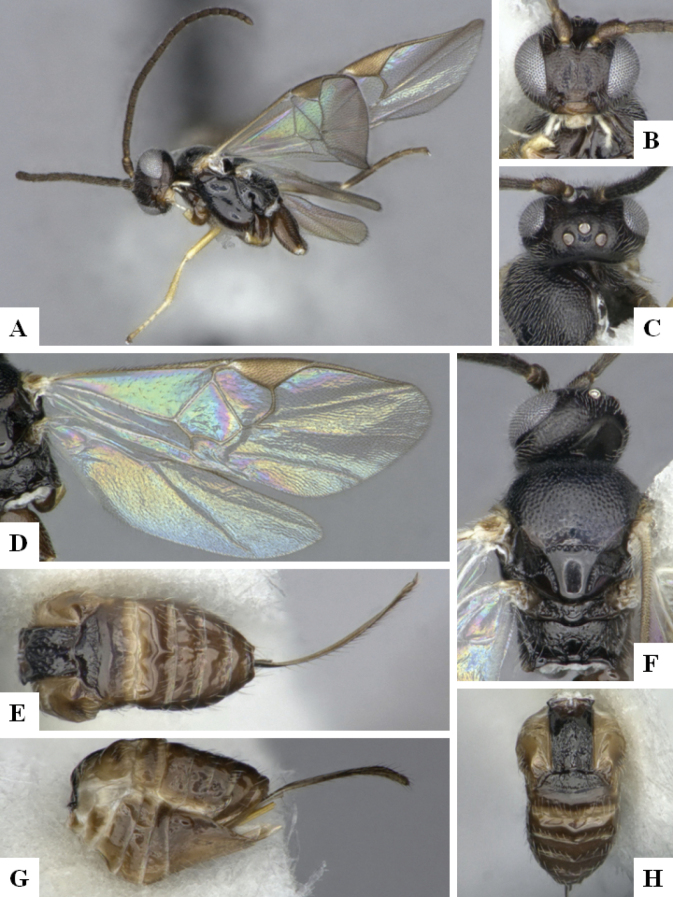
*Dolichogenideaalejandromarini* Fernandez-Triana & Boudreault holotype female DHJPAR0031574 **A** habitus, lateral **B** head, frontal **C** head, dorsal **D** wings **E** metasoma, dorsal **F** mesosoma, dorsal **G** metasoma, lateral **H** T1–T3, dorsal.

##### Diagnostic description.

Propodeum with complete areola; T1 2.2× as long as wide at posterior margin; T2 transverse, its width at posterior margin 3.0× its central length; T1 with some sculpture on posterior 0.5; T2 sculptured around margins, centrally smooth; tegula yellow, humeral complex half yellow half brown; all coxae, mesofemur and most of metafemur (except for anterior 0.4 which is yellow-white) brown to dark brown; body length: 1.88 mm; fore wing length: 1.98 mm. Among all species with T2 smooth, this species can be distinguished by the color of its legs, tegula and humeral complex, shape and sculpture of T1 and T2 and propodeum areola.

##### Distribution.

Costa Rica.

##### Biology.

No host data available.

##### DNA barcoding data.

BINBOLD:AAI9746 (2 sequences, 2 barcode compliant).

##### Etymology.

Named in honor of Dr. Alejandro Marin in recognition of his recent years of being an apprentice to field Director Sigifredo Marin for multiple Guanacaste Dry Forest Conservation Fund projects in and near Area de Conservación Guanacaste.

#### ﻿ Dolichogenidea alejandromasisi

Taxon classificationAnimaliaHymenopteraBraconidae

Fernandez-Triana & Boudreault, 2019

384001AE-22A3-5818-B365-BB1B75E71003

**Figure 11.**
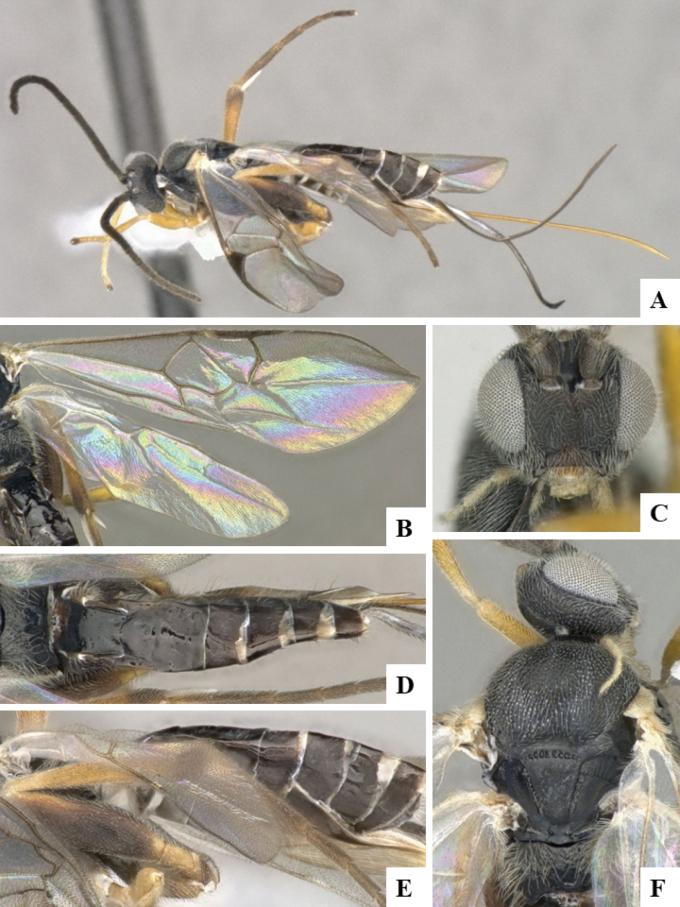
*Dolichogenideaalejandromasisi* Fernandez-Triana & Boudreault holotype female DHJPAR0035291 **A** habitus, lateral **B** wings **C** head, frontal **D** metasoma, dorsal **E** hind leg **F** mesosoma, dorsal.

Figs 11A–F , 151B , 152B

##### Notes.

Full details for this species in Fernandez-Triana et al. (2019). See also the key and Table 1 above.

#### ﻿ Dolichogenidea alerce

Taxon classificationAnimaliaHymenopteraBraconidae

Fernandez-Triana & Boudreault sp. nov.

200A0FC2-873B-57C6-9680-F3554B23D1F5

https://zoobank.org/DF43D69D-8373-496A-9E28-4535ED466A2A

Fig. 12A–G

##### Type material.

***Holotype*.** Chile • Female, CNC; Rx, Mtn Alerce, Costero, E of Mirador; 40°10'55"S, 73°26'21"W; 916 m; 2005; Brown & Berezovskiy leg.; Voucher code: CNCH2455.

**Figure 12.**
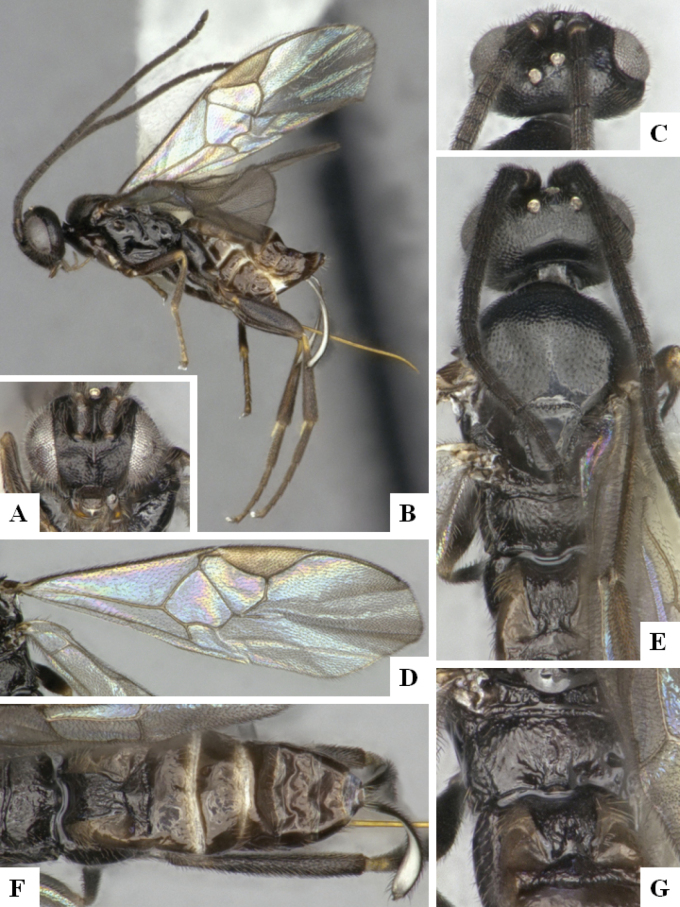
*Dolichogenideaalerce* Fernandez-Triana & Boudreault holotype female CNCH2455 **A** head, frontal **B** habitus, lateral **C** head, dorsal **D** wings **E** mesosoma, dorsal **F** metasoma, dorsal **G** propodeum, dorsal.

##### Diagnostic description.

Vein R1 longer than pterostigma length and much longer than distance between its end and end of vein 3RSb; pterostigma broader and not angulated at lower anterior margin; vein r usually arising at ~ 0.5 of pterostigma length; propodeum with areola defined on posterior 0.5; T1 narrowing near posterior margin but not as strongly (its length 3.0× its width at posterior margin, and width at anterior margin 2.0× width at posterior margin). Comparatively dark colored species, with all legs entirely dark brown to black (except for yellow-brown on posterior 0.1 of pro- and mesofemora and anterior 0.1–0.2 of tibiae); palpi, tegula and humeral complex dark brown; body length: 2.36 mm; fore wing length: 2.36 mm. Among all known species of *Dolichogenidea* in the New World, *D.alerce* can be recognized by its T1 comparatively narrow and overall dark coloration of body and legs. *Dolichogenidearubymacpearsae* is morphologically similar but it has T1 more strongly narrowing, a much shorter vein R1, and a characteristic pterostigma (see diagnostic for that species).

##### Distribution.

Chile.

##### Biology.

No host data available.

##### DNA barcoding data.

BINBOLD:AAH1316 (1 sequence, barcode compliant).

##### Etymology.

Named after the Alerce Costero National Park, where the holotype was collected. The Patagonian cypress (*Fitzroyacupressoides*) also known as “alerce”, an outstanding tree, gives its name to the park and, indirectly, to this new wasp species.

#### ﻿ Dolichogenidea alexamasisae

Taxon classificationAnimaliaHymenopteraBraconidae

Fernandez-Triana & Boudreault sp. nov.

E5948918-B169-55D5-9949-7E4456B8BF0C

https://zoobank.org/24EEB0F9-F660-4ED9-B98D-FE3340E27FAE

Figs 13A–F , 152B

##### Type material.

***Holotype*.** Costa Rica • Female, CNC; Alajuela, Area de Conservación Guanacaste, Sector Rincon Rain Forest, Estacion Botarrama; 10.9599, -85.283; 160 m; 11.i.2013; Kemberly Villalobos leg.; Host: *Rhectocraspeda* Solis05; Voucher code: DHJPAR0051056; Host voucher code: 13-SRNP-67156. ***Paratypes*.** Costa Rica, Ecuador, Venezuela • 1 Female, 2 Males, CNC; CNCH0621, CNC1180057, DHJPAR0051053.

**Figure 13.**
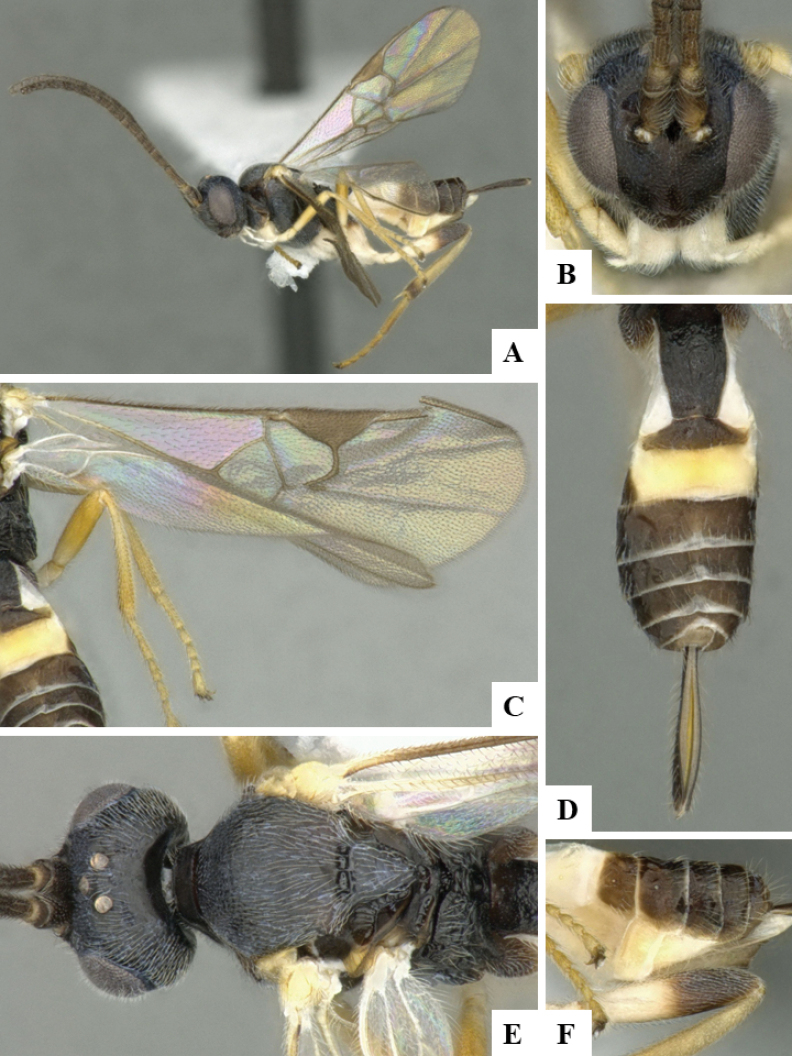
*Dolichogenideaalexamasisae* Fernandez-Triana & Boudreault holotype female DHJPAR0051056 **A** habitus, lateral **B** head, frontal **C** wings **D** metasoma, dorsal **E** head & mesosoma, dorsal **F** metasoma, lateral.

##### Diagnostic description.

T1 and T2 smooth; apical 0.3 of T1 strongly narrowing towards posterior margin, so that T1 length is > 3.0× its width at posterior margin; pterostigma uniformly colored, mostly brown to pale brown; pro- and mesocoxae yellow-white, metacoxa mostly yellow-white with brown spot on anterior 0.1; anterior 0.5 of metafemur yellow-white and posterior 0.5 brown; T3 pale yellow, contrasting with rest of tergites which are brown or dark brown; body length: 2.70–2.73 mm; fore wing length: 2.65–2.88 mm. The color of pterostigma, metafemur and T3, as well as the shape of T1 distinguish this species among all with pale pro- and mesocoxae and smooth T2.

##### Distribution.

Costa Rica, Ecuador, Venezuela.

##### Biology.

Solitary. Crambidae: *Herpetogramma* Janzen04, *Rhectocraspeda* Solis05.

##### DNA barcoding data.

BINBOLD:AAF5364 (10 sequences, 5 barcode compliant).

##### Etymology.

Named in honor of Ms. Alexa Masis in recognition of her robust participation in the family, country, and conservation life of the Boshart-Masis household for the directorate of Area de Conservación Guanacaste (ACG).

#### ﻿ Dolichogenidea alexandrei

Taxon classificationAnimaliaHymenopteraBraconidae

Fernandez-Triana & Boudreault sp. nov.

B73AFCB2-146C-5F2F-BAEB-DCCBBE02692A

https://zoobank.org/B1138CC1-F76E-47F0-A69F-83D77F21AEE4

Fig. 14A–F

##### Type material.

***Holotype*.** Guatemala • Female, CNC; Zacapa, San Lorenzo; 15°6'58.10"N, 89°37'59.91"W; 1,700 m; xi.1986; M. Sharkey leg; Voucher code: CNC650284.

**Figure 14.**
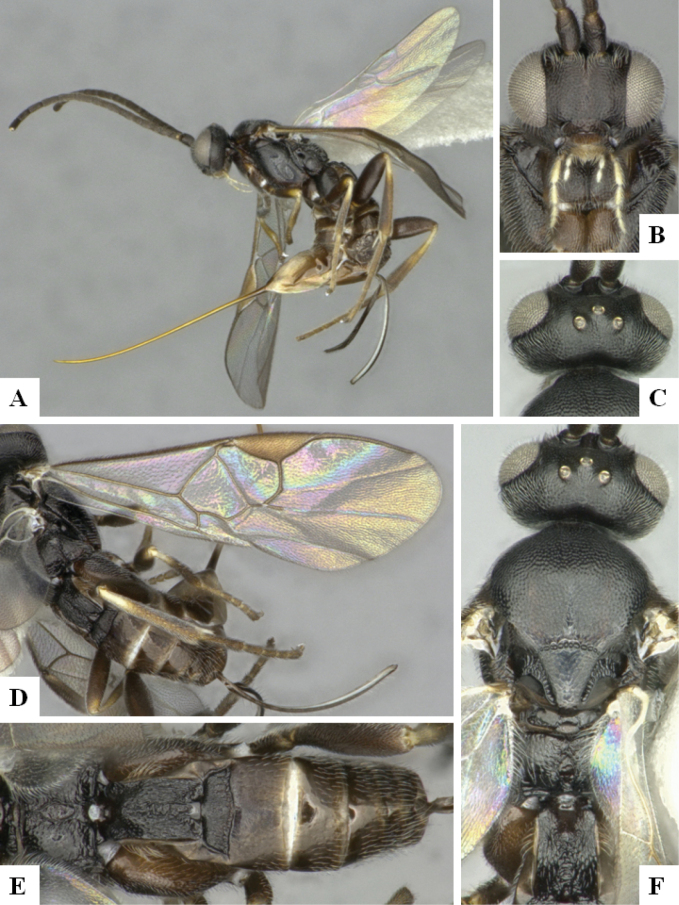
*Dolichogenideaalexandrei* Fernandez-Triana & Boudreault holotype female CNC650284 **A** habitus, lateral **B** head, frontal **C** head, dorsal **D** wings **E** metasoma, dorsal **F** mesosoma, dorsal.

##### Diagnostic description.

T1 and T2 heavily sculptured with strong longitudinal striae (rarely strong reticulated sculpture) covering entire surface of T2 and most of T1; T1 mostly parallel-sided but posterior 0.1–0.3 slightly narrowing towards posterior margin; T1 length 1.8× its width at posterior margin; T2 trapezoidal and rather small, not covering entire surface of tergum; ovipositor sheath 1.6× metatibia length; wings hyaline; tegula and humeral complex dark brown; pterostigma mostly yellow-white but with thin brown margins; legs almost entirely brown to dark brown (except for yellow protibia and protarsus, and very small, paler spots on posterior 0.1 of pro- and mesofemora and anterior 0.1–0.2 of meso- and metatibiae); body length: 2.50 mm; fore wing length: 2.88 mm. Among all species with heavily sculptured T1 and T2, this species is distinctive by the comparatively thinner T1 (≥ 2.0× its width at posterior margin), shape of T2, and color of wings and legs. It looks similar to *D.yungas* but *D.alexandrei* has a longer vein R1 in fore wing, first pair of legs paler colored, a much broader T1 and T2, longer ovipositor and ovipositor sheath and wings not infuscated.

##### Distribution.

Guatemala.

##### Biology.

No host data available.

##### DNA barcoding data.

No data.

##### Etymology.

The second author dedicates this species to her dear brother Alexandre Boudreault in appreciation for his love and support, fun times, and shared special moments. Alexandre is a genius with electronics. He always has new projects going on like a new gadget for his son’s car racetrack or a new alarm clock made from scratch!

#### ﻿ Dolichogenidea alixhamiltonae

Taxon classificationAnimaliaHymenopteraBraconidae

Fernandez-Triana & Boudreault sp. nov.

1E7A609B-9CF0-583C-A1D1-5FB728944FF9

https://zoobank.org/F6063253-B0F0-49AF-9676-CE01311EA04B

Figs 15A–F , 16A–G

##### Type material.

***Holotype*.** Costa Rica • Female, CNC; Guanacaste, Area de Conservación Guanacaste, Sector Santa Rosa, Bosque San Emilio; 10.8439, -85.6138; 300 m; 07.vii.1983; Host: *Banisia* myrsusalisDHJ01; Voucher code: CNC1179780; Host voucher code: 83-SRNP-912. ***Paratypes*.** Costa Rica • 4 Females, 1 Male, CNC; CNC5342701, CNC5342702, CNC5342703, CNC5342704, CNC5342705.

**Figure 15.**
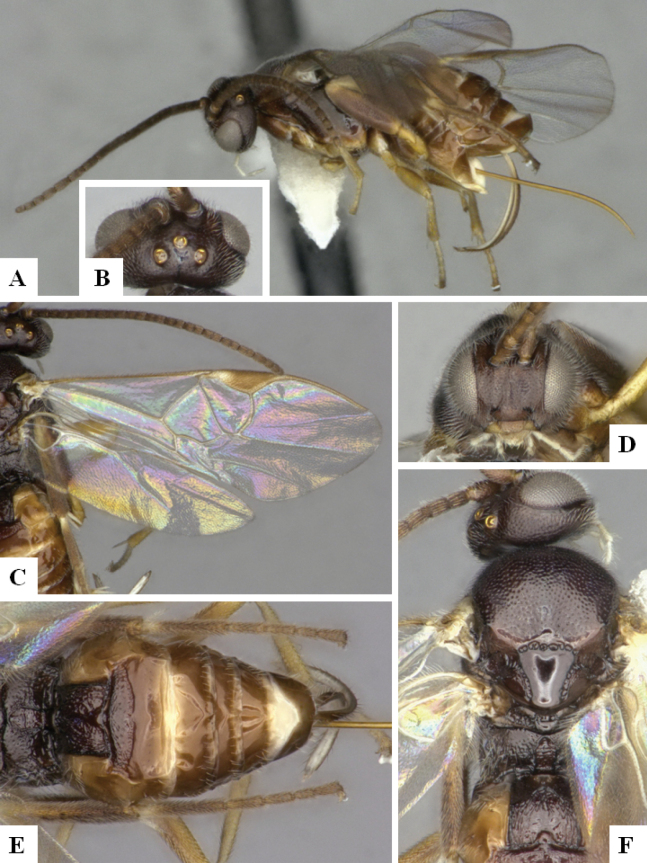
*Dolichogenideaalixhamiltonae* Fernandez-Triana & Boudreault holotype female CNC1179780 **A** habitus, lateral **B** head, dorsal **C** wings **D** head, frontal **E** metasoma, dorsal **F** mesosoma, dorsal.

**Figure 16.**
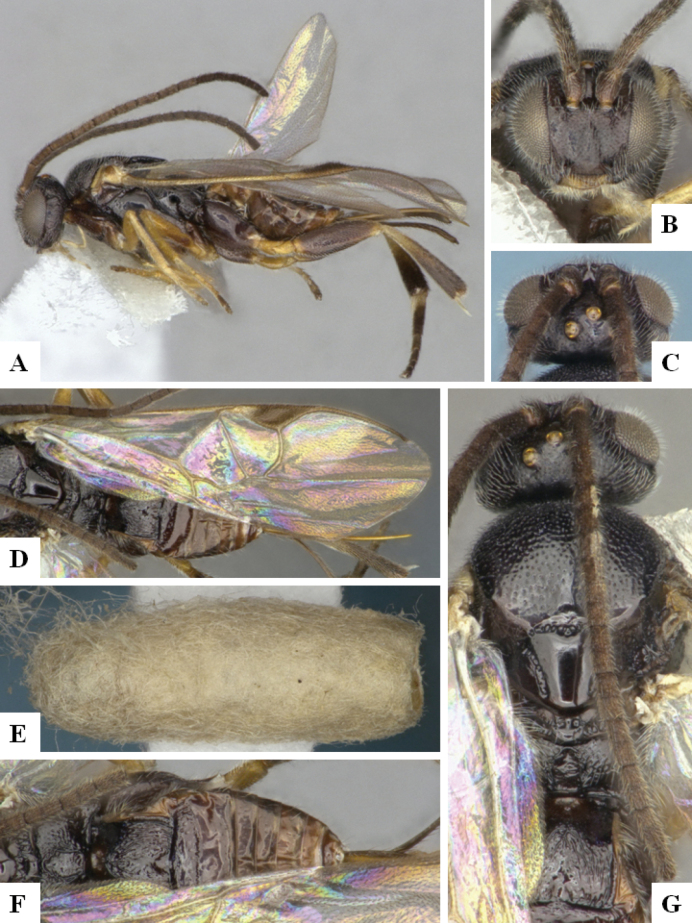
*Dolichogenideaalixhamiltonae* Fernandez-Triana & Boudreault paratype female CNC5342703 **A** habitus, lateral **B** head, frontal **C** head, dorsal **D** wings **E** cocoon **F** metasoma, dorsal **G** mesosoma, dorsal.

##### Other material.

Costa Rica • 1 Female, CNC; DHJPAR0004274.

##### Diagnostic description.

Overall body comparatively mostly smooth and shiny; T1 comparatively broad, covering most of dorsal surface of tergum, its median length ~ 1.2× its width at posterior margin; T1 mostly smooth and shiny (sculpture limited along lateral margins on posterior 0.5); T2 mostly smooth (sculpture limited to margins); ovipositor sheath ~ 1.2× metatibia length; body color comparatively paler colored, mostly yellow-brown or reddish brown, including pale brown antenna; tegula and trochantelli yellow, humeral complex partially yellow and partially brown; pterostigma brown with pale spot at base; all coxae brown; metafemur mostly brown; body length: 2.48–2.58 mm; fore wing length: 2.60–2.75 mm. Among species with T2 mostly smooth and metafemur dark, this species can be distinguished by T1 shape, ovipositor sheath length, and tegula, humeral complex and trochantelli color.

##### Distribution.

Costa Rica.

##### Biology.

Solitary. Thyrididae, *Banisia* myrsusalisDHJ01.

##### DNA barcoding data.

BINBOLD:AAL2285 (1 sequence, barcode compliant).

##### Etymology.

Named in honor of Ms. Alix Hamilton in recognition of her recent and ongoing support for the financial and psychological well-being of Area de Conservación Guanacaste (ACG) and its NGO Guanacaste Dry Forest Conservation Fund (GDFCF) for the GDFCF BioAlfa initiative.

#### ﻿ Dolichogenidea amazonas

Taxon classificationAnimaliaHymenopteraBraconidae

Fernandez-Triana & Boudreault sp. nov.

C4C4FD15-E031-5424-9BBD-8CFEC2B2B9E4

https://zoobank.org/2F485AEB-3EE2-40EE-A5D6-C65858CEBF51

Fig. 17A–F

##### Type material.

***Holotype*.** Peru • Female, CNC; Amazonas; 6°53'S, 77°40'W; 2,000 m; 12.ii.1973; J. Helava leg.; Voucher code: CNC1196966. ***Paratypes*.** Peru • 4 Females, 3 Males, CNC; CNC1180037, CNC1180087, CNC1180095, CNC1180105, CNC1196543, CNC1196548, CNC1196885.

**Figure 17.**
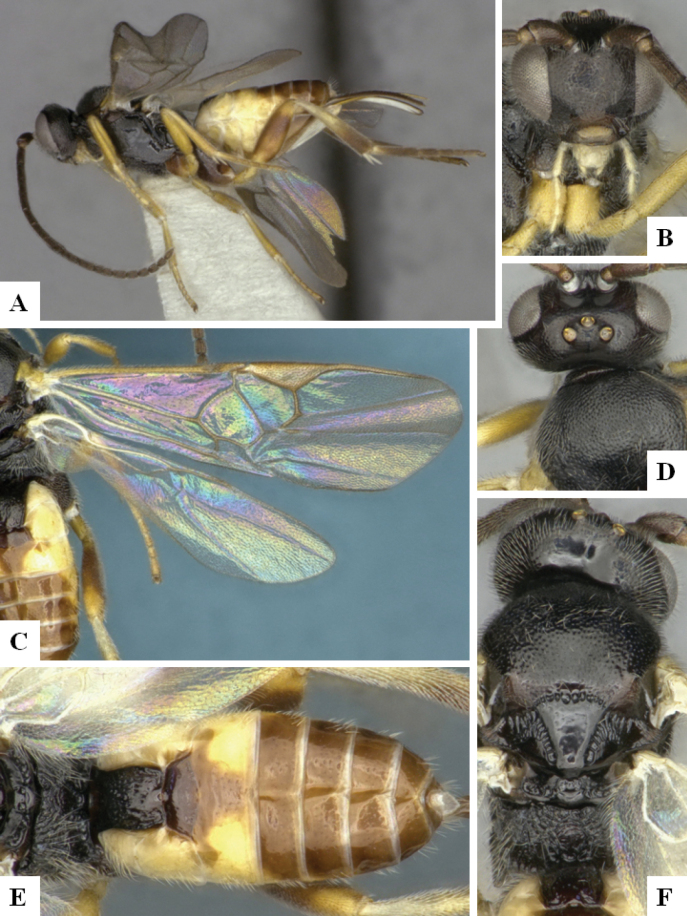
*Dolichogenideaamazonas* Fernandez-Triana & Boudreault holotype female CNC1196966 **A** habitus, lateral **B** head, frontal **C** wings **D** head, dorsal **E** metasoma, dorsal **F** mesosoma, dorsal.

##### Diagnostic description.

Fore wing veins r and 2RS strongly angulate; T1 mostly strongly sculptured, T2 entirely smooth; T1 mostly parallel-sided but slightly widening centrally, its central length > 2.0× its width at posterior margin; T2 comparatively narrow and sub-quadrate, its width at posterior margin 2.0× its central length; pterostigma mostly yellow-white with thin brown margins; first and second pair of legs almost entirely yellow (except for pale brown mesocoxa), third pair of legs mostly brown to dark brown (except for yellow trochanter, trochantellus, anterior 0.1 and posterior 0.1 of metafemur, and anterior 0.5–0.6 of metatibia); T3 mostly yellow (only pale brown on central part); body length: 2.50–3.16 mm; fore wing length: 2.69–3.13 mm. Among all species with T1 strongly sculptured but T2 smooth, this species is clearly distinguishable based on its metasoma coloration, especially its T3 mostly bright yellow with only central area brown; other diagnostic characters include the sub-quadrate shape of T2 and strongly angulate veins r and 2RS in fore wing.

##### Distribution.

Peru.

##### Biology.

No host data available.

##### DNA barcoding data.

No data.

##### Etymology.

Named after the Department of Amazonas, Peru, where all specimens were collected, in recognition of the extraordinary biodiversity of the region.

##### Notes.

All paratypes were collected in the same place and same date than the holotype, which could indicate that this is a gregarious species. The coordinates of the collecting locality (as given in the labels of all eight specimens) are probably inaccurate, as they fall outside of the Amazonas Department, in the neighboring San Martin Department; also the elevation of the place indicated by such coordinates is much higher (~ 3,400 m) than the elevation mentioned in the labels (2,000 m), and there are no roads or any access to that point. Instead, we suspect that the actual collecting locality must have been some 20 km northwest of the coordinates indicated in the labels, in the vicinity of the town of Leimebamba, where there is a paved road, several attractions and lodging facilities, and the elevation of the area is ~ 2,000 m.

#### ﻿ Dolichogenidea anacamposae

Taxon classificationAnimaliaHymenopteraBraconidae

Fernandez-Triana & Boudreault sp. nov.

E01553E2-0D5D-536D-B8CB-97B5C4220897

https://zoobank.org/7BBD568D-7E58-4925-B722-525AD0A9B658

Figs 18A–F , 153A

##### Type material.

***Holotype*.** Costa Rica • Female, CNC; Guanacaste, Area de Conservación Guanacaste, Sector San Cristobal, Sendero Perdido; 10.87940, -85.38607; 620 m; 19.iii.2013; Elda Araya leg.; Host: *Olethreutes* Brown22; Voucher code: DHJPAR0052277; Host voucher code: 13-SRNP-1343. ***Paratypes*.** Costa Rica • 5 Females, CNC; DHJPAR0049429, DHJPAR0052276, DHJPAR0049423, DHJPAR0049308, DHJPAR0049265.

**Figure 18.**
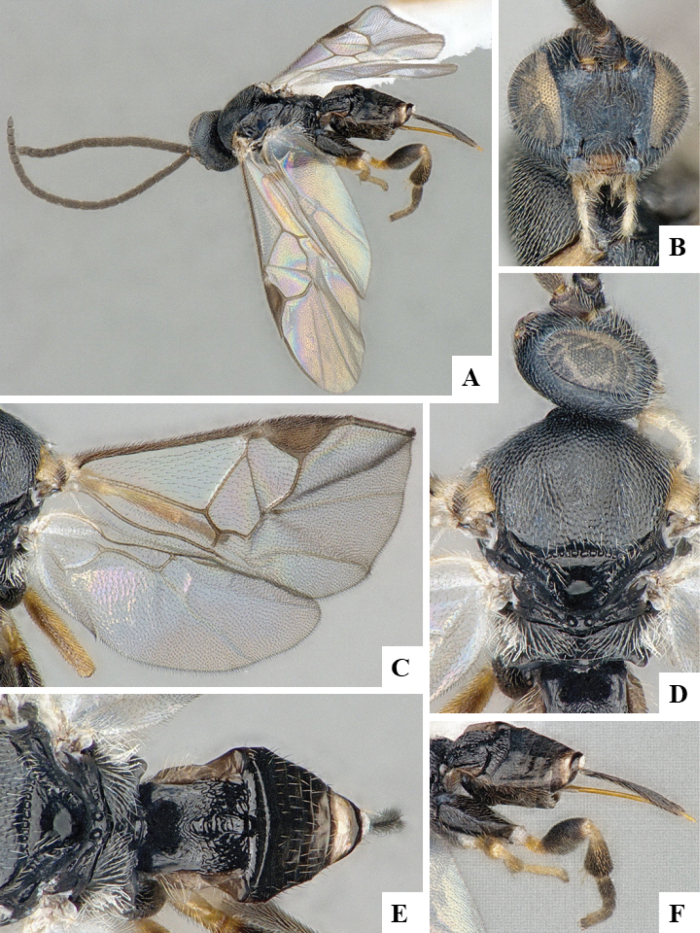
*Dolichogenideaanacamposae* Fernandez-Triana & Boudreault holotype female DHJPAR0052277 **A** habitus, lateral **B** head, frontal **C** wings. **D** mesosoma, dorsal **E** metasoma, dorsal **F** ovipositor.

##### Diagnostic description.

Ocelli comparatively smaller, ocular ocellar line > 3.0× diameter of posterior ocellus; anteromesoscutum mostly with rather coarse punctures; propodeum with almost complete areola (open anteriorly); T1 1.3× as long as wide at posterior margin; T2 transverse, its width at posterior margin > 3.5× its central length; T1 with strong sculpture on posterior 0.5; T2 mostly sculptured but with some smooth areas along anterior margin; ovipositor sheath as long as metatibia length; tegula yellow, humeral complex mostly brown; pterostigma mostly dark brown (with pale spot on anterior 0.1 or less); all coxae, mesofemur and most of metafemur (except for anterior 0.2 which is yellow) brown to dark brown; body length: 2.35–2.81 mm; fore wing length: 2.65–2.97 mm. While *D.anacamposae* has T2 almost entirely sculptured (in that sense it would appear to run through the first half of couplet 9), there are smooth areas along anterior margin that are different from other species with sculptured T2. Additionally, this species can be distinguished by the color of its tegula, humeral complex and legs, anteromesoscutum sculpture, propodeum areola, shape and sculpture of T1 and T2, and length of ovipositor sheath.

##### Distribution.

Costa Rica.

##### Biology.

Solitary. Tortricidae: *Olethreutes* Brown22, *Olethreutes* Janzen323.

##### DNA barcoding data.

BINBOLD:AAE8612 (10 sequences, 10 barcode compliant).

##### Etymology.

Named in honor of Sra. Ana Campos of Liberia, the GDFCF lawyer for land purchases and other local legal affairs, in recognition of her decades of enthusiastic service to the ACG forest restoration effort.

#### ﻿ Dolichogenidea andreamezae

Taxon classificationAnimaliaHymenopteraBraconidae

Fernandez-Triana & Boudreault sp. nov.

38F6CDDB-D43A-5F04-9ACA-0749680D3904

https://zoobank.org/438717F4-2A82-427D-8798-7B3E216AAEA8

Fig. 19A–F

##### Type material.

***Holotype*.** Costa Rica • Female, CNC; Alajuela, Area de Conservación Guanacaste, Sector Rincon Rain Forest, Puente Rio Negro; 10.9038, -85.6027; 340 m; 29.iii.2011; Pablo Umana leg.; Host: *Rivula* Poole03; Voucher code: DHJPAR0043054; Host voucher code: 11-SRNP-41476.

**Figure 19.**
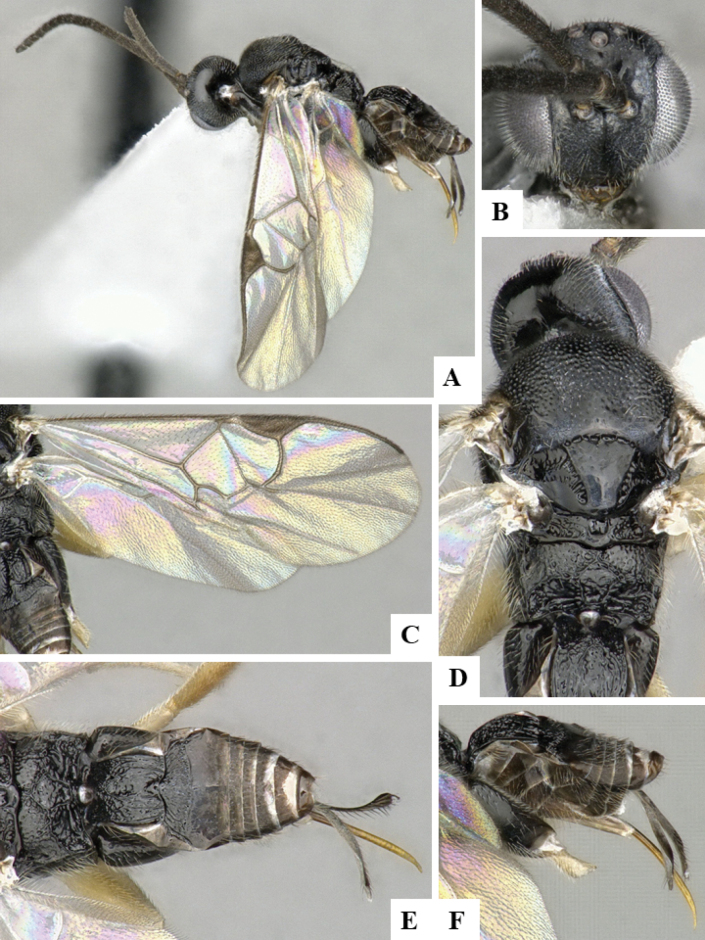
*Dolichogenideaandreamezae* Fernandez-Triana & Boudreault holotype female DHJPAR0043054 **A** habitus, lateral **B** head, frontal **C** wings **D** mesosoma, dorsal **E** metasoma, dorsal **F** ovipositor.

##### Diagnostic description.

Scutellar disc smooth; anterior half of mesopleuron smooth, anteromesoscutum with sparse and relatively shallow punctures; propodeum areola comparatively broad (its height ~ 1.2× its central width) and open anteriorly; T1 and T2 heavily sculptured with strong longitudinal striae; T1 comparatively thin and mostly parallel-sided; T2, comparatively less transverse, its width at posterior margin 3.0× its central length; ovipositor comparatively thicker, at least as thick as 0.8× flagellomeres width; tegula white-yellow, humeral complex mostly brown; pterostigma mostly pale brown with small, paler spot anteriorly; metacoxa entirely dark brown; body length: 2.31 mm; fore wing length: 2.53 mm; BINBOLD:ABA7252, which is 3.71% different from the nearest BIN in BOLD as of March 2022. *Dolichogenideaandreamezae* runs up to couplet 38 in the key above, where it cannot be separate from *D.pedroleoni* because the only known specimen of *D.andreamezae* lacks both hind legs (color and length of metatibia being important to differentiate the species). However, it can be recognized on the basis of smaller size (body length and fore wing length), distinctive DNA barcode, host family (Erebidae) and the wasp making solitary cocoons.

##### Distribution.

Costa Rica.

##### Biology.

Solitary. Reared from a single species of Erebidae, *Rivula* Poole03.

##### DNA barcoding data.

BINBOLD:ABA7252 (2 sequences, 2 barcode compliant).

##### Etymology.

Named in honor of Sra. Andrea Meza of Costa Rica for her strong support of the non-damaging biodevelopment of Costa Rica’s wild biodiversity, during her term as Ministra of Costa Rica’s Ministerio del Ambiente y Energia (MINAE) and before.

#### ﻿ Dolichogenidea angelagonzalezae

Taxon classificationAnimaliaHymenopteraBraconidae

Fernandez-Triana & Boudreault, 2019

23DEA9F8-C88C-5A31-ACE5-3F3C5859547C

Figs 20A–F , 153B

##### Notes.

Full details for this species in Fernandez-Triana et al. (2019). See also the key and Table 1 above.

**Figure 20.**
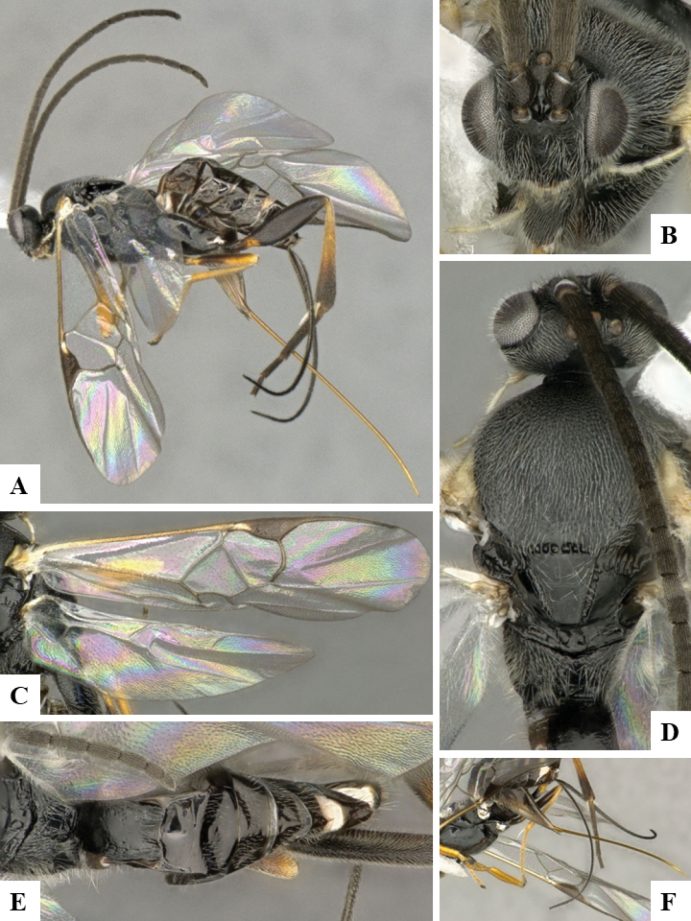
*Dolichogenideaangelagonzalezae* Fernandez-Triana & Boudreault holotype female DHJPAR0020711 **A** habitus, lateral **B** head, frontal **C** wings **D** mesosoma, dorsal **E** metasoma, dorsal **F** ovipositor.

#### ﻿ Dolichogenidea angelsolisi

Taxon classificationAnimaliaHymenopteraBraconidae

Fernandez-Triana & Boudreault sp. nov.

934AC144-45A7-535C-AD2C-6E0D045D3F62

https://zoobank.org/F1C04CF7-F973-4FDB-9FCD-91198F3741A7

Figs 21A–G , 154A

##### Type material.

***Holotype*.** Costa Rica • Female, CNC; Guanacaste, Area de Conservación Guanacaste, Sector Pitilla, Quebradona; 10.99102, -85.39539; 475 m; 13.v.2013; Ricardo Calero leg.; Host: immidJanzen01 Janzen26; Voucher code: DHJPAR0052324; Host voucher code: 13-SRNP-70810. ***Paratypes*.** Costa Rica • 2 Males, CNC; DHJPAR0052333, DHJPAR0053056.

**Figure 21.**
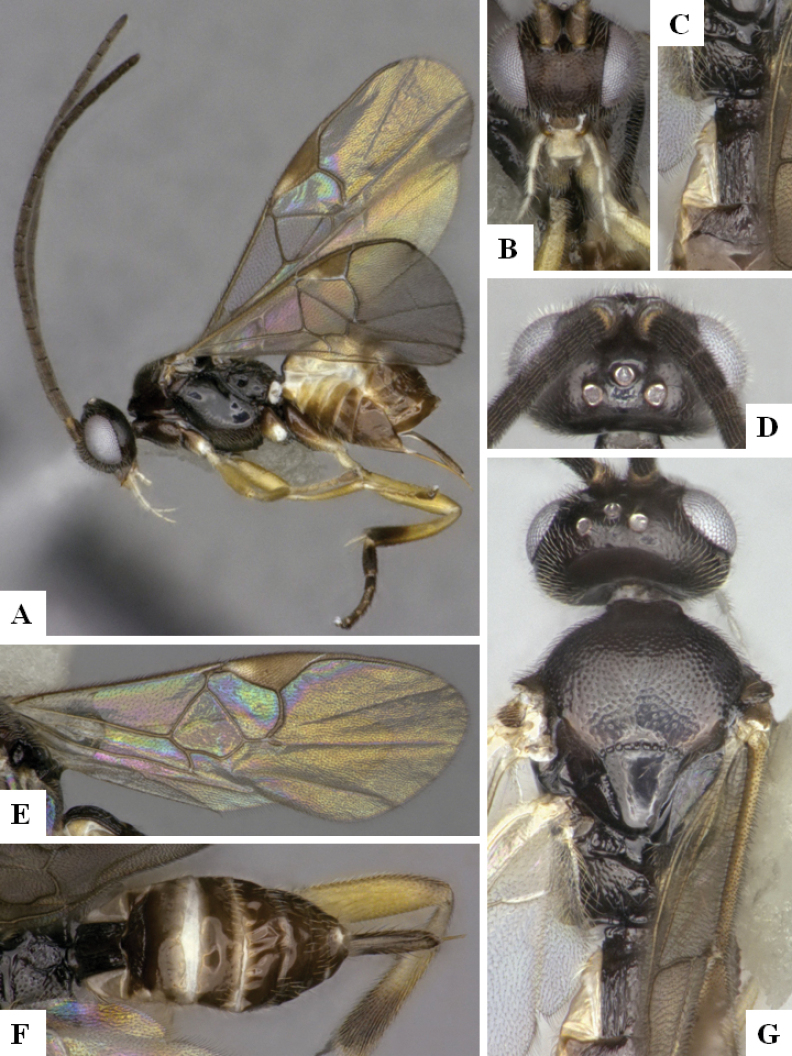
*Dolichogenideaangelsolisi* Fernandez-Triana & Boudreault holotype female DHJPAR0052324 **A** habitus, lateral **B** head, frontal **C** propodeum & T1–T3, dorsal **D** head, dorsal **E** wings **F** metasoma, dorsal **G** mesosoma, dorsal.

##### Diagnostic description.

T1 length medially ~ 3.0× its width at posterior margin; T2 more or less trapezoidal in shape; T2 sculptured ~ margins, centrally smooth; hypopygium with single, small pleat; ovipositor sheath < 0.5× metatibia length; all coxae brown to dark brown; metafemur entirely to mostly yellow (at most with darker spot on posterior 0.3 or less); body length: 2.28 mm; fore wing length: 2.56 mm. Among all species with dark coxae and T2 at least partially smooth, *D.angelsolisi* can be distinguished by its almost unpleated hypopygium and short ovipositor sheath. Another species, *D.bernardoespinozai*, is very similar and we could not find any morphological characters to reliably separate them. However, they can be diagnosed by strong differences in DNA barcodes (almost 10% base pairs difference between the two species) as well as the fact that they have been found at different elevations and ecosystems (see more details provided under *D.bernardoespinozai*).

##### Distribution.

Costa Rica.

##### Biology.

Solitary. Immidae: indetermined species with interim name immidJanzen01 Janzen26.

##### DNA barcoding data.

BINBOLD: BOLD:ACI3413 (3 sequences, 3 barcode compliant).

##### Etymology.

Named in honor of Sr. Angel Solis of Costa Rica, and the Costa Rican National Museum, BioAlfa and the former INBio (Instituto Nacional de Biodiversity) in recognition of his four+ decades dedicated to the biodiversity understanding of the Coleoptera of Costa Rica.

#### ﻿ Dolichogenidea anikenpalolae

Taxon classificationAnimaliaHymenopteraBraconidae

Fernandez-Triana & Boudreault sp. nov.

0B009C23-4B0B-5D69-8436-016F2BD866FD

https://zoobank.org/B3CB983A-660C-4425-AE1E-B3F951062459

Figs 22A–F , 154B

##### Type material.

***Holotype*.** Costa Rica • Female, CNC; Alajuela, Area de Conservación Guanacaste, Sector Rincon Rain Forest, Sendero Albergue Crater; 10.8489, -85.3281; 980 m; 31.v.2010; Carolina Cano leg.; Host: spiloBioLep01 BioLep379; Voucher code: DHJPAR0040482; Host voucher code: 10-SRNP-2764. ***Paratypes*.** Costa Rica • 2 Females, 1 Male, CNC; DHJPAR0049280, DHJPAR0049330, DHJPAR0049340.

**Figure 22.**
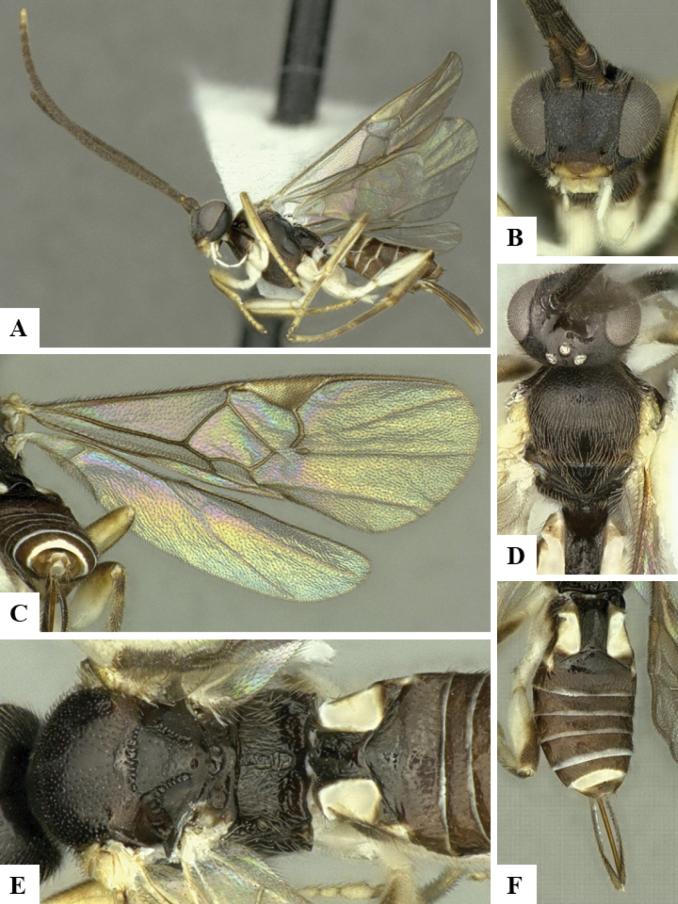
*Dolichogenideaanikenpalolae* Fernandez-Triana & Boudreault holotype female DHJPAR0040482 **A** habitus, lateral **B** head, frontal **C** wings **D** mesoscutum, dorsal **E** mesosoma, dorsal **F** metasoma, dorsal.

##### Diagnostic description.

T1 and T2 smooth; apical 0.5 of T1 narrowing towards posterior margin, so that T1 length is ~ 3.0× its width at posterior margin; pterostigma mostly white-yellow on anterior 0.5–0.7, with darker (pale brown) margins; pro- and mesocoxae yellow-white, metacoxa mostly yellow-white with brown spot on anterior 0.1–0.2; metafemur and metatibia mostly yellow-white but with brown to dark brown margin dorsally (dark dorsal margin only partially defined on metafemur); body length: 2.97–3.22 mm; fore wing length: 3.06–3.47 mm. The color of pterostigma, metafemur and metatibia, as well as shape of T1 distinguish this species among all with smooth T2 and pale pro- and mesocoxae.

##### Distribution.

Costa Rica.

##### Biology.

Solitary. Crambidae: Spilomeninae, spiloBioLep01 BioLep379

##### DNA barcoding data.

BINBOLD:ABY1812 (5 sequences, 5 barcode compliant).

##### Etymology.

Named in honor of Ms. Aniken Palola in recognition of her decade-plus of weathering the demands of being a major part of the Palola family with Mr. Eric Palola, as the two-country Executive Director of the NGO Guanacaste Dry Forest Conservation Fund and its integration with the Costa Rican government’s Area de Conservación Guanacaste (ACG) in northwestern Costa Rica.

#### ﻿ Dolichogenidea anniapicadoae

Taxon classificationAnimaliaHymenopteraBraconidae

Fernandez-Triana & Boudreault sp. nov.

483BAF9D-4D25-50F2-AB9C-9E98EF171E84

https://zoobank.org/B49E79EF-FF05-4F26-A19A-13B892DAB86E

Figs 23A–F , 155A

##### Type material.

***Holotype*.** Costa Rica • Female, CNC; Guanacaste, Area de Conservación Guanacaste, Sector Cacao, Sendero Cima; 10.93328, -85.45729; 1,460 m; 22.v.2000; D. H. Janzen & W. Hallwachs leg.; Malaise trap; Voucher code: DHJPAR0012546. ***Paratypes*.** Costa Rica • 4 Females, CNC; DHJPAR0012552, DHJPAR0031440, DHJPAR0034104, DHJPAR0034159.

**Figure 23.**
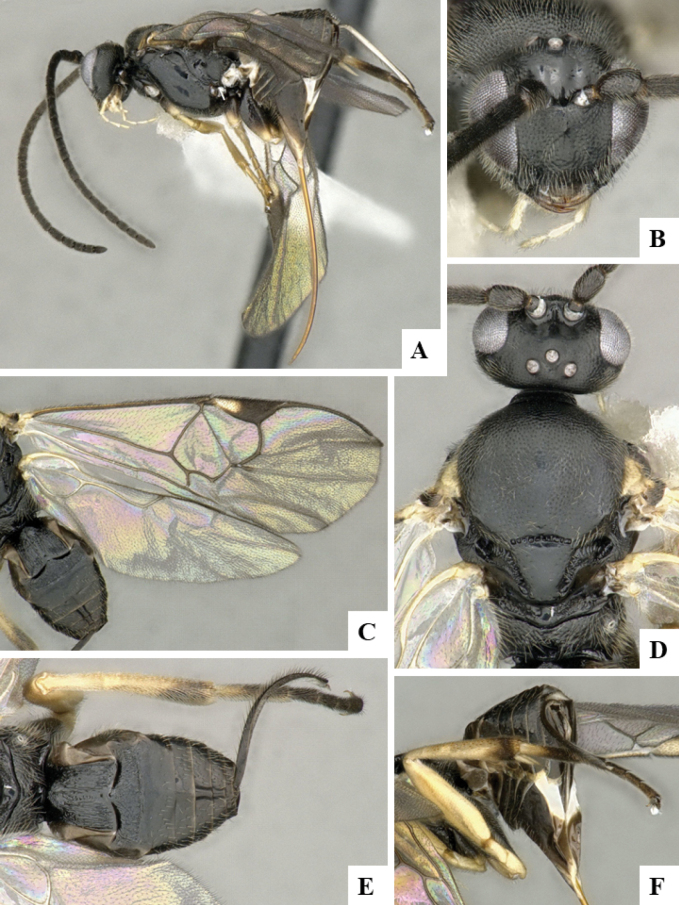
*Dolichogenideaanniapicadoae* Fernandez-Triana & Boudreault holotype female DHJPAR0012546 **A** habitus, lateral **B** head, frontal **C** wings **D** head & Mesosoma, dorsal **E** metasoma, dorsal **F** metasoma, postero-lateral.

##### Diagnostic description.

Posterior 0.5–0.6 of T1 and T2 mostly with strong sculpture, usually longitudinal striae covering entire surface (but T2 with small polished area centrally); T1 parallel-sided to slightly broadening posteriorly; T2 comparatively very transverse but with anterior and posterior margins strongly arcuate, so that T2 length is longer medially than laterally and thus T2 width at posterior margin is usually < 3.0× its length medially; ovipositor ~ 2.0× as metatibia length; pterostigma with relatively large pale (yellow-white) spot at base that occupies 0.3–0.4 pterostigma length. This species has strong sculpture (usually longitudinal striae) covering posterior 0.5–0.6 of T1 and most of T2. However, unlike the majority of species with similarly strong sculpture, T2 has a central area which is smooth and also T2 is very transverse and with anterior and posterior margins strongly arcuate; body length: 2.96–3.16 mm; fore wing length: 3.56–3.76 mm. Because of that unique shape and sculpture pattern of T2, as well as its metafemur color, it can be separate from all the species with entirely and strongly sculptured T2 which is not transverse, as well as all the species with smooth T2 and/or broad T2. Among similar species, *D.anniapicadoae* can be distinguished from *D.jorgecarvajali* and *D.rexhamiltoni* because of its comparatively much longer ovipositor and pterostigma color.

##### Distribution.

Costa Rica.

##### Biology.

Solitary. Crambidae: *Ategumialotanalis*.

##### DNA barcoding data.

BINBOLD:ABY7999 (9 sequences, 9 barcode compliant).

##### Etymology.

Named in honor of parataxonomist Sra. Annia Picado of Costa Rica, and of BioAlfa and the former INBio (Instituto Nacional de Biodiversity) in recognition of her two+ decades dedicated to specimen preparation for the biodiversity understanding of the Diptera and Lepidoptera of Costa Rica.

#### ﻿ Dolichogenidea annlisterudae

Taxon classificationAnimaliaHymenopteraBraconidae

Fernandez-Triana & Boudreault sp. nov.

B7B1797F-5190-5DC0-AA28-A920662DD24D

https://zoobank.org/B1389056-E9FB-49E3-9CE1-FAF571E11349

**Figure 24.**
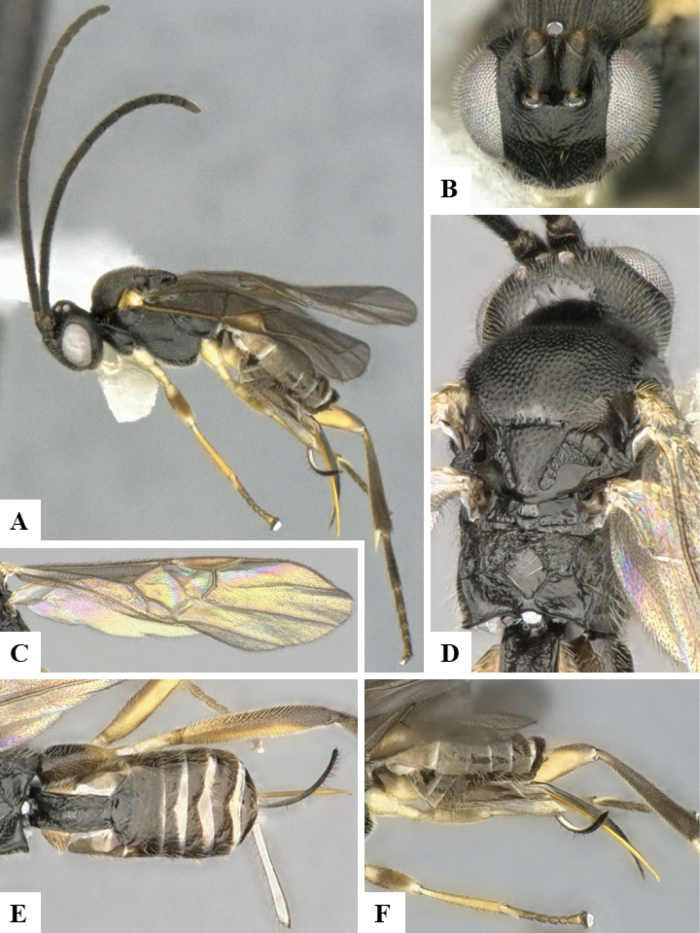
*Dolichogenideaannlisterudae* Fernandez-Triana & Boudreault holotype female DHJPAR0012553 **A** habitus, lateral **B** head, frontal **C** wings **D** mesosoma, dorsal **E** metasoma, dorsal **F** metasoma, lateral.

**Figure 25.**
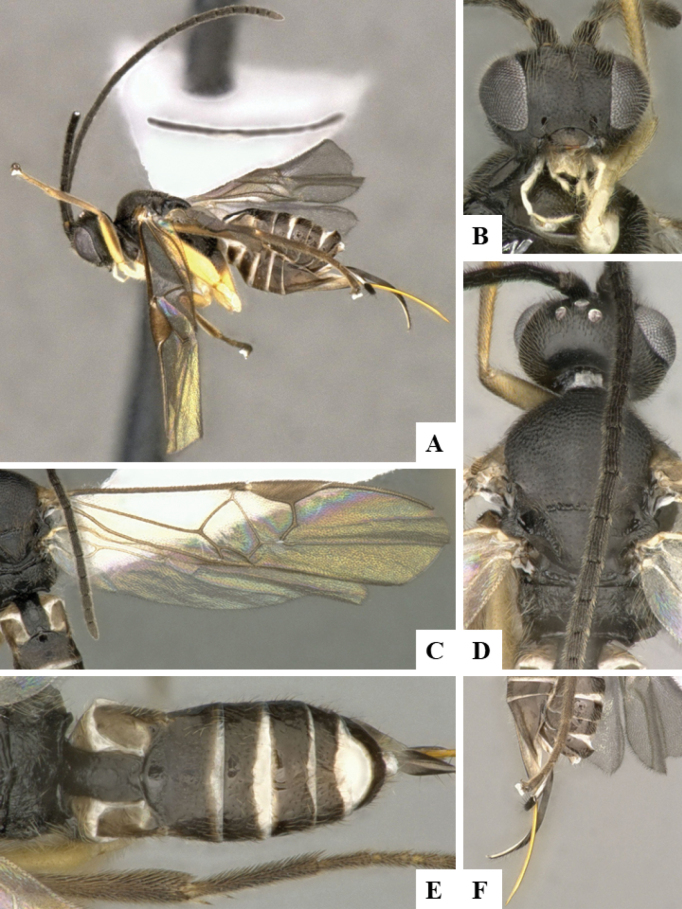
*Dolichogenideaannlisterudae* Fernandez-Triana & Boudreault paratype female DHJPAR0012551 **A** habitus, lateral **B** head, frontal **C** wings **D** mesosoma, dorsal **E** metasoma, dorsal **F** ovipositor, lateral.

**Figure 26.**
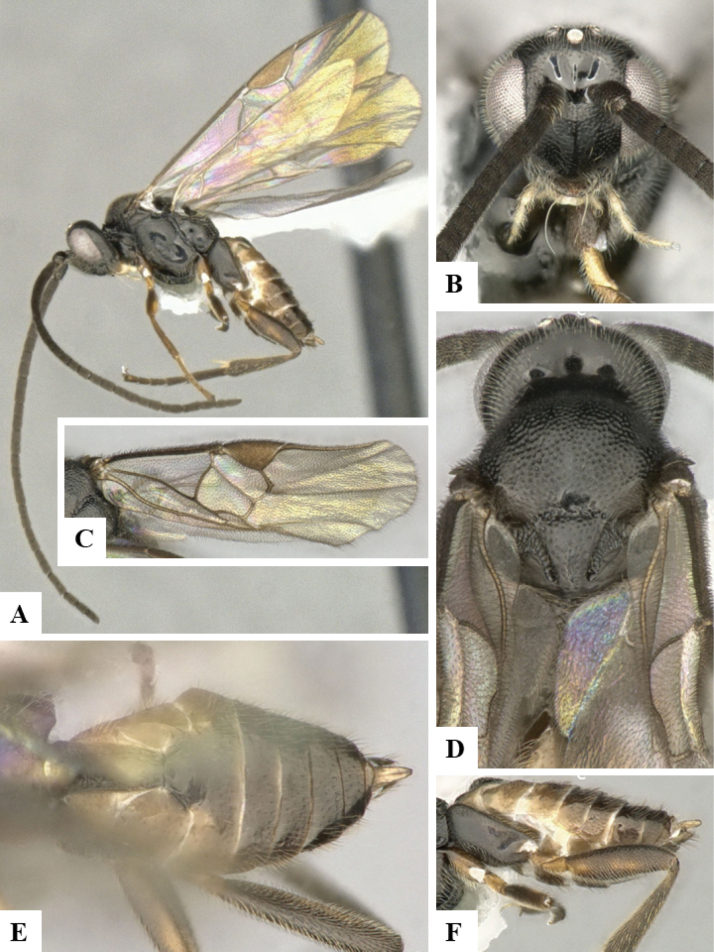
*Dolichogenideaannlisterudae* Fernandez-Triana & Boudreault paratype male DHJPAR0012561 **A** habitus, lateral **B** head, frontal **C** wings **D** mesosoma, dorsal **E** metasoma, dorsal **F** metasoma, lateral.

Figs 24A–F , 25A–F , 26A–F , 155B

##### Type material.

***Holotype*.** Costa Rica • Female, CNC; Guanacaste, Area de Conservación Guanacaste, Sector Cacao, Sendero Cima; 10.93328, -85.45729; 1,460 m; 29.v.2000; D. H. Janzen & W. Hallwachs leg.; Malaise trap; Voucher code: DHJPAR0012553. ***Paratypes*.** Costa Rica • 6 Females, 4 Males, CNC; DHJPAR0012551, DHJPAR0012556, DHJPAR0012561, DHJPAR0012724, DHJPAR0012732, DHJPAR0013471, DHJPAR0031468, DHJPAR0031545, DHJPAR0034157, DHJPAR0033923.

##### Diagnostic description.

T1 length medially ~ 3.0× its width at posterior margin; T2 more or less trapezoidal in shape; T2 mostly smooth but with some sculpture around margins; tegula and humeral complex yellow; pro- and mesocoxae yellow-white or yellow; metacoxa mostly yellow with dark brown spot on anterior 0.3; metafemur mostly yellow (thin brown area dorsally on apical 0.2–0.3); metatibia brown to dark brown; body length 2.35–2.65 mm; fore wing length: 2.65–3.03 mm. Among all species with T2 mostly to entirely smooth and pro- and mesocoxae pale, *D.annlisterudae* can be distinguished by T1 shape, T2 shape and sculpture, and color of tegula, humeral complex and coxae, mesofemur and metatibia.

##### Distribution.

Costa Rica.

##### Biology.

No host data available.

##### DNA barcoding data.

BINs BOLD:AAB5549 (52 sequences, 45 barcode compliant), and BOLD:ACF0272 (16 sequences, 9 barcode compliant).

##### Etymology.

Named after Anne Listerud, neighbor to DHJ and WH in Philadelphia, who has helped the inventory in many ways.

##### Notes.

Specimens of BINBOLD:AAB5549 have stigma centrally whitish, T2 slightly less sculptured (almost entirely smooth), and F15 comparatively shorter (its length ~ 0.5 length of F16); whereas the three specimens of BINBOLD:ACF0272 have entirely brown stigma, T2 slightly more sculptured (only centrally smooth), and F15 comparatively longer (its length ~ 0.7 length of F16). However, those differences are very subtle and only three females of each BIN were available for study. They were collected by Malaise traps in the same locality (1 km apart from each other) and there is no host data for any of them. Because their DNA barcodes are 98.72% similar (1.28% bp different) we here consider them to represent the same species.

#### ﻿ Dolichogenidea annychaverae

Taxon classificationAnimaliaHymenopteraBraconidae

Fernandez-Triana & Boudreault sp. nov.

40571638-579B-5375-BE5E-6A3BA14CA955

https://zoobank.org/F7FE69A7-0A7E-47B1-8F70-36883295EFB9

Fig. 27A–E

##### Type material.

***Holotype*.** Costa Rica • Female, CNC; Guanacaste, Area de Conservación Guanacaste, Sector Santa Rosa, Bosque San Emilio, 10.8439, -85.6138; 300 m; 26.iv.1999; D. H. Janzen & W. Hallwachs leg.; Malaise trap; Voucher code: DHJPAR0013185.

**Figure 27.**
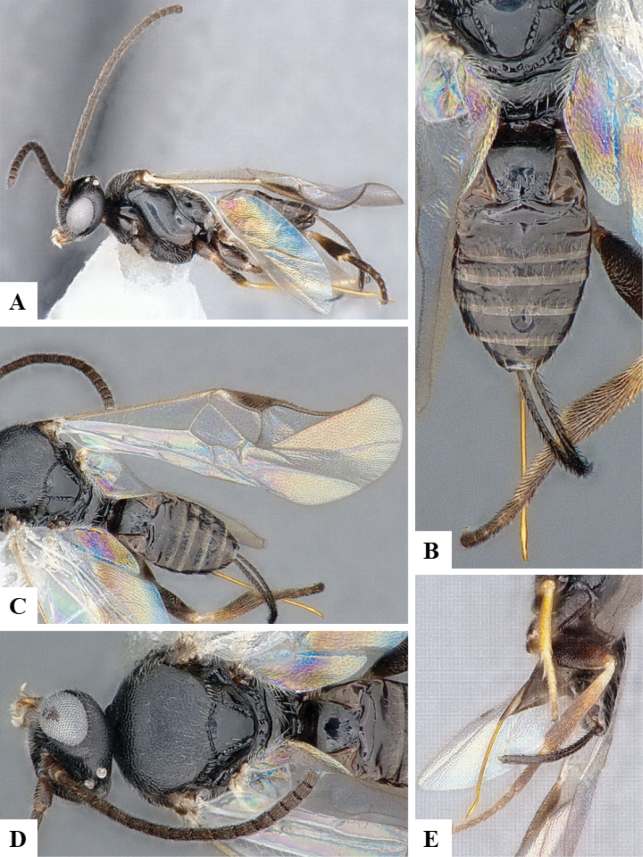
*Dolichogenideaannychaverae* Fernandez-Triana & Boudreault holotype female DHJPAR0013185 **A** habitus, lateral **B** metasoma, dorsal **C** wings **D** mesosoma, dorsal **E** ovipositor, lateral.

##### Diagnostic description.

F15 comparatively sub-cubic, L/W 1.15×; scutellar disc smooth and shiny; propodeum with short carinae weakly defining an areola only on posterior half or less; T1 and T2 smooth; T1 strongly narrowing near posterior margin, T1 length 2.0× its maximum width, and T1 width at anterior margin 2.0× T1 width at posterior margin; ovipositor sheath length 1.15× metatibia length; most legs dark brown to black; body length: 1.80 mm; fore wing length: 2.08 mm. Among all species with dark coxae and smooth T1 and T2, *D.annychaverae* is distinguished by shape of T1, overall body sculpture, small body size and ovipositor sheath length. *D.alanflemingi* is morphologically similar but has F15 comparatively much longer, T1 comparatively thinner and with different shape, ovipositor sheath comparatively shorter (see key for better diagnosis of these two species). They also have different DNA barcodes.

##### Distribution.

Costa Rica.

##### Biology.

No host data available.

##### DNA barcoding data.

BINBOLD:AAD5258 (7 sequences, 7 barcode compliant).

##### Etymology.

Named after Anny Chavera of San Jose, Costa Rica in recognition of her years of biodiversity administration.

#### ﻿ Dolichogenidea antioquia

Taxon classificationAnimaliaHymenopteraBraconidae

Fernandez-Triana & Boudreault sp. nov.

D242DE9F-508A-5F65-A831-18745AD13CDF

https://zoobank.org/D3E43DA3-A1A2-47B0-887F-94EAEC994925

Fig. 28A–F

##### Type material.

***Holotype*.** Colombia • Female, CNC; Antioquia; 7°5'N [here corrected to 7°3'N, see Notes below] 76°30'W; 1,800 m; 15.iv.1973; J. Helava leg.; Voucher code: CNC1179669. ***Paratypes*.** Colombia• 3 Females, 4 Males, CNC; CNC1179732, CNC1179829, CNC1179888, CNC1179923, CNC1179928, CNC1179931, CNC1179939.

**Figure 28.**
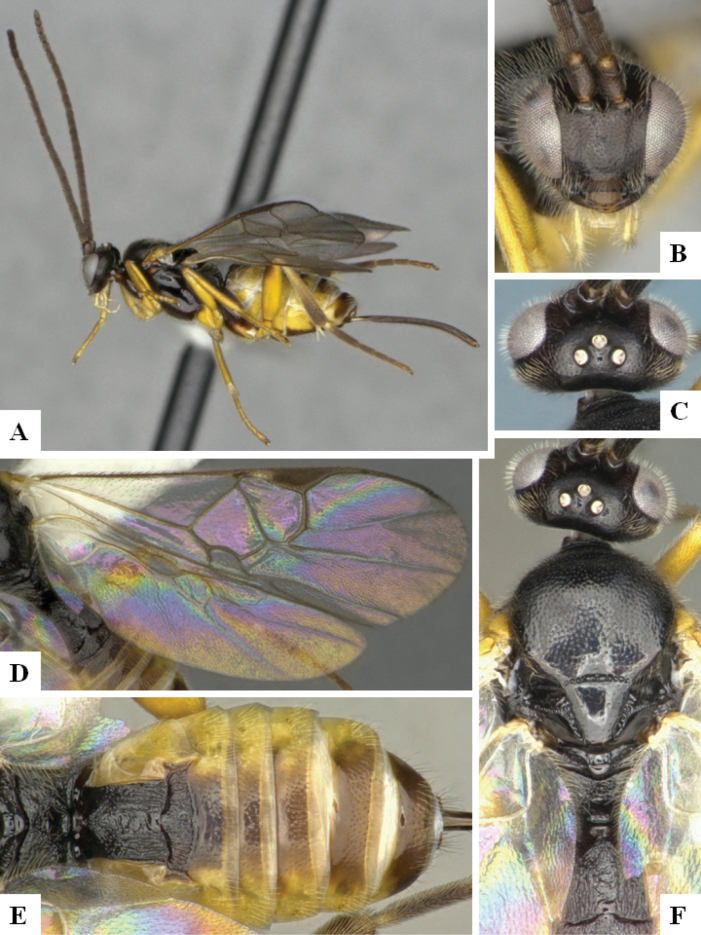
*Dolichogenideaantioquia* Fernandez-Triana & Boudreault holotype female CNC1179669 **A** habitus, lateral **B** head, frontal **C** head, dorsal **D** wings **E** metasoma, dorsal **F** mesosoma, dorsal.

##### Diagnostic description.

F2 length 2.5× F14 length; T1 and T2 heavily sculptured with strong longitudinal striae; T1 comparatively broad, mostly parallel-sided but slightly widening towards posterior margin; T2 more or less transverse, with anterior margin centrally arcuate, so that its width at posterior margin is ~ 3.0× its length medially; pterostigma mostly brown but with pale spot on proximal 0.2; metacoxa almost entirely dark brown (very small yellow spot on posterior 0.1); metatibia dark brown to black on posterior 0.5; metatibial spurs entirely yellow; metasoma mostly pale colored, with T1 black, T2 dark brown to black, T3–T7 mostly yellow but with small pale brown band centrally, all laterotergites yellow, sternites and hypopygium mostly yellow but ventrally with small brown band; body length and fore wing lengths: 3.50–3.70 mm. Among all species with heavily sculptured T1 and T2, this species is distinctive by its T2 shape (especially anterior margin), metatibia posterior 0.5 dark brown, body and fore wing length size, and extensive yellow coloration of metasoma.

##### Distribution.

Colombia.

##### Biology.

No host data available.

##### DNA barcoding data.

No data.

##### Etymology.

Named after the Colombian department where the species was collected.

##### Notes.

When the coordinates written in the labels of the collected specimens are checked in a map, they correspond to ~ 700 m of elevation, much less than the 1,800 m indicated in those same labels. A careful analysis of the surrounding area shows that if the latitude would be slightly modified by ~ 2 minutes (7°3'N instead of the 7°5'N written in the labels), the resulting place would be the road close to “Termales Peque Antioquia”, which is an accessible and commonly visited place in the area and it happens to be at ~ 1,800 m. Although it is impossible to be entirely sure, it is a reasonable assumption to consider that the labels had an error (either a typo when writing the latitude or an error when measuring it), and that the amended latitude by us is the most likely type locality for the species.

#### ﻿ Dolichogenidea antjevirkusae

Taxon classificationAnimaliaHymenopteraBraconidae

Fernandez-Triana & Boudreault sp. nov.

543176A7-B225-5D5A-8DE8-983D302164DE

https://zoobank.org/5762E230-9299-4B4A-9F7F-929D13480DE1

Fig. 29A–F

##### Type material.

***Holotype*.** Costa Rica • Female, CNC; Guanacaste, Area de Conservación Guanacaste, Sector Cacao, Sendero Cima; 10.93328, -85.45729; 1,460 m; 18.xii.2008; D. H. Janzen & W. Hallwachs leg.; Malaise trap; Voucher code: DHJPAR0031522.

**Figure 29.**
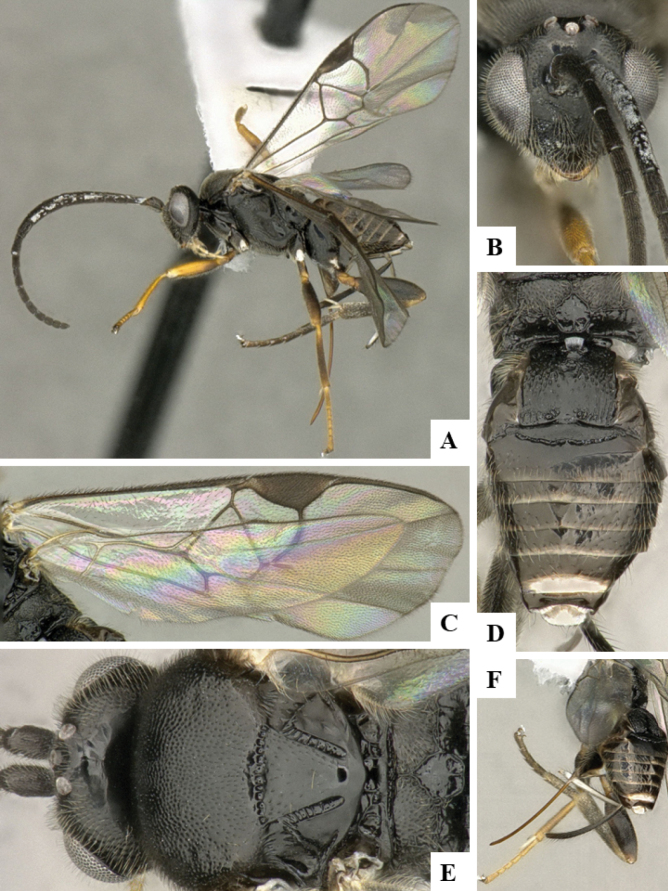
*Dolichogenideaantjevirkusae* Fernandez-Triana & Boudreault holotype female DHJPAR0031522 **A** habitus, lateral **B** head, frontal **C** wings **D** metasoma, dorsal **E** mesosoma, dorsal **F** metasoma, lateral.

##### Diagnostic description.

Posterior 0.5–0.6 of T1 mostly with strong sculpture, usually longitudinal striae; T1 slightly broadening posteriorly; T2 mostly smooth; T2 comparatively very transverse but with anterior margin arcuate; ovipositor sheath clearly longer (1.15–1.25×) than metatibia length; tegula and humeral complex dark brown; coxae dark brown to black; trochantelli mostly yellow-brown; metafemur dark brown; metatibia dark brown on posterior 0.8; body length: 2.96 mm; fore wing length: 3.44 mm. Among species with smooth T2 and metafemur dark, this species can be distinguished by T1 shape, ovipositor sheath length, and tegula, humeral complex and trochantelli color.

##### Distribution.

Costa Rica.

##### Biology.

No host data available.

##### DNA barcoding data.

BINBOLD:AAM5849 (1 sequence, barcode compliant).

##### Etymology.

Named in honor of Mrs. Antje Virkus of Germany in recognition of her recent efforts to support Area de Conservación Guanacaste biodiversity through establishing neighboring plantation reforestation as a business venture that will also be invaded by wild ACG biodiversity.

#### ﻿ Dolichogenidea arenal

Taxon classificationAnimaliaHymenopteraBraconidae

Fernandez-Triana & Boudreault sp. nov.

12DB0A06-6A85-5835-BE9C-D05DEBDF0F8E

https://zoobank.org/3AD3B14A-6BC4-41A6-AAD3-FA89308835F1

Fig. 30A–F

##### Type material.

***Holotype*.** Costa Rica • Female, CNC; Alajuela, Arenal Volcano, northern slope; 550 m; 26–27.ix.1972; J. Helava leg.; Voucher code: CNC1179955.

**Figure 30.**
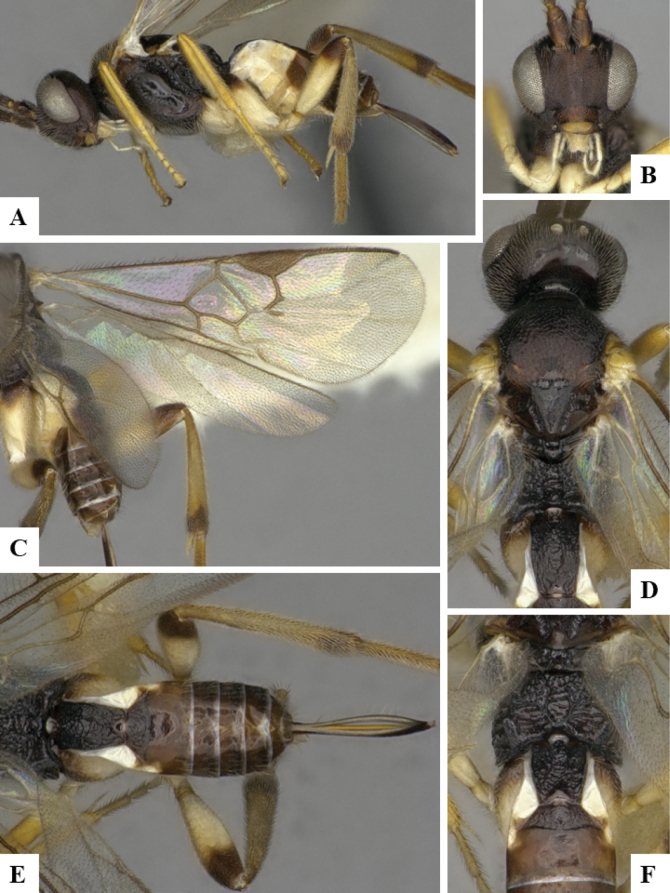
*Dolichogenideaarenal* Fernandez-Triana & Boudreault holotype female CNC1179955 **A** close-up of habitus, lateral **B** head, frontal **C** wings **D** mesosoma, dorsal **E** metasoma, dorsal **F** propodeum & T1–T3, dorsal.

##### Diagnostic description.

Fore wing veins r and 2RS meeting at a strong angle; T1 mostly parallel-sided but narrowing towards posterior margin near its posterior half, its length medially ~ 3.0× its width at posterior margin and its width at anterior margin 1.4× its width at posterior margin; T2 length medially 3.0× its width at posterior margin; ovipositor sheath 0.75× as long as metatibia; pterostigma uniformly colored, mostly brown to pale brown; pro- and mesocoxae yellow-white, metacoxa mostly yellow with small brown spot on anterior 0.2; metafemur and metatibia mostly yellow or yellow-white, with brown spot on posterior 0.3 and 0.1 respectively; body length: 2.63 mm; fore wing length: 2.72 mm. Among species with T2 not strongly sculptured, *D.arenal* is distinctive based on strong angulation of fore wing veins r and 2RS, as well as leg color, shape of T1 and T2, ovipositor sheath lengths, and body size.

##### Distribution.

Costa Rica.

##### Biology.

No host data available.

##### DNA barcoding data.

No data.

##### Etymology.

Named after the type locality, the Arenal Volcan, in Costa Rica.

#### ﻿ Dolichogenidea bernardoespinozai

Taxon classificationAnimaliaHymenopteraBraconidae

Fernandez-Triana & Boudreault sp. nov.

7A6E80DE-EEA5-5A76-ADBD-BD5B24FF4FDF

https://zoobank.org/C988E427-A182-41FE-90FA-6688048CD598

Fig. 31A–F

##### Type material.

***Holotype*.** Costa Rica • Female, CNC; Guanacaste, Area de Conservación Guanacaste, Sector Cacao, Sendero Cima; 10.93328, -85.45729; 1,460 m; 18.xii.2008; D. H. Janzen & W. Hallwachs leg.; Malaise trap; Voucher code: DHJPAR0031544. ***Paratypes*.** Costa Rica • 8 Females, 1 Male, CNC; DHJPAR0046031, DHJPAR0031476, DHJPAR0013459, DHJPAR0031475, DHJPAR0012550, DHJPAR0031451, DHJPAR0031482, DHJPAR0031483, DHJPAR0031519.

**Figure 31.**
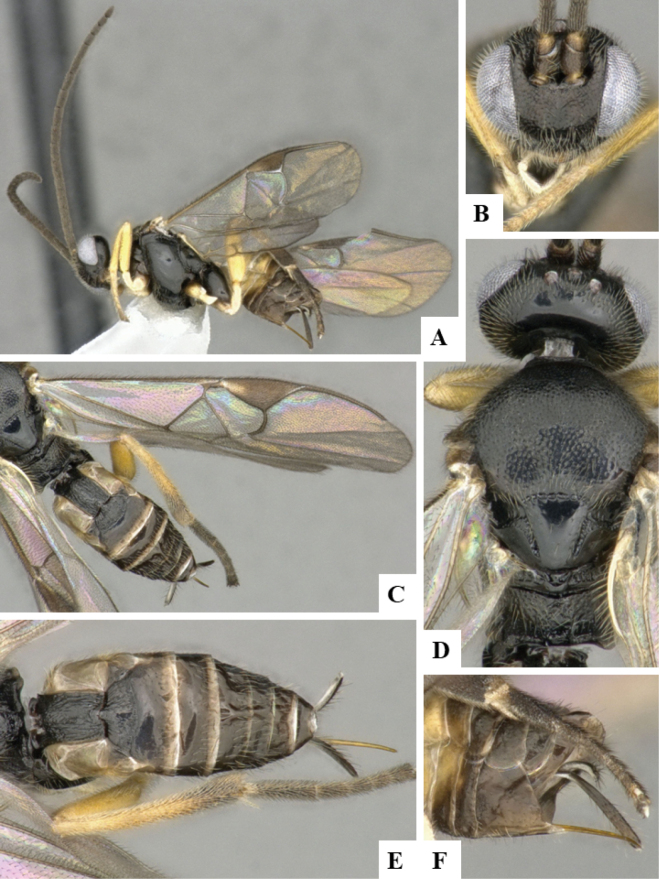
*Dolichogenideabernardoespinozai* Fernandez-Triana & Boudreault paratype female DHJPAR0012550 **A** habitus, lateral **B** head, frontal **C** wings **D** mesosoma, dorsal **E** metasoma, dorsal **F** ovipositor, lateral.

##### Diagnostic description.

T1 mostly sculptured with longitudinal striae; T2 with central smooth area; T2 width at posterior margin < 3.0× its central length; ovipositor sheath 1.3× as long as metatibia length; pterostigma entirely brown; legs mostly brown to dark brown, but metatibia with anterior 0.2 yellow; body length: 2.03–2.41 mm; fore wing length: 2.33–2.84 mm. Among species with T2 not entirely sculptured and dark coxae, *D.bernardoespinozai* can be distinguished by T1 and T2 sculpture, short ovipositor sheath length and pterostigma color. We could not find any reliable morphological character to separate it from *D.angelsolisi*, but the two species can be diagnosed based on strong molecular differences (almost 10% base pairs difference between the two of them). Furthermore, the two species have been found at different elevations and ecosystems. *D.bernardoespinozai* has been collected in rainforest and cloud forests, at higher elevations between 1,220–1,460 m in ACG and as up to 3,000 m elsewhere in Costa Rica (Parque Nacional Los Quetzales, San José Province), with only a single specimen collected at 815 m (ACG); whereas *D.angelsolisi* has only been collected at a much lower elevation, 475 m in ACG, corresponding to dry forest.

##### Distribution.

Costa Rica.

##### Biology.

No host data available.

##### DNA barcoding data.

BINBOLD:AAE8596 (17 sequences, 16 barcode compliant).

##### Etymology.

Named in honor of Sr. Bernardo Espinoza of San Jose, and the Costa Rican National Museum, BioAlfa and the former INBio (Instituto Nacional de Biodiversity) in recognition of his more than two decades dedicated to the taxonomic biodiversity understanding of the Arctiinae (Erebidae) Lepidoptera of Costa Rica.

#### ﻿ Dolichogenidea beryllacosteae

Taxon classificationAnimaliaHymenopteraBraconidae

Fernandez-Triana & Boudreault sp. nov.

B35E489C-2F2C-5388-8909-8B2822FB8CC0

https://zoobank.org/6B17A173-955C-44CF-8A1A-36BDAECB1203

Fig. 32A–F

##### Type material.

***Holotype*.** Costa Rica • Female, CNC; Guanacaste, Area de Conservación Guanacaste, Sector Rincon Rain Forest, Estacion Llanura; 10.93332, -85.25331; 135 m; 15.vi.2012; Cirilo Umana leg.; Host: *Microscahedialis*; Voucher code: DHJPAR0049843; Host voucher code: 12-SRNP-75991.

**Figure 32.**
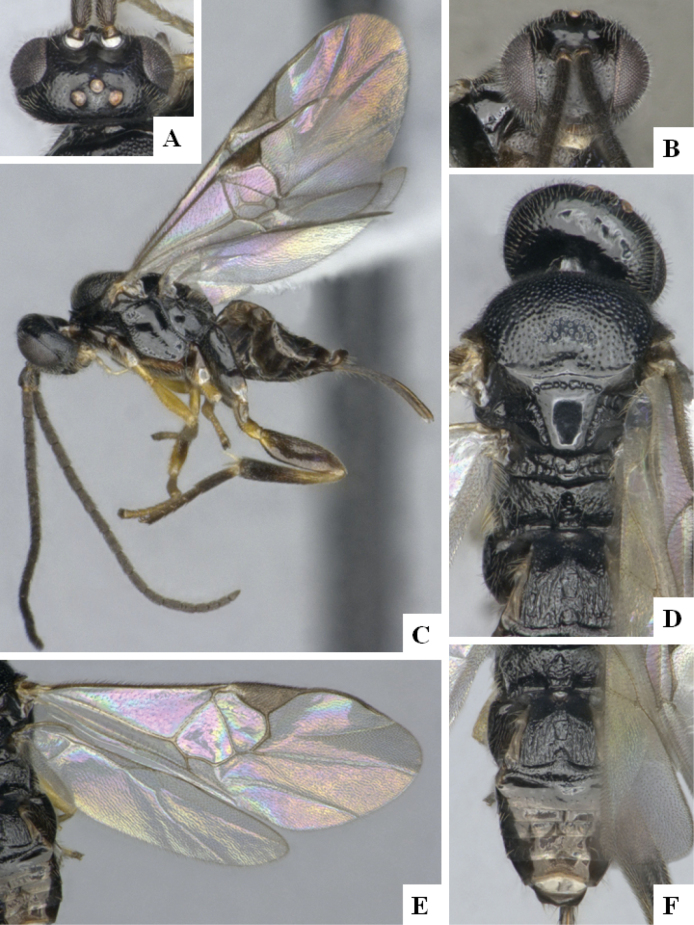
*Dolichogenideaberyllacosteae* Fernandez-Triana & Boudreault holotype female DHJPAR0049843 **A** head, dorsal **B** head, frontal **C** habitus, lateral **D** mesosoma, dorsal **E** wings **F** metasoma, dorsal.

##### Diagnostic description.

F15 clearly rectangular, its length 1.6× its width; posterior 0.5–0.6 of T1 mostly with strong sculpture, usually longitudinal striae; T1 slightly broadening posteriorly; T2 mostly smooth, except along posterior margin; T2 comparatively very transverse but with anterior margin arcuate; ovipositor sheath slightly shorter than metatibia length; tegula dark brown; mesosternum with stripe pale brown, contrasting with rest of black mesosternum; pro- and mesocoxae brown, metacoxa dark brown to black; metafemur dark brown; metatibia brown on posterior 0.6; body length: 2.30 mm; fore wing length: 2.66 mm. Among species with smooth T2 and metafemur dark, this species can be distinguished by F15 length, T1 shape, ovipositor sheath length, and tegula and mesosternum color.

##### Distribution.

Costa Rica.

##### Biology.

Gregarious. Thyrididae: *Microscahedialis*.

##### DNA barcoding data.

BINBOLD:AAM1098 (3 sequences, 3 barcode compliant).

##### Etymology.

Named in honor of Mrs. Beryl Lacoste of France, Florida, USA and Guanacaste Province Costa Rica in recognition of her recent and ongoing support for the financial and psychological well-being of Area de Conservación Guanacaste (ACG) and its NGO Guanacaste Dry Forest Conservation Fund (GDFCF) for the GDFCF BioAlfa initiative.

#### ﻿ Dolichogenidea bradzlotnicki

Taxon classificationAnimaliaHymenopteraBraconidae

Fernandez-Triana & Boudreault sp. nov.

6D5B724F-468B-5BC8-A5E8-21E519D90224

https://zoobank.org/25D2B856-AD81-4F2B-A83F-542C347224B0

Fig. 33A–F

##### Type material.

***Holotype*.** Costa Rica • Female, CNC; Guanacaste, Area de Conservación Guanacaste, Sector San Cristobal, Bosque Transición; 10.86472, -85.41531; 540 m; 05.vii.2012; Gloria Sihezar leg.; Host: *Chlamydastisvividella*; Voucher code: DHJPAR0049894; Host voucher code: 12-SRNP-2858.

**Figure 33.**
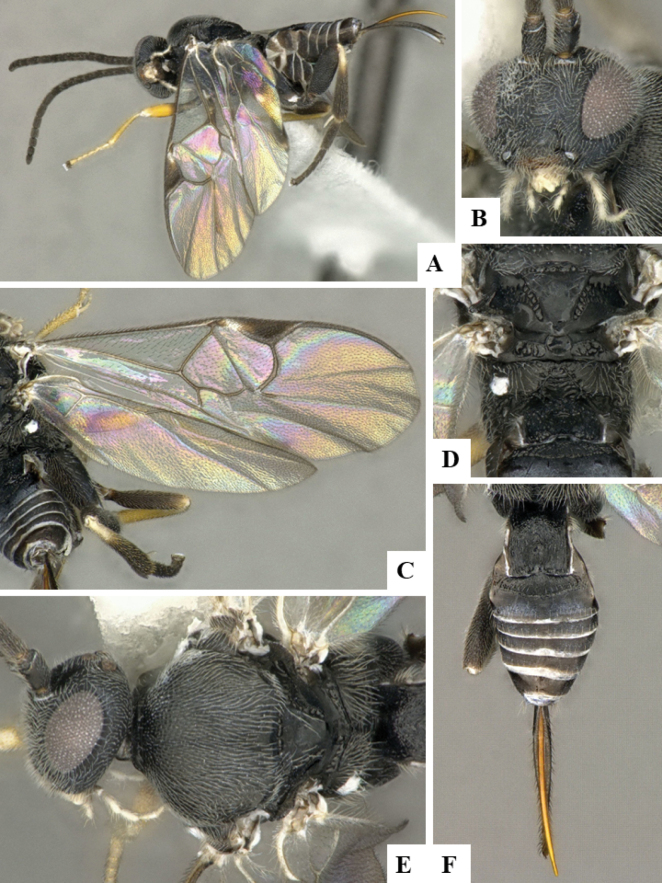
*Dolichogenideabradzlotnicki* Fernandez-Triana & Boudreault holotype female DHJPAR0049894 **A** habitus, lateral **B** head, frontal **C** wings **D** propodeum, dorsal **E** mesoscutum, dorsal **F** metasoma, dorsal.

##### Diagnostic description.

T1 parallel-sided; T1 with strong, longitudinal striae on posterior 0.5; T2 transverse; T2 mostly sculptured, but with smooth areas centrally and near posterior margin; ovipositor sheath 1.2× as long as metatibia; tegula and humeral complex, all coxae, anterior 0.5 of profemur, mesofemur, metafemur and most of metatibia (except for anterior 0.2 which is yellow-white) dark brown to black; body length: 2.60 mm; fore wing length: 2.75 mm;. *D.bradzlotnicki* is distinctive because of T1 and T2 sculpture, ovipositor sheath length, and legs color.

##### Distribution.

Costa Rica.

##### Biology.

Solitary. Depressariidae: *Chlamydastisvividella*, *Stenoma* Janzen199.

##### DNA barcoding data.

BINBOLD:ACC1295 (2 sequences, 2 barcode compliant).

##### Etymology.

Named in honor of Dr. Brad Zlotnick of Palo Alto, California for his three decades of steady and enthusiastic interest in, and support of, all of the GDFCF and ACG activities as a member of the Board of Directors for the Guanacaste Dry Forest Conservation Fund in its integration with Area de Conservación Guanacaste, Costa Rica.

#### ﻿ Dolichogenidea bushnelli

Taxon classificationAnimaliaHymenopteraBraconidae

(Muesebeck, 1933)

ED1EDA2A-5746-54C1-B9EE-CEF978931698

Figs 34A–G , 35A–C

##### Notes.

This species has not been found in the Neotropical region, but it is included in the key above because of its occurrence in at least one southern USA state (Florida).

**Figure 34.**
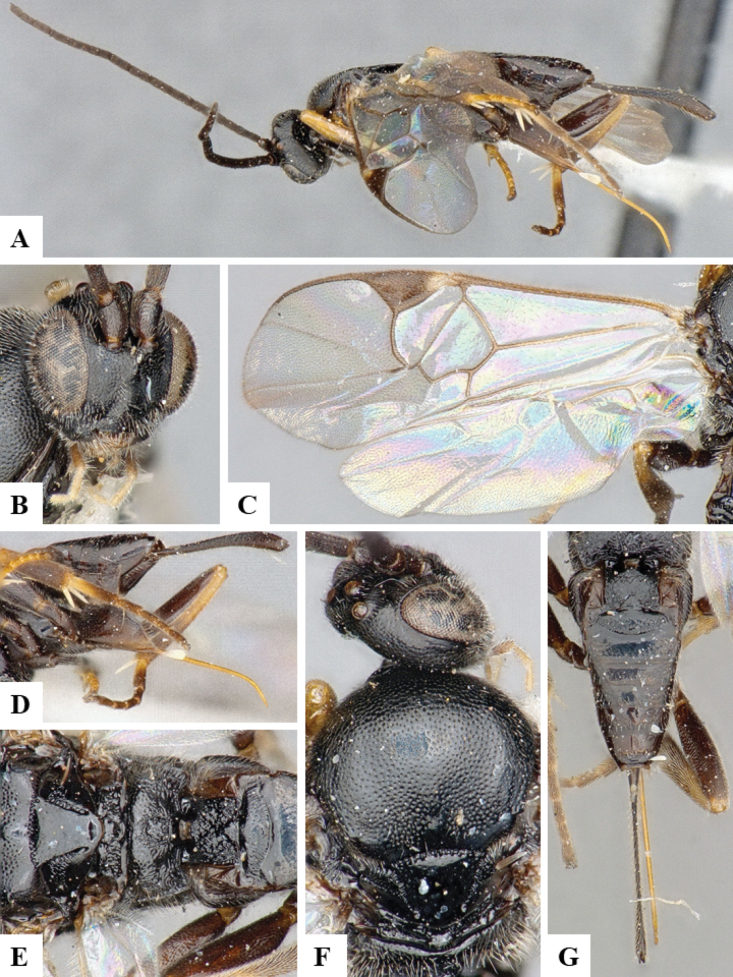
*Dolichogenideabushnelli* (Muesebeck) female CNCHYM00992 **A** habitus, lateral **B** head, fronto-lateral **C** wings **D** ovipositor, lateral **E** propodeum & T1–T2, dorsal **F** mesoscutum, dorsal **G** metasoma, dorsal.

**Figure 35.**
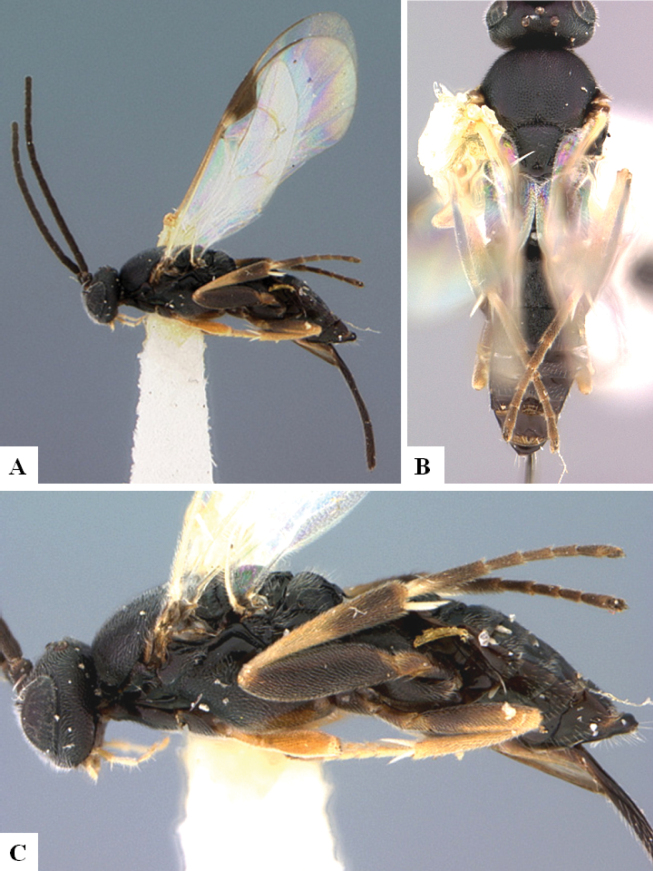
*Dolichogenideabushnelli* (Muesebeck) holotype female **A** habitus, lateral **B** habitus, dorsal **C** close-up of habitus, dorsal.

#### ﻿ Dolichogenidea caldas

Taxon classificationAnimaliaHymenopteraBraconidae

Fernandez-Triana & Boudreault sp. nov.

A624A581-4F56-5292-AAC5-BA6B6C45BEA1

https://zoobank.org/CF241704-FA51-448A-9DDE-9B0DFE25DB38

Fig. 36A–G

##### Type material.

***Holotype*.** Colombia • Female, CNC; Caldas, Elfin forest; 5°15'N, 75°25'W; 3,300–3,500 m; 4.iv.1973; J. Helava leg.; Voucher code: CNC1180001. [See notes below for accuracy of the type locality]. ***Paratype*.** Colombia • 1 Male, CNC; CNC1180027.

**Figure 36.**
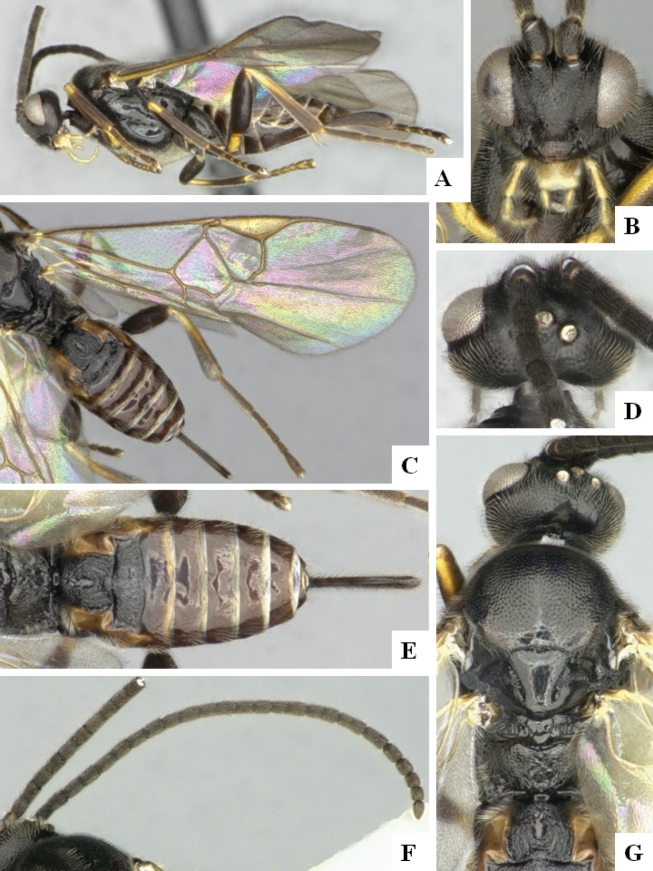
*Dolichogenideacaldas* Fernandez-Triana & Boudreault holotype female CNC1180001 **A** habitus, lateral **B** head, frontal **C** wings **D** head, dorsal **E** metasoma, dorsal **F** antennae **G** mesosoma, dorsal.

##### Diagnostic description.

F15 sub-cubic (1.1× as long as high); propodeum areola mostly defined, but open on anterior ~ 0.3, and without defined transverse carinae; T1 and T2 heavily sculptured with strong longitudinal striae covering posterior 0.6 of T1 and most of T2 (except for small central area which is smooth); T1 mostly parallel-sided but slightly widening towards posterior margin; ovipositor sheath spatula-shaped and 0.8× as long as metatibia length; tegula dark brown to black, humeral complex mostly dark brown; pterostigma bright yellow-white but with thin brown margins, most of wing veins pale yellow-brown; coxae dark brown to black, rest of legs mostly dark brown (except for bright yellow trochanters and trochantelli, dorsal margin of profemur, anterior 0.1 of pro- and mesotibiae and anterior 0.3 of metatibia, and all tibial spurs white-yellow); body length: 3.80 mm; fore wing length: 4.20 mm. Among species with T1 and T2 heavily sculptured and T1 slightly broadening towards posterior margin, *D.caldas* can be distinguished by the shape of its ovipositor sheath, distinctive leg and pterostigma coloration, propodeum areola and body size.

##### Distribution.

Colombia.

##### Biology.

No host data available.

##### DNA barcoding data.

No data.

##### Etymology.

Named after the Colombian department where the specimens were collected.

##### Notes.

The holotype label probably contains a typo resulting in an incorrect longitude value for the locality. It is stated as “76°25'W”; however, that figure would place the locality in a different Colombian department (Risaralda instead of Caldas) and at a much lower elevation (~ 250 m) than what the label states is the actual elevation (3,300–3,500 m). The paratype (which has the same label data) has a correction of the longitude from 76 to 75 degrees (added manually), which would place the locality in the right Colombian department (Caldas) and at an elevation (~ 3,000 m) that approximates what is in the label. Thus, here we are changing the info for the holotype to follow the correction done in the paratype label. It is likely that the minute value associated with the longitude is also slightly inaccurate (probably a factor of GPS accuracy at the time), as having the minutes changed from 25’ to 22’ or 23’ would place the locality at the correct elevation (3,300–3,500 m) and on or very near to the road crossing those mountains. Whatever the exact coordinates of the type locality might actually be is not that important though, as a range of 2 or 3 minutes in the longitude value translates to a maximum of 3 km of separation in that extensive area of elfin forests.

#### ﻿ Dolichogenidea carlosalvaradoi

Taxon classificationAnimaliaHymenopteraBraconidae

Fernandez-Triana & Boudreault sp. nov.

5D8AB6BA-D163-5493-AD88-CF03639B45A4

https://zoobank.org/A70CF60A-5628-4CE2-825F-DF1CF3023955

Fig. 37A–F

##### Type material.

***Holotype*.** Costa Rica • Female, CNC; Guanacaste, Area de Conservación Guanacaste, Sector Cacao, Cerro Pedregal; 10.9277, -85.4745; 1,080 m; 18.xii.2008; D. H. Janzen & W. Hallwachs leg.; Malaise trap; Voucher code: DHJPAR0031380.

**Figure 37.**
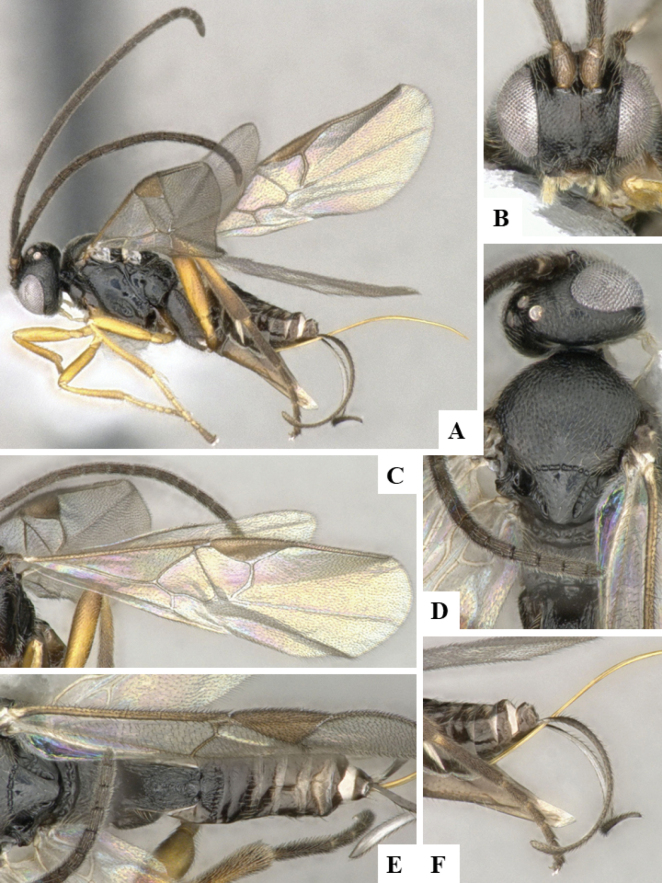
*Dolichogenideacarlosalvaradoi* Fernandez-Triana & Boudreault holotype female DHJPAR0031380 **A** habitus, lateral **B** head, frontal **C** wings **D** mesosoma, dorsal **E** metasoma, dorsal **F** ovipositor, lateral.

##### Diagnostic description.

Propodeum areola comparatively narrow (its height > 1.5× its central width) and open anteriorly; T1 and T2 heavily sculptured with strong longitudinal striae; T1 comparatively thin and mostly parallel-sided but posterior 0.1–0.3 slightly narrowing towards posterior margin; T2 broadly trapezoidal in shape (with posterior margin slightly arcuate); ovipositor sheath ≤ 1.2× metatibia length; ovipositor strongly sinuate; tegula brown; metacoxa entirely dark brown; body length: 2.30 mm; fore wing length: 2.30 mm; BINBOLD:AAM5848 which is 8.17% different from the nearest BIN in BOLD as of March 2022. The color of metacoxa and tegula, shape of ovipositor and propodeum areola distinguish it from all species with T1 and T2 heavily sculptured and T1 comparatively thin.

##### Distribution.

Costa Rica.

##### Biology.

No host data available.

##### DNA barcoding data.

BINBOLD:AAM5848 (1 sequence, barcode compliant).

##### Etymology.

Named in honor of Sr. Carlos Alvarado of San Jose, Costa Rica, in recognition of his bravery for being the President of Costa Rica for 2018–2022 and strongly supporting the non-damaging biodevelopment of its wild biodiversity.

#### ﻿ Dolichogenidea carlosmanuelrodriguezi

Taxon classificationAnimaliaHymenopteraBraconidae

Fernandez-Triana & Boudreault, 2019

DC1F4946-A8DC-5F59-8F5C-D3D45D9ECFB3

Figs 38A–E , 156A

##### Notes.

Full details for this species in Fernandez-Triana et al. (2019). See also the key and Table 1 above.

**Figure 38.**
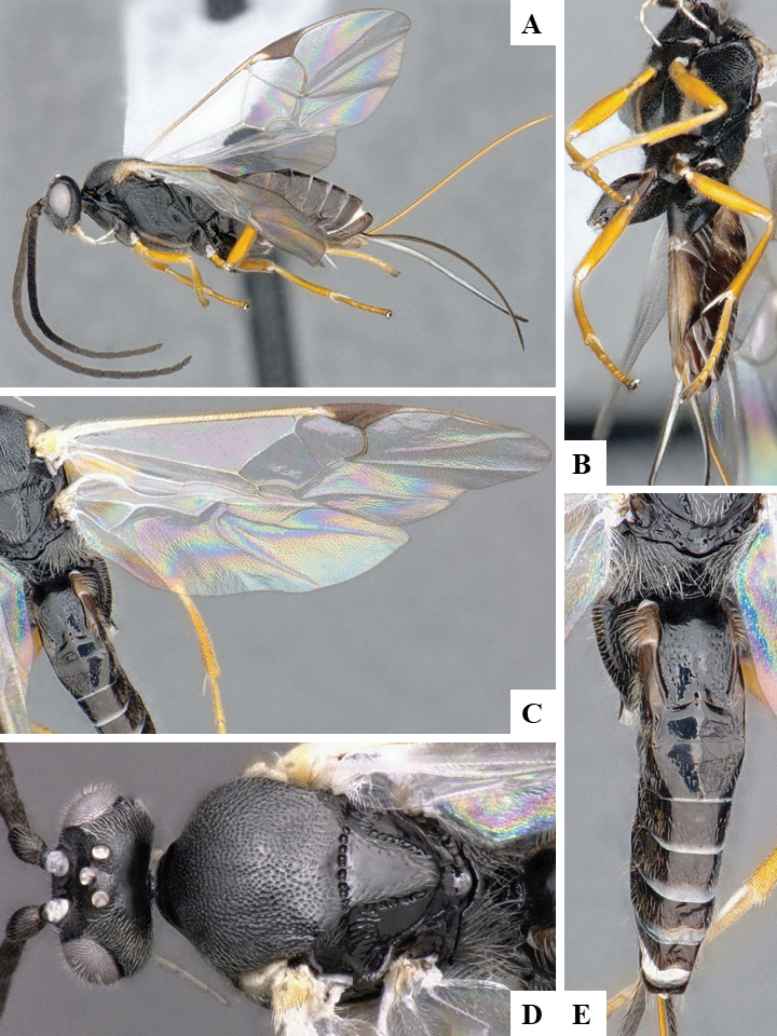
*Dolichogenideacarlosmanuelrodriguezi* Fernandez-Triana & Boudreault holotype female DHJPAR0039047 **A** habitus, lateral **B** habitus, ventral **C** wings **D** mesosoma, dorsal **E** metasoma, dorsal.

#### ﻿ Dolichogenidea carlosviquezi

Taxon classificationAnimaliaHymenopteraBraconidae

Fernandez-Triana & Boudreault sp. nov.

4F491750-D876-57D6-A743-0194692F5212

https://zoobank.org/74187E37-AE05-4AF0-AB1B-97EF2451B736

Fig. 39A–G

##### Type material.

***Holotype*.** Costa Rica • Female, CNC; Guanacaste, Area de Conservación Guanacaste, Santa Rosa National Park, site # H-10; 300 m; 1–29.xi.1986; Gauld & Janzen leg.; Malaise trap; Voucher code: CNC1179847.

**Figure 39.**
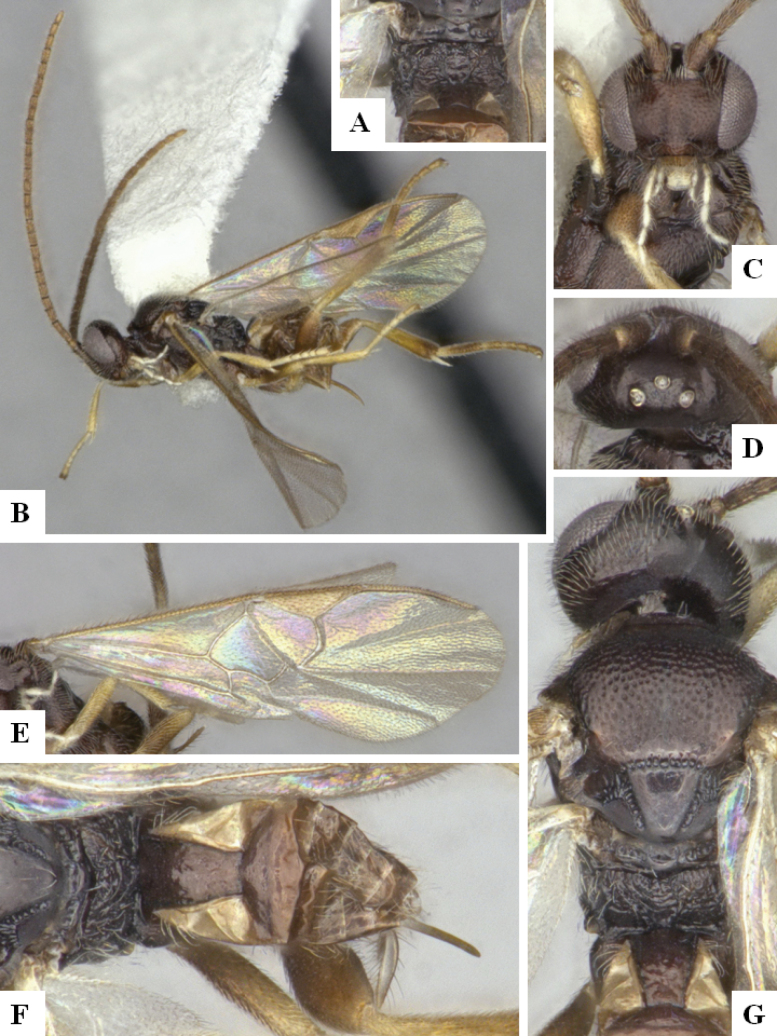
*Dolichogenideacarlosviquezi* Fernandez-Triana & Boudreault holotype female CNC1179847 **A** propodeum, dorsal **B** habitus, lateral **C** head, frontal **D** head, dorsal **E** wings **F** metasoma, dorsal **G** mesosoma, dorsal.

##### Diagnostic description.

Scutellar disc entirely smooth and shiny; propodeum with carinae clearly defining a more or less complete areola; T1 and T2 smooth; T1 evenly narrowing towards posterior margin (T1 width at anterior margin 2.2× T1 width at posterior margin); ovipositor sheath comparatively much shorter, its length (0.29 mm) 0.50× metatibia length (0.62 mm); legs mostly yellow or pale brown, with all coxae brown, metafemur pale brown and metatibia yellow with posterior 0.3 pale brown; body length: 1.90 mm; fore wing length: 2.10 mm. This species can be recognized by the unique combination of entirely smooth scutellar disc, T1 and T2, short ovipositor sheaths, narrowed T1, small body size and comparatively pale brown legs.

##### Distribution.

Costa Rica.

##### Biology.

No host data available.

##### DNA barcoding data.

No data.

##### Etymology.

Named in honor of Sr. Carlos Viquez of Costa Rica in recognition of his willingness to sell his forested property to the Guanacaste Dry Forest Conservation Fund for incorporation in the restoration efforts of Area de Conservación Guanacaste in 2021.

#### ﻿ Dolichogenidea cedenoae

Taxon classificationAnimaliaHymenopteraBraconidae

Fernandez-Triana & Boudreault nom. nov.

E7F775DD-4AD8-52C9-8E88-58AC25BCEA49

Figs 40A–E , 165B

Dolichogenidea yeimycedenoae Fernandez-Triana & Boudreault, 2019; junior synonym.

##### Notes.

Full details for this species in Fernandez-Triana et al. (2019). See also the key, Table 1 and discussion at the beginning of the Results section above.

**Figure 40.**
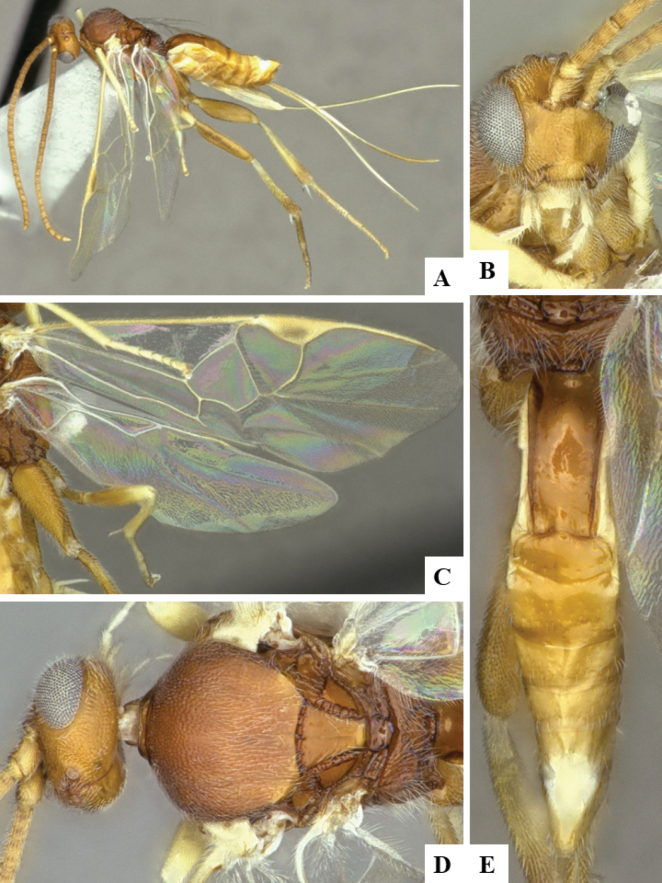
*Dolichogenideacedenoae* Fernandez-Triana & Boudreault holotype female DHJPAR0054623 **A** habitus, lateral **B** head, frontal **C** wings **D** mesosoma, dorsal **E** metasoma, dorsal.

#### ﻿ Dolichogenidea chichicastenango

Taxon classificationAnimaliaHymenopteraBraconidae

Fernandez-Triana & Boudreault sp. nov.

A4E835D6-34A3-5578-ABA4-36CD45B8B65C

https://zoobank.org/4611B8C3-7EDC-4879-BCD3-7AA0C27407FC

Fig. 41A–F

##### Type material.

***Holotype*.** Guatemala • Female, CNC; El Quiché, 2 km S of Chichicastenango on Rio Tesoro; 2,000 m; 11.ix.1987; M. Sharkey leg.; Voucher code: CNC1196553.

**Figure 41.**
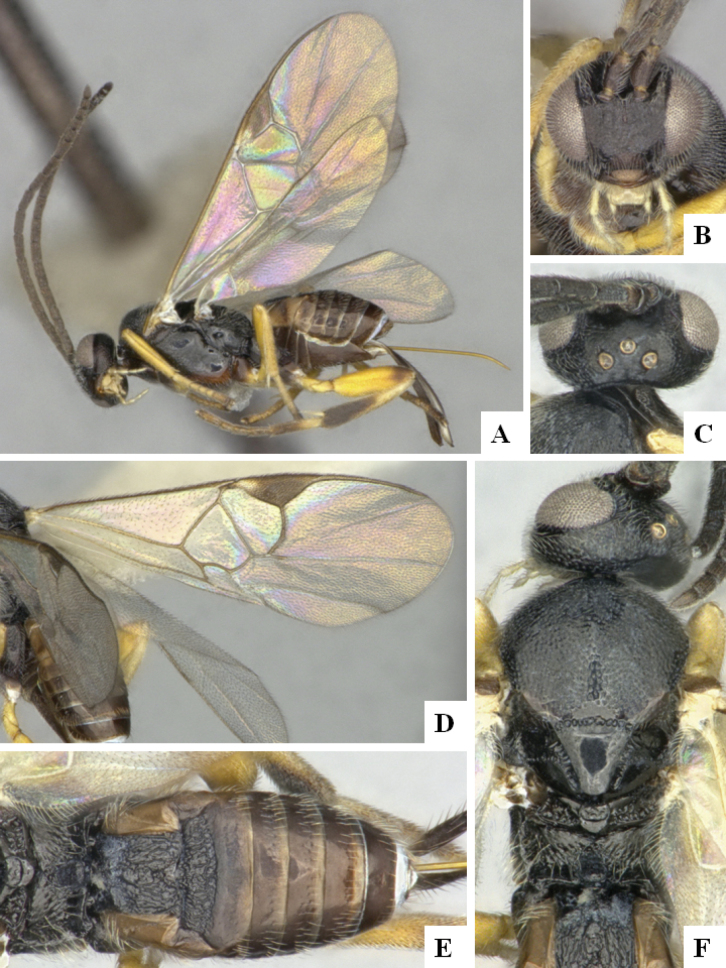
*Dolichogenideachichicastenango* Fernandez-Triana & Boudreault holotype female CNC1196553 **A** habitus, lateral **B** head, frontal **C** head, dorsal **D** wings **E** metasoma, dorsal **F** mesosoma, dorsal.

##### Diagnostic description.

F15 length 1.2× its height; T1 and T2 heavily sculptured with strong longitudinal striae; T1 length < 1.5× T1 width at posterior margin; T2 broadly rectangular (but posterior margin sinuate), covering most surface of tergum; tegula white-yellow, clearly paler than brown humeral complex; pterostigma with pale spot on anterior 0.25; pro- and mesocoxae dark reddish brown; metafemur almost entirely yellow (small brown spot on posterior 0.1); metatibia dark brown to black on posterior 0.7; all laterotergites and sternites pale brown to dark brown; body length: 2.75 mm; fore wing length: 3.06 mm. Among all species with heavily sculptured T1 and T2, T1 comparatively broad and T2 rectangular, *D.chichicastenango* can be distinguished by the shape of F15 and color of tegula, humeral complex, pterostigma; procoxa, mesocoxa, metafemur and metatibia, laterotergites and sternites. The closest species, *D.felipechavarriai* from Costa Rica, has paler coloration of legs and metasoma, and comparatively longer F15.

##### Distribution.

Guatemala.

##### Biology.

No host data available.

##### DNA barcoding data.

No data.

##### Etymology.

Named after the type locality.

#### ﻿ Dolichogenidea christinaagapakisae

Taxon classificationAnimaliaHymenopteraBraconidae

Fernandez-Triana & Boudreault sp. nov.

3BA85F32-E692-508D-B280-AAEC98485615

https://zoobank.org/202499DC-8049-41C1-BF3F-949ACEC1F934

Fig. 42A–F

##### Type material.

***Holotype*.** Costa Rica • Female, CNC; Alajuela, Area de Conservación Guanacaste, Sector Rincon Rain Forest, Sendero Rincon; 10.8962, -85.27769; 430 m; 28.iii.2012; Jose Perez leg.; Host: gelJanzen01 Janzen23; Voucher code: DHJPAR0049406; Host voucher code: 12-SRNP-41329.

**Figure 42.**
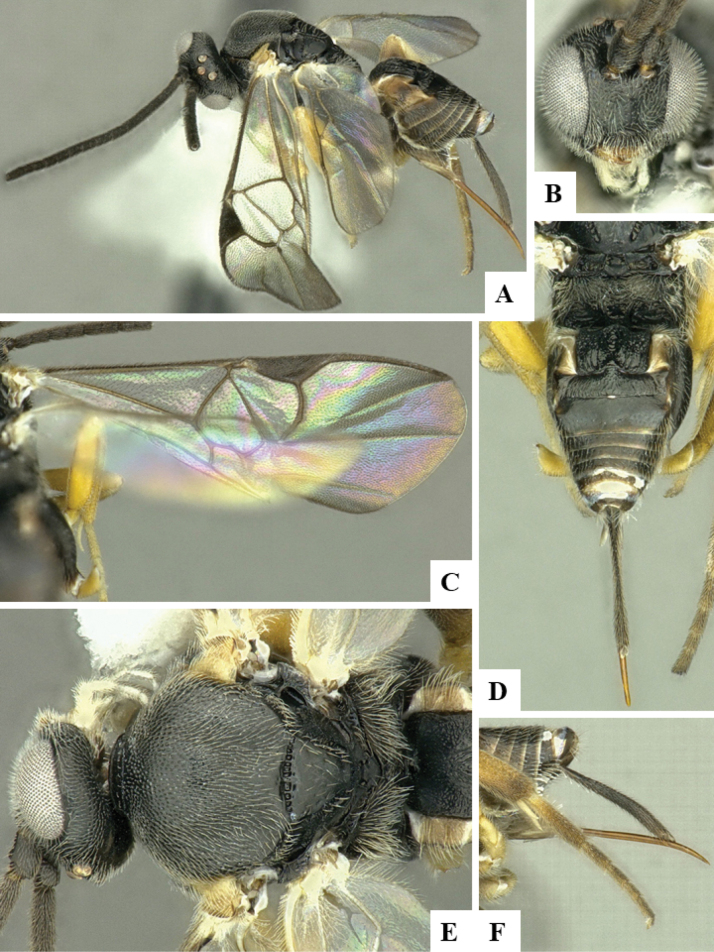
*Dolichogenideachristinaagapakisae* Fernandez-Triana & Boudreault holotype female DHJPAR0049406 **A** habitus, lateral **B** head, frontal **C** wings **D** metasoma, dorsal **E** mesoscutum, dorsal **F** ovipositor, lateral.

##### Diagnostic description.

Ocelli comparatively larger, ocular ocellar line < 2.2× posterior ocellar line; anteromesoscutum more or less shiny but with well-marked punctures; scutellar disc smooth and shiny, without punctures; fore wing vein 2CU medially raised or arched in a sharp angle; T1 strongly sculptured on posterior 0.5; T2 mostly sculptured but smooth centrally or along margins; T2 transverse, its width at posterior margin ~ 3.5× its central length; tegula and humeral complex yellow; all coxae brown to dark brown; metatibia (except for darker spot on posterior 0.1) and part of metafemur (anterior 0.2 and posterior 0.1) yellow; body length: 3.03 mm; fore wing length: 3.22 mm. The shape and sculpture of T1 and T2, ocelli size, fore wing venation and color of tegula, humeral complex and legs distinguish this species among all others with T2 sculptured but transverse and dark coxae.

##### Distribution.

Costa Rica.

##### Biology.

Solitary. Depressariidae: *Gonionota* Janzen22, Gelechiidae: gelJanzen01 Janzen23.

##### DNA barcoding data.

BINBOLD:AAI9755 (4 sequences, 4 barcode compliant).

##### Etymology.

Named in honor of Dr. Christiana Agapakis of Gingko Bioworks in recognition of her warm and detailed 2022 welcome to representatives from GDFCF/ACG exploring the potential of a synthetic biology foundry with strong potential for non-damaging development of complex tropical biodiversity genomics as a potential product from wild tropical ecosystems, and therefore increasing their intact desirability by tropical societies.

#### ﻿ Dolichogenidea claudiadoblesae

Taxon classificationAnimaliaHymenopteraBraconidae

Fernandez-Triana & Boudreault sp. nov.

E7C67608-440A-5DE3-9776-176D6DFE172F

https://zoobank.org/AF09D24A-F358-4A1A-BDA0-98FEF17C568D

Fig. 43A–F

##### Type material.

***Holotype*.** Costa Rica • Female, CNC; Guanacaste, Area de Conservación Guanacaste, Sector Santa Rosa, Area Administrativa; 10.8376, -85.6187; 295 m; 25.xii.2008; D. H. Janzen & W. Hallwachs leg.; Malaise trap; Voucher code: DHJPAR0031810. ***Paratypes*.** Costa Rica • 7 Females, CNC; DHJPAR0031862, DHJPAR0031863, DHJPAR0031585, DHJPAR0031758, DHJPAR0031684, DHJPAR0031719, DHJPAR0031844.

**Figure 43.**
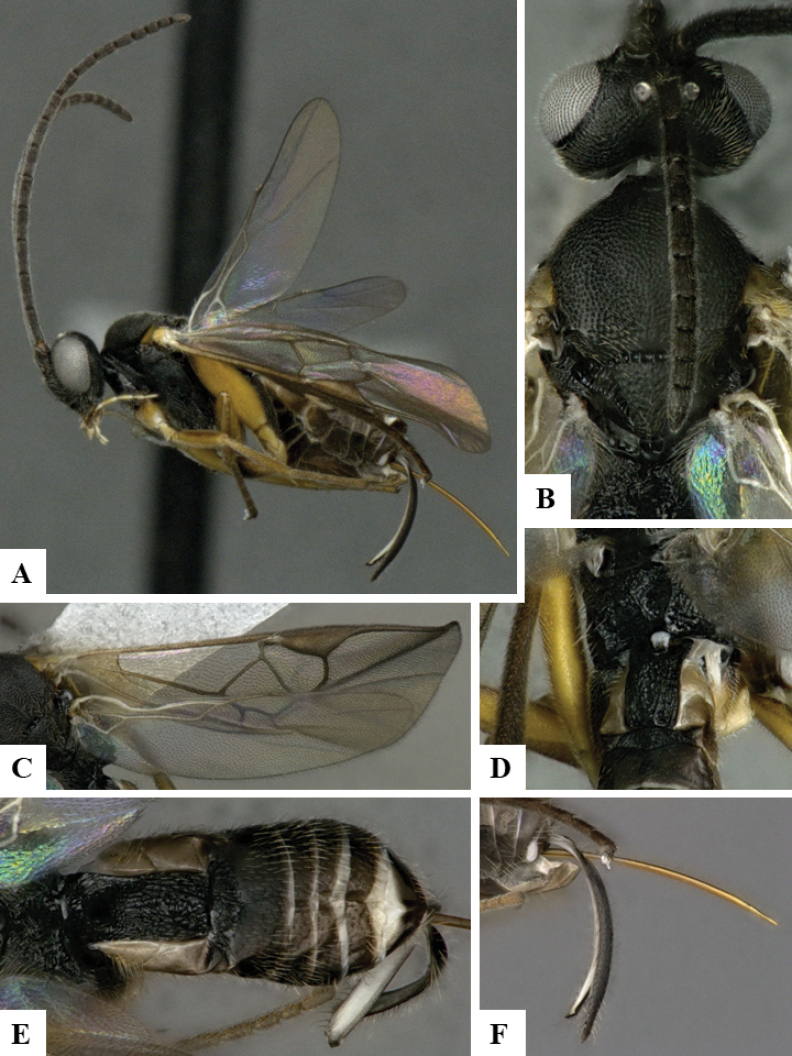
*Dolichogenideaclaudiadoblesae* Fernandez-Triana & Boudreault holotype female DHJPAR0031810 **A** habitus, lateral **B** mesosoma, dorsal **C** wings **D** propodeum & T1–T3, dorsal **E** metasoma, dorsal **F** ovipositor, lateral.

##### Diagnostic description.

Scutellar disc with coarse punctures; anterior half of mesopleuron and anteromesoscutum with relatively coarse punctures; propodeum areola comparatively broad (its height ~ 1.2× its central width) and open anteriorly; T1 and T2 heavily sculptured with strong longitudinal striae; T1 comparatively thin and mostly parallel-sided but posterior 0.1–0.3 slightly narrowing towards posterior margin; T2 broadly trapezoidal in shape (with posterior margin slightly arcuate); ovipositor sheath ≤ 1.2× metatibia length; ovipositor not sinuate; tegula and humeral complex yellow; pterostigma pale brown but centrally paler than margins; metacoxa entirely dark brown; metatibia entirely brown to dark brown; body length: 2.70–3.00 mm; fore wing length: 2.80–3.10 mm; BINBOLD:AAD2236, which is 5.61% different from the nearest BIN in BOLD as of March 2022. The color of tegula, metacoxa, metatibia and pterostigma, and the sculpture of scutellar disc and anterior half of mesopleuron and anteromesoscutum separates this species from others with T1 and T2 heavily sculptured and T1 comparatively thin.

##### Distribution.

Costa Rica.

##### Biology.

No host data available.

##### DNA barcoding data.

BINBOLD:AAD2236 (109 sequences, 109 barcode compliant).

##### Etymology.

Named in honor of Sra. Claudia Dobles of San Jose, Costa Rica in recognition of her support for her family, husband and the non-damaging biodevelopment of Costa Rica during his term as President (2018–2022).

#### ﻿ Dolichogenidea croceicornis

Taxon classificationAnimaliaHymenopteraBraconidae

(Muesebeck, 1958) comb. nov.

E26F4EDE-BDA1-53F0-8AD2-FC80B13B5D69

Fig. 44A–F

##### Notes.

We have examined the holotype (USNM) and this species clearly belongs to *Dolichogenidea*, based on the vannal lobe entirely setose.

**Figure 44.**
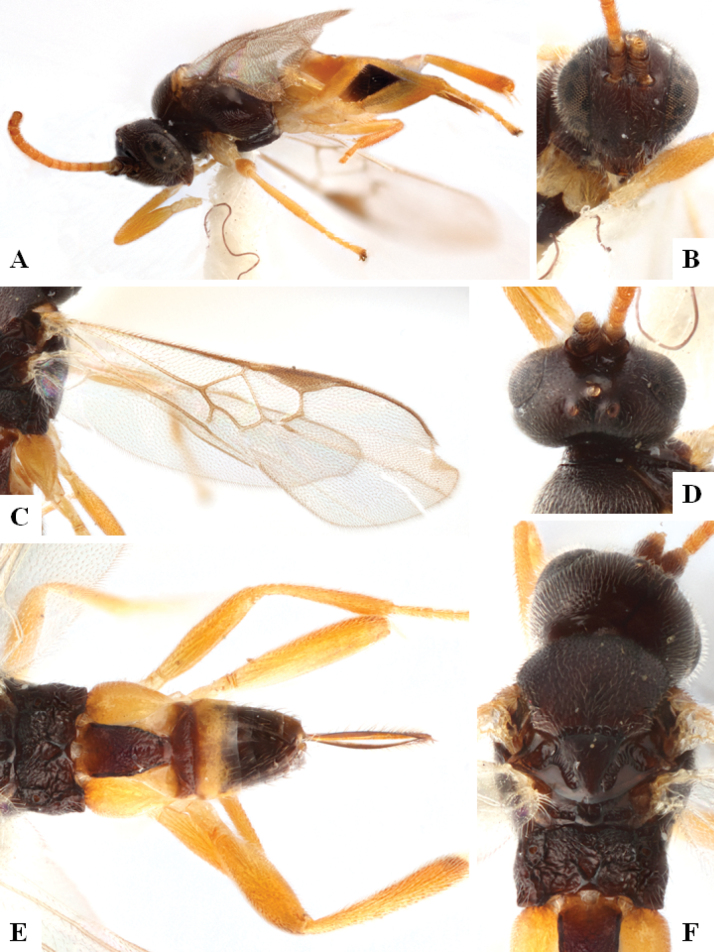
*Dolichogenideacroceicornis* (Muesebeck) holotype female USNMENT01569140 **A** habitus, lateral **B** head, frontal **C** wings **D** head, dorsal **E** metasoma, dorsal **F** mesosoma, dorsal.

#### ﻿ Dolichogenidea dole

Taxon classificationAnimaliaHymenopteraBraconidae

Fernandez-Triana & Boudreault sp. nov.

92BA8FAD-7D96-52A3-8481-ACACAC7EB9C6

https://zoobank.org/EDAC4EB6-AEB5-4F8E-830F-68E5827C224E

Fig. 45A–G

##### Type material.

***Holotype*.** Costa Rica • Female, CNC; Guanacaste, Area de Conservación Guanacaste, Sector Cacao, Sendero Arenales; 10.92471, -85.46738; 1,080 m; 18.xii.2008; D. H. Janzen & W. Hallwachs leg.; Malaise trap; Voucher code: DHJPAR0031280.

**Figure 45.**
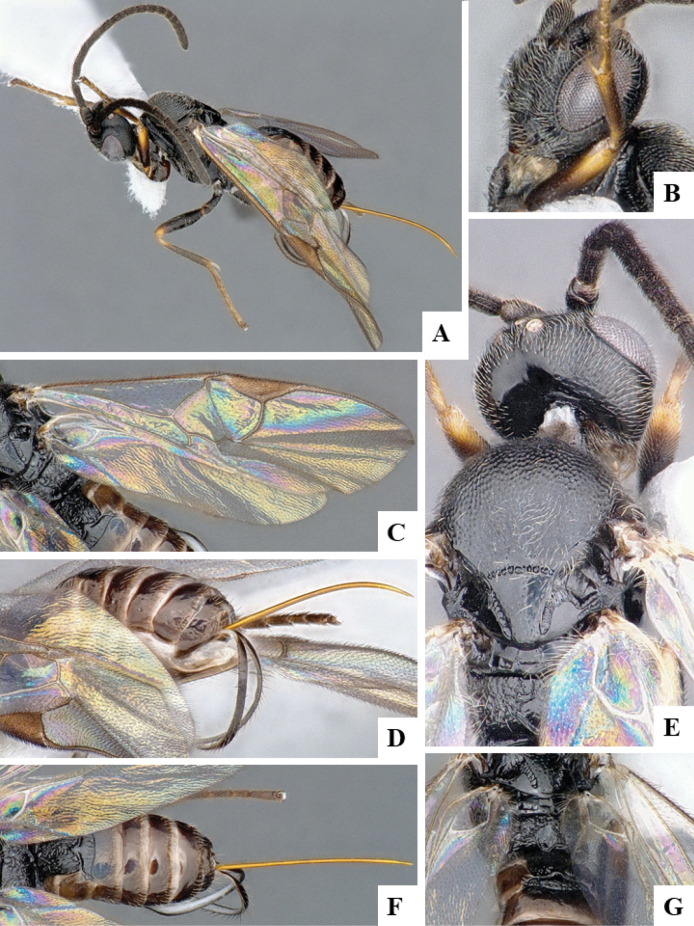
*Dolichogenideadole* Fernandez-Triana & Boudreault holotype female DHJPAR0031280 **A** habitus, lateral **B** head, lateral **C** wings **D** ovipositor, lateral **E** mesosoma, dorsal **F** metasoma, dorsal **G** propodeum, dorsal.

##### Diagnostic description.

T1 length medially ~ 3.0× its width at posterior margin; T2 more or less trapezoidal in shape; T2 sculptured around margins, centrally smooth; hypopygium with single, small pleat; ovipositor sheath < 0.5× metatibia length; all coxae brown to dark brown; metafemur entirely to mostly yellow (at most with darker spot on posterior 0.3 or less); body length 2.59 mm; fore wing length: 2.56 mm. Among all species with dark coxae and T2 at least partially smooth, *D.dole* can be distinguished by its almost unpleated hypopygium and short ovipositor sheath.

##### Distribution.

Costa Rica.

##### Biology.

No host data available.

##### DNA barcoding data.

BINBOLD:AAM5739 (1 sequence, barcode compliant).

##### Etymology.

Named in honor of the Dole Pineapple Company plantation in the central northern lowlands of what used to be Costa Rican Caribbean coastal rain forest, for being willing to support Malaise trapping for all insects for the Costa Rican BioAlfa DNA barcode library that live in the plantation and adjacent secondary successional rain forest in 2022, and to understand the biodiversity dynamics of the crop itself.

#### ﻿ Dolichogenidea encruzilhada

Taxon classificationAnimaliaHymenopteraBraconidae

Fernandez-Triana & Boudreault sp. nov.

2BD27CAF-7F2E-5ABA-AB6F-472D4D4C3C5A

https://zoobank.org/DEF202BF-36E8-41E2-96CD-9B86DF7C30EE

Fig. 46A–F

##### Type material.

***Holotype*.** Brazil • Female, CNC; Bahia, Encruzilhada; 980 m; xi.1974; M. Alvarenga leg.; voucher code: CNC1180167.

**Figure 46.**
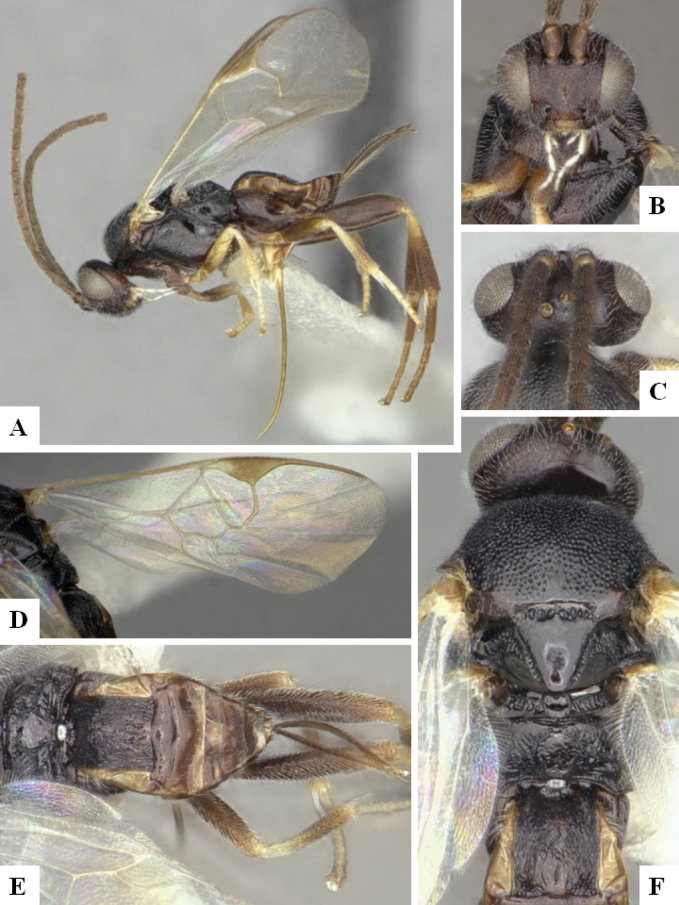
*Dolichogenideaencruzilhada* Fernandez-Triana & Boudreault holotype female CNC1180167 **A** habitus, lateral **B** head, frontal **C** head, dorsal **D** wings **E** metasoma, dorsal **F** mesosoma, dorsal.

##### Diagnostic description.

T1 strongly sculptured with longitudinal striae; T1 parallel-sided, 1.3× as long as its width at posterior margin; T2 entirely smooth and transverse; ovipositor sheath around same length (1.15×) than metatibia length; tegula and humeral complex yellow; pterostigma mostly brown with pale spot at base; all coxae brown; meso- and metatrochantelli dark brown to black; profemur yellow, meso- and metafemora brown; metatibia mostly brown, with only anterior 0.2 yellow; body mostly dark brown to pale brown; comparatively smaller species; body length: 2.30 mm; fore wing length: 2.60 mm. Among species with T1 strongly sculptured but T2 smooth and transverse, this species can be recognized by the coloration of tegula, humeral complex and legs, pterostigma and length of ovipositor sheaths. *D.luishamiltoni* is similar but can be differentiated from *D.encruzilhada* because the former has darker coloration, slightly broader T1 and comparatively larger body size.

##### Distribution.

Brazil (BA).

##### Biology.

No host data available.

##### DNA barcoding data.

No data.

##### Etymology.

Named after the municipality of Encruzilhada, where the holotype was collected, in the Atlantic Forest (Mata Atlântica). This is a region characterized by high biodiversity and endemism, but also needing conservation due to its high degree of deforestation threatening many plant and animal species with extinction.

#### ﻿ Dolichogenidea ensiger

Taxon classificationAnimaliaHymenopteraBraconidae

(Say, 1836)

34E35C81-9CC7-5FB2-9E9B-B9524EA78EA9

Fig. 47A–E

##### Distribution.

Canada (AB, MB, NB, NL, NS, NT, ON, PE, QC, SK), Costa Rica, United States (AK, AL, CO, CT, DE, DC, FL, GA, IL, IN, IA, KS, LA, MA, MD, MO, MT, NH, NJ, NY, NC, TN).

**Figure 47.**
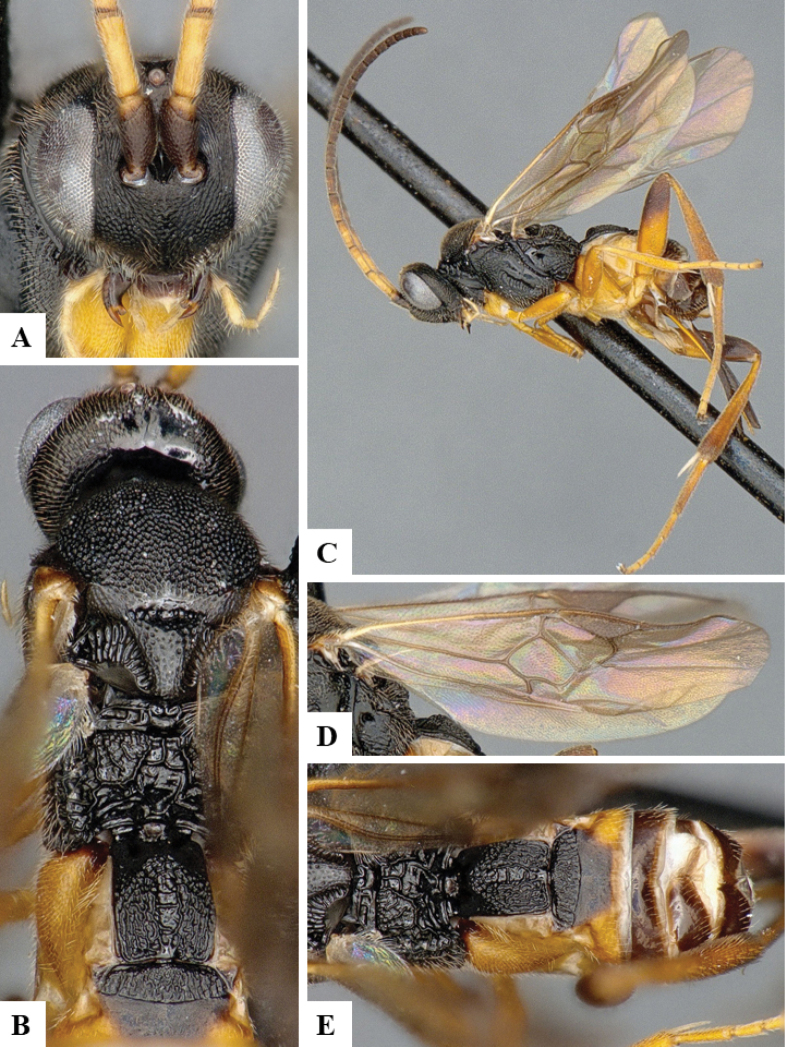
*Dolichogenideaensigner* (Say) female CNC474714 **A** head, frontal **B** mesosoma, dorsal **C** habitus, lateral **D** wings **E** metasoma, dorsal.

##### Biology.

Solitary (?). Crambidae: *Fissicrambusmutabilis*, *Neodactriazeellus*; Tortricidae: *Choristoneurafreemani*, *Epiblemastrenuana*.

##### DNA barcoding data.

BINBOLD:AAA3764 (335 sequences, 333 barcode compliant).

##### Notes.

This species is here recorded for the first time in the Neotropical region, based on a single specimen from ACG, Costa Rica (voucher code ON101953). All other known records are from North America, where the species is widely distributed (e.g., Fernandez-Triana et al. 2014b, 2014c).

#### ﻿ Dolichogenidea ericpalolai

Taxon classificationAnimaliaHymenopteraBraconidae

Fernandez-Triana & Boudreault sp. nov.

BAA37BA7-6DC4-5E53-9772-35D5FA4F6E6F

https://zoobank.org/E6943912-4239-4DBB-9A52-77B6F7F4E3D2

Fig. 48A–G

##### Type material.

***Holotype*.** Costa Rica • Female, CNC; Alajuela, Area de Conservación Guanacaste, Sector San Cristobal, Rio Blanco Abajo; 10.9004, -85.3725; 500 m; 3.viii.2007; D. H. Janzen & W. Hallwachs leg.; Malaise trap; Voucher code: DHJPAR0026313. ***Paratypes*.** Costa Rica • 1 Female, 1 Male, CNC; DHJPAR0025187, DHJPAR0025511.

**Figure 48.**
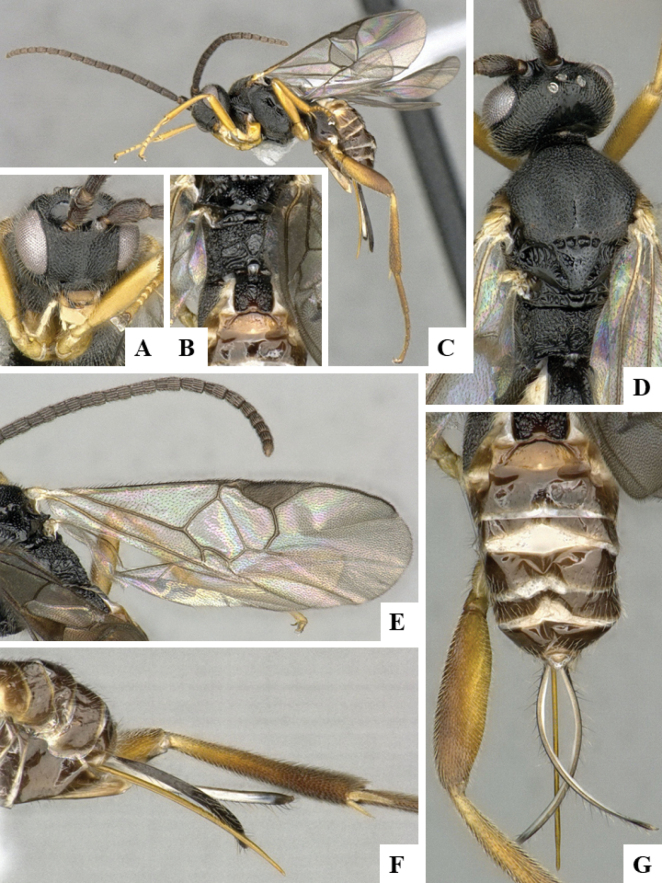
*Dolichogenideaericpalolai* Fernandez-Triana & Boudreault holotype female DHJPAR0026313 **A** head, frontal **B** propodeum & T1–T3, dorsal **C** habitus, lateral **D** mesosoma, dorsal **E** wings **F** ovipositor, lateral **G** metasoma, dorsal.

##### Diagnostic description.

Anteromesoscutum with coarse, deep and dense punctures (separation between punctures less than individual puncture diameter); face, propleuron, pronotum, most of mesopleuron, scutellar disc, and most of outer side of metacoxa mostly to entirely covered by relatively coarse punctures; T2 smooth; pro- and mesocoxae yellow; metacoxa entirely to almost entirely dark brown; posterior 0.1–0.2 of T1 (centrally) and entire T2 yellow to pale brown-yellow; T3+ pale brown; body length: 2.84–3.16 mm; fore wing length: 2.81–2.88 mm. The coloration of T1–T3 as well as many areas on head and mesosoma with coarse punctures distinguish this species among all with smooth T2 and pale pro- and mesocoxae.

##### Distribution.

Costa Rica.

##### Biology.

No host data available.

##### DNA barcoding data.

BINBOLD:AAF7717 (3 sequences, 3 barcode compliant).

##### Etymology.

Named in honor of Mr. Eric Palola in recognition of his decade-plus of weathering the demands of being the two-country Executive Director of the NGO Guanacaste Dry Forest Conservation Fund and its integration with the Costa Rican government’s Area de Conservación Guanacaste (ACG) in northwestern Costa Rica.

#### ﻿ Dolichogenidea ericsimoni

Taxon classificationAnimaliaHymenopteraBraconidae

Fernandez-Triana & Boudreault sp. nov.

E47A2A4F-3E79-5B57-9247-DC2F772AFF57

https://zoobank.org/DAE9FEAB-B586-4CCD-90AF-EC3C12232AF5

Fig. 49A–F

##### Type material.

***Holotype*.** Chile • Female, CNC; Malleco, Victoria; 200 m; xii.1976; Voucher code: CNC1196845.

**Figure 49.**
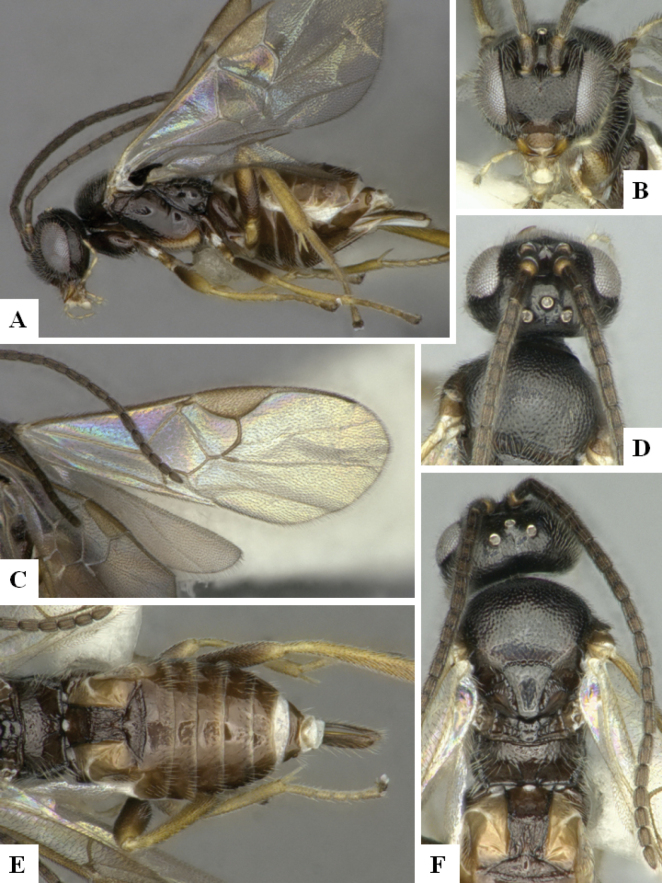
*Dolichogenideaericsimoni* Fernandez-Triana & Boudreault holotype female CNC1196845 **A** habitus, lateral **B** head, frontal **C** wings **D** head, dorsal **E** metasoma, dorsal **F** mesosoma, dorsal.

##### Diagnostic description.

Propodeum mostly smooth with only two carinae partially defining an areola on posterior 0.4; T1 mostly sculptured on posterior 0.6 and slightly narrowing towards posterior margin; T2 mostly smooth, very transverse; ovipositor sheath 0.6× as long as metatibia length; pterostigma mostly white-yellow but with thin brown margins; all coxae dark brown; profemur brown on anterior half; mesofemur mostly brown; metafemur brown on anterior half and yellow on posterior half; body length: 2.23 mm; fore wing length: 2.10 mm. Among all Neotropical species of *Dolichogenidea* with T2 mostly sculptured and dark coxae, this species has a unique combination of comparatively very short ovipositor sheath, smooth propodeum and color of metafemur. The overall smooth and shiny coloration of the body and color of pterostigma are also characteristics.

##### Distribution.

Chile.

##### Biology.

No host data available.

##### DNA barcoding data.

No data.

##### Etymology.

The second author dedicates this species in honor of Eric Simoneau, a good friend of the family. Eric’s enthusiasm, friendliness and support are greatly appreciated. The letters “eau” at the end of the last name “Simoneau” have been removed to make the species name easier to say.

#### ﻿ Dolichogenidea escobarae

Taxon classificationAnimaliaHymenopteraBraconidae

Fernandez-Triana & Boudreault sp. nov.

2300BD2C-5EB4-5487-9024-A30257C1DFA6

https://zoobank.org/328CE404-D7E6-4B36-942A-25EACE299E2C

Fig. 50A–F

##### Type material.

***Holotype*.** Brazil • Female, CNC; Guanabara, Represa Rio Grande; vii.1972; F. H. Oliveira leg.; Voucher code: CNC1179672.

**Figure 50.**
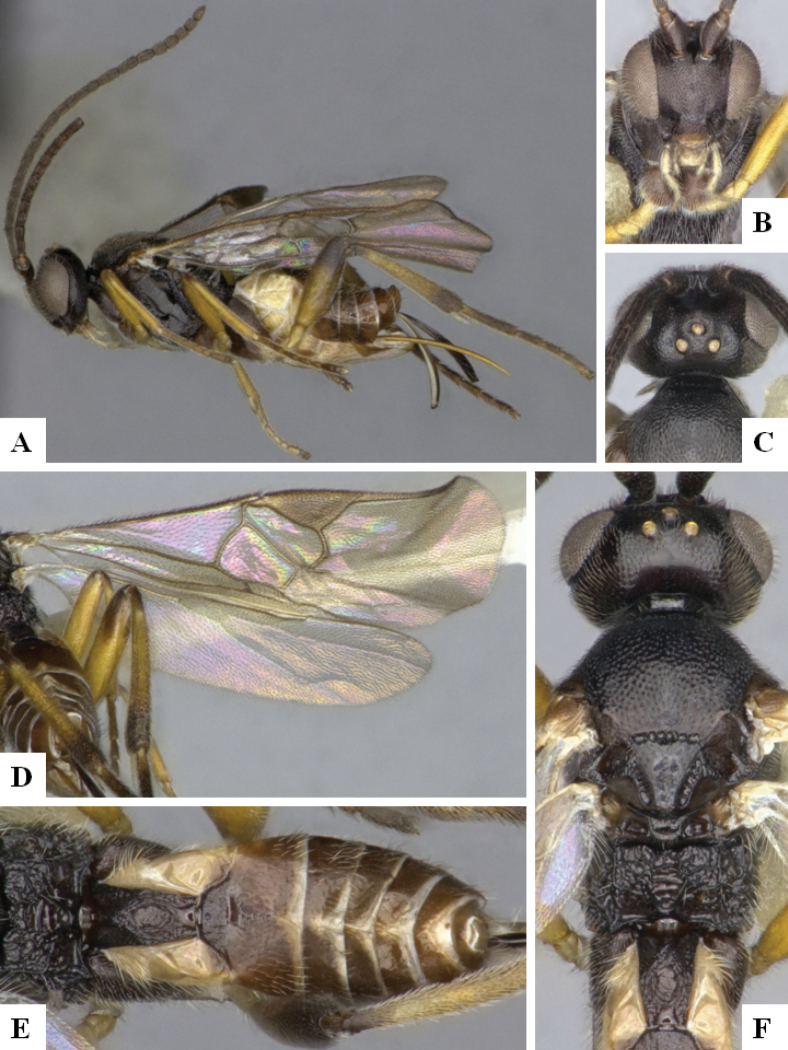
*Dolichogenideaescobarae* Fernandez-Triana & Boudreault holotype female CNC1179672 **A** habitus, lateral **B** head, frontal **C** head, dorsal **D** wings **E** metasoma, dorsal **F** mesosoma, dorsal.

##### Diagnostic description.

Anteromesoscutum mostly smooth, with relatively shallow punctures; scutellar disc smooth and shiny, without punctures; T1 strongly narrowing towards posterior margin (width at anterior margin 1.8× width at posterior margin), T1 length 4.5× its width at posterior margin; T2 weakly sculptured and trapezoidal, its width at posterior margin 2.5× its central length; ovipositor sheath 0.9× metatibia length; all coxae dark brown, all tarsus brown, rest of legs mostly yellow except for metafemur and metatibia with posterior 0.2 brown; body length: 2.83 mm; fore wing length: 2.60 mm. Among species with T1 not rectangular and T2 not strongly sculptured, this species is characterized by its strongly narrowing T1, trapezoidal T2, all coxae dark brown and ovipositor sheath almost as long as metatibia length.

##### Distribution.

Brazil (RJ).

##### Biology.

No host data available.

##### DNA barcoding data.

No data.

##### Etymology.

The second author dedicates this species in honor of Cecilia Escobar of the Smithsonian Institution (USNM) in Washington DC, USA. Cecilia has been of tremendous help when I visited the Smithsonian twice. Cecilia has been an inspiration by her joyful personality, kindness and her love of insects.

##### Notes.

About the type locality, we received a personal communication from Eduardo Shimbori to consider ‘Estado da Guanabara’ as a proxy for the current municipality of Rio de Janeiro. That is because this former state was fairly small and most of its area corresponds to what is now the territory of the capital of the actual Rio de Janeiro state (the Wikipedia page shows in red the area of the Guanabara state compared to the area of the Rio de Janeiro state: https://pt.wikipedia.org/wiki/Guanabara). In old maps of the Estado da Guanabara it is possible to see a river named Rio Grande (close to Pedra Branca); this is a very common name for rivers in Brazil, and there are probably hundreds of ‘Rio Grande’ rivers in the country. As for Represa, this could be any small dam made in that river (e.g., a farmer could make a small dam to have a lake within his property and people will call it Represa). In conclusion, checking old maps and the location of Pedra Branca, it is correct to say that this location is in the municipality of Rio de Janeiro, probably in Jacarepagua, which is a large neighborhood in the west part of the city. In fact, there is a small neighborhood called Rio Grande very close to the possible collection site, which corroborates the hypothesis that this is the area referred to in the label. Unfortunately, no dam could be located in the area, even though some small lakes or ponds can be seen between Pedra Branca and the Rio Grande neighborhood (e.g., https://maps.app.goo.gl/QifF1ixJY53TwQo19).

#### ﻿ Dolichogenidea evadne

Taxon classificationAnimaliaHymenopteraBraconidae

(Nixon, 1955)

77420E25-9F92-5659-ABC3-3796ACF31D5F

Figs 51A–F , 52A–C

##### Distribution.

Juan Fernández Islands.

**Figure 51.**
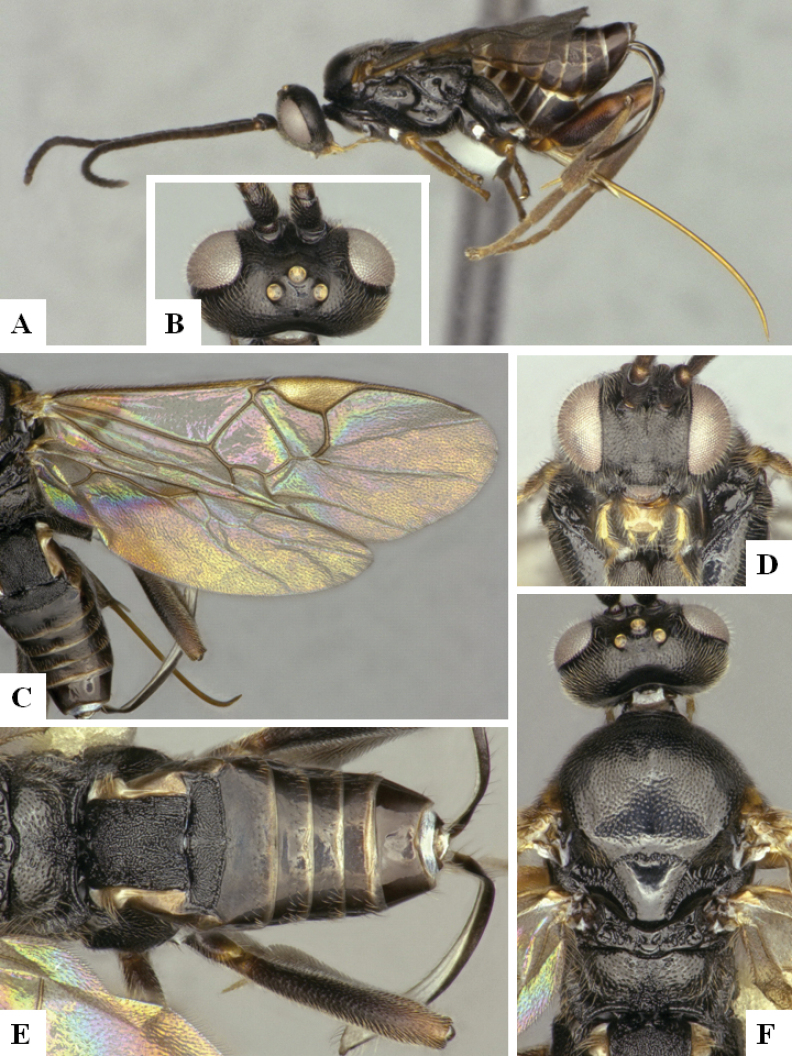
*Dolichogenideaevadne* (Nixon) female CNC1801962 **A** habitus, lateral **B** head, dorsal **C** wings **D** head, frontal **E** metasoma, dorsal **F** mesosoma, dorsal.

**Figure 52.**
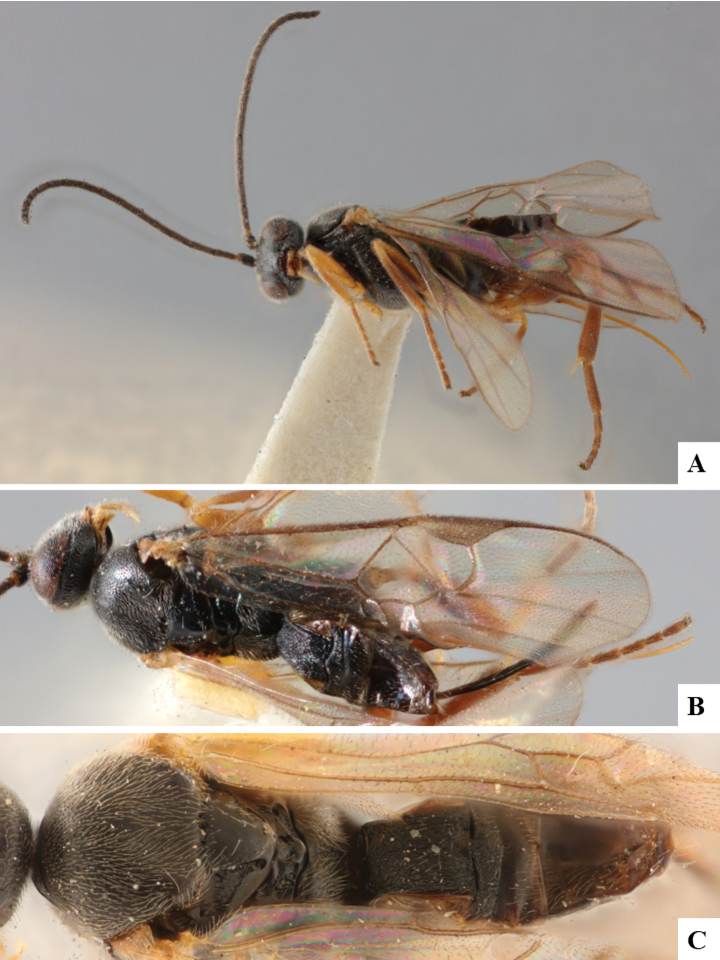
*Dolichogenideaevadne* (Nixon) holotype female **A** habitus, lateral **B** fore wing **C** habitus, dorsal.

##### Biology.

No host data available.

##### DNA barcoding data.

No data.

##### Notes.

This species was described based on two females and one male, collected in two localities of Juan Fernández Islands between December 1951 and February 1952 (Nixon 1955: 164). In the CNC we have seen three specimens from two additional localities, one male collected in February 1973 and two females in January 1992.

#### ﻿ Dolichogenidea felipechavarriai

Taxon classificationAnimaliaHymenopteraBraconidae

Fernandez-Triana & Boudreault sp. nov.

58377254-7FFC-59DA-82EF-24C7CC2D73D3

https://zoobank.org/C26FC9F1-FC46-4E69-BABD-AF1972E83FF8

Figs 53A–F , 54A–F

##### Type material.

***Holotype*.** Costa Rica • Female, CNC; Alajuela, Area de Conservación Guanacaste, sector: Sector San Cristobal, Jardin Estrada; 10.8655, -85.3969; 722 m; 23.xi.2012; Carolina Cano leg.; Host: *Gonionota* Janzen116; Voucher code: DHJPAR0051066; Host voucher code: 12-SRNP-5125. ***Paratypes*.** Costa Rica • 4 Females, CNC; DHJPAR0050163, DHJPAR0054786, DHJPAR0054816, DHJPAR0054819.

**Figure 53.**
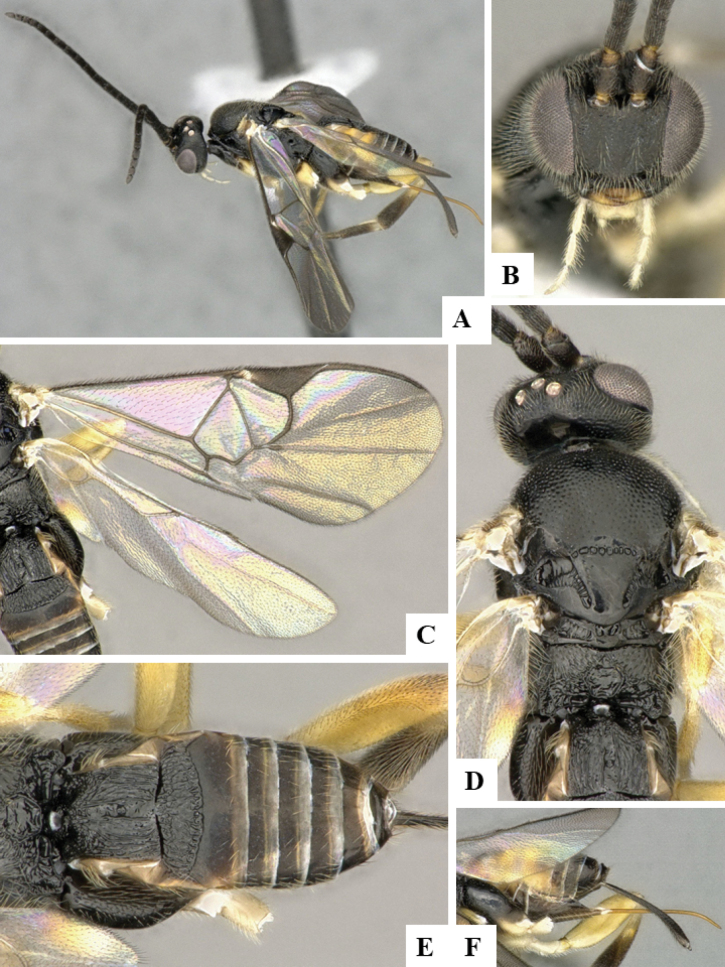
*Dolichogenideafelipechavarriai* Fernandez-Triana & Boudreault holotype female DHJPAR0051066 **A** habitus, lateral **B** head, frontal **C** wings **D** mesosoma, dorsal **E** metasoma, dorsal **F** ovipositor, lateral.

**Figure 54.**
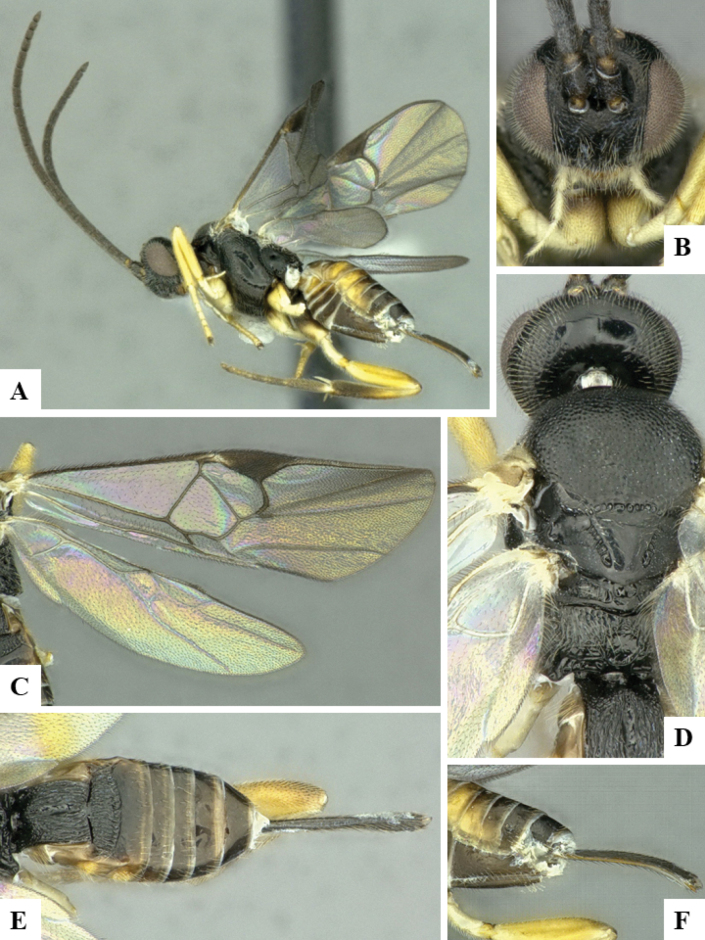
*Dolichogenideafelipechavarriai* Fernandez-Triana & Boudreault paratype female DHJPAR0054786 **A** habitus, lateral **B** head, frontal **C** wings **D** mesosoma, dorsal **E** metasoma, dorsal **F** ovipositor, lateral.

##### Diagnostic description.

F15 length 1.4–1.5× its height; T1 and T2 heavily sculptured with strong longitudinal striae; T1 length < 1.5× T1 width at posterior margin; T2 broadly rectangular (but posterior margin sinuate), covering most surface of tergum; tegula white-yellow, clearly paler than brown humeral complex; pterostigma with very small paler spot on anterior 0.1 or less; pro- and mesocoxae yellow; metafemur entirely yellow; metatibia dark brown to black on posterior 0.7; laterotergites 1–4 and at least sternites 1–2 entirely to mostly yellow; body length: 2.53–2.97 mm; fore wing length: 2.72–3.05 mm. Among all species with heavily sculptured T1 and T2, T1 comparatively broad and T2 rectangular, *D.felipechavarriai* can be distinguished by the shape of F15 and color of tegula, humeral complex, pterostigma, procoxa, mesocoxa, metafemur and metatibia, laterotergites and sternites. The closest species, *D.chichicastenango* from Guatemala, has darker coloration of legs and metasoma, and comparatively shorter F15.

##### Distribution.

Costa Rica.

##### Biology.

Solitary. Depressariidae, *Gonionota* Janzen116.

##### DNA barcoding data.

BINBOLD:ACC4119 (6 sequences, 6 barcode compliant).

##### Etymology.

Named in honor of Sr. Luis Felipe Chavarria of Area de Conservación Guanacaste in northwestern Costa Rica, for his outstanding performance of managing the on-site financial administration for the Guanacaste Dry Forest Conservation Fund and simultaneously photo-documenting a major portion of the landscape and vertebrates.

#### ﻿ Dolichogenidea frankjoycei

Taxon classificationAnimaliaHymenopteraBraconidae

Fernandez-Triana & Boudreault sp. nov.

F8725B41-C9C6-5954-847D-1D6CD7C28C48

https://zoobank.org/5C8BDA51-C3BE-416A-8B76-244D0AB19724

Fig. 55A–E

##### Type material.

***Holotype*.** Costa Rica • Female, CNC; Alajuela, Area de Conservación Guanacaste, Sector Rincón Rain Forest, Sendero Tucán; 10.9042, -85.2712; 410 m; 5.iii.2012; Anabelle Cordoba leg.; Host: *Platynota* rostranaDHJ02; Voucher code: DHJPAR0049373; Host voucher code: 12-SRNP-40934.

**Figure 55.**
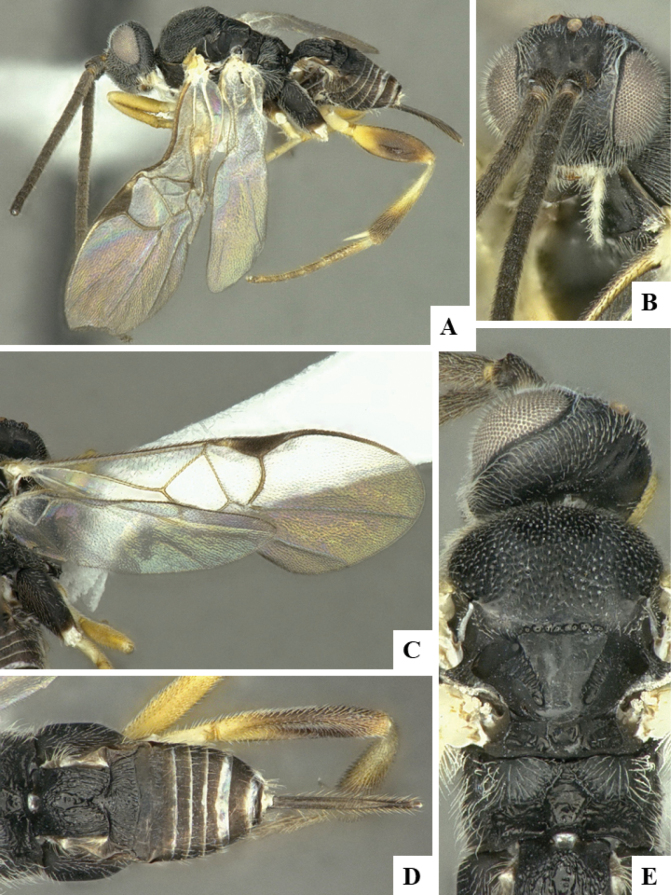
*Dolichogenideafrankjoycei* Fernandez-Triana & Boudreault holotype female DHJPAR0049373 **A** habitus, lateral **B** head, frontal **C** wings **D** metasoma, dorsal **E** mesosoma, dorsal.

##### Diagnostic description.

F15 1.4× as long as wide; T1 and T2 heavily sculptured with strong longitudinal striae; T1 length < 1.5× T1 width at posterior margin; T2 rectangular, covering most surface of tergum; ovipositor sheath clearly shorter than metatibia length (0.80×); metafemur mostly brown; metatibia dark brown to black on posterior 0.5; body length: 2.50 mm; fore wing length: 2.60 mm. Among all species with heavily sculptured T1 and T2, T1 comparatively broad and T2 rectangular, *D.frankjoycei* can be distinguished by the color of metafemur and metatibia, and length of F15. The only species closely similar morphologically is *D.rociocordobae* which has slightly longer ovipositor sheath and slightly shorter F15, as well as different hosts and DNA barcode.

##### Distribution.

Costa Rica.

##### Biology.

Solitary. Tortricidae, *Platynota* rostranaDHJ01, *Platynota* rostranaDHJ02.

##### DNA barcoding data.

BINBOLD:ABA3469 (7 sequences, 5 barcode compliant).

##### Etymology.

Named in honor of Dr. Frank Joyce of Monteverde, Cuajiniquil and ACG in recognition of his decades of support for the biodiversity conservation efforts by Area de Conservación Guanacaste and the NGO Guanacaste Dry Forest Conservation Fund in northwestern Costa Rica.

#### ﻿ Dolichogenidea fredhicksi

Taxon classificationAnimaliaHymenopteraBraconidae

Fernandez-Triana & Boudreault sp. nov.

A7610010-D31A-59A0-A800-A65872296921

https://zoobank.org/83E3A909-4282-46A5-9606-18CECC3B8235

**Figure 56.**
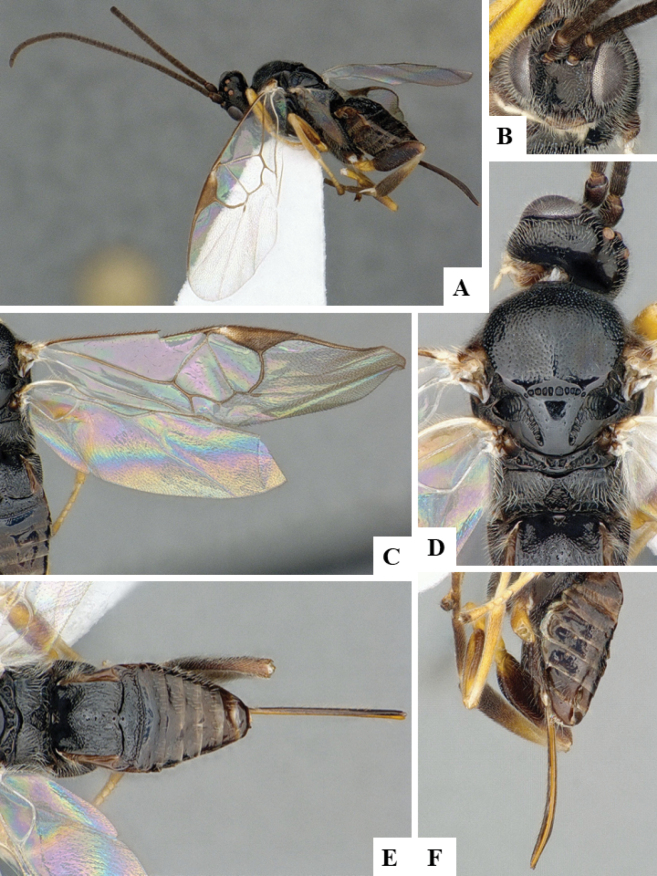
*Dolichogenideafredhicksi* Fernandez-Triana & Boudreault holotype female DHJPAR0005166 **A** habitus, lateral **B** head, frontal **C** wings **D** mesosoma, dorsal **E** metasoma, dorsal **F** ovipositor, lateral.

**Figure 57.**
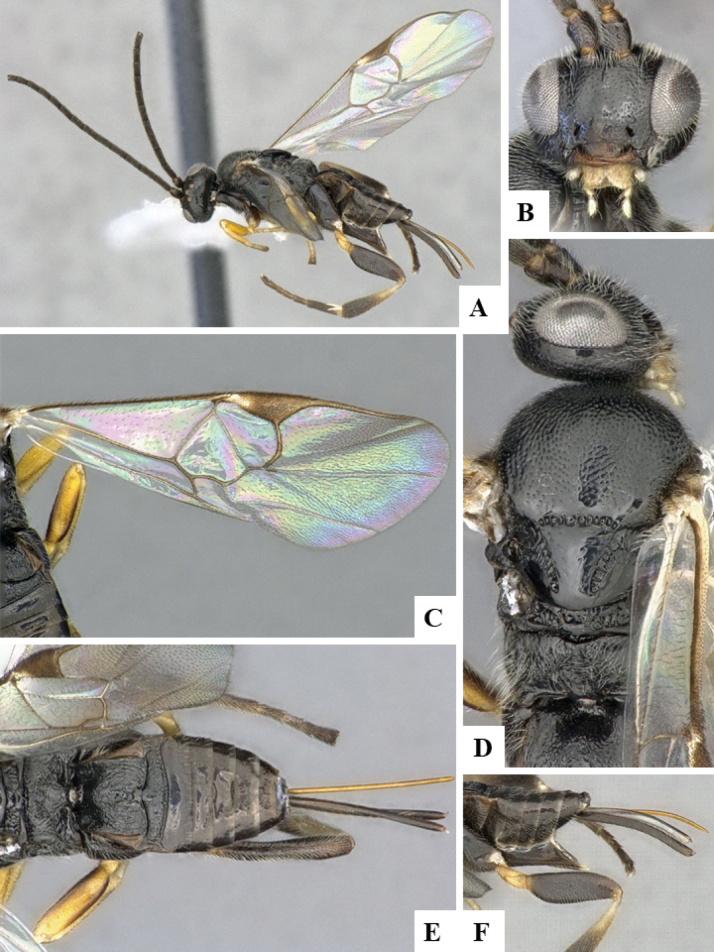
*Dolichogenideafredhicksi* Fernandez-Triana & Boudreault paratype female DHJPAR0005171 **A** habitus, lateral **B** head, frontal **C** fore wing **D** mesosoma, dorsal **E** metasoma, dorsal **F** ovipositor, lateral.

Figs 56A–F , 57A–F , 156B

##### Type material.

***Holotype*.** Costa Rica • Female, CNC; Guanacaste, Area de Conservación Guanacaste, Sector San Cristobal, Quebrada Cementerio; 10.87124, -85.38749; 700 m; 10.x.1998; Gloria Sihezar leg.; Host: *Stenoma* Janzen27; Voucher code: DHJPAR0005166; Host voucher code: 98-SRNP-14538. ***Paratype*.** Costa Rica • 1 Female, CNC; DHJPAR0005171.

##### Diagnostic description.

Posterior 0.5 of propodeum (beyond transverse carinae of areola) mostly striated; posterior 0.5–0.6 of T1 and T2 mostly with strong sculpture, usually longitudinal striae covering entire surface (but T2 with small polished area centrally); T1 broadening posteriorly; T2 comparatively very transverse but with anterior margin arcuate; ovipositor sheath clearly longer (1.15–1.25×) than metatibia length; tegula brown; fore wing venation mostly pale brown to yellow-brown; pterostigma with comparatively large bright yellow-white spot on anterior 0.2 which is clearly defined; pro- and mesocoxae brown, metacoxa dark brown to black; metafemur dark brown; metatibia brown on posterior 0.5; body length: 3.03–3.16 mm; fore wing length: 3.43–3.50 mm. This species has strong sculpture (usually longitudinal striae) covering posterior 0.5–0.6 of T1 and most of T2. However, unlike the majority of species with similarly strong sculpture, T2 has a central area which is smooth and also T2 is very transverse and with anterior margin strongly arcuate. Because of that unique shape and sculpture pattern of T2, as well as its metafemur color and ovipositor sheath length, it can be separated from all the species with entirely and strongly sculptured T2 which is not transverse, as well as all the species with smooth T2 and/or broad T2.

##### Distribution.

Costa Rica.

##### Biology.

Gregarious. Depressariidae: *Anadasmus* Janzen25, *Stenoma* Janzen27.

##### DNA barcoding data.

BINBOLD:AAK2061 (2 sequences, 2 barcode compliant).

##### Etymology.

Named in honor of Mr. Fred Hicks of Costa Rica in recognition of his recent efforts to support Area de Conservación Guanacaste biodiversity through facilitating neighboring plantation reforestation as a business venture that will also be invaded by wild ACG biodiversity.

#### ﻿ Dolichogenidea gelechiidivoris

Taxon classificationAnimaliaHymenopteraBraconidae

(Marsh, 1975)

A6E74B42-B3B7-5B91-B569-4ADF718DE113

Fig. 58A–G

##### Notes.

Full details for this species in Krache et al. (2021), including a complete morphological and molecular characterization.

**Figure 58.**
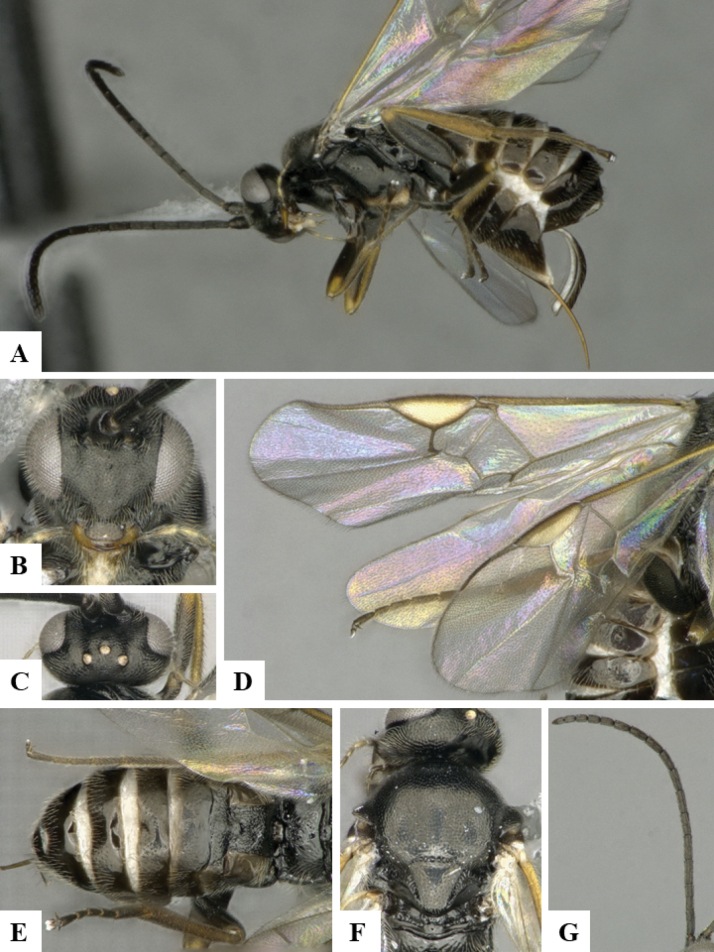
*Dolichogenideagelechiidivoris* (Marsh) female CNC1196542 **A** habitus, lateral **B** head, frontal **C** head, dorsal **D** wings **E** metasoma, dorsal **F** mesosoma, dorsal **G** antenna.

#### ﻿ Dolichogenidea genuarnunezi

Taxon classificationAnimaliaHymenopteraBraconidae

Fernandez-Triana & Boudreault, 2019

B7A7B619-5656-5FC1-8BC5-2327F1905A8C

Fig. 59A–F

##### Notes.

Full details for this species in Fernandez-Triana et al. (2019). See also the key and Table 1 above.

**Figure 59.**
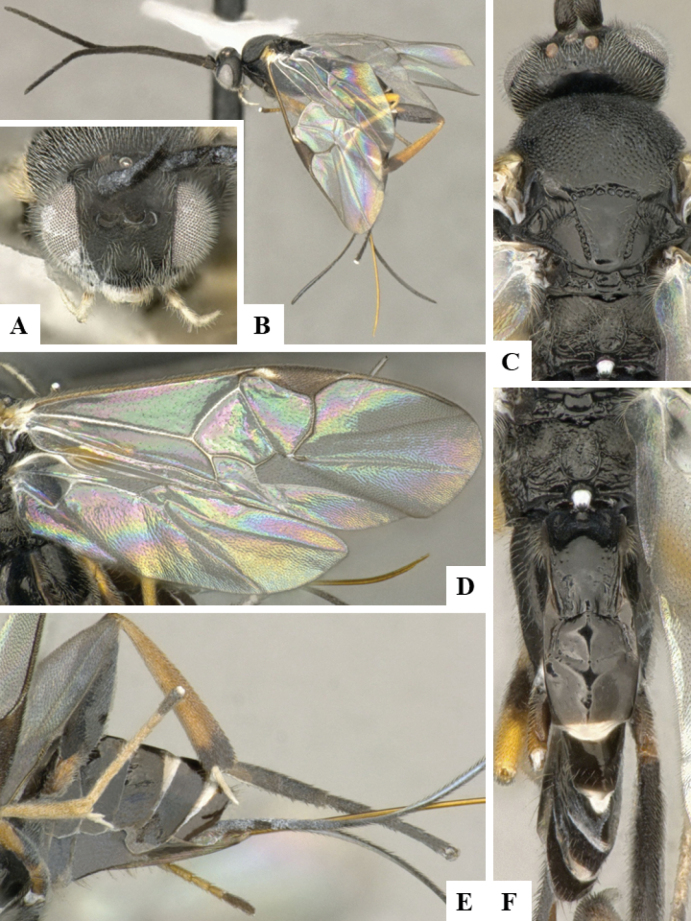
*Dolichogenideagenuarnunezi* Fernandez-Triana & Boudreault holotype female DHJPAR0050092 **A** head, frontal **B** habitus, lateral **C** mesosoma, dorsal **D** wings **E** ovipositor, lateral **F** metasoma, dorsal.

#### ﻿ Dolichogenidea hedyleptae

Taxon classificationAnimaliaHymenopteraBraconidae

(Muesebeck, 1958)

B4BA3F6A-5371-5640-8ABF-2D1E697A7EEC

Fig. 60A–C

##### Distribution.

Puerto Rico.

**Figure 60.**
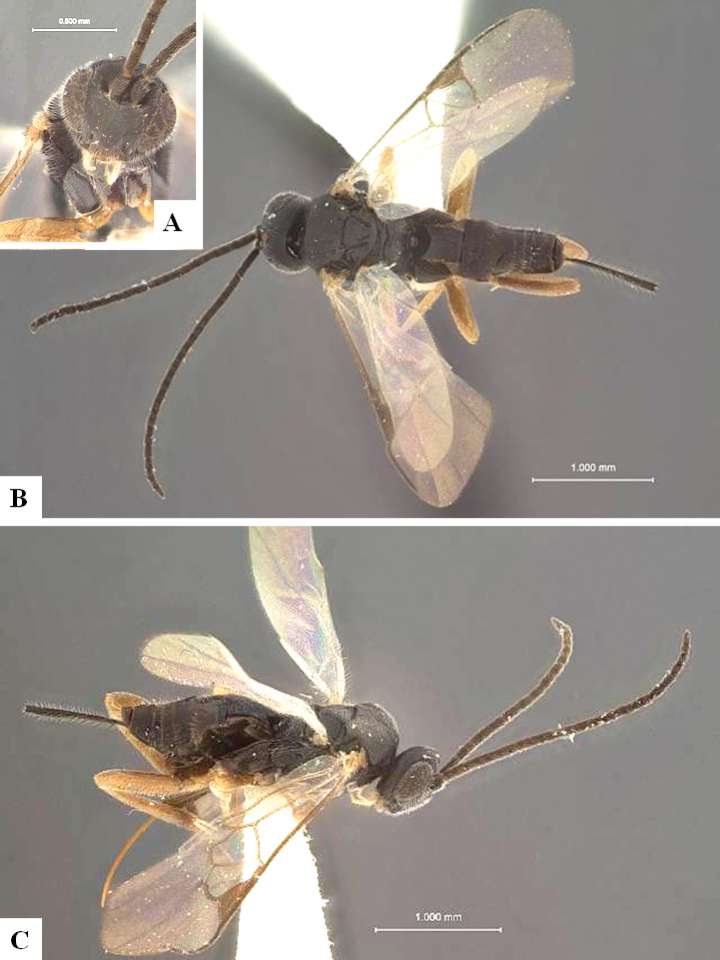
*Dolichogenideahedyleptae* (Muesebeck) holotype female **A** head, frontal **B** habitus, dorsal **C** habitus, lateral.

##### Biology.

Probably gregarious. Pyralidae: *Marucavitrata*, *Omiodesindicata*.

##### Notes.

See comments under the newly described species *D.oiketicus* for differences between these two species.

#### ﻿ Dolichogenidea helenedumasae

Taxon classificationAnimaliaHymenopteraBraconidae

Fernandez-Triana & Boudreault sp. nov.

DDC02801-DF07-5E50-8F0C-38320A79AC06

https://zoobank.org/F0627619-4743-41AA-BAE0-0D6707C803E3

Fig. 61A–H

##### Type material.

***Holotype*.** French Guiana • Female, CNC; Montagne de Kaw, Relais Patawa; ii.1999; A.E.I. Guyane-J. Cerda leg.; Malaise trap; Voucher code: CNC491966. ***Paratypes*.** Brazil • 2 Females, CNC; CNCHYM 00125, CNCHYM 00126. French Guiana • 1 Female, CCDB; CCDB-07375 B04.

**Figure 61.**
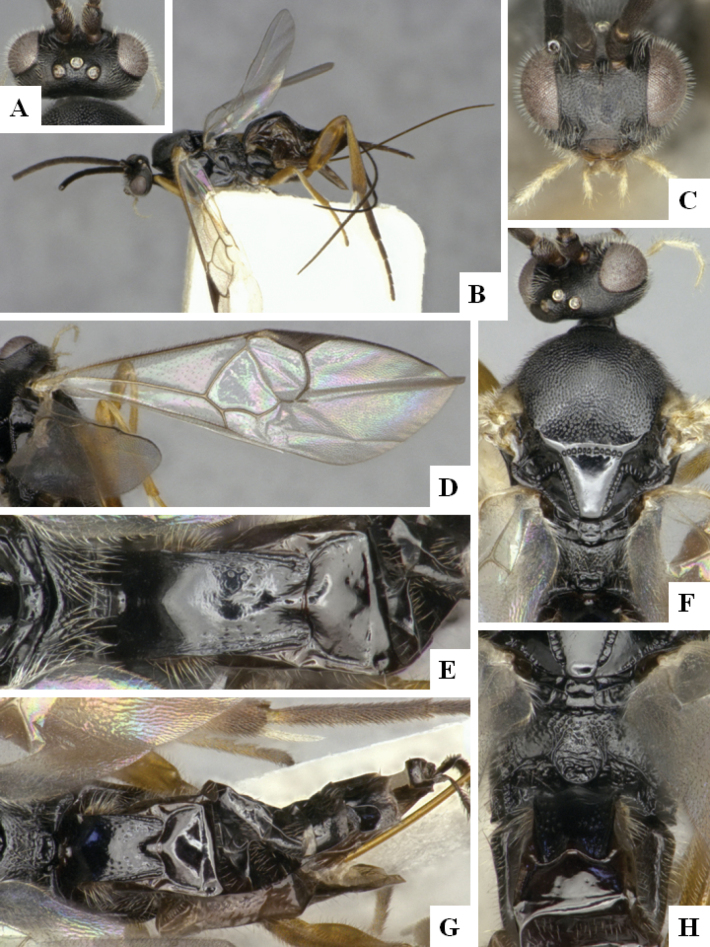
*Dolichogenideahelenedumasae* Fernandez-Triana & Boudreault holotype female CNC491966 **A** head, dorsal **B** habitus, lateral **C** head, frontal **D** wings **E** T1–T2, dorsal **F** mesosoma, dorsal **G** metasoma, dorsal **H** propodeum & T1–T2, dorsal.

##### Diagnostic description.

T1 almost entirely smooth (only weak punctures near posterior margin); T1 more or less parallel-sided; T2 entirely smooth and shiny; T2 sub-quadrate, its width at posterior margin < 1.9× its length centrally; ovipositor sheath 2.0× as long as metatibia length; metatrochanter black, metatrochantellus yellow-orange; mesofemur and metafemur entirely yellow; body length: 4.10–4.20 mm; fore wing length: 4.20–4.30 mm; ovipositor sheath length: 3.00–3.20 mm. Among all species within the *carlosmanuelrodriguezi* group, it can be recognized by the combination of its body size and mesofemur and metafemur coloration, as well as distinctive DNA barcodes.

##### Distribution.

Brazil (MG), French Guiana.

##### Biology.

No host data available.

##### DNA barcoding data.

BINBOLD:ABZ4155 (15 sequences, 15 barcode compliant), but see notes below. The paratype from French Guiana has a full barcode, the two Brazil paratypes have partial sequences (363 and 164 bp).

##### Etymology.

Named after Hélène Dumas (la Ciotat, France), in recognition for her efforts filming insects, especially Microgastrinae wasps in France. Hélène is the daughter of Frédéric Dumas, who was a member of the commander Jacques-Yves Cousteau’s team.

##### Notes.

The material we have studied included three specimens deposited in the CNC, one from French Guiana (holotype) and two paratypes from Brazil. They match very closely with the photo in BOLD of a different barcoded specimen from French Guiana, deposited in the CCDB and collected in a locality close to that of the holotype. Although we have not been able to examine that specimen, its body size (as indicated in the scale bar of the photo in BOLD), matches closely with the CNC specimens. Body length is a relevant and diagnostic feature for this new species; based on that we consider all those specimens (from French Guiana and Brazil) to be conspecific. The full sequence of the CCDB specimen corresponds to the same BIN than that of the species *D.carlosmanuelrodriguezi*, from Costa Rica. However, that BIN contains more than one species, as mentioned in the original description of *D.carlosmanuelrodriguezi* (Fernandez-Triana et al. 2019: 100). Those authors indicated that, in addition to *D.carlosmanuelrodriguezi*, there were four other undescribed species from Costa Rica (ACG), and here we account for an additional one from South America. The full barcode sequence of *D.helenedumasae* (paratype from French Guiana) is > 1.3% bp different from the closest ACG species (which remains undescribed and with interim name of *Dolichogenidea* Janzen156 in BOLD), and the other ACG species within this BIN are farther apart. We consider the molecular differences and the body size difference as sufficient to recognize *D.helenedumasae* as a distinct species from the ACG species.

#### ﻿ Dolichogenidea heredia

Taxon classificationAnimaliaHymenopteraBraconidae

Fernandez-Triana & Boudreault sp. nov.

D7900FCB-6908-5D2D-8008-D7F761AA8D32

https://zoobank.org/0AC05C0F-46D4-4B17-912D-CF7D887B0E35

Fig. 62A–G

##### Type material.

***Holotype*.** Costa Rica • Female, CNC; Heredia; 10°17'N, 84°10'W; 1,400 m; J. Helava leg.; Voucher code: CNC1180108.

**Figure 62.**
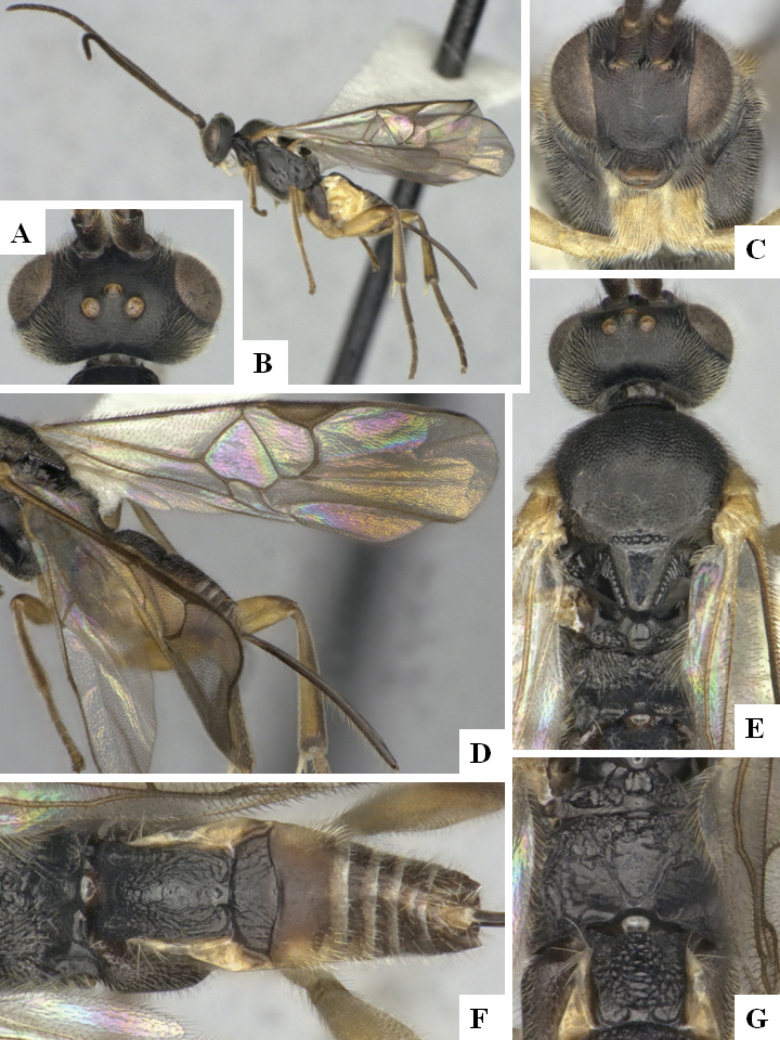
*Dolichogenideaheredia* Fernandez-Triana & Boudreault holotype female CNC1180108 **A** head, dorsal **B** habitus, lateral **C** head, frontal **D** wings **E** mesosoma, dorsal **F** metasoma, dorsal **G** propodeum & T1, dorsal.

##### Diagnostic description.

Hind legs tarsal claws simple; T1 and T2 heavily sculptured with strong longitudinal striae (but T2 striae sparser, with small smoother area centrally); T1 more or less parallel-sided but comparatively narrow, ~ 2.0× as long as wide at posterior margin; T2 comparatively broad, its width at posterior margin ~ 2.5× its length medially; ovipositor sheath length 1.3× metatibia length; pterostigma mostly pale yellow-brown but with thin brown margins; metacoxa dark brown on anterior 0.7 and yellow on posterior 0.3; metatibia with dorsal dark brown band on entire length of metatibia; body length: 3.25 mm; fore wing length: 3.41 mm. Among all species with heavily sculptured T1 and T2, this species is distinctive by its T2 slightly less sculptured (especially centrally), metacoxa and metatibia color, ovipositor sheath length and pterostigma mostly pale colored but with thin brown margins.

##### Distribution.

Costa Rica.

##### Biology.

No host data available.

##### DNA barcoding data.

No data.

##### Etymology.

Named after the type locality.

#### ﻿ Dolichogenidea homoeosomae

Taxon classificationAnimaliaHymenopteraBraconidae

(Muesebeck, 1933)

FFD81F75-3279-5FCB-B9A6-E174ECD67184

Figs 63A–G , 64A–C

##### Distribution.

Canada (SK), Cuba, United States (CA, MS, MO, SD, TX, WA).

**Figure 63.**
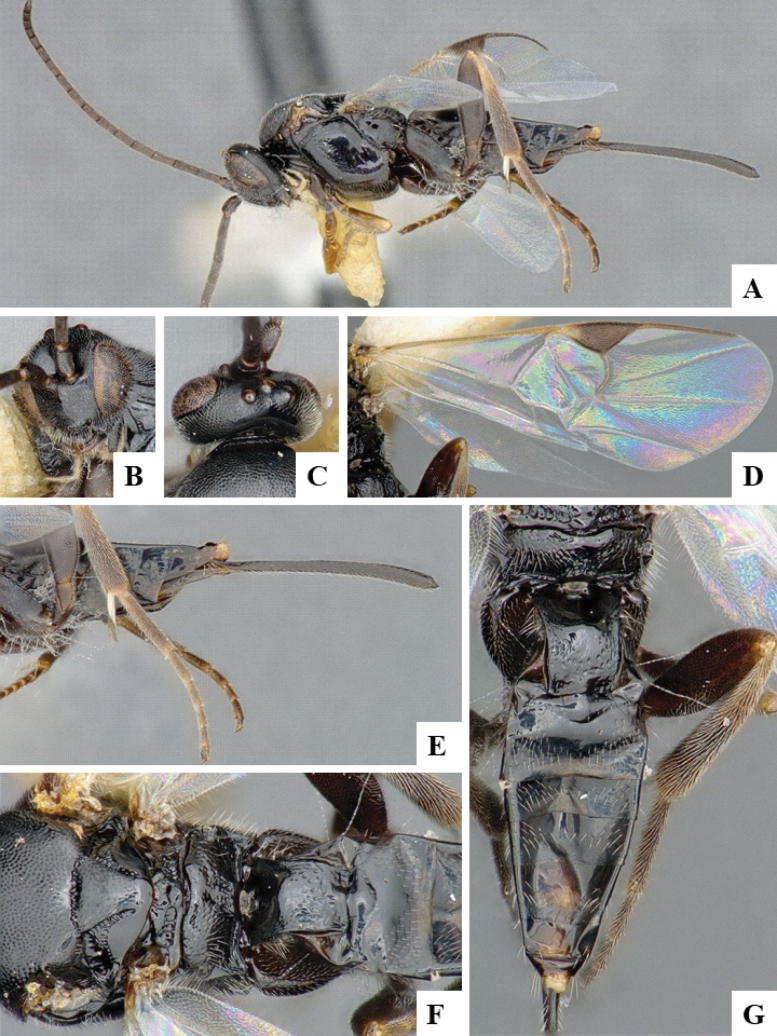
*Dolichogenideahomoeosomae* (Muesebeck) female CNCHYM01051 **A** habitus, lateral **B** head, frontal **C** head, dorso-lateral **D** wings **E** ovipositor, lateral **F** mesosoma, dorsal **G** metasoma, dorsal.

**Figure 64.**
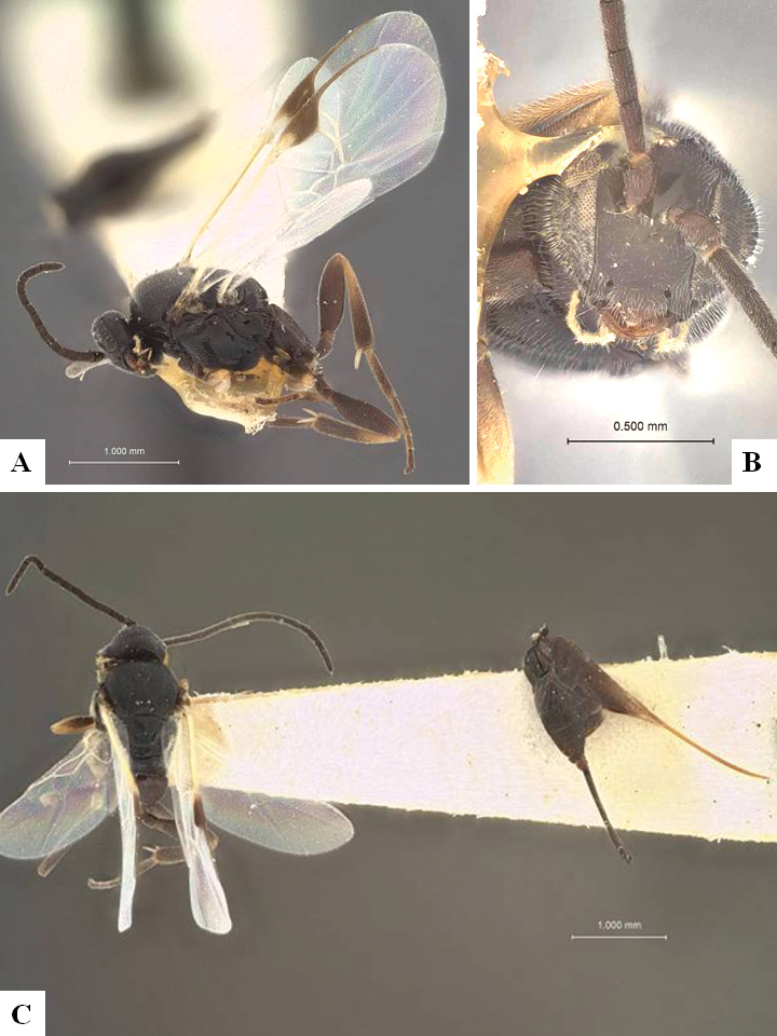
*Dolichogenideahomoeosomae* (Muesebeck) holotype female **A** habitus, lateral **B** head, frontal **C** mesosoma, dorsal & metasoma, lateral.

##### Biology.

Solitary. Pyralidae: *Homoeosomaelectellum*.

##### DNA barcoding data.

Partial sequences available, including two 425 bp long (voucher codes: CNCHYM 01050, CNCHYM 01052).

#### ﻿ Dolichogenidea ingredolsonae

Taxon classificationAnimaliaHymenopteraBraconidae

Fernandez-Triana & Boudreault sp. nov.

0C4F2A6F-8E56-5AE3-8695-4C2A2AEC1A07

https://zoobank.org/5F1E8C82-5488-461B-8AF0-5BFD50415870

Fig. 65A–F

##### Type material.

***Holotype*.** Costa Rica • Female, CNC; Guanacaste, Area de Conservación Guanacaste, Sector Santa Rosa, Area Administrativa; 10.83764, -85.61871; 295 m; 25.xii.2008; D. H. Janzen & W. Hallwachs leg.; Malaise trap; Voucher code: DHJPAR0031845.

**Figure 65.**
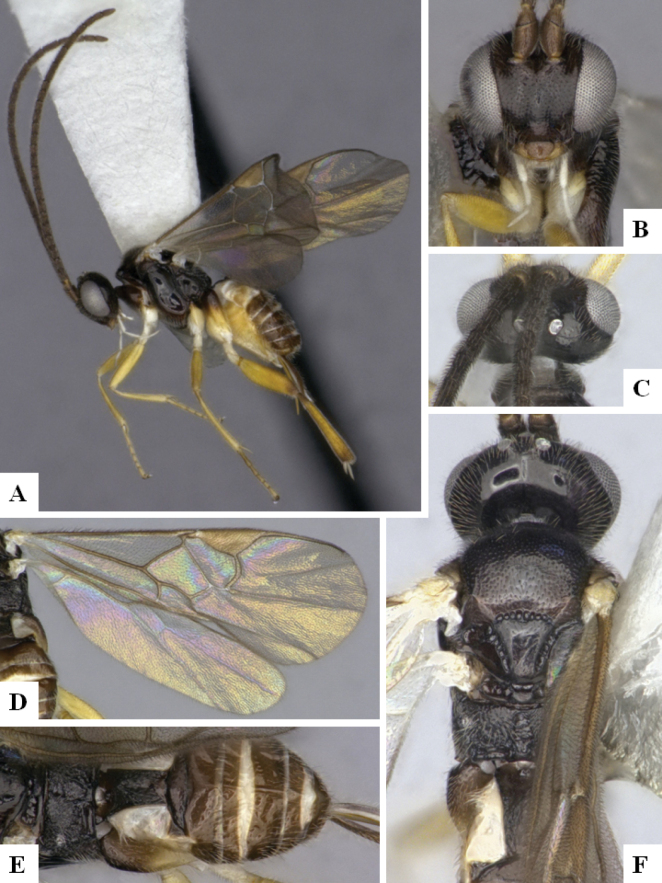
*Dolichogenideaingredolsonae* Fernandez-Triana & Boudreault holotype female DHJPAR0031845 **A** habitus, lateral **B** head, frontal **C** head, dorsal **D** wings **E** metasoma, dorsal **F** mesosoma, dorsal.

##### Diagnostic description.

T1 strongly narrowing towards posterior margin, its length medially ~ 4.0–5.0× its width at posterior margin and its width at anterior margin 2.0× its width at posterior margin; T2 smooth; ovipositor sheath shorter than metatibia length (0.7×); tegula brown, darker than yellow humeral complex; legs mostly palely colored (including pro- and mesocoxae entirely white-yellow, metacoxa with apical 0.6 yellow, metafemur and metatibia mostly yellow, and metatibial spurs yellow); body length: 2.30 mm; fore wing length: 2.63 mm. The color of tegula, humeral complex and legs, as well as the shape of T1 distinguish this species among most species with smooth T2 and pale pro- and mesocoxae. *Dolichogenideajunhyongkimi* is relatively similar morphologically but it has T1 more strongly narrowing, longer ovipositor sheath and different color of tegula.

##### Distribution.

Costa Rica.

##### Biology.

No host data available.

##### DNA barcoding data.

BINBOLD:AAM5850 (1 sequence, 1 barcode compliant).

##### Etymology.

Named in honor of Dr. Ingred Olson in recognition of her intensely supportive role in the family of Dr. Junhyong Kim during his outstanding yet especially stressful 5-year term as Department Chairman of the Biology Department of the University of Pennsylvania, Philadelphia, Pennsylvania, USA, during these years of COVID and university turmoil.

#### ﻿ Dolichogenidea isabelleae

Taxon classificationAnimaliaHymenopteraBraconidae

Fernandez-Triana & Boudreault sp. nov.

85765775-7323-5F37-9FA1-76E5F4EF7A15

https://zoobank.org/EDD74ECC-DB3C-49BF-AC37-A76DA0B4DA0E

Fig. 66A–F

##### Type material.

***Holotype*.** Ecuador • Female, CNC; Pichincha, Santo Domingo, 47 km S of Rio Palenque; 200 m; 15.vii.1976; S. Peck leg.; Voucher code: CNC1179687.

**Figure 66.**
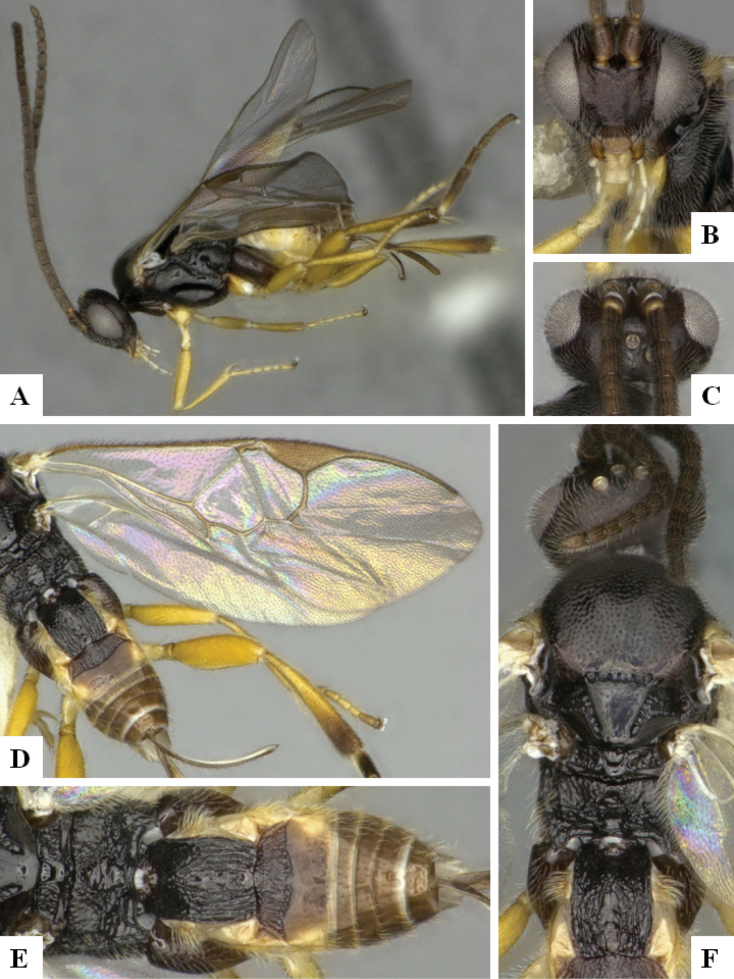
*Dolichogenideaisabelleae* Fernandez-Triana & Boudreault holotype female CNC1179687 **A** habitus, lateral **B** head, frontal **C** head, dorsal **D** wings **E** metasoma, dorsal **F** mesosoma, dorsal.

##### Diagnostic description.

F15 1.3× as long as high; scutellar disc smooth; anterior half of mesopleuron smooth, anteromesoscutum with sparse and relatively shallow punctures; propodeum with carinae defining areola strongly risen and sharp; T1 and T2 heavily sculptured with strong longitudinal striae (rarely strong reticulated sculpture) covering entire surface of T2 and most of T1; T1 comparatively thinner, mostly parallel-sided but posterior 0.1–0.3 slightly narrowing towards posterior margin; tegula white-yellow, humeral complex mostly brown; pterostigma mostly pale brown with small, paler spot anteriorly; pro- and mesocoxae entirely yellow, metacoxa reddish brown; metatibia yellow at least on anterior 0.4; T3 with yellow spots laterally, centrally pale brown, T4+ pale brown to brown; laterotergites 1–5 yellow; most sternites at least partially yellow; hypopygium partially yellow and partially pale brown; body length: 2.70 mm; fore wing length: 2.80 mm. Among all species with heavily sculptured T1 and T2, this species is distinctive by its coloration, especially metasoma, legs and pterostigma, as well as scutellar disc sculpture and propodeum strongly carinate.

##### Distribution.

Ecuador.

##### Biology.

No host data available.

##### DNA barcoding data.

No data.

##### Etymology.

The second author dedicates this species in honor of her very close friend Isabelle Guindon. Isabelle has been an inspiration by her aliveness, contagious happiness, and true enthusiasm; and also, for all the fun we have together!

#### ﻿ Dolichogenidea isidrochaconi

Taxon classificationAnimaliaHymenopteraBraconidae

Fernandez-Triana & Boudreault sp. nov.

33FE7828-2CBE-573D-BECC-EAAE00EBE93D

https://zoobank.org/CBAE4A47-9448-4B73-ADD6-E5BA1F65E018

Fig. 67A–F

##### Type material.

***Holotype*.** Costa Rica • Female, CNC; Guanacaste, Area de Conservación Guanacaste, Sector Santa Rosa, Area Administrativa; 10.83764, -85.61871; 295 m; 25.xii.2008; D. H. Janzen & W. Hallwachs leg.; Malaise trap; Voucher code: DHJPAR0031750. ***Paratypes*.** Costa Rica • 9 Females, CNC; DHJPAR0031600, DHJPAR0031694, DHJPAR0031695, DHJPAR0031706, DHJPAR0031710, DHJPAR0031739, DHJPAR0031750, DHJPAR0031755, DHJPAR0031809.

**Figure 67.**
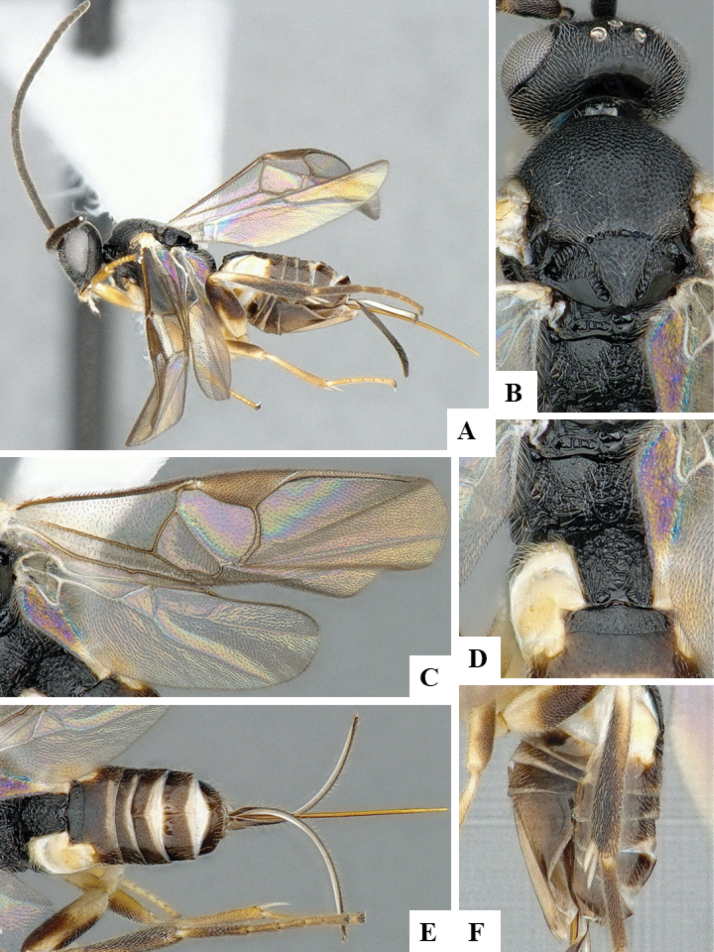
*Dolichogenideaisidrochaconi* Fernandez-Triana & Boudreault holotype female DHJPAR0031750 **A** habitus, lateral **B** mesosoma, dorsal **C** wings **D** propodeum & T1–T3, dorsal **E** metasoma, dorsal **F** metasoma, lateral.

##### Diagnostic description.

T1 mostly parallel-sided, its length medially ~ 2.5× its width at posterior margin; T2 transverse, its length medially 4.0–5.0× its width at posterior margin; T1 mostly sculptured on posterior 0.5; T2 mostly sculptured, central smooth area very small; tegula white-yellow, clearly paler in color than yellow humeral complex; pro- and mesocoxae yellow-white or yellow; metacoxa mostly yellow with dark brown spot on anterior 0.3; most of metafemur and metatibia dark brown to black; body length and fore wing length: 2.63–2.85 mm. Among all species with T2 at least partially smooth and pro- and mesocoxae pale, *D.isidrochaconi* can be distinguished by T1 shape, T2 shape and sculpture, and color of tegula, humeral complex, coxae, mesofemur and metatibia. Two species (*D.jennyphillipsae* and *D.robertofernandezi*) are very similar morphologically to *D.isidrochaconi* and can only be reliably separated by DNA barcodes (see comments and details under the diagnostic description of *D.jennyphillipsae*).

##### Distribution.

Costa Rica.

##### Biology.

No host data available.

##### DNA barcoding data.

BINBOLD: BOLD:AAB9372 (48 sequences, 37 barcode compliant).

##### Etymology.

Named in honor of Sr. Isidro Chacon of Boconera, Costa Rica, and the Costa Rican National Museum, BioAlfa and the former INBio (Instituto Nacional de Biodiversity) in recognition of his four decades dedicated to the biodiversity understanding of the Lepidoptera of Costa Rica.

#### ﻿ Dolichogenidea jaimelewisi

Taxon classificationAnimaliaHymenopteraBraconidae

Fernandez-Triana & Boudreault sp. nov.

2D3149D5-D912-5522-866F-BC7FBAD82DAD

https://zoobank.org/F544C439-578B-4EB2-931E-5342EFAF1EE2

Figs 68A–F , 69A-F

##### Type material.

***Holotype*.** Costa Rica • Female, CNC; Guanacaste, Area de Conservación Guanacaste, Sector Cacao, Sendero Nayo; 10.92446, -85.46953; 1,090 m; 13.viii.2010; Manuel Pereira leg.; Host: *Herpetogramma* Dapkey27; Voucher code: DHJPAR0040384; Host voucher code: 10-SRNP-35722. ***Paratypes*.** Costa Rica • 2 Females, CNC; DHJPAR0031233, DHJPAR0040398.

**Figure 68.**
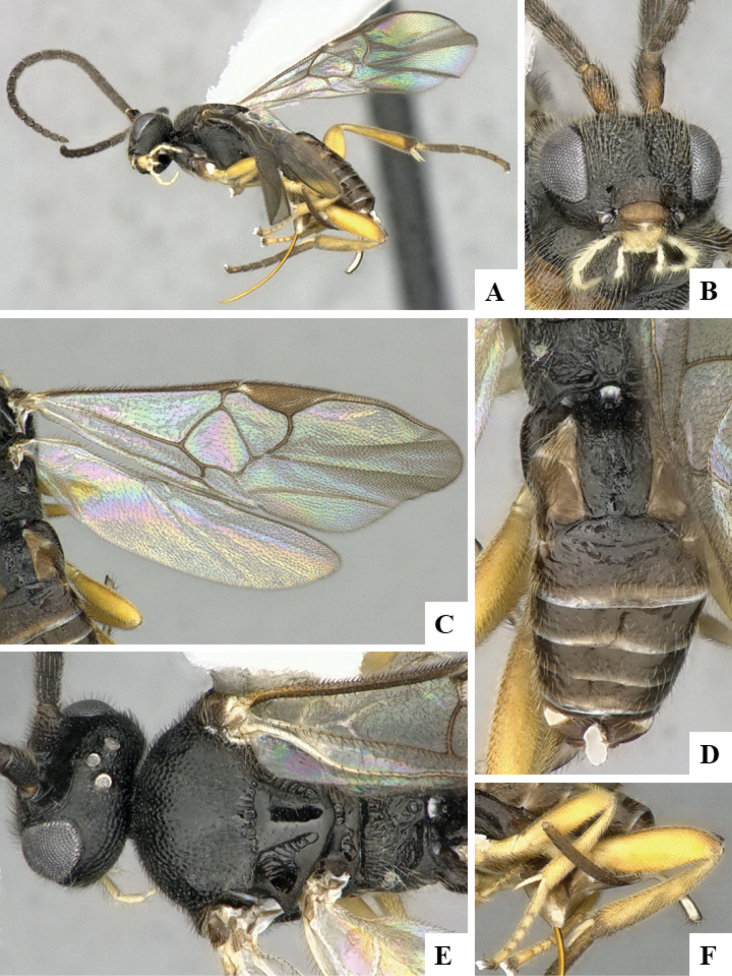
*Dolichogenideajaimelewisi* Fernandez-Triana & Boudreault holotype female DHJPAR0040384 **A** habitus, lateral **B** head, frontal **C** wings **D** metasoma, dorsal **E** mesosoma, dorsal **F** metasoma, lateral.

**Figure 69.**
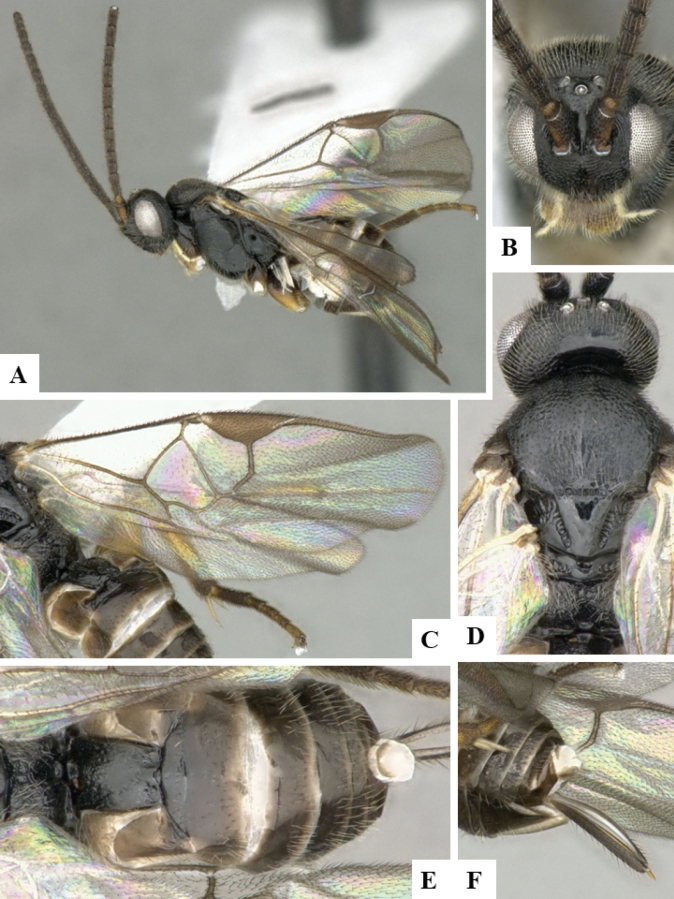
*Dolichogenideajaimelewisi* Fernandez-Triana & Boudreault paratype female DHJPAR0031233 **A** habitus, lateral **B** head, frontal **C** wings **D** mesosoma, dorsal **E** metasoma, dorsal **F** ovipositor, lateral.

##### Diagnostic description.

Anteromesoscutum punctures near end of notauli fused, unlike punctures on rest of anteromesoscutum; T1 mostly parallel-sided but posterior 0.3 slightly narrowing towards posterior margin; T1 mostly sculptured on posterior 0.5; T2 smooth; T2 transverse and comparatively narrow, its width at posterior margin > 3.0× its length medially; scape ventrally yellow-brown, distinctly paler colored than dorsal side; tegula brown, same color than humeral complex; pro- and mesocoxae partially pale brown partially yellow; metacoxa mostly dark brown to black but with posterior 0.1–0.2 yellow; metafemur yellow; metatibia mostly yellow, with posterior 0.1–0.2 brown; body length: 2.81–2.88 mm; fore wing length: 2.94–3.03 mm. Among all species with T2 smooth and dark coxae *Dolichogenideajaimelewisi* can be distinguished by T1 sculpture, T2 shape, anteromesoscutum punctures, and scape, tegula and leg color. Another species, *D.juanmatai*, is similar morphologically but has different coloration of scape, tegula and legs, as well as different puncture sculpture on anteromesoscutum.

##### Distribution.

Costa Rica.

##### Biology.

Gregarious. Crambidae: *Herpetogrammasalbialis*, *Herpetogramma* Dapkey27, unidentified lepidopteran with provisional name ‘spiloBioLep01 BioLep617’.

##### DNA barcoding data.

BINBOLD:AAM5738 (6 sequences, 6 barcode compliant).

##### Etymology.

Named in honor of Mr. Jaime Lewis of San Jose, and a volunteer taxonomist for Hemiptera in the Costa Rican National Museum, BioAlfa and the former INBio (Instituto Nacional de Biodiversity) in recognition of his decade dedicated to the biodiversity understanding of the Hemiptera of Costa Rica.

#### ﻿ Dolichogenidea jasonkelleyi

Taxon classificationAnimaliaHymenopteraBraconidae

Fernandez-Triana & Boudreault sp. nov.

535A121B-2982-593E-8718-009E91708636

https://zoobank.org/61E467F1-092D-498D-8DA3-93212ABEF4D5

Fig. 70A–F

##### Type material.

***Holotype*.** Costa Rica • Female, CNC; Guanacaste, Area de Conservación Guanacaste, Sector Cacao, Sendero Circular; 10.92714, -85.46683; 1,185 m; 18.xii.2008; D. H. Janzen & W. Hallwachs leg.; Malaise trap; Voucher code: DHJPAR0031200. ***Paratype*.** Costa Rica • 1 Male, CNC; DHJPAR0031217.

**Figure 70.**
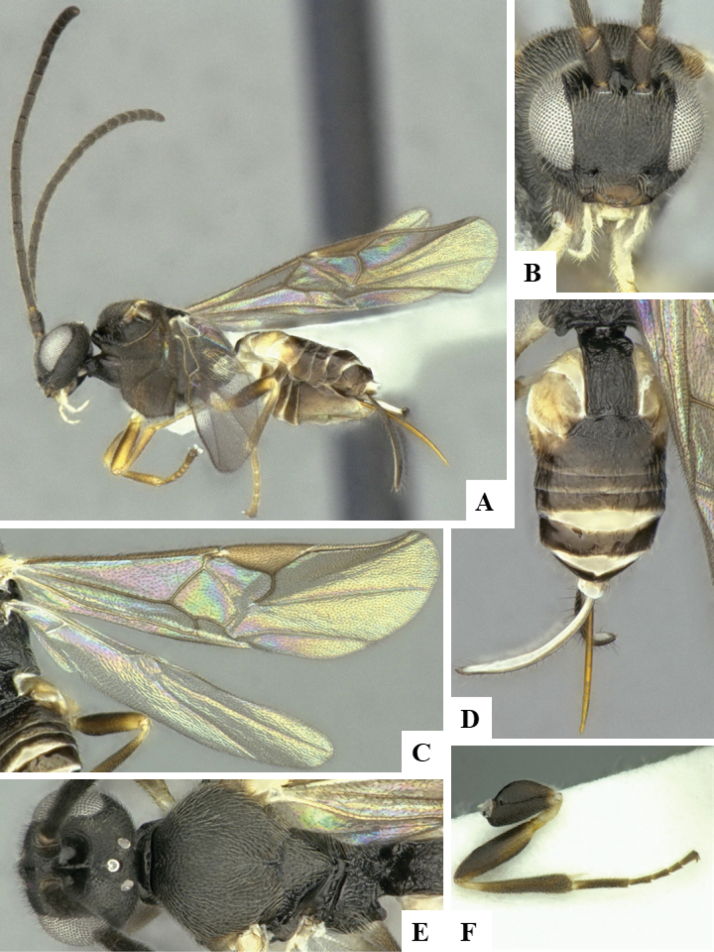
*Dolichogenideajasonkelleyi* Fernandez-Triana & Boudreault holotype female DHJPAR0031200 **A** habitus, lateral **B** head, frontal **C** wings **D** metasoma, dorsal **E** mesosoma, dorsal **F** hind leg, lateral.

##### Diagnostic description.

Scutellar disc mostly with shallow punctures; T1 parallel-sided but relatively thinner, its median length 2.2× its width at posterior margin; T1 mostly sculptured and with central hump; T2 mostly sculptured but not strongly and with smooth central area; ovipositor sheath approx. same length than metatibia length; tegula and humeral complex yellow; very small reddish brown spots along posterior margins of propleuron, dorsal margin of pronotum and postero-lateral margins of anteromesocutum, all of which are almost indistinguishable from mostly black mesosoma; pro- and mesocoxae mostly brown, metacoxa dark brown to black; mesofemur, metatibia (except for small pale spot on anterior 0.1) and metatarsus dark brown; body length: 2.60 mm; fore wing length: 2.50 mm. The shape and sculpture pattern of T1 and T2, ovipositor sheath length and leg coloration differentiate this species.

##### Distribution.

Costa Rica.

##### Biology.

No host data available.

##### DNA barcoding data.

BINBOLD:AAI9747 (21 sequences, 19 barcode compliant).

##### Etymology.

Named in honor and recognition of Mr. Jason Kelly of Boston as CEO of Gingko Bioworks, a synthetic biology foundry with strong potential for non-damaging development of complex tropical biodiversity genomics as a potential product from wild tropical ecosystems, and therefore increasing their intact desirability by tropical societies.

#### ﻿ Dolichogenidea jennyphillipsae

Taxon classificationAnimaliaHymenopteraBraconidae

Fernandez-Triana & Boudreault sp. nov.

2FEF1685-E34C-5AFB-8D89-BFDA142C0121

https://zoobank.org/4B713E45-D31B-459F-A4E4-BE9FB03A5642

Fig. 71A–G

##### Type material.

***Holotype*.** Costa Rica • Female, CNC; Guanacaste, Area de Conservación Guanacaste, Sector Santa Rosa, Bosque Humedo; 10.85145, -85.60801; 290 m; 05.vi.2000; D. H. Janzen & W. Hallwachs leg.; Malaise trap; Voucher code: DHJPAR0013114.

**Figure 71.**
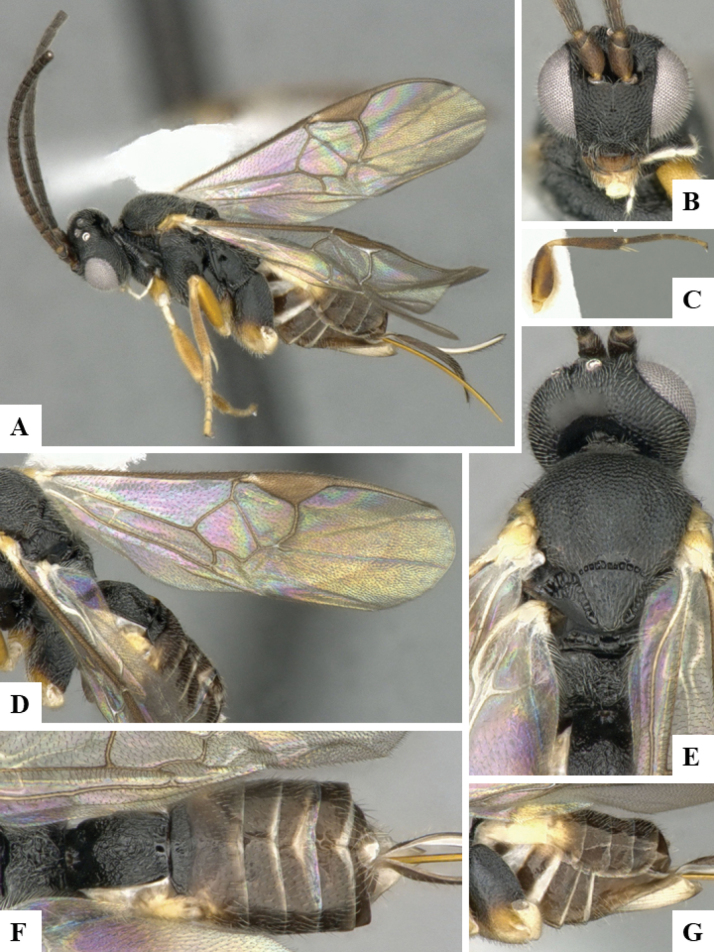
*Dolichogenideajennyphillipsae* Fernandez-Triana & Boudreault holotype female DHJPAR0013114 **A** habitus, lateral **B** head, frontal **C** hind leg, lateral **D** wings **E** mesosoma, dorsal **F** metasoma, dorsal **G** metasoma, lateral.

##### Other material.

Costa Rica • 1 Female, CNC; DHJPAR0013076.

##### Diagnostic description.

T1 mostly parallel-sided, its length medially ~ 2.5× its width at posterior margin; T2 transverse, its length medially 4.0–5.0× its width at posterior margin; T1 mostly sculptured on posterior 0.5; T2 mostly sculptured but with central area smooth; tegula and humeral complex yellow; pro- and mesocoxae yellow-white or yellow; metacoxa mostly yellow with dark brown spot on anterior 0.3; most of metafemur and metatibia brown; body length: 2.60 mm; fore wing length: 2.53 mm. Among all species with T2 at least partially smooth and pro- and mesocoxae pale, *D.jennyphillipsae* can be distinguished by T1 shape, T2 shape and sculpture, and color of tegula, humeral complex, coxae, mesofemur and metatibia. Two species (*D.isidrochaconi* and *D.robertofernandezi*) are very similar morphologically to *D.jennyphillipsae* and we could not find any substantial morphological character to reliably separate them; the characters provided in the key are subtle and may not hold for all specimens of each species. These three species were found in the same localities and at the same elevation (~ 300 m); and in all cases the host data is unknown (the three species were only collected with Malaise traps). However, here we consider them as separate species based on the substantial differences of their DNA barcodes: a) *D.jennyphillipsae* has 53 specimens with barcode-complaint sequences, which only have 0–5 base pairs of intraspecific variation (0.06–0.72%) whereas it is different from its closest ACG species (*D.isidrochaconi*) by 30 base pairs (4.64%) and it has 21 diagnostic base pairs to differentiate from the other two species; b) *D.robertofernandezi* has 33 specimens with barcode-complaint sequences, which only have 0–1 base pairs of intraspecific variation (0.01–0.18%) whereas it is different from its closest species (*D.isidrochaconi*) by 16 base pairs (2.42%) and it has ten diagnostic base pairs to differentiate from the other two species; c) *D.isidrochaconi* has 48 specimens with barcode-complaint sequences, which only have 1–6 base pairs of intraspecific variation (0.11–0.93%) whereas it is different from its closest species (*D.robertofernandezi*) by 16 base pairs (2.42%) and it has five diagnostic base pairs to differentiate from the other two species.

##### Distribution.

Costa Rica.

##### Biology.

No host data available.

##### DNA barcoding data.

BINBOLD: BOLD:AAM5088 (79 sequences, 53 barcode compliant).

##### Etymology.

Named in honor of Dr. Eugenie Phillips of San Jose, Costa Rica, a long-standing member of the taxonomic effort to identify and describe the microlepidoptera of Costa Rica, in recognition of that decades-long role and her current role as a member of the directorate of BioAlfa, the ACG/GDFCF project to facilitate bioliteracy for the non-damaging conservation of wild tropical biodiversity.

#### ﻿ Dolichogenidea jessiehillae

Taxon classificationAnimaliaHymenopteraBraconidae

Fernandez-Triana & Boudreault sp. nov.

00E8350F-03A6-5410-A3FB-05B15337494C

https://zoobank.org/CB782718-B4F7-49B8-A23F-662F5FED1DD5

Fig. 72A–F

##### Type material.

***Holotype*.** Costa Rica • Female, CNC; Guanacaste, Area de Conservación Guanacaste, Sector Santa Rosa, Area Administrativa; 10.83764, -85.61871; 295 m; 25.xii.2008; D. H. Janzen & W. Hallwachs leg.; Malaise trap; Voucher code: DHJPAR0031865. ***Paratypes*.** Costa Rica • 10 Females, CNC; DHJPAR0031701, DHJPAR0031868, DHJPAR0031861, DHJPAR0031867, DHJPAR0031849, DHJPAR0031857, DHJPAR0031855, DHJPAR0031866, DHJPAR0031847, DHJPAR0031856.

**Figure 72.**
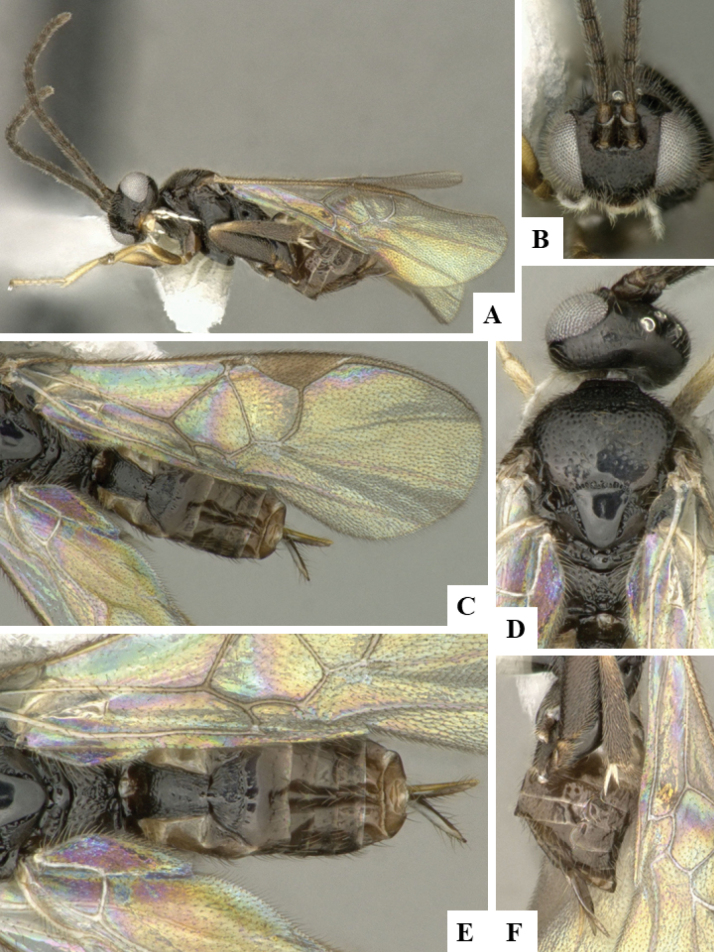
*Dolichogenideajessiehillae* Fernandez-Triana & Boudreault holotype female DHJPAR0031865 **A** habitus, lateral **B** head, frontal **C** wings **D** mesosoma, dorsal **E** metasoma, dorsal **F** metasoma, lateral.

##### Diagnostic description.

Propodeum areola less defined anteriorly; T1 parallel-sided on anterior 0.6, from that point evenly narrowing towards posterior margin; T1 width at anterior margin 1.2× T1 width at posterior margin, T1 length 2.2× T1 width at posterior margin; T1 mostly smooth, but with some sculpture along lateral margins on posterior 0.5; T2 trapezoidal, its width at posterior margin ~ 2.0× its central length; T2 mostly sculptured but with relative large area smooth centrally; hypopygium mostly inflexible, with only small, apical, reduced (one or two) pleats; ovipositor sheath 0.50–0.55× as long as metatibia; tegula and humeral complex brown; profemur brown on anterior ~ 0.5; all coxae, metafemur and most of metatibia (except for anterior 0.2–0.3 yellow-white) brown to dark brown; body length: 1.80–1.90 mm; fore wing length: 1.90–2.10 mm. This species is distinctive because of its short ovipositor sheath, few and poorly defined hypopygium pleats, T1 and T2 sculpture and body size and color.

##### Distribution.

Costa Rica.

##### Biology.

No host data available.

##### DNA barcoding data.

BINBOLD:AAM5851 (11 sequences, 11 barcode compliant).

##### Etymology.

Named in honor of Mrs. Jessie Hill of Hawaii and Philadelphia, Pennsylvania, USA in recognition of her steady and enthusiastic interest in, and support of all of the GDFCF and ACG activities as an enthusiastic member of the Board of Directors for the Guanacaste Dry Forest Conservation Fund in its integration with Area de Conservación Guanacaste.

#### ﻿ Dolichogenidea johnrobinsoni

Taxon classificationAnimaliaHymenopteraBraconidae

Fernandez-Triana & Boudreault sp. nov.

DDA243D5-6CFF-550B-992D-45E87199BA84

https://zoobank.org/0A289322-8107-423E-A4E6-B4889CE0DC00

Fig. 73A–F

##### Type material.

***Holotype*.** Costa Rica • Female, CNC; Guanacaste, Area de Conservación Guanacaste, Sector Cacao, Cerro Pedregal, 10.92767, -85.47449; 1,080 m; 22.xi.2008; D. H. Janzen & W. Hallwachs leg.; Malaise trap; Voucher code: DHJPAR0033777.

**Figure 73.**
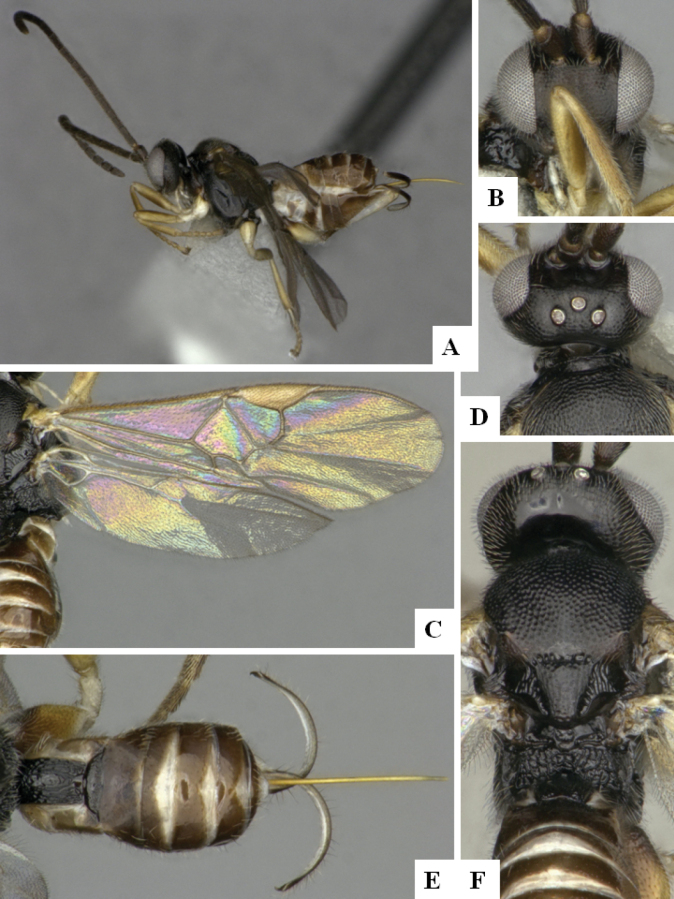
*Dolichogenideajohnrobinsoni* Fernandez-Triana & Boudreault holotype female DHJPAR0033777 **A** habitus, lateral **B** head, frontal **C** wings **D** head, dorsal **E** metasoma, dorsal **F** mesosoma, dorsal.

##### Diagnostic description.

T1 length medially ~ 2.2× its width at posterior margin; T2 more or less trapezoidal in shape; T2 mostly smooth but with some sculpture around margins; tegula and humeral complex yellow; pro- and mesocoxae yellow-white or yellow; metacoxa mostly yellow with dark brown spot on anterior 0.3; metafemur mostly yellow (thin brown area dorsally on apical 0.2–0.3); metatibia brown to dark brown; body length: 2.33 mm; fore wing length: 2.50 mm. Among all species with T2 mostly to entirely smooth and pro- and mesocoxae pale, *D.johnrobinsoni* can be distinguished by T1 shape, T2 shape and sculpture, and color of tegula, humeral complex, coxae, mesofemur and metatibia.

##### Distribution.

Costa Rica.

##### Biology.

No host data available.

##### DNA barcoding data.

BINBOLD:ACF0267 (1 sequence, barcode compliant).

##### Etymology.

Named after John Robinson of Philadelphia, USA, for his many helpful acts for DHJ and WH as the building administrator for their office on the University of Pennsylvania campus.

#### ﻿ Dolichogenidea jorgecarvajali

Taxon classificationAnimaliaHymenopteraBraconidae

Fernandez-Triana & Boudreault sp. nov.

E10FF026-1E7D-529A-B1FE-FC3755785804

https://zoobank.org/471DFCBB-8C89-4F9A-BA4A-383D8A308137

Fig. 74A–F

##### Type material.

***Holotype*.** Costa Rica • Female, CNC; Guanacaste, Area de Conservación Guanacaste, Sector Cacao, Cerro Pedregal; 10.92767, -85.47449; 1,080 m; 18.xii.2008; D. H. Janzen & W. Hallwachs leg.; Malaise trap; Voucher code: DHJPAR0031378. ***Paratypes*.** Costa Rica • 2 Males, CNC; DHJPAR0012730, DHJPAR0012559.

**Figure 74.**
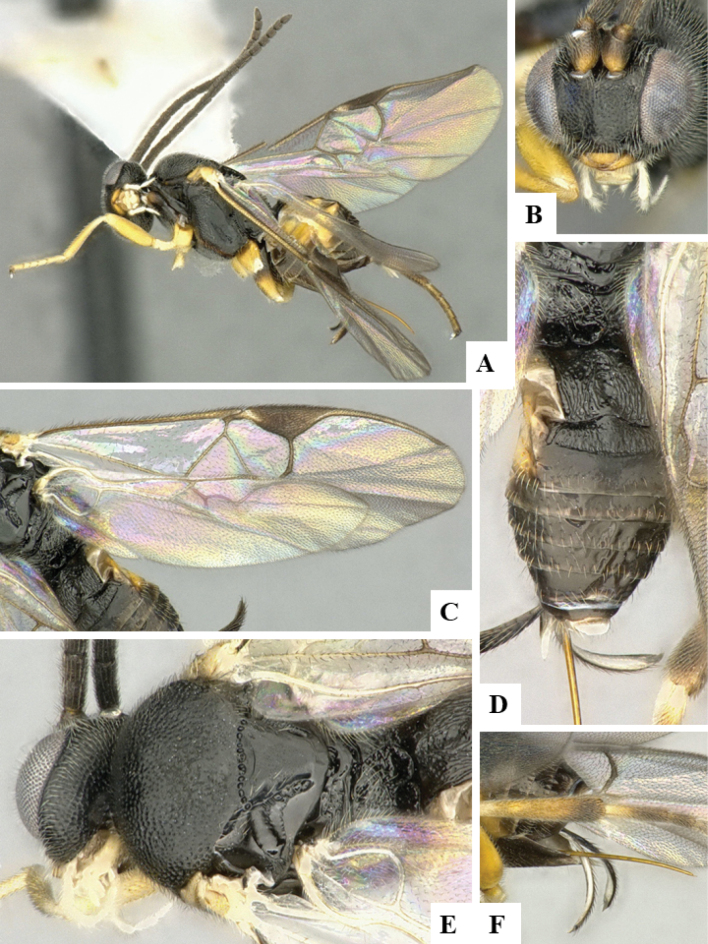
*Dolichogenideajorgecarvajali* Fernandez-Triana & Boudreault holotype female DHJPAR0031378 **A** habitus, lateral **B** head, frontal **C.** wings **D** metasoma, dorsal **E** mesosoma, dorsal **F** ovipositor, lateral.

##### Diagnostic description.

Posterior 0.5–0.6 of T1 and T2 mostly with strong sculpture, usually longitudinal striae covering entire surface (but T2 with small polished area centrally); T1 parallel-sided to slightly broadening posteriorly; T2 comparatively very transverse but with anterior and posterior margins strongly arcuate, so that T2 length is longer medially than laterally and thus T2 width at posterior margin is usually < 3.0× its length medially; ovipositor ≤ 1.4× as metatibia length; tegula and humeral complex yellow; pterostigma usually without pale spot at base or with small pale spot occupying < 0.1 pterostigma length; most laterotergites, some sternites and sometimes hypopygium yellow to yellow-brown; body length: 2.50 mm; fore wing length: 2.75 mm. This species has strong sculpture (usually longitudinal striae) covering posterior 0.5–0.6 of T1 and most of T2. However, unlike the majority of species with similarly strong sculpture, T2 has a central area which is smooth and also T2 is very transverse and with anterior and posterior margins strongly arcuate. Because of that unique shape and sculpture pattern of T2, as well as its metafemur color, it can be separate from all the species with entirely and strongly sculptured T2 which is not transverse, as well as all the species with smooth T2 and/or broad T2. Among similar species, *D.jorgecarvajali* can be distinguished from *D.anniapicadoae* because of its much shorter ovipositor, and from *D.rexhamiltoni* because of different coloration of tegula, humeral complex, laterotergites, sternites and hypopygium.

##### Distribution.

Costa Rica.

##### Biology.

No host data available.

##### DNA barcoding data.

BINBOLD:AAM5847 (65 sequences, 65 barcode compliant).

##### Etymology.

Named in honor of Sr. Jorge Carvajal of Santo Domingo de Heredia, San Jose, Costa Rica in recognition of his two decades of being highly reliable support staff for the former INBio and now, BioAlfa headquartered in the INBio facilities in Santo Domingo de Heredia.

#### ﻿ Dolichogenidea jorgecortesi

Taxon classificationAnimaliaHymenopteraBraconidae

Fernandez-Triana & Boudreault sp. nov.

BDE0EBC2-3FE7-518B-9858-B66EAE1F97CE

https://zoobank.org/31A2A21D-B2D6-4DB9-8ABB-828C4FE4FA7D

Fig. 75A–H

##### Type material.

***Holotype*.** Costa Rica • Female, CNC; Guanacaste, Area de Conservación Guanacaste, Sector Cacao, Sendero Arenales; 10.9247, -85.4674; 1,080 m; 18.xii.2008; D. H. Janzen & W. Hallwachs leg.; Malaise trap; Voucher code: DHJPAR0031285.

**Figure 75.**
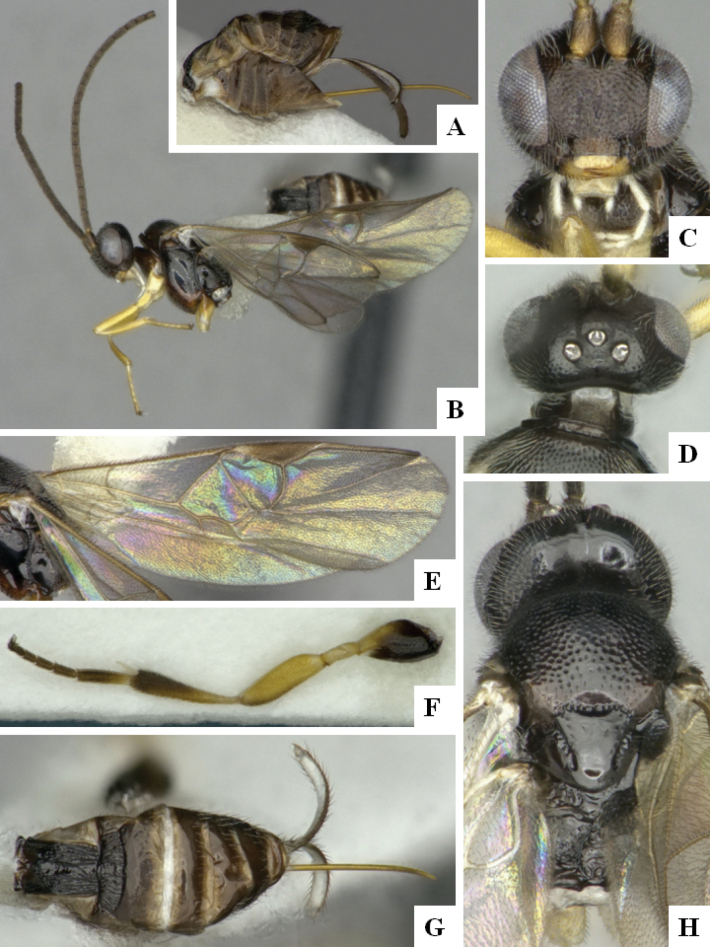
*Dolichogenideajorgecortesi* Fernandez-Triana & Boudreault holotype female DHJPAR0031285 **A** metasoma, lateral **B** habitus, lateral **C** head, frontal **D** head, dorsal **E** wings **F** hind leg, lateral **G** metasoma, dorsal **H** mesosoma, dorsal.

##### Diagnostic description.

Scutellar disc smooth; anterior half of mesopleuron smooth; anteromesoscutum with sparse and relatively shallow punctures; propodeum areola comparatively broad (its height ~ 1.2× its central width) and open anteriorly; T1 and T2 heavily sculptured with strong longitudinal striae; T1 comparatively thin and mostly parallel-sided but posterior 0.1–0.3 slightly narrowing towards posterior margin; T2, comparatively less transverse, its width at posterior margin 2.8× its central length; ovipositor sheath ~ 1.0× metatibia length; ovipositor comparatively very thin, much thinner than half flagellomeres width; tegula white-yellow, humeral complex mostly brown; pterostigma mostly pale brown with small, paler spot anteriorly; metacoxa entirely dark brown; metatibia yellow at least on anterior 0.4–0.5; body length: 2.35 mm; fore wing length: 2.53 mm; BINBOLD:AAM5846, which is 4.52% different from the nearest BIN in BOLD as of March 2022. The coloration pattern, shape of T2, ovipositor length and width, and body size are distinctive among species with T1 and T2 heavily sculptured and T1 comparatively thin.

##### Distribution.

Costa Rica.

##### Biology.

No host data available.

##### DNA barcoding data.

BINBOLD:AAM5846 (1 sequence, barcode compliant).

##### Etymology.

Named in honor of Dr. Jorge Cortes of San Jose, Costa Rica and of CIMAR of the Universidad de Costa Rica for his strong example and support of integrating university-level education with the Marine Parataxonomists program of GDFCF and ACG in northwestern Costa Rica.

#### ﻿ Dolichogenidea josealfredohernandezi

Taxon classificationAnimaliaHymenopteraBraconidae

Fernandez-Triana & Boudreault, 2019

372123CC-4E13-5826-98FA-58421EA35B5B

Fig. 76A–E

##### Notes.

Full details for this species in Fernandez-Triana et al. (2019). See also the key and Table 1 above.

**Figure 76.**
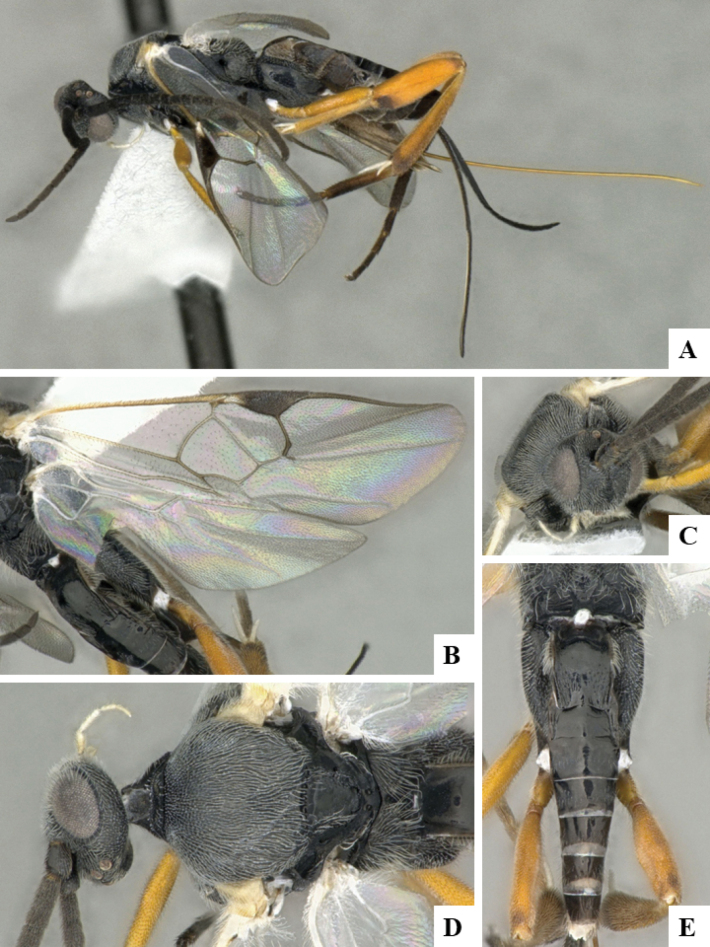
*Dolichogenideajosealfredohernandezi* Fernandez-Triana & Boudreault holotype female DHJPAR0049909 **A** habitus, lateral **B** wings **C** head, fronto-lateral **D** mesosoma, dorsal **E** metasoma, dorsal.

#### ﻿ Dolichogenidea josephfridmani

Taxon classificationAnimaliaHymenopteraBraconidae

Fernandez-Triana & Boudreault sp. nov.

EBCDE47C-EDC5-5FC9-AD32-E2215F6538C4

https://zoobank.org/235F2EC2-8BEF-4218-9463-B682A6F1EE40

Fig. 77A–F

##### Type material.

***Holotype*.** Costa Rica • Female, CNC; Guanacaste, Area de Conservación Guanacaste, Sector El Hacha, Sendero Bejuquilla; 11.03004, -85.52699; 280 m; 05.iv.1999; D. H. Janzen & W. Hallwachs leg.; Malaise trap; Voucher code: DHJPAR0012558. ***Paratypes*.** Costa Rica • 2 Females, 1 Male, CNC; DHJPAR0024737, DHJPAR0024730, DHJPAR0024738.

**Figure 77.**
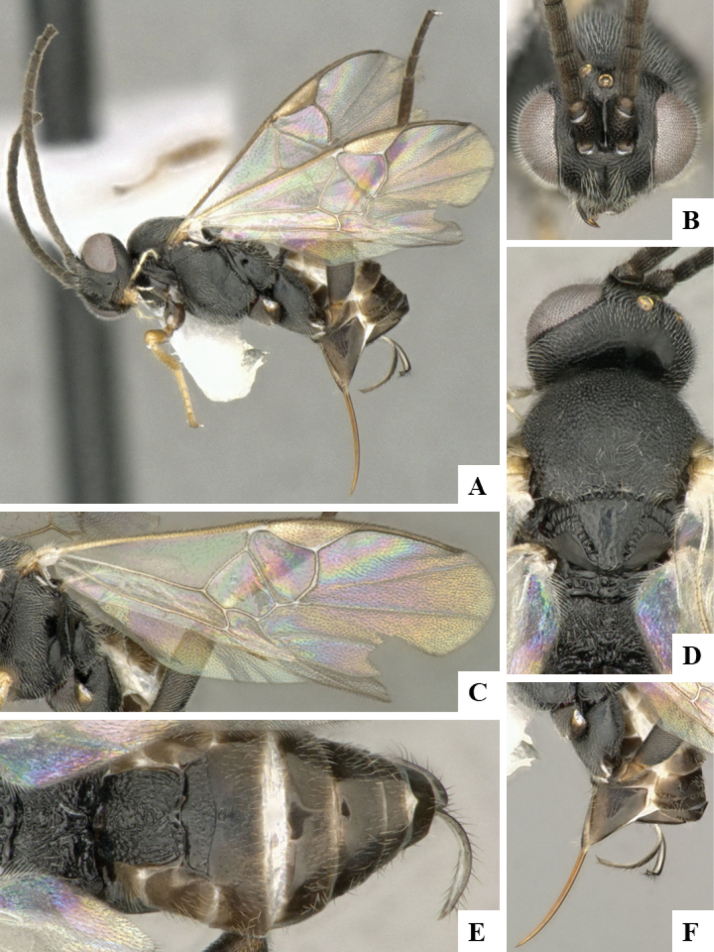
*Dolichogenideajosephfridmani* Fernandez-Triana & Boudreault holotype female DHJPAR0012558 **A** habitus, lateral **B** head, frontal **C** wings **D** mesosoma, dorsal **E** metasoma, dorsal **F** ovipositor, lateral.

##### Diagnostic description.

Anteromesoscutum dull, with rather coarse punctures; scutellar disc mostly smooth and shiny, with very few punctures along margins; fore wing vein R1 significantly longer than pterostigma and > 4.0× as long as the space between its end and end of vein 3RSb; propodeum with coarse sculpture; T1 strongly sculptured on posterior 0.6; T2 almost entirely sculptured but smooth centrally or along margins; T2 transverse, its width at posterior margin 4.0× its central length; tegula and humeral complex yellow; pterostigma mostly yellow or white-yellow, with thin brown margins; pro- and mesocoxae brown, metacoxa dark brown to black; metafemur mostly dark brown, metatibia yellow on anterior ~ 0.4, rest dark brown; body length: 2.35–2.73 mm; fore wing length: 2.50–2.60 mm. The shape and sculpture of T1 and T2, sculpture of mesosoma (especially contrast between anteromesoscutum, scutellar disc and propodeum), and color of tegula, humeral complex, pterostigma and legs distinguish this species among all others with T2 sculptured but transverse.

##### Distribution.

Costa Rica.

##### Biology.

No host data available.

##### DNA barcoding data.

BINBOLD:AAE8602 (20 sequences, 20 barcode compliant).

##### Etymology.

Named in honor of Mr. Joseph Fridman of Gingko Bioworks in recognition of his warm and detailed 2022 welcome to representatives from GDFCF/ACG exploring the potential of a synthetic biology foundry with strong potential for non-damaging development of complex tropical biodiversity genomics as a potential product from wild tropical ecosystems, and therefore increasing their intact desirability by tropical societies.

#### ﻿ Dolichogenidea joshdarfleri

Taxon classificationAnimaliaHymenopteraBraconidae

Fernandez-Triana & Boudreault sp. nov.

11F43944-4DCC-5978-9609-2C4466AC41CA

https://zoobank.org/DEB1FC02-BA73-4F4B-8761-36BDB61CFBD9

Fig. 78A–F

##### Type material.

***Holotype*.** Costa Rica • Female, CNC; Alajuela, Area de Conservación Guanacaste, Sector Rincon Rain Forest, Vado Rio Francia; 10.90093, -85.28915; 400 m; 09.ix.2007; D. H. Janzen & W. Hallwachs leg.; Malaise trap; Voucher code: DHJPAR0025369. ***Paratypes*.** Costa Rica • 3 Females, CNC; DHJPAR0025356, DHJPAR0025400, DHJPAR0025424.

**Figure 78.**
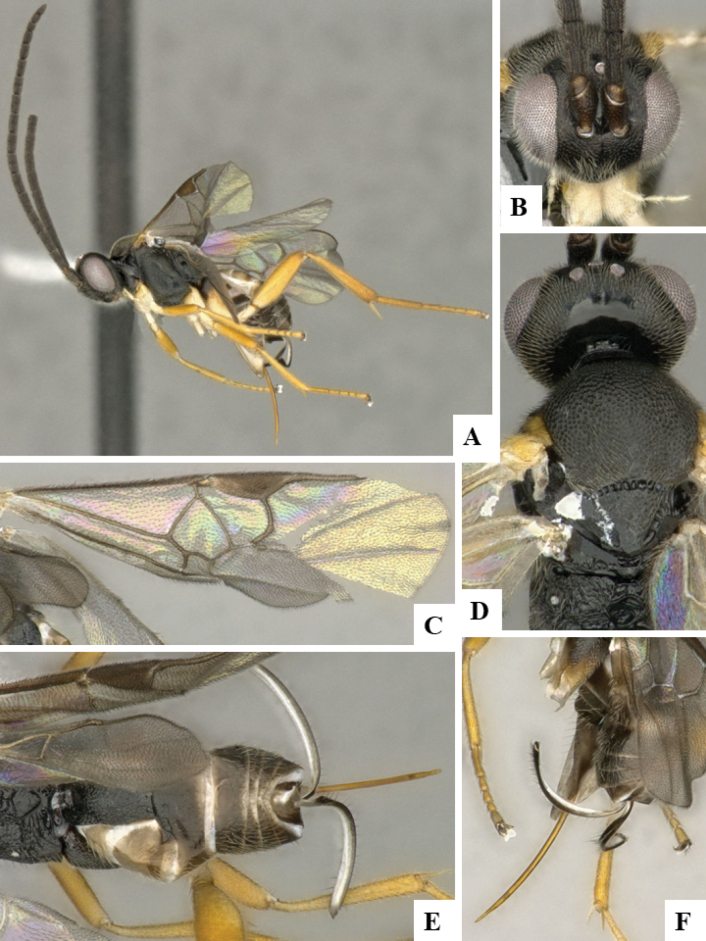
*Dolichogenideajoshdarfleri* Fernandez-Triana & Boudreault holotype female DHJPAR0025369 **A** habitus, lateral **B** head, frontal **C** wings **D** mesosoma, dorsal **E** metasoma, dorsal **F** ovipositor, lateral.

##### Diagnostic description.

Anteromesoscutum with relatively coarse punctures; scutellar disc mostly with punctures; anterior 0.3 of mesopleuron and posterior 0.4–0.5 of metapleuron sculptured; T1 parallel-sided or mostly parallel-sided, its length medially less ~ 3.0× its width at posterior margin; T2 centrally smooth, with some sculpture along margins; T2 comparatively very transverse, its length medially ~ 5.0× its width at posterior margin; tegula and humeral complex yellow; legs mostly pale (yellow), except for metacoxa with anterior 0.4 brown; body length: 2.70–2.90 mm; fore wing length: 2.80–3.00 mm. Among all species with smooth T2 and pale pro- and mesocoxae, *D.joshdarfleri* can be distinguished by coarse punctures on several areas of mesosoma, its T1 and T2 shape, T2 sculpture, color of tegula and humeral complex, and body size.

##### Distribution.

Costa Rica.

##### Biology.

No host data available.

##### DNA barcoding data.

BINBOLD:AAC7481 (11 sequences, 6 barcode compliant).

##### Etymology.

Named after Josh Darfler in recognition of his months of administrating meetings for the Department of Biology housing DHJ and WH.

#### ﻿ Dolichogenidea juanmatai

Taxon classificationAnimaliaHymenopteraBraconidae

Fernandez-Triana & Boudreault sp. nov.

AF2457E2-D6C7-59FA-83DB-811383D303D3

https://zoobank.org/DFD3117D-2C09-434A-81EE-1D55DECE1E30

Fig. 79A–F

##### Type material.

***Holotype*.** Costa Rica • Female, CNC; Guanacaste, Area de Conservación Guanacaste, Sector Cacao, Sendero Arenales; 10.92471, -85.46738; 1,080 m; 18.xii.2008; D. H. Janzen & W. Hallwachs leg.; Malaise trap; Voucher code: DHJPAR0031295.

**Figure 79.**
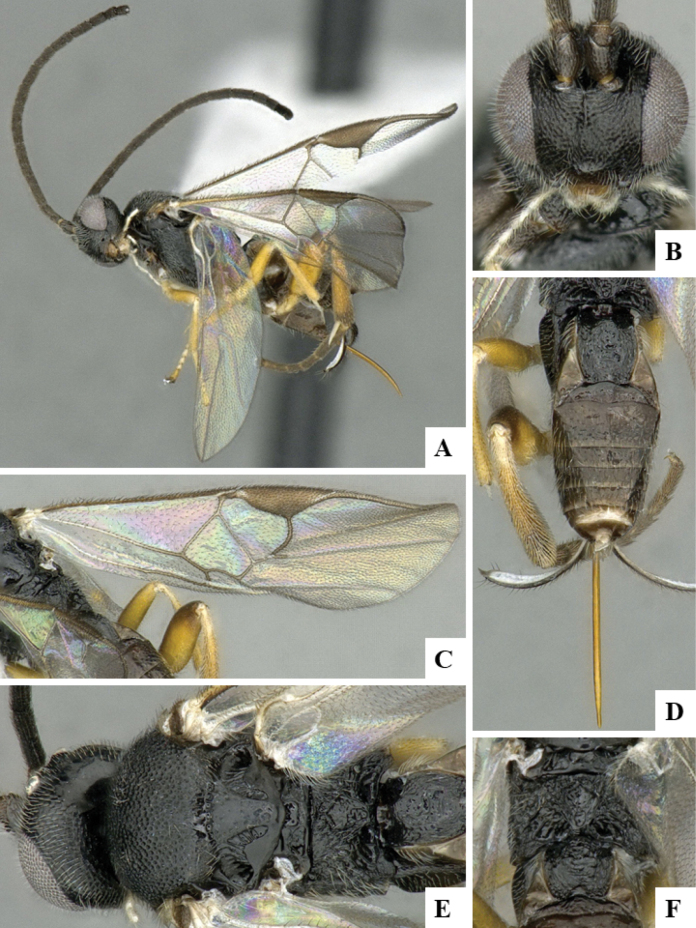
*Dolichogenideajuanmatai* Fernandez-Triana & Boudreault holotype female DHJPAR0031295 **A** habitus, lateral **B** head, frontal **C** fore wing **D** metasoma, dorsal **E** mesosoma, dorsal **F** propodeum & T1–T3, dorsal.

##### Diagnostic description.

Anteromesoscutum punctures near end of notauli well separated and similar to punctures on rest of anteromesoscutum; T1 mostly parallel-sided but posterior 0.3 slightly narrowing towards posterior margin; T1 mostly sculptured on posterior 0.5; T2 smooth; T2 transverse and comparatively narrow, its width at posterior margin > 3.0× its length medially; scape entirely dark brown to black; tegula yellow, much paler than brown humeral complex; pro- and mesocoxae brown, metacoxa dark brown to black; metafemur mostly yellow, with brown spot on apical 0.1 dorsally; metatibia mostly yellow, with posterior 0.1–0.2 brown; body length: 2.45 mm; fore wing length: 2.65 mm. Among all species with T2 smooth and dark coxae *D.juanmatai* can be distinguished by T1 sculpture, T2 shape, anteromesoscutum punctures, and scape, tegula and leg color. Another species, *D.jaimelewisi* is similar morphologically but has different coloration of scape, tegula and legs, as well as different puncture sculpture on anteromesoscutum.

##### Distribution.

Costa Rica.

##### Biology.

No host data available.

##### DNA barcoding data.

BINBOLD:AAM5740 (one sequence, barcode compliant).

##### Etymology.

Named in honor of Sr. Juan Mata of Costa Rica, and the Costa Rican National Museum, BioAlfa and the former INBio (Instituto Nacional de Biodiversity) in recognition of his two+ decades dedicated to the biodiversity understanding of the Mantidae and the photography of taxonomic specimens for publications.

#### ﻿ Dolichogenidea junhyongkimi

Taxon classificationAnimaliaHymenopteraBraconidae

Fernandez-Triana & Boudreault sp. nov.

059B6EA8-3234-507E-B66F-BE52695BC8DB

https://zoobank.org/20076685-FB58-4527-A199-54F7F193A696

Fig. 80A–F

##### Type material.

***Holotype*.** Costa Rica • Female, CNC; Alajuela, Area de Conservación Guanacaste, Sector San Cristobal, Rio Blanco Abajo; 10.90037, -85.37254; 500 m; 28.vii.2007; D. H. Janzen & W. Hallwachs leg.; Malaise trap; Voucher code: DHJPAR0025352. ***Paratypes*.** Costa Rica • 2 Females, 2 Males, CNC; DHJPAR0026801, DHJPAR0025581, DHJPAR0026946, DHJPAR0026805.

**Figure 80.**
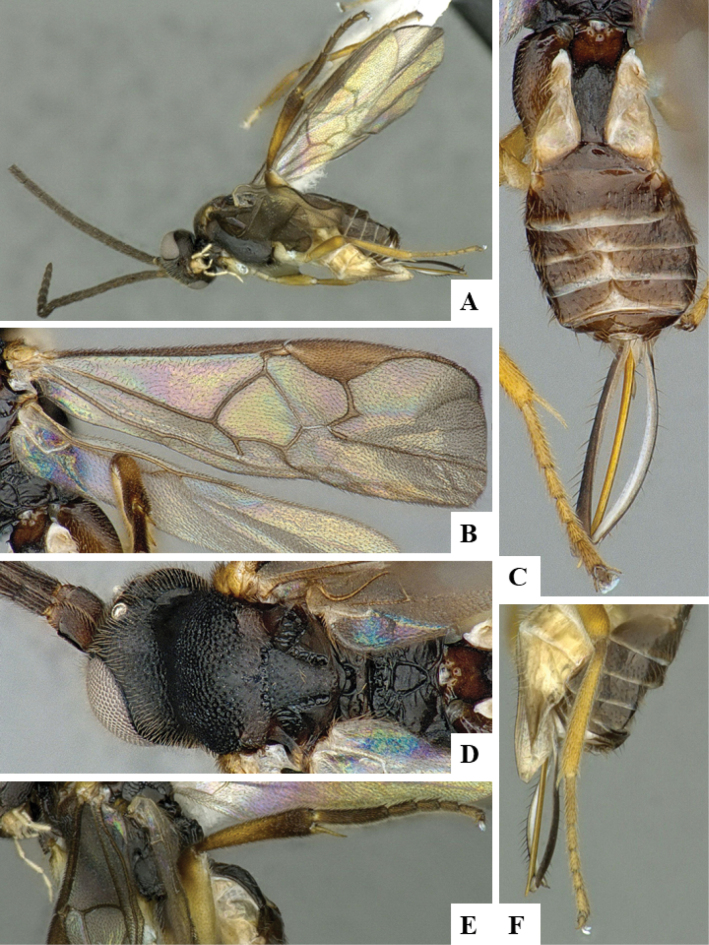
*Dolichogenideajunhyongkimi* Fernandez-Triana & Boudreault holotype female DHJPAR0025352 **A** habitus, lateral **B** wings **C** metasoma, dorsal **D** mesosoma, dorsal **E** hind leg, lateral **F** ovipositor, lateral.

##### Diagnostic description.

T1 very strongly narrowing towards posterior margin (T1 width at anterior margin > 2.0× width at posterior margin, and T1 median length > 5.0× width at posterior margin); T2 smooth; ovipositor sheath almost as long as metatibia length (0.9×); tegula yellow; legs mostly pale colored (including pro- and mesocoxae entirely white-yellow, metacoxa with apical 0.3 yellow, metafemur and metatibia mostly yellow, and metatibial spurs yellow); body length: 2.23–2.38 mm; fore wing length: 2.35–2.65 mm; BINBOLD:AAD6850, which is 5.93% different from the nearest BIN in BOLD as of March 2022. No other described *Dolichogenidea* species in the Neotropics has T1 so strongly narrowing towards posterior margin. *D.ingredolsonae* is relatively similar morphologically, but it has T1 less strongly narrowing, shorter ovipositor sheath and different color of tegula.

##### Distribution.

Costa Rica.

##### Biology.

No host data available.

##### DNA barcoding data.

BINBOLD:AAD6850 (5 sequences, 5 barcode compliant).

##### Etymology.

Named in honor of Dr. Junhyong Kim in recognition of his outstanding yet especially stressful 5-year term as Department Chairman of the Biology Department of the University of Pennsylvania, Philadelphia, Pennsylvania, USA, during these years of COVID and university turmoil.

#### ﻿ Dolichogenidea kasiiya

Taxon classificationAnimaliaHymenopteraBraconidae

Fernandez-Triana & Boudreault sp. nov.

BDEF767A-39B2-53CC-B580-F437719A05B7

https://zoobank.org/130CB3C0-3085-4709-96C3-C80E35F65CEC

Fig. 81A–E

##### Type material.

***Holotype*.** Costa Rica • Female, CNC; Guanacaste, Area de Conservación Guanacaste, Sector Santa Rosa, Area Administrativa; 10.83764, -85.61871; 295 m; 25.xii.2008; D. H. Janzen & W. Hallwachs leg.; Malaise trap; Voucher code: DHJPAR0031741.

**Figure 81.**
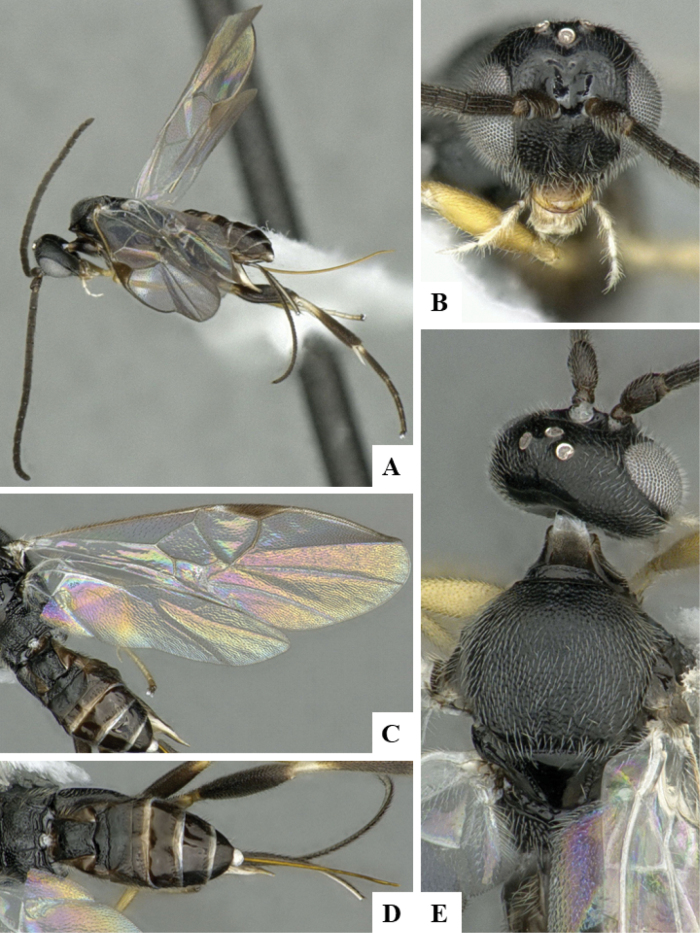
*Dolichogenideakasiiya* Fernandez-Triana & Boudreault holotype female DHJPAR0031741 **A** habitus, lateral **B** head, frontal **C** wings **D** metasoma, dorsal **E** mesosoma, dorsal.

##### Diagnostic description.

Propodeum sculptured but with almost complete areola (clearly defined posteriorly by carinae, anteriorly open); T1 mostly smooth, at most with weak sculpture on posterior 0.3; T2 smooth; ovipositor sheath 1.6× as long metatibia length; ovipositor apically sinuate; mesosternum with a stripe yellow-brown, contrasting with rest of dark brown mesosternum; all coxae dark brown; hind leg mostly dark brown except for anterior 0.3 of metatibia yellow and yellow-white metatibial spurs; body length: 2.50 mm; fore wing length: 2.60 mm. Among all species with dark coxae and smooth or mostly smooth T1 and T2, *D.kasiiya* is distinctive because of its apically sinuate ovipositor, length of ovipositor sheath, smooth T2 and propodeum sculpture.

##### Distribution.

Costa Rica.

##### Biology.

No host data available.

##### DNA barcoding data.

BINBOLD:AAM5750 (1 sequence, barcode compliant).

##### Etymology.

Named in honor of Mr. Mehdi Rheljari’s High End Hotel Kasiiya in whose tropical dry forest an inventory Malaise trap is now supported and running, on the Nicoya coast of Guanacaste Province for 2022–2023.

#### ﻿ Dolichogenidea katiemccluskeyae

Taxon classificationAnimaliaHymenopteraBraconidae

Fernandez-Triana & Boudreault sp. nov.

162AD70A-A536-50A4-AECD-E1C947C8B8D3

https://zoobank.org/3B213634-6281-4AA4-9DC5-AF4D2EF9C50E

Fig. 82A–F

##### Type material.

***Holotype*.** Costa Rica • Female, CNC; Guanacaste, Area de Conservación Guanacaste, Sector Cacao, Sendero Arenales; 10.92471, -85.46738; 1,080 m; 18.xii.2008; D. H. Janzen & W. Hallwachs leg.; Malaise trap; Voucher code: DHJPAR0031303.

**Figure 82.**
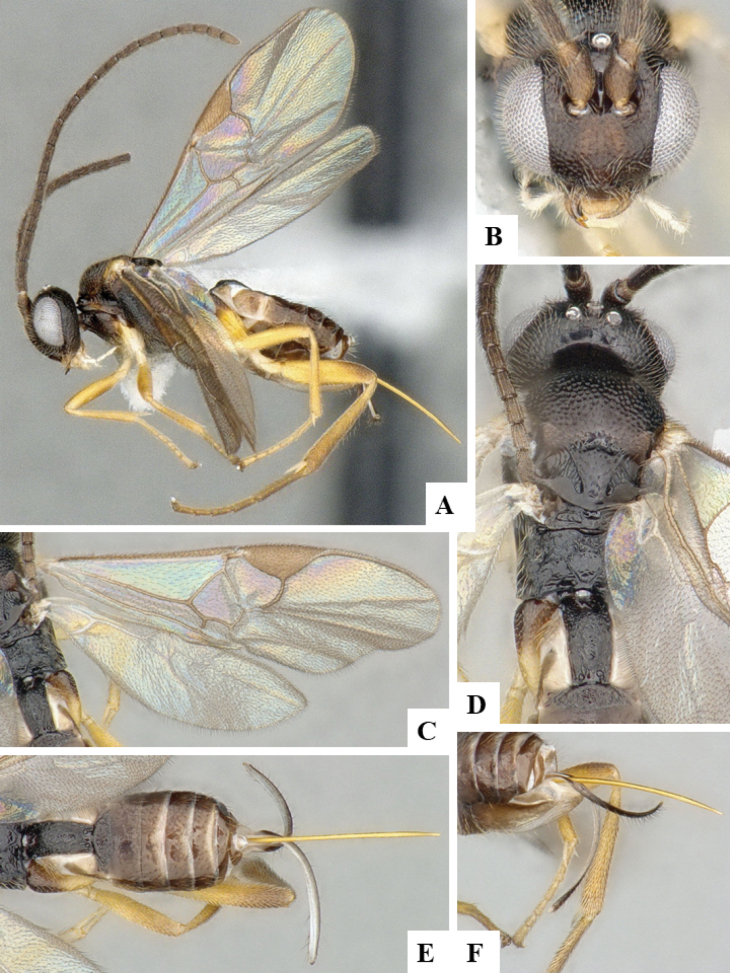
*Dolichogenideakatiemccluskeyae* Fernandez-Triana & Boudreault holotype female DHJPAR0031303 **A** habitus, lateral **B** head, frontal **C** wings **D** mesosoma, dorsal **E** metasoma, dorsal **F** ovipositor, lateral.

##### Other material.

Costa Rica • 2 Females, CCDB; BIOUG70860-G04, BIOUG91473-F09. Honduras • 1 Female, CCDB; BIOUG08167-E03.

##### Diagnostic description.

T1 parallel-sided or mostly parallel-sided, its length medially ≤ 3.0× its width at posterior margin; T2 smooth; T2 comparatively less transverse, its length medially 3.0× its width at posterior margin; ovipositor sheath 1.0× as long as metatibia; legs mostly pale (yellow), except for metacoxa with anterior 0.5 brown; body length: 2.00 mm; fore wing length: 2.20 mm. Among all species with smooth T2 and pale pro- and mesocoxae, *D.katiemccluskeyae* can be distinguished by its T1 and T2 shape, and comparatively small body size (among the smallest described species *Dolichogenidea* in the Neotropics).

##### Distribution.

Costa Rica, Honduras.

##### Biology.

No host data available.

##### DNA barcoding data.

BINBOLD:ACE8228 (4 sequences, 4 barcode compliant).

##### Etymology.

Named in honor of Ms. Katie McCluskey, a double Master student at the University of Pennsylvania and Office Technician for the Janzen-Hallwachs insect processing laboratory, and specifically for DNA barcoding de-legging many of the same specimens described in this taxonomic treatment of *Dolichogenidea*.

##### Notes.

The specimen from Honduras is assigned to this species based on its sequence matching with the ACG sequences (the holotype and two other ACG specimens which we could not examine and therefore are not considered as paratypes).

#### ﻿ Dolichogenidea kenzabaddouae

Taxon classificationAnimaliaHymenopteraBraconidae

Fernandez-Triana & Boudreault sp. nov.

55BFC17A-66EE-5031-BD04-8F66BDCF466F

https://zoobank.org/748C74F2-2D6A-4207-B91B-35FE5C14BD80

**Figure 83.**
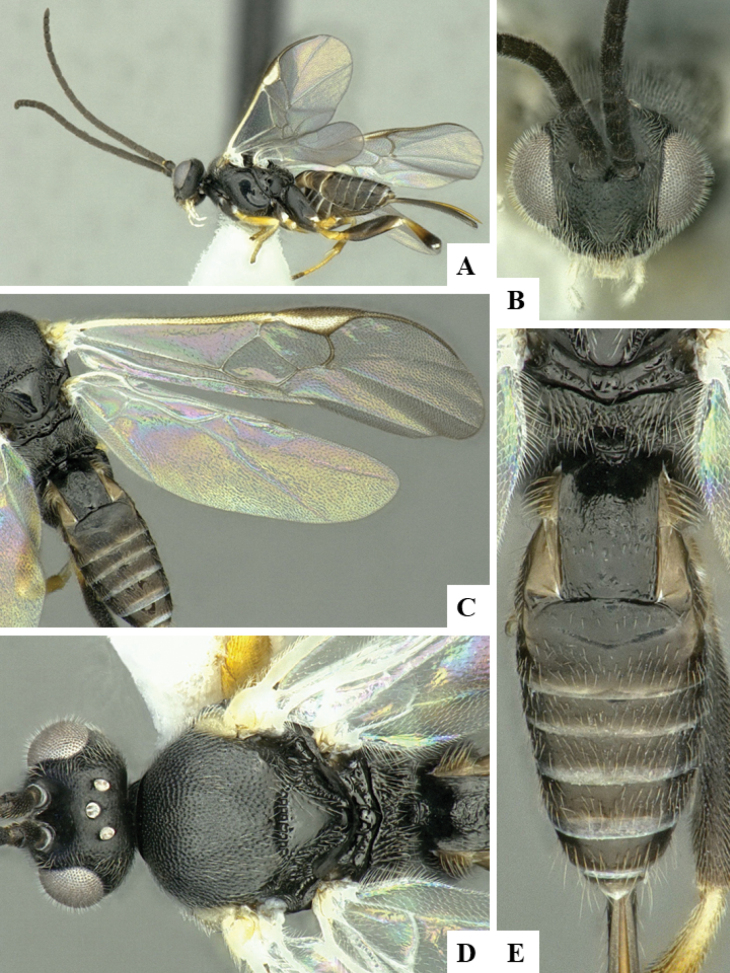
*Dolichogenideakenzabaddouae* Fernandez-Triana & Boudreault holotype female DHJPAR0051275 **A** habitus, lateral **B** head, frontal **C** wings **D** mesosoma, dorsal **E** metasoma, dorsal.

**Figure 84.**
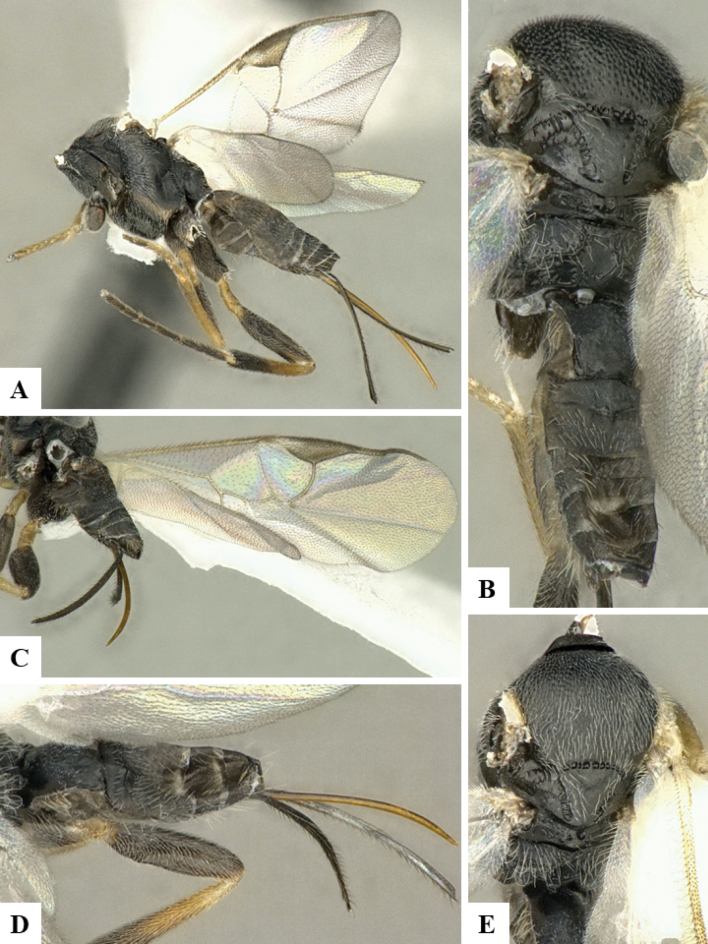
*Dolichogenideakenzabaddouae* Fernandez-Triana & Boudreault paratype female DHJPAR0051868 **A** habitus, lateral **B** habitus, dorso-lateral **C** wings **D** metasoma, dorso-lateral **E** mesosoma, dorsal.

Figs 83A–E , 84A–E , 157A

##### Type material.

***Holotype*.** Costa Rica • Female, CNC; Guanacaste, Area de Conservación Guanacaste, Sector San Cristobal, Finca San Gabriel; 10.87766, -85.39343; 645 m; 23.i.2013; Gloria Sihezar leg.; Host: *Antaeotricha* Janzen221; Voucher code: DHJPAR0051275; Host voucher code: 13-SRNP-379. ***Paratype*.** Costa Rica • 1 Female, DHJPAR0051868.

##### Diagnostic description.

Anteromesoscutum rather coarsely punctured; vein R1 much longer than pterostigma length; propodeum with complete areola; T1 and T2 mostly smooth but T1 with some weak sculpture on posterior 0.5, especially along margins; ovipositor sheath shorter than metasoma length and 1.3× as long as metatibia; tegula and humeral complex white or yellow; pterostigma pale colored, either mostly yellow-white with thin brown margins, or very pale brown (almost transparent) with small, paler spot at base; body length and fore wing length: 2.60–2.70 mm. Among all species with dark coxae and smooth or mostly smooth T1 and T2, *D.kenzabaddouae* can be distinguished by T1 sculpture, propodeum areola, color of tegula, humeral complex and pterostigma, ovipositor sheath length and fore wing vein R1 length.

##### Distribution.

Costa Rica.

##### Biology.

Solitary. Depressariidae: *Antaeotricha* Janzen221.

##### DNA barcoding data.

BINBOLD:AAY4695 (11 sequences, 9 barcode compliant).

##### Etymology.

Named in honor of Mrs. Kenzabaddou in recognition of her willingness to support a Malaise trap insect inventory of her tropical dry forest on her ecologically-oriented hotel Kasiiya on the Nicoya coast of Guanacaste Province for 2022–2023.

#### ﻿ Dolichogenidea lacochaparamo

Taxon classificationAnimaliaHymenopteraBraconidae

Fernandez-Triana & Boudreault sp. nov.

99E1C94B-0ABA-5475-B19D-F2AF17989CCC

https://zoobank.org/45C6E123-6A10-4E99-ADF8-FE54961A218C

Fig. 85A–G

##### Type material.

***Holotype*.** Colombia • Female, CNC; Putumayo, 1°10'N, 77°15'W; 2,900 m; 2.xii.1972; J. Helava leg.; Voucher code: CNC1180003.

**Figure 85.**
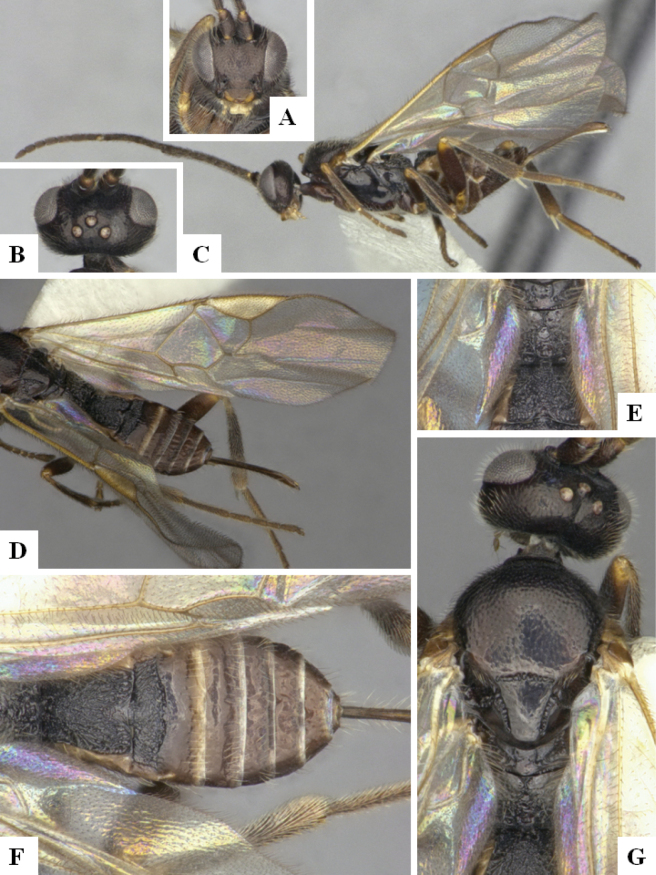
*Dolichogenidealacochaparamo* Fernandez-Triana & Boudreault holotype female CNC1180003 **A** head, frontal **B** head, dorsal **C** habitus, lateral **D** wings **E** propodeum & T1, dorsal **F** metasoma, dorsal **G** mesosoma, dorsal.

##### Diagnostic description.

T1 broadening towards posterior margin, 1.1× as long as width at posterior margin; T1 with strong, longitudinal striae on posterior 0.5; T2 mostly sculptured, with anterior and posterior margin weakly sinuate; ovipositor sheath approx. as long as metatibia; comparatively very dark colored species, with palpi yellow, tegula pale yellow-brown, humeral complex half yellow and half brown; most veins in fore wing yellow-white; pterostigma strongly yellow-white with very thin brown margins, most legs dark brown; body length: 2.80 mm; fore wing length: 3.10 mm. Among species with T1 and T2 sculptured (but with T2 transverse and without strong longitudinal striae), this species is characterized by its very dark coloration, pterostigma color and body size. The species *D.virgendelparamo* is morphologically similar but has darker colored palpi, tegula humeral complex and pterostigma, larger body size and less sculptured T2.

##### Distribution.

Colombia.

##### Biology.

No host data available.

##### DNA barcoding data.

No data.

##### Etymology.

Named after the type locality, which is within the La Cocha-Patascoy azonal paramo, an important area for the conservation of Andes species.

##### Notes.

The holotype label states Putumayo as the Department of the type locality. However, the coordinates provided in that same label would place the locality on the western side of the La Cocha Lagoon, which belongs to the Nariño Department. This discrepancy should be considered as a minor one because the locality is still very close (~ 10 kms apart) from the border between these two departments, and in fact the other (eastern) side of the La Cocha Lagoon belongs to the Putumayo. It could be a small inaccuracy of the GPS used to establish the original coordinates of the locality; an alternative explanation is that, between 1953 and 1957 Putumayo was fused with Nariño and was not elevated again as a Department until 1991 (https://es.wikipedia.org/wiki/Putumayo_(Colombia)). Regardless of that, the type locality clearly belongs to the area of the La Cocha-Patascoy azonal paramo, which straddles between the borders of those two departments (https://corponarino.gov.co/wp-content/uploads/2021/09/17.-D.A-PARAMO-LA-COCHA-PATASCOY.pdf).

#### ﻿ Dolichogenidea leahdennisae

Taxon classificationAnimaliaHymenopteraBraconidae

Fernandez-Triana & Boudreault sp. nov.

5F23DF56-E4CF-5843-9F87-4772465E1562

https://zoobank.org/FDF7A8DF-AAF9-4FAD-9DDF-E5DE2642DCD1

Fig. 86A–H

##### Type material.

***Holotype*.** Costa Rica • Female, CNC; Alajuela, Area de Conservación Guanacaste, Sector San Cristobal, Bosque Trampa Malaise; 10.8628, -85.3846; 815 m; 10.vi.2007; D. H. Janzen & W. Hallwachs leg.; Malaise trap; Voucher code: DHJPAR0026079.

**Figure 86.**
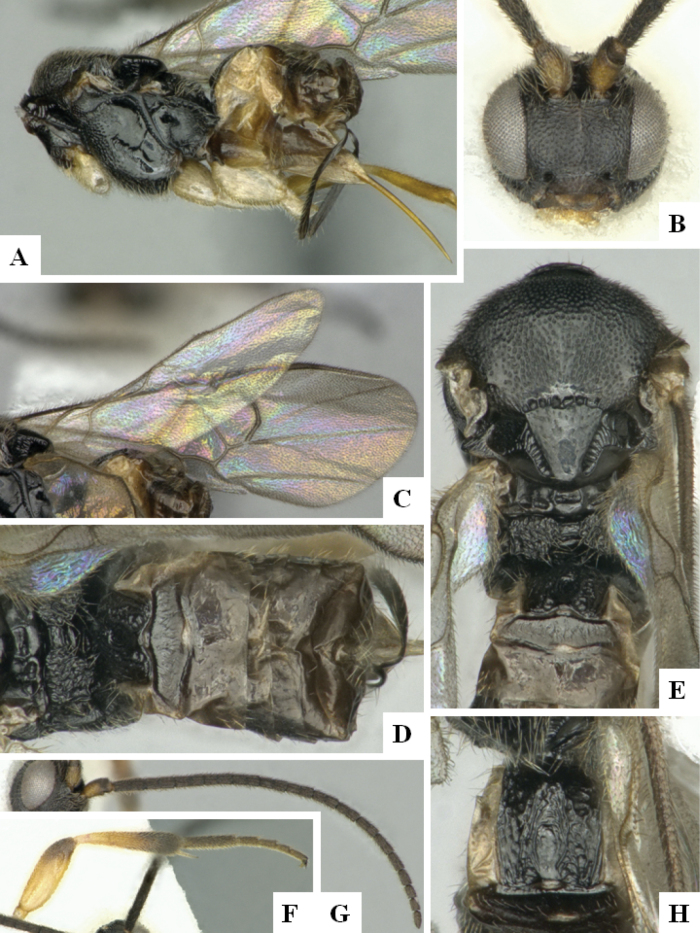
*Dolichogenidealeahdennisae* Fernandez-Triana & Boudreault holotype female DHJPAR0026079 **A** habitus, lateral **B** head, frontal **C** wings **D** metasoma, dorsal **E** mesosoma, dorsal **F** hind leg, lateral **G** antenna **H** T1–T2, dorsal.

##### Diagnostic description.

T1 parallel-sided or mostly parallel-sided, its length medially < 3.0× its width at posterior margin; T2 mostly smooth but with some sculpture along posterior margin; T2 comparatively more transverse, its length medially 4.0–5.0× its width at posterior margin; tegula and humeral complex dark brown; legs mostly pale (yellow), except for metacoxa with anterior 0.3 brown; body length: 2.80 mm; fore wing length: 3.00 mm. Among all species with smooth T2 and pale pro- and mesocoxae, *D.leahdennisae* can be distinguished by its T1 and T2 shape, T2 sculpture, color of tegula and humeral complex, and body size.

##### Distribution.

Costa Rica.

##### Biology.

No host data available.

##### DNA barcoding data.

BINBOLD:AAJ1396 (2 sequences, 2 barcode compliant).

##### Etymology.

Named after Leah Dennis in recognition of her many years of administrating the Biology Department office housing DHJ and WH at the University of Pennsylvania.

#### ﻿ Dolichogenidea limoncocha

Taxon classificationAnimaliaHymenopteraBraconidae

Fernandez-Triana & Boudreault sp. nov.

5D76EABE-B0FD-5787-9E60-60CEB7F180FD

https://zoobank.org/1B554407-F532-4C3A-833D-B56700AD1755

Fig. 87A–F

##### Type material.

***Holotype*.** Ecuador • Female, CNC; Napo, Limoncocha; 250 m; 15–28.vi.1976; S & J Peck leg.; CNC1179901.

**Figure 87.**
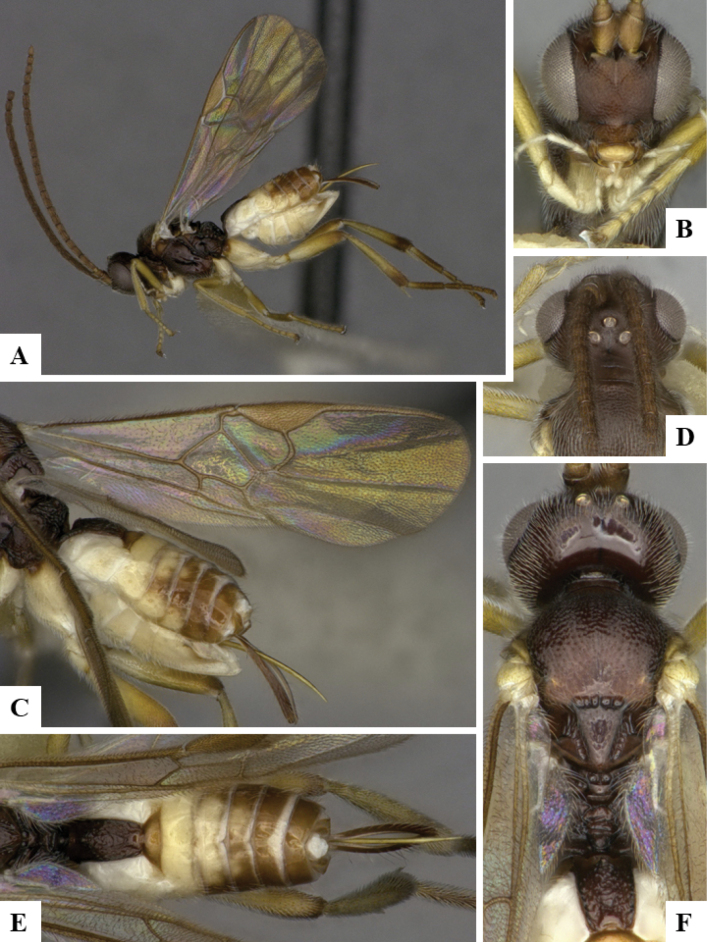
*Dolichogenidealimoncocha* Fernandez-Triana & Boudreault holotype female CNC1179901 **A** habitus, lateral **B** head, frontal **C** wings **D** head, dorsal **E** metasoma, dorsal **F** mesosoma, dorsal.

##### Diagnostic description.

Fore wing veins r and 2RS strongly angulate; T1 strongly sculptured; T1 more or less parallel-sided, only very slightly narrowing near posterior margin, > 2.5× as long as wide at posterior margin; T2 trapezoidal and mostly smooth; tegula and humeral complex yellow; all coxae yellow-white; metafemur mostly yellow-white with only posterior 0.1 brown; comparatively paler colored metasoma, with most laterotergites and all sternites and hypopygium yellow-white, T1 dark brown, T2 pale yellow-brown, and T3 (entirely), T4 (mostly), and T5 (partially, centrally) yellow to yellow-white; body length: 2.78 mm; fore wing length: 2.73 mm. A very distinctive species based on its rather unique pale color of metasoma (especially T4 and T5), pale colored legs, and T1 more or less parallel-sided.

##### Distribution.

Ecuador.

##### Biology.

No host data available.

##### DNA barcoding data.

No data.

##### Etymology.

Named after the type locality.

#### ﻿ Dolichogenidea luishamiltoni

Taxon classificationAnimaliaHymenopteraBraconidae

Fernandez-Triana & Boudreault sp. nov.

29F04156-9DDD-51B9-8A9D-94DD7B94F443

https://zoobank.org/83C2FD1A-6F4E-47C3-A09C-6250F07F1FA7

Fig. 88A–G

##### Type material.

***Holotype*.** Costa Rica • Female, CNC; Guanacaste, Area de Conservación Guanacaste, Sector San Cristobal, Bosque Transición; 10.86472, -85.41531; 540 m; 29.viii.2010; Gloria Sihezar leg.; Host: gelJanzen01 Janzen394; Voucher code: DHJPAR0042017; Host voucher code: 10-SRNP-4850. ***Paratypes*.** Costa Rica • 3 Females, CNC; DHJPAR0050116, DHJPAR0050174, DHJPAR0051148.

**Figure 88.**
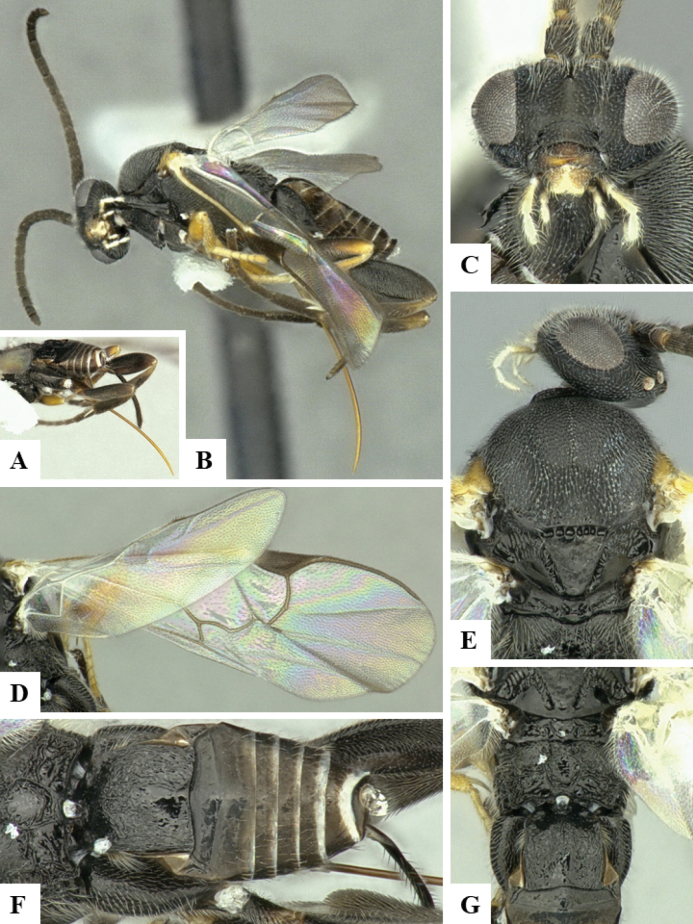
*Dolichogenidealuishamiltoni* Fernandez-Triana & Boudreault holotype female DHJPAR0042017 **A** metasoma, lateral **B** habitus, lateral **C** head, frontal **D** wings **E** mesosoma, dorsal **F** metasoma, dorsal **G** propodeum & T1–T2, dorsal.

##### Diagnostic description.

Posterior 0.5–0.6 of T1 mostly with strong sculpture, usually longitudinal striae; T1 slightly broadening posteriorly; T2 mostly smooth; T2 comparatively very transverse but with anterior margin arcuate; ovipositor sheath clearly longer (1.15–1.25×) than metatibia length; tegula and humeral complex yellow; coxae dark brown to black; trochantelli dark brown to black; metafemur dark brown; metatibia dark brown on posterior 0.8; body length: 2.81–2.88 mm; fore wing length: 3.13–3.28 mm. Among species with smooth T2 and metafemur dark, this species can be distinguished by T1 shape, ovipositor sheath length, and tegula, humeral complex and trochantelli color.

##### Distribution.

Costa Rica.

##### Biology.

Solitary. Gelechiidae, gelJanzen01 Janzen394.

##### DNA barcoding data.

BINBOLD:AAT8840 (9 sequences, 9 barcode compliant).

##### Etymology.

Named in honor of Mr. Luis Hamilton in recognition of his recent and ongoing support for the financial and psychological well-being of Area de Conservación Guanacaste (ACG) and its NGO Guanacaste Dry Forest Conservation Fund (GDFCF) for the GDFCF BioAlfa initiative.

#### ﻿ Dolichogenidea luzmariaromeroae

Taxon classificationAnimaliaHymenopteraBraconidae

Fernandez-Triana & Boudreault sp. nov.

2597EF13-668C-509E-A98D-47EEAC4BE58F

https://zoobank.org/7A853486-5929-4D0E-8B6C-AB087F1FEF7C

Figs 89A–F , 157B

##### Type material.

***Holotype*.** Costa Rica • Female, CNC; Alajuela, Area de Conservación Guanacaste, Sector Rincon Rain Forest, Sendero Anonas; 10.90528, -85.27882; 405 m; 02.vi.2011; Pablo Umana leg.; Host: phyBioLep01 BioLep758; Voucher code: DHJPAR0043141; Host voucher code: 11-SRNP-42637.

**Figure 89.**
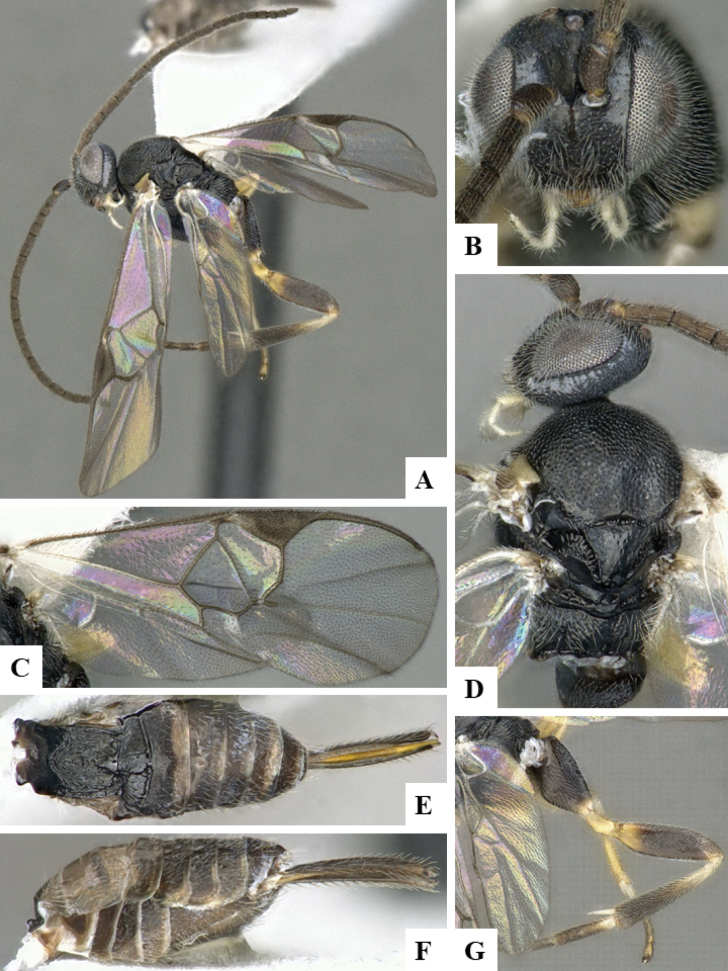
*Dolichogenidealuzmariaromeroae* Fernandez-Triana & Boudreault holotype female DHJPAR0043141 **A** habitus, lateral **B** head, frontal **C** wings **D** mesosoma, dorsal **E** metasoma, dorsal **F** metasoma, lateral **G** hind leg, lateral.

##### Diagnostic description.

Propodeum with complete areola; T1 1.7× as long as wide at posterior margin; T2 transverse, its width at posterior margin 3.5× its central length; T1 with strong sculpture on posterior 0.5; T2 mostly sculptured but with some smooth areas near posterior margin; ovipositor sheath 0.7× as long as metatibia length; tegula yellow, humeral complex half yellow half brown; all coxae, mesofemur and most of metafemur (except for anterior 0.2 which is yellow-white) brown to dark brown; body length: 2.45 mm; fore wing length: 2.63 mm. While *D.luzmariaromeroae* has T2 almost entirely sculptured (in that sense it would appear to run through the first half of couplet 9), there are smooth areas near posterior margin that are different from other species with sculptured T2. Additionally, this species can be distinguished by the color of its legs, tegula and humeral complex, length of ovipositor sheath, propodeum areola, and shape and sculpture of T1 and T2.

##### Distribution.

Costa Rica.

##### Biology.

Solitary. Pyralidae: Phycitinae, phyBioLep01 BioLep758.

##### DNA barcoding data.

BINBOLD:ABX5620 (3 sequences, 3 barcode compliant).

##### Etymology.

Named in honor of Sra. Luz Maria Romero, the primary technical guidance for the daily databasing and Species Pages done by the 30-member parataxonomist program of GDFCF for ACG, as well as her two decades of inventing the Programa de Educacion Biologica (PEB) for Area de Conservación Guanacaste.

#### ﻿ Dolichogenidea machupichu

Taxon classificationAnimaliaHymenopteraBraconidae

Fernandez-Triana & Boudreault sp. nov.

D80B9F68-B769-5A59-859D-BAAE3E332DBB

https://zoobank.org/2CD4D7C0-2A9E-4D82-B096-CB70350605A7

Fig. 90A–F

##### Type material.

***Holotype*.** Peru • Female, CNC; Cuzco, Machu Pichu; 2,400 m; 21.xii.1983; L. Huggert leg.; Voucher code: CNC1196529.

**Figure 90.**
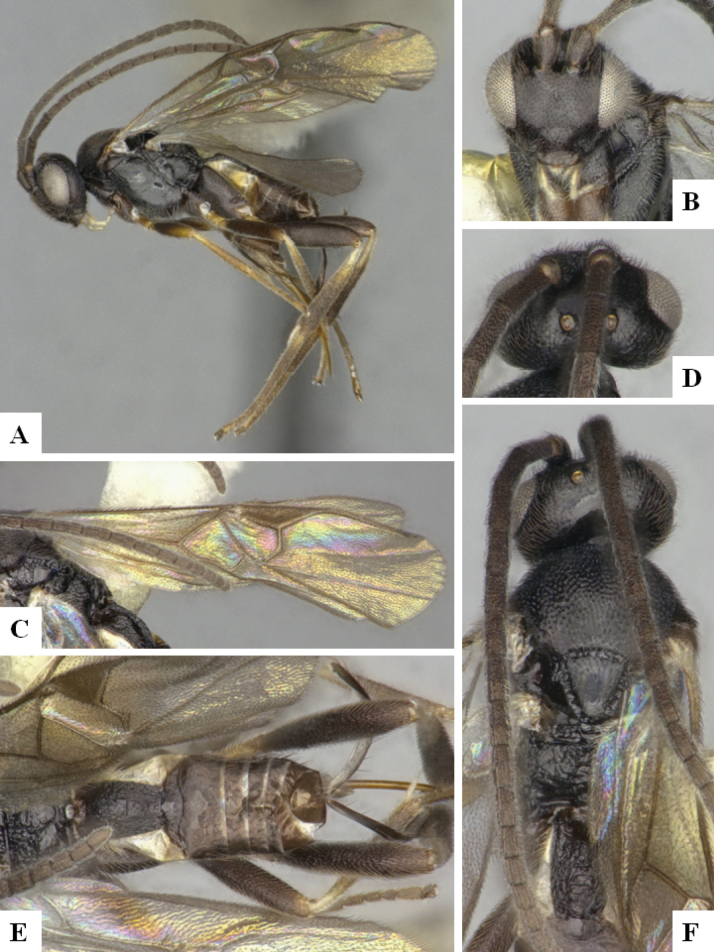
*Dolichogenideamachupichu* Fernandez-Triana & Boudreault holotype female CNC1196529 **A** habitus, lateral **B** head, frontal **C** wings **D** head, dorsal **E** metasoma, dorsal **F** mesosoma, dorsal.

##### Diagnostic description.

Propodeum with complete areola and more or less entirely sculptured on anterior half; T1 more or less parallel-sided but slightly narrowing on posterior 0.3; T1 mostly sculptured (but with central, depressed and smooth area); T2 transverse and more or less sculptured laterally, centrally smooth; ovipositor sheath approx. same length as metatibia; comparatively dark colored species, with tegula and humeral complex dark brown, all legs brown to dark brown (except for yellow protibia), tergites dark brown to black, all sternites and hypopygium dark brown; body length: 2.15 mm; fore wing length: 2.35 mm. Among all species with T1 and T2 sculptured (but neither entirely sculptured), *D.machupichu* can be recognized by the shape of T1 and its centrally depressed and smooth area, T2 centrally smooth, and the mostly dark coloration of legs, tegula, humeral complex, and metasoma.

##### Distribution.

Peru.

##### Biology.

No host data available.

##### DNA barcoding data.

No data.

##### Etymology.

Named after the locality where the holotype was collected.

#### ﻿ Dolichogenidea mariabustosae

Taxon classificationAnimaliaHymenopteraBraconidae

(Fernandez-Triana, 2016)

784A16E3-4E45-5BE7-B62A-A4C08BEC545D

Fig. 91A–G

##### Notes.

Full details for this species in Fernandez-Triana et al. (2016). See also the key and Table 1 above.

**Figure 91.**
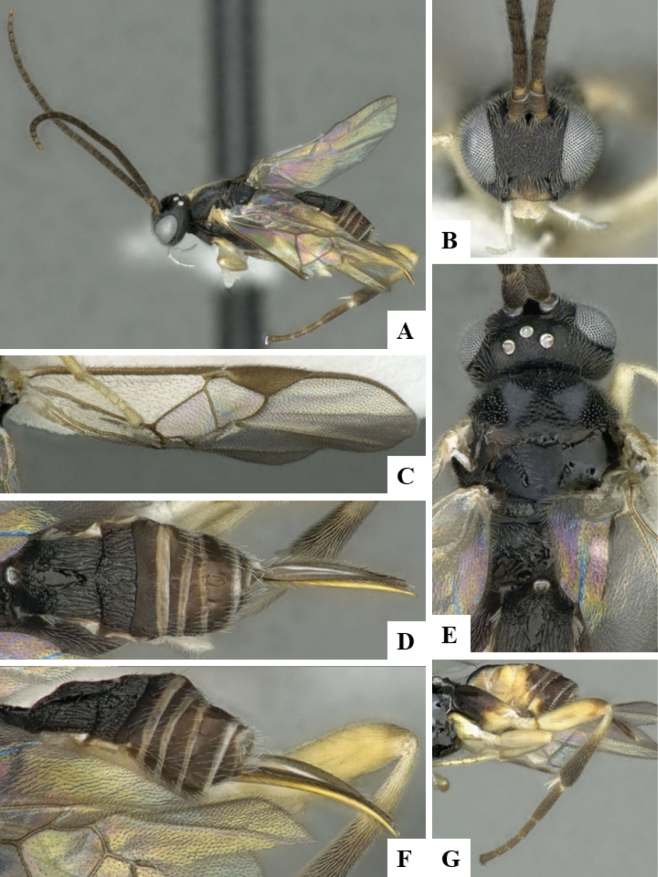
*Dolichogenideamariabustosae* (Fernandez-Triana) holotype female DHJPAR0048181 **A** habitus, lateral **B** head, frontal **C** fore wing **D** metasoma, dorsal **E** mesosoma, dorsal **F** metasoma, dorso-lateral **G** metasoma & hind leg, lateral.

#### ﻿ Dolichogenidea mehdirheljari

Taxon classificationAnimaliaHymenopteraBraconidae

Fernandez-Triana & Boudreault sp. nov.

52A4711D-7B87-5B70-8F18-D249C07AB164

https://zoobank.org/5D244F76-37EC-4C07-BB69-CCB2D8B56DB9

Fig. 92A–F

##### Type material.

***Holotype*.** Costa Rica • Female, CNC; Guanacaste, Area de Conservación Guanacaste, Sector Santa Rosa, Area Administrativa; 10.83764, -85.61871; 295 m; 25.xii.2008; D. H. Janzen & W. Hallwachs leg.; Malaise trap; Voucher code: DHJPAR0031851.

**Figure 92.**
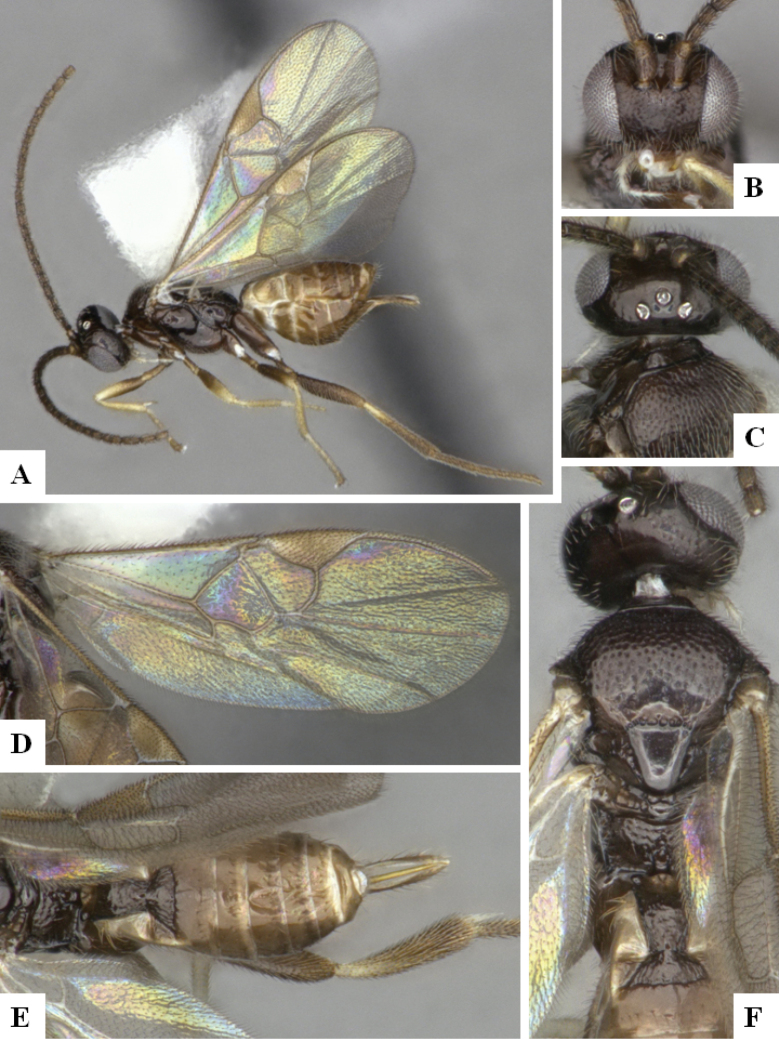
*Dolichogenideamehdirheljari* Fernandez-Triana & Boudreault holotype female DHJPAR0031851 **A** habitus, lateral **B** head, frontal **C** head, dorsal **D** wings **E** metasoma, dorsal, **F** mesosoma, dorsal.

##### Diagnostic description.

Overall body mostly shiny and smooth, including scutellar disc and most of propodeum (except for weak carinae defining a partial areola); T1 evenly narrowing from anterior to posterior margin and ~ 3.0× its width at posterior margin; T1 and T2 mostly smooth; hypopygium mostly inflexible, with only small, apical, reduced (one or two) pleats; ovipositor sheath 0.55× as long as metatibia length; legs mostly dark brown (except for tibiae and tarsi of first two pairs of legs); body length: 1.80 mm; fore wing length: 1.90 mm. Among all species with dark coxae and smooth or mostly smooth T1 and T2, *D.mehdirheljari* is distinguished by very short ovipositor sheath, shape of T1, and small body size.

##### Distribution.

Costa Rica.

##### Biology.

No host data available.

##### DNA barcoding data.

BINBOLD:AAM5852 (1 sequence, barcode compliant).

##### Etymology.

Named in honor of Mr. Mehdi Rheljari in recognition of his willingness to support a Malaise trap insect inventory of his tropical dry forest on his ecologically oriented hotel Kasiiya on the Nicoya coast of Guanacaste Province for 2022–2023.

#### ﻿ Dolichogenidea melaniamunozae

Taxon classificationAnimaliaHymenopteraBraconidae

Fernandez-Triana & Boudreault, 2019

38102B93-F510-50FC-85C0-41A08B371204

**Figure 93.**
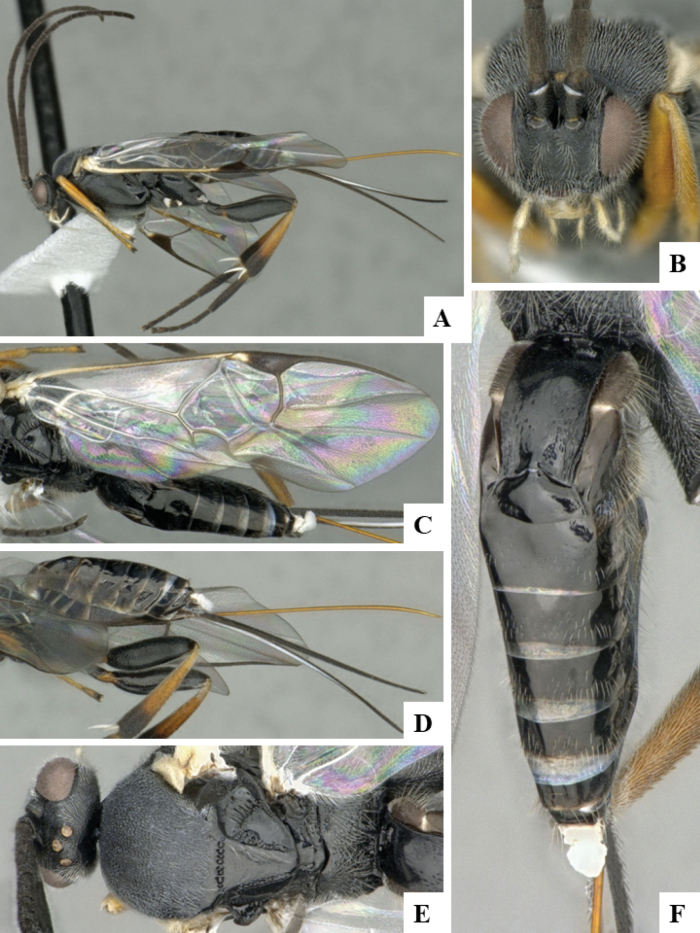
*Dolichogenideamelaniamunozae* Fernandez-Triana & Boudreault holotype female DHJPAR0051857 **A** habitus, lateral **B** head, frontal **C** wings **D** metasoma, lateral **E** mesosoma, dorsal **F** metasoma, dorsal.

Figs 93A–F , 158A, B

##### Notes.

Full details for this species in Fernandez-Triana et al. (2019). See also the key and Table 1 above.

#### ﻿ Dolichogenidea moniqueae

Taxon classificationAnimaliaHymenopteraBraconidae

Fernandez-Triana & Boudreault sp. nov.

BE46D085-8532-55AF-BF5F-2B26904DE6B2

https://zoobank.org/9CEE1E12-6F00-45A6-9A65-913DBC0F1FC9

Fig. 94A–F

##### Type material.

***Holotype*.** Chile • Female, CNC; Malleco, Cabreria, Cordillera de Nahuelbuta; 1100 m; i.1977; L. Pena leg.; Voucher code: CNC1180012.

**Figure 94.**
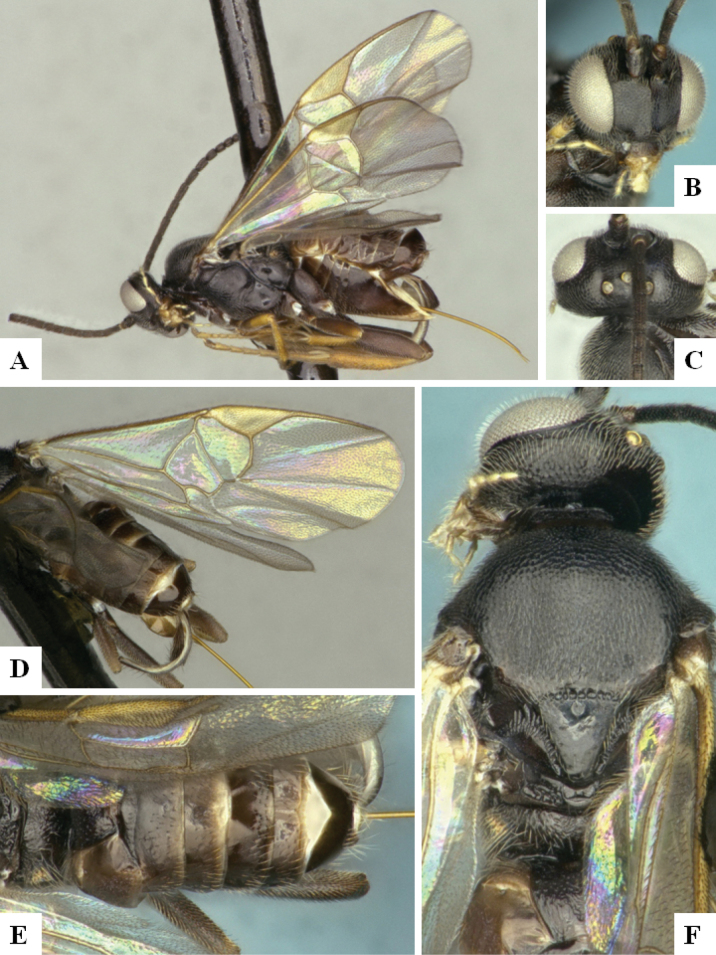
*Dolichogenideamoniqueae* Fernandez-Triana & Boudreault holotype female CNC1180012 **A** habitus, lateral **B** head, frontal **C** head, dorsal **D** wings **E** metasoma, dorsal **F** mesosoma, dorsal.

##### Diagnostic description.

Anteromesoscutum and scutellar disc mostly smooth and shiny; fore wing vein R1 longer than pterostigma and > 3.5× as long as the space between its end and end of vein 3RSb; T1parallel-sided on anterior half, narrowing towards posterior margin on posterior half, central length ~ 2.5× its width at posterior margin; T1 mostly smooth with weak punctures centrally and a polished knob centrally near posterior margin; T2 transverse, its width at posterior margin > 3.0× its central length; T2 entirely smooth; tegula and humeral complex dark brown; pterostigma bright yellow-white; all coxae, most of mesofemur and entire metafemur brown to dark brown, all tibiae and tarsi yellow to yellow-brown; body length: 2.55 mm; fore wing length: 2.58 mm. Among species with smooth T1 and T2 and comparatively paler coloration, *D.moniqueae* can be distinguished by the shape of T1, color of legs, pterostigma, tegula and humeral complex.

##### Distribution.

Chile.

##### Biology.

No host data available.

##### DNA barcoding data.

No data.

##### Etymology.

The second author dedicates this species to her late mother-in-law, Monique Laurendeau, who passed away in 2015. Monique is greatly missed and is remembered for her love of life and her huge smile.

#### ﻿ Dolichogenidea moniquegilbertae

Taxon classificationAnimaliaHymenopteraBraconidae

Fernandez-Triana & Boudreault sp. nov.

BA70B57A-29BE-5976-BB62-685AFC1456DF

https://zoobank.org/51A568AB-89BA-4D1A-86F3-1E966E74F146

Fig. 95A–F

##### Type material.

***Holotype*.** Costa Rica • Female, CNC; Guanacaste, Area de Conservación Guanacaste, Sector Santa Rosa, Area Administrativa; 10.83764, -85.61871; 295 m; 25.xii.2008; D. H. Janzen & W. Hallwachs leg.; Malaise trap; Voucher code: DHJPAR0031740.

**Figure 95.**
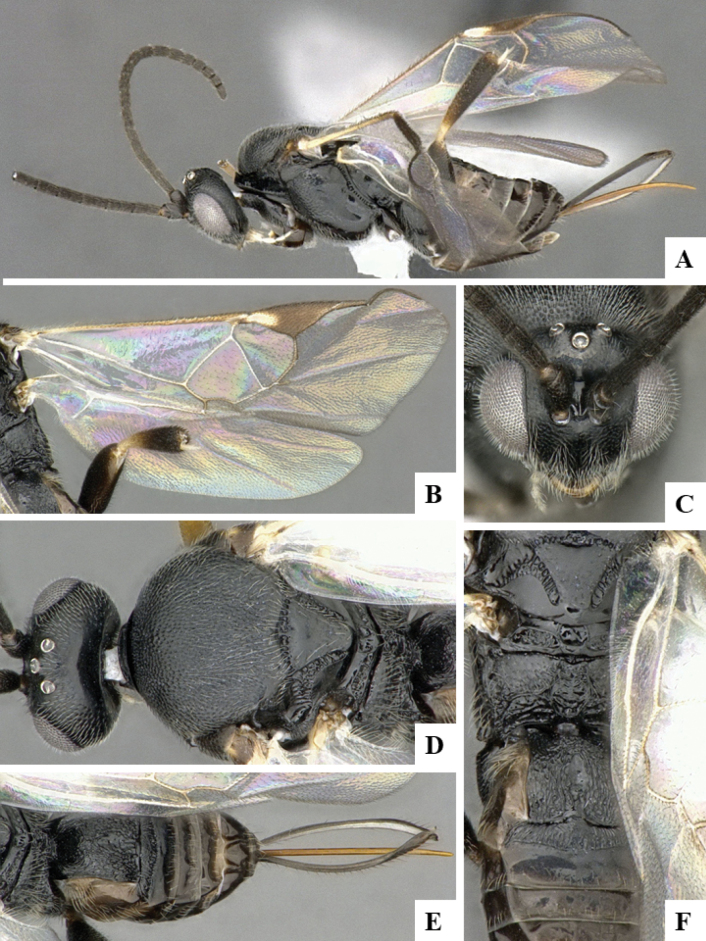
*Dolichogenideamoniquegilbertae* Fernandez-Triana & Boudreault holotype female DHJPAR0031740 **A** habitus, lateral **B** wings **C** head, frontal **D** mesosoma, dorsal **E** metasoma, dorsal **F** propodeum & T1–T4, dorsal.

##### Other material.

Mexico • Female, UNAM; BIOUG14382-G05.

##### Diagnostic description.

Pterostigma comparatively elongate, its length > 3.0× its maximum height; T1 strongly sculptured on posterior 0.5; T2 almost entirely sculptured; T2 very transverse, its width at posterior margin 4.0× its central length; ovipositor straight; pterostigma with pale spot 0.2–0.3 pterostigma length; most veins transparent to yellow white; all trochantelli dark brown, anterior 0.3–0.4 of profemur and entire mesofemur brown; all coxae, metafemur and most of metatibia (except for anterior 0.2 which is yellow) dark brown to black; body length: 2.58 mm; fore wing length: 2.45 mm. This species could be difficult to key out, especially on couplet 10 where the interpretation of T2 sculpture could lead to different alternatives. While *D.moniquegilbertae* has T2 almost entirely sculptured (in that sense it would appear to run through the first half of couplet 9), its shape is very different from all other species with entirely and strongly sculptured T2, as *D.moniquegilbertae* has T2 very transverse (width at posterior margin 4.0× its central length). That character, as well as color of legs, wing veins, and shape and color of pterostigma separate the species from similar ones.

##### Distribution.

Costa Rica, Mexico.

##### Biology.

No host data available.

##### DNA barcoding data.

BINBOLD:AAX8653 (11 sequences, 11 barcode compliant).

##### Etymology.

Named in honor of Mrs. Monique Gilbert of Vermont, USA, in recognition of her decade-plus of weathering the demands of being the two-country Development Officer for the NGO Guanacaste Dry Forest Conservation Fund and its integration with the Costa Rican government’s Area de Conservación Guanacaste (ACG) in northwestern Costa Rica.

##### Notes.

The record from Mexico is based on one sequence in BOLD which matches by 99.33–99.81% (1–4 bp of difference) with the ACG sequences. Because we could not study that specimen (other than examining a photo available in BOLD, which also matches well with the ACG specimen) it is not included as a paratype.

#### ﻿ Dolichogenidea monocavus

Taxon classificationAnimaliaHymenopteraBraconidae

(Valerio & Whitfield, 2004)

2AD43563-7A66-5529-A36D-0F5136E9C426

Fig. 96A, B

##### Notes.

Full details for this species in Valerio et al. (2004) and Fernandez-Triana et al. (2019). See also the key and Table 1 above.

**Figure 96.**
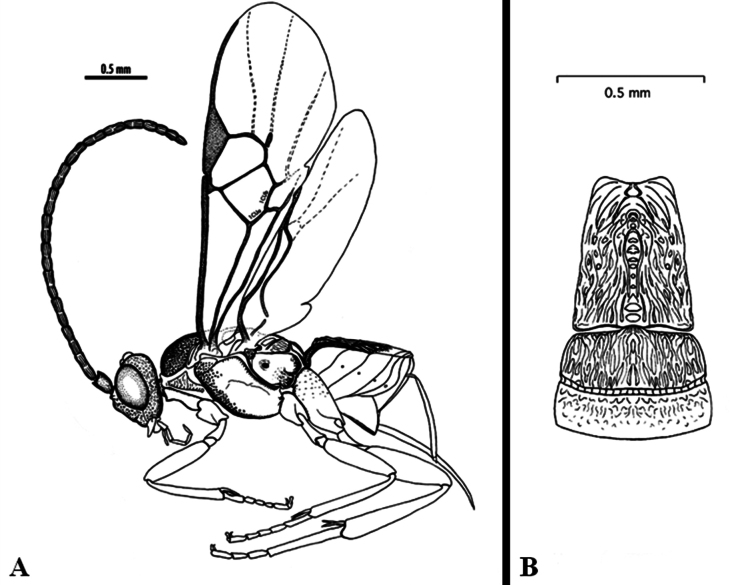
*Dolichogenideamonocavus* (Valerio & Whitfield) holotype female LN 250850-449250 **A** drawing of habitus, lateral **B** drawing of T1–T3, dorsal.

#### ﻿ Dolichogenidea ninamasisae

Taxon classificationAnimaliaHymenopteraBraconidae

Fernandez-Triana & Boudreault sp. nov.

00283EE0-2AE6-51F5-B78B-50ECCC8A18B7

https://zoobank.org/375614FC-ABF7-48E4-8854-DA3057D56CC8

**Figure 97.**
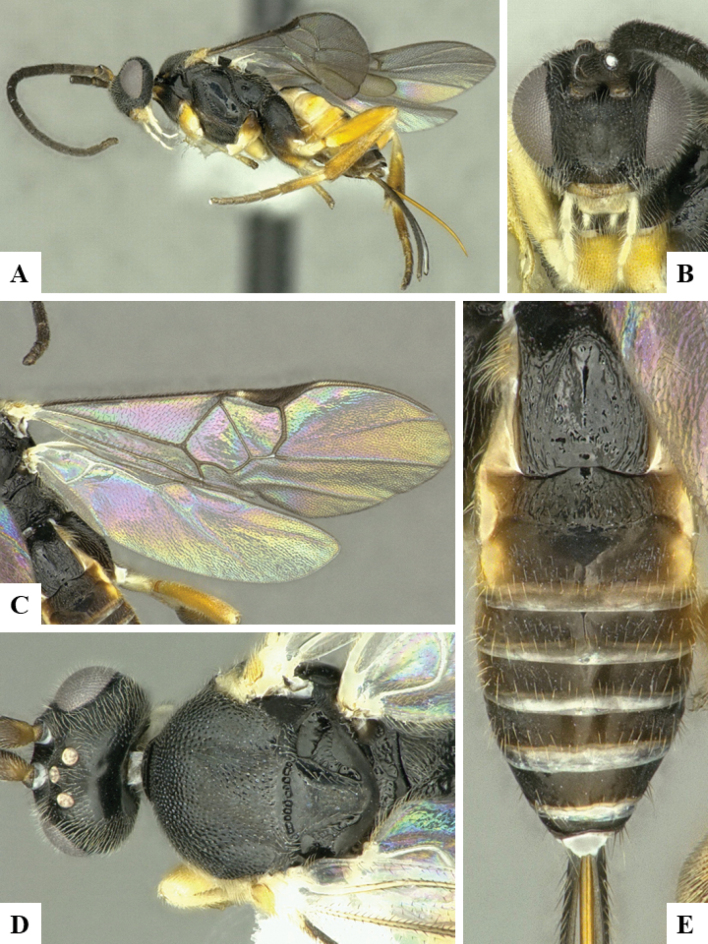
*Dolichogenideaninamasisae* Fernandez-Triana & Boudreault holotype female DHJPAR0049872 **A** habitus, lateral **B** head, frontal **C** wings **D** mesosoma, dorsal **E** metasoma, dorsal.

Figs 97A–E , 159A, B

##### Type material.

***Holotype*.** Costa Rica • Female, CNC; Alajuela, Area de Conservación Guanacaste, Sector San Cristobal, Sendero Perdido; 10.8794, -85.3861; 620 m; 4.vii.2012; G. Sihezar leg; Host: *Megalotaspinula*; Voucher code: DHJPAR0049872; Host voucher code: 12-SRNP-2554. ***Paratypes*.** Costa Rica • 3 Females, CNC; DHJPAR0049874, DHJPAR0049863, DHJPAR0054823.

##### Diagnostic description.

F2 length 2.5× F14 length; hind legs tarsal claws with single spine; T1 and T2 heavily sculptured with strong longitudinal striae; T1 length < 1.5× T1 width at posterior margin; T2 subrectangular, its width at posterior margin < 2.7× its central length; pterostigma mostly brown, with small pale spot on proximal 0.1–0.2; metacoxa almost entirely dark brown (very small yellow spot on posterior 0.1); metatibia mostly yellow with only dark brown to black spot on posterior 0.1; metatibial spurs entirely yellow; metatarsus mostly brown; body length: 3.10–3.30 mm; fore wing length: 3.03–3.16 mm. The color of pterostigma, metacoxa, metatibia, metatibial spurs, the length of F15 and the tarsal claws with a single spine separate *D.ninamasisae* from all other species with heavily sculptured T1 and T2, T1 comparatively broad and T2 rectangular and yellow metatibia. The only species closely similar morphologically is *D.tiboshartae* which has different color of metatarsus, different T2 shape, slightly smaller body size and slightly shorter ovipositor sheath.

##### Distribution.

Costa Rica.

##### Biology.

Solitary. Tortricidae: *Megalotacrassana*, *M.spinulosa*.

##### DNA barcoding data.

BINBOLD:AAY4690 (12 sequences, 12 barcode compliant).

##### Etymology.

Named in honor of Ms. Nina Masis in recognition of her robust participation in the family, country and conservation life of the Boshart-Masis household for the directorate of Area de Conservación Guanacaste (ACG).

#### ﻿ Dolichogenidea nothofagus

Taxon classificationAnimaliaHymenopteraBraconidae

Fernandez-Triana & Boudreault sp. nov.

8FC1A146-9275-50EE-B9F6-6D8AAB0AE6E1

https://zoobank.org/90E1D934-BC6C-44AC-8F4F-3E4CB75C793D

Fig. 98A–F

##### Type material.

***Holotype*.** Chile • Female, CNC; Ñuble, Las Trancas, *Nothofagus* forest; 1,700 m; 6.xii.1984–19.ii.1985; S. & J. Peck leg.; Voucher code: CNC1180107. ***Paratypes*.** Chile • 4 Females, CNC; CNC5342678, CNC5342679, CNC5342680, CNC5342681.

**Figure 98.**
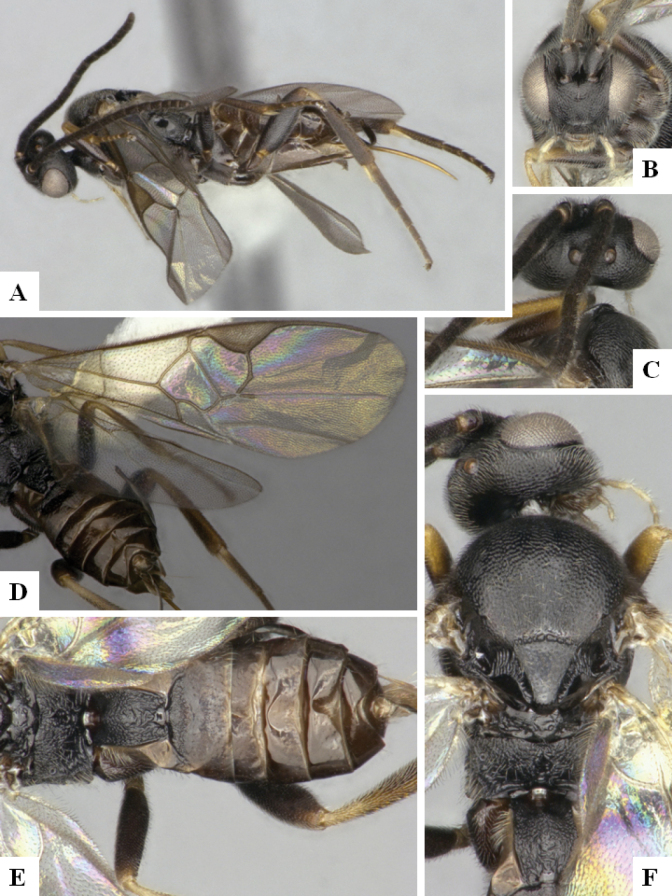
*Dolichogenideanothofagus* Fernandez-Triana & Boudreault holotype female CNC1180107 **A** habitus, lateral **B** head, frontal **C** head, dorsal **D** wings **E** metasoma, dorsal **F** mesosoma, dorsal.

##### Diagnostic description.

Propodeum mostly sculptured, especially on anterior half; T1 mostly sculptured and parallel-sided but narrowing on posterior 0.3; T2 mostly covered by weak sculptured (one of the paratype specimens from Malleco with T2 almost smooth); ovipositor sheath length ~ 1.2× metatibia length; comparatively dark colored species, with tegula and humeral complex dark brown, all legs brown to dark brown (except sometimes for yellow protibia), tergites dark brown to black, all sternites and hypopygium dark brown; body length: 1.95–3.03 mm; fore wing length: 2.03–2.72 mm. Among all species with T2 not strongly sculptured and transverse, this species can be recognized by dark coloration of body and legs, propodeum sculpture and length of the ovipositor sheath.

##### Distribution.

Chile.

##### Biology.

No host data available.

##### DNA barcoding data.

No data.

##### Etymology.

Named after the *Nothofagus* forest where the holotype and two paratypes were collected.

#### ﻿ Dolichogenidea oiketicus

Taxon classificationAnimaliaHymenopteraBraconidae

Fernandez-Triana & Boudreault sp. nov.

9B3AEF01-F97D-580C-BB3F-ED603635340B

https://zoobank.org/A6A468EB-A4FD-4E4A-AD80-6DCAAD16225D

Fig. 99A–F

##### Type material.

***Holotype*.** Trinidad & Tobago • Female, CNC; Curepe; 5.XII.1978; Malaise trap; Voucher code: CNC1180102. ***Paratype*.** Trinidad & Tobago • 1 Female, CNC1180028.

**Figure 99.**
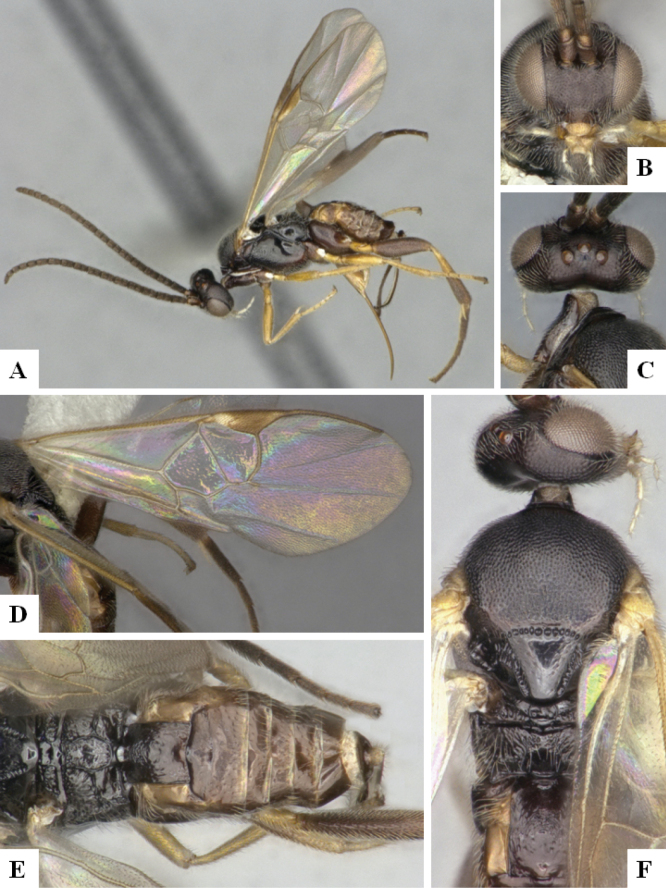
*Dolichogenideaoiketicus* Fernandez-Triana & Boudreault holotype female CNC1180102 **A** habitus, lateral **B** head, frontal **C** head, dorsal **D** wings **E** metasoma, dorsal **F** mesosoma, dorsal.

##### Diagnostic description.

Lunules triangular and comparatively very high; fore wing with vein r arising around middle of pterostigma; propodeum mostly with punctures and rugulosities and with complete and strongly defined areola; T1 parallel-sided and mostly smooth (only with fine sculpture laterally near posterior margin); T2 mostly to entirely smooth and comparatively very transverse, its width at posterior margin 4.0× its central length; tegula yellow, humeral complex yellow; pterostigma with pale spot on anterior 0.3; metafemur and posterior 0.5 of metatibia dark reddish brown; body color, including coxae, mostly dark reddish brown; body length: 2.18–2.30 mm; fore wing length: 2.50–2.55 mm. Among the species with smooth T1 and T2 and T2 transverse, this species is characterized by a combination of its coloration (of tegula, humeral complex, pterostigma, legs), for sculpture (of T1, T2, propodeum) and for high lunules in scutellum. *D.oiketicus* is similar to *D.hedylpetae* but it has a complete areola in the propodeum and the metafemur is darker (see other points mentioned in the key above). The holotype and paratype specimens were collected in the same locality (Curepe) where Cruttwell (1974) recorded *D.hedyleptae* as a parasitoid of *Oiketicuskirbyi* (Psychidae); those specimens were identified by Paul Marsh (USNM) but we believe they actually belong to *D.oiketicus*. Therefore, in this paper we restrict *D.hedyleptae* to Puerto Rico and consider it as a parasitoid of Pyralidae, whereas *D.oiketicus* is here considered to include specimens from Trinidad & Tobago and is a parasitoid of Psychidae. Additionally, *D.hedyleptae* is probably gregarious (based on info from Muesebeck 1958: 444) whereas *D.oiketicus* is solitary (Cruttwell 1974: 145).

##### Distribution.

Trinidad & Tobago.

##### Biology.

Solitary. Psychidae: *Oiketicuskirbyi* Guilding, 1827.

##### DNA barcoding data.

No data.

##### Etymology.

The name refers to the moth host genus that this wasp parasitizes (*Oiketicus*).

#### ﻿ Dolichogenidea palenque

Taxon classificationAnimaliaHymenopteraBraconidae

Fernandez-Triana & Boudreault sp. nov.

7237A0A9-0022-556A-BF31-FF6E3E36A157

https://zoobank.org/1D50E9E7-200B-4A73-BE8D-010F99E0B2CA

Fig. 100A–F

##### Type material.

***Holotype*.** Ecuador • Female, CNC; Pichincha, Centro científico Rio Palenque, 47 km South of Santo Domingo; 160 m; 30.iv-5.v.1987; L. D. Coote leg.; Voucher code: CNC1196947.

**Figure 100.**
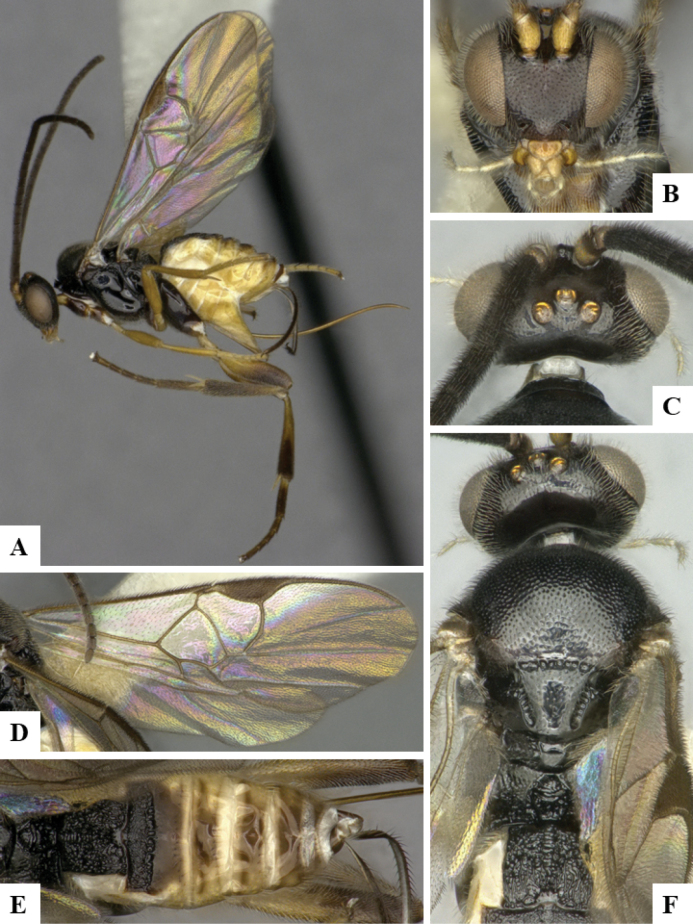
*Dolichogenideapalenque* Fernandez-Triana & Boudreault holotype female CNC1196947 **A** habitus, lateral **B** head, frontal **C** head, dorsal **D** wings **E** metasoma, dorsal **F** mesosoma, dorsal.

##### Diagnostic description.

Tarsal claws of hind legs with single spine-like basally; T1 and T2 heavily sculptured with strong longitudinal striae covering entire surface of T2 and most of T1; T1 broadening towards posterior margin; ovipositor sinuate; metafemur yellow on anterior half and brown on posterior half; metatibia dark brown to black on posterior 0.8; all laterotergites, sternites and hypopygium yellow; T3–T6 centrally brown, laterally with yellow spots; body length: 3.20 mm; fore wing length: 3.30 mm. Among all species with heavily sculptured T1 and T2, this species is distinctive by its legs and metasoma color, especially the yellow sternites and part of terga, as well as tarsal claw spine on hind legs.

##### Distribution.

Ecuador.

##### Biology.

No host data available.

##### DNA barcoding data.

No data.

##### Etymology.

Named after the type locality.

#### ﻿ Dolichogenidea papallacta

Taxon classificationAnimaliaHymenopteraBraconidae

Fernandez-Triana & Boudreault sp. nov.

328C1828-0DCA-5914-B655-DD641B18AA5F

https://zoobank.org/56AB4B39-6D68-49AE-85C5-1A05ED38E37D

Fig. 101A–G

##### Type material.

***Holotype*.** Ecuador • Female, CNC; Napo, Papallacta; 1,219 m; 14.ii.1983; L. Huggert leg.; Voucher code: CNC1180091.

**Figure 101.**
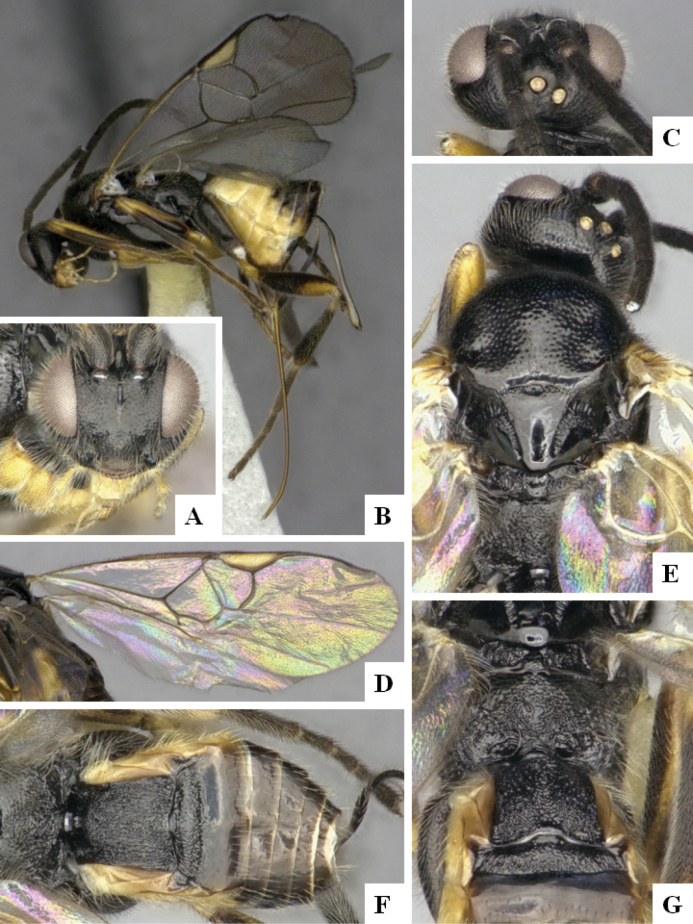
*Dolichogenideapapallacta* Fernandez-Triana & Boudreault holotype female CNC1180091 **A** head frontal **B** habitus, lateral **C** head, dorsal **D** wings **E** mesosoma, dorsal **F** metasoma, dorsal **G** propodeum & T1–T3, dorsal.

##### Diagnostic description.

T1 slightly broadening towards posterior margin, its length 1.2× its width at posterior margin; T1 almost entirely covered by coriaceous sculpture; T2 comparatively less transverse, its width at posterior margin 3.0× its central length; T2 mostly covered by longitudinal sculpture, but with two smoother, small areas near anterior margin centrally; pterostigma mostly yellow-white but with thin brown margins; procoxa yellow, meso- and metacoxae black; profemur yellow, mesofemur mostly yellow but with dark brown to black bands ventrally and dorsally for most of femur length, metafemur mostly dark brown to black, only with small area yellow (ventral and dorsal dark bands are so large that cover most of femur length); all tibia and tarsi dark brown to black; all sternites yellow, hypopygium yellow on anterior 0.5, dark brown on posterior 0.5; body length: 4.00 mm; fore wing length: 4.50 mm. The shape and sculpture of T1 and T2, pterostigma color, large body size and the rather unique coloration of legs and hypopygium are unique to this species and allow its clear recognition.

##### Distribution.

Ecuador.

##### Biology.

No host data available.

##### DNA barcoding data.

No data.

##### Etymology.

Named after the type locality in Ecuador.

#### ﻿ Dolichogenidea parallelis

Taxon classificationAnimaliaHymenopteraBraconidae

(Ashmead, 1900)

AC0DED9A-7048-54A7-B1E7-F2CF9F2C7784

Fig. 102A–D

##### Distribution.

Saint Vincent.

**Figure 102.**
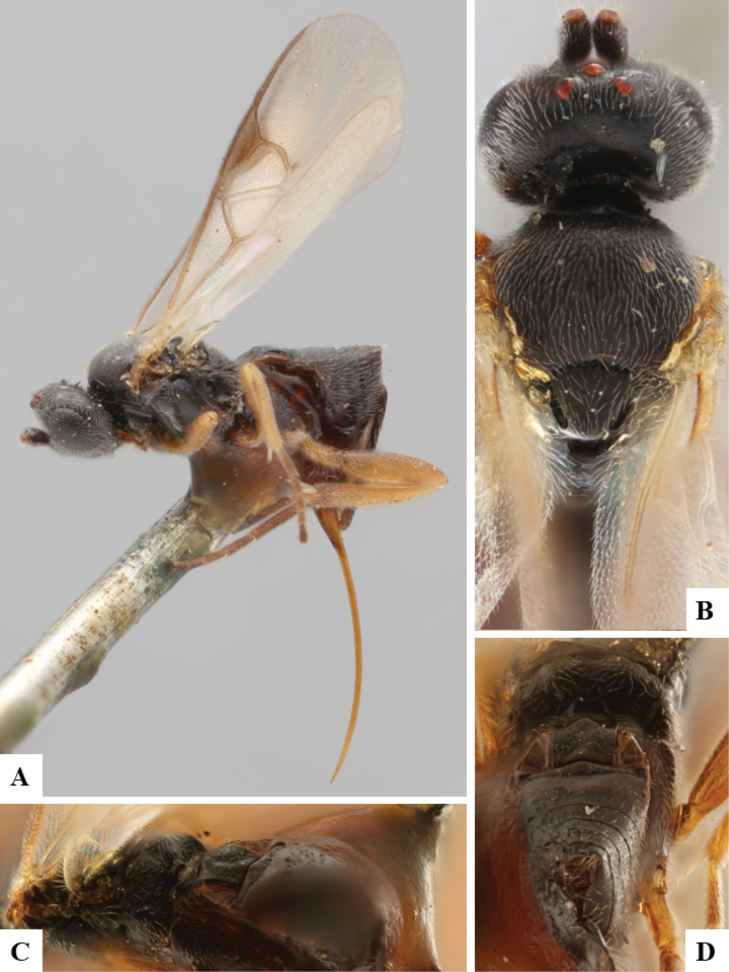
*Dolichogenideaparallelis* (Ashmead) holotype female **A** habitus, lateral **B** mesosoma, dorsal **C** propodeum and T1, dorso-lateral **D** metasoma, postero-dorsal.

##### Biology.

No host data available.

##### DNA barcoding data.

No data.

##### Notes.

This species was discussed and partially described in Fernandez-Triana et al. (2020). See also the key and Table 1 above.

#### ﻿ Dolichogenidea paulfryi

Taxon classificationAnimaliaHymenopteraBraconidae

Fernandez-Triana & Boudreault sp. nov.

78BB6466-6671-580A-B0AA-0ADEC7401BD9

https://zoobank.org/A32A10D0-D7F4-4B5F-8E54-409B34570A12

**Figure 103.**
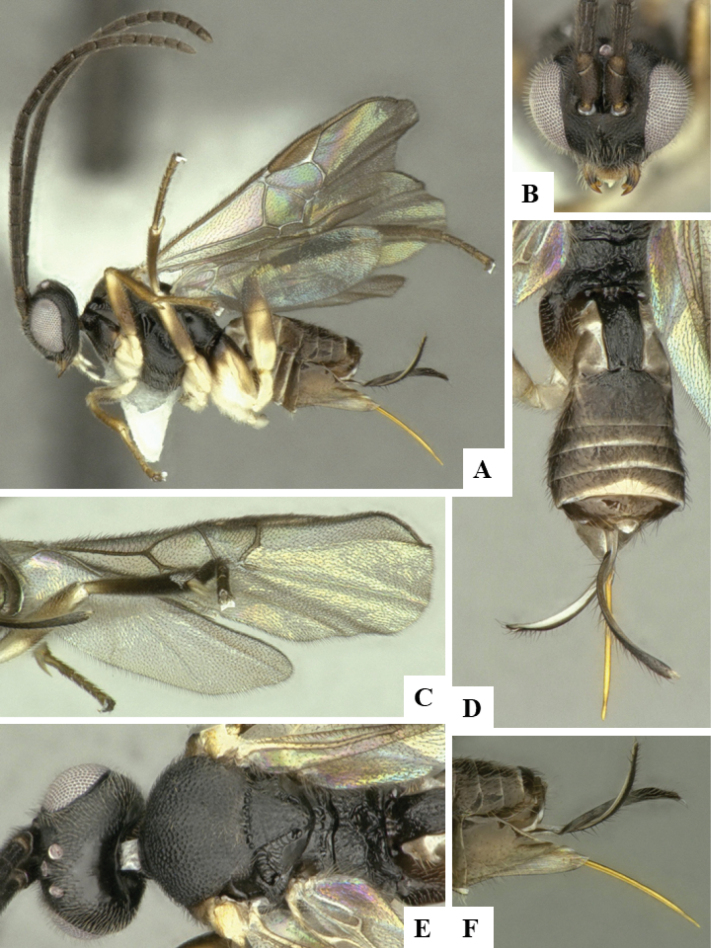
*Dolichogenideapaulfryi* Fernandez-Triana & Boudreault holotype female DHJPAR0025363 **A** habitus, lateral **B** head, frontal **C** wings **D** metasoma, dorsal **E** mesosoma, dorsal **F** ovipositor, lateral.

**Figure 104.**
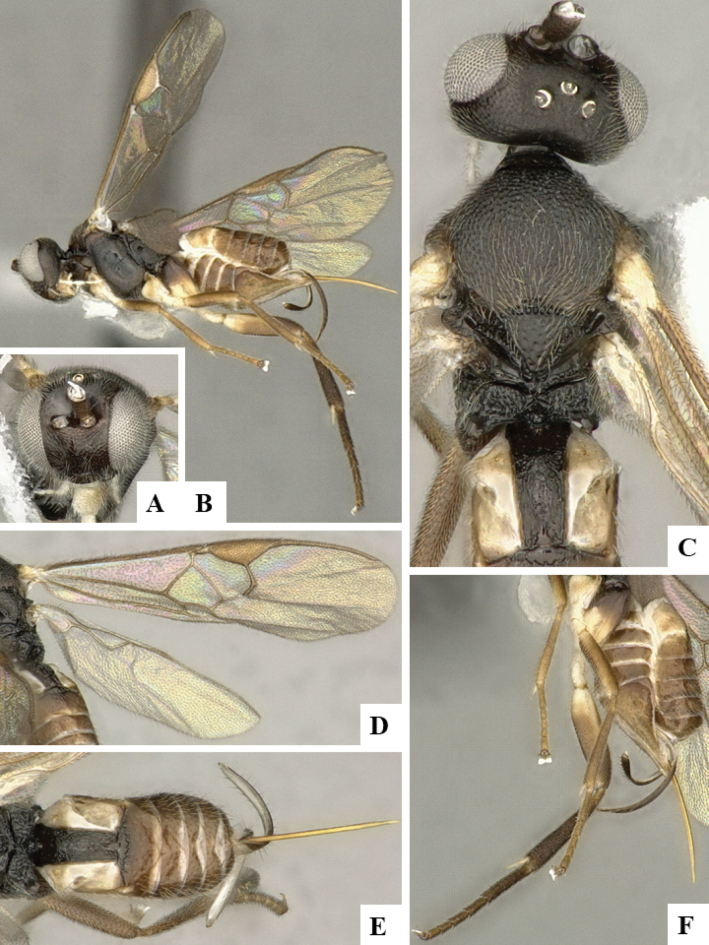
*Dolichogenideapaulfryi* Fernandez-Triana & Boudreault paratype female DHJPAR0012714 **A** head, frontal **B** habitus, lateral **C** mesosoma, dorsal **D** wings **E** metasoma, dorsal **F** metasoma, lateral.

**Figure 105.**
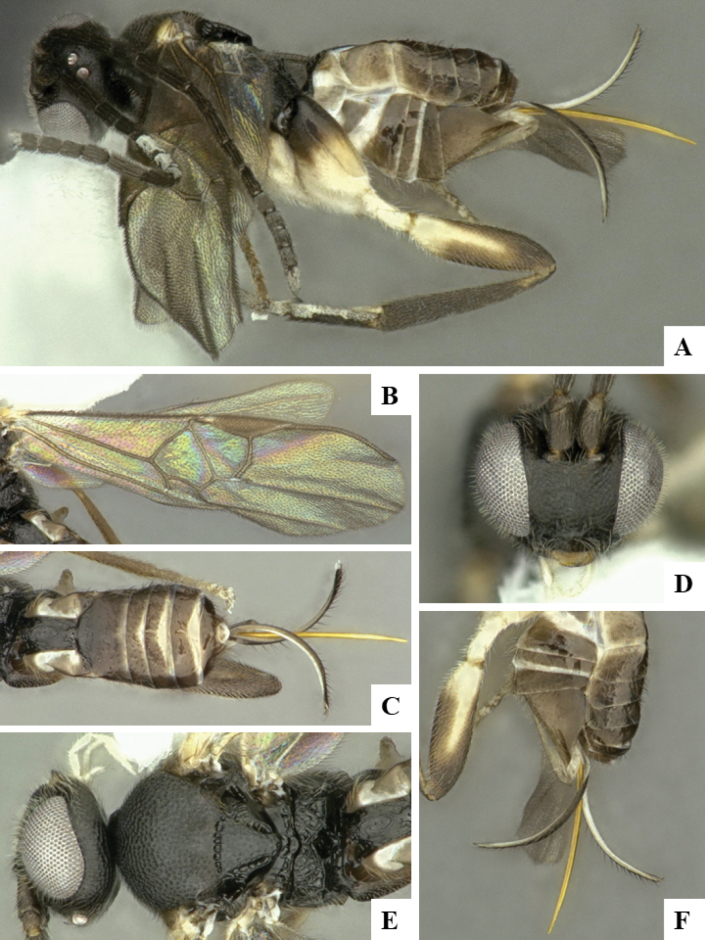
*Dolichogenideapaulfryi* Fernandez-Triana & Boudreault paratype female DHJPAR0025364 **A** habitus, lateral **B** wings **C** metasoma, dorsal **D** head, frontal **E** mesosoma, dorsal **F** metasoma, lateral.

Figs 103A–F , 104A–F , 105A–F

##### Type material.

***Holotype*.** Costa Rica • Female, CNC; Alajuela, Area de Conservación Guanacaste, Sector Rincon Rain Forest, Vado Rio Francia; 10.90093, -85.28915; 400 m; 26.xi.2007; D. H. Janzen & W. Hallwachs leg.; Malaise trap; Voucher code: DHJPAR0025363. ***Paratypes*.** Costa Rica • 2 Females, 1 Male, CNC; DHJPAR0012714, DHJPAR0013641, DHJPAR0025364.

##### Diagnostic description.

T1 comparatively narrow, its length medially ~ 3.0× its width at posterior margin; T2 with some sculpture around margins, centrally smooth; tegula brown, clearly darker than yellow humeral complex; all trochanters, pro- and mesocoxae white; metacoxa mostly white-yellow, with dark brown spot on anterior 0.2–0.3; metatibia entirely dark brown; body length: 2.08–2.18 mm; fore wing length: 2.30–2.38 mm. The shape of T1, partially sculptured T2, and color of tegula, humeral complex and legs differentiates *D.paulfryi* from other species with similar sculpture of T1 andT2 and pale pro- and mesocoxae.

##### Distribution.

Costa Rica.

##### Biology.

No host data available.

##### DNA barcoding data.

BINBOLD:ACF2929 (7 sequences, 7 barcode compliant).

##### Etymology.

Named after Paul Fry, an extremely helpful neighbor to DHJ and WH in Philadelphia, and the other half of the orange that is Anne Listerud.

#### ﻿ Dolichogenidea pedroleoni

Taxon classificationAnimaliaHymenopteraBraconidae

Fernandez-Triana & Boudreault sp. nov.

3DC4664A-9A2A-5991-8D03-DEE23E1EFEDF

https://zoobank.org/48A46E2F-61E3-4F48-828C-962EA8A96491

Figs 106A–F , 160A

##### Type material.

***Holotype*.** Costa Rica • Female, CNC; Alajuela, Area de Conservación Guanacaste, Sector San Cristobal, Bosque Trampa Malaise; 10.8628, -85.3846; 815 m; 16.vi.2007; D. H. Janzen & W. Hallwachs leg.; Malaise trap; Voucher code: DHJPAR0025998. ***Paratypes*.** Costa Rica • 11 Females, CNC; DHJPAR0012760, DHJPAR0004286, CNC5302042, CNC5302043 (there are more specimens in gel capsule attached to pin), CNC5302044, CNC5302045 (there are more specimens in gel capsule attached to pin), CNC5302046, CNC5302047 (there are more specimens in gel capsule attached to pin), CNC5302048 (there are more specimens in gel capsule attached to pin), CNC5302049, CNC5302050.

**Figure 106.**
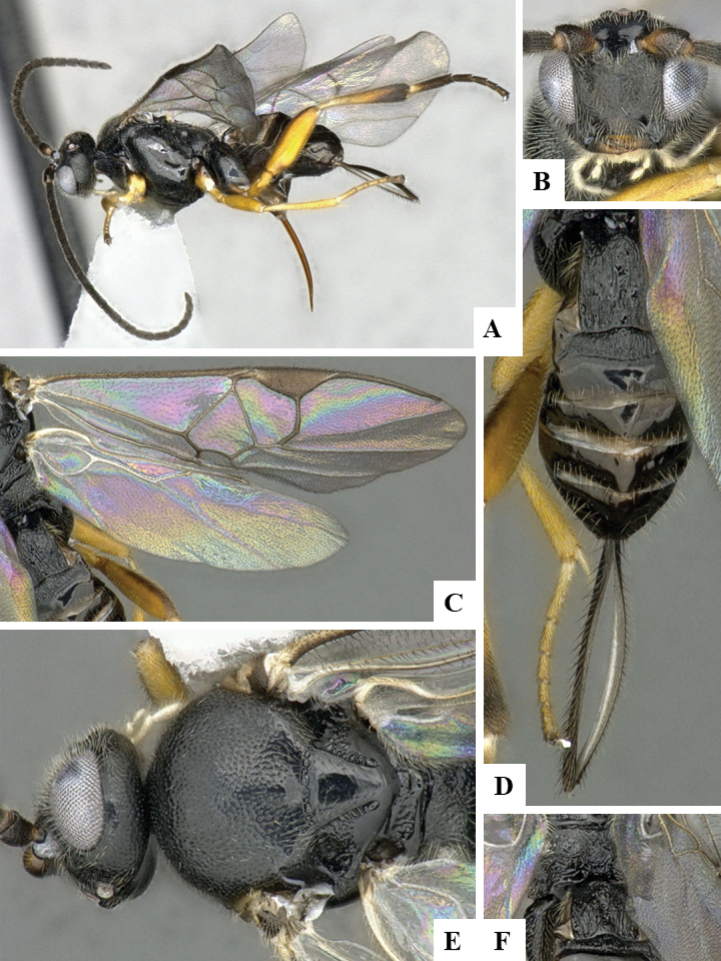
*Dolichogenideapedroleoni* Fernandez-Triana & Boudreault holotype female DHJPAR0025998 **A** habitus, lateral **B** head, frontal **C** wings **D** metasoma, dorsal **E** mesosoma, dorsal **F** propodeum & T1, dorsal.

##### Diagnostic description.

Scutellar disc smooth; anterior half of mesopleuron smooth; anteromesoscutum with sparse and relatively shallow punctures; propodeum areola comparatively broad (its height ~ 1.2× its central width) and open anteriorly; T1 and T2 heavily sculptured with strong longitudinal striae; T1 comparatively thin and mostly parallel-sided; T2, comparatively more transverse, its width at posterior margin 3.1× its central length; ovipositor sheath 1.1–1.2× metatibia length; ovipositor comparatively thicker, at least as thick as 0.8× flagellomeres width; tegula white-yellow, humeral complex mostly brown; pterostigma mostly pale brown with small, paler spot anteriorly; metacoxa entirely dark brown; metatibia mostly yellow, only posterior 0.2 or less dark brown; body length: 2.94–3.19 mm; fore wing length: 2.97–3.13 mm; BINBOLD:AAB4946, which is 2.08% different from the nearest BIN in BOLD as of March 2022. The coloration pattern, shape of T2, ovipositor length and width, and body size are distinctive among species with T1 and T2 heavily sculptured and T1 comparatively thin.

##### Distribution.

Costa Rica.

##### Biology.

Gregarious. Reared from a single species of Mimallonidae, *Eadmuna* Janzen01.

##### DNA barcoding data.

BINBOLD:AAB4946 (29 sequences, 28 barcode compliant).

##### Etymology.

Named in honor of Dr. Pedro Leon of San Jose, Costa Rica and of the Universidad de Costa Rica in honor of his decades of support for Area de Conservación Guanacaste in northwestern Costa Rica and for non-damaging biodevelopment of wild Costa Rican biodiversity.

#### ﻿ Dolichogenidea phthorimaeae

Taxon classificationAnimaliaHymenopteraBraconidae

(Muesebeck, 1921)

471AEE74-47B7-512E-823F-B4C65CCFAEE8

**Figure 107.**
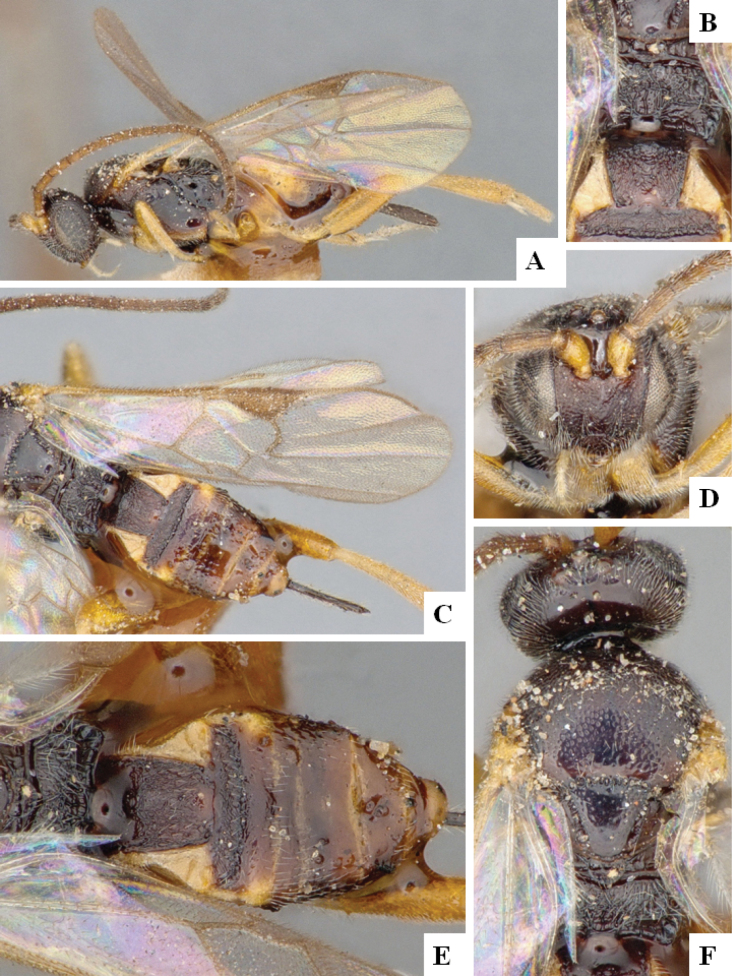
*Dolichogenideaphthorimaeae* (Muesebeck) female CNCHYM 01118 **A** habitus, lateral **B** propodeum & T1–T2, dorsal **C** wings **D** head, frontal **E** metasoma, dorsal **F** mesosoma, dorsal.

**Figure 108.**
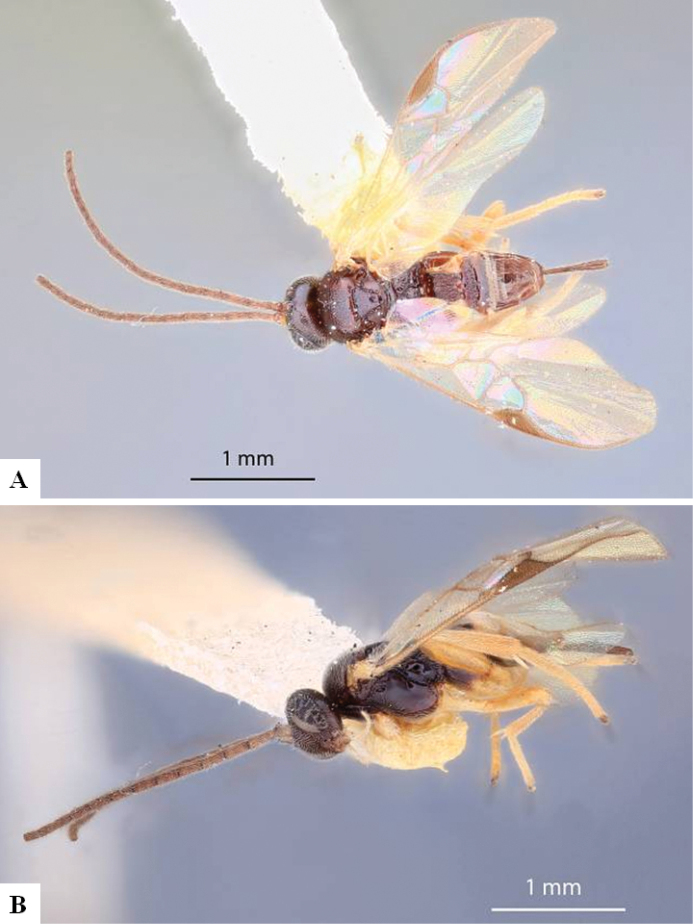
*Dolichogenideaphthorimaeae* (Muesebeck) holotype female **A** habitus, dorsal **B** habitus, lateral.

Figs 107A–F , 108A, B

##### Distribution.

Canada (ON), Honduras, United States (FL, LA).

##### Biology.

Solitary. Gelechiidae: *Keiferiaglochinella*, *Keiferiainconspicuella*, *Keiferialycopersicella*.

##### DNA barcoding data.

No data.

##### Notes.

See key and Table 1 above for more details on this species. Yu et al. (2016) did not list whether the parasitoid is solitary or gregarious, but from the original description (Muesebeck 1921) it is clear that it is solitary.

#### ﻿ Dolichogenidea politiventris

Taxon classificationAnimaliaHymenopteraBraconidae

Muesebeck, 1958

182082D3-D453-584B-82E6-0B019C0DF09A

**Figure 109.**
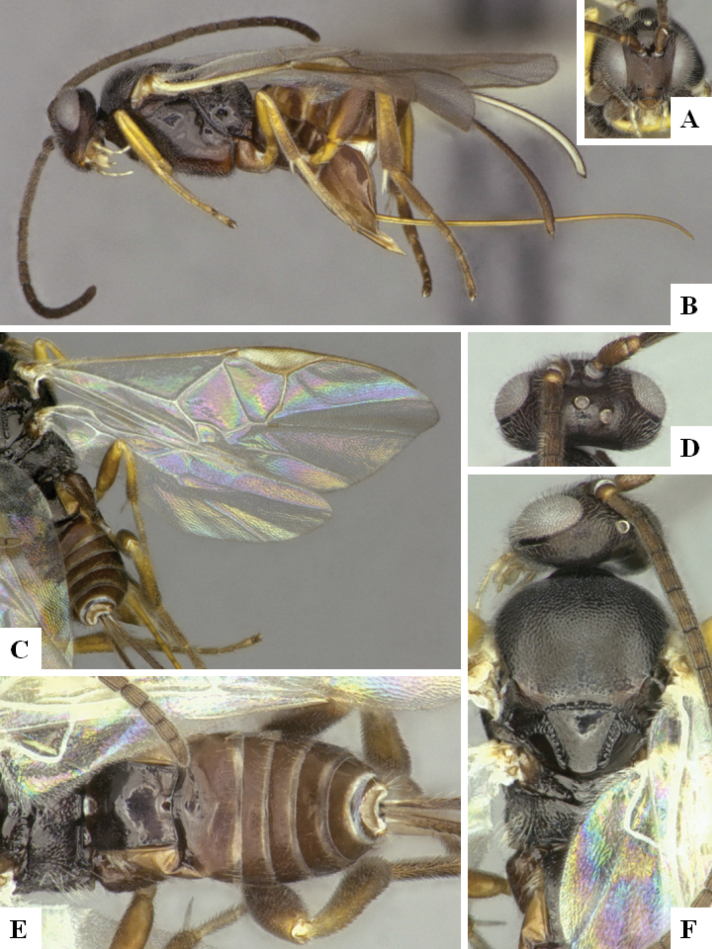
*Dolichogenideapolitiventris* (Muesebeck) female CNC1801957 **A** head, frontal **B** habitus, lateral **C** wings **D** head, dorsal **E** metasoma, dorsal **F** mesosoma, dorsal.

**Figure 110.**
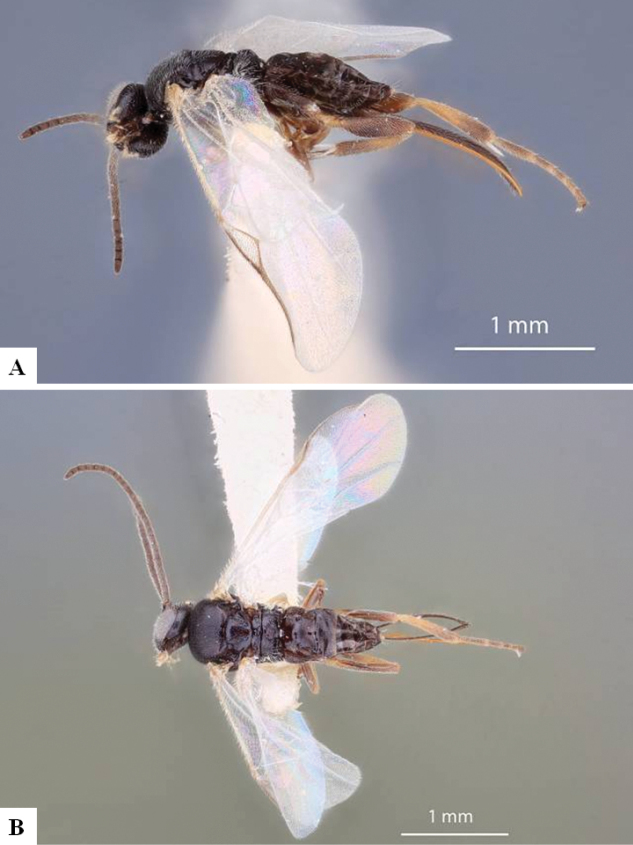
*Dolichogenideapolitiventris* (Muesebeck) holotype female **A** habitus, lateral **B** habitus, dorsal.

Figs 109A–F , 110A, B

##### Distribution.

Colombia, Dominican Republic, Puerto Rico, Saint Vincent, Trinidad & Tobago.

##### Biology.

No host data available.

##### DNA barcoding data.

No data.

##### Notes.

See key and Table 1 above for more details on this species. Previously, this species was only recorded from Puerto Rico. New country records reported here (Colombia, Dominican Republic, Saint Vincent, and Trinidad & Tobago) are based on CNC specimens.

#### ﻿ Dolichogenidea puschendorfi

Taxon classificationAnimaliaHymenopteraBraconidae

Fernandez-Triana & Boudreault sp. nov.

8898C653-DE18-5BEA-B988-9945EEA3A3BA

https://zoobank.org/874C477E-FC72-46B2-9807-C3040BFBD889

Fig. 111A–H

##### Type material.

***Holotype*.** Costa Rica • Female, CNC; Guanacaste, Area de Conservación Guanacaste, Sector Santa Rosa, Area Administrativa; 10.83764, -85.61871; 295 m; 25.xii.2008; D. H. Janzen & W. Hallwachs leg.; Malaise trap; Voucher code: DHJPAR0031864.

**Figure 111.**
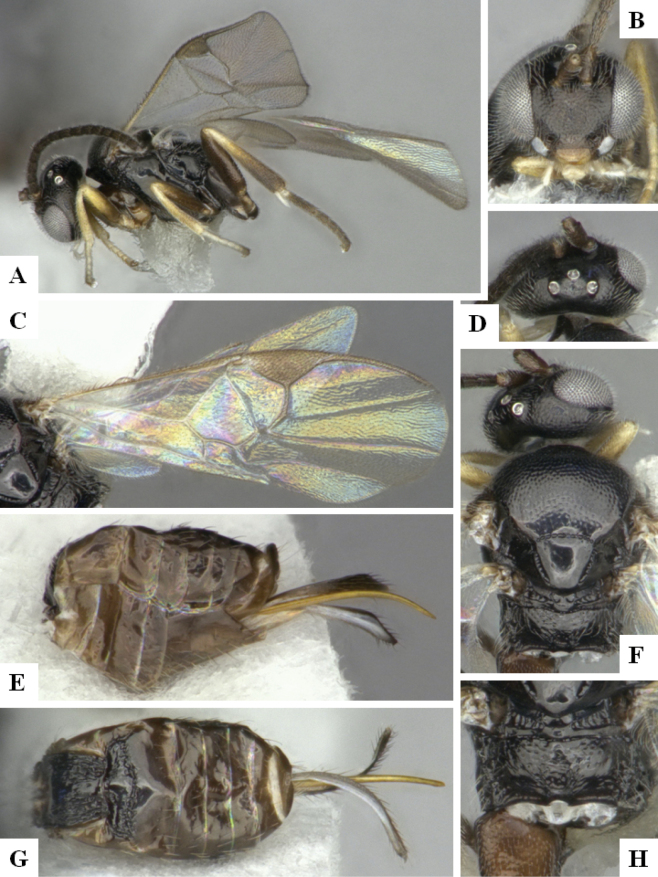
*Dolichogenideapuschendorfi* Fernandez-Triana & Boudreault holotype female DHJPAR0031864 **A** habitus, lateral **B** head, frontal **C** wings **D** head, dorsal **E** metasoma, lateral **F** mesosoma, dorsal **G** metasoma, dorsal **H** propodeum, dorsal.

##### Diagnostic description.

Propodeum areola open anteriorly, propodeum generally with relatively few sculpture; T1 with some sculpture on posterior 0.5; T2 mostly smooth, with few punctures near posterior margin; ovipositor sheath 0.8× as long as metatibia length; tegula and humeral complex brown; all coxae, metafemur and part of metatibia (except for anterior 0.5 yellow-white) brown; body length and fore wing length: 1.80 mm. This is the only Neotropical species of *Dolichogenidea* known to have a white spot on gena. Among species with T1 and T2 mostly smooth, *D.puschendorfi* can be distinguished by its small size, propodeum sculpture and poorly defined areola, length of ovipositor sheath, and color of tegula, humeral complex and legs.

##### Distribution.

Costa Rica.

##### Biology.

No host data available.

##### DNA barcoding data.

BINBOLD:AAM5853 (6 sequences, 6 barcode compliant).

##### Etymology.

Named in honor of Dr. Robert Puschendorf of Plymouth University, UK, and Costa Rica, in recognition of his two decades and ongoing support of the biodiversity growth and survival of Area de Conservación Guanacaste and the GDFCF support of the same.

#### ﻿ Dolichogenidea putumayo

Taxon classificationAnimaliaHymenopteraBraconidae

Fernandez-Triana & Boudreault sp. nov.

B7A56788-EE88-5833-8A14-097F2A64C26E

https://zoobank.org/68ED496C-581F-4611-A910-346A1BD84571

Fig. 112A–E

##### Type material.

***Holotype*.** Colombia • Female, CNC; Putumayo, 1°10'N, 76°45'W; 1,350 m; 1.xii.1972; J. Helava leg.; CNC1196560.

**Figure 112.**
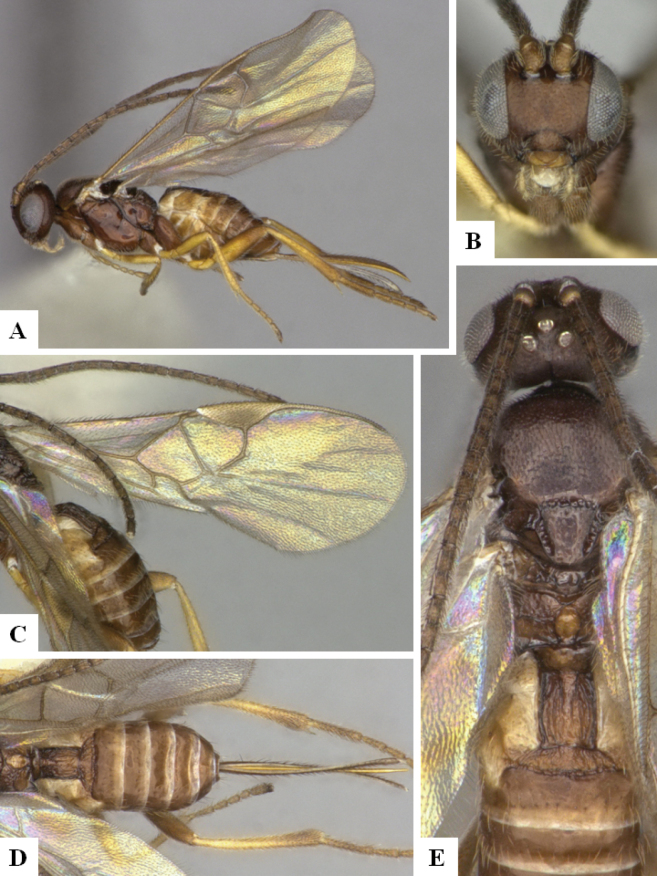
*Dolichogenideaputumayo* Fernandez-Triana & Boudreault holotype female CNC1196560 **A** habitus, lateral **B** head, frontal **C** wings **D** metasoma, dorsal **E** mesosoma, dorsal.

##### Diagnostic description.

Propodeum mostly smooth, with comparatively tall and thin areola that occupies the entire length of propodeum and it is completely defined by carinae; posterior 0.5 of T1 and entire T2 sculptured with strong, longitudinal striae; T1 length 2.0× its width at posterior margin; T2 width at posterior margin 3.7× its length medially; ovipositor sheath 1.5× metatibia length; body color mostly pale reddish brown; pterostigma with small pale yellow with white spots on anterior 0.1 and posterior 0.1; body length: 2.00 mm; fore wing length: 2.20 mm. Among species with T2 strongly sculptured but transverse, *D.putumayo* can be recognized by body and pterostigma color, T1 and T2 shape, ovipositor sheath length and shape and definition of propodeal areola.

##### Distribution.

Colombia.

##### Biology.

No host data available.

##### DNA barcoding data.

No data.

##### Etymology.

Named after the Colombian department where the type locality of the species is found and the Putumayo river, which crosses this biodiverse area.

#### ﻿ Dolichogenidea puyo

Taxon classificationAnimaliaHymenopteraBraconidae

Fernandez-Triana & Boudreault sp. nov.

D03C4532-5040-5293-A716-513211260243

https://zoobank.org/04CF31F6-1201-4F2D-A0FB-72DA22DBF276

Fig. 113A–F

##### Type material.

***Holotype*.** Ecuador • Female, CNC; Pastaza, 25 km N of Puyo; 1,000 m; 4.vii.1976; S. & J. Peck leg.; Voucher code: CNC1180168.

**Figure 113.**
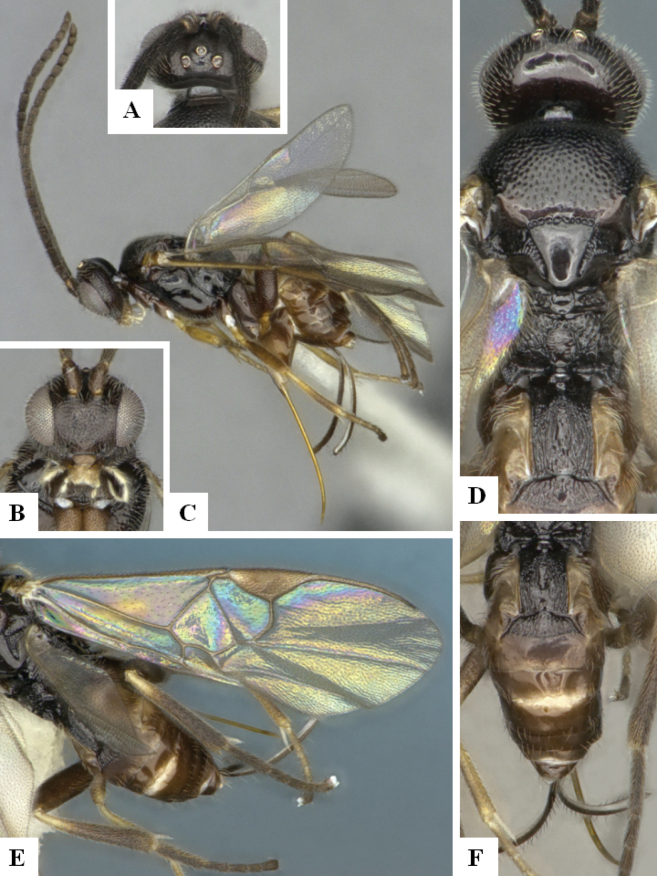
*Dolichogenideapuyo* Fernandez-Triana & Boudreault holotype female CNC1180168 **A** head, dorsal **B** head, frontal **C** habitus, lateral **D** mesosoma, dorsal **E** wings **F** metasoma, dorsal.

##### Diagnostic description.

Scutellar disc smooth; propodeum sculptured on anterior half and with complete areola; T1 rectangular and ~ 2.0× as long as wide; T1 with strong longitudinal striae on most of its surface; T2 transverse, its width at posterior margin ~ 3.0× as central length; T2 with some longitudinal striae but centrally smooth and shiny; ovipositor sheath clearly longer than metatibia length (~ 1.15× its length); tegula yellow, humeral complex half yellow half brown; pterostigma with pale spot at base; all coxae brown; profemur and protibia yellow; pro- and mesotrochantelli yellow; metafemur entirely brown; metatibia dark brown on posterior 0.8, with anterior 0.2 yellow to yellow-brown; body length: 2.00 mm; fore wing length: 2.24 mm. Among species with T2 transverse and with T1 and T2 sculptured (but T2 not entirely so) and with dark coxae, this species can be distinguished by the color pattern of the first two pairs of legs, color of tegula and humeral complex, and pterostigma with pale spot at base.

##### Distribution.

Ecuador.

##### Biology.

No host data available.

##### DNA barcoding data.

No data.

##### Etymology.

Named after the locality where the holotype was collected.

#### ﻿ Dolichogenidea rexhamiltoni

Taxon classificationAnimaliaHymenopteraBraconidae

Fernandez-Triana & Boudreault sp. nov.

79F594D8-C8BB-55BB-B471-8738DA0C352C

https://zoobank.org/A599DE3A-55E2-4A8B-97B3-8EFAE3E59839

Fig. 114A–F

##### Type material.

***Holotype*.** Costa Rica • Female, CNC; Guanacaste, Area de Conservación Guanacaste, Sector Cacao, Sendero Arenales; 10.92471, -85.46738; 1,080 m; 18.xii.2008; D. H. Janzen & W. Hallwachs leg.; Malaise trap; Voucher code: DHJPAR0031362.

**Figure 114.**
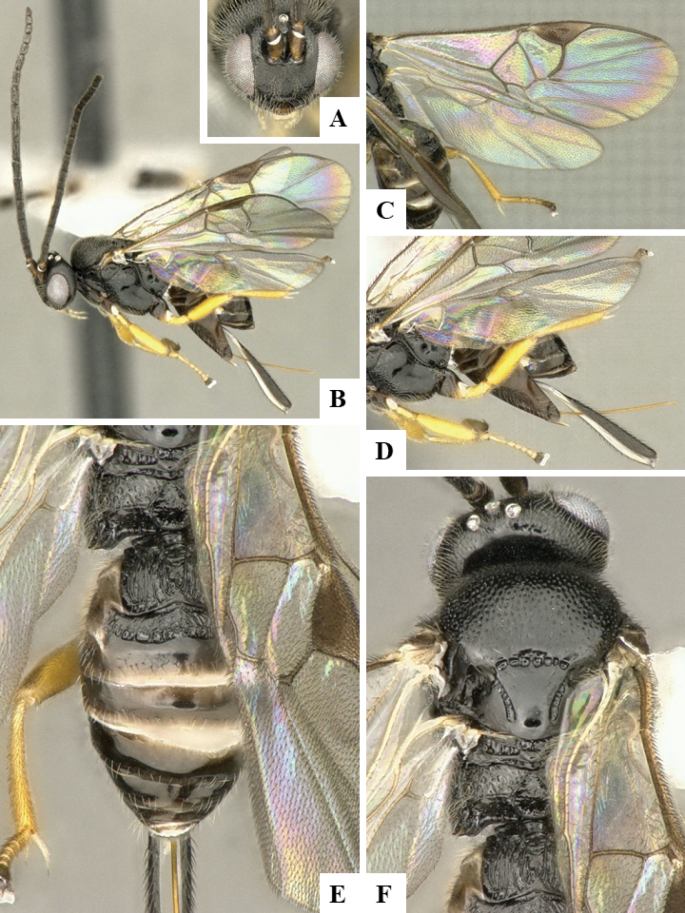
*Dolichogenidearexhamiltoni* Fernandez-Triana & Boudreault holotype female DHJPAR0031362 **A** head, frontal **B** habitus, lateral **C** wings **D** metasoma, lateral **E** metasoma, dorsal **F** mesosoma, dorsal.

##### Diagnostic description.

Posterior 0.5–0.6 of T1 and T2 mostly with strong sculpture, usually longitudinal striae covering entire surface (but T2 with small polished area centrally); T1 parallel-sided to slightly broadening posteriorly; T2 comparatively very transverse but with anterior and posterior margins strongly arcuate, so that T2 length is longer medially than laterally and thus T2 width at posterior margin is usually < 3.0× its length medially; ovipositor ≤ 1.4× as metatibia length; tegula and humeral complex yellow; pterostigma usually without pale spot at base or with small pale spot occupying < 0.1 pterostigma length; most laterotergites, some sternites and sometimes hypopygium yellow to yellow-brown; body length: 2.23 mm; fore wing length: 2.55 mm. This species has strong sculpture (usually longitudinal striae) covering posterior 0.5–0.6 of T1 and most of T2. However, unlike the majority of species with similarly strong sculpture, T2 has a central area which is smooth and also T2 is very transverse and with anterior and posterior margins strongly arcuate. Because of that unique shape and sculpture pattern of T2, as well as its metafemur color, it can be separate from all the species with entirely and strongly sculptured T2 which is not transverse, as well as all the species with smooth T2 and/or broad T2. Among similar species, *D.rexhamiltoni* can be distinguished from *D.anniapicadoae* because of its much shorter ovipositor, and from *D.jorgecarvajali* because of different coloration of tegula, humeral complex, laterotergites, sternites and hypopygium.

##### Distribution.

Costa Rica.

##### Biology.

No host data available.

##### DNA barcoding data.

BINBOLD:AAL2287 (2 sequences, 2 barcode compliant).

##### Etymology.

Named in honor of Mr. Rex Hamilton of Florida, USA and Guanacaste Province, Costa Rica in recognition of his recent and ongoing support for the financial and psychological well-being of Area de Conservación Guanacaste (ACG) and its NGO Guanacaste Dry Forest Conservation Fund (GDFCF) for the GDFCF BioAlfa initiative.

#### ﻿ Dolichogenidea richardashleyi

Taxon classificationAnimaliaHymenopteraBraconidae

(Fernandez-Triana, 2016)

E2CBDD41-9FD1-5F96-86EF-55E8540E1BFB

Fig. 115A–F

##### Notes.

Full details for this species in Fernandez-Triana et al. (2019). See also the key and Table 1 above.

**Figure 115.**
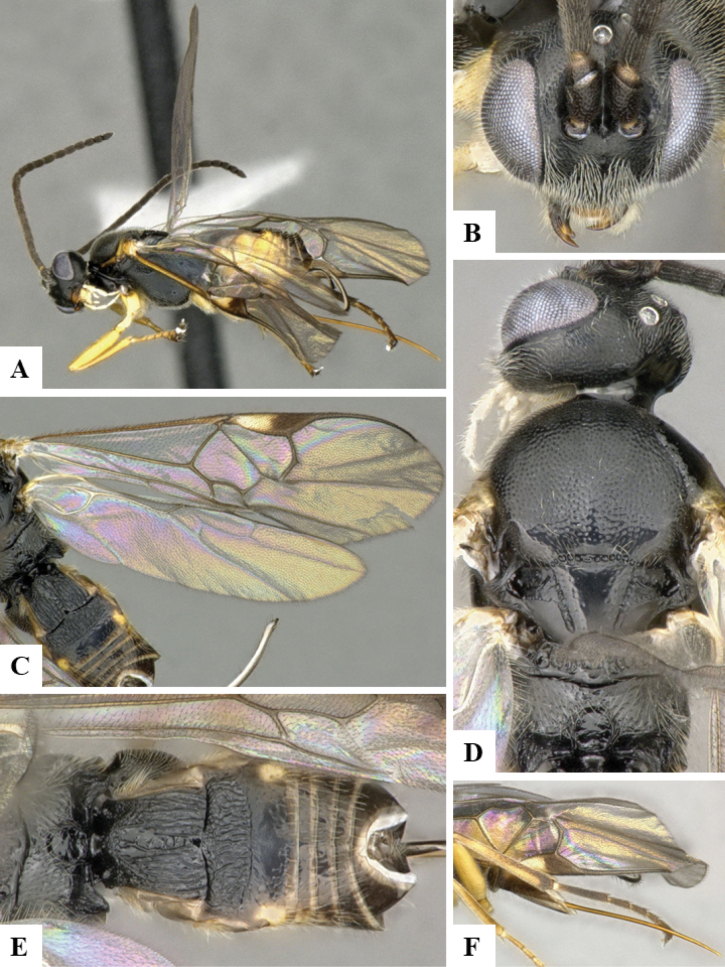
*Dolichogenidearichardashleyi* (Fernandez-Triana) holotype female DHJPAR0031507 **A** habitus, lateral **B** head, frontal **C** wings **D** mesosoma, dorsal **E** metasoma, dorsal **F** ovipositor, lateral.

#### ﻿ Dolichogenidea ritaashleyae

Taxon classificationAnimaliaHymenopteraBraconidae

(Fernandez-Triana, 2016)

BB16DB19-443F-542D-9229-120603A5A5D1

Fig. 116A–F

##### Notes.

Full details for this species in Fernandez-Triana et al. (2019). See also the key and Table 1 above.

**Figure 116.**
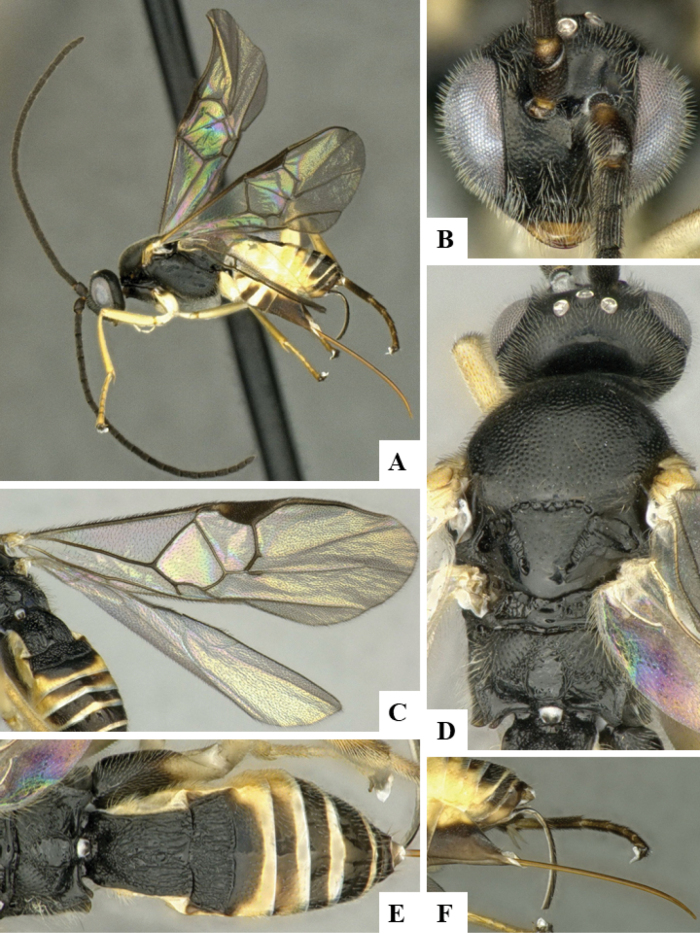
*Dolichogenidearitaashleyae* (Fernandez-Triana) holotype female DHJPAR0031500 **A** habitus, lateral **B** head, frontal **C** wings **D** mesosoma, dorsal **E** metasoma, dorsal **F** ovipositor, lateral.

#### ﻿ Dolichogenidea robertofernandezi

Taxon classificationAnimaliaHymenopteraBraconidae

Fernandez-Triana & Boudreault sp. nov.

7DA8C0ED-EA97-5DE2-8F8D-72D43CA89FDE

https://zoobank.org/B5B4A688-22AA-4DCC-967C-80F6BF697668

Fig. 117A–F

##### Type material.

***Holotype*.** Costa Rica • Female, CNC; Guanacaste, Area de Conservación Guanacaste, Sector Santa Rosa, Bosque San Emilio; 10.84389, -85.61384; 300 m; 1.v.2007; D. H. Janzen & W. Hallwachs leg.; Malaise trap; Voucher code: DHJPAR0013663. ***Paratypes*.** Costa Rica • 5 Females, CNC; DHJPAR0031824, DHJPAR0031724, DHJPAR0031743, DHJPAR0031707, DHJPAR0031703.

**Figure 117.**
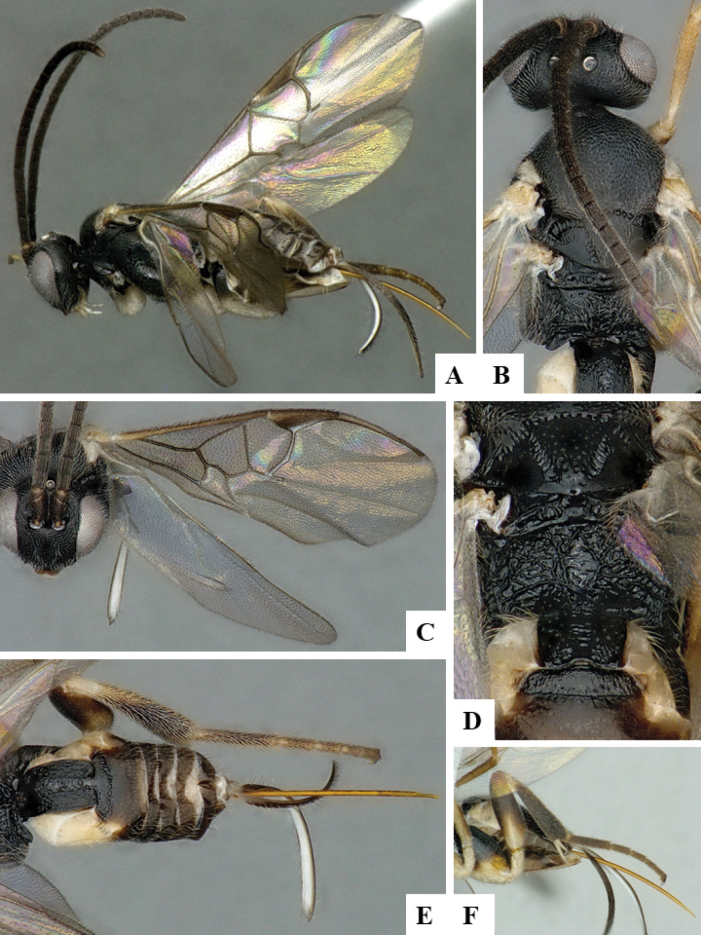
*Dolichogenidearobertofernandezi* Fernandez-Triana & Boudreault holotype female DHJPAR0013663 **A** habitus, lateral **B** mesosoma, dorsal **C** wings **D** propodeum & T1–T3, dorsal **E** metasoma, dorsal **F** metasoma, lateral.

##### Diagnostic description.

T1 mostly parallel-sided, its length medially ~ 2.5× its width at posterior margin; T2 transverse, its length medially 4.0–5.0× its width at posterior margin; T1 mostly sculptured on posterior 0.5; T2 mostly sculptured, central smooth area very small; tegula white-yellow, clearly paler in color than yellow humeral complex; pro- and mesocoxae yellow-white or yellow; metacoxa mostly yellow with dark brown spot on anterior 0.3; most of metafemur and metatibia dark brown to black; body length: 2.50–2.85 mm; fore wing length: 2.48–2.75 mm. Among all species with T2 at least partially smooth and pro- and mesocoxae pale, *D.robertofernandezi* can be distinguished by T1 shape, T2 shape and sculpture, and color of tegula, humeral complex, coxae, mesofemur, and metatibia. Two species (*D.isidrochaconi* and *D.jennyphillipsae*) are very similar morphologically to *D.robertofernandezi* and can only be reliably separated by DNA barcodes (see comments and details under the diagnostic description of *D.jennyphillipsae*).

##### Distribution.

Costa Rica.

##### Biology.

No host data available.

##### DNA barcoding data.

BINBOLD: BOLD:AAC8392 (33 sequences, 19 barcode compliant).

##### Etymology.

Named in honor of Mr Roberto Fernandez of Costa Rica’s Cartago and ACG in his old role as environmental advisor for Costa Rica’s National Electric Agency (ICE) and new role as a member of the directorate of BioAlfa, the ACG/GDFCF project to facilitate bioliteracy for the non-damaging conservation of wild tropical biodiversity.

#### ﻿ Dolichogenidea robinsherwoodae

Taxon classificationAnimaliaHymenopteraBraconidae

Fernandez-Triana & Boudreault sp. nov.

0C838401-F239-566E-AB96-EEF18B886731

https://zoobank.org/04F75793-464F-4014-AEAC-14396A1377C9

Figs 118A–E , 160B

##### Type material.

***Holotype*.** Costa Rica • Female, CNC; Guanacaste, Area de Conservación Guanacaste, Sector El Hacha, Sendero Bejuquilla; 11.03004, -85.52699; 280 m; 26.vii.1999; D. H. Janzen & W. Hallwachs leg.; Malaise trap; Voucher code: DHJPAR0012547. ***Paratypes*.** Costa Rica • 8 Females, 1 Male, CNC; DHJPAR0012544, DHJPAR0012738, DHJPAR0024731, DHJPAR0024734, DHJPAR0024739, DHJPAR0031848, DHJPAR0031850, DHJPAR0031853, DHJPAR0031854.

**Figure 118.**
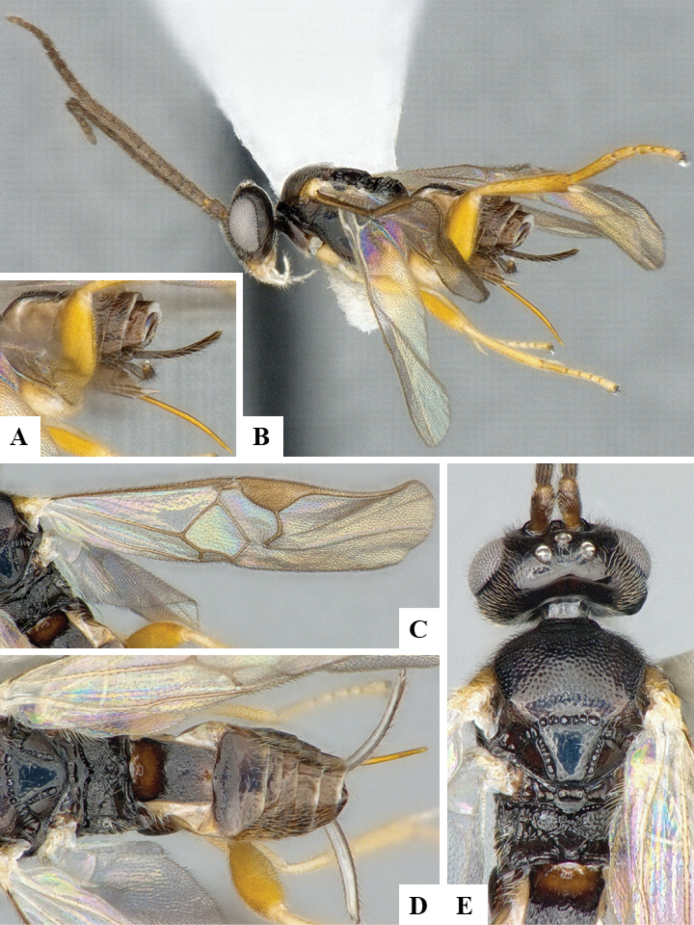
*Dolichogenidearobinsherwoodae* Fernandez-Triana & Boudreault holotype female DHJPAR0012547 **A** metasoma, lateral **B** habitus, lateral **C** wings **D** metasoma, dorsal **E** mesosoma, dorsal.

##### Diagnostic description.

Anteromesoscutum mostly smooth or with relatively shallow punctures; scutellar disc smooth and shiny, without punctures (at most with very shallow punctures along margins); mesopleuron and metapleuron entirely to almost entirely smooth or with few, shallow punctures; T1 parallel-sided, its length medially ≤ 2.5× its width at posterior margin; T2 mostly smooth; T2 comparatively more transverse, its length medially ~ 5.0× its width at posterior margin; ovipositor sheath 0.9–1.0× as long as metatibia; scape brown, same color than flagellomeres; tegula pale brown; humeral complex yellow; legs mostly pale (yellow), except for metacoxa with anterior 0.3–0.4 brown; body length: 1.84–2.40 mm; fore wing length: 2.24–2.63 mm. Among all species with smooth T2 and pale pro- and mesocoxae, *D.robinsherwoodae* can be distinguished by its mostly smooth mesosoma, T1 and T2 shape, ovipositor sheath length, and color of scape, tegula, humeral complex and legs.

##### Distribution.

Costa Rica.

##### Biology.

Solitary. Crambidae: *Antaeotricha* Janzen221.

##### DNA barcoding data.

BINBOLD:ABX5195 (27 sequences, 20 barcode compliant).

##### Etymology.

Named after Dr. Robin Sherwood in recognition of her caring for the Department of Biology that houses DHJ an WH at the University of Pennsylvania.

#### ﻿ Dolichogenidea robmacewani

Taxon classificationAnimaliaHymenopteraBraconidae

Fernandez-Triana & Boudreault sp. nov.

9B337FCE-CE1A-5B58-87CD-3C8D95843C4D

https://zoobank.org/21309F22-0123-41F4-89BC-500891118915

Fig. 119A–F

##### Type material.

***Holotype*.** Brazil • Female, CNC; Bahia, Encruzilhada; 880 m; xi.1974; M. Alvarenga leg.; Voucher code: CNC1196549. ***Paratypes*.** Brazil • 5 Females, CNC; CNC5342690, CNC5342691, CNC5342692, CNC5342693, CNC5342694.

**Figure 119.**
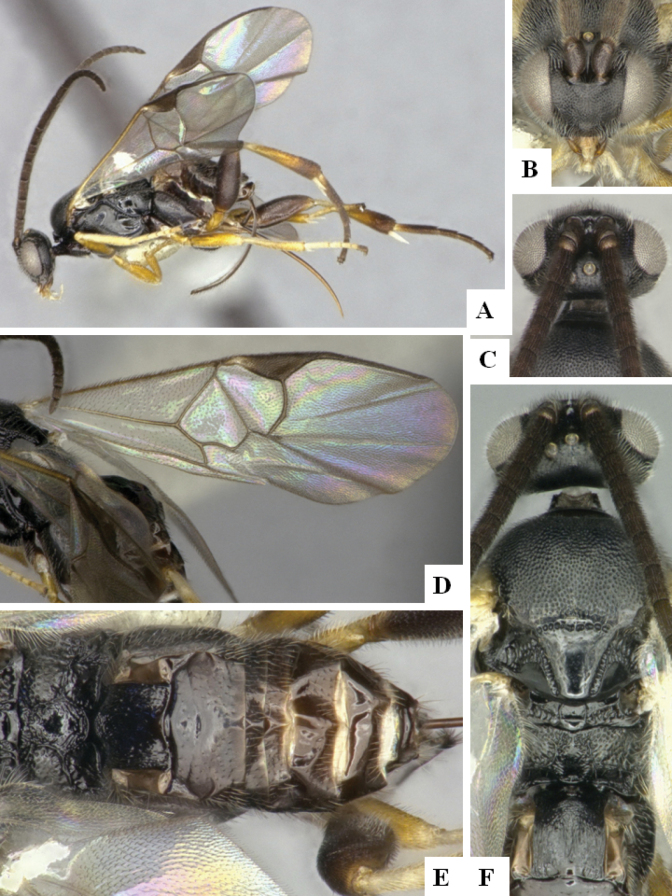
*Dolichogenidearobmacewani* Fernandez-Triana & Boudreault holotype female CNC1196549 **A** habitus, lateral **B** head, frontal **C** head, dorsal **D** wings **E** metasoma, dorsal **F** mesosoma, dorsal.

##### Diagnostic description.

T1 more or less parallel-sided and mostly sculptured with longitudinal striae on posterior 0.5–0.6 (but sometimes with central smooth area near posterior margin); T2 more or less transverse and entirely smooth; ovipositor sheath clearly longer than metatibia length (≥ 1.25×, usually more); labial palpi, tegula and humeral complex yellow; first two pairs of legs entirely yellow (except for coxae brown); third pair of legs mostly brown to dark brown (except for yellow trochanter and trochantellus, anterior 0.7 of metatibia yellow and metatibial spurs yellow-white); body length: 2.44–3.06 mm; fore wing length: 2.84–3.38 mm. Among species with sculptured T1 but smooth and transverse T2, and all coxae dark colored, *D.robmacewani* can be distinguished by its mostly yellow legs (except third pair), yellow tegula, humeral complex and palpi, and relatively long ovipositor sheaths.

##### Distribution.

Brazil (BA, MG).

##### Biology.

No host data available.

##### DNA barcoding data.

No data.

##### Etymology.

The second author dedicates this species in honor of her friend Rob MacEwan. Rob’s knowledge of nature and kindness are both extraordinary! His friendship is greatly appreciated.

##### Notes.

Among the paratypes there is some variation on sculpture of T1 and color of metafemur and metatibia, but they are all kept as one species until more material becomes available for study.

#### ﻿ Dolichogenidea robpringlei

Taxon classificationAnimaliaHymenopteraBraconidae

Fernandez-Triana & Boudreault sp. nov.

F3F4AEB0-8810-5BFE-8134-6CCEE5F51C46

https://zoobank.org/88576D3D-EB6F-4031-8FFA-A97A6870938B

Figs 120A–E , 161A

##### Type material.

***Holotype*.** Costa Rica • Female, CNC; Guanacaste, Area de Conservación Guanacaste, Sector Pitilla, Sendero Cuestona; 10.99455, -85.41461; 640 m; 24.v.2012; Petrona Rios leg.; Host: *Collinsaferreiceps*DHJ01; Voucher code: DHJPAR0049779; Host voucher code: 12-SRNP-30918.

**Figure 120.**
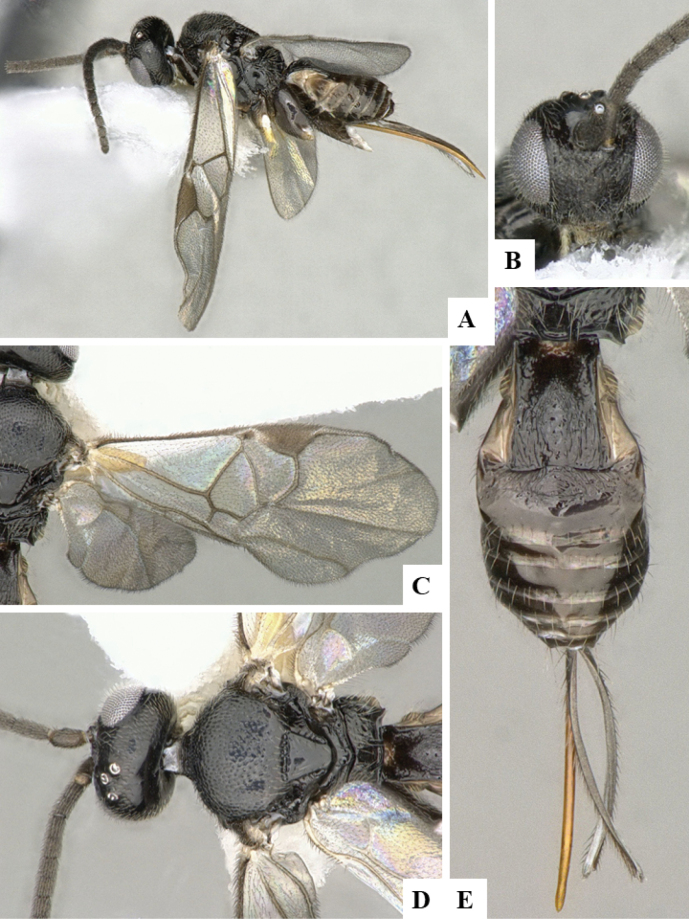
*Dolichogenidearobpringlei* Fernandez-Triana & Boudreault holotype female DHJPAR0049779 **A** habitus, lateral **B** head, frontal **C** wings **D** mesosoma, dorsal **E** metasoma, dorsal.

##### Diagnostic description.

F15 cubic, its length 1.1× its width; sculpture on anteromesoscutum and propodeum shallow; T1 mostly parallel-sided but slightly broadening near posterior margin; T1 with strong, longitudinal striae on posterior 0.5; T2 very transverse, with anterior and posterior margins strongly sinuate; T2 mostly sculptured, but with smooth areas along margins; tegula and humeral complex brown; longitudinal strip on metasternum yellow-white; all coxae brown; pro- and mesofemora entirely yellow; body length: 2.02 mm; fore wing length: 2.20 mm. Among species with mostly sculptured T2, *D.robpringlei* can be distinguished by its T1 and T2 shape and sculpture, anteromesoscutum and propodeum sculpture, legs color and overall body appearance less shiny and less smooth than closest (i.e., similar morphologically) species; F15 length is the main diagnostic character to separate it from *D.scottmilleri*.

##### Distribution.

Costa Rica.

##### Biology.

Gregarious. Thyrididae, *Collinsaferreiceps*.

##### DNA barcoding data.

No data.

##### Etymology.

Named in honor of Dr. Rob Pringle of Princeton University, New Jersey, USA, for his three decades of steady and enthusiastic interest in, and support of, all the GDFCF and ACG activities as a member of the Board of Directors for the Guanacaste Dry Forest Conservation Fund in its integration with Area de Conservación Guanacaste, Costa Rica.

#### ﻿ Dolichogenidea rociocordobae

Taxon classificationAnimaliaHymenopteraBraconidae

Fernandez-Triana & Boudreault sp. nov.

B2C88DBF-6151-5E92-8FC1-54060F0C34D8

https://zoobank.org/095501A2-B96B-4BEF-B981-41A5CA774776

Figs 121A–F , 161B

##### Type material.

***Holotype*.** Costa Rica • Female, CNC; Alajuela, Area de Conservación Guanacaste, Sector San Cristobal, Sendero Huerta; 10.9305, -85.3723; 527 m; 14.xii.2012; Elda Araya leg.; Host: *Dichomeris* Janzen703; Voucher code: DHJPAR0051131; Host voucher code: 12-SRNP-5560. ***Paratypes*.** Costa Rica • 1 Female, 1 Male, CNC; DHJPAR0051072, DHJPAR0051084.

**Figure 121.**
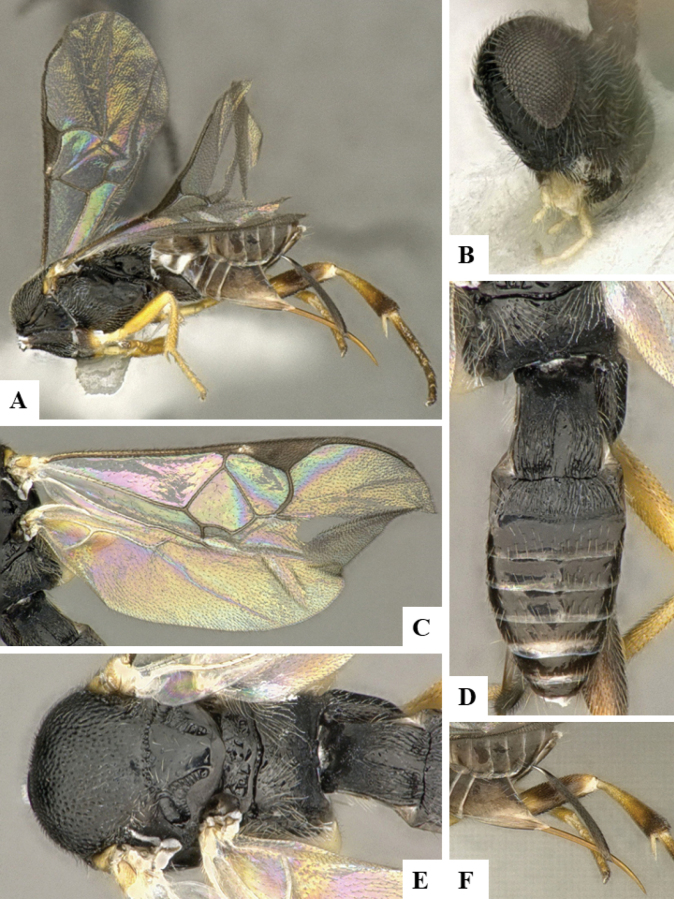
*Dolichogenidearociocordodae* Fernandez-Triana & Boudreault holotype female DHJPAR0051131 **A** habitus, lateral **B** head, fronto-lateral **C** wings **D** metasoma, dorsal **E** mesosoma, dorsal **F** ovipositor, lateral.

##### Diagnostic description.

T1 and T2 heavily sculptured with strong longitudinal striae; T1 length < 1.5× T1 width at posterior margin; T2 rectangular, covering most surface of tergum; ovipositor sheath almost as long as metatibia length (0.95×); F15 1.2× as long as wide; metafemur mostly brown; metatibia dark brown to black on posterior 0.5; body length: 2.50 mm; fore wing length: 2.60 mm. Among all species with heavily sculptured T1 and T2, T1 comparatively broad and T2 rectangular, *D.rociocordobae* can be distinguished by the color of metafemur and metatibia, and length of F15. The only species closely similar morphologically is *D.frankjoycei* which has slightly shorter ovipositor sheath and slightly longer F15, as well as different hosts and DNA barcode.

##### Distribution.

Costa Rica.

##### Biology.

Solitary. Gelechiidae, *Dichomeris* Janzen273, *Dichomeris* Janzen703.

##### DNA barcoding data.

BINBOLD:ACJ2777 (7 sequences, 7 barcode compliant).

##### Etymology.

Named in honor of Sra. Rocio Cordoba (MS) of San Jose, Costa Rica, in recognition of her own decades of efforts in the World Bank on behalf of Costa Rica’s environment as well those of her husband Dr. Jorge Cortes for the same cause.

#### ﻿ Dolichogenidea rodrigogamezi

Taxon classificationAnimaliaHymenopteraBraconidae

Fernandez-Triana & Boudreault sp. nov.

D01F1ED6-BECD-58B3-ABB8-50DD6D70E8B7

https://zoobank.org/2B6FDB76-E2ED-4C21-84E3-6C8EC773E9B5

Fig. 122A–F

##### Type material.

***Holotype*.** Costa Rica • Female, CNC; Guanacaste, Area de Conservación Guanacaste, Sector Cacao, Cerro Pedregal; 10.92767, -85.47449; 1,080 m; 22.xi.2008; D.H. Janzen & W.Hallwachs leg.; Voucher code: DHJPAR0033753.

**Figure 122.**
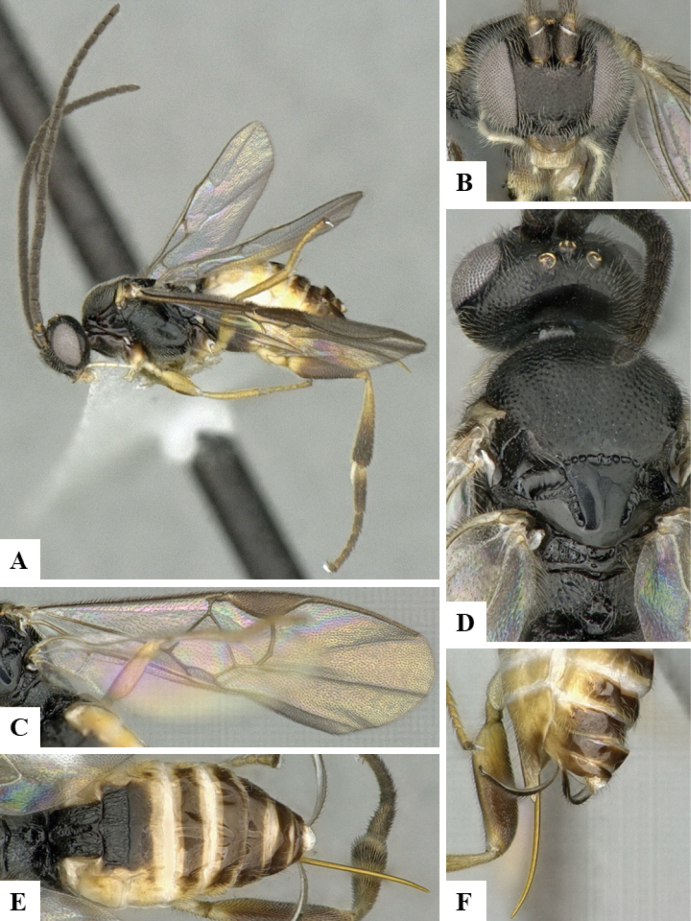
*Dolichogenidearodrigogamezi* Fernandez-Triana & Boudreault holotype female DHJPAR0033753 **A** habitus, lateral **B** head, frontal **C** wings **D** mesosoma, dorsal **E** metasoma, dorsal **F** ovipositor, lateral.

##### Diagnostic description.

F15 2.0× as long as wide; T1 and T2 heavily sculptured with strong longitudinal striae; T1 length < 1.5× T1 width at posterior margin; T2 broadly rectangular and large, covering most surface of tergum; metafemur mostly brown; metatibia dark brown to black on posterior 0.5–0.7; body length: 2.83 mm; fore wing length: 3.00 mm; BINBOLD:AAM5843, which is 5.93% different from the nearest BIN in BOLD as of March 2022. Among all species with heavily sculptured T1 and T2, T1 comparatively broad and T2 rectangular, *D.rodrigogamezi* can be distinguished by the color of metafemur and metatibia, and length of F15.

##### Distribution.

Costa Rica.

##### Biology.

No host data available.

##### DNA barcoding data.

BINBOLD:AAM5843 (one sequence, barcode compliant).

##### Etymology.

Named in honor of Dr. Rodrigo Gámez of San Jose, Costa Rica and of the Universidad de Costa Rica in honor of his decades of support for Area de Conservación Guanacaste in northwestern Costa Rica and for non-damaging biodevelopment of wild Costa Rican biodiversity.

#### ﻿ Dolichogenidea rogerblancoi

Taxon classificationAnimaliaHymenopteraBraconidae

Fernandez-Triana & Boudreault, 2019

633A8B18-DC16-5831-806A-E7E5EE596B9E

Figs 123A–E , 162A

##### Notes.

Full details for this species in Fernandez-Triana et al. (2019). See also the key and Table 1 above. The new country record from Ecuador is based on a perfect match of sequence CNCHYM 00124 (CNC specimen). The host recorded in BOLD for this wasp species (*Antaeotricha* Janzen107) is different from the host reported in the original description (*Antaeotricha* radicalisDHJ01) although it remains in the same genus, a result of an improved identification of the host.

**Figure 123.**
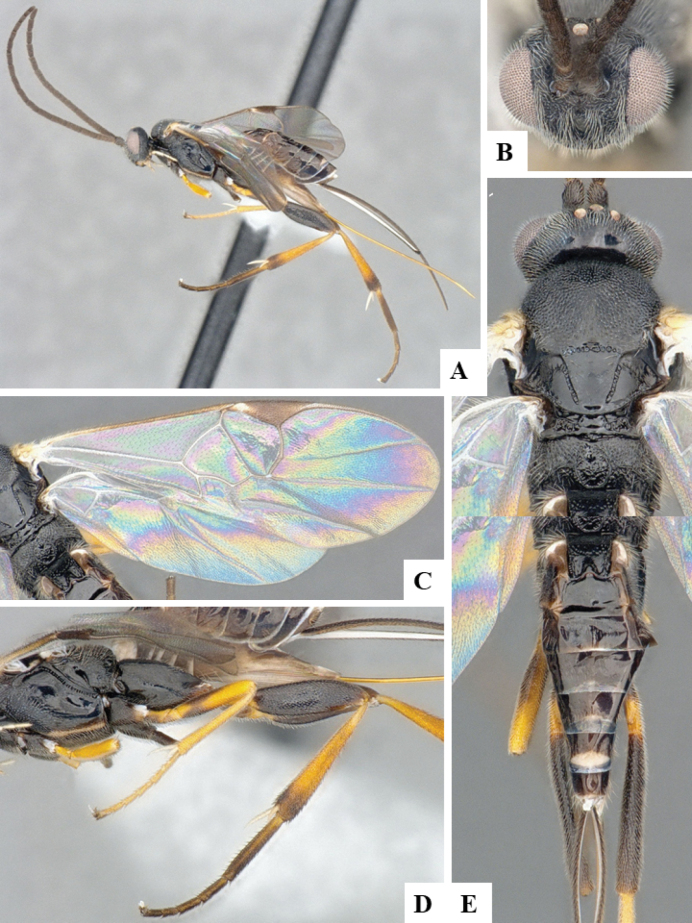
*Dolichogenidearogerblancoi* Fernandez-Triana & Boudreault holotype female DHJPAR0049840 **A** habitus, lateral **B** head, frontal **C** wings **D** hind leg, lateral **E** habitus, dorsal.

#### ﻿ Dolichogenidea ronaldzunigai

Taxon classificationAnimaliaHymenopteraBraconidae

Fernandez-Triana & Boudreault sp. nov.

D5868D20-6DE5-50EB-8A67-E46D46590018

https://zoobank.org/E7A954F4-7786-464E-8DC7-040354504313

Figs 124A–F , 162B

##### Type material.

***Holotype*.** Costa Rica • Female, CNC; Alajuela, Area de Conservación Guanacaste, Sector San Cristobal, Cementerio Viejo; 10.88111, -85.38889; 570 m; 23.vi.2013; Gloria Sihezar leg.; Host: *Chlamydastismontywoodi*; Voucher code: DHJPAR0052991; Host voucher code: 13-SRNP-3309. ***Paratypes*.** Costa Rica • 2 Females, 2 Males, CNC; DHJPAR0041632, DHJPAR0041637, DHJPAR0053800, DHJPAR0053861.

**Figure 124.**
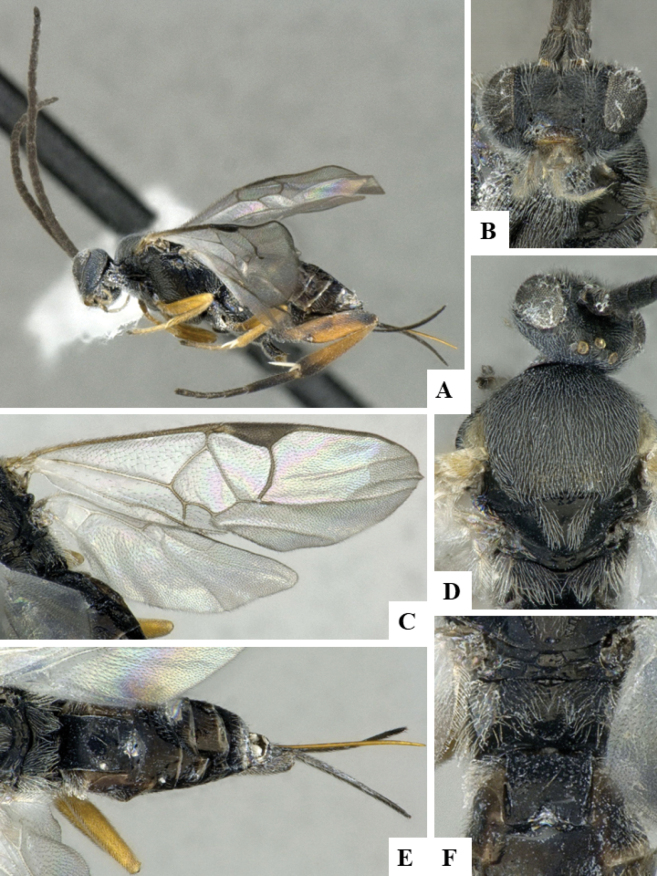
*Dolichogenidearonaldzunigai* Fernandez-Triana & Boudreault holotype female DHJPAR0052991 **A** habitus, lateral **B** head, frontal **C** wings **D** mesosoma, dorsal **E** metasoma, dorsal **F** propodeum & T1–T2, dorsal.

##### Diagnostic description.

T1 parallel-sided but broad, its length medially 2.0× its width at posterior margin; T1 shiny and almost entirely smooth (at most with few shallow punctures along lateral margins on posterior 0.3); T2 transverse and comparatively narrow, its width at posterior margin 2.6–3.0× its length medially; T2 smooth; ovipositor sheaths 1.1–1.2× metatibia length; all coxae black; metafemur mostly yellow-brown with brown spot on apical 0.2 dorsally; metatibia mostly yellow-brown with posterior 0.2–0.3 brown; body length: 2.84–3.31 mm; fore wing length: 3.13–3.41 mm. Among all species with T2 smooth and dark coxae *D.ronaldzunigai* can be distinguished by its entirely smooth, parallel-sided T1 and overall polished aspect of the species, as well as legs color. The species could also be confused with the *carlosmanuelrodriguezi* group based on its overall sculptured and shape of T1 and T2; however, its ovipositor sheaths are much shorter than in that species group (1.1–1.2× versus 1.8–2.0×) and T2 is much less quadrate.

##### Distribution.

Costa Rica.

##### Biology.

Solitary. Depressariidae: *Chlamydastismontywoodi*, *Chlamydastistryphon*, *Chlamydastisvividella*, *Stenoma* Janzen199, elachJanzen01 Janzen693.

##### DNA barcoding data.

BINBOLD:AAT8860 (6 sequences, 6 barcode compliant).

##### Etymology.

Named in honor of Sr. Ronald Zuñiga of Costa Rica, and the Costa Rican National Museum, BioAlfa and the former INBio (Instituto Nacional de Biodiversity) in recognition of his three+ decades dedicated to the biodiversity understanding of the Hymenoptera of Costa Rica.

#### ﻿ Dolichogenidea rosamatarritae

Taxon classificationAnimaliaHymenopteraBraconidae

(Fernandez-Triana, 2016)

2FC8FCE9-386B-5D9A-9A25-A134A535B6CC

Fig. 125A–F

##### Notes.

This species was discussed by Fernandez-Triana et al. (2016); however, no molecular data had been reported before, therefore it is done here. See also the key and Table 1 above for more details.

**Figure 125.**
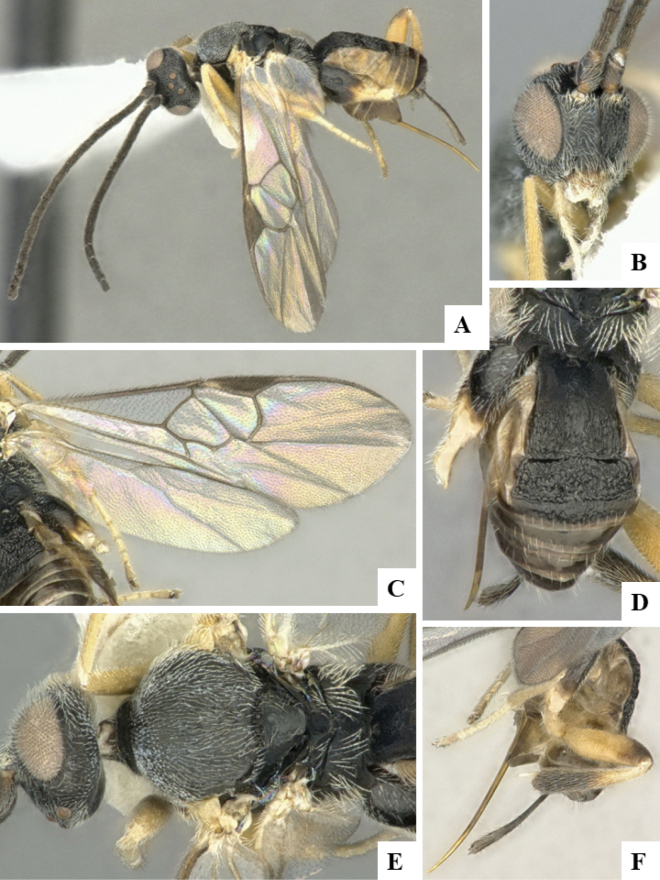
*Dolichogenidearosamatarritae* (Fernandez-Triana) holotype female DHJPAR0053053 **A** habitus, lateral **B** head, frontal **C** wings **D** metasoma, dorsal **E** mesosoma, dorsal **F** metasoma, lateral.

#### ﻿ Dolichogenidea rubymacpearsae

Taxon classificationAnimaliaHymenopteraBraconidae

Fernandez-Triana & Boudreault sp. nov.

8B3A4144-FF7F-5DE7-83E9-DD5AC0558DE8

https://zoobank.org/C9F2BF6E-3EB4-477D-AEB3-A9BE70E28A8A

Fig. 126A–H

##### Type material.

***Holotype*.** Ecuador • Female, CNC; Napo, Quito-Baeza road; 4,100 m; 10.ii.1983; L. Masner leg.; Voucher code: CNC1196551. ***Paratypes*.** Peru • 8 Males, CNC; CNC5342682, CNC5342683, CNC5342684, CNC5342685, CNC5342686, CNC5342687, CNC5342688, CNC5342689.

**Figure 126.**
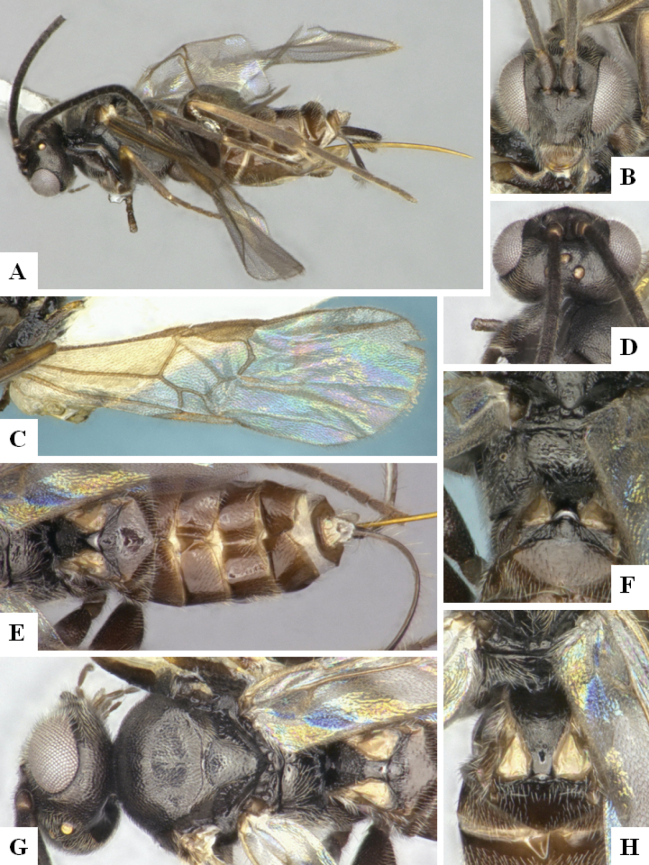
*Dolichogenidearubymacpearsae* Fernandez-Triana & Boudreault holotype female CNC1196551 **A** habitus, lateral **B** head, frontal **C** fore Wing **D** head, dorsal **E** metasoma, dorsal **F** propodeum & T1–T3, dorsal **G** mesosoma, dorsal **H** T1, dorsal.

##### Diagnostic description.

Vein R1 shorter than pterostigma length and approx. as long as distance between its end and end of vein 3RSb; pterostigma comparatively narrow (3.0× as long as wide) and often with lower anterior margin angulated so that it looks as having four sides; vein r arising from apical 0.7 of pterostigma; propodeum with almost no traces of areola, with only some small, poorly defined carinae from nucha; T1 very strongly narrowing near posterior margin, its length 4.5× its width at posterior margin, and width at anterior margin 3.0× width at posterior margin; comparatively dark colored species, with all legs entirely dark brown to black (except for yellow-brown on posterior 0.1 of pro- and mesofemora and anterior 0.1–0.2 of tibiae); palpi, tegula and humeral complex dark brown; body length: 2.40 mm; fore wing length: 2.26 mm. Male specimens show some variation in venation, shape of T1 and shape and sculpture of T2. This is a very distinctive species based on rather unique T1 shape (strongly narrowing), shape and length of pterostigma and overall dark coloration.

##### Distribution.

Ecuador, Peru.

##### Biology.

No host data available, but the fact that eight male specimens were collected the same day in the same locality suggest this species is gregarious.

##### DNA barcoding data.

No data.

##### Etymology.

The second author dedicates this species to Ruby MacEwan, daughter of Rob MacEwan. Rob wanted so much to have a species named after his daughter and I wanted to name one after him, so they are both honored in this paper. The first four letter of the species are her first name “Ruby”, “mac” is part of the last name of Rob and “pears” is Ruby’s mother last name.

##### Notes.

The male specimens are from a locality in Peru, which is > 1,000 km apart from the locality of the holotype, in Ecuador. However, they are considered the same species because of similar morphology and the fact that both localities are more than 4,000 m elevation in the Andes.

#### ﻿ Dolichogenidea rudyamadori

Taxon classificationAnimaliaHymenopteraBraconidae

Fernandez-Triana & Boudreault sp. nov.

3A98948F-C46F-5BC5-A559-0F8D48208876

https://zoobank.org/CD4D86DC-0035-4B01-A7C4-DD1D44634917

Fig. 127A–E

##### Type material.

***Holotype*.** Costa Rica • Female, CNC; Guanacaste, Area de Conservación Guanacaste, Sector Cacao, Sendero Cima; 10.93328, -85.45729; 1,460 m; 05.x.1998; D. H. Janzen & W. Hallwachs leg.; Malaise trap; Voucher code: DHJPAR0012545.

**Figure 127.**
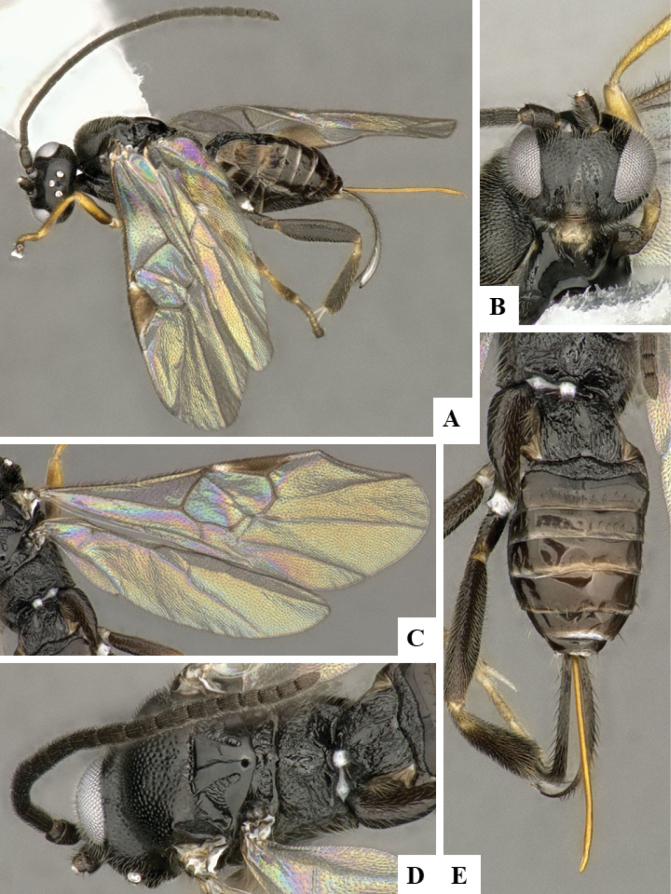
*Dolichogenidearudyamadori* Fernandez-Triana & Boudreault holotype female DHJPAR0012545 **A** habitus, lateral **B** head, frontal **C** wings **D** mesosoma, dorsal **E** metasoma, dorsal.

##### Diagnostic description.

T1 strongly sculptured on posterior 0.5; T2 almost entirely sculptured; T2 very transverse, its width at posterior margin 4.5× its central length; ovipositor tip weakly sinuate; pterostigma with pale spot very small, 0.1 pterostigma length; all coxae, metafemora, and most of metatibia (except for anterior 0.2 which is yellow) dark brown; body length: 2.20 mm; fore wing length: 2.53 mm. This species could be difficult to key out, especially on couplet 10 where the interpretation of T2 sculpture could lead to different alternatives. While *D.rudyamadori* has T2 almost entirely sculptured (in that sense it would appear to run through the first half of couplet 10), its shape is nevertheless very different from all other species with entirely and strongly sculptured T2, as *D.rudyamadori* has T2 very transverse (width at posterior margin 4.5× its central length). In addition to that, the weakly sinuate tip of ovipositor is also very distinctive (and never present in any other species with strongly sculptured T2); thus, these two characters clearly separate the species.

##### Distribution.

Costa Rica.

##### Biology.

No host data available.

##### DNA barcoding data.

BINBOLD:AAX8664 (1 sequence, barcode compliant).

##### Etymology.

Named in honor of Sr. Rudy Amador in recognition of his enthusiastic facilitation of the Dole Pineapple Company plantation, in the central northern lowlands of what used to be Costa Rican Caribbean coastal rain forest, for being willing to support Malaise trapping for all insects for the Costa Rican BioAlfa DNA barcode library that live in the plantation and adjacent secondary successional rain forest in 2022, and to understand the biodiversity dynamics of the crop itself.

#### ﻿ Dolichogenidea sallydaleyae

Taxon classificationAnimaliaHymenopteraBraconidae

Fernandez-Triana & Boudreault sp. nov.

5E936E13-BF3D-5482-A1E3-E7D964CC8C35

https://zoobank.org/7EAE6D7C-E9B0-4861-AA1A-3B62E0CDC4BC

Fig. 128A–F

##### Type material.

***Holotype*.** Costa Rica • Female, CNC; Guanacaste, Area de Conservación Guanacaste, Guanacaste, Sector Pitilla, Sendero Laguna; 10.9888, -85.42336; 680 m; 19.ix.2013; Freddy Quesada leg.; Host: elachBioLep01 BioLep286; Voucher code: DHJPAR0054803; Host voucher code: 13-SRNP-31297.

**Figure 128.**
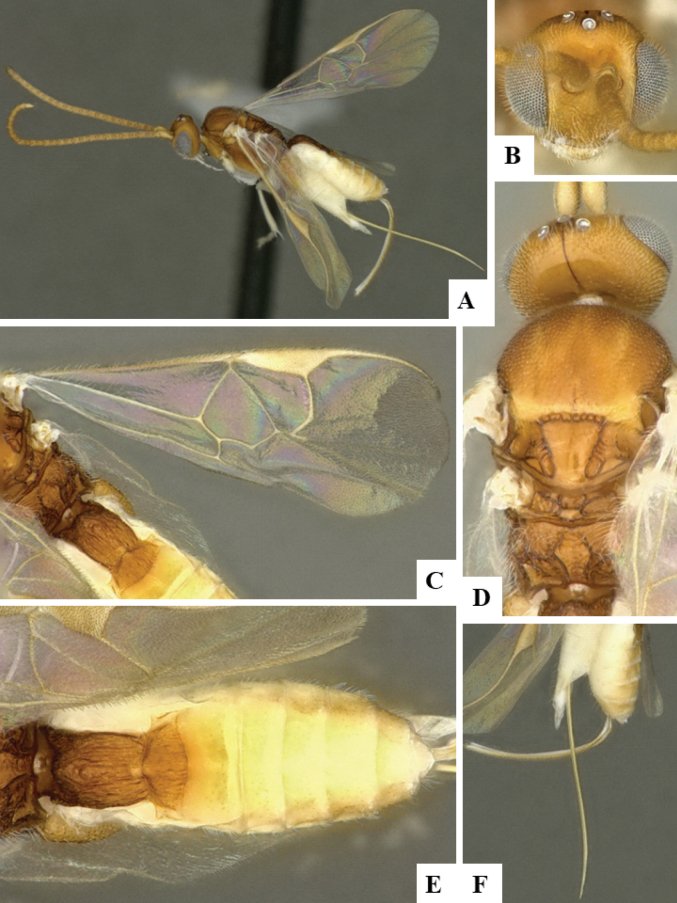
*Dolichogenideasallydaleyae* Fernandez-Triana & Boudreault holotype female DHJPAR0054803 **A** habitus, lateral **B** head, frontal **C** wings **D** mesosoma, dorsal **E** metasoma, dorsal **F** ovipositor, lateral.

##### Diagnostic description.

T1 and T2 heavily sculptured with strong longitudinal striae; T1 length 2.0× its width at posterior margin; T2 broadly rectangular; ovipositor sheath ~ 2.0× metatibia length; metacoxa entirely brown; hypopygium and all sternites yellow; body length: 3.15 mm; fore wing length: 3.06 mm; ovipositor sheath length: 1.50 mm; BINBOLD:ACM2280, which is 2.33% different from the nearest BIN in BOLD as of March 2022.. It can be distinguished among all species with T1 and T2 heavily sculptured with strong longitudinal striae by its comparatively long ovipositor sheath and body size; the morphological similar *D.tomdaleyi* has comparatively longer ovipositor sheath, darker sternites and hypopygium and paler colored metacoxa, as well as slightly longer body size.

##### Distribution.

Costa Rica.

##### Biology.

Solitary. Reared from a single species of Depressariidae with interim name elachBioLep01 BioLep286.

##### DNA barcoding data.

BINBOLD:ACM2280 (1 sequence, barcode compliant).

##### Etymology.

Named in honor of Mrs. Sally Daley of Moraga, California, USA in recognition of her years of administration of financial and tax affairs for the Guanacaste Dry Forest Conservation Fund and its conservation-based integration with Area de Conservación Guanacaste in northwestern Costa Rica.

##### Notes.

The only specimen available for study was a teneral female, its coloration probably being paler than what the species would actually look.

#### ﻿ Dolichogenidea sarahoconnorae

Taxon classificationAnimaliaHymenopteraBraconidae

Fernandez-Triana & Boudreault sp. nov.

4416BFF9-FC57-5520-86E9-DACBF0E044F9

https://zoobank.org/073A137D-5EC4-4187-B0FA-E1F0A051D7D8

**Figure 129.**
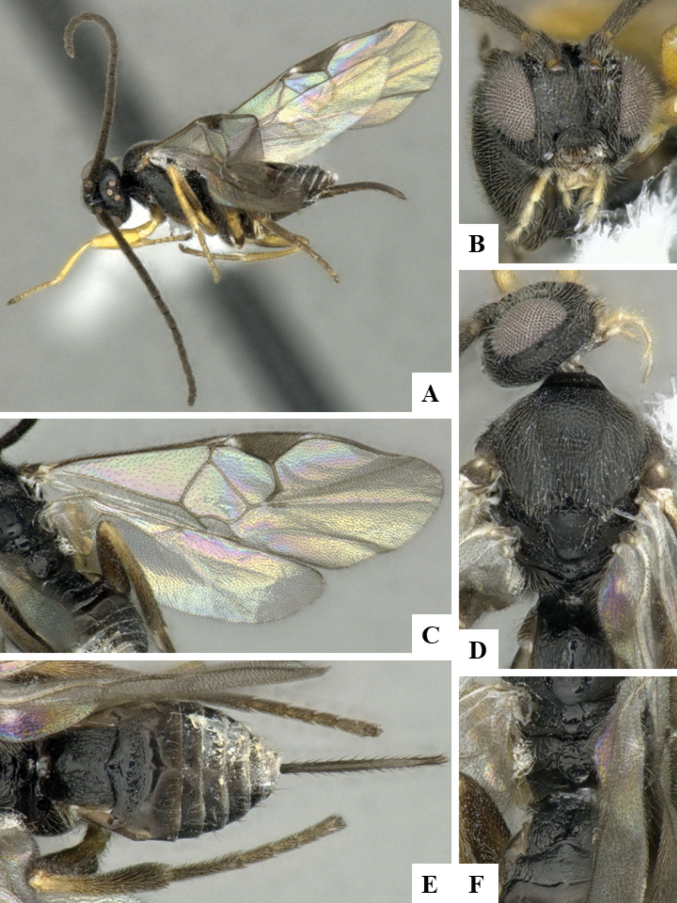
*Dolichogenideasarahoconnorae* Fernandez-Triana & Boudreault holotype female DHJPAR0049819 **A** habitus, lateral **B** head, frontal **C** wings **D** mesosoma, dorsal **E** metasoma, dorsal **F** propodeum & T1–T2, dorsal.

**Figure 130.**
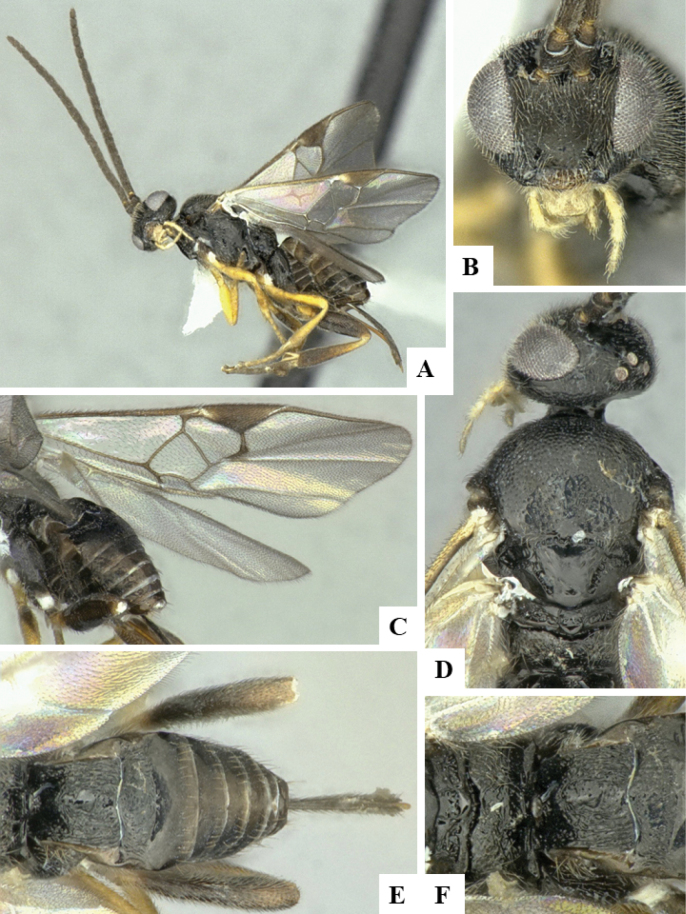
*Dolichogenideasarahoconnorae* Fernandez-Triana & Boudreault paratype female DHJPAR0054637 **A** habitus, lateral **B** head, frontal **C** wings **D** mesosoma, dorsal **E** metasoma, dorsal **F** propodeum & T1–T2, dorsal.

Figs 129A–F , 130A–F , 163A, B

##### Type material.

***Holotype*.** Costa Rica • Female, CNC; Alajuela, Area de Conservación Guanacaste, Sector Rincon Rain Forest, Camino Rio Francia; 10.90425, -85.28651; 410 m; 25.vi.2012; Jose Perez leg.; Host: siculoJanzen01 biolep03; Voucher code: DHJPAR0049819; Host voucher code: 12-SRNP-43137. ***Paratypes*.** Costa Rica • 17 Females, CNC; DHJPAR0054637, CNC5302051, CNC5302052 (There are more specimens in gel capsule attached to the pin), CNC5302053, CNC5302054, CNC5302055 (there are more specimens in gel capsule attached to pin), CNC5302056, CNC5302057, CNC5302058, CNC5302059, CNC5302060, CNC5302061, CNC5302062, CNC5302063, CNC5342655, CNC5342656 (there are more specimens in gel capsule attached to pin), DHJPAR0049123.

##### Diagnostic description.

Anteromesoscutum punctures less coarse on posterior 0.3–0.4; propodeum areola complete; propodeum mostly smooth, with few areas between carinae with some striation; T1 mostly parallel-sided but slightly broadening near posterior margin; T1 mostly sculptured on posterior 0.5; T2 smooth but with scattered punctures near margins, sometimes with stronger sculpture; tegula and humeral complex brown; pedicel, most of mesofemur and longitudinal band on mesosternum yellow to yellow-brown; pro- and mesocoxae yellow-brown to brown; metacoxa brown to dark brown; mesofemur and most of metatibia (except for anterior 0.3–0.4 yellow) brown to dark brown; body length and fore wing length: 2.40–2.60 mm. Among species with smooth T2, *D.sarahoconnorae* can be distinguished by T1 sculpture, propodeum sculpture and carination pattern, body size, and color pattern (especially pedicel, mesosternum, and legs).

##### Distribution.

Costa Rica.

##### Biology.

Gregarious. Thyrididae: *Microscahedialis*, *Microscapolychloralis*, siculoJanzen01 biolep03, siculoJanzen01 Janzen05.

##### DNA barcoding data.

BINBOLD:ABX6008 (67 sequences, 67 barcode compliant).

##### Etymology.

Named in honor of Mrs. Sarah O’Connor of Connecticut, USA, as a new and enthusiastic member of the Board of Directors for the Guanacaste Dry Forest Conservation Fund in its integration with the Area de Conservación Guanacaste.

#### ﻿ Dolichogenidea scottmilleri

Taxon classificationAnimaliaHymenopteraBraconidae

Fernandez-Triana & Boudreault sp. nov.

EB837CF5-36B7-59E8-8277-F1132D837C99

https://zoobank.org/A8276ABF-E512-4E4F-BCE6-AC909064F7E2

**Figure 131.**
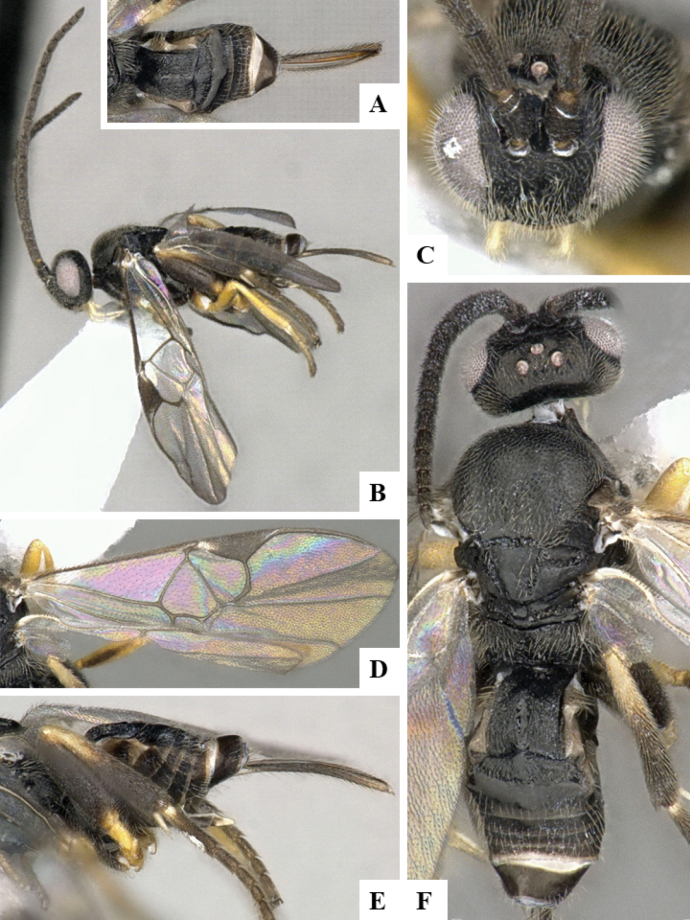
*Dolichogenideascottmilleri* Fernandez-Triana & Boudreault holotype female DHJPAR0054638 **A** metasoma, dorsal **B** habitus, lateral **C** head, frontal **D** wings **E** metasoma, lateral **F** habitus, dorsal.

**Figure 132.**
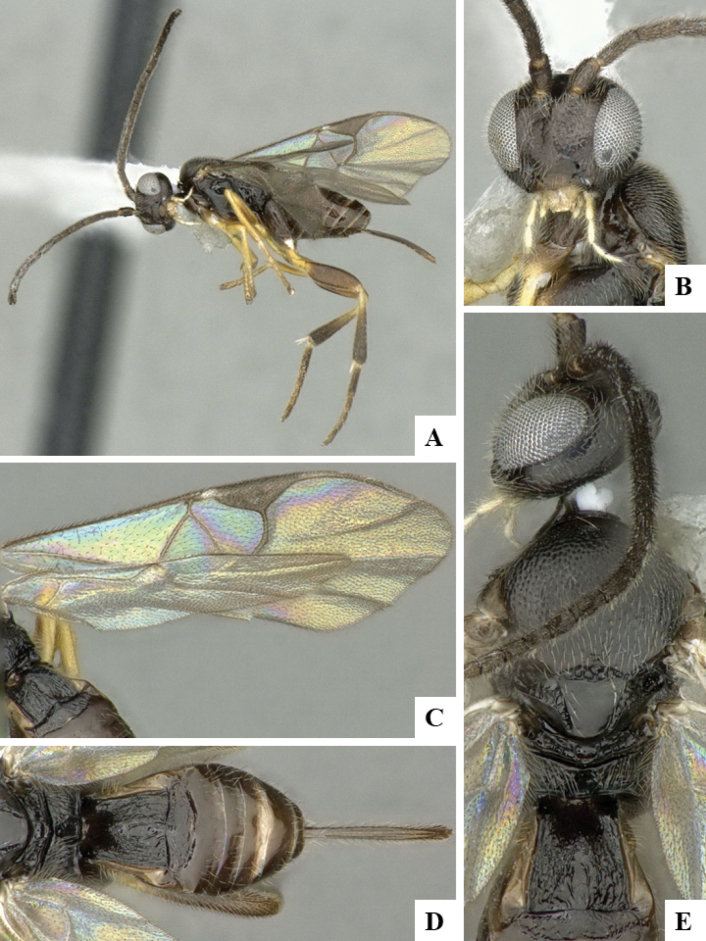
*Dolichogenideascottmilleri* Fernandez-Triana & Boudreault paratype female DHJPAR0049101 **A** habitus, lateral **B** head, fronto-lateral **C** wings **D** metasoma, dorsal **E** mesosoma, dorsal.

Figs 131A–F , 132A–E , 164A

##### Type material.

***Holotype*.** Costa Rica • Female, CNC; Alajuela, Area de Conservación Guanacaste, Sector San Cristobal, Sendero Perdido; 10.87940, -85.38607; 620 m; 09.x.2013; Gloria Sihezar leg.; Host: *Microscapaullula*; Voucher code: DHJPAR0054638; Host voucher code: 13-SRNP-5498. ***Paratypes*.** Costa Rica • 28 Females, 1 Male, CNC; DHJPAR0047270, CNC5342657 (there are more specimens in gel capsule attached to pin), CNC5342658, DHJPAR0053763, DHJPAR0054604, CNC5342659, CNC5342660, CNC5342661, DHJPAR0054586, DHJPAR0047262, CNC5342662 (there are more specimens in gel capsule attached to pin), CNC5342663, DHJPAR0049111, DHJPAR0051782, DHJPAR0049101, CNC5342664, CNC5342665, CNC5342666 (there are more specimens in gel capsule attached to pin), CNC5342667, CNC5342668, CNC5342669 (there are more specimens in gel capsule attached to pin), CNC5342670, CNC5342671, CNC5342672 (there are more specimens in gel capsule attached to pin), CNC5342673 (there are more specimens in gel capsule attached to pin), CNC5342674, CNC5342675 (there are more specimens in gel capsule attached to pin), CNC5342676, CNC5342677.

##### Diagnostic description.

F15 length > 1.5× its width; sculpture on anteromesoscutum and propodeum coarse and deeply indicated (rarely shallow); T1 mostly parallel-sided but slightly broadening near posterior margin; T1 with strong, longitudinal striae on posterior 0.5; T2 transverse, with anterior and posterior margins sinuate; T2 mostly sculptured, but with smooth areas centrally and along margins; ovipositor sheath 1.0–1.1× as long as metatibia; tegula and humeral complex brown; longitudinal strip on metasternum yellow (rarely bright yellow); pro- and mesocoxae yellow-brown to pale brown, metacoxa dark brown to black; pro- and mesofemora entirely yellow; metafemur mostly (except for anterior 0.1) and metatibia mostly (except for anterior 0.2–0.3) brown; body length: 2.10–2.58 mm; fore wing length: 2.28–2.85 mm. Among species with mostly sculptured T2, *D.scottmilleri* can be distinguished by its T1 and T2 shape and sculpture, anteromesoscutum and propodeum sculpture; legs color and overall body appearance less shiny and less smooth than closest (i.e., similar morphologically) species; F15 length is the main diagnostic character to separate it from *D.robpringlei*.

##### Distribution.

Costa Rica.

##### Biology.

Gregarious. Thyrididae: *Microscapaullula*.

##### DNA barcoding data.

BINBOLD:AAC2174 (41 sequences, 35 barcode compliant) and BOLD:ACE8823 (5 sequences, 3 barcode compliant).

##### Etymology.

Named in honor of Dr. Scott Miller of the National Museum of Natural History of the Smithsonian Institution, Washington, D.C., USA, for his two decades of steady and enthusiastic interest in, and support of, all the GDFCF and ACG activities as a member of the Board of Directors for the Guanacaste Dry Forest Conservation Fund in its integration with Area de Conservación Guanacaste, Costa Rica.

##### Notes.

Specimens from this species appear in BOLD as two different BINs (BOLD:AAC2174 and BOLD:ACE8823) that however have identical barcodes. Morphology is also similar so they are considered to be the same species here.

#### ﻿ Dolichogenidea shelleymcsweeneyae

Taxon classificationAnimaliaHymenopteraBraconidae

Fernandez-Triana & Boudreault sp. nov.

1834763F-77F5-59AC-AA11-58E6DB381F2C

https://zoobank.org/0FEE9E8A-DD73-434D-9B80-EC9D17F5D18B

Fig. 133A–F

##### Type material.

***Holotype*.** Costa Rica • Female, CNC; Guanacaste, Area de Conservación Guanacaste, Sector Cacao, Sendero Arenales; 10.92471, -85.46738; 1,080 m; 18.xii.2008; D.H. Janzen & W. Hallwachs leg.; Malaise trap; DHJPAR0031312. ***Paratypes*.** Costa Rica • 1 Female, CNC; DHJPAR0033964. 1 Female, 1 Male, CCDB; BIOUG49263-G08, BIOUG59060-H03.

**Figure 133.**
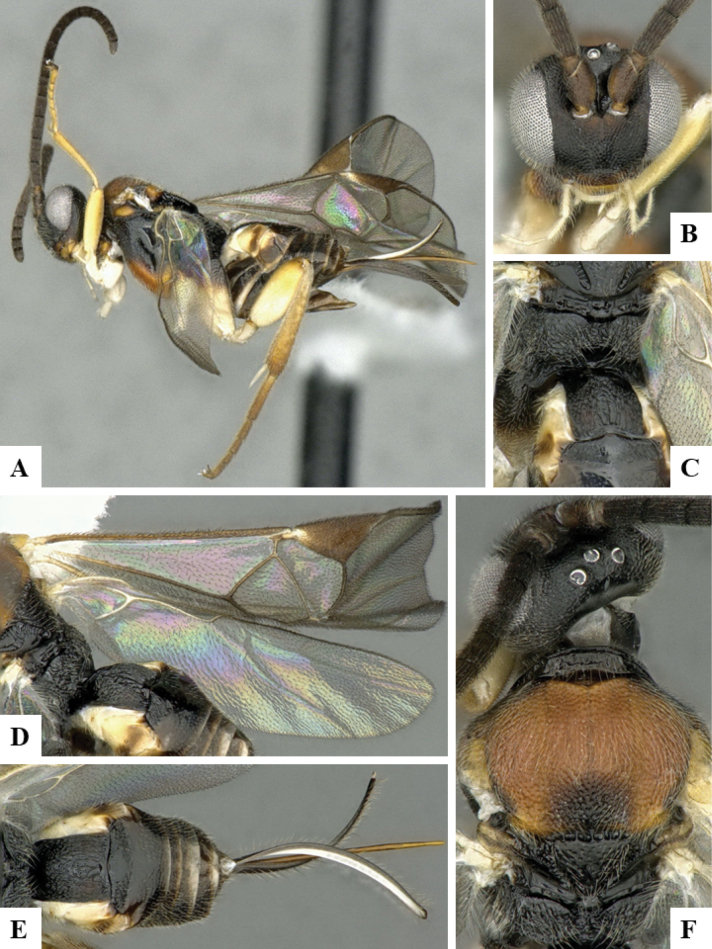
*Dolichogenideashelleymcsweeneyae* Fernandez-Triana & Boudreault holotype female DHJPAR0031312 **A** habitus, lateral **B** head, frontal **C** propodeum & T1, dorsal **D** wings **E** metasoma, dorsal **F** mesosoma, dorsal.

##### Diagnostic description.

T2 smooth; extensive orange coloration, including clypeus, anteromesoscutum (almost entirely, except for small dark spot near scutellar disc), antero-dorsal spot on mesopleuron, propleuron (partially), mesosternum (mostly) and two faint spots postero-laterally on T1; pro- and mesocoxae yellow-white, metacoxa mostly yellow-white with brown spot on anterior 0.1–0.2; body length: 2.97–3.19 mm; fore wing length: 3.13–3.16 mm; BINBOLD:AAM5736 which is 2.56% different from the nearest BIN in BOLD as of March 2022. The extensive orange coloration is sufficient to distinguish this species among all with smooth T2 and pale pro- and mesocoxae.

##### Distribution.

Costa Rica.

##### Biology.

No host data available.

##### DNA barcoding data.

BOLD:AAM5736 (5 sequences, 3 barcode compliant).

##### Etymology.

Named in honor of Mrs. Shelley McSweeney in recognition of her decade-plus of weathering the demands of being a major part of the family with Mr. Eric Palola, as the two-country Executive Director of the NGO Guanacaste Dry Forest Conservation Fund and its integration with the Costa Rican government’s Area de Conservación Guanacaste (ACG) in northwestern Costa Rica.

#### ﻿ Dolichogenidea sigifredomarini

Taxon classificationAnimaliaHymenopteraBraconidae

Fernandez-Triana & Boudreault sp. nov.

E1035B31-1F21-5365-A7E4-26C360690A5A

https://zoobank.org/E3159686-563F-41D1-A04D-5EA9933D77C7

Fig. 134A–F

##### Type material.

***Holotype*.** Costa Rica • Female, CNC; Alajuela, Area de Conservación Guanacaste, Sector San Cristobal, Sendero Corredor; 10.87868, -85.38963; 620 m; 07.v.2013; Gloria Sihezar leg.; Host: Depressariidae; Voucher code: DHJPAR0052330; Host voucher code: 13-SRNP-2323. ***Paratype*.** French Guiana • 1 Female, CNC; CNC491996.

**Figure 134.**
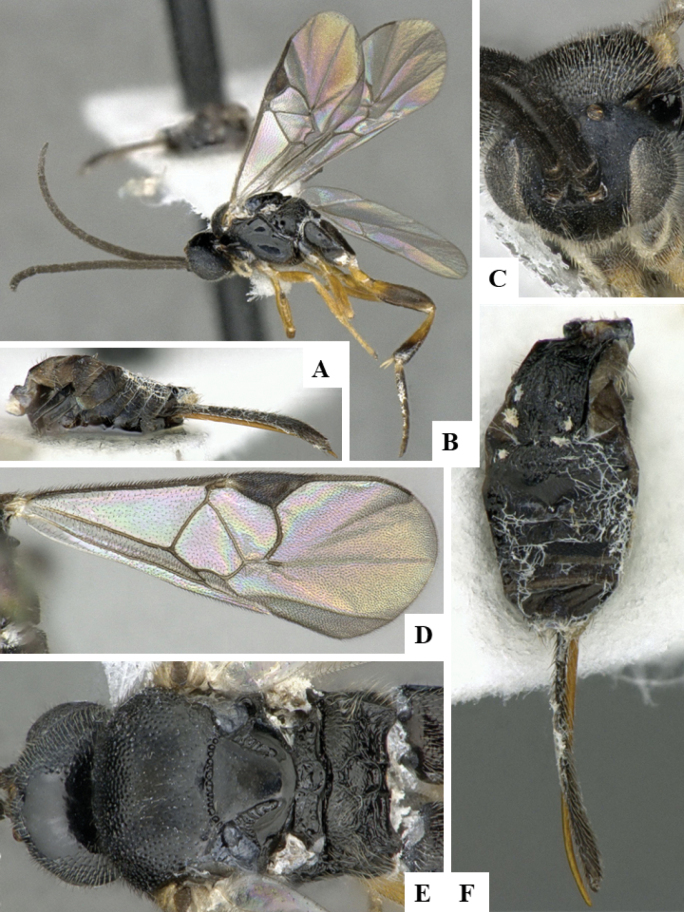
*Dolichogenideasigifredomarini* Fernandez-Triana & Boudreault holotype female DHJPAR0052330 **A** metasoma, lateral **B** habitus, lateral **C** head, frontal **D** fore wing **E** mesosoma, dorsal **F** metasoma, dorsal.

##### Diagnostic description.

Ocelli comparatively smaller, ocular ocellar line > 3.0× posterior ocellar line; anteromesoscutum more or less shiny but with well-marked punctures; scutellar disc smooth and shiny, without punctures; fore wing vein 2CU straight; T1 strongly sculptured on posterior 0.5; T2 mostly sculptured but smooth centrally or along margins; T2 transverse, its width at posterior margin ~ 3.5× its central length; ovipositor sheath length 1.0–1.1× metatibia length; humeral complex almost entirely brown, clearly darker than yellow tegula; pro- and mesocoxae mostly brown but posterior 0.2–0.3 yellow, metacoxa brown to dark brown; metatibia (except for darker spot on posterior 0.3) and part of metafemur (anterior 0.2 and posterior 0.1) yellow; body length: 2.60–2.78 mm; fore wing length: 2.90 mm. The shape and sculpture of T1 and T2, ocelli size, fore wing venation and color of tegula, humeral complex and legs distinguish this species among all others with T2 sculptured but transverse and dark coxae.

##### Distribution.

Costa Rica, French Guiana.

##### Biology.

Solitary. Reared from unidentified Depressariidae.

##### DNA barcoding data.

BINBOLD:ACI3397 (1 sequence, barcode compliant).

##### Etymology.

Named in honor of Sr. Sigifredo Marin of Liberia, Guanacaste Province, Costa Rica in recognition of 35 years and ongoing of steering the founding and growing Area de Conservación Guanacaste in its constant process of restoration and conserving its massive numbers of tropical species in the marine, dry forest, cloud forest and rain forest, and many tens of intergrades, following 400+ years of perturbation by European agriculture; he is currently the Field Director for GDFCF projects in ACG.

#### ﻿ Dolichogenidea stephmae

Taxon classificationAnimaliaHymenopteraBraconidae

Fernandez-Triana & Boudreault sp. nov.

D1F2E27C-BD75-5193-B220-EF457B0DB6FB

https://zoobank.org/2BCB3527-AE08-499E-9E30-A4CF5265AB7E

Fig. 135A–F

##### Type material.

***Holotype*.** Brazil • Female, CNC; São Paulo, Serra da Bocaina, São Jose do Barreiro; 1,650 m; xi.1968; F. M. Oliveira leg; Voucher code: CNC1179700.

**Figure 135.**
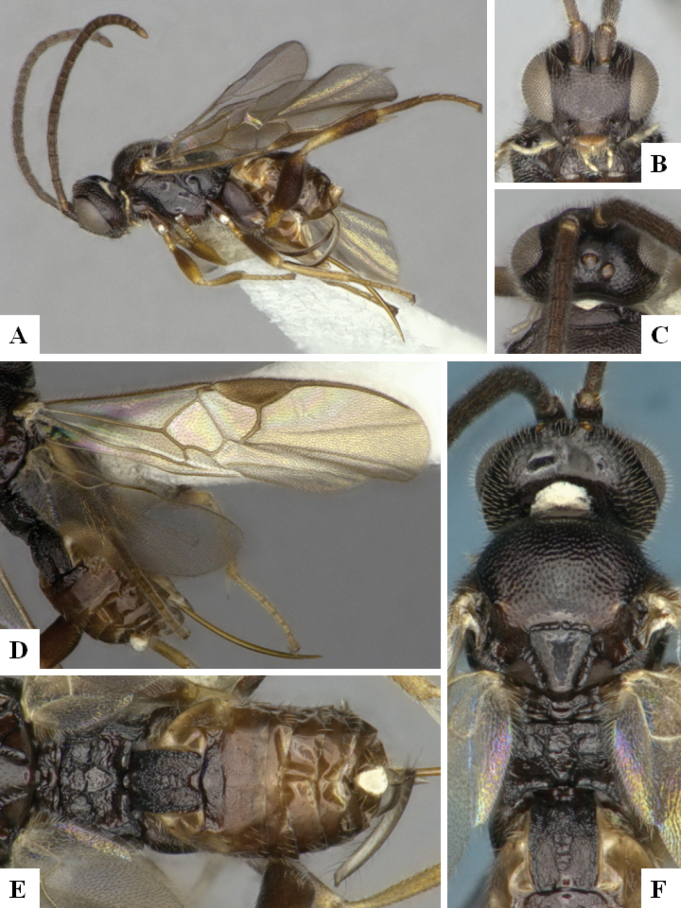
*Dolichogenideastephmae* Fernandez-Triana & Boudreault holotype female CNC1179700 **A** habitus, lateral **B** head, frontal **C** head, dorsal **D** wings **E** metasoma, dorsal **F** mesosoma, dorsal.

##### Diagnostic description.

Antenna shorter than body length; anteromesoscutum with comparatively strong punctures; propodeum entirely areolated and mostly sculptured; T1 parallel-sided and mostly strongly sculptured but with central area depressed and a polished knob centrally at posterior margin; T2 transverse and mostly sculptured but with central area smooth; ovipositor sheath slightly longer (1.1–1.2×) than metatibia length; all coxae brown; anterior 0.5 of profemur and entire meso- and metafemora brown; metatibia with anterior 0.5 yellow and posterior 0.5 brown; comparatively smaller size, body length: 2.10 mm; fore wing length: 2.30 mm. This is a very distinctive species based on sculpture of anteromesoscutum, propodeum and T1 and T2, shape of T1 and T2, short antenna, leg color, and body size.

##### Distribution.

Brazil (SP).

##### Biology.

No host data available.

##### DNA barcoding data.

No data.

##### Etymology.

The second author dedicates this species to her great sister-in-law Stéphanie Mayer. Stéphanie has been an inspiration by her sense of adventure, her love of travelling, and nice personality. The species’ name consists of the first five letters of “Stephanie” and the first letter of her last name “Mayer”.

#### ﻿ Dolichogenidea stevestroudi

Taxon classificationAnimaliaHymenopteraBraconidae

Fernandez-Triana & Boudreault sp. nov.

9B855DBD-CE16-5FFF-9048-F441FDD1361C

https://zoobank.org/BCF7EA7E-4D91-418D-930D-16F19321BD8D

Fig. 136A–F

##### Type material.

***Holotype*.** Costa Rica • Female, CNC; Alajuela, Area de Conservación Guanacaste, Sector Rincon Rain Forest, Sendero Venado; 10.89678, -85.27001; 420 m; 13.iii.2012; Pablo Umaña Calderon leg.; Host: gelJanzen01 Janzen22; Voucher code: DHJPAR0049415; Host voucher code: 12-SRNP-41147. ***Paratype*.** Costa Rica • 1 Female, CNC; DHJPAR0049394.

**Figure 136.**
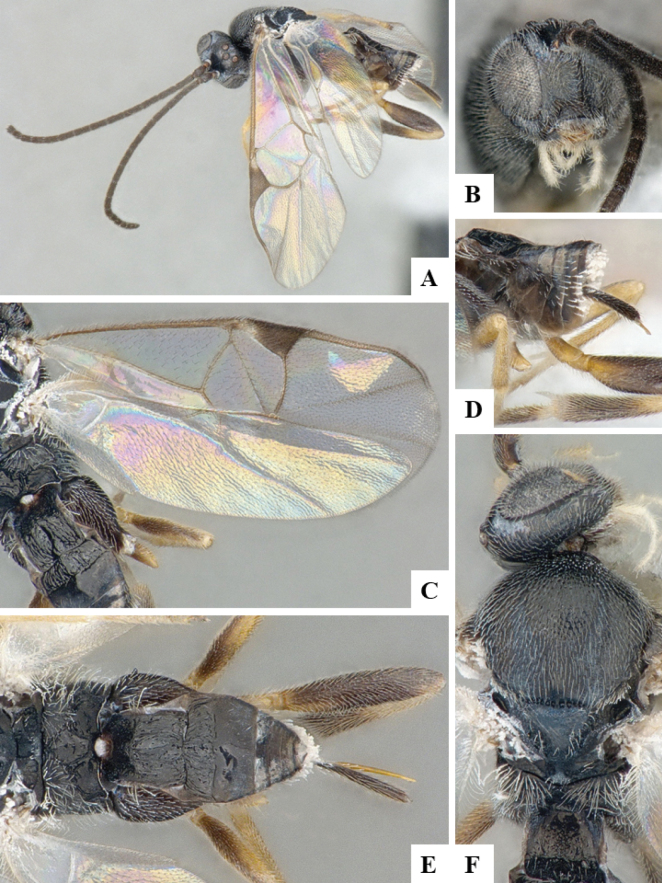
*Dolichogenideastevestroudi* Fernandez-Triana & Boudreault holotype female DHJPAR0049415 **A** habitus, lateral **B** head, frontal **C** wings **D** metasoma, lateral **E** metasoma, dorsal **F** mesosoma, dorsal.

##### Diagnostic description.

Propodeum areola well defined and bounded by carinae; T1 parallel-sided and comparatively broad (T1 length 1.2× T1 width at posterior margin); T1 with some sculpture on posterior 0.5; T2 transverse, its width at posterior margin > 3.0× its central length; T2 mostly sculptured, but with smooth areas centrally and laterally near posterior margin; hypopygium with several pleats; ovipositor sheath 0.6× as long as metatibia; tegula and humeral complex brown; profemur entirely yellow; pro- and mesocoxae brown, metacoxa dark brown to black; most of metafemur (except for anterior 0.2 yellow) and most of metatibia (except for anterior 0.4 yellow) brown; body length: 2.30 mm; fore wing length: 2.60 mm. This species is distinctive because of its short ovipositor sheath, T1 and T2 sculpture and body size and color.

##### Distribution.

Costa Rica.

##### Biology.

Solitary. Gelechiidae: gelJanzen01 Janzen22.

##### DNA barcoding data.

BINBOLD:ACB1629 (11 sequences, 10 barcode compliant).

##### Etymology.

Named in honor of Mr. Steve Stroud of Escazu, Costa Rica and Maine, USA, in recognition of his steady and enthusiastic interest in, and support of, all the GDFCF and ACG activities as a member of the Board of Directors for the Guanacaste Dry Forest Conservation Fund in its integration with Area de Conservación Guanacaste.

#### ﻿ Dolichogenidea susanabramsae

Taxon classificationAnimaliaHymenopteraBraconidae

Fernandez-Triana & Boudreault sp. nov.

122567F4-7031-527E-BB1A-F1CC416E5809

https://zoobank.org/0B17B8B2-F133-4A5C-8A76-74BED720E6AE

Fig. 137A–E

##### Type material.

***Holotype*.** Costa Rica • Female, CNC; Guanacaste, Area de Conservación Guanacaste, Sector Cacao, Cerro Pedregal; 10.92767, -85.47449; 1,080 m; 18.xii.2008; D. H. Janzen & W. Hallwachs leg.; Malaise trap; Voucher code: DHJPAR0031385. ***Paratype*.** Costa Rica • 1 Female, CNC; DHJPAR0031381.

**Figure 137.**
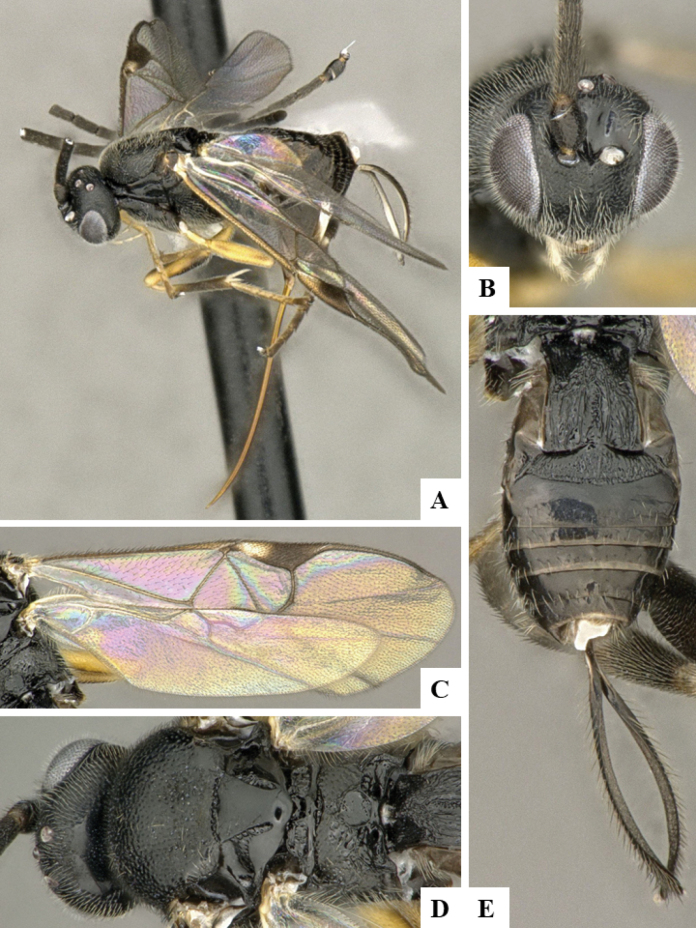
*Dolichogenideasusanabramsae* Fernandez-Triana & Boudreault holotype female DHJPAR0031385 **A** habitus, lateral **B** head, frontal **C** wings **D** mesosoma, dorsal **E** metasoma, dorsal.

##### Diagnostic description.

T1 strongly sculptured on posterior 0.5; T2 almost entirely sculptured; T2 very transverse, its width at posterior margin 4.0× its central length; ovipositor straight; pterostigma with pale spot 0.2–0.3 pterostigma length; all trochantelli yellow, profemur entirely and mesofemur mostly (except for longitudinal brown bands on margins) yellow; all coxae, metafemur and most of metatibia (except for anterior 0.3 which is yellow) dark brown; body length: 2.45–2.98 mm; fore wing length: 2.88–2.90 mm. This species could be difficult to key out, especially on couplet 10 where the interpretation of T2 sculpture could lead to different alternatives. While *D.susanabramsae* has T2 almost entirely sculptured (in that sense it would appear to run through the first half of couplet 9), its shape is very different from all other species with entirely and strongly sculptured T2, as *D.susanabramsae* has T2 very transverse (width at posterior margin 4.0× its central length). That character, as well as color of legs, pterostigma, and straight ovipositor separate the species from similar ones.

##### Distribution.

Costa Rica.

##### Biology.

Solitary. Gelechiidae: *Dichomeris* designatellaDHJ04.

##### DNA barcoding data.

BINBOLD:AAI6323 (4 sequences, 4 barcode compliant).

##### Etymology.

Named in honor of Susan Abrams (RIP) in recognition of her years of shepherding Costa Rican Natural History through the editorial and production process of the University of Chicago Press.

#### ﻿ Dolichogenidea teremariae

Taxon classificationAnimaliaHymenopteraBraconidae

Fernandez-Triana & Boudreault sp. nov.

009312CF-D716-587B-B2FF-05A971F1035B

https://zoobank.org/800DD62F-16DC-4BE8-A31C-F6BE0F50D7F7

Fig. 138A–F

##### Type material.

***Holotype*.** Costa Rica • Female, CNC; Guanacaste, Area de Conservación Guanacaste, Sector Cacao, Cerro Pedregal, 10.92767, -85.47449; 1,080 m; 22.xi.2008; D. H. Janzen & W. Hallwachs leg.; Malaise trap; Voucher code: DHJPAR0033770. ***Paratypes*.** Costa Rica • 19 Females, 1 Male, CNC; DHJPAR0033957, DHJPAR0033887, DHJPAR0033772, DHJPAR0033920, DHJPAR0033956, DHJPAR0033773, DHJPAR0033779, DHJPAR0033769, DHJPAR0033761, DHJPAR0033763, DHJPAR0033789, DHJPAR0033952, DHJPAR0033883, DHJPAR0033882, DHJPAR0033886, DHJPAR0033924, DHJPAR0033790, DHJPAR0033765, DHJPAR0033764.

**Figure 138.**
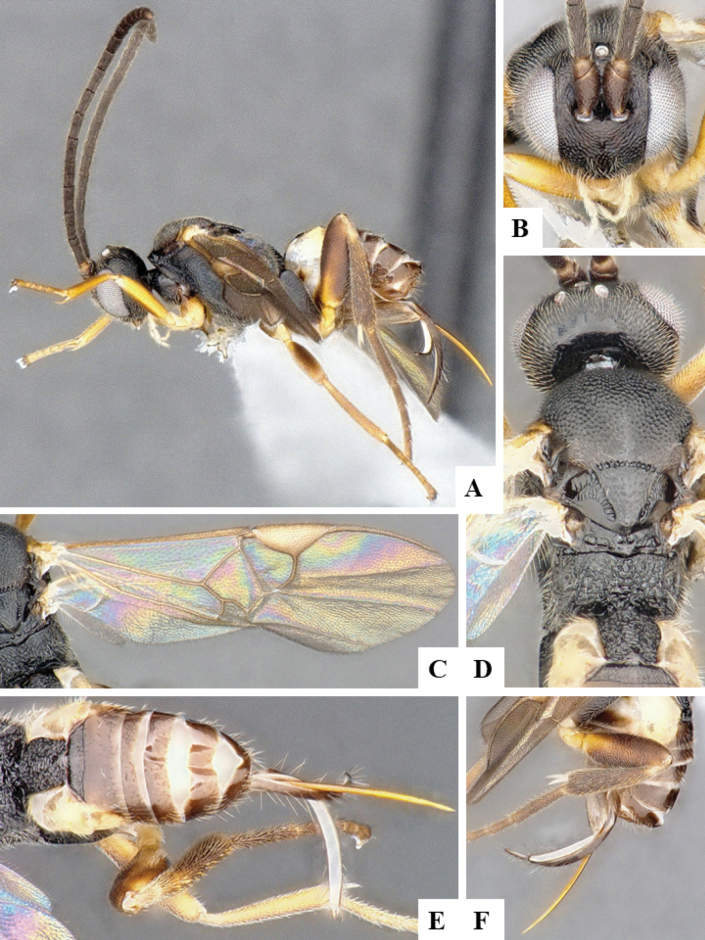
*Dolichogenideateremariae* Fernandez-Triana & Boudreault holotype female DHJPAR0033770 **A** habitus, lateral **B** head, frontal **C** wings **D** mesosoma, dorsal **E** metasoma, dorsal **F** ovipositor, lateral.

##### Other material.

Honduras • 1 Female, 4 undetermined sex, CCDB; BIOUG18845-E06, BIOUG25834-A10, BIOUG26881-B05, BIOUG26888-A01, BIOUG28324-G10.

##### Diagnostic description.

T1 distinctively narrowing from posterior 0.3 towards posterior margin; T2 transverse, its width at posterior margin > 3.5× its central length; T1 posterior 0.6 and T2 (mostly) sculptured; ovipositor sheath approx. same length than metatibia length; reddish yellow spots along posterior margins of propleuron, dorsal margin of pronotum, postero-lateral margins of anteromesoscutum, and mesosternum, all of which are clearly distinctive from rest of mostly black mesosoma; pterostigma yellow-white with thin brown margins; pro- and mesocoxae mostly yellow, metacoxa dark brown; body length and fore wing length: 2.20–2.50 mm. This species can be recognized by its distinctively narrowing T1, rather strong sculpture of T1 and T2, the yellow-white pterostigma and the reddish yellow spots on several areas of mesosoma.

##### Distribution.

Costa Rica, Honduras.

##### Biology.

No host data available.

##### DNA barcoding data.

BINBOLD: BOLD:AAM5842 (286 sequences, 265 barcode compliant).

##### Etymology.

Named after Teresita María (Tere Mari) Galliano Garay, for her long-standing work with the ACG fauna, labelling, mounting and inspecting thousands of Microgastrinae specimens

##### Notes.

The record from Honduras is based on five sequences in BOLD which match by 99.56% (approximately 3 bp of difference) with the almost 300 ACG sequences. Because we could not examine the Honduran specimens (other than examining a single photo available in BOLD, which also matches well with the ACG specimen) they are not included as paratypes but as other material.

#### ﻿ Dolichogenidea tiboshartae

Taxon classificationAnimaliaHymenopteraBraconidae

Fernandez-Triana & Boudreault sp. nov.

5FEEEA81-DC6E-549E-B828-14BF3CEB7459

https://zoobank.org/27FA10CC-883B-4697-A9BB-ADFE78CB0570

Figs 139A–F , 164B

##### Type material.

***Holotype*.** Costa Rica • Female, CNC; Alajuela, Area de Conservación Guanacaste, Sector Rincon Rain Forest, Vado Rio Francia; 10.9009, -85.2891; 400 m; 27.x.2007; D. H. Janzen & W. Hallwachs leg.; Voucher code: DHJPAR0026105. ***Paratypes*.** Costa Rica • 1 Female, 1 Male, CNC; DHJPAR0026122, DHJPAR0051050.

**Figure 139.**
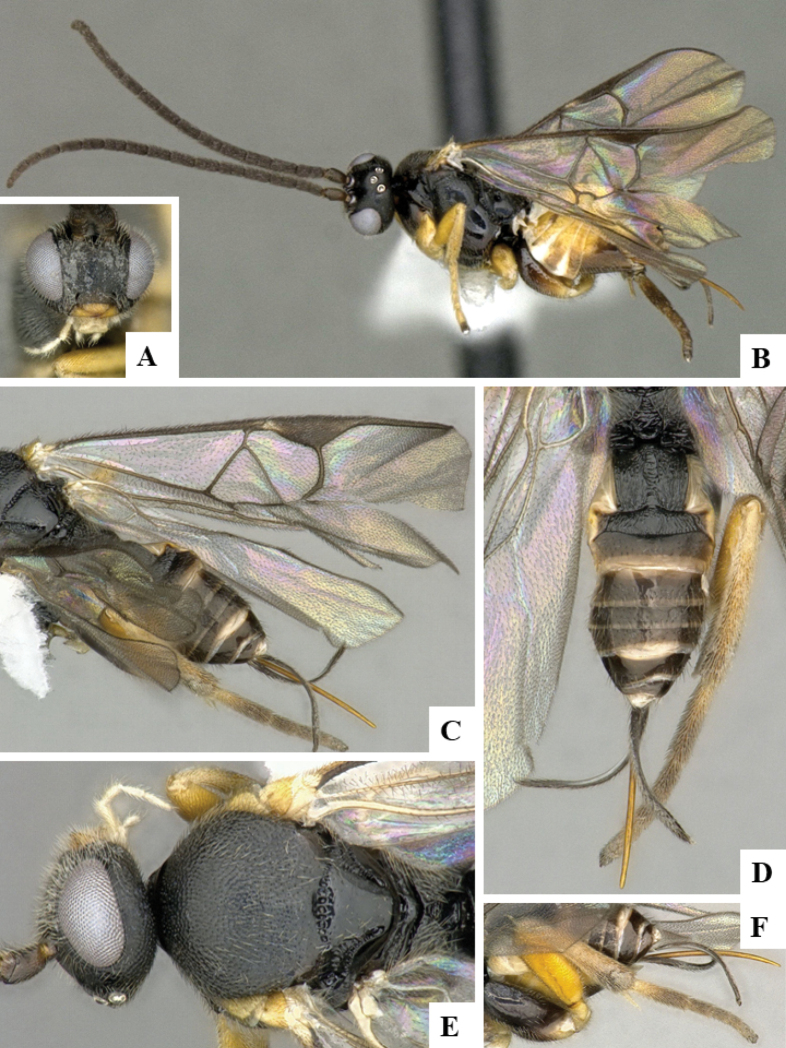
*Dolichogenideatiboshartae* Fernandez-Triana & Boudreault holotype female DHJPAR0026105 **A** head, frontal **B** habitus, lateral **C** wings **D** metasoma, dorsal **E** mesosoma, dorsal **F** ovipositor, lateral.

##### Diagnostic description.

F2 length 2.5× F14 length; hind legs tarsal claws with single spine; T1 and T2 heavily sculptured with strong longitudinal striae; T1 length < 1.5× T1 width at posterior margin; T2 more transverse, its width at posterior margin > 3.0× its central length; T2 with smooth spot centrally near posterior margin; ovipositor sheath 1.0–1.1× metatibia length; pterostigma mostly brown, at most with small pale spot on proximal 0.1–0.2; metacoxa almost entirely dark brown (very small yellow spot on posterior 0.1); metatibial spurs entirely yellow; metatarsus mostly brown; body length: 2.70–2.90 mm; fore wing length: 2.90–2.95 mm. The sculpture of T2, color of pterostigma, metacoxa, metatibia, metatibial spurs, the length of F15 and the tarsal claws with a single spine separate *D.tiboshartae* from all other species with heavily sculptured T1 and T2, T1 comparatively broad and T2 rectangular and yellow metatibia. The only species closely similar morphologically is *D.ninamasisae* which has different color of metatarsus, different T2 shape, slightly larger body size and slightly longer ovipositor sheath.

##### Distribution.

Costa Rica.

##### Biology.

Solitary. Depressariidae: elachJanzen01 Janzen409; Gelechiidae: Dichomeris Janzen76, gelJanzen01 Janzen116.

##### DNA barcoding data.

BINBOLD:AAC5949: (15 sequences, 13 barcode compliant).

##### Etymology.

Named in honor of Mrs. Tishisia Boshart of San Jose and Guanacaste, Costa Rica, in recognition of her persistent and diligent care and management of her household and family with Mr. Alejandro Masis, the Director of Area de Conservación Guanacaste (ACG), and her consistently high-quality recognition posters for honorees with patronyms of new species from ACG.

#### ﻿ Dolichogenidea timrichi

Taxon classificationAnimaliaHymenopteraBraconidae

Fernandez-Triana & Boudreault sp. nov.

A570A9A0-6081-5DA4-85D1-F5FDC92BE589

https://zoobank.org/0EBB8A2E-DD82-41D1-9998-503C6DA1CDC9

Fig. 140A–F

##### Type material.

***Holotype*.** Costa Rica • Female, CNC; Alajuela, Area de Conservación Guanacaste, Sector Rincon Rain Forest, Vado Rio Francia; 10.90093, -85.28915; 400 m; 29.vi.2007; D. H. Janzen & W. Hallwachs leg.; Malaise trap; Voucher code: DHJPAR0025530.

**Figure 140.**
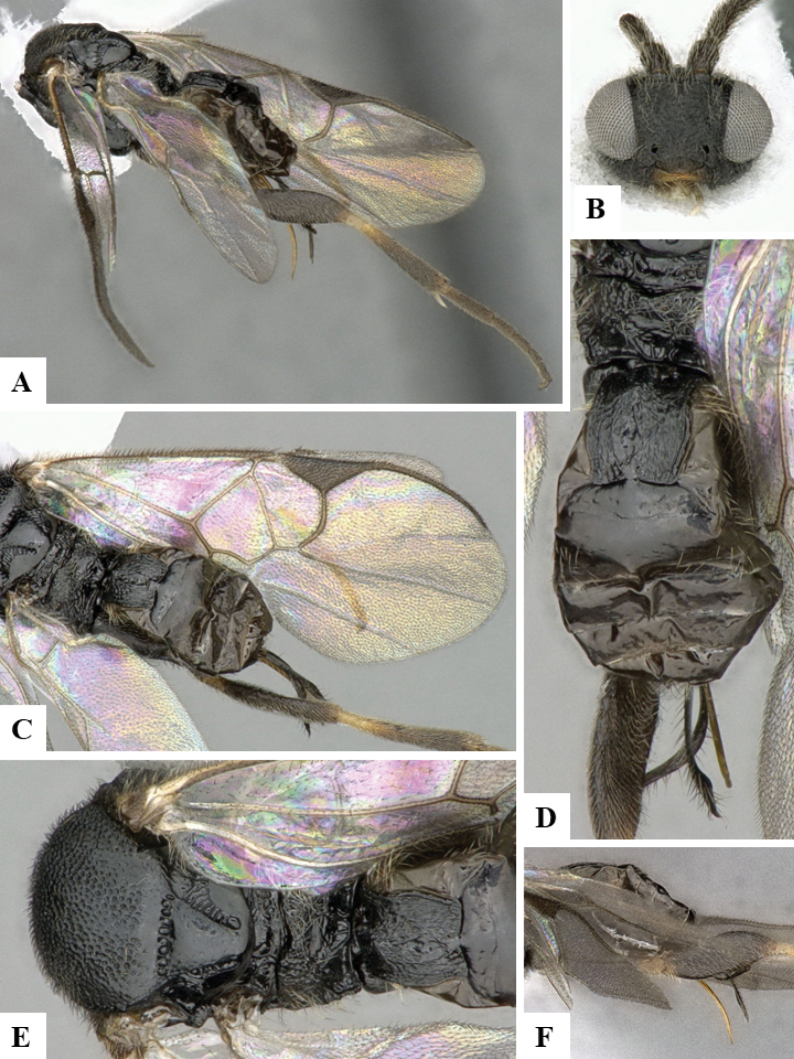
*Dolichogenideatimrichi* Fernandez-Triana & Boudreault holotype female DHJPAR0025530 **A** habitus, lateral **B** head, frontal **C** wings **D** metasoma, dorsal **E** mesosoma, dorsal **F** ovipositor, lateral.

##### Diagnostic description.

Anteromesoscutum mostly covered with relatively coarse punctures; propodeum areola complete; propodeum mostly sculptured, with lateral striation on most areas between carinae; T1 mostly parallel-sided but slightly narrowing near posterior margin; T1 mostly sculptured on posterior 0.5; T2 smooth; tegula and humeral complex brown; pedicel, most of mesofemur and entire mesosternum dark brown to black; all coxae, metafemur and most of metatibia (except for anterior 0.2 which is white) brown to dark brown; body length: 2.40 mm; fore wing length: 2.60 mm. Among species with smooth T2 *D.timrichi* can be distinguished by T1 sculpture, propodeum sculpture and carination pattern, body size, and mostly dark coloration.

##### Distribution.

Costa Rica.

##### Biology.

No host data available.

##### DNA barcoding data.

BINBOLD:AAJ1390 (2 sequences, 2 barcode compliant).

##### Etymology.

Named in honor of Mr. Tim Rich of California in recognition of his decade-plus and continuing steady financial administration of the GDFCF endowments for GDFCF programs in and around Area de Conservación Guanacaste.

##### Notes.

The BIN includes a sequence from Mexico; however, it is 1.95% different from the ACG sequence, therefore we consider best not to include the Mexican specimen as part of this species until it can be examined.

#### ﻿ Dolichogenidea tomdaleyi

Taxon classificationAnimaliaHymenopteraBraconidae

Fernandez-Triana & Boudreault sp. nov.

7050AD2E-08F6-5508-B736-0FFB65B73AF0

https://zoobank.org/2431C646-F08F-43A7-9863-B8FB3C4E7BBB

**Figure 141.**
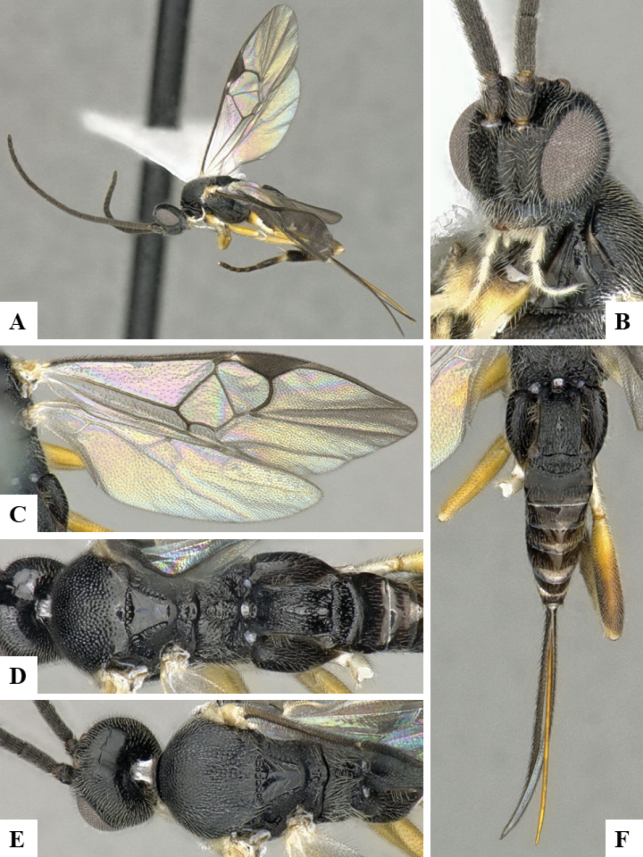
*Dolichogenideatomdaleyi* Fernandez-Triana & Boudreault holotype female DHJPAR0053027 **A** habitus, lateral **B** head, fronto-lateral **C** wings **D** propodeum & T1–T3, dorsal **E** mesosoma, dorsal **F** metasoma, dorsal.

**Figure 142.**
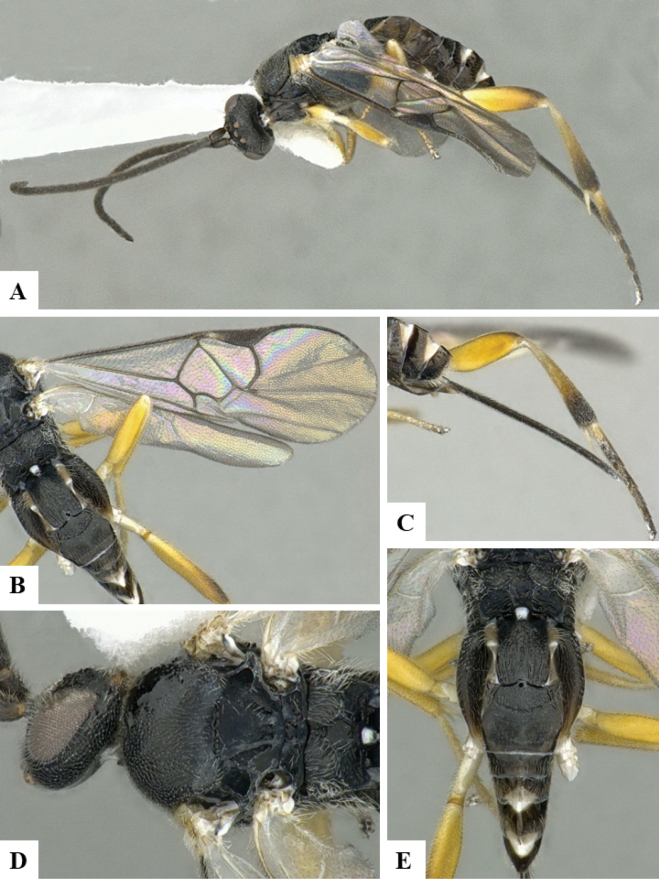
*Dolichogenideatomdaleyi* Fernandez-Triana & Boudreault paratype female DHJPAR0051077 **A** habitus, lateral **B** wings **C** ovipositor sheaths & hind leg, lateral **D** mesosoma, dorsal **E** metasoma, dorsal.

Figs 141A–F , 142A–E , 165A

##### Type material.

***Holotype*.** Costa Rica • Female, CNC; Alajuela, Area de Conservación Guanacaste, Sector San Cristobal, Sendero Perdido; 10.8794, -85.3861; 620 m; 12.viii.2013; Elda Araya leg.; Host: *Stenoma* Janzen699; DHJPAR0053027. [The label of the holotype has a wrong date printed (08/01/2013) and it does not specify the host information, both data were checked from BOLD and http://janzen.sas.upenn.edu/caterpillars/database.htm]. ***Paratypes*.** Costa Rica • 12 Females 1 Male, CNC; DHJPAR0051077, DHJPAR0049871, DHJPAR0048179, DHJPAR0047198, DHJPAR0041613, DHJPAR0049315, DHJPAR0051123, DHJPAR0020706, DHJPAR0050138, DHJPAR0054862, DHJPAR0020677, DHJPAR0020676, DHJPAR0020793.

##### Diagnostic description.

T1 and T2 heavily sculptured with strong longitudinal striae; T1 comparatively thin and mostly parallel-sided but posterior 0.1–0.3 slightly narrowing towards posterior margin; T2 broadly rectangular in shape (with posterior margin slightly arcuate); ovipositor sheath ~ 2.0× metatibia length; metacoxa partially yellow partially dark brown; body length: 2.60–3.10 mm; fore wing length: 2.72–3.13 mm; BINBOLD:AAD8952, which is 4.97% different from the nearest BIN in BOLD as of March 2022. The ovipositor sheath length, body size and metacoxa color separate it from all other species with T1 and T2 heavily sculptured and T1 comparatively thin.

##### Distribution.

Costa Rica.

##### Biology.

Solitary. Reared from many host species within three genera of Depressariidae (Stenomatinae): *Antaeotrichaincrassata*, *Antaeotricha* cirrhoxanthaDHJ02, *Antaeotricha* similisEPR01, *Antaeotricha* similisEPR02, *Antaeotricha* BioLep46, *Antaeotricha* Janzen23, *Antaeotricha* Janzen31, *Antaeotricha* Janzen77, *Antaeotricha* Janzen106, *Antaeotricha* Janzen146, *Antaeotricha* Janzen290, *Antaeotricha* Janzen292DHJ0, *Antaeotricha* Janzen364, *Antaeotricha* Philips01, *Chlamydastis* Janzen04, *Stenoma* Janzen18, *Stenoma* Janzen58, *Stenoma* Janzen199, *Stenoma* Janzen699.

##### DNA barcoding data.

BINBOLD:AAD8952 (62 sequences, 58 barcode compliant).

##### Etymology.

Named in honor of Mr. Tom Daley of Moraga, California, USA in recognition of his years of administration of financial and tax affairs for the Guanacaste Dry Forest Conservation Fund and its conservation-based integration with Area de Conservación Guanacaste in northwestern Costa Rica.

#### ﻿ Dolichogenidea tristanpalolai

Taxon classificationAnimaliaHymenopteraBraconidae

Fernandez-Triana & Boudreault sp. nov.

EF69D4E4-47EE-5A37-9F47-A86D0C6CCD67

https://zoobank.org/0907E67A-8C1A-423E-8A0A-919973C4A6DA

Fig. 143A–E

##### Type material.

***Holotype*.** Costa Rica • Female, CNC; Guanacaste, Area de Conservación Guanacaste, Sector Santa Rosa, Area Administrativa; 10.83764, -85.61871; 295 m; 25.xii.2008; D. H. Janzen & W. Hallwachs leg.; Malaise trap; Voucher code: DHJPAR0031859. ***Paratypes*.** Costa Rica • 1 Female, 2 Males, CNC; DHJPAR0012735, DHJPAR0024742, DHJPAR0031860.

**Figure 143.**
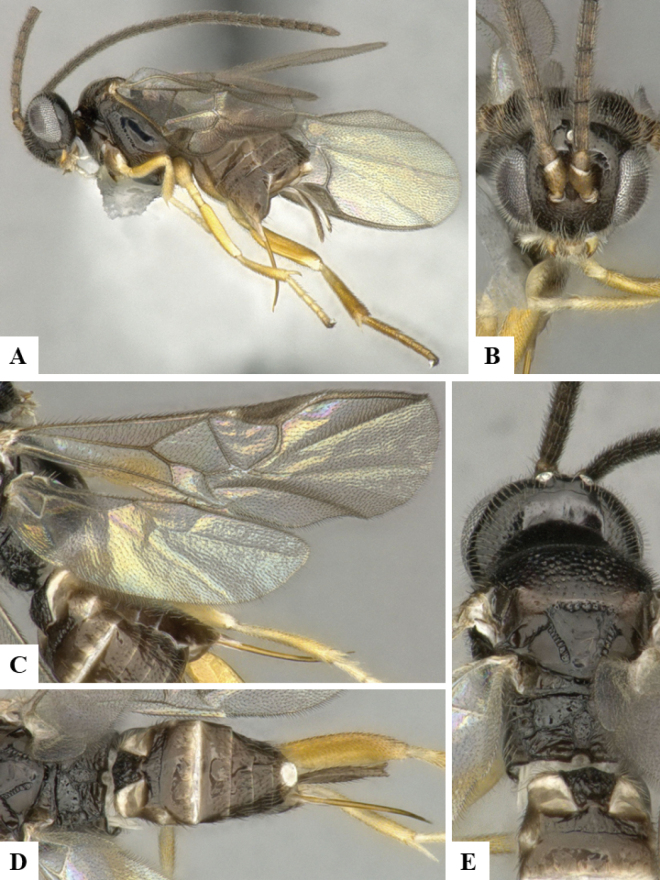
*Dolichogenideatristanpalolai* Fernandez-Triana & Boudreault holotype female DHJPAR0031859 **A** habitus, lateral **B** head, frontal **C** wings **D** metasoma, dorsal **E** mesosoma, dorsal.

##### Diagnostic description.

T1 strongly narrowing towards posterior margin, its length medially ~ 4.0–5.0× its width at posterior margin and its width at anterior margin 2.0× its width at posterior margin; T2 smooth; pro- and mesocoxae mostly yellow to yellow-pale brown, metacoxa mostly brown; metafemur and metatibia yellow; humeral complex, all laterotergites, sternites and hypopygium brown to dark brown; body length: 1.76–1.80 mm; fore wing length: 1.90–2.08 mm. The color of humeral complex, coxae, and lateral and ventral areas of metasoma, as well as the shape of T1 distinguish this species among all with smooth T2 and pale pro- and mesocoxae.

##### Distribution.

Costa Rica.

##### Biology.

No host data available.

##### DNA barcoding data.

BINBOLD:AAI9740 (5 sequences, 4 barcode compliant).

##### Etymology.

Named in honor of Mr. Tristan Palola in recognition of his decade-plus of weathering the demands of being a major part of the Palola family with Mr. Eric Palola, as the two-country Executive Director of the NGO Guanacaste Dry Forest Conservation Fund and its integration with the Costa Rican government’s Area de Conservación Guanacaste (ACG) in northwestern Costa Rica.

#### ﻿ Dolichogenidea tucuman

Taxon classificationAnimaliaHymenopteraBraconidae

Fernandez-Triana & Boudreault sp. nov.

FAB35336-C927-503F-B43A-6767C08C54E1

https://zoobank.org/8ABA908B-3F59-4CD0-BD84-A6E29DC9E43F

Fig. 144A–F

##### Type material.

***Holotype*.** Argentina • Female, CNC; Tucuman, Reserva experimental Horco Molle; viii.1968; C. C. Porter leg.; Voucher code: CNC1179678.

**Figure 144.**
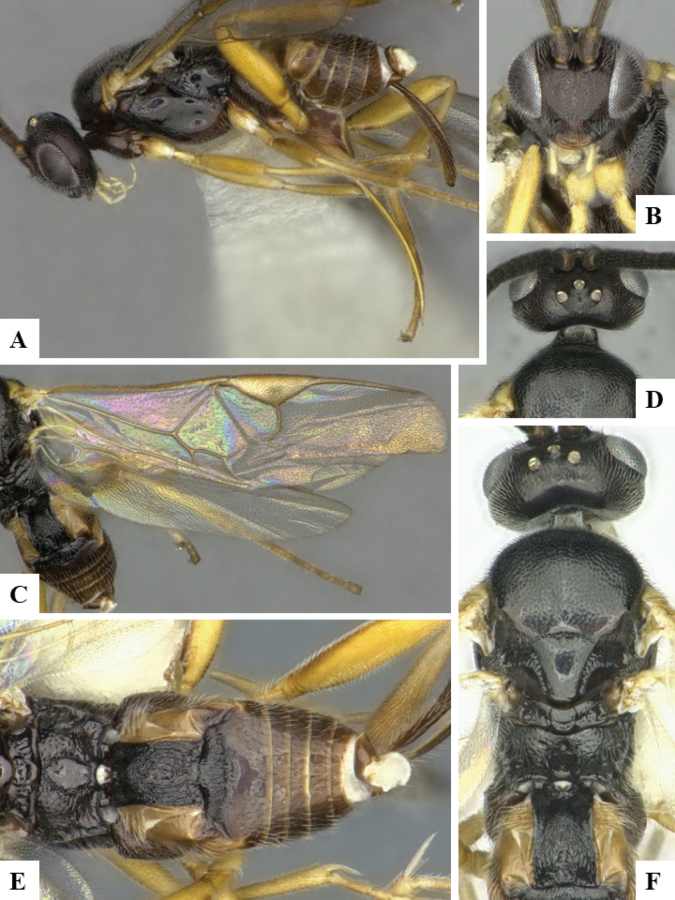
*Dolichogenideatucuman* Fernandez-Triana & Boudreault holotype female CNC1179678 **A** habitus, lateral **B** head, frontal **C** wings **D** head, dorsal **E** metasoma, dorsal **F** mesosoma, dorsal.

##### Diagnostic description.

Anteromesoscutum mostly with relatively shallow punctures; scutellar disc smooth and shiny, without punctures; mesopleuron and metapleuron entirely to almost entirely smooth or with few, shallow punctures; T1 more or less parallel-sided, its length medially < 2.0× its width at posterior margin; T2 entirely sculptured; T2 comparatively transverse, its length medially ~ 4.0× its width at posterior margin; ovipositor sheath 0.9× as long as metatibia; scape brown, same color than flagellomeres; tegula and humeral complex yellow; pterostigma mostly bright yellow-white (but with central, darker spot which is pale brown); pro- and mesocoxae yellow, metacoxa mostly dark brown; metafemur and metatibia entirely yellow; T1 entirely black; body length: 2.50 mm; fore wing length: 2.93 mm. Among all species with pale coloration of legs and with T1 and T2 not strongly sculptured (but notice that T2 is entirely sculptured, just not strongly), *D.tucuman* can be recognized by its more or less parallel-sided T1, rather transverse T2, smooth scutellar disc, and color of tegula and humeral complex. The new species resembles *D.phthorimaeae* but *D.tucuman* has longer ovipositor sheaths and different color of scape and pterostigma.

##### Distribution.

Argentina.

##### Biology.

No host data available.

##### DNA barcoding data.

No data.

##### Etymology.

Named after the province of the type locality in Argentina.

#### ﻿ Dolichogenidea verobrondexae

Taxon classificationAnimaliaHymenopteraBraconidae

Fernandez-Triana & Boudreault sp. nov.

922162F0-0A56-5EC7-AA1F-CF21733DDF07

https://zoobank.org/C037DA61-4812-4AB9-BB2C-ABC30028F529

Fig. 145A–F

##### Type material.

***Holotype*.** Venezuela • Female, CNC; Merida, Tabay Lamucuy, streamside meadow; 18.vi-2.viii.1989; S. & J. Peck leg.; Malaise trap; Voucher code: CNC1179692.

**Figure 145.**
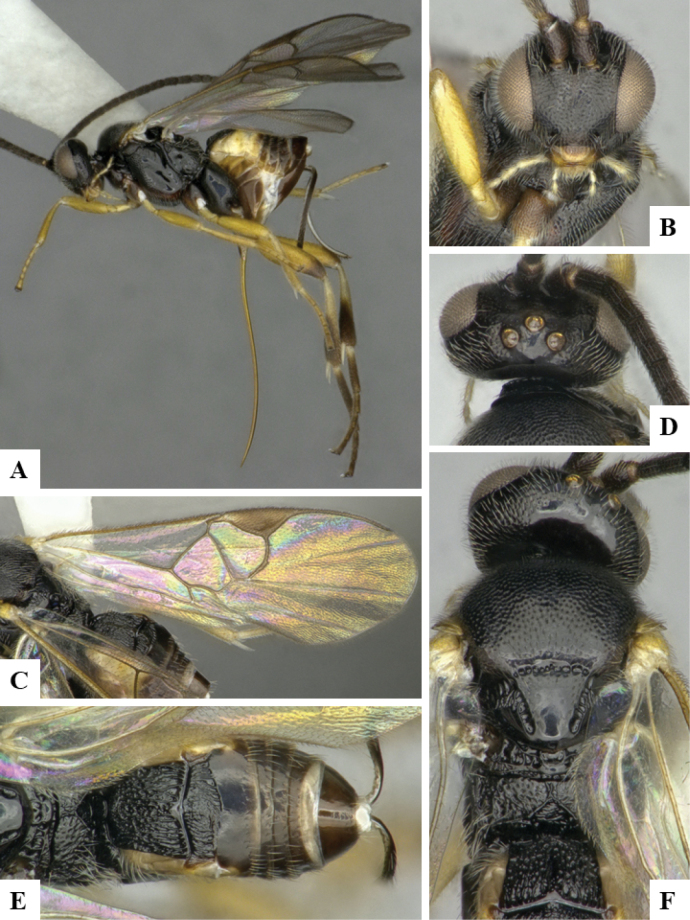
*Dolichogenideaverobrondexae* Fernandez-Triana & Boudreault holotype female CNC1179692 **A** habitus, lateral **B** head, frontal **C** wings **D** head, dorsal **E** metasoma, dorsal **F** mesosoma, dorsal.

##### Diagnostic description.

F15 length/width 1.1; ocular ocellar line 1.4× posterior ocellar line; T1 and T2 heavily sculptured with strong longitudinal striae covering entire surface of T2 and most of T1; T1 evenly broadening towards posterior margin; T2 broadly rectangular and large, covering most surface of tergum; tegula and humeral complex yellow; pterostigma brown with small pale spot at base; metatibia dark brown to black on posterior 0.5; body length: 2.70 mm; fore wing length: 3.00 mm. Among all species with heavily sculptured T1 and T2, this species is distinctive by its body size, metatibia and pterostigma color, cubic F15 (1.1× as long as wide), and ocelli size.

##### Distribution.

Venezuela.

##### Biology.

No host data available.

##### DNA barcoding data.

No data.

##### Etymology.

The second author dedicates this species in honor of her good friend Véronique Brondex. Véronique has been an inspiration by her kindness, joyful personality, courage, and aliveness. She is always ready for new adventures! The first part of the species’ name “vero” is the shortened version of Veronique.

#### ﻿ Dolichogenidea virgendelparamo

Taxon classificationAnimaliaHymenopteraBraconidae

Fernandez-Triana & Boudreault sp. nov.

29AAE435-C5B9-5160-A03D-84D286FC58E0

https://zoobank.org/DE1F9C0E-4CA9-40ED-98E8-C9532B9EB129

Fig. 146A–F

##### Type material.

***Holotype*.** Ecuador • Female, CNC; Napo, Quito-Baeza road; 4,000 m; 10.ii.1983; L. Masner & M. Sharkey leg.; Voucher code: CNC1179670.

**Figure 146.**
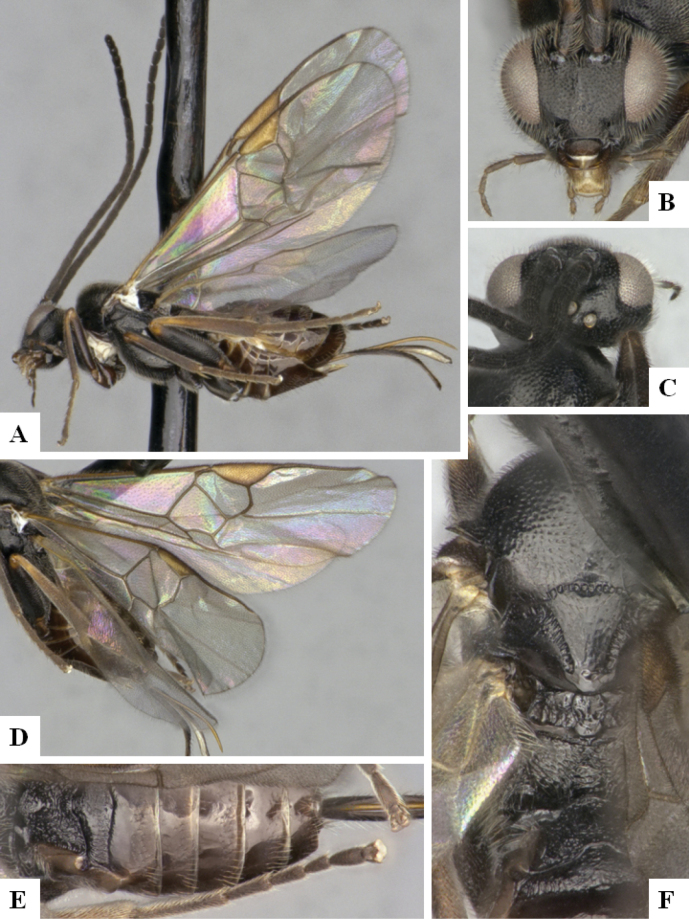
*Dolichogenideavirgendelparamo* Fernandez-Triana & Boudreault holotype female CNC1179670 **A** habitus, lateral **B** head, frontal **C** head, dorsal **D** wings **E** metasoma, dorsal **F** mesosoma, dorsal.

***Paratypes*.** Ecuador • 3 Females, 10 Males, CNC; CNC1179676, CNC1179691, CNC1179697, CNC1179707, CNC1179760, CNC1179762, CNC1179803, CNC1179823, CNC1179824, CNC1179825, CNC1179827, CNC1179828, CNC1179899.

##### Diagnostic description.

T1 broadening towards posterior margin, 1.2× as long as width at posterior margin; T1 with strong, longitudinal striae on posterior 0.5; T2 mostly sculptured (but centrally smooth, with one female having T2 almost entirely smooth), with anterior and posterior margin strongly sinuate; ovipositor sheath 1.0–1.1× as long as metatibia; comparatively very dark-colored species, with all legs entirely dark brown to black (except for anterior 0.2–0.3 of metatibia which is yellow-brown); palpi, tegula and humeral complex dark brown; pterostigma mostly yellow but with brown margins, most veins in fore wing brown; body length: 3.20–3.50 mm; fore wing length: 3.50–3.80 mm. Among species with T1 and T2 sculptured (but with T2 not entirely sculptured and very transverse), this species is characterized by its very dark coloration, pterostigma mostly yellow with brown margins and body size, as well as characteristic sculpture and shape of T1. The species *D.lacochaparamo* is morphologically similar but has paler colored palpi, tegula humeral complex and pterostigma, smaller body size and more strongly sculptured T2.

##### Distribution.

Ecuador.

##### Biology.

No host data available.

##### DNA barcoding data.

No data.

##### Etymology.

Named after the lookout “Virgen Del Paramo”, which at 4,000–4,100 m is the highest point in the road from Quito to Baeza and the locality where most of the specimens from this species have been collected.

#### ﻿ Dolichogenidea weaversway

Taxon classificationAnimaliaHymenopteraBraconidae

Fernandez-Triana & Boudreault sp. nov.

E8FA64CA-74B2-5CA9-B26C-CC8803BB20BB

https://zoobank.org/FD67D149-4B50-416E-A2CD-603AC5EEDF55

Fig. 147A–F

##### Type material.

***Holotype*.** Costa Rica • Female, CNC; Guanacaste, Area de Conservación Guanacaste, Sector Santa Rosa, Sendero Natural; 10.83575, -85.61253; 290 m; 06.ii.1981; D. H. Janzen leg.; Host: *Telphusa* BioLep476; Voucher code: CNC1196849; Host voucher code: 81-SRNP-107.

**Figure 147.**
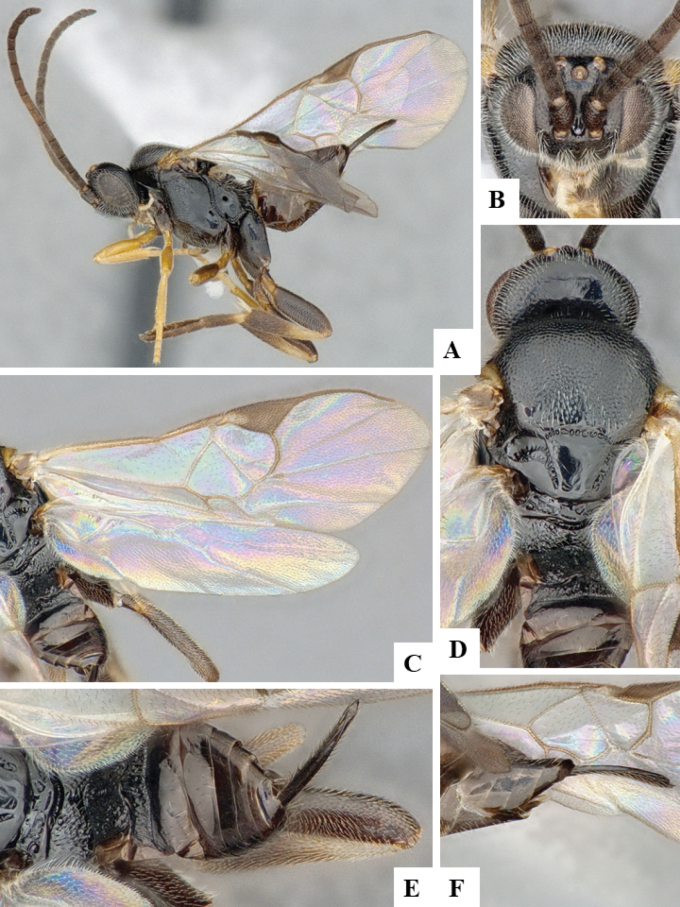
*Dolichogenideaweaversway* Fernandez-Triana & Boudreault holotype female CNC1196849 **A** habitus, lateral **B** head, frontal **C** wings **D** mesosoma, dorsal **E** metasoma, dorsal **F** metasoma, lateral.

##### Diagnostic description.

F15 cubic, its length 1.1× its width; posterior 0.5–0.6 of T1 and T2 mostly with strong sculpture, usually longitudinal striae covering entire surface (but T2 with small polished area centrally); T1 slightly broadening posteriorly; T2 comparatively very transverse but with anterior margin arcuate; ovipositor sheath slightly shorter than metatibia length; tegula yellow; mesosternum entire black; pro- and mesocoxae brown, metacoxa dark brown to black; metafemur dark brown; metatibia brown on posterior 0.5; body length: 2.30 mm; fore wing length: 2.63 mm. This species has strong sculpture (usually longitudinal striae) covering posterior 0.5–0.6 of T1 and most of T2. However, unlike the majority of species with similarly strong sculpture, T2 has a central area which is smooth and also T2 is very transverse with its anterior margin strongly arcuate. Because of that unique shape and sculpture pattern of T2, as well as its metafemur color, it can be separate from all the species with entirely and strongly sculptured T2 which is not transverse, as well as all the species with smooth T2 and/or broad T2.

##### Distribution.

Costa Rica.

##### Biology.

Gelechiidae: *Telphusa* BioLep476.

##### DNA barcoding data.

No data.

##### Etymology.

Named in recognition of the Weaver’s Way grocery store of Mt. Airy, Philadelphia, for years of food provisioning for Dan Janzen and Winnie Hallwachs during the inventory.

#### ﻿ Dolichogenidea yeimycedenoae

Taxon classificationAnimaliaHymenopteraBraconidae

(Fernandez-Triana, 2016)

55A01B75-9618-5BC0-B231-0BCDABE5621D

Fig. 148A–F

##### Notes.

Full details for this species in Fernandez-Triana et al. (2016). See also the key and Table 1 above.

**Figure 148.**
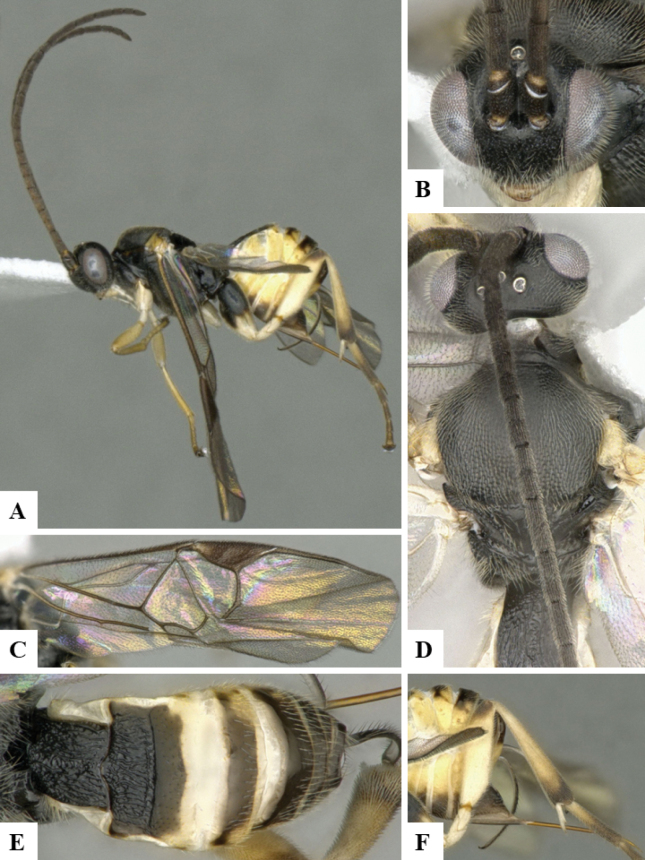
*Dolichogenideayeimycedenoae* (Fernandez-Triana) holotype female DHJPAR0031496 **A** habitus, lateral **B** head, frontal **C** wings **D** mesosoma, dorsal **E** metasoma, dorsal **F** ovipositor.

#### ﻿ Dolichogenidea yungas

Taxon classificationAnimaliaHymenopteraBraconidae

Fernandez-Triana & Boudreault sp. nov.

501043D4-DDC9-5E93-9E94-04ED7C93E0E7

https://zoobank.org/4F60DF20-27C5-42E6-879C-EF171491B193

Fig. 149A–G

##### Type material.

***Holotype*.** Bolivia • Female, CNC; La Paz, Yungas, 50 km North of La Paz; 2,200 m; 27.i.1973; J. Helava leg.; Voucher code: CNC1196945.

**Figure 149.**
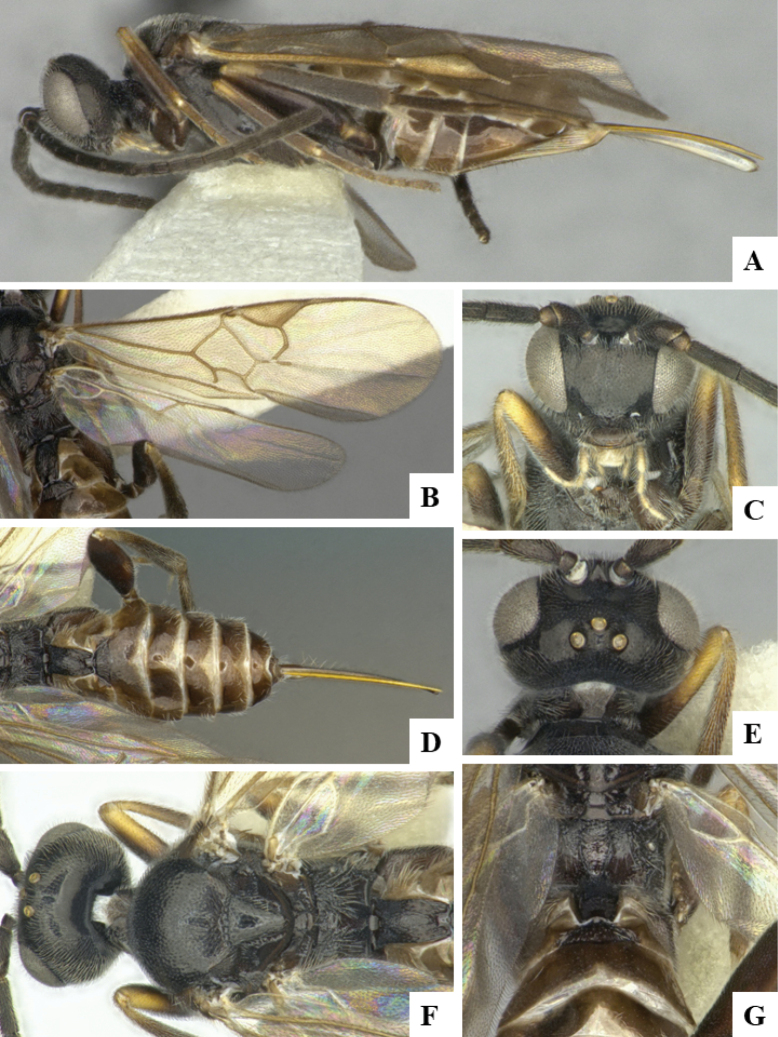
*Dolichogenideayungas* Fernandez-Triana & Boudreault holotype female CNC1196945 **A** habitus, lateral **B** wings **C** head, frontal **D** metasoma, dorsal **E** head, dorsal **F** mesosoma, dorsal **G** propodeum, dorsal.

##### Diagnostic description.

F15 cubic (around same length than width); T1 more or less parallel-sided but on posterior 0.3 narrowing towards posterior margin so that T1 length is 3.0× its width at posterior margin; T1 strongly sculptured on posterior 0.7; T2 shape trapezoidal but rather narrow, barely wider than T1; T2 with strong longitudinal striae; tegula and humeral complex brown; wings slightly infumated; pterostigma mostly yellow-white but with thin brown margins; all legs entirely brown to dark brown (except for very small, paler spots on posterior 0.1 of pro- and mesofemora and anterior 0.1–0.2 of tibiae); body length: 3.25 mm; fore wing length: 2.93 mm. Among species with T2 strongly sculptured but transverse, *Dolichogenideayungas* is similar to *D.alexandrei*, but it can be distinguished from it because of its much narrower T1 and T2, shorter ovipositor and ovipositor sheath, darker colored legs, infuscate wings and shorter vein R1 in fore wing.

##### Distribution.

Bolivia.

##### Biology.

No host data available.

##### DNA barcoding data.

No data.

##### Etymology.

Named after the area where the species has been collected; the Yungas forests, along the eastern slope of the Andes Mountains, are extremely diverse and represent a transitional zone between the Andean highlands and the eastern forests.

#### ﻿ Dolichogenidea yvesbraeti

Taxon classificationAnimaliaHymenopteraBraconidae

Fernandez-Triana & Boudreault sp. nov.

EE2204C4-CE79-5634-A968-F69449C4A1B2

https://zoobank.org/947DA4CC-DF28-463A-90BC-AB1DA80FB00B

Fig. 150A–F

##### Type material.

***Holotype*.** French Guiana • Female, CNC; Montagne de Kaw, Relais Patawa, Pk37,5; 4°32'42"N, 52°09'09"W; iii.2001; A.E.I. Guyane-J. Cerda leg.; Malaise Trap; Voucher code: CNC492795. ***Paratypes*.** French Guiana • 1 Female, CNC; voucher code: CNC492720.

**Figure 150.**
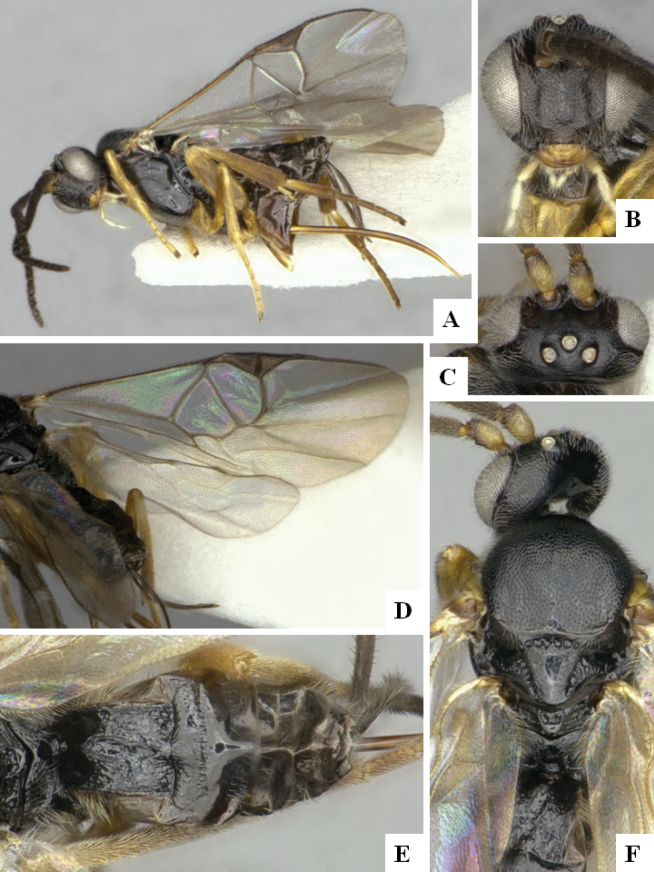
*Dolichogenideayvesbraeti* Fernandez-Triana & Boudreault holotype female CNC492720 **A** habitus, lateral **B** head, frontal **C** head, dorsal **D** wings **E** metasoma, dorsal **F** mesosoma, dorsal.

##### Diagnostic description.

F15 comparatively very long, 2.0× as long as its width; fore wing vein r longer than pterostigma height and ~ 2.0× as long as vein 2RS; T1 parallel-sided, < 3.0× its width at posterior margin; T1 mostly sculptured; T2 smooth and transverse; ovipositor comparatively thicker, as wide or wider than flagellomeres width; pterostigma mostly brown but with pale spot at anterior 0.2; all legs yellow or orange yellow, except for metacoxa mostly dark brown to black but with posterior 0.2 yellow; body length: 2.94–3.28 mm; fore wing length: 3.41–3.44 mm. Among all species with T1 sculptured but T2 smooth, this species can be recognized by the legs being entirely yellow to orange yellow, pterostigma color, long F15 and comparatively thick ovipositor.

##### Distribution.

French Guiana.

##### Biology.

No host data available.

##### DNA barcoding data.

No data.

##### Etymology.

Named after Yves Braet, as recognition to his contributions to the study of the French Guiana fauna of braconid parasitoid wasps.

**Figure 151.**
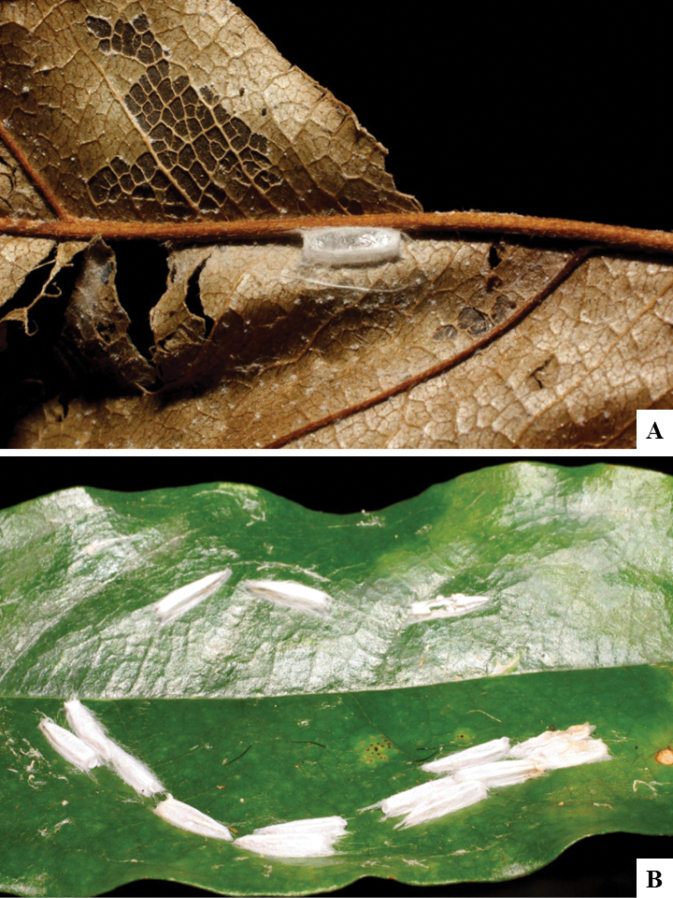
Parasite Cocoons of **A***Dolichogenideaalanflemingi* Janzen118 13-SRNP-67921-DHJ804735 **B***Dolichogenideaalejandromasisi* 06-SRNP-30366-DHJ409892.

**Figure 152.**
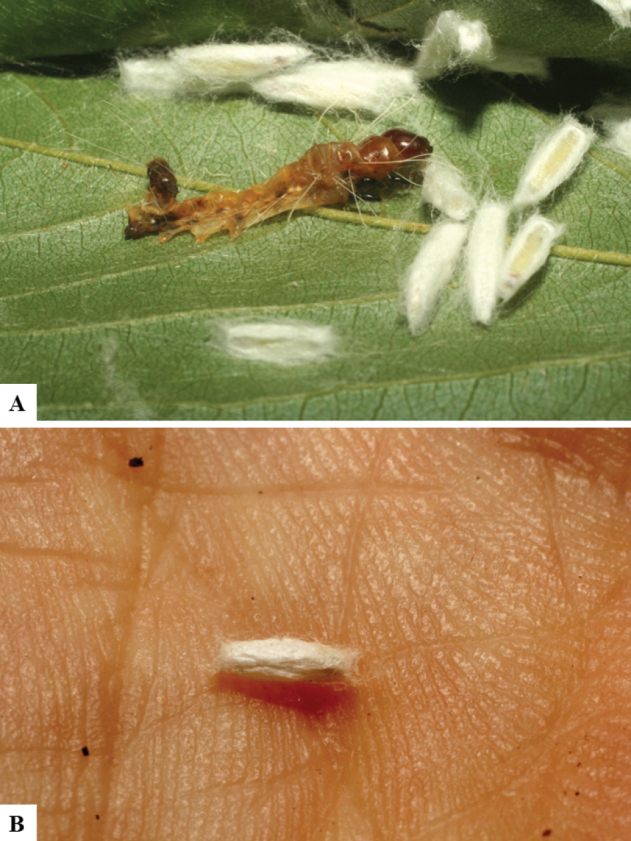
Parasite Cocoons of **A***Dolichogenideaalejandromasisi* 14-SRNP-65108-DHJ488109 **B***Dolichogenideaalexamasisae* Janzen257 13-SRNP-67147-DHJ804727.

**Figure 153.**
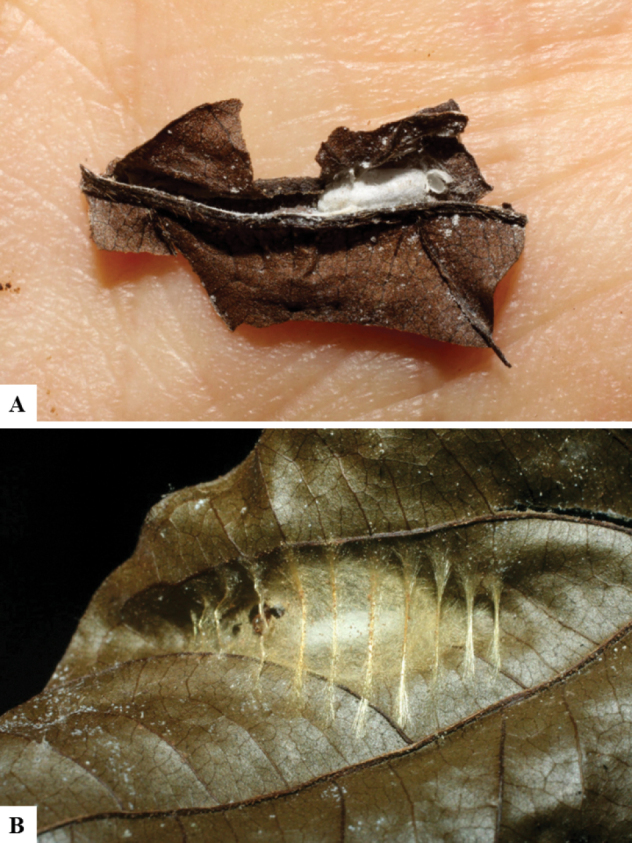
Parasite Cocoons of **A***Dolichogenideaanacamposae* Janzen55 09-SRNP-30898-DHJ474025 **B***Dolichogenideaangelagonzalezae* Janzen52 07-SRNP-42383-DHJ452922.

**Figure 154.**
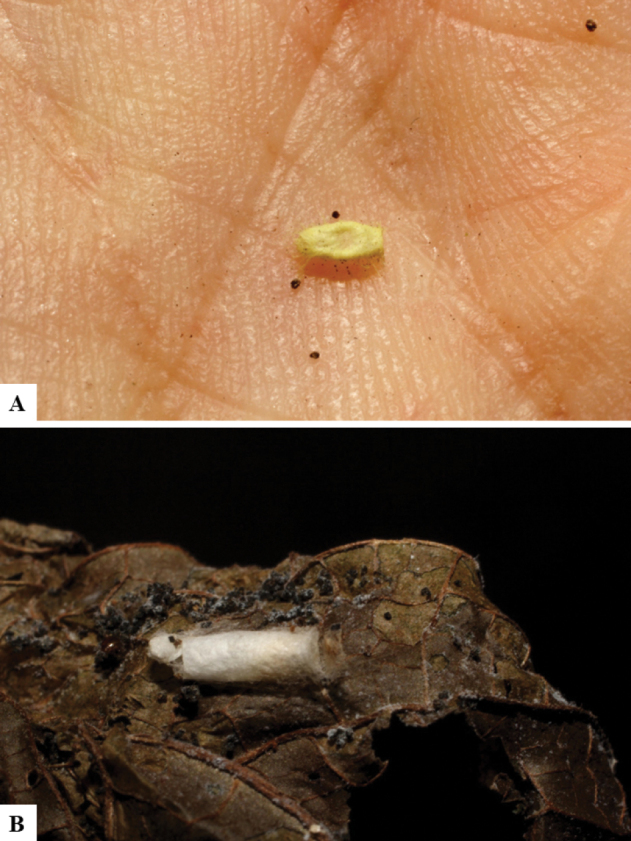
Parasite Cocoons of **A***Dolichogenideaangelsolisi* Janzen62 13-SRNP-70810-DHJ804622 **B***Dolichogenideaanikenpalolae* Whitfield80 10-SRNP-2762-DHJ496086.

**Figure 155.**
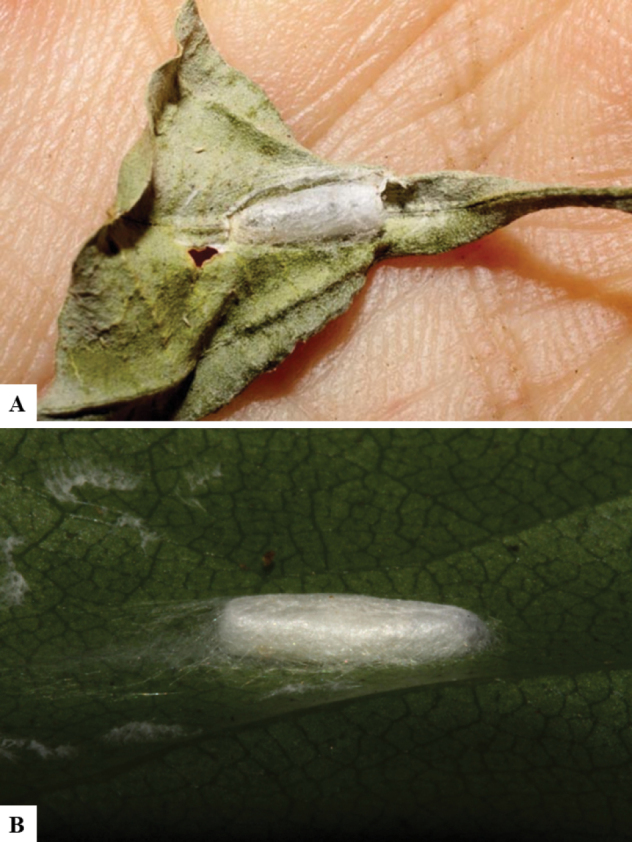
Parasite Cocoons of **A***Dolichogenideaanniapicadoae* Janzen25 09-SRNP-35297-DHJ470081 **B***Dolichogenideaannlisterudae* Janzen272 13-SRNP-31297-DHJ701517.

**Figure 156.**
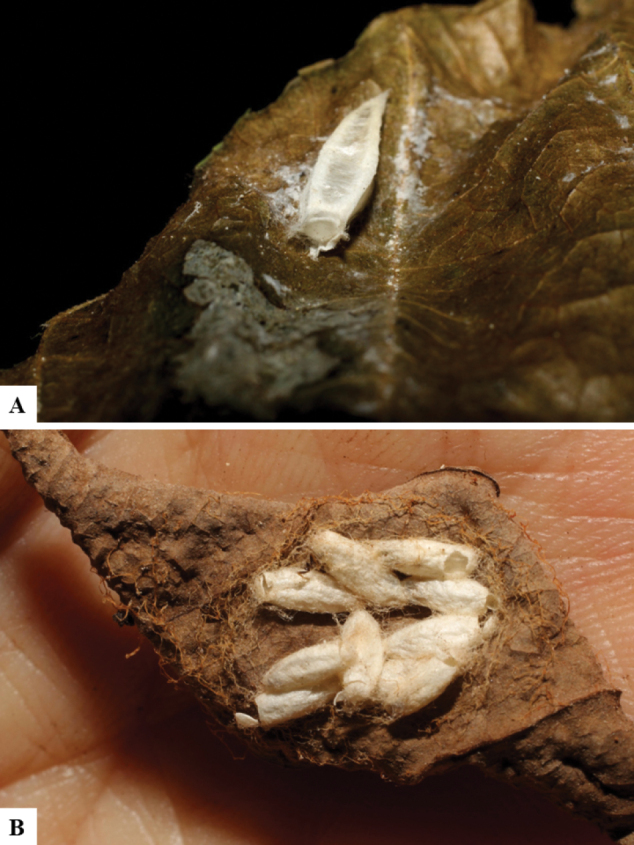
Parasite Cocoons of **A***Dolichogenideacarlosmanuelrodriguezi* Janzen245 09-SRNP-44335-DHJ474117 **B***Dolichogenideafredhicksi* Janzen90 08-SRNP-24699-DHJ495850.

**Figure 157.**
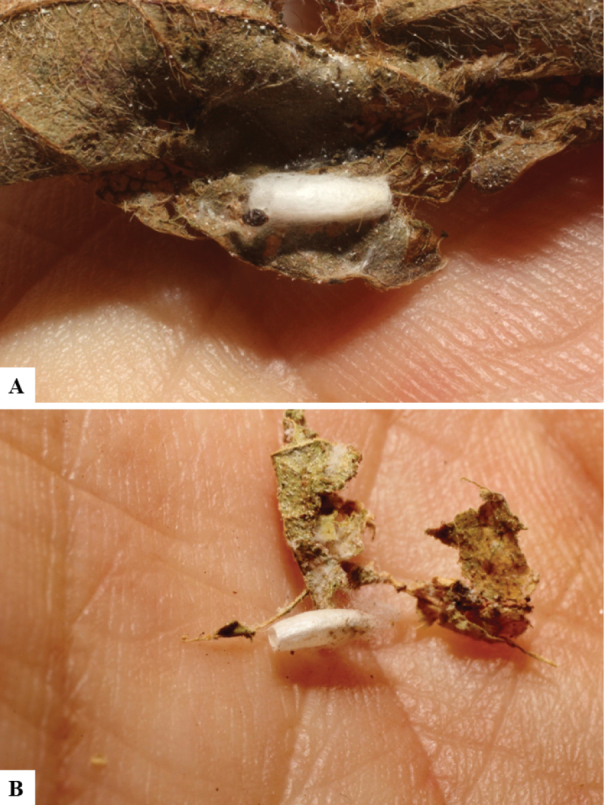
Parasite Cocoons of **A***Dolichogenideakenzabaddouae* Janzen116 13-SRNP-381-DHJ804665 **B***Dolichogenidealuzmariaromeroae* Janzen128 11-SRNP-42637-DHJ800828.

**Figure 158.**
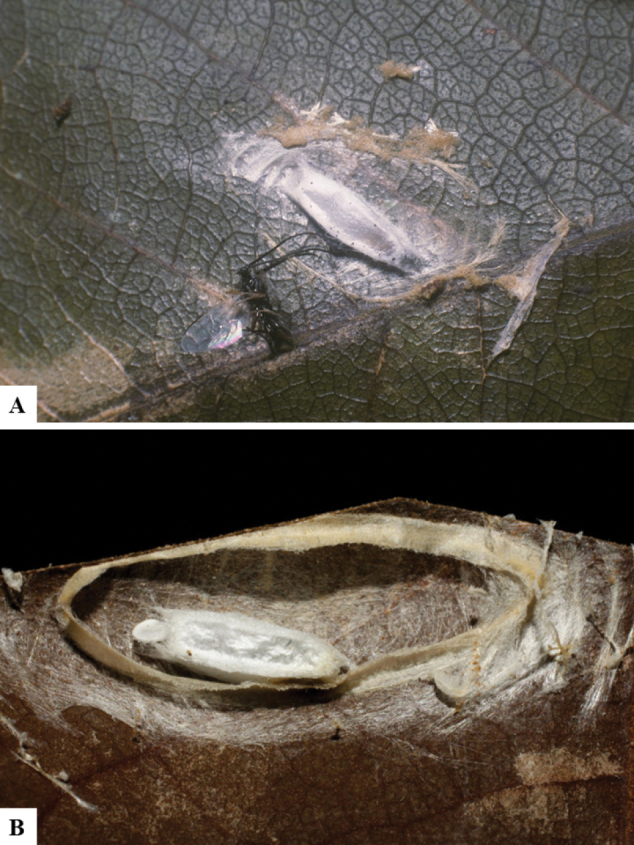
Parasite Cocoons of **A***Dolichogenideamelaniamunozae* Janzen35 04-SRNP-41854-DHJ87029 **B***Dolichogenideamelaniamunozae* Janzen35 09-SRNP-40585-DHJ474094.

**Figure 159.**
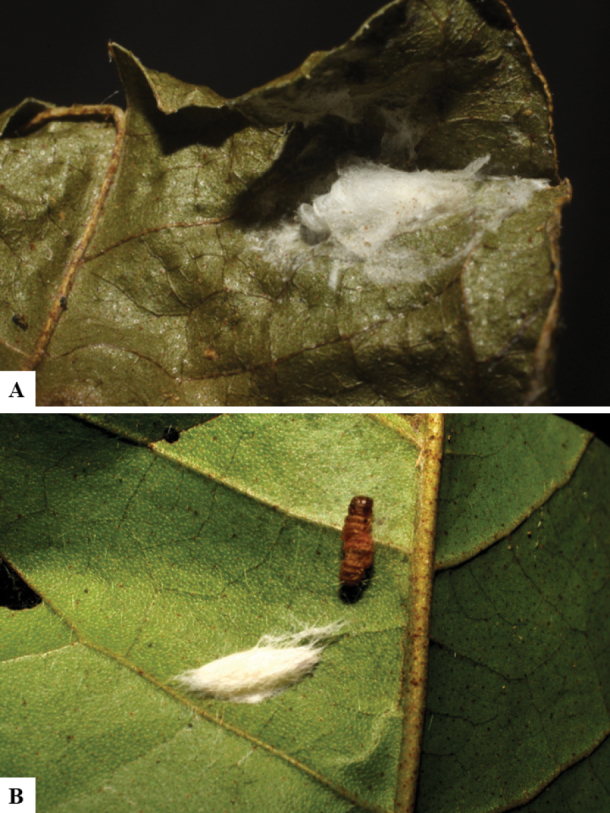
Parasite Cocoons of **A***Dolichogenideaninamasisae* Janzen120 10-SRNP-5690-DHJ480345 **B***Dolichogenideaninamasisae* Rodriguez157 07-SRNP-41992-DHJ427675.

**Figure 160.**
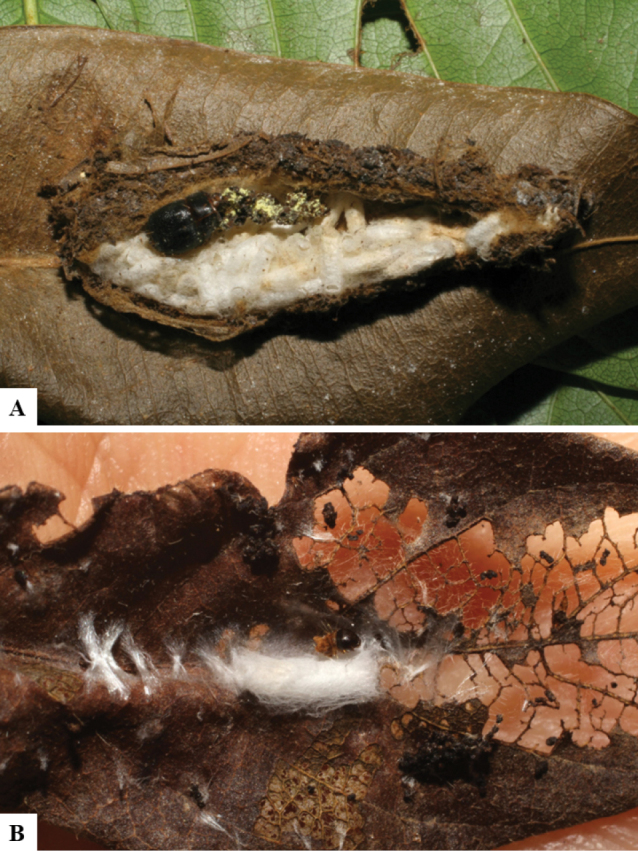
Parasite Cocoons of **A***Dolichogenideapedroleoni* Janzen09 04-SRNP-31206-DHJ401496 **B***Dolichogenidearobinsherwoodae* Janzen30 10-SRNP-2088-DHJ496019.

**Figure 161.**
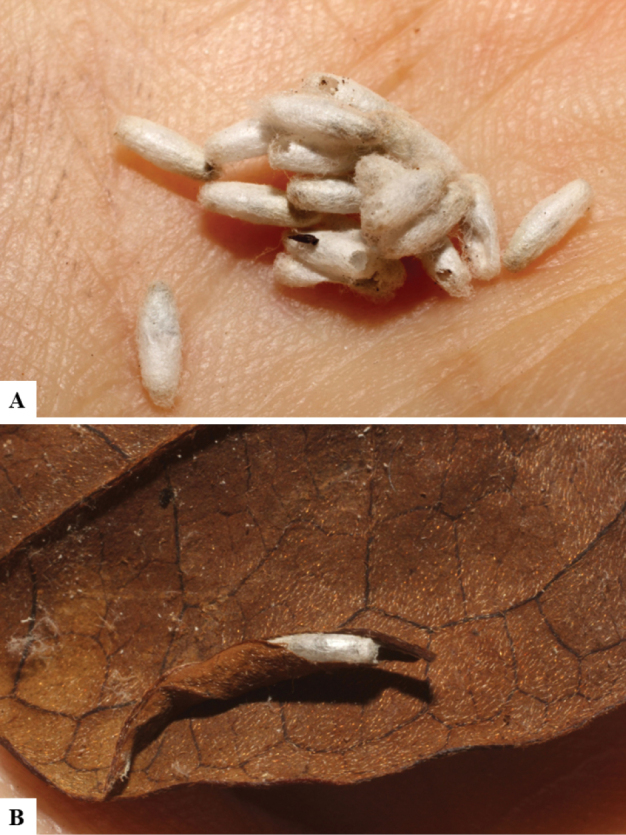
Parasite Cocoons of **A***Dolichogenidearobpringlei* Janzen07 10-SRNP-1795-DHJ496013 **B***Dolichogenidearociocordobae* Janzen10 14-SRNP-40704-DHJ804851.

**Figure 162.**
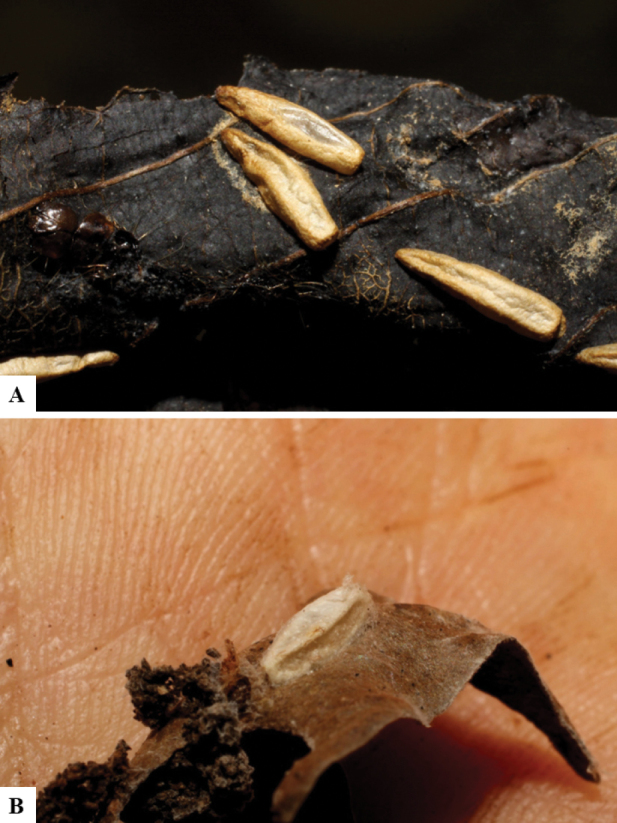
Parasite Cocoons of **A***Dolichogenidearogerblancoi* Janzen33 09-SRNP-32079-DHJ470027 **B***Dolichogenidearonaldzunigai* Janzen36 09-SRNP-21357-DHJ495863.

**Figure 163.**
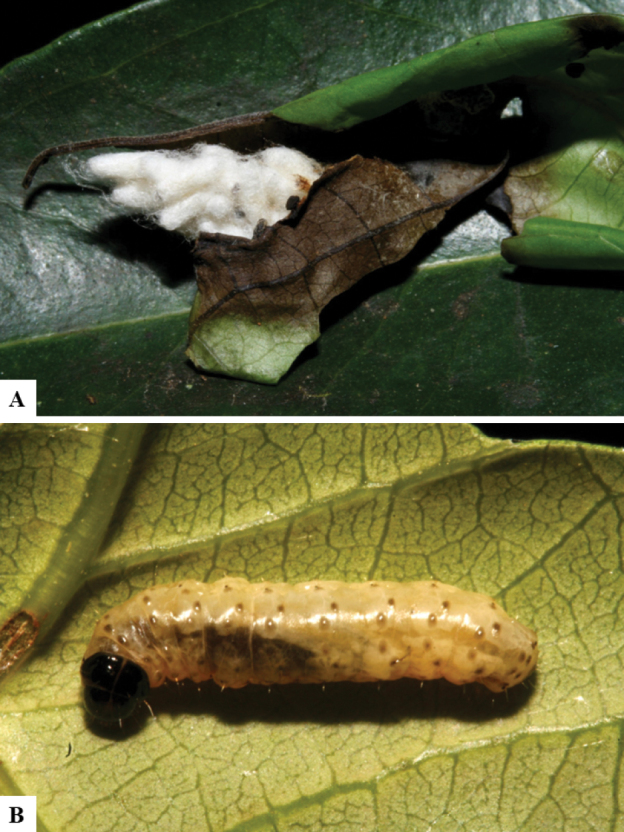
**A** Parasite Cocoons of *Dolichogenideasarahoconnorae* Janzen11 10-SRNP-40675-DHJ472743 **B** Caterpillar Parasitized by *Dolichogenideasarahoconnorae* 11-SRNP-41694-DHJ483080.

**Figure 164.**
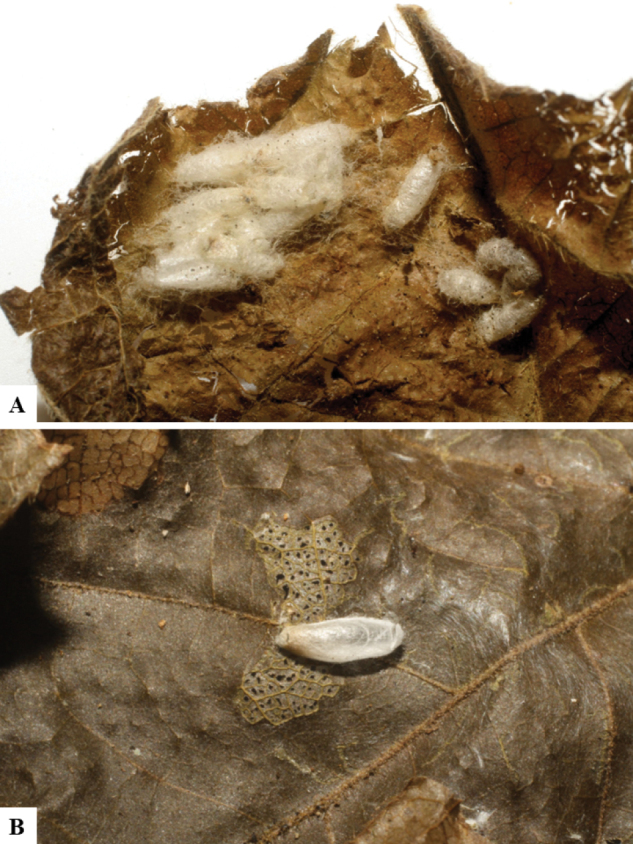
Parasite Cocoons of **A***Dolichogenideascottmilleri* Janzen02 04-SRNP-31333-DHJ415298 **B***Dolichogenideatiboshartae* Janzen49 10-SRNP-6273-DHJ480415.

**Figure 165.**
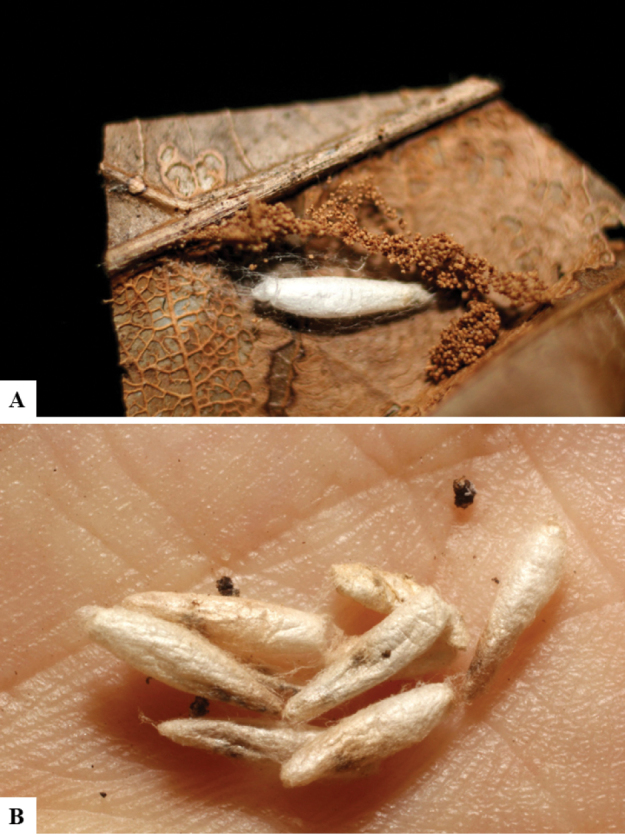
Parasite Cocoons of **A***Dolichogenideatomdaleyi* Janzen14 06-SRNP-31849-DHJ436533 **B***Dolichogenideacedenoae* 13-SRNP-31589-DHJ804613.

## ﻿﻿Additional information

### Author ORCIDs

Jose L. Fernandez-Triana https://orcid.org/0000-0003-0425-0309

Caroline Boudreault https://orcid.org/0000-0002-4511-2626

James B. Whitfield https://orcid.org/0000-0002-3031-9106

Amelie Höcherl https://orcid.org/0009-0007-4211-7468

M. Alex Smith https://orcid.org/0000-0002-8650-2575

Daniel H. Janzen https://orcid.org/0000-0002-7335-5107

### Data availability

All of the data that support the findings of this study are available in the main text or Supplementary Information.

## Supplementary materials

﻿Supplementary material 1 NJ tree all available sequences Data type pdf https://binary.pensoft.net/file/1321071This dataset is made available under the Open Database License (http://opendatacommons.org/licenses/odbl/1.0/). The Open Database License (ODbL) is a license agreement intended to allow users to freely share, modify, and use this Dataset while maintaining this same freedom for others, provided that the original source and author(s) are credited.Jose L. Fernandez-Triana, Caroline Boudreault, James B. Whitfield, Amelie Höcherl, M. Alex Smith, Winnifred Hallwachs, Daniel H. Janzen

﻿Supplementary material 2 Bayesian tree all sequences no collapsed tree Data type pdf https://binary.pensoft.net/file/1321072This dataset is made available under the Open Database License (http://opendatacommons.org/licenses/odbl/1.0/). The Open Database License (ODbL) is a license agreement intended to allow users to freely share, modify, and use this Dataset while maintaining this same freedom for others, provided that the original source and author(s) are credited.Jose L. Fernandez-Triana, Caroline Boudreault, James B. Whitfield, Amelie Höcherl, M. Alex Smith, Winnifred Hallwachs, Daniel H. Janzen

﻿Supplementary material 3 All sequences as fasta files Data type fas https://binary.pensoft.net/file/1321073This dataset is made available under the Open Database License (http://opendatacommons.org/licenses/odbl/1.0/). The Open Database License (ODbL) is a license agreement intended to allow users to freely share, modify, and use this Dataset while maintaining this same freedom for others, provided that the original source and author(s) are credited.Jose L. Fernandez-Triana, Caroline Boudreault, James B. Whitfield, Amelie Höcherl, M. Alex Smith, Winnifred Hallwachs, Daniel H. Janzen

﻿Supplementary material 4 BEAT settings Data type xml https://binary.pensoft.net/file/1321074This dataset is made available under the Open Database License (http://opendatacommons.org/licenses/odbl/1.0/). The Open Database License (ODbL) is a license agreement intended to allow users to freely share, modify, and use this Dataset while maintaining this same freedom for others, provided that the original source and author(s) are credited.Jose L. Fernandez-Triana, Caroline Boudreault, James B. Whitfield, Amelie Höcherl, M. Alex Smith, Winnifred Hallwachs, Daniel H. Janzen

﻿Supplementary material 5 Additional figures Data type pdf https://binary.pensoft.net/file/1321075This dataset is made available under the Open Database License (http://opendatacommons.org/licenses/odbl/1.0/). The Open Database License (ODbL) is a license agreement intended to allow users to freely share, modify, and use this Dataset while maintaining this same freedom for others, provided that the original source and author(s) are credited.Jose L. Fernandez-Triana, Caroline Boudreault, James B. Whitfield, Amelie Höcherl, M. Alex Smith, Winnifred Hallwachs, Daniel H. Janzen

﻿Supplementary material 6 Additional information Data type fas https://binary.pensoft.net/file/1321076This dataset is made available under the Open Database License (http://opendatacommons.org/licenses/odbl/1.0/). The Open Database License (ODbL) is a license agreement intended to allow users to freely share, modify, and use this Dataset while maintaining this same freedom for others, provided that the original source and author(s) are credited.Jose L. Fernandez-Triana, Caroline Boudreault, James B. Whitfield, Amelie Höcherl, M. Alex Smith, Winnifred Hallwachs, Daniel H. Janzen
