# Supplementary material for: ﻿A revision of the parasitoid wasp genus Dolichogenidea Viereck (Hymenoptera, Braconidae) in the Neotropical region, with the description of 102 new species
Source: Zookeys. 2025 May 7;1237:1–250. doi: 10.3897/zookeys.1237.141007 (PMC12079117; doi:10.3897/zookeys.1237.141007)
Supplement: ﻿Supplementary material 1 — NJ tree all available sequences [file zookeys-1237-001_article-141007__-s001.pdf]

BOLD TaxonID Tree

Title : Tree Result - Search: Sample IDs; Include public records (1962 records returned) (1962 records selected)

Date : 08-Jan-2024

Data Type : Nucleotide

Distance Model : Kimura 2 Parameter

Marker : COI-5P

Colourization : [blue]=Stop Codons [red]=Contamination or misidentification

Label : Sample ID

Label : Taxon

Label : Country

Label : Sequence Length

Label : Barcode Cluster (BIN)

Filter : length > 100bp only

Filter : exclude records flagged as misidentifications

Filter : exclude records with stop codons

Filter : exclude contaminants

Sequence Count : 1962

Species count : 100

Genus count : 4

Family count : 1

Unidentified : 64

BIN Count : 84

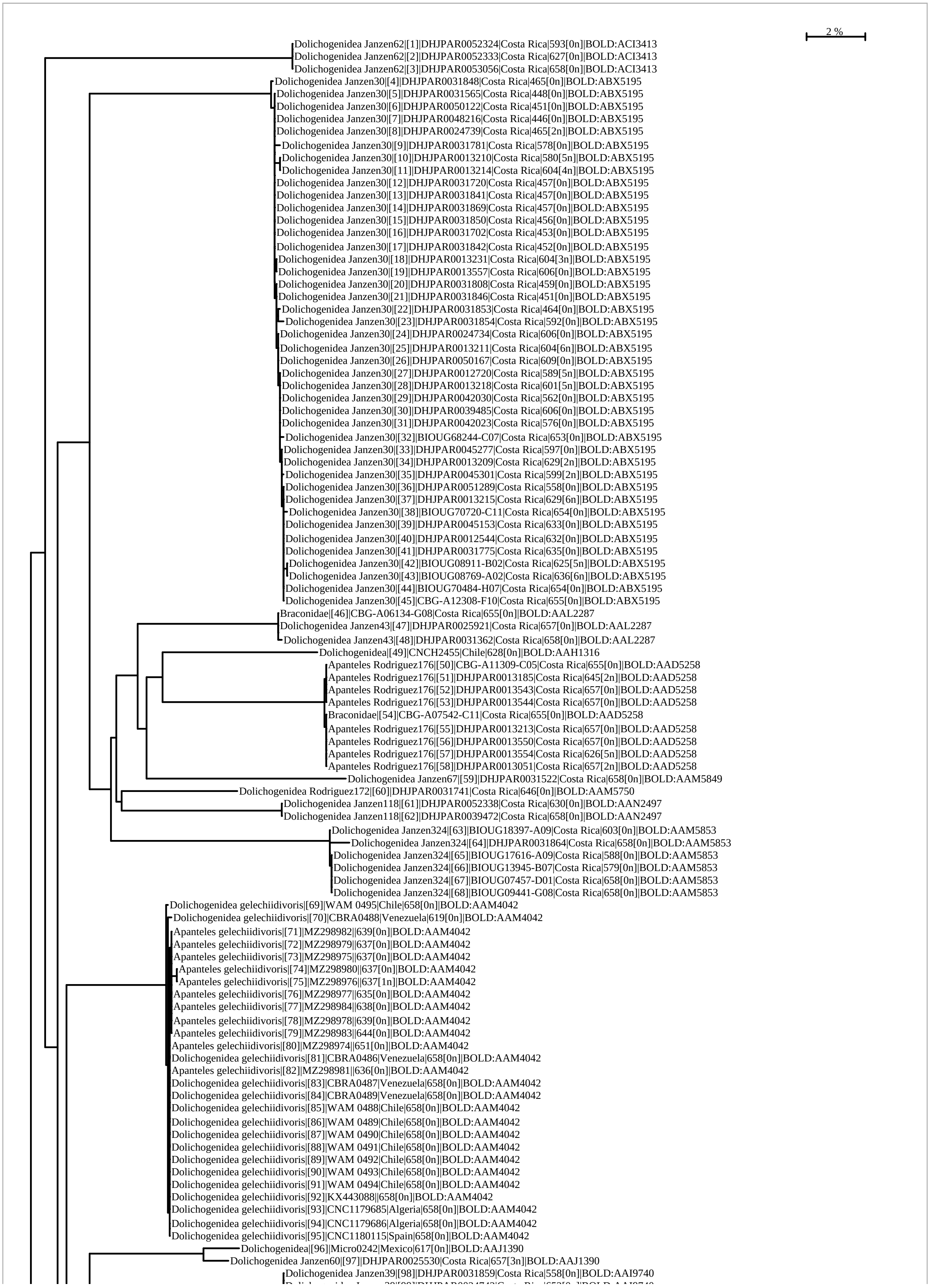
2 %
Dolichogenidea Janzen62|[1]|DHJPAR0052324|Costa Rica|593[0n]|BOLD:ACI3413
Dolichogenidea Janzen62|[2]|DHJPAR0052333|Costa Rica|627[0n]|BOLD:ACI3413
Dolichogenidea Janzen62|[3]|DHJPAR0053056|Costa Rica|658[0n]|BOLD:ACI3413
Dolichogenidea Janzen30|[4]|DHJPAR0031848|Costa Rica|465[0n]|BOLD:ABX5195
Dolichogenidea Janzen30|[5]|DHJPAR0031565|Costa Rica|448[0n]|BOLD:ABX5195
Dolichogenidea Janzen30|[6]|DHJPAR0050122|Costa Rica|451[0n]|BOLD:ABX5195
Dolichogenidea Janzen30|[7]|DHJPAR0048216|Costa Rica|446[0n]|BOLD:ABX5195
Dolichogenidea Janzen30|[8]|DHJPAR0024739|Costa Rica|465[2n]|BOLD:ABX5195
Dolichogenidea Janzen30|[9]|DHJPAR0031781|Costa Rica|578[0n]|BOLD:ABX5195
Dolichogenidea Janzen30|[10]|DHJPAR0013210|Costa Rica|580[5n]|BOLD:ABX5195
Dolichogenidea Janzen30|[11]|DHJPAR0013214|Costa Rica|604[4n]|BOLD:ABX5195
Dolichogenidea Janzen30|[12]|DHJPAR0031720|Costa Rica|457[0n]|BOLD:ABX5195
Dolichogenidea Janzen30|[13]|DHJPAR0031841|Costa Rica|457[0n]|BOLD:ABX5195
Dolichogenidea Janzen30|[14]|DHJPAR0031869|Costa Rica|457[0n]|BOLD:ABX5195
Dolichogenidea Janzen30|[15]|DHJPAR0031850|Costa Rica|456[0n]|BOLD:ABX5195
Dolichogenidea Janzen30|[16]|DHJPAR0031702|Costa Rica|453[0n]|BOLD:ABX5195
Dolichogenidea Janzen30|[17]|DHJPAR0031842|Costa Rica|452[0n]|BOLD:ABX5195
Dolichogenidea Janzen30|[18]|DHJPAR0013231|Costa Rica|604[3n]|BOLD:ABX5195
Dolichogenidea Janzen30|[19]|DHJPAR0013557|Costa Rica|606[0n]|BOLD:ABX5195
Dolichogenidea Janzen30|[20]|DHJPAR0031808|Costa Rica|459[0n]|BOLD:ABX5195
Dolichogenidea Janzen30|[21]|DHJPAR0031846|Costa Rica|451[0n]|BOLD:ABX5195
Dolichogenidea Janzen30|[22]|DHJPAR0031853|Costa Rica|464[0n]|BOLD:ABX5195
Dolichogenidea Janzen30|[23]|DHJPAR0031854|Costa Rica|592[0n]|BOLD:ABX5195
Dolichogenidea Janzen30|[24]|DHJPAR0024734|Costa Rica|606[0n]|BOLD:ABX5195
Dolichogenidea Janzen30|[25]|DHJPAR0013211|Costa Rica|604[6n]|BOLD:ABX5195
Dolichogenidea Janzen30|[26]|DHJPAR0050167|Costa Rica|609[0n]|BOLD:ABX5195
Dolichogenidea Janzen30|[27]|DHJPAR0012720|Costa Rica|589[5n]|BOLD:ABX5195
Dolichogenidea Janzen30|[28]|DHJPAR0013218|Costa Rica|601[5n]|BOLD:ABX5195
Dolichogenidea Janzen30|[29]|DHJPAR0042030|Costa Rica|562[0n]|BOLD:ABX5195
Dolichogenidea Janzen30|[30]|DHJPAR0039485|Costa Rica|606[0n]|BOLD:ABX5195
Dolichogenidea Janzen30|[31]|DHJPAR0042023|Costa Rica|576[0n]|BOLD:ABX5195
Dolichogenidea Janzen30|[32]|BIOUG68244-C07|Costa Rica|653[0n]|BOLD:ABX5195
Dolichogenidea Janzen30|[33]|DHJPAR0045277|Costa Rica|597[0n]|BOLD:ABX5195
Dolichogenidea Janzen30|[34]|DHJPAR0013209|Costa Rica|629[2n]|BOLD:ABX5195
Dolichogenidea Janzen30|[35]|DHJPAR0045301|Costa Rica|599[2n]|BOLD:ABX5195
Dolichogenidea Janzen30|[36]|DHJPAR0051289|Costa Rica|558[0n]|BOLD:ABX5195
Dolichogenidea Janzen30|[37]|DHJPAR0013215|Costa Rica|629[6n]|BOLD:ABX5195
Dolichogenidea Janzen30|[38]|BIOUG70720-C11|Costa Rica|654[0n]|BOLD:ABX5195
Dolichogenidea Janzen30|[39]|DHJPAR0045153|Costa Rica|633[0n]|BOLD:ABX5195
Dolichogenidea Janzen30|[40]|DHJPAR0012544|Costa Rica|632[0n]|BOLD:ABX5195
Dolichogenidea Janzen30|[41]|DHJPAR0031775|Costa Rica|635[0n]|BOLD:ABX5195
Dolichogenidea Janzen30|[42]|BIOUG08911-B02|Costa Rica|625[5n]|BOLD:ABX5195
Dolichogenidea Janzen30|[43]|BIOUG08769-A02|Costa Rica|636[6n]|BOLD:ABX5195
Dolichogenidea Janzen30|[44]|BIOUG70484-H07|Costa Rica|654[0n]|BOLD:ABX5195
Dolichogenidea Janzen30|[45]|CBG-A12308-F10|Costa Rica|655[0n]|BOLD:ABX5195
Braconidae|[46]|CBG-A06134-G08|Costa Rica|655[0n]|BOLD:AAL2287
Dolichogenidea Janzen43|[47]|DHJPAR0025921|Costa Rica|657[0n]|BOLD:AAL2287
Dolichogenidea Janzen43|[48]|DHJPAR0031362|Costa Rica|658[0n]|BOLD:AAL2287
Dolichogenidea|[49]|CNCH2455|Chile|628[0n]|BOLD:AAH1316
Apanteles Rodriguez176|[50]|CBG-A11309-C05|Costa Rica|655[0n]|BOLD:AAD5258
Apanteles Rodriguez176|[51]|DHJPAR0013185|Costa Rica|645[2n]|BOLD:AAD5258
Apanteles Rodriguez176|[52]|DHJPAR0013543|Costa Rica|657[0n]|BOLD:AAD5258
Apanteles Rodriguez176|[53]|DHJPAR0013544|Costa Rica|657[0n]|BOLD:AAD5258
Braconidae|[54]|CBG-A07542-C11|Costa Rica|655[0n]|BOLD:AAD5258
Apanteles Rodriguez176|[55]|DHJPAR0013213|Costa Rica|657[0n]|BOLD:AAD5258
Apanteles Rodriguez176|[56]|DHJPAR0013550|Costa Rica|657[0n]|BOLD:AAD5258
Apanteles Rodriguez176|[57]|DHJPAR0013554|Costa Rica|626[5n]|BOLD:AAD5258
Apanteles Rodriguez176|[58]|DHJPAR0013051|Costa Rica|657[2n]|BOLD:AAD5258
Dolichogenidea Janzen67|[59]|DHJPAR0031522|Costa Rica|658[0n]|BOLD:AAM5849
Dolichogenidea Rodriguez172|[60]|DHJPAR0031741|Costa Rica|646[0n]|BOLD:AAM5750
Dolichogenidea Janzen118|[61]|DHJPAR0052338|Costa Rica|630[0n]|BOLD:AAN2497
Dolichogenidea Janzen118|[62]|DHJPAR0039472|Costa Rica|658[0n]|BOLD:AAN2497
Dolichogenidea Janzen324|[63]|BIOUG18397-A09|Costa Rica|603[0n]|BOLD:AAM5853
Dolichogenidea Janzen324|[64]|DHJPAR0031864|Costa Rica|658[0n]|BOLD:AAM5853
Dolichogenidea Janzen324|[65]|BIOUG17616-A09|Costa Rica|588[0n]|BOLD:AAM5853
Dolichogenidea Janzen324|[66]|BIOUG13945-B07|Costa Rica|579[0n]|BOLD:AAM5853
Dolichogenidea Janzen324|[67]|BIOUG07457-D01|Costa Rica|658[0n]|BOLD:AAM5853
Dolichogenidea Janzen324|[68]|BIOUG09441-G08|Costa Rica|658[0n]|BOLD:AAM5853
Dolichogenidea gelechiidivoris|[69]|WAM 0495|Chile|658[0n]|BOLD:AAM4042
Dolichogenidea gelechiidivoris|[70]|CBRA0488|Venezuela|619[0n]|BOLD:AAM4042
Apanteles gelechiidivoris|[71]|MZ298982||639[0n]|BOLD:AAM4042
Apanteles gelechiidivoris|[72]|MZ298979||637[0n]|BOLD:AAM4042
Apanteles gelechiidivoris|[73]|MZ298975||637[0n]|BOLD:AAM4042
Apanteles gelechiidivoris|[74]|MZ298980||637[0n]|BOLD:AAM4042
Apanteles gelechiidivoris|[75]|MZ298976||637[1n]|BOLD:AAM4042
Apanteles gelechiidivoris|[76]|MZ298977||635[0n]|BOLD:AAM4042
Apanteles gelechiidivoris|[77]|MZ298984||638[0n]|BOLD:AAM4042
Apanteles gelechiidivoris|[78]|MZ298978||639[0n]|BOLD:AAM4042
Apanteles gelechiidivoris|[79]|MZ298983||644[0n]|BOLD:AAM4042
Apanteles gelechiidivoris|[80]|MZ298974||651[0n]|BOLD:AAM4042
Dolichogenidea gelechiidivoris|[81]|CBRA0486|Venezuela|658[0n]|BOLD:AAM4042
Apanteles gelechiidivoris|[82]|MZ298981||636[0n]|BOLD:AAM4042
Dolichogenidea gelechiidivoris|[83]|CBRA0487|Venezuela|658[0n]|BOLD:AAM4042
Dolichogenidea gelechiidivoris|[84]|CBRA0489|Venezuela|658[0n]|BOLD:AAM4042
Dolichogenidea gelechiidivoris|[85]|WAM 0488|Chile|658[0n]|BOLD:AAM4042
Dolichogenidea gelechiidivoris|[86]|WAM 0489|Chile|658[0n]|BOLD:AAM4042
Dolichogenidea gelechiidivoris|[87]|WAM 0490|Chile|658[0n]|BOLD:AAM4042
Dolichogenidea gelechiidivoris|[88]|WAM 0491|Chile|658[0n]|BOLD:AAM4042
Dolichogenidea gelechiidivoris|[89]|WAM 0492|Chile|658[0n]|BOLD:AAM4042
Dolichogenidea gelechiidivoris|[90]|WAM 0493|Chile|658[0n]|BOLD:AAM4042
Dolichogenidea gelechiidivoris|[91]|WAM 0494|Chile|658[0n]|BOLD:AAM4042
Dolichogenidea gelechiidivoris|[92]|KX443088||658[0n]|BOLD:AAM4042
Dolichogenidea gelechiidivoris|[93]|CNC1179685|Algeria|658[0n]|BOLD:AAM4042
Dolichogenidea gelechiidivoris|[94]|CNC1179686|Algeria|658[0n]|BOLD:AAM4042
Dolichogenidea gelechiidivoris|[95]|CNC1180115|Spain|658[0n]|BOLD:AAM4042
Dolichogenidea|[96]|Micro0242|Mexico|617[0n]|BOLD:AAJ1390
Dolichogenidea Janzen60|[97]|DHJPAR0025530|Costa Rica|657[3n]|BOLD:AAJ1390
Dolichogenidea Janzen39|[98]|DHJPAR0031859|Costa Rica|558[0n]|BOLD:AAI9740

Dolichogenidea|[96]|Micro0242|Mexico|617[0n]|BOLD:AAJ1390
Dolichogenidea Janzen60|[97]|DHJPAR0025530|Costa Rica|657[3n]|BOLD:AAJ1390
Dolichogenidea Janzen39|[98]|DHJPAR0031859|Costa Rica|558[0n]|BOLD:AAI9740
Dolichogenidea Janzen39|[99]|DHJPAR0024742|Costa Rica|652[0n]|BOLD:AAI9740
Dolichogenidea Janzen39|[100]|DHJPAR0024710|Costa Rica|657[0n]|BOLD:AAI9740
Dolichogenidea Janzen39|[101]|DHJPAR0031860|Costa Rica|658[0n]|BOLD:AAI9740
Dolichogenidea Janzen39|[102]|DHJPAR0031872|Costa Rica|658[0n]|BOLD:AAI9740
Dolichogenidea Whitfield79|[103]|DHJPAR0031845|Costa Rica|658[0n]|BOLD:AAM5850
Dolichogenidea Whitfield80|[104]|DHJPAR0040468|Costa Rica|627[0n]|BOLD:ABY1812
Dolichogenidea Whitfield80|[105]|DHJPAR0040482|Costa Rica|627[0n]|BOLD:ABY1812
Dolichogenidea Whitfield80|[106]|DHJPAR0040479|Costa Rica|365[0n]|BOLD:ABY1812
Dolichogenidea Whitfield80|[107]|DHJPAR0049280|Costa Rica|658[0n]|BOLD:ABY1812
Dolichogenidea Whitfield80|[108]|DHJPAR0049330|Costa Rica|658[0n]|BOLD:ABY1812
Dolichogenidea Whitfield80|[109]|DHJPAR0049340|Costa Rica|658[0n]|BOLD:ABY1812
Dolichogenidea|[110]|CCDB-07375 B04|French Guiana|658[0n]|BOLD:ABZ4155
Dolichogenidea angelagonzalezae|[111]|DHJPAR0038168|Costa Rica|421[0n]|BOLD:AAL2298
Dolichogenidea angelagonzalezae|[112]|DHJPAR0020708|Costa Rica|612[0n]|BOLD:AAL2298
Dolichogenidea angelagonzalezae|[113]|DHJPAR0020711|Costa Rica|620[0n]|BOLD:AAL2298
Dolichogenidea Janzen156|[114]|DHJPAR0041976|Costa Rica|658[0n]|BOLD:ABZ4155
Dolichogenidea Janzen156|[115]|DHJPAR0042003|Costa Rica|658[0n]|BOLD:ABZ4155
Dolichogenidea Janzen259|[116]|DHJPAR0048195|Costa Rica|658[0n]|BOLD:ABZ4155
Dolichogenidea Janzen51|[117]|DHJPAR0039019|Costa Rica|658[0n]|BOLD:ABZ4155
Dolichogenidea Janzen51|[118]|DHJPAR0039025|Costa Rica|658[0n]|BOLD:ABZ4155
Dolichogenidea Janzen51|[119]|DHJPAR0039461|Costa Rica|658[0n]|BOLD:ABZ4155
Dolichogenidea Janzen51|[120]|DHJPAR0039468|Costa Rica|658[0n]|BOLD:ABZ4155
Dolichogenidea Janzen51|[121]|DHJPAR0043028|Costa Rica|658[0n]|BOLD:ABZ4155
Dolichogenidea Janzen51|[122]|DHJPAR0043060|Costa Rica|658[0n]|BOLD:ABZ4155
Dolichogenidea Janzen325|[123]|DHJPAR0038235|Costa Rica|658[0n]|BOLD:ABZ4155
Dolichogenidea carlosmanuelrodriguezi|[124]|DHJPAR0039056|Costa Rica|632[0n]|BOLD:ABZ4155
Dolichogenidea carlosmanuelrodriguezi|[125]|DHJPAR0039047|Costa Rica|633[0n]|BOLD:ABZ4155
Dolichogenidea carlosmanuelrodriguezi|[126]|DHJPAR0035494|Costa Rica|658[0n]|BOLD:ABZ4155
Dolichogenidea carlosmanuelrodriguezi|[127]|DHJPAR0049471|Costa Rica|658[0n]|BOLD:ABZ4155
Dolichogenidea josealfredohernandezi|[128]|DHJPAR0045125|Costa Rica|658[0n]|BOLD:ABA9255
Dolichogenidea josealfredohernandezi|[129]|DHJPAR0056646|Costa Rica|658[0n]|BOLD:ABA9255
Dolichogenidea josealfredohernandezi|[130]|DHJPAR0039856|Costa Rica|407[0n]|BOLD:ABA9255
Dolichogenidea josealfredohernandezi|[131]|DHJPAR0048168|Costa Rica|658[0n]|BOLD:ABA9255
Dolichogenidea josealfredohernandezi|[132]|DHJPAR0049909|Costa Rica|658[0n]|BOLD:ABA9255
Dolichogenidea josealfredohernandezi|[133]|DHJPAR0053856|Costa Rica|661[0n]|BOLD:ABA9255
Dolichogenidea alejandromasisi|[134]|DHJPAR0042507|Costa Rica|623[0n]|BOLD:ABX6174
Dolichogenidea alejandromasisi|[135]|DHJPAR0055439|Costa Rica|618[0n]|BOLD:ABX6174
Dolichogenidea alejandromasisi|[136]|DHJPAR0004258|Costa Rica|627[0n]|BOLD:ABX6174
Dolichogenidea alejandromasisi|[137]|DHJPAR0003956|Costa Rica|576[0n]|BOLD:ABX6174
Dolichogenidea alejandromasisi|[138]|DHJPAR0004859|Costa Rica|630[5n]|BOLD:ABX6174
Dolichogenidea alejandromasisi|[139]|DHJPAR0004870|Costa Rica|600[3n]|BOLD:ABX6174
Arthropoda|[140]|21-SRNP-41439|Costa Rica|654[0n]|BOLD:ABX6174
Arthropoda|[141]|21-SRNP-41413|Costa Rica|653[0n]|BOLD:ABX6174
Dolichogenidea alejandromasisi|[142]|DHJPAR0045173|Costa Rica|658[1n]|BOLD:ABX6174
Dolichogenidea alejandromasisi|[143]|DHJPAR0035291|Costa Rica|658[0n]|BOLD:ABX6174
Dolichogenidea alejandromasisi|[144]|DHJPAR0035387|Costa Rica|658[0n]|BOLD:ABX6174
Dolichogenidea alejandromasisi|[145]|DHJPAR0035388|Costa Rica|658[0n]|BOLD:ABX6174
Dolichogenidea alejandromasisi|[146]|DHJPAR0035395|Costa Rica|658[0n]|BOLD:ABX6174
Dolichogenidea alejandromasisi|[147]|DHJPAR0035466|Costa Rica|658[0n]|BOLD:ABX6174
Dolichogenidea alejandromasisi|[148]|DHJPAR0035471|Costa Rica|658[0n]|BOLD:ABX6174
Dolichogenidea alejandromasisi|[149]|DHJPAR0038083|Costa Rica|658[0n]|BOLD:ABX6174
Dolichogenidea alejandromasisi|[150]|DHJPAR0038129|Costa Rica|658[0n]|BOLD:ABX6174
Dolichogenidea alejandromasisi|[151]|DHJPAR0061672|Costa Rica|658[0n]|BOLD:ABX6174
Dolichogenidea alejandromasisi|[152]|DHJPAR0060685|Costa Rica|658[0n]|BOLD:ABX6174
Dolichogenidea alejandromasisi|[153]|DHJPAR0060680|Costa Rica|658[0n]|BOLD:ABX6174
Dolichogenidea alejandromasisi|[154]|DHJPAR0060675|Costa Rica|658[0n]|BOLD:ABX6174
Dolichogenidea alejandromasisi|[155]|DHJPAR0060674|Costa Rica|658[0n]|BOLD:ABX6174
Dolichogenidea alejandromasisi|[156]|DHJPAR0058905|Costa Rica|658[0n]|BOLD:ABX6174
Dolichogenidea alejandromasisi|[157]|DHJPAR0058904|Costa Rica|658[0n]|BOLD:ABX6174
Dolichogenidea alejandromasisi|[158]|DHJPAR0058885|Costa Rica|658[0n]|BOLD:ABX6174
Dolichogenidea alejandromasisi|[159]|DHJPAR0058871|Costa Rica|658[0n]|BOLD:ABX6174
Dolichogenidea alejandromasisi|[160]|DHJPAR0058833|Costa Rica|658[0n]|BOLD:ABX6174
Dolichogenidea alejandromasisi|[161]|DHJPAR0055453|Costa Rica|658[0n]|BOLD:ABX6174
Dolichogenidea alejandromasisi|[162]|DHJPAR0055446|Costa Rica|658[0n]|BOLD:ABX6174
Dolichogenidea alejandromasisi|[163]|DHJPAR0055427|Costa Rica|658[0n]|BOLD:ABX6174
Dolichogenidea alejandromasisi|[164]|DHJPAR0055425|Costa Rica|658[0n]|BOLD:ABX6174
Dolichogenidea alejandromasisi|[165]|DHJPAR0055424|Costa Rica|658[0n]|BOLD:ABX6174
Dolichogenidea alejandromasisi|[166]|DHJPAR0055420|Costa Rica|658[0n]|BOLD:ABX6174
Dolichogenidea alejandromasisi|[167]|DHJPAR0055250|Costa Rica|658[0n]|BOLD:ABX6174
Dolichogenidea alejandromasisi|[168]|DHJPAR0054648|Costa Rica|658[0n]|BOLD:ABX6174
Dolichogenidea alejandromasisi|[169]|DHJPAR0054631|Costa Rica|658[0n]|BOLD:ABX6174
Dolichogenidea alejandromasisi|[170]|DHJPAR0052965|Costa Rica|658[0n]|BOLD:ABX6174
Dolichogenidea alejandromasisi|[171]|DHJPAR0052952|Costa Rica|658[0n]|BOLD:ABX6174
Dolichogenidea alejandromasisi|[172]|DHJPAR0052924|Costa Rica|658[0n]|BOLD:ABX6174
Dolichogenidea alejandromasisi|[173]|DHJPAR0051014|Costa Rica|658[0n]|BOLD:ABX6174
Dolichogenidea alejandromasisi|[174]|DHJPAR0049832|Costa Rica|658[0n]|BOLD:ABX6174
Dolichogenidea alejandromasisi|[175]|DHJPAR0049173|Costa Rica|658[0n]|BOLD:ABX6174
Dolichogenidea alejandromasisi|[176]|DHJPAR0049142|Costa Rica|658[0n]|BOLD:ABX6174
Dolichogenidea alejandromasisi|[177]|DHJPAR0049097|Costa Rica|658[0n]|BOLD:ABX6174
Dolichogenidea alejandromasisi|[178]|DHJPAR0049092|Costa Rica|658[0n]|BOLD:ABX6174
Dolichogenidea alejandromasisi|[179]|DHJPAR0048134|Costa Rica|658[0n]|BOLD:ABX6174
Dolichogenidea alejandromasisi|[180]|DHJPAR0048130|Costa Rica|658[0n]|BOLD:ABX6174
Dolichogenidea alejandromasisi|[181]|DHJPAR0047086|Costa Rica|658[0n]|BOLD:ABX6174
Dolichogenidea alejandromasisi|[182]|DHJPAR0047080|Costa Rica|658[0n]|BOLD:ABX6174
Dolichogenidea alejandromasisi|[183]|DHJPAR0047076|Costa Rica|658[0n]|BOLD:ABX6174
Dolichogenidea alejandromasisi|[184]|DHJPAR0045264|Costa Rica|658[0n]|BOLD:ABX6174
Dolichogenidea alejandromasisi|[185]|DHJPAR0045182|Costa Rica|658[0n]|BOLD:ABX6174
Dolichogenidea alejandromasisi|[186]|DHJPAR0042935|Costa Rica|658[0n]|BOLD:ABX6174
Dolichogenidea alejandromasisi|[187]|DHJPAR0042930|Costa Rica|658[0n]|BOLD:ABX6174
Dolichogenidea alejandromasisi|[188]|DHJPAR0042929|Costa Rica|658[0n]|BOLD:ABX6174
Dolichogenidea alejandromasisi|[189]|DHJPAR0042927|Costa Rica|658[0n]|BOLD:ABX6174
Dolichogenidea alejandromasisi|[190]|DHJPAR0042906|Costa Rica|658[0n]|BOLD:ABX6174
Dolichogenidea alejandromasisi|[191]|DHJPAR0042509|Costa Rica|658[0n]|BOLD:ABX6174
Dolichogenidea alejandromasisi|[192]|DHJPAR0041805|Costa Rica|658[0n]|BOLD:ABX6174
Dolichogenidea alejandromasisi|[193]|DHJPAR0041798|Costa Rica|658[0n]|BOLD:ABX6174
Dolichogenidea alejandromasisi|[194]|DHJPAR0041791|Costa Rica|658[0n]|BOLD:ABX6174
Dolichogenidea alejandromasisi|[195]|DHJPAR0041749|Costa Rica|658[0n]|BOLD:ABX6174
Dolichogenidea alejandromasisi|[196]|DHJPAR0041643|Costa Rica|658[0n]|BOLD:ABX6174
Dolichogenidea alejandromasisi|[197]|DHJPAR0041623|Costa Rica|658[0n]|BOLD:ABX6174

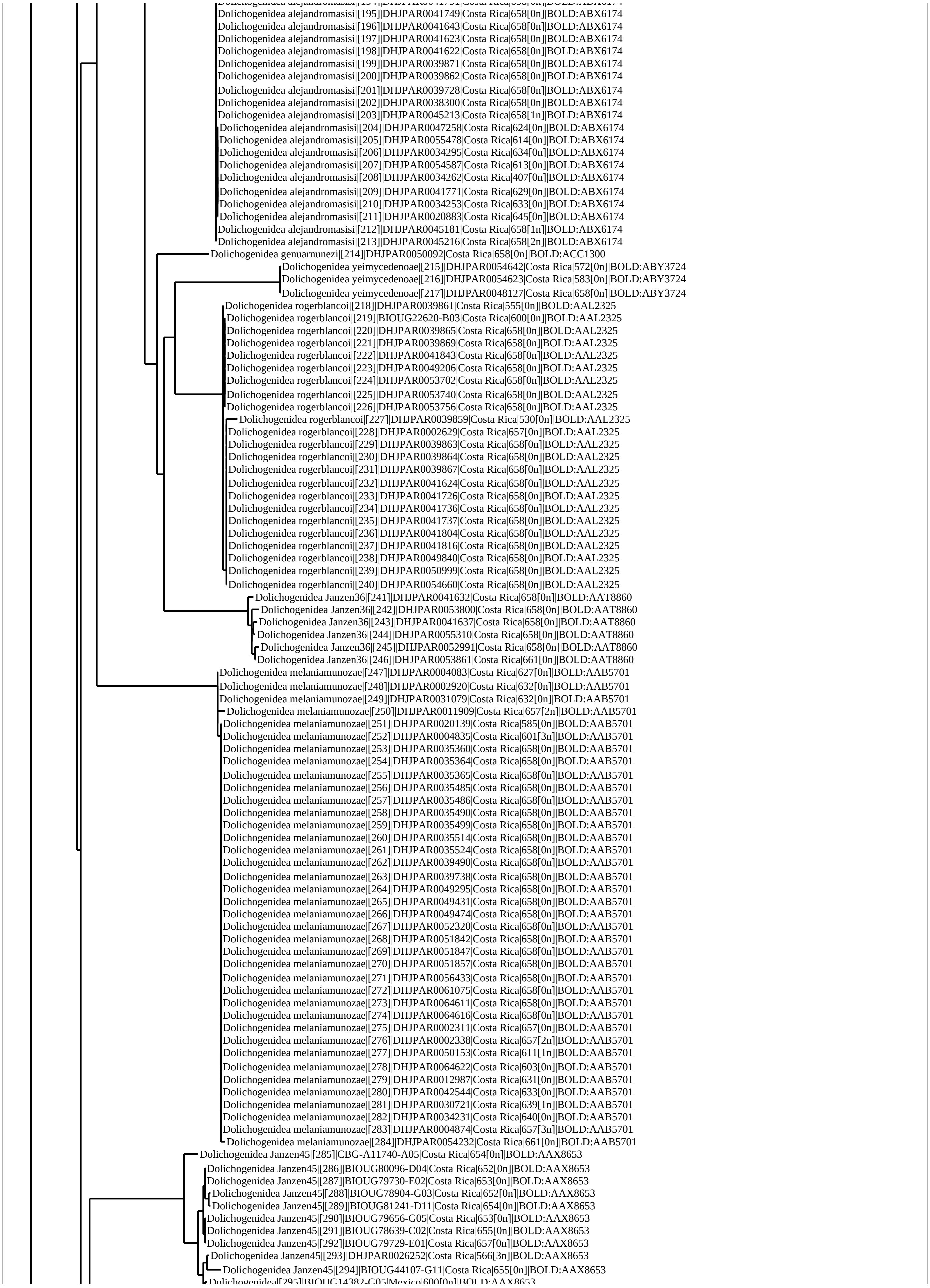
Dolichogenidea alejandromasisi|[194]|DHJPAR0041791|Costa Rica|658[0n]|BOLD:ABX6174
Dolichogenidea alejandromasisi|[195]|DHJPAR0041749|Costa Rica|658[0n]|BOLD:ABX6174
Dolichogenidea alejandromasisi|[196]|DHJPAR0041643|Costa Rica|658[0n]|BOLD:ABX6174
Dolichogenidea alejandromasisi|[197]|DHJPAR0041623|Costa Rica|658[0n]|BOLD:ABX6174
Dolichogenidea alejandromasisi|[198]|DHJPAR0041622|Costa Rica|658[0n]|BOLD:ABX6174
Dolichogenidea alejandromasisi|[199]|DHJPAR0039871|Costa Rica|658[0n]|BOLD:ABX6174
Dolichogenidea alejandromasisi|[200]|DHJPAR0039862|Costa Rica|658[0n]|BOLD:ABX6174
Dolichogenidea alejandromasisi|[201]|DHJPAR0039728|Costa Rica|658[0n]|BOLD:ABX6174
Dolichogenidea alejandromasisi|[202]|DHJPAR0038300|Costa Rica|658[0n]|BOLD:ABX6174
Dolichogenidea alejandromasisi|[203]|DHJPAR0045213|Costa Rica|658[1n]|BOLD:ABX6174
Dolichogenidea alejandromasisi|[204]|DHJPAR0047258|Costa Rica|624[0n]|BOLD:ABX6174
Dolichogenidea alejandromasisi|[205]|DHJPAR0055478|Costa Rica|614[0n]|BOLD:ABX6174
Dolichogenidea alejandromasisi|[206]|DHJPAR0034295|Costa Rica|634[0n]|BOLD:ABX6174
Dolichogenidea alejandromasisi|[207]|DHJPAR0054587|Costa Rica|613[0n]|BOLD:ABX6174
Dolichogenidea alejandromasisi|[208]|DHJPAR0034262|Costa Rica|407[0n]|BOLD:ABX6174
Dolichogenidea alejandromasisi|[209]|DHJPAR0041771|Costa Rica|629[0n]|BOLD:ABX6174
Dolichogenidea alejandromasisi|[210]|DHJPAR0034253|Costa Rica|633[0n]|BOLD:ABX6174
Dolichogenidea alejandromasisi|[211]|DHJPAR0020883|Costa Rica|645[0n]|BOLD:ABX6174
Dolichogenidea alejandromasisi|[212]|DHJPAR0045181|Costa Rica|658[1n]|BOLD:ABX6174
Dolichogenidea alejandromasisi|[213]|DHJPAR0045216|Costa Rica|658[2n]|BOLD:ABX6174
Dolichogenidea genuarnunezi|[214]|DHJPAR0050092|Costa Rica|658[0n]|BOLD:ACC1300
Dolichogenidea yeimycedenoae|[215]|DHJPAR0054642|Costa Rica|572[0n]|BOLD:ABY3724
Dolichogenidea yeimycedenoae|[216]|DHJPAR0054623|Costa Rica|583[0n]|BOLD:ABY3724
Dolichogenidea yeimycedenoae|[217]|DHJPAR0048127|Costa Rica|658[0n]|BOLD:ABY3724
Dolichogenidea rogerblancoi|[218]|DHJPAR0039861|Costa Rica|555[0n]|BOLD:AAL2325
Dolichogenidea rogerblancoi|[219]|BIOUG22620-B03|Costa Rica|600[0n]|BOLD:AAL2325
Dolichogenidea rogerblancoi|[220]|DHJPAR0039865|Costa Rica|658[0n]|BOLD:AAL2325
Dolichogenidea rogerblancoi|[221]|DHJPAR0039869|Costa Rica|658[0n]|BOLD:AAL2325
Dolichogenidea rogerblancoi|[222]|DHJPAR0041843|Costa Rica|658[0n]|BOLD:AAL2325
Dolichogenidea rogerblancoi|[223]|DHJPAR0049206|Costa Rica|658[0n]|BOLD:AAL2325
Dolichogenidea rogerblancoi|[224]|DHJPAR0053702|Costa Rica|658[0n]|BOLD:AAL2325
Dolichogenidea rogerblancoi|[225]|DHJPAR0053740|Costa Rica|658[0n]|BOLD:AAL2325
Dolichogenidea rogerblancoi|[226]|DHJPAR0053756|Costa Rica|658[0n]|BOLD:AAL2325
Dolichogenidea rogerblancoi|[227]|DHJPAR0039859|Costa Rica|530[0n]|BOLD:AAL2325
Dolichogenidea rogerblancoi|[228]|DHJPAR0002629|Costa Rica|657[0n]|BOLD:AAL2325
Dolichogenidea rogerblancoi|[229]|DHJPAR0039863|Costa Rica|658[0n]|BOLD:AAL2325
Dolichogenidea rogerblancoi|[230]|DHJPAR0039864|Costa Rica|658[0n]|BOLD:AAL2325
Dolichogenidea rogerblancoi|[231]|DHJPAR0039867|Costa Rica|658[0n]|BOLD:AAL2325
Dolichogenidea rogerblancoi|[232]|DHJPAR0041624|Costa Rica|658[0n]|BOLD:AAL2325
Dolichogenidea rogerblancoi|[233]|DHJPAR0041726|Costa Rica|658[0n]|BOLD:AAL2325
Dolichogenidea rogerblancoi|[234]|DHJPAR0041736|Costa Rica|658[0n]|BOLD:AAL2325
Dolichogenidea rogerblancoi|[235]|DHJPAR0041737|Costa Rica|658[0n]|BOLD:AAL2325
Dolichogenidea rogerblancoi|[236]|DHJPAR0041804|Costa Rica|658[0n]|BOLD:AAL2325
Dolichogenidea rogerblancoi|[237]|DHJPAR0041816|Costa Rica|658[0n]|BOLD:AAL2325
Dolichogenidea rogerblancoi|[238]|DHJPAR0049840|Costa Rica|658[0n]|BOLD:AAL2325
Dolichogenidea rogerblancoi|[239]|DHJPAR0050999|Costa Rica|658[0n]|BOLD:AAL2325
Dolichogenidea rogerblancoi|[240]|DHJPAR0054660|Costa Rica|658[0n]|BOLD:AAL2325
Dolichogenidea Janzen36|[241]|DHJPAR0041632|Costa Rica|658[0n]|BOLD:AAT8860
Dolichogenidea Janzen36|[242]|DHJPAR0053800|Costa Rica|658[0n]|BOLD:AAT8860
Dolichogenidea Janzen36|[243]|DHJPAR0041637|Costa Rica|658[0n]|BOLD:AAT8860
Dolichogenidea Janzen36|[244]|DHJPAR0055310|Costa Rica|658[0n]|BOLD:AAT8860
Dolichogenidea Janzen36|[245]|DHJPAR0052991|Costa Rica|658[0n]|BOLD:AAT8860
Dolichogenidea Janzen36|[246]|DHJPAR0053861|Costa Rica|661[0n]|BOLD:AAT8860
Dolichogenidea melaniamunozae|[247]|DHJPAR0004083|Costa Rica|627[0n]|BOLD:AAB5701
Dolichogenidea melaniamunozae|[248]|DHJPAR0002920|Costa Rica|632[0n]|BOLD:AAB5701
Dolichogenidea melaniamunozae|[249]|DHJPAR0031079|Costa Rica|632[0n]|BOLD:AAB5701
Dolichogenidea melaniamunozae|[250]|DHJPAR0011909|Costa Rica|657[2n]|BOLD:AAB5701
Dolichogenidea melaniamunozae|[251]|DHJPAR0020139|Costa Rica|585[0n]|BOLD:AAB5701
Dolichogenidea melaniamunozae|[252]|DHJPAR0004835|Costa Rica|601[3n]|BOLD:AAB5701
Dolichogenidea melaniamunozae|[253]|DHJPAR0035360|Costa Rica|658[0n]|BOLD:AAB5701
Dolichogenidea melaniamunozae|[254]|DHJPAR0035364|Costa Rica|658[0n]|BOLD:AAB5701
Dolichogenidea melaniamunozae|[255]|DHJPAR0035365|Costa Rica|658[0n]|BOLD:AAB5701
Dolichogenidea melaniamunozae|[256]|DHJPAR0035485|Costa Rica|658[0n]|BOLD:AAB5701
Dolichogenidea melaniamunozae|[257]|DHJPAR0035486|Costa Rica|658[0n]|BOLD:AAB5701
Dolichogenidea melaniamunozae|[258]|DHJPAR0035490|Costa Rica|658[0n]|BOLD:AAB5701
Dolichogenidea melaniamunozae|[259]|DHJPAR0035499|Costa Rica|658[0n]|BOLD:AAB5701
Dolichogenidea melaniamunozae|[260]|DHJPAR0035514|Costa Rica|658[0n]|BOLD:AAB5701
Dolichogenidea melaniamunozae|[261]|DHJPAR0035524|Costa Rica|658[0n]|BOLD:AAB5701
Dolichogenidea melaniamunozae|[262]|DHJPAR0039490|Costa Rica|658[0n]|BOLD:AAB5701
Dolichogenidea melaniamunozae|[263]|DHJPAR0039738|Costa Rica|658[0n]|BOLD:AAB5701
Dolichogenidea melaniamunozae|[264]|DHJPAR0049295|Costa Rica|658[0n]|BOLD:AAB5701
Dolichogenidea melaniamunozae|[265]|DHJPAR0049431|Costa Rica|658[0n]|BOLD:AAB5701
Dolichogenidea melaniamunozae|[266]|DHJPAR0049474|Costa Rica|658[0n]|BOLD:AAB5701
Dolichogenidea melaniamunozae|[267]|DHJPAR0052320|Costa Rica|658[0n]|BOLD:AAB5701
Dolichogenidea melaniamunozae|[268]|DHJPAR0051842|Costa Rica|658[0n]|BOLD:AAB5701
Dolichogenidea melaniamunozae|[269]|DHJPAR0051847|Costa Rica|658[0n]|BOLD:AAB5701
Dolichogenidea melaniamunozae|[270]|DHJPAR0051857|Costa Rica|658[0n]|BOLD:AAB5701
Dolichogenidea melaniamunozae|[271]|DHJPAR0056433|Costa Rica|658[0n]|BOLD:AAB5701
Dolichogenidea melaniamunozae|[272]|DHJPAR0061075|Costa Rica|658[0n]|BOLD:AAB5701
Dolichogenidea melaniamunozae|[273]|DHJPAR0064611|Costa Rica|658[0n]|BOLD:AAB5701
Dolichogenidea melaniamunozae|[274]|DHJPAR0064616|Costa Rica|658[0n]|BOLD:AAB5701
Dolichogenidea melaniamunozae|[275]|DHJPAR0002311|Costa Rica|657[0n]|BOLD:AAB5701
Dolichogenidea melaniamunozae|[276]|DHJPAR0002338|Costa Rica|657[2n]|BOLD:AAB5701
Dolichogenidea melaniamunozae|[277]|DHJPAR0050153|Costa Rica|611[1n]|BOLD:AAB5701
Dolichogenidea melaniamunozae|[278]|DHJPAR0064622|Costa Rica|603[0n]|BOLD:AAB5701
Dolichogenidea melaniamunozae|[279]|DHJPAR0012987|Costa Rica|631[0n]|BOLD:AAB5701
Dolichogenidea melaniamunozae|[280]|DHJPAR0042544|Costa Rica|633[0n]|BOLD:AAB5701
Dolichogenidea melaniamunozae|[281]|DHJPAR0030721|Costa Rica|639[1n]|BOLD:AAB5701
Dolichogenidea melaniamunozae|[282]|DHJPAR0034231|Costa Rica|640[0n]|BOLD:AAB5701
Dolichogenidea melaniamunozae|[283]|DHJPAR0004874|Costa Rica|657[3n]|BOLD:AAB5701
Dolichogenidea melaniamunozae|[284]|DHJPAR0054232|Costa Rica|661[0n]|BOLD:AAB5701
Dolichogenidea Janzen45|[285]|CBG-A11740-A05|Costa Rica|654[0n]|BOLD:AAX8653
Dolichogenidea Janzen45|[286]|BIOUG80096-D04|Costa Rica|652[0n]|BOLD:AAX8653
Dolichogenidea Janzen45|[287]|BIOUG79730-E02|Costa Rica|653[0n]|BOLD:AAX8653
Dolichogenidea Janzen45|[288]|BIOUG78904-G03|Costa Rica|652[0n]|BOLD:AAX8653
Dolichogenidea Janzen45|[289]|BIOUG81241-D11|Costa Rica|654[0n]|BOLD:AAX8653
Dolichogenidea Janzen45|[290]|BIOUG79656-G05|Costa Rica|653[0n]|BOLD:AAX8653
Dolichogenidea Janzen45|[291]|BIOUG78639-C02|Costa Rica|655[0n]|BOLD:AAX8653
Dolichogenidea Janzen45|[292]|BIOUG79729-E01|Costa Rica|657[0n]|BOLD:AAX8653
Dolichogenidea Janzen45|[293]|DHJPAR0026252|Costa Rica|566[3n]|BOLD:AAX8653
Dolichogenidea Janzen45|[294]|BIOUG44107-G11|Costa Rica|655[0n]|BOLD:AAX8653
Dolichogenidea|[295]|BIOUG14382-G05|Mexico|600[0n]|BOLD:AAX8653

Dolichogenidea Janzen45|[293]|DHJPAR0026252|Costa Rica|566[3n]|BOLD:AAX8653
Dolichogenidea Janzen45|[294]|BIOUG44107-G11|Costa Rica|655[0n]|BOLD:AAX8653
Dolichogenidea|[295]|BIOUG14382-G05|Mexico|600[0n]|BOLD:AAX8653
Dolichogenidea Janzen45|[296]|DHJPAR0031740|Costa Rica|658[0n]|BOLD:AAX8653
Dolichogenidea Janzen133|[297]|DHJPAR0049894|Costa Rica|658[0n]|BOLD:ACC1295
Dolichogenidea Janzen133|[298]|DHJPAR0055314|Costa Rica|658[0n]|BOLD:ACC1295
Dolichogenidea Janzen08|[299]|DHJPAR0051148|Costa Rica|614[0n]|BOLD:AAT8840
Dolichogenidea Janzen08|[300]|DHJPAR0050174|Costa Rica|630[0n]|BOLD:AAT8840
Dolichogenidea Janzen08|[301]|DHJPAR0040475|Costa Rica|658[0n]|BOLD:AAT8840
Dolichogenidea Janzen08|[302]|DHJPAR0040477|Costa Rica|658[0n]|BOLD:AAT8840
Dolichogenidea Janzen08|[303]|DHJPAR0040491|Costa Rica|658[0n]|BOLD:AAT8840
Dolichogenidea Janzen08|[304]|DHJPAR0040496|Costa Rica|658[0n]|BOLD:AAT8840
Dolichogenidea Janzen08|[305]|DHJPAR0042017|Costa Rica|658[0n]|BOLD:AAT8840
Dolichogenidea Janzen08|[306]|DHJPAR0042033|Costa Rica|658[0n]|BOLD:AAT8840
Dolichogenidea Janzen08|[307]|DHJPAR0050116|Costa Rica|658[0n]|BOLD:AAT8840
Dolichogenidea|[308]|MACN-Bar-Ins-ct 01863|Argentina|661[0n]|BOLD:AAY4695
Dolichogenidea Janzen116|[309]|DHJPAR0041920|Costa Rica|599[0n]|BOLD:AAY4695
Dolichogenidea Janzen116|[310]|DHJPAR0041886|Costa Rica|407[0n]|BOLD:AAY4695
Dolichogenidea Janzen116|[311]|DHJPAR0041880|Costa Rica|410[0n]|BOLD:AAY4695
Dolichogenidea Janzen116|[312]|DHJPAR0051273|Costa Rica|614[0n]|BOLD:AAY4695
Dolichogenidea Janzen116|[313]|DHJPAR0051275|Costa Rica|618[0n]|BOLD:AAY4695
Dolichogenidea Janzen116|[314]|DHJPAR0051282|Costa Rica|658[0n]|BOLD:AAY4695
Dolichogenidea Janzen116|[315]|DHJPAR0051868|Costa Rica|658[0n]|BOLD:AAY4695
Hymenoptera|[316]|CBG-A03339-D12|Costa Rica|654[0n]|BOLD:AAY4695
Hymenoptera|[317]|CBG-A03302-A06|Costa Rica|655[0n]|BOLD:AAY4695
Dolichogenidea|[318]|MACN-Bar-Ins-ct 01873|Argentina|661[0n]|BOLD:AAY4695
Dolichogenidea Janzen161|[319]|DHJPAR0002324|Costa Rica|657[6n]|BOLD:AAI6323
Dolichogenidea Janzen161|[320]|DHJPAR0002335|Costa Rica|657[0n]|BOLD:AAI6323
Dolichogenidea Janzen161|[321]|DHJPAR0031381|Costa Rica|658[0n]|BOLD:AAI6323
Dolichogenidea Janzen161|[322]|DHJPAR0031385|Costa Rica|658[0n]|BOLD:AAI6323
Dolichogenidea homoeosomae|[323]|CNCHYM 01050|Canada|425[0n]
Dolichogenidea homoeosomae|[324]|CNCHYM 01052|Canada|425[0n]
Hymenoptera|[325]|CBG-A04265-A08|Costa Rica|653[0n]|BOLD:AAX8664
Dolichogenidea Janzen29|[326]|DHJPAR0012545|Costa Rica|657[0n]|BOLD:AAX8664
Dolichogenidea Janzen456|[327]|DHJPAR0031851|Costa Rica|658[0n]|BOLD:AAM5852
Dolichogenidea Janzen15|[328]|DHJPAR0031865|Costa Rica|658[0n]|BOLD:AAM5851
Dolichogenidea Janzen15|[329]|DHJPAR0031866|Costa Rica|658[0n]|BOLD:AAM5851
Dolichogenidea Janzen15|[330]|DHJPAR0031867|Costa Rica|658[0n]|BOLD:AAM5851
Dolichogenidea Janzen15|[331]|DHJPAR0031847|Costa Rica|510[0n]|BOLD:AAM5851
Dolichogenidea Janzen15|[332]|DHJPAR0031701|Costa Rica|658[0n]|BOLD:AAM5851
Dolichogenidea Janzen15|[333]|DHJPAR0031849|Costa Rica|658[0n]|BOLD:AAM5851
Dolichogenidea Janzen15|[334]|DHJPAR0031855|Costa Rica|658[0n]|BOLD:AAM5851
Dolichogenidea Janzen15|[335]|DHJPAR0031856|Costa Rica|658[0n]|BOLD:AAM5851
Dolichogenidea Janzen15|[336]|DHJPAR0031858|Costa Rica|658[0n]|BOLD:AAM5851
Dolichogenidea Janzen15|[337]|DHJPAR0031861|Costa Rica|658[0n]|BOLD:AAM5851
Dolichogenidea Janzen15|[338]|DHJPAR0031868|Costa Rica|658[0n]|BOLD:AAM5851
Dolichogenidea Janzen27|[339]|DHJPAR0012560|Costa Rica|657[0n]|BOLD:AAI9746
Dolichogenidea Janzen27|[340]|DHJPAR0031574|Costa Rica|658[0n]|BOLD:AAI9746
Dolichogenidea Janzen55|[341]|DHJPAR0035371|Costa Rica|658[0n]|BOLD:AAE8612
Dolichogenidea Janzen55|[342]|DHJPAR0039039|Costa Rica|658[0n]|BOLD:AAE8612
Dolichogenidea Janzen55|[343]|DHJPAR0035351|Costa Rica|658[0n]|BOLD:AAE8612
Dolichogenidea Janzen55|[344]|DHJPAR0039464|Costa Rica|658[0n]|BOLD:AAE8612
Dolichogenidea Janzen55|[345]|DHJPAR0049265|Costa Rica|658[0n]|BOLD:AAE8612
Dolichogenidea Janzen55|[346]|DHJPAR0049308|Costa Rica|658[0n]|BOLD:AAE8612
Dolichogenidea Janzen55|[347]|DHJPAR0049423|Costa Rica|658[0n]|BOLD:AAE8612
Dolichogenidea Janzen55|[348]|DHJPAR0049429|Costa Rica|658[0n]|BOLD:AAE8612
Dolichogenidea Janzen55|[349]|DHJPAR0052276|Costa Rica|658[0n]|BOLD:AAE8612
Dolichogenidea Janzen55|[350]|DHJPAR0052277|Costa Rica|658[0n]|BOLD:AAE8612
Dolichogenidea Janzen11|[351]|DHJPAR0004063|Costa Rica|627[0n]|BOLD:ABX6008
Dolichogenidea Janzen11|[352]|DHJPAR0004263|Costa Rica|627[0n]|BOLD:ABX6008
Dolichogenidea Janzen11|[353]|DHJPAR0045131|Costa Rica|627[0n]|BOLD:ABX6008
Dolichogenidea Janzen11|[354]|DHJPAR0060673|Costa Rica|618[0n]|BOLD:ABX6008
Dolichogenidea Janzen11|[355]|DHJPAR0045247|Costa Rica|658[1n]|BOLD:ABX6008
Dolichogenidea Janzen11|[356]|DHJPAR0038954|Costa Rica|658[0n]|BOLD:ABX6008
Dolichogenidea Janzen11|[357]|DHJPAR0038955|Costa Rica|658[0n]|BOLD:ABX6008
Dolichogenidea Janzen11|[358]|DHJPAR0038987|Costa Rica|658[0n]|BOLD:ABX6008
Dolichogenidea Janzen11|[359]|DHJPAR0041668|Costa Rica|658[0n]|BOLD:ABX6008
Dolichogenidea Janzen11|[360]|DHJPAR0042886|Costa Rica|658[0n]|BOLD:ABX6008
Dolichogenidea Janzen11|[361]|DHJPAR0042891|Costa Rica|658[0n]|BOLD:ABX6008
Dolichogenidea Janzen11|[362]|DHJPAR0042933|Costa Rica|658[0n]|BOLD:ABX6008
Dolichogenidea Janzen11|[363]|DHJPAR0042947|Costa Rica|658[0n]|BOLD:ABX6008
Dolichogenidea Janzen11|[364]|DHJPAR0043132|Costa Rica|658[0n]|BOLD:ABX6008
Dolichogenidea Janzen11|[365]|DHJPAR0047097|Costa Rica|658[0n]|BOLD:ABX6008
Dolichogenidea Janzen11|[366]|DHJPAR0047099|Costa Rica|658[0n]|BOLD:ABX6008
Dolichogenidea Janzen11|[367]|DHJPAR0047102|Costa Rica|658[0n]|BOLD:ABX6008
Dolichogenidea Janzen11|[368]|DHJPAR0047112|Costa Rica|658[0n]|BOLD:ABX6008
Dolichogenidea Janzen11|[369]|DHJPAR0047126|Costa Rica|658[0n]|BOLD:ABX6008
Dolichogenidea Janzen11|[370]|DHJPAR0047249|Costa Rica|658[0n]|BOLD:ABX6008
Dolichogenidea Janzen11|[371]|DHJPAR0047250|Costa Rica|658[0n]|BOLD:ABX6008
Dolichogenidea Janzen11|[372]|DHJPAR0048122|Costa Rica|658[0n]|BOLD:ABX6008
Dolichogenidea Janzen11|[373]|DHJPAR0048147|Costa Rica|658[0n]|BOLD:ABX6008
Dolichogenidea Janzen11|[374]|DHJPAR0048148|Costa Rica|658[0n]|BOLD:ABX6008
Dolichogenidea Janzen11|[375]|DHJPAR0048150|Costa Rica|658[0n]|BOLD:ABX6008
Dolichogenidea Janzen11|[376]|DHJPAR0049123|Costa Rica|658[0n]|BOLD:ABX6008
Dolichogenidea Janzen11|[377]|DHJPAR0049167|Costa Rica|658[0n]|BOLD:ABX6008
Dolichogenidea Janzen11|[378]|DHJPAR0049168|Costa Rica|658[0n]|BOLD:ABX6008
Dolichogenidea Janzen11|[379]|DHJPAR0049178|Costa Rica|658[0n]|BOLD:ABX6008
Dolichogenidea Janzen11|[380]|DHJPAR0049179|Costa Rica|658[0n]|BOLD:ABX6008
Dolichogenidea Janzen11|[381]|DHJPAR0049776|Costa Rica|658[0n]|BOLD:ABX6008
Dolichogenidea Janzen11|[382]|DHJPAR0049777|Costa Rica|658[0n]|BOLD:ABX6008
Dolichogenidea Janzen11|[383]|DHJPAR0049819|Costa Rica|658[0n]|BOLD:ABX6008
Dolichogenidea Janzen11|[384]|DHJPAR0049835|Costa Rica|658[0n]|BOLD:ABX6008
Dolichogenidea Janzen11|[385]|DHJPAR0051002|Costa Rica|658[0n]|BOLD:ABX6008
Dolichogenidea Janzen11|[386]|DHJPAR0051005|Costa Rica|658[0n]|BOLD:ABX6008
Dolichogenidea Janzen11|[387]|DHJPAR0051008|Costa Rica|658[0n]|BOLD:ABX6008
Dolichogenidea Janzen11|[388]|DHJPAR0051187|Costa Rica|658[0n]|BOLD:ABX6008
Dolichogenidea Janzen11|[389]|DHJPAR0052906|Costa Rica|658[0n]|BOLD:ABX6008
Dolichogenidea Janzen11|[390]|DHJPAR0052914|Costa Rica|658[0n]|BOLD:ABX6008
Dolichogenidea Janzen11|[391]|DHJPAR0054637|Costa Rica|658[0n]|BOLD:ABX6008
Dolichogenidea Janzen11|[392]|DHJPAR0054675|Costa Rica|658[0n]|BOLD:ABX6008
Dolichogenidea Janzen11|[393]|DHJPAR0054698|Costa Rica|658[0n]|BOLD:ABX6008

Dolichogenidea Janzen11|[391]|DHJPAR0054637|Costa Rica|658[0n]|BOLD:ABX6008
Dolichogenidea Janzen11|[392]|DHJPAR0054675|Costa Rica|658[0n]|BOLD:ABX6008
Dolichogenidea Janzen11|[393]|DHJPAR0054698|Costa Rica|658[0n]|BOLD:ABX6008
Dolichogenidea Janzen11|[394]|DHJPAR0054701|Costa Rica|658[0n]|BOLD:ABX6008
Dolichogenidea Janzen11|[395]|DHJPAR0054702|Costa Rica|658[0n]|BOLD:ABX6008
Dolichogenidea Janzen11|[396]|DHJPAR0054783|Costa Rica|658[0n]|BOLD:ABX6008
Dolichogenidea Janzen11|[397]|DHJPAR0055281|Costa Rica|658[0n]|BOLD:ABX6008
Dolichogenidea Janzen11|[398]|DHJPAR0055455|Costa Rica|658[0n]|BOLD:ABX6008
Dolichogenidea Janzen11|[399]|DHJPAR0056489|Costa Rica|658[0n]|BOLD:ABX6008
Dolichogenidea Janzen11|[400]|DHJPAR0056594|Costa Rica|658[0n]|BOLD:ABX6008
Dolichogenidea Janzen11|[401]|DHJPAR0056601|Costa Rica|658[0n]|BOLD:ABX6008
Dolichogenidea Janzen11|[402]|DHJPAR0060672|Costa Rica|658[0n]|BOLD:ABX6008
Dolichogenidea Janzen11|[403]|DHJPAR0060684|Costa Rica|658[0n]|BOLD:ABX6008
Dolichogenidea Janzen11|[404]|DHJPAR0060686|Costa Rica|658[0n]|BOLD:ABX6008
Dolichogenidea Janzen11|[405]|DHJPAR0061667|Costa Rica|658[0n]|BOLD:ABX6008
Dolichogenidea Janzen11|[406]|DHJPAR0063198|Costa Rica|658[0n]|BOLD:ABX6008
Dolichogenidea Janzen11|[407]|DHJPAR0063200|Costa Rica|658[0n]|BOLD:ABX6008
Dolichogenidea Janzen11|[408]|DHJPAR0063206|Costa Rica|658[0n]|BOLD:ABX6008
Dolichogenidea Janzen11|[409]|DHJPAR0063419|Costa Rica|658[0n]|BOLD:ABX6008
Dolichogenidea Janzen11|[410]|DHJPAR0051010|Costa Rica|626[0n]|BOLD:ABX6008
Dolichogenidea Janzen11|[411]|DHJPAR0038988|Costa Rica|631[0n]|BOLD:ABX6008
Dolichogenidea Janzen11|[412]|DHJPAR0038995|Costa Rica|633[0n]|BOLD:ABX6008
Dolichogenidea Janzen11|[413]|DHJPAR0047251|Costa Rica|633[0n]|BOLD:ABX6008
Dolichogenidea Janzen11|[414]|DHJPAR0060670|Costa Rica|633[0n]|BOLD:ABX6008
Dolichogenidea Janzen11|[415]|DHJPAR0045263|Costa Rica|658[1n]|BOLD:ABX6008
Dolichogenidea Janzen11|[416]|DHJPAR0051777|Costa Rica|661[0n]|BOLD:ABX6008
Dolichogenidea Janzen11|[417]|DHJPAR0051790|Costa Rica|661[0n]|BOLD:ABX6008
Dolichogenidea Janzen1098|[418]|DHJPAR0038983|Costa Rica|658[0n]|BOLD:AAM1098
Dolichogenidea Janzen1098|[419]|DHJPAR0055253|Costa Rica|658[0n]|BOLD:AAM1098
Dolichogenidea Janzen1098|[420]|DHJPAR0055316|Costa Rica|658[0n]|BOLD:AAM1098
Dolichogenidea Janzen8823|[421]|DHJPAR0056592|Costa Rica|517[4n]|BOLD:ACE8823
Dolichogenidea Janzen8823|[422]|DHJPAR0039870|Costa Rica|636[1n]|BOLD:ACE8823
Dolichogenidea Janzen8823|[423]|DHJPAR0040418|Costa Rica|539[0n]|BOLD:ACE8823
Microgastrinae|[424]|CBG-A08277-G10|Costa Rica|652[0n]|BOLD:ACE8823
Dolichogenidea Janzen8823|[425]|DHJPAR0055466|Costa Rica|564[0n]|BOLD:ACE8823
Dolichogenidea Janzen8823|[426]|DHJPAR0038070|Costa Rica|592[1n]|BOLD:ACE8823
Dolichogenidea Janzen8823|[427]|BIOUG97255-C05|Costa Rica|654[1n]|BOLD:ACE8823
Hymenoptera|[428]|CBG-A01339-G01|Costa Rica|654[1n]|BOLD:ACE8823
Dolichogenidea Janzen02|[429]|DHJPAR0041711|Costa Rica|614[2n]|BOLD:AAC2174
Dolichogenidea Janzen02|[430]|DHJPAR0041837|Costa Rica|617[5n]|BOLD:AAC2174
Dolichogenidea Janzen02|[431]|DHJPAR0030848|Costa Rica|539[4n]|BOLD:AAC2174
Dolichogenidea Janzen02|[432]|DHJPAR0040394|Costa Rica|542[2n]|BOLD:AAC2174
Dolichogenidea Janzen8823|[433]|DHJPAR0038157|Costa Rica|576[4n]|BOLD:AAC2174
Dolichogenidea Janzen02|[434]|DHJPAR0042939|Costa Rica|570[0n]|BOLD:AAC2174
Dolichogenidea Janzen02|[435]|DHJPAR0039436|Costa Rica|574[0n]|BOLD:AAC2174
Dolichogenidea Janzen02|[436]|DHJPAR0047091|Costa Rica|622[0n]|BOLD:AAC2174
Dolichogenidea Janzen02|[437]|DHJPAR0042528|Costa Rica|612[1n]|BOLD:AAC2174
Dolichogenidea Janzen02|[438]|DHJPAR0047262|Costa Rica|618[0n]|BOLD:AAC2174
Dolichogenidea Janzen02|[439]|DHJPAR0034213|Costa Rica|610[0n]|BOLD:AAC2174
Dolichogenidea Janzen02|[440]|DHJPAR0042925|Costa Rica|621[0n]|BOLD:AAC2174
Dolichogenidea Janzen02|[441]|DHJPAR0038146|Costa Rica|621[1n]|BOLD:AAC2174
Dolichogenidea Janzen02|[442]|DHJPAR0004270|Costa Rica|627[0n]|BOLD:AAC2174
Dolichogenidea Janzen02|[443]|DHJPAR0004272|Costa Rica|627[0n]|BOLD:AAC2174
Dolichogenidea Janzen02|[444]|DHJPAR0041777|Costa Rica|626[0n]|BOLD:AAC2174
Dolichogenidea Janzen02|[445]|DHJPAR0041618|Costa Rica|624[0n]|BOLD:AAC2174
Dolichogenidea Janzen02|[446]|DHJPAR0041832|Costa Rica|623[0n]|BOLD:AAC2174
Dolichogenidea Janzen02|[447]|DHJPAR0041779|Costa Rica|622[0n]|BOLD:AAC2174
Dolichogenidea Janzen02|[448]|DHJPAR0002283|Costa Rica|657[8n]|BOLD:AAC2174
Dolichogenidea Janzen02|[449]|DHJPAR0002197|Costa Rica|621[0n]|BOLD:AAC2174
Arthropoda|[450]|21-SRNP-31452|Costa Rica|655[0n]|BOLD:AAC2174
Dolichogenidea Janzen02|[451]|BIOUG97050-H02|Costa Rica|654[0n]|BOLD:AAC2174
bracMalaise01 Malaise2174|[452]|CBG-A01392-A06|Costa Rica|653[0n]|BOLD:AAC2174
Dolichogenidea Janzen02|[453]|DHJPAR0002236|Costa Rica|657[0n]|BOLD:AAC2174
Dolichogenidea Janzen02|[454]|DHJPAR0038085|Costa Rica|658[0n]|BOLD:AAC2174
Dolichogenidea Janzen02|[455]|DHJPAR0038111|Costa Rica|658[0n]|BOLD:AAC2174
Dolichogenidea Janzen02|[456]|DHJPAR0038284|Costa Rica|658[0n]|BOLD:AAC2174
Dolichogenidea Janzen02|[457]|DHJPAR0004276|Costa Rica|657[0n]|BOLD:AAC2174
Dolichogenidea Janzen02|[458]|DHJPAR0002232|Costa Rica|657[0n]|BOLD:AAC2174
Dolichogenidea Janzen02|[459]|DHJPAR0053763|Costa Rica|658[0n]|BOLD:AAC2174
Dolichogenidea Janzen02|[460]|DHJPAR0041647|Costa Rica|628[0n]|BOLD:AAC2174
Dolichogenidea Janzen02|[461]|DHJPAR0047270|Costa Rica|623[0n]|BOLD:AAC2174
Dolichogenidea Janzen02|[462]|DHJPAR0056773|Costa Rica|658[0n]|BOLD:AAC2174
Dolichogenidea Janzen02|[463]|DHJPAR0041732|Costa Rica|658[1n]|BOLD:AAC2174
Dolichogenidea Janzen02|[464]|DHJPAR0041829|Costa Rica|623[0n]|BOLD:AAC2174
Dolichogenidea Janzen02|[465]|BIOUG97261-D03|Costa Rica|652[0n]|BOLD:AAC2174
Dolichogenidea Janzen02|[466]|BIOUG97054-A02|Costa Rica|652[0n]|BOLD:AAC2174
Dolichogenidea Janzen02|[467]|DHJPAR0039414|Costa Rica|658[0n]|BOLD:AAC2174
Dolichogenidea Janzen02|[468]|DHJPAR0047090|Costa Rica|658[0n]|BOLD:AAC2174
Dolichogenidea Janzen02|[469]|DHJPAR0004631|Costa Rica|657[0n]|BOLD:AAC2174
Dolichogenidea Janzen02|[470]|DHJPAR0030860|Costa Rica|657[0n]|BOLD:AAC2174
Dolichogenidea Janzen02|[471]|DHJPAR0042902|Costa Rica|632[0n]|BOLD:AAC2174
Dolichogenidea Janzen02|[472]|DHJPAR0038079|Costa Rica|658[1n]|BOLD:AAC2174
Dolichogenidea Janzen02|[473]|DHJPAR0054604|Costa Rica|658[1n]|BOLD:AAC2174
Dolichogenidea Janzen02|[474]|DHJPAR0035418|Costa Rica|658[2n]|BOLD:AAC2174
Dolichogenidea Janzen430|[475]|DHJPAR0051827|Costa Rica|621[0n]|BOLD:ACB1629
Dolichogenidea Janzen430|[476]|DHJPAR0049394|Costa Rica|658[0n]|BOLD:ACB1629
Dolichogenidea Janzen430|[477]|DHJPAR0049415|Costa Rica|658[0n]|BOLD:ACB1629
Dolichogenidea Janzen430|[478]|DHJPAR0051870|Costa Rica|658[0n]|BOLD:ACB1629
Dolichogenidea Janzen430|[479]|DHJPAR0051878|Costa Rica|658[0n]|BOLD:ACB1629
Dolichogenidea Janzen430|[480]|DHJPAR0051880|Costa Rica|658[0n]|BOLD:ACB1629
Dolichogenidea Janzen430|[481]|DHJPAR0051883|Costa Rica|658[0n]|BOLD:ACB1629
Dolichogenidea Janzen430|[482]|DHJPAR0051886|Costa Rica|658[0n]|BOLD:ACB1629
Dolichogenidea Janzen430|[483]|DHJPAR0051818|Costa Rica|661[0n]|BOLD:ACB1629
Dolichogenidea Janzen430|[484]|DHJPAR0051819|Costa Rica|661[0n]|BOLD:ACB1629
Dolichogenidea Janzen430|[485]|DHJPAR0051829|Costa Rica|661[0n]|BOLD:ACB1629
Dolichogenidea Janzen128|[486]|DHJPAR0045297|Costa Rica|658[1n]|BOLD:ABX5620
Dolichogenidea Janzen128|[487]|DHJPAR0043141|Costa Rica|658[0n]|BOLD:ABX5620
Dolichogenidea Janzen128|[488]|DHJPAR0045287|Costa Rica|658[2n]|BOLD:ABX5620
Dolichogenidea Janzen25|[489]|DHJPAR0012546|Costa Rica|657[0n]|BOLD:ABY7999
Dolichogenidea Janzen25|[490]|DHJPAR0012552|Costa Rica|657[0n]|BOLD:ABY7999
Dolichogenidea Janzen25|[491]|DHJPAR0013229|Costa Rica|657[0n]|BOLD:ABY7999

Dolichogenidea Janzen25|[489]|DHJPAR0012546|Costa Rica|657[0n]|BOLD:ABY7999
Dolichogenidea Janzen25|[490]|DHJPAR0012552|Costa Rica|657[0n]|BOLD:ABY7999
Dolichogenidea Janzen25|[491]|DHJPAR0013229|Costa Rica|657[0n]|BOLD:ABY7999
Dolichogenidea Janzen25|[492]|DHJPAR0013221|Costa Rica|657[0n]|BOLD:ABY7999
Dolichogenidea Janzen25|[493]|DHJPAR0013228|Costa Rica|657[1n]|BOLD:ABY7999
Dolichogenidea Janzen25|[494]|DHJPAR0035512|Costa Rica|658[0n]|BOLD:ABY7999
Dolichogenidea Janzen25|[495]|DHJPAR0034104|Costa Rica|658[0n]|BOLD:ABY7999
Dolichogenidea Janzen25|[496]|DHJPAR0034159|Costa Rica|658[0n]|BOLD:ABY7999
Dolichogenidea Janzen25|[497]|DHJPAR0031440|Costa Rica|658[0n]|BOLD:ABY7999
Dolichogenidea Janzen16|[498]|CBG-A12322-G11|Costa Rica|654[0n]|BOLD:AAM5843
Dolichogenidea Janzen16|[499]|DHJPAR0033753|Costa Rica|658[0n]|BOLD:AAM5843
Dolichogenidea Janzen72|[500]|DHJPAR0054803|Costa Rica|631[0n]|BOLD:ACM2280
Dolichogenidea Janzen31|[501]|DHJPAR0003988|Costa Rica|627[6n]|BOLD:AAB4946
Dolichogenidea Janzen328|[502]|DHJPAR0003965|Costa Rica|419[0n]|BOLD:AAB4946
Dolichogenidea Janzen323|[503]|DHJPAR0004274|Costa Rica|627[2n]|BOLD:AAL2285
Dolichogenidea Janzen09|[504]|DHJPAR0004254|Costa Rica|627[1n]|BOLD:AAB4946
Dolichogenidea Janzen09|[505]|DHJPAR0004256|Costa Rica|627[2n]|BOLD:AAB4946
Dolichogenidea Janzen09|[506]|DHJPAR0004071|Costa Rica|470[2n]|BOLD:AAB4946
Dolichogenidea Janzen09|[507]|DHJPAR0003999|Costa Rica|629[0n]|BOLD:AAB4946
Dolichogenidea Janzen09|[508]|DHJPAR0003969|Costa Rica|627[0n]|BOLD:AAB4946
Dolichogenidea Janzen09|[509]|DHJPAR0003987|Costa Rica|627[1n]|BOLD:AAB4946
Dolichogenidea Janzen09|[510]|DHJPAR0002943|Costa Rica|632[0n]|BOLD:AAB4946
Dolichogenidea Janzen09|[511]|DHJPAR0002981|Costa Rica|632[0n]|BOLD:AAB4946
Dolichogenidea Janzen09|[512]|DHJPAR0049137|Costa Rica|555[5n]|BOLD:AAB4946
Dolichogenidea Janzen09|[513]|DHJPAR0012967|Costa Rica|657[2n]|BOLD:AAB4946
Dolichogenidea Janzen09|[514]|DHJPAR0012968|Costa Rica|657[1n]|BOLD:AAB4946
Dolichogenidea Janzen09|[515]|DHJPAR0004269|Costa Rica|629[4n]|BOLD:AAB4946
Dolichogenidea Janzen09|[516]|DHJPAR0001620|Costa Rica|657[0n]|BOLD:AAB4946
Dolichogenidea Janzen09|[517]|DHJPAR0004247|Costa Rica|657[0n]|BOLD:AAB4946
Dolichogenidea Janzen09|[518]|DHJPAR0004253|Costa Rica|657[0n]|BOLD:AAB4946
Dolichogenidea Janzen09|[519]|DHJPAR0004265|Costa Rica|657[0n]|BOLD:AAB4946
Dolichogenidea Janzen09|[520]|DHJPAR0004283|Costa Rica|657[0n]|BOLD:AAB4946
Dolichogenidea Janzen09|[521]|DHJPAR0004286|Costa Rica|657[0n]|BOLD:AAB4946
Dolichogenidea Janzen09|[522]|DHJPAR0012304|Costa Rica|657[0n]|BOLD:AAB4946
Dolichogenidea Janzen09|[523]|DHJPAR0012310|Costa Rica|657[0n]|BOLD:AAB4946
Dolichogenidea Janzen09|[524]|DHJPAR0012389|Costa Rica|657[0n]|BOLD:AAB4946
Dolichogenidea Janzen09|[525]|DHJPAR0012760|Costa Rica|657[0n]|BOLD:AAB4946
Dolichogenidea Janzen09|[526]|DHJPAR0025998|Costa Rica|657[0n]|BOLD:AAB4946
Dolichogenidea Janzen09|[527]|DHJPAR0038071|Costa Rica|658[0n]|BOLD:AAB4946
Dolichogenidea Janzen09|[528]|DHJPAR0038298|Costa Rica|658[0n]|BOLD:AAB4946
Dolichogenidea Janzen09|[529]|DHJPAR0038973|Costa Rica|658[0n]|BOLD:AAB4946
Dolichogenidea Janzen09|[530]|DHJPAR0053718|Costa Rica|658[0n]|BOLD:AAB4946
Dolichogenidea Janzen09|[531]|DHJPAR0063404|Costa Rica|658[0n]|BOLD:AAB4946
Dolichogenidea Janzen09|[532]|DHJPAR0064045|Costa Rica|658[0n]|BOLD:AAB4946
Dolichogenidea Rodriguez90|[533]|DHJPAR0034254|Costa Rica|631[0n]|BOLD:AAK2061
Dolichogenidea Rodriguez90|[534]|DHJPAR0038025|Costa Rica|658[4n]|BOLD:AAK2061
Dolichogenidea Janzen28|[535]|BIOUG78250-A04|Costa Rica|654[0n]|BOLD:AAE8596
Dolichogenidea Janzen28|[536]|BIOUG87420-F12|Costa Rica|651[0n]|BOLD:AAE8596
Dolichogenidea Janzen28|[537]|DHJPAR0027648|Costa Rica|657[1n]|BOLD:AAE8596
Dolichogenidea Janzen28|[538]|DHJPAR0013518|Costa Rica|657[0n]|BOLD:AAE8596
Dolichogenidea Janzen28|[539]|DHJPAR0031511|Costa Rica|658[0n]|BOLD:AAE8596
Dolichogenidea Janzen28|[540]|BIOUG31516-E04|Costa Rica|603[0n]|BOLD:AAE8596
Microgastrinae|[541]|CBG-A06859-D03|Costa Rica|654[0n]|BOLD:AAE8596
Hymenoptera|[542]|CBG-A06031-F11|Costa Rica|653[0n]|BOLD:AAE8596
Dolichogenidea Janzen28|[543]|DHJPAR0031451|Costa Rica|658[0n]|BOLD:AAE8596
Dolichogenidea Janzen28|[544]|DHJPAR0031454|Costa Rica|658[0n]|BOLD:AAE8596
Dolichogenidea Janzen28|[545]|DHJPAR0031475|Costa Rica|658[0n]|BOLD:AAE8596
Dolichogenidea Janzen28|[546]|DHJPAR0031476|Costa Rica|658[0n]|BOLD:AAE8596
Dolichogenidea Janzen28|[547]|DHJPAR0031477|Costa Rica|658[0n]|BOLD:AAE8596
Dolichogenidea Janzen28|[548]|DHJPAR0031482|Costa Rica|658[0n]|BOLD:AAE8596
Dolichogenidea Janzen28|[549]|DHJPAR0031483|Costa Rica|658[0n]|BOLD:AAE8596
Dolichogenidea Janzen28|[550]|DHJPAR0031484|Costa Rica|658[0n]|BOLD:AAE8596
Dolichogenidea Janzen28|[551]|DHJPAR0031519|Costa Rica|658[0n]|BOLD:AAE8596
Dolichogenidea Janzen28|[552]|DHJPAR0031544|Costa Rica|658[0n]|BOLD:AAE8596
Dolichogenidea Janzen28|[553]|DHJPAR0012550|Costa Rica|655[0n]|BOLD:AAE8596
Dolichogenidea Janzen28|[554]|DHJPAR0013459|Costa Rica|657[0n]|BOLD:AAE8596
Dolichogenidea Janzen28|[555]|DHJPAR0046031|Costa Rica|658[0n]|BOLD:AAE8596
Exoryza yeimycedenoae|[556]|DHJPAR0013235|Costa Rica|656[2n]|BOLD:ABX6267
Hymenoptera|[557]|CBG-A04265-H08|Costa Rica|655[0n]|BOLD:ABX6267
Hymenoptera|[558]|CBG-A04261-F07|Costa Rica|654[0n]|BOLD:ABX6267
Hymenoptera|[559]|CBG-A06372-H10|Costa Rica|654[0n]|BOLD:ABX6267
Hymenoptera|[560]|CBG-A04261-H03|Costa Rica|653[0n]|BOLD:ABX6267
Exoryza yeimycedenoae|[561]|BIOUG70586-C06|Costa Rica|651[0n]|BOLD:ABX6267
Exoryza yeimycedenoae|[562]|DHJPAR0013223|Costa Rica|657[0n]|BOLD:ABX6267
Exoryza yeimycedenoae|[563]|DHJPAR0013222|Costa Rica|657[0n]|BOLD:ABX6267
Exoryza yeimycedenoae|[564]|DHJPAR0012557|Costa Rica|657[0n]|BOLD:ABX6267
Exoryza yeimycedenoae|[565]|DHJPAR0013220|Costa Rica|657[0n]|BOLD:ABX6267
Exoryza yeimycedenoae|[566]|DHJPAR0031496|Costa Rica|658[0n]|BOLD:ABX6267
Exoryza ritaashleyae|[567]|BIOUG70585-C07|Costa Rica|654[0n]|BOLD:ABX6267
Exoryza ritaashleyae|[568]|BIOUG71122-C12|Costa Rica|654[0n]|BOLD:ABX6267
Exoryza ritaashleyae|[569]|DHJPAR0034091|Costa Rica|658[0n]|BOLD:ABX6267
Exoryza ritaashleyae|[570]|DHJPAR0034103|Costa Rica|658[0n]|BOLD:ABX6267
Exoryza ritaashleyae|[571]|DHJPAR0034150|Costa Rica|658[0n]|BOLD:ABX6267
Exoryza richardashleyi|[572]|BIOUG84210-E08|Costa Rica|652[0n]|BOLD:ABX6267
Exoryza richardashleyi|[573]|BIOUG89258-E08|Costa Rica|653[0n]|BOLD:ABX6267
Exoryza richardashleyi|[574]|BIOUG86531-D12|Costa Rica|654[0n]|BOLD:ABX6267
Exoryza richardashleyi|[575]|BIOUG89258-B08|Costa Rica|654[0n]|BOLD:ABX6267
Exoryza richardashleyi|[576]|DHJPAR0013346|Costa Rica|657[0n]|BOLD:ABX6267
Exoryza richardashleyi|[577]|DHJPAR0031469|Costa Rica|658[0n]|BOLD:ABX6267
Exoryza richardashleyi|[578]|DHJPAR0040489|Costa Rica|658[0n]|BOLD:ABX6267
Exoryza richardashleyi|[579]|DHJPAR0042036|Costa Rica|658[0n]|BOLD:ABX6267
Dolichogenidea Janzen312|[580]|DHJPAR0043034|Costa Rica|658[0n]|BOLD:ABA7252
Dolichogenidea Janzen312|[581]|DHJPAR0043054|Costa Rica|658[0n]|BOLD:ABA7252
Exoryza mariabustosae|[582]|DHJPAR0039483|Costa Rica|624[0n]|BOLD:AAN2496
Exoryza mariabustosae|[583]|DHJPAR0039488|Costa Rica|658[0n]|BOLD:AAN2496
Exoryza mariabustosae|[584]|DHJPAR0039467|Costa Rica|658[0n]|BOLD:AAN2496
Exoryza mariabustosae|[585]|DHJPAR0040481|Costa Rica|658[0n]|BOLD:AAN2496
Exoryza mariabustosae|[586]|DHJPAR0048194|Costa Rica|658[0n]|BOLD:AAN2496
Exoryza mariabustosae|[587]|DHJPAR0048197|Costa Rica|658[0n]|BOLD:AAN2496
Exoryza mariabustosae|[588]|DHJPAR0048176|Costa Rica|658[0n]|BOLD:AAN2496
Exoryza mariabustosae|[589]|DHJPAR0048181|Costa Rica|658[0n]|BOLD:AAN2496
[illegible]

Exoryza mariabustosae|[587]|DHJPAR0048197|Costa Rica|658[0n]|BOLD:AAN2496
Exoryza mariabustosae|[588]|DHJPAR0048176|Costa Rica|658[0n]|BOLD:AAN2496
Exoryza mariabustosae|[589]|DHJPAR0048181|Costa Rica|658[0n]|BOLD:AAN2496
Exoryza mariabustosae|[590]|DHJPAR0040499|Costa Rica|658[0n]|BOLD:AAN2496
Exoryza mariabustosae|[591]|DHJPAR0042004|Costa Rica|658[0n]|BOLD:AAN2496
Exoryza mariabustosae|[592]|DHJPAR0048202|Costa Rica|658[0n]|BOLD:AAN2496
Exoryza mariabustosae|[593]|DHJPAR0048207|Costa Rica|658[0n]|BOLD:AAN2496
Exoryza mariabustosae|[594]|DHJPAR0048208|Costa Rica|658[0n]|BOLD:AAN2496
Exoryza mariabustosae|[595]|DHJPAR0051184|Costa Rica|614[0n]|BOLD:AAN2496
Exoryza mariabustosae|[596]|DHJPAR0052269|Costa Rica|658[0n]|BOLD:AAN2496
Exoryza mariabustosae|[597]|DHJPAR0052281|Costa Rica|658[0n]|BOLD:AAN2496
Exoryza mariabustosae|[598]|DHJPAR0051074|Costa Rica|658[0n]|BOLD:AAN2496
Exoryza mariabustosae|[599]|DHJPAR0051423|Costa Rica|661[0n]|BOLD:AAN2496
Exoryza rosamatarritae|[600]|DHJPAR0043165|Costa Rica|613[0n]|BOLD:ABY5258
Exoryza rosamatarritae|[601]|DHJPAR0043021|Costa Rica|658[0n]|BOLD:ABY5258
Exoryza rosamatarritae|[602]|DHJPAR0049364|Costa Rica|658[0n]|BOLD:ABY5258
Exoryza rosamatarritae|[603]|DHJPAR0051128|Costa Rica|658[0n]|BOLD:ABY5258
Exoryza rosamatarritae|[604]|DHJPAR0052978|Costa Rica|658[0n]|BOLD:ABY5258
Exoryza rosamatarritae|[605]|DHJPAR0052979|Costa Rica|658[0n]|BOLD:ABY5258
Exoryza rosamatarritae|[606]|DHJPAR0052993|Costa Rica|658[0n]|BOLD:ABY5258
Exoryza rosamatarritae|[607]|DHJPAR0052995|Costa Rica|658[0n]|BOLD:ABY5258
Exoryza rosamatarritae|[608]|DHJPAR0053053|Costa Rica|658[0n]|BOLD:ABY5258
Exoryza rosamatarritae|[609]|DHJPAR0051044|Costa Rica|658[1n]|BOLD:ABY5258
Exoryza rosamatarritae|[610]|DHJPAR0033751|Costa Rica|658[0n]|BOLD:ABY5258
Exoryza rosamatarritae|[611]|DHJPAR0043140|Costa Rica|643[0n]|BOLD:ABY5258
Exoryza rosamatarritae|[612]|DHJPAR0053038|Costa Rica|658[0n]|BOLD:ABY5258
Exoryza rosamatarritae|[613]|DHJPAR0053807|Costa Rica|658[0n]|BOLD:ABY5258
Exoryza rosamatarritae|[614]|DHJPAR0053848|Costa Rica|661[0n]|BOLD:ABY5258
Dolichogenidea Janzen97|[615]|DHJPAR0039854|Costa Rica|569[0n]|BOLD:ABA3469
Dolichogenidea Janzen97|[616]|BIOUG60135-B05|Costa Rica|654[0n]|BOLD:ABA3469
Dolichogenidea Janzen97|[617]|BIOUG63607-A03|Costa Rica|654[0n]|BOLD:ABA3469
Dolichogenidea Janzen97|[618]|DHJPAR0056827|Costa Rica|630[0n]|BOLD:ABA3469
Dolichogenidea Janzen97|[619]|DHJPAR0042974|Costa Rica|658[0n]|BOLD:ABA3469
Dolichogenidea Janzen97|[620]|DHJPAR0043053|Costa Rica|658[0n]|BOLD:ABA3469
Dolichogenidea Janzen97|[621]|DHJPAR0049373|Costa Rica|658[0n]|BOLD:ABA3469
Hymenoptera|[622]|CBG-A04808-B09|Costa Rica|655[0n]|BOLD:ACJ2777
Dolichogenidea Janzen10|[623]|DHJPAR0043093|Costa Rica|658[0n]|BOLD:ACJ2777
Microgastrinae|[624]|CBG-A09168-A10|Costa Rica|653[0n]|BOLD:ACJ2777
Dolichogenidea Janzen10|[625]|DHJPAR0054738|Costa Rica|658[0n]|BOLD:ACJ2777
Dolichogenidea Janzen10|[626]|DHJPAR0062775|Costa Rica|658[0n]|BOLD:ACJ2777
Dolichogenidea Janzen10|[627]|DHJPAR0051072|Costa Rica|658[0n]|BOLD:ACJ2777
Dolichogenidea Janzen10|[628]|DHJPAR0051084|Costa Rica|658[0n]|BOLD:ACJ2777
Dolichogenidea Janzen10|[629]|DHJPAR0051131|Costa Rica|658[0n]|BOLD:ACJ2777
Dolichogenidea Janzen10|[630]|DHJPAR0064199|Costa Rica|658[0n]|BOLD:ACJ2777
Microgastrinae|[631]|CBG-A09933-D04|Costa Rica|673[0n]|BOLD:ACJ2777
Dolichogenidea Janzen50|[632]|DHJPAR0035488|Costa Rica|658[0n]|BOLD:AAD8952
Dolichogenidea Janzen50|[633]|DHJPAR0031299|Costa Rica|658[0n]|BOLD:AAD8952
Dolichogenidea Janzen50|[634]|DHJPAR0031383|Costa Rica|658[0n]|BOLD:AAD8952
Dolichogenidea Janzen50|[635]|DHJPAR0039855|Costa Rica|658[0n]|BOLD:AAD8952
Dolichogenidea Janzen50|[636]|DHJPAR0055289|Costa Rica|658[0n]|BOLD:AAD8952
Dolichogenidea Janzen50|[637]|DHJPAR0055321|Costa Rica|658[0n]|BOLD:AAD8952
Dolichogenidea Janzen50|[638]|DHJPAR0039857|Costa Rica|658[0n]|BOLD:AAD8952
Dolichogenidea Janzen50|[639]|DHJPAR0055517|Costa Rica|658[0n]|BOLD:AAD8952
Dolichogenidea Janzen50|[640]|DHJPAR0040501|Costa Rica|658[0n]|BOLD:AAD8952
Dolichogenidea Janzen50|[641]|DHJPAR0041612|Costa Rica|658[0n]|BOLD:AAD8952
Dolichogenidea Janzen50|[642]|DHJPAR0041613|Costa Rica|658[0n]|BOLD:AAD8952
Dolichogenidea Janzen50|[643]|DHJPAR0041901|Costa Rica|658[0n]|BOLD:AAD8952
Dolichogenidea Janzen50|[644]|DHJPAR0041910|Costa Rica|658[0n]|BOLD:AAD8952
Dolichogenidea Janzen50|[645]|DHJPAR0041970|Costa Rica|658[0n]|BOLD:AAD8952
Dolichogenidea Janzen50|[646]|DHJPAR0041977|Costa Rica|658[0n]|BOLD:AAD8952
Dolichogenidea Janzen50|[647]|DHJPAR0049871|Costa Rica|658[0n]|BOLD:AAD8952
Dolichogenidea Janzen50|[648]|DHJPAR0050138|Costa Rica|658[0n]|BOLD:AAD8952
Dolichogenidea Janzen50|[649]|DHJPAR0053027|Costa Rica|658[0n]|BOLD:AAD8952
Dolichogenidea Janzen50|[650]|DHJPAR0055827|Costa Rica|658[0n]|BOLD:AAD8952
Dolichogenidea Janzen50|[651]|DHJPAR0020464|Costa Rica|657[2n]|BOLD:AAD8952
Dolichogenidea Janzen50|[652]|DHJPAR0039014|Costa Rica|658[0n]|BOLD:AAD8952
Dolichogenidea Janzen50|[653]|DHJPAR0047198|Costa Rica|658[0n]|BOLD:AAD8952
Dolichogenidea Janzen50|[654]|DHJPAR0048179|Costa Rica|658[0n]|BOLD:AAD8952
Dolichogenidea Janzen50|[655]|DHJPAR0041993|Costa Rica|563[1n]|BOLD:AAD8952
Dolichogenidea Janzen50|[656]|DHJPAR0055530|Costa Rica|658[0n]|BOLD:AAD8952
Dolichogenidea Janzen50|[657]|DHJPAR0055297|Costa Rica|658[0n]|BOLD:AAD8952
Dolichogenidea Janzen50|[658]|DHJPAR0061867|Costa Rica|658[0n]|BOLD:AAD8952
Dolichogenidea Janzen14|[659]|DHJPAR0056865|Costa Rica|582[1n]|BOLD:AAD8952
Dolichogenidea Janzen14|[660]|DHJPAR0056868|Costa Rica|582[4n]|BOLD:AAD8952
Dolichogenidea Janzen14|[661]|DHJPAR0050088|Costa Rica|658[0n]|BOLD:AAD8952
Dolichogenidea Janzen14|[662]|DHJPAR0051045|Costa Rica|658[0n]|BOLD:AAD8952
Dolichogenidea Janzen14|[663]|DHJPAR0051119|Costa Rica|613[0n]|BOLD:AAD8952
Dolichogenidea Janzen14|[664]|DHJPAR0020706|Costa Rica|656[0n]|BOLD:AAD8952
Dolichogenidea Janzen14|[665]|DHJPAR0055542|Costa Rica|626[0n]|BOLD:AAD8952
Dolichogenidea Janzen14|[666]|DHJPAR0005015|Costa Rica|657[1n]|BOLD:AAD8952
Dolichogenidea Janzen14|[667]|DHJPAR0051077|Costa Rica|634[0n]|BOLD:AAD8952
Dolichogenidea Janzen14|[668]|DHJPAR0041874|Costa Rica|658[0n]|BOLD:AAD8952
Dolichogenidea Janzen14|[669]|DHJPAR0041900|Costa Rica|658[0n]|BOLD:AAD8952
Dolichogenidea Janzen14|[670]|DHJPAR0041902|Costa Rica|658[0n]|BOLD:AAD8952
Dolichogenidea Janzen14|[671]|DHJPAR0041912|Costa Rica|658[0n]|BOLD:AAD8952
Dolichogenidea Janzen14|[672]|DHJPAR0041973|Costa Rica|658[0n]|BOLD:AAD8952
Dolichogenidea Janzen14|[673]|DHJPAR0042002|Costa Rica|658[0n]|BOLD:AAD8952
Dolichogenidea Janzen14|[674]|DHJPAR0049315|Costa Rica|658[0n]|BOLD:AAD8952
Dolichogenidea Janzen14|[675]|DHJPAR0051123|Costa Rica|658[0n]|BOLD:AAD8952
Dolichogenidea Janzen14|[676]|DHJPAR0054829|Costa Rica|658[0n]|BOLD:AAD8952
Dolichogenidea Janzen14|[677]|DHJPAR0054862|Costa Rica|658[0n]|BOLD:AAD8952
Dolichogenidea Janzen14|[678]|DHJPAR0055309|Costa Rica|658[0n]|BOLD:AAD8952
Dolichogenidea Janzen14|[679]|DHJPAR0051073|Costa Rica|658[0n]|BOLD:AAD8952
Dolichogenidea Janzen14|[680]|DHJPAR0059071|Costa Rica|658[0n]|BOLD:AAD8952
Dolichogenidea Janzen14|[681]|DHJPAR0064191|Costa Rica|658[0n]|BOLD:AAD8952
Dolichogenidea Janzen14|[682]|DHJPAR0039032|Costa Rica|658[0n]|BOLD:AAD8952
Dolichogenidea Janzen14|[683]|DHJPAR0056860|Costa Rica|581[5n]|BOLD:AAD8952
Dolichogenidea Janzen14|[684]|DHJPAR0020465|Costa Rica|628[6n]|BOLD:AAD8952
Dolichogenidea Janzen14|[685]|DHJPAR0040520|Costa Rica|642[0n]|BOLD:AAD8952
Dolichogenidea Janzen14|[686]|DHJPAR0020677|Costa Rica|657[0n]|BOLD:AAD8952
Dolichogenidea Janzen14|[687]|DHJPAR0020793|Costa Rica|657[0n]|BOLD:AAD8952
Dolichogenidea Janzen14|[688]|DHJPAR005549[illegible]|Costa Rica|658[0n]|BOLD:AAD8952

Dolichogenidea Janzen14|[685]|DHJPAR0046520|Costa Rica|642[0n]|BOLD:AAD8952
Dolichogenidea Janzen14|[686]|DHJPAR0020677|Costa Rica|657[0n]|BOLD:AAD8952
Dolichogenidea Janzen14|[687]|DHJPAR0020793|Costa Rica|657[0n]|BOLD:AAD8952
Dolichogenidea Janzen14|[688]|DHJPAR0055492|Costa Rica|658[0n]|BOLD:AAD8952
Dolichogenidea Janzen14|[689]|DHJPAR0020676|Costa Rica|657[0n]|BOLD:AAD8952
Dolichogenidea Janzen14|[690]|DHJPAR0056837|Costa Rica|642[0n]|BOLD:AAD8952
Dolichogenidea Janzen14|[691]|DHJPAR0064098|Costa Rica|658[0n]|BOLD:AAD8952
Dolichogenidea Janzen14|[692]|DHJPAR0064120|Costa Rica|658[0n]|BOLD:AAD8952
Dolichogenidea Janzen14|[693]|DHJPAR0056864|Costa Rica|661[0n]|BOLD:AAD8952
Dolichogenidea Janzen120|[694]|DHJPAR0049872|Costa Rica|658[0n]|BOLD:AAY4690
Dolichogenidea Janzen120|[695]|DHJPAR0049874|Costa Rica|658[0n]|BOLD:AAY4690
Dolichogenidea Janzen120|[696]|DHJPAR0054722|Costa Rica|658[0n]|BOLD:AAY4690
Dolichogenidea Janzen120|[697]|DHJPAR0054823|Costa Rica|658[0n]|BOLD:AAY4690
Dolichogenidea Janzen120|[698]|DHJPAR0042038|Costa Rica|658[0n]|BOLD:AAY4690
Dolichogenidea Janzen120|[699]|DHJPAR0049863|Costa Rica|658[0n]|BOLD:AAY4690
Dolichogenidea Janzen120|[700]|DHJPAR0041888|Costa Rica|658[0n]|BOLD:AAY4690
Dolichogenidea Janzen120|[701]|DHJPAR0041896|Costa Rica|658[0n]|BOLD:AAY4690
Dolichogenidea Janzen120|[702]|DHJPAR0056628|Costa Rica|658[0n]|BOLD:AAY4690
Dolichogenidea Janzen120|[703]|DHJPAR0041979|Costa Rica|658[0n]|BOLD:AAY4690
Dolichogenidea Janzen120|[704]|DHJPAR0064106|Costa Rica|658[0n]|BOLD:AAY4690
Dolichogenidea Janzen120|[705]|DHJPAR0064108|Costa Rica|658[0n]|BOLD:AAY4690
Dolichogenidea Janzen5847|[706]|BIOUG52311-A10|Costa Rica|655[0n]|BOLD:AAM5847
Dolichogenidea Janzen5847|[707]|BIOUG52311-B05|Costa Rica|657[0n]|BOLD:AAM5847
Dolichogenidea Janzen5847|[708]|BIOUG58493-C05|Costa Rica|656[0n]|BOLD:AAM5847
Dolichogenidea Janzen5847|[709]|BIOUG53256-E01|Costa Rica|653[0n]|BOLD:AAM5847
Dolichogenidea Janzen5847|[710]|BIOUG54199-E04|Costa Rica|653[0n]|BOLD:AAM5847
Dolichogenidea Janzen5847|[711]|BIOUG63822-F09|Costa Rica|653[0n]|BOLD:AAM5847
Dolichogenidea Janzen5847|[712]|BIOUG64840-E10|Costa Rica|653[0n]|BOLD:AAM5847
Dolichogenidea Janzen5847|[713]|BIOUG59834-G05|Costa Rica|653[0n]|BOLD:AAM5847
Dolichogenidea Janzen5847|[714]|CBG-A17863-B09|Costa Rica|653[0n]|BOLD:AAM5847
Dolichogenidea Janzen5847|[715]|BIOUG51361-C03|Costa Rica|653[0n]|BOLD:AAM5847
Dolichogenidea Janzen5847|[716]|BIOUG52312-C02|Costa Rica|653[0n]|BOLD:AAM5847
Dolichogenidea Janzen5847|[717]|BIOUG44676-F04|Costa Rica|654[0n]|BOLD:AAM5847
Dolichogenidea Janzen5847|[718]|BIOUG51392-B01|Costa Rica|654[0n]|BOLD:AAM5847
Dolichogenidea Janzen5847|[719]|BIOUG52311-H04|Costa Rica|654[0n]|BOLD:AAM5847
Dolichogenidea Janzen5847|[720]|BIOUG54543-B09|Costa Rica|654[0n]|BOLD:AAM5847
Dolichogenidea Janzen5847|[721]|BIOUG58497-A09|Costa Rica|654[0n]|BOLD:AAM5847
Dolichogenidea Janzen5847|[722]|BIOUG59163-B02|Costa Rica|654[0n]|BOLD:AAM5847
Dolichogenidea Janzen5847|[723]|BIOUG60193-C08|Costa Rica|654[0n]|BOLD:AAM5847
Dolichogenidea Janzen5847|[724]|BIOUG61592-D10|Costa Rica|654[0n]|BOLD:AAM5847
Dolichogenidea Janzen5847|[725]|BIOUG59498-H01|Costa Rica|654[0n]|BOLD:AAM5847
Dolichogenidea Janzen5847|[726]|BIOUG63826-A05|Costa Rica|654[0n]|BOLD:AAM5847
Dolichogenidea Janzen5847|[727]|CBG-A17864-A09|Costa Rica|654[0n]|BOLD:AAM5847
Dolichogenidea Janzen5847|[728]|CBG-A17279-H02|Costa Rica|654[0n]|BOLD:AAM5847
Dolichogenidea Janzen5847|[729]|BIOUG52597-B10|Costa Rica|655[0n]|BOLD:AAM5847
Dolichogenidea Janzen5847|[730]|BIOUG60574-H03|Costa Rica|655[0n]|BOLD:AAM5847
Dolichogenidea Janzen5847|[731]|BIOUG62764-F10|Costa Rica|655[0n]|BOLD:AAM5847
Dolichogenidea Janzen5847|[732]|BIOUG63629-E02|Costa Rica|655[0n]|BOLD:AAM5847
Dolichogenidea Janzen5847|[733]|BIOUG50360-E06|Costa Rica|656[0n]|BOLD:AAM5847
Dolichogenidea Janzen5847|[734]|BIOUG54060-E06|Costa Rica|656[0n]|BOLD:AAM5847
Dolichogenidea Janzen5847|[735]|BIOUG52313-E12|Costa Rica|653[0n]|BOLD:AAM5847
Dolichogenidea Janzen5847|[736]|BIOUG51361-B02|Costa Rica|652[0n]|BOLD:AAM5847
Dolichogenidea Janzen5847|[737]|BIOUG59105-G06|Costa Rica|652[0n]|BOLD:AAM5847
Dolichogenidea Janzen5847|[738]|BIOUG59769-D10|Costa Rica|652[0n]|BOLD:AAM5847
Dolichogenidea Janzen5847|[739]|BIOUG60617-E05|Costa Rica|652[0n]|BOLD:AAM5847
Dolichogenidea Janzen5847|[740]|BIOUG61265-B11|Costa Rica|652[0n]|BOLD:AAM5847
Dolichogenidea Janzen5847|[741]|BIOUG59494-B12|Costa Rica|644[0n]|BOLD:AAM5847
Dolichogenidea Janzen5847|[742]|BIOUG52336-A04|Costa Rica|640[0n]|BOLD:AAM5847
Dolichogenidea Janzen5847|[743]|BIOUG62765-B02|Costa Rica|652[0n]|BOLD:AAM5847
Dolichogenidea Janzen5847|[744]|BIOUG62116-C10|Costa Rica|652[0n]|BOLD:AAM5847
Dolichogenidea Janzen5847|[745]|BIOUG60571-E09|Costa Rica|651[0n]|BOLD:AAM5847
Dolichogenidea Janzen5847|[746]|BIOUG62116-C06|Costa Rica|651[0n]|BOLD:AAM5847
Dolichogenidea Janzen5847|[747]|BIOUG46544-F07|Costa Rica|640[0n]|BOLD:AAM5847
Dolichogenidea Janzen5847|[748]|BIOUG29072-F02|Costa Rica|600[0n]|BOLD:AAM5847
Dolichogenidea Janzen5847|[749]|BIOUG29681-F04|Costa Rica|600[0n]|BOLD:AAM5847
Dolichogenidea Janzen5847|[750]|BIOUG29717-B11|Costa Rica|591[0n]|BOLD:AAM5847
Dolichogenidea Janzen5847|[751]|BIOUG52334-G12|Costa Rica|640[0n]|BOLD:AAM5847
Dolichogenidea Janzen5847|[752]|BIOUG59167-G03|Costa Rica|642[0n]|BOLD:AAM5847
Dolichogenidea Janzen5847|[753]|BIOUG60781-D05|Costa Rica|653[0n]|BOLD:AAM5847
Dolichogenidea Janzen5847|[754]|BIOUG62192-B01|Costa Rica|653[0n]|BOLD:AAM5847
Dolichogenidea Janzen5847|[755]|BIOUG44186-B12|Costa Rica|654[0n]|BOLD:AAM5847
Dolichogenidea Janzen5847|[756]|BIOUG44678-D11|Costa Rica|654[0n]|BOLD:AAM5847
Dolichogenidea Janzen5847|[757]|BIOUG44638-E06|Costa Rica|654[0n]|BOLD:AAM5847
Dolichogenidea Janzen5847|[758]|BIOUG52094-F06|Costa Rica|654[0n]|BOLD:AAM5847
Dolichogenidea Janzen5847|[759]|BIOUG59171-H11|Costa Rica|654[0n]|BOLD:AAM5847
Dolichogenidea Janzen5847|[760]|BIOUG52094-E10|Costa Rica|655[0n]|BOLD:AAM5847
Dolichogenidea Janzen5847|[761]|BIOUG28761-G10|Costa Rica|600[0n]|BOLD:AAM5847
Dolichogenidea Janzen5847|[762]|BIOUG29660-F02|Costa Rica|600[0n]|BOLD:AAM5847
Dolichogenidea Janzen5847|[763]|BIOUG29660-F09|Costa Rica|600[0n]|BOLD:AAM5847
Dolichogenidea Janzen5847|[764]|BIOUG29712-B06|Costa Rica|597[0n]|BOLD:AAM5847
Dolichogenidea Janzen5847|[765]|BIOUG29073-F05|Costa Rica|576[0n]|BOLD:AAM5847
Dolichogenidea Janzen5847|[766]|BIOUG29073-F01|Costa Rica|585[0n]|BOLD:AAM5847
Dolichogenidea Janzen5847|[767]|BIOUG29660-B10|Costa Rica|585[0n]|BOLD:AAM5847
Dolichogenidea Janzen5847|[768]|BIOUG29660-A07|Costa Rica|603[0n]|BOLD:AAM5847
Dolichogenidea Janzen5847|[769]|BIOUG44706-H04|Costa Rica|654[0n]|BOLD:AAM5847
Dolichogenidea Janzen5847|[770]|BIOUG52368-A12|Costa Rica|655[0n]|BOLD:AAM5847
Dolichogenidea Janzen5847|[771]|BIOUG59832-C12|Costa Rica|656[0n]|BOLD:AAM5847
Dolichogenidea Janzen5847|[772]|DHJPAR0031378|Costa Rica|658[0n]|BOLD:AAM5847
Dolichogenidea Janzen5847|[773]|BIOUG52094-E08|Costa Rica|658[0n]|BOLD:AAM5847
Dolichogenidea Janzen32|[774]|DHJPAR0054865|Costa Rica|645[4n]|BOLD:ACC4119
Dolichogenidea Janzen32|[775]|DHJPAR0050163|Costa Rica|658[1n]|BOLD:ACC4119
Dolichogenidea Janzen32|[776]|DHJPAR0051066|Costa Rica|658[0n]|BOLD:ACC4119
Dolichogenidea Janzen32|[777]|DHJPAR0054786|Costa Rica|645[0n]|BOLD:ACC4119
Dolichogenidea Janzen32|[778]|DHJPAR0054724|Costa Rica|630[0n]|BOLD:ACC4119
Dolichogenidea Janzen32|[779]|DHJPAR0054819|Costa Rica|658[2n]|BOLD:ACC4119
Dolichogenidea Janzen21|[780]|DHJPAR0031285|Costa Rica|630[0n]|BOLD:AAM5846
Dolichogenidea Janzen70|[781]|DHJPAR0052330|Costa Rica|658[0n]|BOLD:ACI3397
Dolichogenidea Janzen13|[782]|DHJPAR0004087|Costa Rica|627[0n]|BOLD:AAI9755
Dolichogenidea Janzen13|[783]|BIOUG82127-A06|Costa Rica|653[0n]|BOLD:AAI9755
Dolichogenidea Janzen13|[784]|CBG-A04785-G11|Costa Rica|653[0n]|BOLD:AAI9755
Dolichogenidea Janzen13|[785]|BIOUG79897-H10|Costa Rica|652[0n]|BOLD:AAI9755
Dolichogenidea Janzen13|[786]|BIOUG86109-B07|Costa Rica|653[0n]|BOLD:AAI9755

[illegible]
Dolichogenidea Janzen13|[784]|CBG-A04785-G11|Costa Rica|653[0n]|BOLD:AAI9755
Dolichogenidea Janzen13|[785]|BIOUG79897-H10|Costa Rica|652[0n]|BOLD:AAI9755
Dolichogenidea Janzen13|[786]|BIOUG86109-B07|Costa Rica|653[0n]|BOLD:AAI9755
Dolichogenidea Janzen13|[787]|BIOUG86129-D01|Costa Rica|654[0n]|BOLD:AAI9755
Dolichogenidea Janzen13|[788]|DHJPAR0025485|Costa Rica|657[0n]|BOLD:AAI9755
Dolichogenidea Janzen13|[789]|DHJPAR0049406|Costa Rica|658[0n]|BOLD:AAI9755
Dolichogenidea Janzen13|[790]|DHJPAR0064121|Costa Rica|658[0n]|BOLD:AAI9755
Dolichogenidea Janzen49|[791]|DHJPAR0027527|Costa Rica|629[0n]|BOLD:AAC5949
Dolichogenidea Janzen49|[792]|DHJPAR0027556|Costa Rica|629[0n]|BOLD:AAC5949
Dolichogenidea Janzen49|[793]|CBG-A01394-F01|Costa Rica|653[0n]|BOLD:AAC5949
Hymenoptera|[794]|CBG-A04836-C10|Costa Rica|653[0n]|BOLD:AAC5949
Hymenoptera|[795]|CBG-A06285-E05|Costa Rica|653[0n]|BOLD:AAC5949
Dolichogenidea Janzen49|[796]|BIOUG97062-B10|Costa Rica|653[0n]|BOLD:AAC5949
Dolichogenidea Janzen49|[797]|DHJPAR0027536|Costa Rica|630[0n]|BOLD:AAC5949
Dolichogenidea Janzen49|[798]|DHJPAR0027696|Costa Rica|603[0n]|BOLD:AAC5949
Dolichogenidea Janzen49|[799]|DHJPAR0051050|Costa Rica|634[0n]|BOLD:AAC5949
Dolichogenidea|[800]|CBG-A12197-D03|Costa Rica|655[0n]|BOLD:AAC5949
Dolichogenidea Janzen49|[801]|DHJPAR0025242|Costa Rica|656[0n]|BOLD:AAC5949
Dolichogenidea Janzen49|[802]|DHJPAR0025251|Costa Rica|656[0n]|BOLD:AAC5949
Dolichogenidea Janzen49|[803]|DHJPAR0025254|Costa Rica|656[0n]|BOLD:AAC5949
Dolichogenidea Janzen49|[804]|DHJPAR0025939|Costa Rica|657[0n]|BOLD:AAC5949
Dolichogenidea Janzen49|[805]|DHJPAR0026105|Costa Rica|657[0n]|BOLD:AAC5949
Dolichogenidea Janzen49|[806]|DHJPAR0026122|Costa Rica|657[0n]|BOLD:AAC5949
Dolichogenidea Janzen49|[807]|DHJPAR0026437|Costa Rica|657[0n]|BOLD:AAC5949
Dolichogenidea Janzen49|[808]|DHJPAR0027546|Costa Rica|657[0n]|BOLD:AAC5949
Dolichogenidea Janzen49|[809]|DHJPAR0039853|Costa Rica|658[0n]|BOLD:AAC5949
Dolichogenidea Janzen49|[810]|DHJPAR0041981|Costa Rica|658[0n]|BOLD:AAC5949
Dolichogenidea Janzen49|[811]|BIOUG93201-F03|Costa Rica|675[0n]|BOLD:AAC5949
Dolichogenidea Rodriguez159|[812]|DHJPAR0031295|Costa Rica|658[0n]|BOLD:AAM5740
Apanteles Janzen28|[813]|DHJPAR0031280|Costa Rica|658[0n]|BOLD:AAM5739
Apanteles Janzen21|[814]|DHJPAR0031380|Costa Rica|658[0n]|BOLD:AAM5848
Apanteles Rodriguez162|[815]|DHJPAR0031233|Costa Rica|658[0n]|BOLD:AAM5738
Apanteles Rodriguez162|[816]|DHJPAR0039982|Costa Rica|658[0n]|BOLD:AAM5738
Apanteles Rodriguez162|[817]|DHJPAR0040384|Costa Rica|658[0n]|BOLD:AAM5738
Apanteles Rodriguez162|[818]|DHJPAR0040398|Costa Rica|658[0n]|BOLD:AAM5738
Apanteles Rodriguez162|[819]|DHJPAR0041652|Costa Rica|658[0n]|BOLD:AAM5738
Apanteles Rodriguez162|[820]|DHJPAR0041700|Costa Rica|658[0n]|BOLD:AAM5738
Dolichogenidea ensiger|[821]|CAM0960|Canada|658[0n]|BOLD:AAA3764
Dolichogenidea ensiger|[822]|BIOUG06471-C10|Canada|538[0n]|BOLD:AAA3764
Dolichogenidea ensiger|[823]|09BBEHY-2030|Canada|658[0n]|BOLD:AAA3764
Dolichogenidea ensiger|[824]|CNCH3194|United States|658[0n]|BOLD:AAA3764
Dolichogenidea ensiger|[825]|BIOUG00726-B07|Canada|658[0n]|BOLD:AAA3764
Dolichogenidea ensiger|[826]|PCPP10-0466|Canada|658[0n]|BOLD:AAA3764
Dolichogenidea ensiger|[827]|BIOUG00991-B03|Canada|658[0n]|BOLD:AAA3764
Dolichogenidea ensiger|[828]|BIOUG00761-A09|Canada|658[0n]|BOLD:AAA3764
Dolichogenidea ensiger|[829]|BIOUG01631-G11|Canada|658[0n]|BOLD:AAA3764
Dolichogenidea ensiger|[830]|BIOUG01597-F04|Canada|658[0n]|BOLD:AAA3764
Dolichogenidea ensiger|[831]|CNC469251|Canada|658[0n]|BOLD:AAA3764
Dolichogenidea ensiger|[832]|CNC469241|Canada|658[0n]|BOLD:AAA3764
Dolichogenidea ensiger|[833]|CNC469341|Canada|658[0n]|BOLD:AAA3764
Dolichogenidea ensiger|[834]|CNC469291|Canada|658[0n]|BOLD:AAA3764
Dolichogenidea ensiger|[835]|CNC469662|Canada|658[0n]|BOLD:AAA3764
Dolichogenidea ensiger|[836]|CNC469654|Canada|658[0n]|BOLD:AAA3764
Dolichogenidea ensiger|[837]|CNC509456|Canada|658[0n]|BOLD:AAA3764
Dolichogenidea ensiger|[838]|CNC469681|Canada|658[0n]|BOLD:AAA3764
Dolichogenidea ensiger|[839]|BIOUG33048-G11|Canada|658[0n]|BOLD:AAA3764
Dolichogenidea ensiger|[840]|CNC509460|Canada|658[0n]|BOLD:AAA3764
Dolichogenidea ensiger|[841]|BIOUG33773-A02|Canada|658[0n]|BOLD:AAA3764
Dolichogenidea ensiger|[842]|BIOUG33769-G06|Canada|658[0n]|BOLD:AAA3764
Microgastrinae|[843]|CNC1009850|Canada|658[0n]|BOLD:AAA3764
Microgastrinae|[844]|CNC1902535|Canada|658[0n]|BOLD:AAA3764
Dolichogenidea ensiger|[845]|BIOUG34473-E06|Canada|658[0n]|BOLD:AAA3764
Dolichogenidea ensiger|[846]|BIOUG34472-E12|Canada|658[0n]|BOLD:AAA3764
Dolichogenidea ensiger|[847]|BIOUG34528-G09|Canada|658[0n]|BOLD:AAA3764
Dolichogenidea ensiger|[848]|BIOUG34528-G06|Canada|658[0n]|BOLD:AAA3764
Dolichogenidea ensiger|[849]|CNC620367|Canada|658[0n]|BOLD:AAA3764
Dolichogenidea ensiger|[850]|CNC620330|Canada|658[0n]|BOLD:AAA3764
Dolichogenidea ensiger|[851]|CNC620293|Canada|658[0n]|BOLD:AAA3764
Dolichogenidea ensiger|[852]|CNC620289|Canada|658[0n]|BOLD:AAA3764
Dolichogenidea ensiger|[853]|CNC620213|Canada|658[0n]|BOLD:AAA3764
Dolichogenidea ensiger|[854]|CNC620186|Canada|658[0n]|BOLD:AAA3764
Dolichogenidea ensiger|[855]|CNC620159|Canada|658[0n]|BOLD:AAA3764
Dolichogenidea ensiger|[856]|CNC620152|Canada|658[0n]|BOLD:AAA3764
Dolichogenidea ensiger|[857]|BIOUG33773-C03|Canada|658[0n]|BOLD:AAA3764
Dolichogenidea ensiger|[858]|BIOUG33773-C01|Canada|658[0n]|BOLD:AAA3764
Dolichogenidea ensiger|[859]|BIOUG33773-A12|Canada|658[0n]|BOLD:AAA3764
Dolichogenidea ensiger|[860]|BIOUG33773-A06|Canada|658[0n]|BOLD:AAA3764
Dolichogenidea ensiger|[861]|CNC620149|Canada|658[0n]|BOLD:AAA3764
Dolichogenidea ensiger|[862]|BIOUG35403-A02|Canada|658[0n]|BOLD:AAA3764
Dolichogenidea ensiger|[863]|BIOUG35396-B11|Canada|658[0n]|BOLD:AAA3764
Dolichogenidea ensiger|[864]|BIOUG35351-E03|Canada|658[0n]|BOLD:AAA3764
Dolichogenidea ensiger|[865]|BIOUG35325-A06|Canada|658[0n]|BOLD:AAA3764
Dolichogenidea ensiger|[866]|BIOUG35472-H07|Canada|658[0n]|BOLD:AAA3764
Dolichogenidea ensiger|[867]|BIOUG35293-E10|Canada|658[0n]|BOLD:AAA3764
Dolichogenidea ensiger|[868]|BIOUG35044-D07|Canada|658[0n]|BOLD:AAA3764
Dolichogenidea ensiger|[869]|BIOUG35044-D06|Canada|658[0n]|BOLD:AAA3764
Dolichogenidea ensiger|[870]|BIOUG34899-A05|Canada|658[0n]|BOLD:AAA3764
Dolichogenidea ensiger|[871]|BIOUG34296-C10|Canada|658[0n]|BOLD:AAA3764
Dolichogenidea ensiger|[872]|BIOUG35100-D04|Canada|658[0n]|BOLD:AAA3764
Dolichogenidea ensiger|[873]|BIOUG35135-E02|Canada|658[0n]|BOLD:AAA3764
Dolichogenidea ensiger|[874]|BIOUG35134-F05|Canada|658[0n]|BOLD:AAA3764
Dolichogenidea ensiger|[875]|BIOUG33769-B07|Canada|658[0n]|BOLD:AAA3764
Dolichogenidea ensiger|[876]|BIOUG33769-B04|Canada|658[0n]|BOLD:AAA3764
Dolichogenidea ensiger|[877]|BIOUG34908-C11|Canada|658[0n]|BOLD:AAA3764
Dolichogenidea ensiger|[878]|BIOUG35016-H10|Canada|658[0n]|BOLD:AAA3764
Dolichogenidea ensiger|[879]|BIOUG33401-C08|Canada|658[0n]|BOLD:AAA3764
Dolichogenidea ensiger|[880]|BIOUG33682-D11|Canada|658[0n]|BOLD:AAA3764
Dolichogenidea ensiger|[881]|BIOUG33641-C04|Canada|658[0n]|BOLD:AAA3764
Dolichogenidea ensiger|[882]|BIOUG33607-H02|Canada|658[0n]|BOLD:AAA3764
Dolichogenidea ensiger|[883]|BIOUG35016-G11|Canada|658[0n]|BOLD:AAA3764
Dolichogenidea ensiger|[884]|BIOUG35016-D05|Canada|658[0n]|BOLD:AAA3764

Dolichogenidea ensiger|[882]|BIOUG33607-H02|Canada|658[0n]|BOLD:AAA3764
Dolichogenidea ensiger|[883]|BIOUG35016-G11|Canada|658[0n]|BOLD:AAA3764
Dolichogenidea ensiger|[884]|BIOUG35016-D05|Canada|658[0n]|BOLD:AAA3764
Dolichogenidea ensiger|[885]|BIOUG35014-C03|Canada|658[0n]|BOLD:AAA3764
Dolichogenidea ensiger|[886]|BIOUG34922-D05|Canada|658[0n]|BOLD:AAA3764
Dolichogenidea ensiger|[887]|BIOUG34671-F09|Canada|658[0n]|BOLD:AAA3764
Dolichogenidea ensiger|[888]|BIOUG34671-E11|Canada|658[0n]|BOLD:AAA3764
Dolichogenidea ensiger|[889]|BIOUG34671-E10|Canada|658[0n]|BOLD:AAA3764
Dolichogenidea ensiger|[890]|BIOUG34671-E06|Canada|658[0n]|BOLD:AAA3764
Dolichogenidea ensiger|[891]|BIOUG34671-C09|Canada|658[0n]|BOLD:AAA3764
Dolichogenidea ensiger|[892]|BIOUG34474-G03|Canada|658[0n]|BOLD:AAA3764
Dolichogenidea ensiger|[893]|BIOUG33917-H09|Canada|658[0n]|BOLD:AAA3764
Dolichogenidea ensiger|[894]|BIOUG33917-A12|Canada|658[0n]|BOLD:AAA3764
Dolichogenidea ensiger|[895]|BIOUG33913-E12|Canada|658[0n]|BOLD:AAA3764
Dolichogenidea ensiger|[896]|BIOUG33773-H08|Canada|658[0n]|BOLD:AAA3764
Dolichogenidea ensiger|[897]|BIOUG33773-G08|Canada|658[0n]|BOLD:AAA3764
Dolichogenidea ensiger|[898]|BIOUG33773-F11|Canada|658[0n]|BOLD:AAA3764
Dolichogenidea ensiger|[899]|BIOUG33773-E08|Canada|658[0n]|BOLD:AAA3764
Dolichogenidea ensiger|[900]|BIOUG33773-C10|Canada|658[0n]|BOLD:AAA3764
Dolichogenidea ensiger|[901]|07PROBE-23393|Canada|657[0n]|BOLD:AAA3764
Dolichogenidea ensiger|[902]|CAM0009|Canada|657[0n]|BOLD:AAA3764
Dolichogenidea ensiger|[903]|CAM0022|Canada|657[0n]|BOLD:AAA3764
Dolichogenidea ensiger|[904]|CAM0024|Canada|657[0n]|BOLD:AAA3764
Dolichogenidea ensiger|[905]|CAM0074|Canada|657[0n]|BOLD:AAA3764
Dolichogenidea ensiger|[906]|CAM0114|Canada|657[0n]|BOLD:AAA3764
Dolichogenidea ensiger|[907]|CAM0286|Canada|657[0n]|BOLD:AAA3764
Dolichogenidea ensiger|[908]|CAM0154|Canada|657[0n]|BOLD:AAA3764
Dolichogenidea ensiger|[909]|CAM0289|Canada|657[0n]|BOLD:AAA3764
Dolichogenidea ensiger|[910]|CAM0288|Canada|657[0n]|BOLD:AAA3764
Dolichogenidea ensiger|[911]|CAM0448|Canada|657[0n]|BOLD:AAA3764
Dolichogenidea ensiger|[912]|CAM0290|Canada|657[0n]|BOLD:AAA3764
Dolichogenidea ensiger|[913]|MIC 000910|Canada|657[0n]|BOLD:AAA3764
Dolichogenidea ensiger|[914]|MIC 000637|Canada|657[0n]|BOLD:AAA3764
Dolichogenidea ensiger|[915]|CAM0522|Canada|657[0n]|BOLD:AAA3764
Dolichogenidea ensiger|[916]|CAM0521|Canada|657[0n]|BOLD:AAA3764
Dolichogenidea ensiger|[917]|CAM0526|Canada|657[0n]|BOLD:AAA3764
Dolichogenidea ensiger|[918]|CAM0523|Canada|657[0n]|BOLD:AAA3764
Dolichogenidea ensiger|[919]|CAM0529|Canada|657[0n]|BOLD:AAA3764
Dolichogenidea ensiger|[920]|CAM0528|Canada|657[0n]|BOLD:AAA3764
Dolichogenidea ensiger|[921]|CAM0531|Canada|657[0n]|BOLD:AAA3764
Dolichogenidea ensiger|[922]|BIOUG35351-B06|Canada|657[1n]|BOLD:AAA3764
Dolichogenidea ensiger|[923]|BIOUG33769-C12|Canada|658[0n]|BOLD:AAA3764
Dolichogenidea ensiger|[924]|07PROBE-22328|Canada|654[0n]|BOLD:AAA3764
Dolichogenidea ensiger|[925]|ASGLE-0880|Canada|658[0n]|BOLD:AAA3764
Dolichogenidea ensiger|[926]|BIOUG33048-F08|Canada|658[0n]|BOLD:AAA3764
Dolichogenidea ensiger|[927]|BIOUG33917-G06|Canada|658[0n]|BOLD:AAA3764
Dolichogenidea ensiger|[928]|BIOUG24493-H08|Canada|600[0n]|BOLD:AAA3764
Dolichogenidea ensiger|[929]|BIOUG08752-A08|Canada|594[0n]|BOLD:AAA3764
Dolichogenidea ensiger|[930]|BIOUG09765-A06|Canada|585[0n]|BOLD:AAA3764
Dolichogenidea ensiger|[931]|BIOUG21791-A11|Canada|582[0n]|BOLD:AAA3764
Dolichogenidea ensiger|[932]|BIOUG07902-D06|Canada|579[0n]|BOLD:AAA3764
Dolichogenidea ensiger|[933]|BIOUG21126-G04|Canada|579[0n]|BOLD:AAA3764
Dolichogenidea ensiger|[934]|BIOUG21138-G08|Canada|576[0n]|BOLD:AAA3764
Dolichogenidea ensiger|[935]|BIOUG09770-A04|Canada|570[0n]|BOLD:AAA3764
Dolichogenidea ensiger|[936]|BIOUG11331-F04|Canada|567[0n]|BOLD:AAA3764
Dolichogenidea ensiger|[937]|BIOUG07902-D08|Canada|564[0n]|BOLD:AAA3764
Dolichogenidea ensiger|[938]|BIOUG21837-B04|Canada|552[0n]|BOLD:AAA3764
Dolichogenidea ensiger|[939]|BIOUG20971-G10|Canada|543[0n]|BOLD:AAA3764
Dolichogenidea ensiger|[940]|BIOUG20975-B05|Canada|543[0n]|BOLD:AAA3764
Dolichogenidea ensiger|[941]|BIOUG21791-C01|Canada|543[0n]|BOLD:AAA3764
Dolichogenidea ensiger|[942]|BIOUG21791-D03|Canada|540[0n]|BOLD:AAA3764
Dolichogenidea ensiger|[943]|BIOUG20972-E11|Canada|540[0n]|BOLD:AAA3764
Dolichogenidea ensiger|[944]|BIOUG20970-D11|Canada|537[3n]|BOLD:AAA3764
Dolichogenidea ensiger|[945]|BIOUG21213-A01|Canada|531[1n]|BOLD:AAA3764
Dolichogenidea ensiger|[946]|BIOUG33641-G07|Canada|658[0n]|BOLD:AAA3764
Dolichogenidea ensiger|[947]|CNCHYM 00106|United States|407[1n]|BOLD:AAA3764
Dolichogenidea ensiger|[948]|BIOUG33682-F06|Canada|658[0n]|BOLD:AAA3764
Dolichogenidea ensiger|[949]|BIOUG01657-F02|Canada|658[0n]|BOLD:AAA3764
Dolichogenidea ensiger|[950]|BIOUG01657-E06|Canada|658[0n]|BOLD:AAA3764
Dolichogenidea ensiger|[951]|BIOUG01661-E11|Canada|658[0n]|BOLD:AAA3764
Dolichogenidea ensiger|[952]|BIOUG01657-F10|Canada|658[0n]|BOLD:AAA3764
Dolichogenidea ensiger|[953]|BIOUG33048-F03|Canada|658[0n]|BOLD:AAA3764
Dolichogenidea ensiger|[954]|BIOUG01631-D07|Canada|658[0n]|BOLD:AAA3764
Dolichogenidea ensiger|[955]|BIOUG33824-E03|Canada|658[0n]|BOLD:AAA3764
Dolichogenidea ensiger|[956]|BIOUG33641-D12|Canada|658[0n]|BOLD:AAA3764
Dolichogenidea ensiger|[957]|BIOUG01631-A09|Canada|658[0n]|BOLD:AAA3764
Dolichogenidea ensiger|[958]|BIOUG01631-A05|Canada|658[0n]|BOLD:AAA3764
Dolichogenidea ensiger|[959]|BIOUG33769-D02|Canada|658[0n]|BOLD:AAA3764
Dolichogenidea ensiger|[960]|BIOUG33769-A06|Canada|658[0n]|BOLD:AAA3764
Dolichogenidea ensiger|[961]|BIOUG33641-A04|Canada|658[0n]|BOLD:AAA3764
Dolichogenidea ensiger|[962]|BIOUG33247-E05|Canada|658[0n]|BOLD:AAA3764
Dolichogenidea ensiger|[963]|BIOUG01657-C11|Canada|658[0n]|BOLD:AAA3764
Dolichogenidea ensiger|[964]|BIOUG33773-G09|Canada|658[0n]|BOLD:AAA3764
Dolichogenidea ensiger|[965]|BIOUG33913-G04|Canada|658[0n]|BOLD:AAA3764
Dolichogenidea ensiger|[966]|BIOUG33917-B08|Canada|658[0n]|BOLD:AAA3764
Dolichogenidea ensiger|[967]|BIOUG34153-B05|Canada|658[0n]|BOLD:AAA3764
Dolichogenidea ensiger|[968]|BIOUG34146-H01|Canada|658[0n]|BOLD:AAA3764
Dolichogenidea ensiger|[969]|BIOUG34151-C03|Canada|658[0n]|BOLD:AAA3764
Dolichogenidea ensiger|[970]|BIOUG34474-H01|Canada|658[0n]|BOLD:AAA3764
Dolichogenidea ensiger|[971]|BIOUG34671-E09|Canada|658[0n]|BOLD:AAA3764
Dolichogenidea ensiger|[972]|BIOUG34922-C09|Canada|658[0n]|BOLD:AAA3764
Dolichogenidea ensiger|[973]|CNC620193|Canada|658[0n]|BOLD:AAA3764
Dolichogenidea ensiger|[974]|BIOUG21126-E05|Canada|540[4n]|BOLD:AAA3764
Dolichogenidea ensiger|[975]|BIOUG21765-H05|Canada|546[0n]|BOLD:AAA3764
Dolichogenidea ensiger|[976]|BIOUG21211-H09|Canada|558[0n]|BOLD:AAA3764
Dolichogenidea ensiger|[977]|BIOUG08749-G01|Canada|570[0n]|BOLD:AAA3764
Dolichogenidea ensiger|[978]|BIOUG07902-D03|Canada|576[0n]|BOLD:AAA3764
Dolichogenidea ensiger|[979]|BIOUG20971-G03|Canada|585[0n]|BOLD:AAA3764
Dolichogenidea ensiger|[980]|BIOUG06549-D07|Canada|589[0n]|BOLD:AAA3764
Dolichogenidea ensiger|[981]|BIOUG09765-B03|Canada|594[0n]|BOLD:AAA3764
Dolichogenidea ensiger|[982]|BIOUG16029-H04|Canada|594[0n]|BOLD:AAA3764

Dolichogenidea ensiger|[980]|BIOUG06549-D07|Canada|589[0n]|BOLD:AAA3764
Dolichogenidea ensiger|[981]|BIOUG09765-B03|Canada|594[0n]|BOLD:AAA3764
Dolichogenidea ensiger|[982]|BIOUG16029-H04|Canada|594[0n]|BOLD:AAA3764
Dolichogenidea ensiger|[983]|BIOUG20972-F06|Canada|594[0n]|BOLD:AAA3764
Dolichogenidea ensiger|[984]|BIOUG20973-G10|Canada|594[0n]|BOLD:AAA3764
Dolichogenidea ensiger|[985]|BIOUG21209-A05|Canada|594[0n]|BOLD:AAA3764
Dolichogenidea ensiger|[986]|BIOUG09765-A12|Canada|600[0n]|BOLD:AAA3764
Dolichogenidea ensiger|[987]|BIOUG20971-A02|Canada|600[0n]|BOLD:AAA3764
Dolichogenidea ensiger|[988]|CAM0447|Canada|657[0n]|BOLD:AAA3764
Dolichogenidea ensiger|[989]|CAM0025|Canada|657[0n]|BOLD:AAA3764
Dolichogenidea ensiger|[990]|CAM0445|Canada|657[0n]|BOLD:AAA3764
Dolichogenidea ensiger|[991]|CAM0530|Canada|657[0n]|BOLD:AAA3764
Dolichogenidea ensiger|[992]|BIOUG34146-G08|Canada|658[0n]|BOLD:AAA3764
Dolichogenidea ensiger|[993]|BIOUG33927-C09|Canada|658[2n]|BOLD:AAA3764
Dolichogenidea ensiger|[994]|BIOUG25474-E09|Canada|586[0n]|BOLD:AAA3764
Dolichogenidea ensiger|[995]|BIOUG24516-D02|Canada|585[0n]|BOLD:AAA3764
Dolichogenidea ensiger|[996]|BIOUG11758-F08|Canada|546[0n]|BOLD:AAA3764
Dolichogenidea ensiger|[997]|BIOUG20974-B06|Canada|537[3n]|BOLD:AAA3764
Dolichogenidea ensiger|[998]|BIOUG25616-A05|Canada|579[0n]|BOLD:AAA3764
Dolichogenidea ensiger|[999]|BIOUG12497-F12|Canada|585[0n]|BOLD:AAA3764
Dolichogenidea ensiger|[1000]|BIOUG20972-B06|Canada|585[0n]|BOLD:AAA3764
Dolichogenidea ensiger|[1001]|BIOUG20975-E06|Canada|585[0n]|BOLD:AAA3764
Dolichogenidea ensiger|[1002]|BIOUG21793-A01|Canada|585[0n]|BOLD:AAA3764
Dolichogenidea ensiger|[1003]|BIOUG20972-B03|Canada|594[0n]|BOLD:AAA3764
Dolichogenidea ensiger|[1004]|BIOUG21765-H07|Canada|552[0n]|BOLD:AAA3764
Dolichogenidea ensiger|[1005]|BIOUG14107-D02|Canada|561[0n]|BOLD:AAA3764
Dolichogenidea ensiger|[1006]|BIOUG03220-A01|Canada|630[0n]|BOLD:AAA3764
Dolichogenidea ensiger|[1007]|BIOUG03979-G06|Canada|630[0n]|BOLD:AAA3764
Dolichogenidea ensiger|[1008]|BIOUG03979-B04|Canada|628[0n]|BOLD:AAA3764
Dolichogenidea ensiger|[1009]|BIOUG03863-G08|Canada|633[0n]|BOLD:AAA3764
Dolichogenidea ensiger|[1010]|BIOUG04041-A02|Canada|633[0n]|BOLD:AAA3764
Dolichogenidea ensiger|[1011]|CAM0524|Canada|632[0n]|BOLD:AAA3764
Dolichogenidea ensiger|[1012]|BIOUG03979-B03|Canada|632[0n]|BOLD:AAA3764
Dolichogenidea ensiger|[1013]|BIOUG03979-H08|Canada|632[0n]|BOLD:AAA3764
Dolichogenidea ensiger|[1014]|CAM0527|Canada|621[1n]|BOLD:AAA3764
Dolichogenidea ensiger|[1015]|BIOUG33769-F04|Canada|658[0n]|BOLD:AAA3764
Dolichogenidea ensiger|[1016]|BIOUG33607-B03|Canada|658[0n]|BOLD:AAA3764
Dolichogenidea ensiger|[1017]|CNCH1358|United States|538[0n]|BOLD:AAA3764
Dolichogenidea ensiger|[1018]|BIOUG34544-A03|Canada|658[0n]|BOLD:AAA3764
Dolichogenidea ensiger|[1019]|GOU 0303|Canada|407[0n]|BOLD:AAA3764
Dolichogenidea ensiger|[1020]|BIOUG33917-D06|Canada|657[1n]|BOLD:AAA3764
Dolichogenidea ensiger|[1021]|BIOUG33912-E06|Canada|658[0n]|BOLD:AAA3764
Dolichogenidea ensiger|[1022]|BIOUG34472-D06|Canada|658[0n]|BOLD:AAA3764
Dolichogenidea ensiger|[1023]|BIOUG01572-D01|Canada|658[1n]|BOLD:AAA3764
Dolichogenidea ensiger|[1024]|10BBCHY-1305|Canada|658[0n]|BOLD:AAA3764
Dolichogenidea ensiger|[1025]|10BBCHY-1304|Canada|658[0n]|BOLD:AAA3764
Dolichogenidea ensiger|[1026]|BIOUG33048-H04|Canada|658[0n]|BOLD:AAA3764
Dolichogenidea ensiger|[1027]|BIOUG01572-H03|Canada|658[0n]|BOLD:AAA3764
Dolichogenidea ensiger|[1028]|BIOUG33769-A04|Canada|658[0n]|BOLD:AAA3764
Microgastrinae|[1029]|CNC1688122|Canada|658[0n]|BOLD:AAA3764
Dolichogenidea ensiger|[1030]|BIOUG33641-F11|Canada|658[0n]|BOLD:AAA3764
Dolichogenidea ensiger|[1031]|BIOUG33838-F03|Canada|658[0n]|BOLD:AAA3764
Dolichogenidea ensiger|[1032]|BIOUG33913-H01|Canada|658[0n]|BOLD:AAA3764
Dolichogenidea ensiger|[1033]|BIOUG33382-A01|Canada|658[0n]|BOLD:AAA3764
Dolichogenidea ensiger|[1034]|BIOUG34472-B08|Canada|658[0n]|BOLD:AAA3764
Dolichogenidea ensiger|[1035]|BIOUG33912-A01|Canada|658[0n]|BOLD:AAA3764
Dolichogenidea ensiger|[1036]|BIOUG34671-B07|Canada|658[0n]|BOLD:AAA3764
Dolichogenidea ensiger|[1037]|BIOUG33917-D10|Canada|658[0n]|BOLD:AAA3764
Dolichogenidea ensiger|[1038]|BIOUG34814-C06|Canada|658[0n]|BOLD:AAA3764
Dolichogenidea ensiger|[1039]|CNC620349|Canada|658[0n]|BOLD:AAA3764
Dolichogenidea ensiger|[1040]|BIOUG35823-G02|Canada|658[0n]|BOLD:AAA3764
Dolichogenidea ensiger|[1041]|HYM00001003|Canada|657[0n]|BOLD:AAA3764
Dolichogenidea ensiger|[1042]|CAM0335|Canada|657[0n]|BOLD:AAA3764
Dolichogenidea ensiger|[1043]|CAM0348|Canada|657[0n]|BOLD:AAA3764
Dolichogenidea ensiger|[1044]|CAM0446|Canada|657[0n]|BOLD:AAA3764
Dolichogenidea ensiger|[1045]|BIOUG03887-F12|Canada|621[0n]|BOLD:AAA3764
Dolichogenidea ensiger|[1046]|BIOUG03863-E06|Canada|627[0n]|BOLD:AAA3764
Dolichogenidea ensiger|[1047]|BIOUG03979-A07|Canada|628[0n]|BOLD:AAA3764
Dolichogenidea ensiger|[1048]|BIOUG25616-A04|Canada|594[0n]|BOLD:AAA3764
Dolichogenidea ensiger|[1049]|BIOUG06823-D07|Canada|589[0n]|BOLD:AAA3764
Dolichogenidea ensiger|[1050]|BIOUG10202-A04|Canada|591[0n]|BOLD:AAA3764
Dolichogenidea ensiger|[1051]|BIOUG25479-C01|Canada|585[0n]|BOLD:AAA3764
Dolichogenidea ensiger|[1052]|BIOUG20790-A01|Canada|564[0n]|BOLD:AAA3764
Dolichogenidea ensiger|[1053]|BIOUG07903-F12|Canada|552[0n]|BOLD:AAA3764
Dolichogenidea ensiger|[1054]|BIOUG14169-A12|Canada|522[0n]|BOLD:AAA3764
Dolichogenidea ensiger|[1055]|BIOUG21213-D07|Canada|528[2n]|BOLD:AAA3764
Dolichogenidea ensiger|[1056]|BIOUG25752-B12|Canada|588[0n]|BOLD:AAA3764
Dolichogenidea ensiger|[1057]|BIOUG03123-B05|Canada|631[0n]|BOLD:AAA3764
Dolichogenidea ensiger|[1058]|BIOUG09765-A01|Canada|585[0n]|BOLD:AAA3764
Dolichogenidea ensiger|[1059]|BIOUG20972-B04|Canada|585[0n]|BOLD:AAA3764
Dolichogenidea ensiger|[1060]|BIOUG20792-H06|Canada|570[0n]|BOLD:AAA3764
Dolichogenidea ensiger|[1061]|BIOUG21213-G04|Canada|531[0n]|BOLD:AAA3764
Dolichogenidea ensiger|[1062]|BIOUG20970-B02|Canada|579[0n]|BOLD:AAA3764
Dolichogenidea ensiger|[1063]|BIOUG34671-A02|Canada|658[0n]|BOLD:AAA3764
Dolichogenidea ensiger|[1064]|BIOUG21792-E06|Canada|591[0n]|BOLD:AAA3764
Dolichogenidea ensiger|[1065]|BIOUG14170-E05|Canada|546[0n]|BOLD:AAA3764
Dolichogenidea ensiger|[1066]|BIOUG14099-G10|Canada|588[0n]|BOLD:AAA3764
Dolichogenidea ensiger|[1067]|BIOUG14172-G04|Canada|594[0n]|BOLD:AAA3764
Dolichogenidea ensiger|[1068]|CAM0635|Canada|658[0n]|BOLD:AAA3764
Dolichogenidea ensiger|[1069]|BIOUG33682-E05|Canada|658[0n]|BOLD:AAA3764
Dolichogenidea ensiger|[1070]|BIOUG21220-D05|Canada|564[0n]|BOLD:AAA3764
Dolichogenidea ensiger|[1071]|BIOUG20975-E04|Canada|552[0n]|BOLD:AAA3764
Dolichogenidea ensiger|[1072]|BIOUG21791-H04|Canada|561[0n]|BOLD:AAA3764
Dolichogenidea ensiger|[1073]|BIOUG21126-C09|Canada|606[0n]|BOLD:AAA3764
Dolichogenidea ensiger|[1074]|CAM0161|Canada|657[0n]|BOLD:AAA3764
Dolichogenidea ensiger|[1075]|BIOUG33773-E03|Canada|658[0n]|BOLD:AAA3764
Dolichogenidea ensiger|[1076]|CNC620207|Canada|658[0n]|BOLD:AAA3764
Dolichogenidea ensiger|[1077]|CAM0525|Canada|657[0n]|BOLD:AAA3764
Dolichogenidea ensiger|[1078]|BIOUG33641-B02|Canada|657[1n]|BOLD:AAA3764
Dolichogenidea ensiger|[1079]|BIOUG34912-H08|Canada|658[0n]|BOLD:AAA3764
Dolichogenidea ensiger|[1080]|BIOUG34923-B07|Canada|658[0n]|BOLD:AAA3764

Dolichogenidea ensiger|[1078]|BIOUG33641-B02|Canada|657[1n]|BOLD:AAA3764
Dolichogenidea ensiger|[1079]|BIOUG34912-H08|Canada|658[0n]|BOLD:AAA3764
Dolichogenidea ensiger|[1080]|BIOUG34923-B07|Canada|658[0n]|BOLD:AAA3764
Dolichogenidea ensiger|[1081]|10BBHYM-1225|United States|649[0n]|BOLD:AAA3764
Dolichogenidea ensiger|[1082]|BBP-60|United States|658[0n]|BOLD:AAA3764
Apanteles juliodiazi|[1083]|ON101953|Costa Rica|701[0n]|BOLD:AAA3764
Dolichogenidea ensiger|[1084]|BIOUG07004-C04|Canada|546[0n]|BOLD:AAA3764
Dolichogenidea ensiger|[1085]|BIOUG06823-E10|Canada|580[0n]|BOLD:AAA3764
Dolichogenidea ensiger|[1086]|BIOUG12486-D10|Canada|579[0n]|BOLD:AAA3764
Dolichogenidea ensiger|[1087]|BIOUG21842-C11|Canada|579[0n]|BOLD:AAA3764
Dolichogenidea ensiger|[1088]|BIOUG12977-D01|Canada|579[0n]|BOLD:AAA3764
Dolichogenidea ensiger|[1089]|BIOUG20970-C10|Canada|537[4n]|BOLD:AAA3764
Dolichogenidea ensiger|[1090]|BIOUG20972-C12|Canada|537[4n]|BOLD:AAA3764
Dolichogenidea ensiger|[1091]|BIOUG12974-A03|Canada|564[0n]|BOLD:AAA3764
Dolichogenidea ensiger|[1092]|BIOUG21791-E06|Canada|564[0n]|BOLD:AAA3764
Dolichogenidea ensiger|[1093]|BIOUG21126-H09|Canada|564[0n]|BOLD:AAA3764
Dolichogenidea ensiger|[1094]|BIOUG12736-B05|Canada|564[0n]|BOLD:AAA3764
Dolichogenidea ensiger|[1095]|BIOUG14108-F04|Canada|576[0n]|BOLD:AAA3764
Dolichogenidea ensiger|[1096]|BIOUG21130-F08|Canada|576[0n]|BOLD:AAA3764
Dolichogenidea ensiger|[1097]|BIOUG14261-G11|Canada|555[0n]|BOLD:AAA3764
Dolichogenidea ensiger|[1098]|BIOUG06823-E08|Canada|589[0n]|BOLD:AAA3764
Dolichogenidea ensiger|[1099]|BIOUG11625-E04|Canada|589[0n]|BOLD:AAA3764
Dolichogenidea ensiger|[1100]|BIOUG12460-D06|Canada|588[0n]|BOLD:AAA3764
Dolichogenidea ensiger|[1101]|BIOUG06823-E06|Canada|589[0n]|BOLD:AAA3764
Dolichogenidea ensiger|[1102]|BIOUG20791-G09|Canada|594[0n]|BOLD:AAA3764
Dolichogenidea ensiger|[1103]|BIOUG14171-B01|Canada|595[0n]|BOLD:AAA3764
Dolichogenidea ensiger|[1104]|BIOUG12487-D03|Canada|594[0n]|BOLD:AAA3764
Dolichogenidea ensiger|[1105]|BIOUG14172-C09|Canada|594[0n]|BOLD:AAA3764
Dolichogenidea ensiger|[1106]|BIOUG20973-C01|Canada|594[0n]|BOLD:AAA3764
Dolichogenidea ensiger|[1107]|BIOUG11771-G06|Canada|594[0n]|BOLD:AAA3764
Dolichogenidea ensiger|[1108]|BIOUG21221-G04|Canada|594[0n]|BOLD:AAA3764
Dolichogenidea ensiger|[1109]|BIOUG20971-F08|Canada|594[0n]|BOLD:AAA3764
Dolichogenidea ensiger|[1110]|BIOUG21837-B07|Canada|594[0n]|BOLD:AAA3764
Dolichogenidea ensiger|[1111]|BIOUG20973-D02|Canada|594[0n]|BOLD:AAA3764
Dolichogenidea ensiger|[1112]|BIOUG07898-C04|Canada|588[0n]|BOLD:AAA3764
Dolichogenidea ensiger|[1113]|BIOUG11726-F01|Canada|588[0n]|BOLD:AAA3764
Dolichogenidea ensiger|[1114]|BIOUG20974-C03|Canada|555[0n]|BOLD:AAA3764
Dolichogenidea ensiger|[1115]|BIOUG12977-D11|Canada|555[0n]|BOLD:AAA3764
Dolichogenidea ensiger|[1116]|BIOUG20970-B09|Canada|558[0n]|BOLD:AAA3764
Dolichogenidea ensiger|[1117]|BIOUG20971-C11|Canada|558[0n]|BOLD:AAA3764
Dolichogenidea ensiger|[1118]|BIOUG20971-F12|Canada|543[0n]|BOLD:AAA3764
Dolichogenidea ensiger|[1119]|BIOUG20974-D06|Canada|558[0n]|BOLD:AAA3764
Dolichogenidea ensiger|[1120]|BIOUG21127-G04|Canada|552[0n]|BOLD:AAA3764
Dolichogenidea ensiger|[1121]|BIOUG11597-E04|Canada|549[0n]|BOLD:AAA3764
Dolichogenidea ensiger|[1122]|BIOUG06823-D09|Canada|561[0n]|BOLD:AAA3764
Dolichogenidea ensiger|[1123]|BIOUG20972-G09|Canada|549[0n]|BOLD:AAA3764
Dolichogenidea ensiger|[1124]|BIOUG20970-E01|Canada|588[0n]|BOLD:AAA3764
Dolichogenidea ensiger|[1125]|BIOUG21791-E09|Canada|552[0n]|BOLD:AAA3764
Dolichogenidea ensiger|[1126]|BIOUG21791-B04|Canada|594[0n]|BOLD:AAA3764
Dolichogenidea ensiger|[1127]|BIOUG17667-C02|Canada|600[0n]|BOLD:AAA3764
Dolichogenidea ensiger|[1128]|BIOUG21130-C02|Canada|600[0n]|BOLD:AAA3764
Dolichogenidea ensiger|[1129]|BIOUG21126-G11|Canada|543[0n]|BOLD:AAA3764
Dolichogenidea ensiger|[1130]|BIOUG11707-A06|Canada|596[0n]|BOLD:AAA3764
Dolichogenidea ensiger|[1131]|BIOUG21307-F05|United States|600[0n]|BOLD:AAA3764
Dolichogenidea ensiger|[1132]|BIOUG20793-B07|Canada|609[5n]|BOLD:AAA3764
Dolichogenidea ensiger|[1133]|BIOUG20971-A09|Canada|588[1n]|BOLD:AAA3764
Dolichogenidea ensiger|[1134]|BIOUG21791-D11|Canada|543[0n]|BOLD:AAA3764
Dolichogenidea ensiger|[1135]|BIOUG20970-B06|Canada|588[0n]|BOLD:AAA3764
Dolichogenidea ensiger|[1136]|BIOUG11771-G09|Canada|582[0n]|BOLD:AAA3764
Dolichogenidea ensiger|[1137]|BIOUG20971-H07|Canada|582[0n]|BOLD:AAA3764
Dolichogenidea ensiger|[1138]|BIOUG11759-F05|Canada|591[0n]|BOLD:AAA3764
Dolichogenidea ensiger|[1139]|BIOUG20628-B04|Canada|588[0n]|BOLD:AAA3764
Dolichogenidea ensiger|[1140]|BIOUG20973-E03|Canada|588[0n]|BOLD:AAA3764
Dolichogenidea ensiger|[1141]|BIOUG20970-B04|Canada|588[0n]|BOLD:AAA3764
Dolichogenidea ensiger|[1142]|BIOUG36377-G05|Canada|594[0n]|BOLD:AAA3764
Dolichogenidea ensiger|[1143]|BIOUG45402-D04|Canada|653[0n]|BOLD:AAA3764
Dolichogenidea ensiger|[1144]|BIOUG39675-E09|Canada|654[0n]|BOLD:AAA3764
Dolichogenidea ensiger|[1145]|BIOUG39678-G05|Canada|655[0n]|BOLD:AAA3764
Dolichogenidea ensiger|[1146]|BIOUG20974-G04|Canada|504[0n]|BOLD:AAA3764
Dolichogenidea ensiger|[1147]|BIOUG11996-F03|Canada|531[0n]|BOLD:AAA3764
Dolichogenidea ensiger|[1148]|BIOUG11999-F03|Canada|525[0n]|BOLD:AAA3764
Dolichogenidea ensiger|[1149]|BIOUG33773-D10|Canada|658[0n]|BOLD:AAA3764
Dolichogenidea ensiger|[1150]|BIOUG21791-F09|Canada|594[0n]|BOLD:AAA3764
Dolichogenidea ensiger|[1151]|BIOUG16166-F07|Canada|564[0n]|BOLD:AAA3764
Dolichogenidea ensiger|[1152]|BIOUG33949-F05|Canada|658[0n]|BOLD:AAA3764
Dolichogenidea ensiger|[1153]|BIOUG34390-D11|Canada|658[0n]|BOLD:AAA3764
Dolichogenidea ensiger|[1154]|BIOUG35380-A02|Canada|658[0n]|BOLD:AAA3764
Dolichogenidea ensiger|[1155]|BIOUG20975-A11|Canada|504[0n]|BOLD:AAA3764
Dolichogenidea Rodriguez144|[1156]|BIOUG74598-D09|Costa Rica|653[0n]|BOLD:AAD2236
Dolichogenidea Rodriguez144|[1157]|BIOUG74598-A06|Costa Rica|651[0n]|BOLD:AAD2236
Dolichogenidea Rodriguez144|[1158]|BIOUG60155-B04|Costa Rica|654[0n]|BOLD:AAD2236
Dolichogenidea Rodriguez144|[1159]|BIOUG54057-E07|Costa Rica|651[0n]|BOLD:AAD2236
Dolichogenidea Rodriguez144|[1160]|BIOUG44129-B02|Costa Rica|654[0n]|BOLD:AAD2236
Dolichogenidea Rodriguez144|[1161]|BIOUG55550-C12|Costa Rica|654[0n]|BOLD:AAD2236
Dolichogenidea Rodriguez144|[1162]|BIOUG57652-B06|Costa Rica|654[0n]|BOLD:AAD2236
Dolichogenidea Rodriguez144|[1163]|BIOUG61847-D11|Costa Rica|654[0n]|BOLD:AAD2236
Dolichogenidea Rodriguez144|[1164]|BIOUG58267-F06|Costa Rica|656[0n]|BOLD:AAD2236
Dolichogenidea Rodriguez144|[1165]|BIOUG59007-E10|Costa Rica|657[0n]|BOLD:AAD2236
Dolichogenidea Rodriguez144|[1166]|BIOUG55902-C05|Costa Rica|654[0n]|BOLD:AAD2236
Dolichogenidea Rodriguez144|[1167]|DHJPAR0013219|Costa Rica|656[1n]|BOLD:AAD2236
Dolichogenidea Rodriguez144|[1168]|DHJPAR0031863|Costa Rica|658[0n]|BOLD:AAD2236
Dolichogenidea Rodriguez144|[1169]|BIOUG57274-B06|Costa Rica|658[0n]|BOLD:AAD2236
Dolichogenidea Rodriguez144|[1170]|DHJPAR0031758|Costa Rica|658[0n]|BOLD:AAD2236
Dolichogenidea Rodriguez144|[1171]|DHJPAR0031810|Costa Rica|658[0n]|BOLD:AAD2236
Dolichogenidea Rodriguez144|[1172]|DHJPAR0031844|Costa Rica|658[0n]|BOLD:AAD2236
Dolichogenidea Rodriguez144|[1173]|DHJPAR0031862|Costa Rica|658[0n]|BOLD:AAD2236
Dolichogenidea Rodriguez144|[1174]|DHJPAR0012519|Costa Rica|657[0n]|BOLD:AAD2236
Dolichogenidea Rodriguez144|[1175]|DHJPAR0012528|Costa Rica|657[0n]|BOLD:AAD2236
Dolichogenidea Rodriguez144|[1176]|DHJPAR0012539|Costa Rica|657[0n]|BOLD:AAD2236
Dolichogenidea Rodriguez144|[1177]|DHJPAR0012548|Costa Rica|657[0n]|BOLD:AAD2236
Dolichogenidea Rodriguez144|[1178]|DHJPAR0012549|Costa Rica|657[0n]|BOLD:AAD2236
[illegible]

Dolichogenidea Rodriguez144|[1176]|DHJPAR0012539|Costa Rica|657[0n]|BOLD:AAD2236
Dolichogenidea Rodriguez144|[1177]|DHJPAR0012548|Costa Rica|657[0n]|BOLD:AAD2236
Dolichogenidea Rodriguez144|[1178]|DHJPAR0012549|Costa Rica|657[0n]|BOLD:AAD2236
Dolichogenidea Rodriguez144|[1179]|DHJPAR0012555|Costa Rica|657[0n]|BOLD:AAD2236
Dolichogenidea Rodriguez144|[1180]|BIOUG57397-G05|Costa Rica|656[0n]|BOLD:AAD2236
Dolichogenidea Rodriguez144|[1181]|BIOUG57395-D08|Costa Rica|655[0n]|BOLD:AAD2236
Dolichogenidea Rodriguez144|[1182]|BIOUG64072-D04|Costa Rica|641[0n]|BOLD:AAD2236
Dolichogenidea Rodriguez144|[1183]|BIOUG58264-A08|Costa Rica|640[0n]|BOLD:AAD2236
Dolichogenidea Rodriguez144|[1184]|BIOUG55713-D12|Costa Rica|656[0n]|BOLD:AAD2236
Dolichogenidea Rodriguez144|[1185]|BIOUG57366-C07|Costa Rica|656[0n]|BOLD:AAD2236
Dolichogenidea Rodriguez144|[1186]|BIOUG60699-F03|Costa Rica|656[0n]|BOLD:AAD2236
Dolichogenidea Rodriguez144|[1187]|BIOUG60136-D03|Costa Rica|654[0n]|BOLD:AAD2236
Dolichogenidea Rodriguez144|[1188]|BIOUG63016-B12|Costa Rica|654[0n]|BOLD:AAD2236
Dolichogenidea Rodriguez144|[1189]|BIOUG57237-D01|Costa Rica|655[0n]|BOLD:AAD2236
Dolichogenidea Rodriguez144|[1190]|BIOUG57276-C03|Costa Rica|655[0n]|BOLD:AAD2236
Dolichogenidea Rodriguez144|[1191]|BIOUG57395-A05|Costa Rica|655[0n]|BOLD:AAD2236
Dolichogenidea Rodriguez144|[1192]|BIOUG58914-E07|Costa Rica|655[0n]|BOLD:AAD2236
Dolichogenidea Rodriguez144|[1193]|BIOUG61370-A09|Costa Rica|655[0n]|BOLD:AAD2236
Dolichogenidea Rodriguez144|[1194]|BIOUG64176-A02|Costa Rica|655[0n]|BOLD:AAD2236
Dolichogenidea Rodriguez144|[1195]|BIOUG57354-H05|Costa Rica|655[0n]|BOLD:AAD2236
Dolichogenidea Rodriguez144|[1196]|BIOUG57368-B11|Costa Rica|655[0n]|BOLD:AAD2236
Dolichogenidea Rodriguez144|[1197]|BIOUG55716-G08|Costa Rica|655[0n]|BOLD:AAD2236
Dolichogenidea Rodriguez144|[1198]|BIOUG55744-B10|Costa Rica|655[0n]|BOLD:AAD2236
Dolichogenidea Rodriguez144|[1199]|BIOUG58291-H08|Costa Rica|654[0n]|BOLD:AAD2236
Dolichogenidea Rodriguez144|[1200]|BIOUG57911-G09|Costa Rica|654[0n]|BOLD:AAD2236
Dolichogenidea Rodriguez144|[1201]|BIOUG58267-F09|Costa Rica|654[0n]|BOLD:AAD2236
Dolichogenidea Rodriguez144|[1202]|BIOUG56761-E01|Costa Rica|654[0n]|BOLD:AAD2236
Dolichogenidea Rodriguez144|[1203]|BIOUG55881-C02|Costa Rica|655[0n]|BOLD:AAD2236
Dolichogenidea Rodriguez144|[1204]|BIOUG55681-C10|Costa Rica|655[0n]|BOLD:AAD2236
Dolichogenidea Rodriguez144|[1205]|BIOUG60135-H09|Costa Rica|655[0n]|BOLD:AAD2236
Dolichogenidea Rodriguez144|[1206]|BIOUG60136-D05|Costa Rica|655[0n]|BOLD:AAD2236
Dolichogenidea Rodriguez144|[1207]|BIOUG55717-C05|Costa Rica|656[0n]|BOLD:AAD2236
Dolichogenidea Rodriguez144|[1208]|BIOUG57253-G04|Costa Rica|656[0n]|BOLD:AAD2236
Dolichogenidea Rodriguez144|[1209]|BIOUG57276-E11|Costa Rica|656[0n]|BOLD:AAD2236
Dolichogenidea Rodriguez144|[1210]|BIOUG57355-B06|Costa Rica|656[0n]|BOLD:AAD2236
Dolichogenidea Rodriguez144|[1211]|BIOUG58618-A06|Costa Rica|654[0n]|BOLD:AAD2236
Dolichogenidea Rodriguez144|[1212]|BIOUG44619-A05|Costa Rica|654[0n]|BOLD:AAD2236
Dolichogenidea Rodriguez144|[1213]|BIOUG54373-A09|Costa Rica|654[0n]|BOLD:AAD2236
Dolichogenidea Rodriguez144|[1214]|BIOUG55093-E08|Costa Rica|654[0n]|BOLD:AAD2236
Dolichogenidea Rodriguez144|[1215]|BIOUG58817-F01|Costa Rica|654[0n]|BOLD:AAD2236
Dolichogenidea Rodriguez144|[1216]|BIOUG57400-C07|Costa Rica|654[0n]|BOLD:AAD2236
Dolichogenidea Rodriguez144|[1217]|BIOUG55707-A03|Costa Rica|654[0n]|BOLD:AAD2236
Dolichogenidea Rodriguez144|[1218]|BIOUG55717-A07|Costa Rica|654[0n]|BOLD:AAD2236
Dolichogenidea Rodriguez144|[1219]|BIOUG55748-A08|Costa Rica|654[0n]|BOLD:AAD2236
Dolichogenidea Rodriguez144|[1220]|BIOUG57237-G09|Costa Rica|654[0n]|BOLD:AAD2236
Dolichogenidea Rodriguez144|[1221]|BIOUG55771-B04|Costa Rica|654[0n]|BOLD:AAD2236
Dolichogenidea Rodriguez144|[1222]|BIOUG57957-H11|Costa Rica|654[0n]|BOLD:AAD2236
Dolichogenidea Rodriguez144|[1223]|BIOUG57958-H01|Costa Rica|654[0n]|BOLD:AAD2236
Dolichogenidea Rodriguez144|[1224]|BIOUG57959-F07|Costa Rica|654[0n]|BOLD:AAD2236
Dolichogenidea Rodriguez144|[1225]|BIOUG56081-A10|Costa Rica|654[0n]|BOLD:AAD2236
Dolichogenidea Rodriguez144|[1226]|BIOUG55697-H06|Costa Rica|654[0n]|BOLD:AAD2236
Dolichogenidea Rodriguez144|[1227]|BIOUG56307-D01|Costa Rica|654[0n]|BOLD:AAD2236
Dolichogenidea Rodriguez144|[1228]|BIOUG56438-G09|Costa Rica|654[0n]|BOLD:AAD2236
Dolichogenidea Rodriguez144|[1229]|BIOUG55890-E03|Costa Rica|654[0n]|BOLD:AAD2236
Dolichogenidea Rodriguez144|[1230]|BIOUG54430-H05|Costa Rica|654[0n]|BOLD:AAD2236
Dolichogenidea Rodriguez144|[1231]|BIOUG57274-B07|Costa Rica|654[0n]|BOLD:AAD2236
Dolichogenidea Rodriguez144|[1232]|BIOUG55884-A10|Costa Rica|654[0n]|BOLD:AAD2236
Dolichogenidea Rodriguez144|[1233]|BIOUG56763-E10|Costa Rica|654[0n]|BOLD:AAD2236
Dolichogenidea Rodriguez144|[1234]|BIOUG57276-G06|Costa Rica|654[0n]|BOLD:AAD2236
Dolichogenidea Rodriguez144|[1235]|BIOUG56761-D09|Costa Rica|654[0n]|BOLD:AAD2236
Dolichogenidea Rodriguez144|[1236]|BIOUG57256-A12|Costa Rica|654[0n]|BOLD:AAD2236
Dolichogenidea Rodriguez144|[1237]|BIOUG43806-B09|Costa Rica|653[0n]|BOLD:AAD2236
Dolichogenidea Rodriguez144|[1238]|BIOUG58268-E09|Costa Rica|654[0n]|BOLD:AAD2236
Dolichogenidea Rodriguez144|[1239]|BIOUG56256-H07|Costa Rica|653[0n]|BOLD:AAD2236
Dolichogenidea Rodriguez144|[1240]|BIOUG46647-H04|Costa Rica|653[0n]|BOLD:AAD2236
Dolichogenidea Rodriguez144|[1241]|BIOUG57915-E07|Costa Rica|653[0n]|BOLD:AAD2236
Dolichogenidea Rodriguez144|[1242]|BIOUG57274-F12|Costa Rica|653[0n]|BOLD:AAD2236
Dolichogenidea Rodriguez144|[1243]|BIOUG64963-F11|Costa Rica|653[0n]|BOLD:AAD2236
Dolichogenidea Rodriguez144|[1244]|BIOUG57914-D07|Costa Rica|653[0n]|BOLD:AAD2236
Dolichogenidea Rodriguez144|[1245]|BIOUG57959-E07|Costa Rica|653[0n]|BOLD:AAD2236
Dolichogenidea Rodriguez144|[1246]|BIOUG57599-B12|Costa Rica|653[0n]|BOLD:AAD2236
Dolichogenidea Rodriguez144|[1247]|BIOUG63633-A04|Costa Rica|653[0n]|BOLD:AAD2236
Dolichogenidea Rodriguez144|[1248]|BIOUG61846-D09|Costa Rica|653[0n]|BOLD:AAD2236
Dolichogenidea Rodriguez144|[1249]|BIOUG57344-C10|Costa Rica|653[0n]|BOLD:AAD2236
Dolichogenidea Rodriguez144|[1250]|BIOUG57344-A11|Costa Rica|653[0n]|BOLD:AAD2236
Dolichogenidea Rodriguez144|[1251]|BIOUG57344-C03|Costa Rica|652[0n]|BOLD:AAD2236
Dolichogenidea Rodriguez144|[1252]|BIOUG63524-E07|Costa Rica|652[0n]|BOLD:AAD2236
Dolichogenidea Rodriguez144|[1253]|BIOUG58965-C07|Costa Rica|651[0n]|BOLD:AAD2236
Dolichogenidea Rodriguez144|[1254]|BIOUG58640-H07|Costa Rica|651[0n]|BOLD:AAD2236
Dolichogenidea Rodriguez144|[1255]|BIOUG64115-B12|Costa Rica|651[0n]|BOLD:AAD2236
Dolichogenidea Rodriguez144|[1256]|BIOUG53287-H09|Costa Rica|652[0n]|BOLD:AAD2236
Dolichogenidea Rodriguez144|[1257]|BIOUG44676-F07|Costa Rica|654[0n]|BOLD:AAD2236
Dolichogenidea Rodriguez144|[1258]|BIOUG74828-D05|Costa Rica|654[0n]|BOLD:AAD2236
Dolichogenidea Rodriguez144|[1259]|BIOUG60167-H08|Costa Rica|655[0n]|BOLD:AAD2236
Dolichogenidea Rodriguez144|[1260]|DHJPAR0024677|Costa Rica|657[0n]|BOLD:AAD2236
Dolichogenidea Rodriguez144|[1261]|BIOUG55743-B03|Costa Rica|657[0n]|BOLD:AAD2236
Dolichogenidea Rodriguez144|[1262]|BIOUG57013-H08|Costa Rica|657[0n]|BOLD:AAD2236
Dolichogenidea Rodriguez144|[1263]|BIOUG57367-C01|Costa Rica|657[0n]|BOLD:AAD2236
Dolichogenidea Rodriguez144|[1264]|DHJPAR0031585|Costa Rica|658[0n]|BOLD:AAD2236
Dolichogenidea Rodriguez144|[1265]|DHJPAR0031684|Costa Rica|658[0n]|BOLD:AAD2236
Dolichogenidea Rodriguez144|[1266]|DHJPAR0031719|Costa Rica|658[0n]|BOLD:AAD2236
Dolichogenidea Rodriguez144|[1267]|BIOUG58268-F01|Costa Rica|658[0n]|BOLD:AAD2236
Dolichogenidea Janzen20|[1268]|DHJPAR0031238|Costa Rica|642[0n]|BOLD:AAI9747
Dolichogenidea Janzen20|[1269]|BIOUG71056-A02|Costa Rica|654[0n]|BOLD:AAI9747
Dolichogenidea Janzen20|[1270]|BIOUG71832-H05|Costa Rica|655[0n]|BOLD:AAI9747
Dolichogenidea Janzen20|[1271]|DHJPAR0012736|Costa Rica|657[0n]|BOLD:AAI9747
Dolichogenidea Janzen20|[1272]|DHJPAR0013483|Costa Rica|657[0n]|BOLD:AAI9747
Dolichogenidea Janzen20|[1273]|DHJPAR0031504|Costa Rica|658[0n]|BOLD:AAI9747
Dolichogenidea Janzen20|[1274]|DHJPAR0031505|Costa Rica|658[0n]|BOLD:AAI9747
Dolichogenidea Janzen20|[1275]|DHJPAR0031495|Costa Rica|658[0n]|BOLD:AAI9747
Dolichogenidea Janzen20|[1276]|DHJPAR0031501|Costa Rica|658[0n]|BOLD:AAI9747
Dolichogenidea Janzen20|[1277]|DHJPAR0031252|Costa Rica|658[0n]|BOLD:AAI9747

Dolichogenidea Janzen20|[1274]|DHJPAR0031[illegible]|Costa Rica|658[0n]|BOLD:AAI9747
Dolichogenidea Janzen20|[1275]|DHJPAR0031495|Costa Rica|658[0n]|BOLD:AAI9747
Dolichogenidea Janzen20|[1276]|DHJPAR0031501|Costa Rica|658[0n]|BOLD:AAI9747
Dolichogenidea Janzen20|[1277]|DHJPAR0031252|Costa Rica|658[0n]|BOLD:AAI9747
Dolichogenidea Janzen20|[1278]|DHJPAR0031255|Costa Rica|658[0n]|BOLD:AAI9747
Dolichogenidea Janzen20|[1279]|DHJPAR0031249|Costa Rica|658[0n]|BOLD:AAI9747
Dolichogenidea Janzen20|[1280]|DHJPAR0031251|Costa Rica|658[0n]|BOLD:AAI9747
Dolichogenidea Janzen20|[1281]|DHJPAR0033945|Costa Rica|658[0n]|BOLD:AAI9747
Dolichogenidea Janzen20|[1282]|DHJPAR0031200|Costa Rica|658[0n]|BOLD:AAI9747
Dolichogenidea Janzen20|[1283]|DHJPAR0031217|Costa Rica|658[0n]|BOLD:AAI9747
Dolichogenidea Janzen20|[1284]|DHJPAR0031239|Costa Rica|658[0n]|BOLD:AAI9747
Dolichogenidea Janzen20|[1285]|DHJPAR0031240|Costa Rica|658[0n]|BOLD:AAI9747
Dolichogenidea Janzen20|[1286]|DHJPAR0031242|Costa Rica|658[0n]|BOLD:AAI9747
Dolichogenidea Janzen20|[1287]|DHJPAR0031248|Costa Rica|658[0n]|BOLD:AAI9747
Dolichogenidea Janzen20|[1288]|DHJPAR0031558|Costa Rica|658[0n]|BOLD:AAI9747
Dolichogenidea Janzen94|[1289]|BIOUG57839-G01|Costa Rica|654[0n]|BOLD:AAE8602
Dolichogenidea Janzen94|[1290]|BIOUG60743-D06|Costa Rica|656[0n]|BOLD:AAE8602
Dolichogenidea Janzen94|[1291]|BIOUG58293-E03|Costa Rica|655[0n]|BOLD:AAE8602
Dolichogenidea Janzen94|[1292]|BIOUG58965-A04|Costa Rica|655[0n]|BOLD:AAE8602
Dolichogenidea Janzen94|[1293]|BIOUG54727-G08|Costa Rica|654[0n]|BOLD:AAE8602
Dolichogenidea Janzen94|[1294]|BIOUG64570-B01|Costa Rica|654[0n]|BOLD:AAE8602
Dolichogenidea Janzen94|[1295]|BIOUG64900-A10|Costa Rica|654[0n]|BOLD:AAE8602
Dolichogenidea Janzen94|[1296]|BIOUG50565-F09|Costa Rica|653[0n]|BOLD:AAE8602
Dolichogenidea Janzen94|[1297]|BIOUG63137-C08|Costa Rica|653[0n]|BOLD:AAE8602
Dolichogenidea Janzen94|[1298]|BIOUG64570-A01|Costa Rica|653[0n]|BOLD:AAE8602
Dolichogenidea Janzen94|[1299]|BIOUG65331-B02|Costa Rica|652[0n]|BOLD:AAE8602
Dolichogenidea Janzen94|[1300]|BIOUG65372-B05|Costa Rica|652[0n]|BOLD:AAE8602
Dolichogenidea Janzen94|[1301]|BIOUG58778-E10|Costa Rica|651[0n]|BOLD:AAE8602
Dolichogenidea Janzen94|[1302]|BIOUG58586-A12|Costa Rica|655[0n]|BOLD:AAE8602
Dolichogenidea Janzen94|[1303]|BIOUG56764-A09|Costa Rica|656[0n]|BOLD:AAE8602
Dolichogenidea Janzen94|[1304]|BIOUG64593-E09|Costa Rica|656[0n]|BOLD:AAE8602
Dolichogenidea Janzen94|[1305]|DHJPAR0012558|Costa Rica|657[0n]|BOLD:AAE8602
Dolichogenidea Janzen94|[1306]|DHJPAR0024730|Costa Rica|657[0n]|BOLD:AAE8602
Dolichogenidea Janzen94|[1307]|DHJPAR0024737|Costa Rica|657[0n]|BOLD:AAE8602
Dolichogenidea Janzen94|[1308]|DHJPAR0024738|Costa Rica|657[0n]|BOLD:AAE8602
Dolichogenidea Janzen57|[1309]|DHJPAR0033972|Costa Rica|658[0n]|BOLD:AAM5842
Dolichogenidea Janzen57|[1310]|DHJPAR0033804|Costa Rica|658[0n]|BOLD:AAM5842
Dolichogenidea Janzen57|[1311]|DHJPAR0033837|Costa Rica|658[0n]|BOLD:AAM5842
Dolichogenidea Janzen57|[1312]|DHJPAR0034039|Costa Rica|658[0n]|BOLD:AAM5842
Dolichogenidea Janzen57|[1313]|DHJPAR0033857|Costa Rica|658[0n]|BOLD:AAM5842
Dolichogenidea Janzen57|[1314]|DHJPAR0034072|Costa Rica|658[0n]|BOLD:AAM5842
Dolichogenidea|[1315]|BIOUG25834-A10|Honduras|564[0n]|BOLD:AAM5842
Dolichogenidea|[1316]|BIOUG18845-E06|Honduras|576[0n]|BOLD:AAM5842
Dolichogenidea|[1317]|BIOUG26888-A01|Honduras|579[0n]|BOLD:AAM5842
Dolichogenidea|[1318]|BIOUG26881-B05|Honduras|600[0n]|BOLD:AAM5842
Dolichogenidea|[1319]|BIOUG28324-G10|Honduras|600[0n]|BOLD:AAM5842
Hymenoptera|[1320]|CBG-A04315-C11|Costa Rica|653[0n]|BOLD:AAM5842
Dolichogenidea Janzen57|[1321]|DHJPAR0033825|Costa Rica|658[0n]|BOLD:AAM5842
Dolichogenidea Janzen57|[1322]|DHJPAR0033991|Costa Rica|658[0n]|BOLD:AAM5842
Dolichogenidea Janzen57|[1323]|DHJPAR0034003|Costa Rica|658[0n]|BOLD:AAM5842
Dolichogenidea Janzen57|[1324]|DHJPAR0033830|Costa Rica|658[0n]|BOLD:AAM5842
Dolichogenidea Janzen57|[1325]|DHJPAR0034005|Costa Rica|658[0n]|BOLD:AAM5842
Dolichogenidea Janzen57|[1326]|DHJPAR0034002|Costa Rica|658[0n]|BOLD:AAM5842
Dolichogenidea Janzen57|[1327]|DHJPAR0031235|Costa Rica|658[0n]|BOLD:AAM5842
Dolichogenidea Janzen57|[1328]|DHJPAR0033836|Costa Rica|658[0n]|BOLD:AAM5842
Dolichogenidea Janzen57|[1329]|DHJPAR0034009|Costa Rica|658[0n]|BOLD:AAM5842
Dolichogenidea Janzen57|[1330]|DHJPAR0034004|Costa Rica|658[0n]|BOLD:AAM5842
Dolichogenidea Janzen57|[1331]|DHJPAR0033839|Costa Rica|658[0n]|BOLD:AAM5842
Dolichogenidea Janzen57|[1332]|DHJPAR0034008|Costa Rica|658[0n]|BOLD:AAM5842
Dolichogenidea Janzen57|[1333]|DHJPAR0031232|Costa Rica|658[0n]|BOLD:AAM5842
Dolichogenidea Janzen57|[1334]|DHJPAR0031234|Costa Rica|658[0n]|BOLD:AAM5842
Dolichogenidea Janzen57|[1335]|DHJPAR0031227|Costa Rica|658[0n]|BOLD:AAM5842
Dolichogenidea Janzen57|[1336]|DHJPAR0031228|Costa Rica|658[0n]|BOLD:AAM5842
Dolichogenidea Janzen57|[1337]|DHJPAR0031229|Costa Rica|658[0n]|BOLD:AAM5842
Dolichogenidea Janzen57|[1338]|DHJPAR0031230|Costa Rica|658[0n]|BOLD:AAM5842
Dolichogenidea Janzen57|[1339]|DHJPAR0031224|Costa Rica|658[0n]|BOLD:AAM5842
Dolichogenidea Janzen57|[1340]|DHJPAR0031225|Costa Rica|658[0n]|BOLD:AAM5842
Dolichogenidea Janzen57|[1341]|DHJPAR0034035|Costa Rica|658[0n]|BOLD:AAM5842
Dolichogenidea Janzen57|[1342]|DHJPAR0033853|Costa Rica|658[0n]|BOLD:AAM5842
Dolichogenidea Janzen57|[1343]|DHJPAR0034033|Costa Rica|658[0n]|BOLD:AAM5842
Dolichogenidea Janzen57|[1344]|DHJPAR0034034|Costa Rica|658[0n]|BOLD:AAM5842
Dolichogenidea Janzen57|[1345]|DHJPAR0031219|Costa Rica|658[0n]|BOLD:AAM5842
Dolichogenidea Janzen57|[1346]|DHJPAR0031221|Costa Rica|658[0n]|BOLD:AAM5842
Dolichogenidea Janzen57|[1347]|DHJPAR0031216|Costa Rica|658[0n]|BOLD:AAM5842
Dolichogenidea Janzen57|[1348]|DHJPAR0031218|Costa Rica|658[0n]|BOLD:AAM5842
Dolichogenidea Janzen57|[1349]|DHJPAR0033883|Costa Rica|658[0n]|BOLD:AAM5842
Dolichogenidea Janzen57|[1350]|DHJPAR0031215|Costa Rica|658[0n]|BOLD:AAM5842
Dolichogenidea Janzen57|[1351]|DHJPAR0033886|Costa Rica|658[0n]|BOLD:AAM5842
Dolichogenidea Janzen57|[1352]|DHJPAR0033882|Costa Rica|658[0n]|BOLD:AAM5842
Dolichogenidea Janzen57|[1353]|DHJPAR0033920|Costa Rica|658[0n]|BOLD:AAM5842
Dolichogenidea Janzen57|[1354]|DHJPAR0033887|Costa Rica|658[0n]|BOLD:AAM5842
Dolichogenidea Janzen57|[1355]|DHJPAR0033851|Costa Rica|658[0n]|BOLD:AAM5842
Dolichogenidea Janzen57|[1356]|DHJPAR0034027|Costa Rica|658[0n]|BOLD:AAM5842
Dolichogenidea Janzen57|[1357]|DHJPAR0034064|Costa Rica|658[0n]|BOLD:AAM5842
Dolichogenidea Janzen57|[1358]|DHJPAR0034066|Costa Rica|658[0n]|BOLD:AAM5842
Dolichogenidea Janzen57|[1359]|DHJPAR0034026|Costa Rica|658[0n]|BOLD:AAM5842
Dolichogenidea Janzen57|[1360]|DHJPAR0033850|Costa Rica|658[0n]|BOLD:AAM5842
Dolichogenidea Janzen57|[1361]|DHJPAR0034062|Costa Rica|658[0n]|BOLD:AAM5842
Dolichogenidea Janzen57|[1362]|DHJPAR0034063|Costa Rica|658[0n]|BOLD:AAM5842
Dolichogenidea Janzen57|[1363]|DHJPAR0033865|Costa Rica|658[0n]|BOLD:AAM5842
Dolichogenidea Janzen57|[1364]|DHJPAR0034060|Costa Rica|658[0n]|BOLD:AAM5842
Dolichogenidea Janzen57|[1365]|DHJPAR0034018|Costa Rica|658[0n]|BOLD:AAM5842
Dolichogenidea Janzen57|[1366]|DHJPAR0034025|Costa Rica|658[0n]|BOLD:AAM5842
Dolichogenidea Janzen57|[1367]|DHJPAR0034024|Costa Rica|658[0n]|BOLD:AAM5842
Dolichogenidea Janzen57|[1368]|DHJPAR0034017|Costa Rica|658[0n]|BOLD:AAM5842
Dolichogenidea Janzen57|[1369]|DHJPAR0034020|Costa Rica|658[0n]|BOLD:AAM5842
Dolichogenidea Janzen57|[1370]|DHJPAR0034021|Costa Rica|658[0n]|BOLD:AAM5842
Dolichogenidea Janzen57|[1371]|DHJPAR0034016|Costa Rica|658[0n]|BOLD:AAM5842
Dolichogenidea Janzen57|[1372]|DHJPAR0034019|Costa Rica|658[0n]|BOLD:AAM5842
Dolichogenidea Janzen57|[1373]|DHJPAR0034059|Costa Rica|658[0n]|BOLD:AAM5842
Dolichogenidea Janzen57|[1374]|DHJPAR0033864|Costa Rica|658[0n]|BOLD:AAM5842
Dolichogenidea Janzen57|[1375]|DHJPAR0034054|Costa Rica|658[0n]|BOLD:AAM5842

[illegible]
Dolichogenidea Janzen57|[1373]|DHJPAR0034059|Costa Rica|658[0n]|BOLD:AAM5842
Dolichogenidea Janzen57|[1374]|DHJPAR0033864|Costa Rica|658[0n]|BOLD:AAM5842
Dolichogenidea Janzen57|[1375]|DHJPAR0034054|Costa Rica|658[0n]|BOLD:AAM5842
Dolichogenidea Janzen57|[1376]|DHJPAR0034055|Costa Rica|658[0n]|BOLD:AAM5842
Dolichogenidea Janzen57|[1377]|DHJPAR0034013|Costa Rica|658[0n]|BOLD:AAM5842
Dolichogenidea Janzen57|[1378]|DHJPAR0034014|Costa Rica|658[0n]|BOLD:AAM5842
Dolichogenidea Janzen57|[1379]|DHJPAR0034011|Costa Rica|658[0n]|BOLD:AAM5842
Dolichogenidea Janzen57|[1380]|DHJPAR0034012|Costa Rica|658[0n]|BOLD:AAM5842
Dolichogenidea Janzen57|[1381]|DHJPAR0034048|Costa Rica|658[0n]|BOLD:AAM5842
Dolichogenidea Janzen57|[1382]|DHJPAR0034053|Costa Rica|658[0n]|BOLD:AAM5842
Dolichogenidea Janzen57|[1383]|DHJPAR0033863|Costa Rica|658[0n]|BOLD:AAM5842
Dolichogenidea Janzen57|[1384]|DHJPAR0034047|Costa Rica|658[0n]|BOLD:AAM5842
Dolichogenidea Janzen57|[1385]|DHJPAR0034007|Costa Rica|658[0n]|BOLD:AAM5842
Dolichogenidea Janzen57|[1386]|DHJPAR0034010|Costa Rica|658[0n]|BOLD:AAM5842
Dolichogenidea Janzen57|[1387]|DHJPAR0033769|Costa Rica|658[0n]|BOLD:AAM5842
Dolichogenidea Janzen57|[1388]|DHJPAR0034006|Costa Rica|658[0n]|BOLD:AAM5842
Dolichogenidea Janzen57|[1389]|DHJPAR0033781|Costa Rica|658[0n]|BOLD:AAM5842
Dolichogenidea Janzen57|[1390]|DHJPAR0033766|Costa Rica|658[0n]|BOLD:AAM5842
Dolichogenidea Janzen57|[1391]|DHJPAR0034044|Costa Rica|658[0n]|BOLD:AAM5842
Dolichogenidea Janzen57|[1392]|DHJPAR0033862|Costa Rica|658[0n]|BOLD:AAM5842
Dolichogenidea Janzen57|[1393]|DHJPAR0033861|Costa Rica|658[0n]|BOLD:AAM5842
Dolichogenidea Janzen57|[1394]|DHJPAR0034042|Costa Rica|658[0n]|BOLD:AAM5842
Dolichogenidea Janzen57|[1395]|DHJPAR0033790|Costa Rica|658[0n]|BOLD:AAM5842
Dolichogenidea Janzen57|[1396]|DHJPAR0033779|Costa Rica|658[0n]|BOLD:AAM5842
Dolichogenidea Janzen57|[1397]|DHJPAR0033796|Costa Rica|658[0n]|BOLD:AAM5842
Dolichogenidea Janzen57|[1398]|DHJPAR0033791|Costa Rica|658[0n]|BOLD:AAM5842
Dolichogenidea Janzen57|[1399]|DHJPAR0034041|Costa Rica|658[0n]|BOLD:AAM5842
Dolichogenidea Janzen57|[1400]|DHJPAR0033856|Costa Rica|658[0n]|BOLD:AAM5842
Dolichogenidea Janzen57|[1401]|DHJPAR0033798|Costa Rica|658[0n]|BOLD:AAM5842
Dolichogenidea Janzen57|[1402]|DHJPAR0033795|Costa Rica|658[0n]|BOLD:AAM5842
Dolichogenidea Janzen57|[1403]|DHJPAR0033855|Costa Rica|658[0n]|BOLD:AAM5842
Dolichogenidea Janzen57|[1404]|DHJPAR0034037|Costa Rica|658[0n]|BOLD:AAM5842
Dolichogenidea Janzen57|[1405]|DHJPAR0034031|Costa Rica|658[0n]|BOLD:AAM5842
Dolichogenidea Janzen57|[1406]|DHJPAR0034032|Costa Rica|658[0n]|BOLD:AAM5842
Dolichogenidea Janzen57|[1407]|DHJPAR0033794|Costa Rica|658[0n]|BOLD:AAM5842
Dolichogenidea Janzen57|[1408]|DHJPAR0033782|Costa Rica|658[0n]|BOLD:AAM5842
Dolichogenidea Janzen57|[1409]|DHJPAR0033805|Costa Rica|658[0n]|BOLD:AAM5842
Dolichogenidea Janzen57|[1410]|DHJPAR0033799|Costa Rica|658[0n]|BOLD:AAM5842
Dolichogenidea Janzen57|[1411]|DHJPAR0033809|Costa Rica|658[0n]|BOLD:AAM5842
Dolichogenidea Janzen57|[1412]|DHJPAR0033802|Costa Rica|658[0n]|BOLD:AAM5842
Dolichogenidea Janzen57|[1413]|DHJPAR0033813|Costa Rica|658[0n]|BOLD:AAM5842
Dolichogenidea Janzen57|[1414]|DHJPAR0033808|Costa Rica|658[0n]|BOLD:AAM5842
Dolichogenidea Janzen57|[1415]|DHJPAR0033801|Costa Rica|658[0n]|BOLD:AAM5842
Dolichogenidea Janzen57|[1416]|DHJPAR0033797|Costa Rica|658[0n]|BOLD:AAM5842
Dolichogenidea Janzen57|[1417]|DHJPAR0033815|Costa Rica|658[0n]|BOLD:AAM5842
Dolichogenidea Janzen57|[1418]|DHJPAR0033812|Costa Rica|658[0n]|BOLD:AAM5842
Dolichogenidea Janzen57|[1419]|DHJPAR0033817|Costa Rica|658[0n]|BOLD:AAM5842
Dolichogenidea Janzen57|[1420]|DHJPAR0033814|Costa Rica|658[0n]|BOLD:AAM5842
Dolichogenidea Janzen57|[1421]|DHJPAR0033822|Costa Rica|658[0n]|BOLD:AAM5842
Dolichogenidea Janzen57|[1422]|DHJPAR0033816|Costa Rica|658[0n]|BOLD:AAM5842
Dolichogenidea Janzen57|[1423]|DHJPAR0033986|Costa Rica|658[0n]|BOLD:AAM5842
Dolichogenidea Janzen57|[1424]|DHJPAR0031259|Costa Rica|658[0n]|BOLD:AAM5842
Dolichogenidea Janzen57|[1425]|DHJPAR0033772|Costa Rica|658[0n]|BOLD:AAM5842
Dolichogenidea Janzen57|[1426]|DHJPAR0033984|Costa Rica|658[0n]|BOLD:AAM5842
Dolichogenidea Janzen57|[1427]|DHJPAR0033989|Costa Rica|658[0n]|BOLD:AAM5842
Dolichogenidea Janzen57|[1428]|DHJPAR0033770|Costa Rica|658[0n]|BOLD:AAM5842
Dolichogenidea Janzen57|[1429]|DHJPAR0033994|Costa Rica|658[0n]|BOLD:AAM5842
Dolichogenidea Janzen57|[1430]|DHJPAR0033987|Costa Rica|658[0n]|BOLD:AAM5842
Dolichogenidea Janzen57|[1431]|DHJPAR0033998|Costa Rica|658[0n]|BOLD:AAM5842
Dolichogenidea Janzen57|[1432]|DHJPAR0033823|Costa Rica|658[0n]|BOLD:AAM5842
Dolichogenidea Janzen57|[1433]|DHJPAR0034001|Costa Rica|658[0n]|BOLD:AAM5842
Dolichogenidea Janzen57|[1434]|DHJPAR0033995|Costa Rica|658[0n]|BOLD:AAM5842
Dolichogenidea Janzen57|[1435]|DHJPAR0033831|Costa Rica|658[0n]|BOLD:AAM5842
Dolichogenidea Janzen57|[1436]|DHJPAR0033999|Costa Rica|658[0n]|BOLD:AAM5842
Dolichogenidea Janzen57|[1437]|DHJPAR0033901|Costa Rica|658[0n]|BOLD:AAM5842
Dolichogenidea Janzen57|[1438]|DHJPAR0033884|Costa Rica|658[0n]|BOLD:AAM5842
Dolichogenidea Janzen57|[1439]|DHJPAR0033807|Costa Rica|658[0n]|BOLD:AAM5842
Dolichogenidea Janzen57|[1440]|DHJPAR0033820|Costa Rica|658[0n]|BOLD:AAM5842
Dolichogenidea Janzen57|[1441]|DHJPAR0033811|Costa Rica|658[0n]|BOLD:AAM5842
Dolichogenidea Janzen57|[1442]|DHJPAR0033806|Costa Rica|658[0n]|BOLD:AAM5842
Dolichogenidea Janzen57|[1443]|DHJPAR0033930|Costa Rica|658[0n]|BOLD:AAM5842
Dolichogenidea Janzen57|[1444]|DHJPAR0033918|Costa Rica|658[0n]|BOLD:AAM5842
Dolichogenidea Janzen57|[1445]|DHJPAR0033951|Costa Rica|658[0n]|BOLD:AAM5842
Dolichogenidea Janzen57|[1446]|DHJPAR0033924|Costa Rica|658[0n]|BOLD:AAM5842
Dolichogenidea Janzen57|[1447]|DHJPAR0033953|Costa Rica|658[0n]|BOLD:AAM5842
Dolichogenidea Janzen57|[1448]|DHJPAR0033950|Costa Rica|658[0n]|BOLD:AAM5842
Dolichogenidea Janzen57|[1449]|DHJPAR0033955|Costa Rica|658[0n]|BOLD:AAM5842
Dolichogenidea Janzen57|[1450]|DHJPAR0033952|Costa Rica|658[0n]|BOLD:AAM5842
Dolichogenidea Janzen57|[1451]|DHJPAR0033827|Costa Rica|658[0n]|BOLD:AAM5842
Dolichogenidea Janzen57|[1452]|DHJPAR0033810|Costa Rica|658[0n]|BOLD:AAM5842
Dolichogenidea Janzen57|[1453]|DHJPAR0033961|Costa Rica|658[0n]|BOLD:AAM5842
Dolichogenidea Janzen57|[1454]|DHJPAR0033954|Costa Rica|658[0n]|BOLD:AAM5842
Dolichogenidea Janzen57|[1455]|DHJPAR0033967|Costa Rica|658[0n]|BOLD:AAM5842
Dolichogenidea Janzen57|[1456]|DHJPAR0033962|Costa Rica|658[0n]|BOLD:AAM5842
Dolichogenidea Janzen57|[1457]|DHJPAR0033974|Costa Rica|658[0n]|BOLD:AAM5842
Dolichogenidea Janzen57|[1458]|DHJPAR0033965|Costa Rica|658[0n]|BOLD:AAM5842
Dolichogenidea Janzen57|[1459]|DHJPAR0033976|Costa Rica|658[0n]|BOLD:AAM5842
Dolichogenidea Janzen57|[1460]|DHJPAR0033968|Costa Rica|658[0n]|BOLD:AAM5842
Dolichogenidea Janzen57|[1461]|DHJPAR0033980|Costa Rica|658[0n]|BOLD:AAM5842
Dolichogenidea Janzen57|[1462]|DHJPAR0033977|Costa Rica|658[0n]|BOLD:AAM5842
Dolichogenidea Janzen57|[1463]|DHJPAR0033983|Costa Rica|658[0n]|BOLD:AAM5842
Dolichogenidea Janzen57|[1464]|DHJPAR0033979|Costa Rica|658[0n]|BOLD:AAM5842
Dolichogenidea Janzen57|[1465]|DHJPAR0033833|Costa Rica|658[0n]|BOLD:AAM5842
Dolichogenidea Janzen57|[1466]|DHJPAR0033826|Costa Rica|658[0n]|BOLD:AAM5842
Dolichogenidea Janzen57|[1467]|DHJPAR0031214|Costa Rica|658[0n]|BOLD:AAM5842
Dolichogenidea Janzen57|[1468]|DHJPAR0033981|Costa Rica|658[0n]|BOLD:AAM5842
Dolichogenidea Janzen57|[1469]|DHJPAR0031212|Costa Rica|658[0n]|BOLD:AAM5842
Dolichogenidea Janzen57|[1470]|DHJPAR0031213|Costa Rica|658[0n]|BOLD:AAM5842
Dolichogenidea Janzen57|[1471]|DHJPAR0033875|Costa Rica|658[0n]|BOLD:AAM5842
Dolichogenidea Janzen57|[1472]|DHJPAR0031211|Costa Rica|658[0n]|BOLD:AAM5842
Dolichogenidea Janzen57|[1473]|DHJPAR0031208|Costa Rica|658[0n]|BOLD:AAM5842

Dolichogenidea Janzen57|[1471]|DHJPAR0033875|Costa Rica|658[0n]|BOLD:AAM5842
Dolichogenidea Janzen57|[1472]|DHJPAR0031211|Costa Rica|658[0n]|BOLD:AAM5842
Dolichogenidea Janzen57|[1473]|DHJPAR0031208|Costa Rica|658[0n]|BOLD:AAM5842
Dolichogenidea Janzen57|[1474]|DHJPAR0033874|Costa Rica|658[0n]|BOLD:AAM5842
Dolichogenidea Janzen57|[1475]|DHJPAR0031199|Costa Rica|658[0n]|BOLD:AAM5842
Dolichogenidea Janzen57|[1476]|DHJPAR0031205|Costa Rica|658[0n]|BOLD:AAM5842
Dolichogenidea Janzen57|[1477]|DHJPAR0033873|Costa Rica|658[0n]|BOLD:AAM5842
Dolichogenidea Janzen57|[1478]|DHJPAR0031196|Costa Rica|658[0n]|BOLD:AAM5842
Dolichogenidea Janzen57|[1479]|DHJPAR0033835|Costa Rica|658[0n]|BOLD:AAM5842
Dolichogenidea Janzen57|[1480]|DHJPAR0033832|Costa Rica|658[0n]|BOLD:AAM5842
Dolichogenidea Janzen57|[1481]|DHJPAR0031195|Costa Rica|658[0n]|BOLD:AAM5842
Dolichogenidea Janzen57|[1482]|DHJPAR0033872|Costa Rica|658[0n]|BOLD:AAM5842
Dolichogenidea Janzen57|[1483]|DHJPAR0033871|Costa Rica|658[0n]|BOLD:AAM5842
Dolichogenidea Janzen57|[1484]|DHJPAR0031194|Costa Rica|658[0n]|BOLD:AAM5842
Dolichogenidea Janzen57|[1485]|DHJPAR0031189|Costa Rica|658[0n]|BOLD:AAM5842
Dolichogenidea Janzen57|[1486]|DHJPAR0033870|Costa Rica|658[0n]|BOLD:AAM5842
Dolichogenidea Janzen57|[1487]|DHJPAR0033869|Costa Rica|658[0n]|BOLD:AAM5842
Dolichogenidea Janzen57|[1488]|DHJPAR0031188|Costa Rica|658[0n]|BOLD:AAM5842
Dolichogenidea Janzen57|[1489]|DHJPAR0034075|Costa Rica|658[0n]|BOLD:AAM5842
Dolichogenidea Janzen57|[1490]|DHJPAR0033867|Costa Rica|658[0n]|BOLD:AAM5842
Dolichogenidea Janzen57|[1491]|DHJPAR0034067|Costa Rica|658[0n]|BOLD:AAM5842
Dolichogenidea Janzen57|[1492]|DHJPAR0034070|Costa Rica|658[0n]|BOLD:AAM5842
Dolichogenidea Janzen57|[1493]|DHJPAR0033845|Costa Rica|658[0n]|BOLD:AAM5842
Dolichogenidea Janzen57|[1494]|DHJPAR0033834|Costa Rica|658[0n]|BOLD:AAM5842
Dolichogenidea Janzen57|[1495]|DHJPAR0031294|Costa Rica|658[0n]|BOLD:AAM5842
Dolichogenidea Janzen57|[1496]|DHJPAR0031426|Costa Rica|658[0n]|BOLD:AAM5842
Dolichogenidea Janzen57|[1497]|DHJPAR0031427|Costa Rica|658[0n]|BOLD:AAM5842
Dolichogenidea Janzen57|[1498]|DHJPAR0031261|Costa Rica|658[0n]|BOLD:AAM5842
Dolichogenidea Janzen57|[1499]|DHJPAR0031429|Costa Rica|658[0n]|BOLD:AAM5842
Dolichogenidea Janzen57|[1500]|DHJPAR0031293|Costa Rica|658[0n]|BOLD:AAM5842
Dolichogenidea Janzen57|[1501]|DHJPAR0031306|Costa Rica|658[0n]|BOLD:AAM5842
Dolichogenidea Janzen57|[1502]|DHJPAR0031428|Costa Rica|658[0n]|BOLD:AAM5842
Dolichogenidea Janzen57|[1503]|DHJPAR0031244|Costa Rica|658[0n]|BOLD:AAM5842
Dolichogenidea Janzen57|[1504]|DHJPAR0031296|Costa Rica|658[0n]|BOLD:AAM5842
Dolichogenidea Janzen57|[1505]|DHJPAR0031300|Costa Rica|658[0n]|BOLD:AAM5842
Dolichogenidea Janzen57|[1506]|DHJPAR0031304|Costa Rica|658[0n]|BOLD:AAM5842
Dolichogenidea Janzen57|[1507]|DHJPAR0031397|Costa Rica|658[0n]|BOLD:AAM5842
Dolichogenidea Janzen57|[1508]|DHJPAR0031263|Costa Rica|658[0n]|BOLD:AAM5842
Dolichogenidea Janzen57|[1509]|DHJPAR0031246|Costa Rica|658[0n]|BOLD:AAM5842
Dolichogenidea Janzen57|[1510]|DHJPAR0031241|Costa Rica|658[0n]|BOLD:AAM5842
Dolichogenidea Janzen57|[1511]|DHJPAR0031422|Costa Rica|658[0n]|BOLD:AAM5842
Dolichogenidea Janzen57|[1512]|DHJPAR0031245|Costa Rica|658[0n]|BOLD:AAM5842
Dolichogenidea Janzen57|[1513]|DHJPAR0031417|Costa Rica|658[0n]|BOLD:AAM5842
Dolichogenidea Janzen57|[1514]|DHJPAR0031421|Costa Rica|658[0n]|BOLD:AAM5842
Dolichogenidea Janzen57|[1515]|DHJPAR0031393|Costa Rica|658[0n]|BOLD:AAM5842
Dolichogenidea Janzen57|[1516]|DHJPAR0031394|Costa Rica|658[0n]|BOLD:AAM5842
Dolichogenidea Janzen57|[1517]|DHJPAR0031400|Costa Rica|658[0n]|BOLD:AAM5842
Dolichogenidea Janzen57|[1518]|DHJPAR0031349|Costa Rica|658[0n]|BOLD:AAM5842
Dolichogenidea Janzen57|[1519]|DHJPAR0031264|Costa Rica|658[0n]|BOLD:AAM5842
Dolichogenidea Janzen57|[1520]|DHJPAR0031399|Costa Rica|658[0n]|BOLD:AAM5842
Dolichogenidea Janzen57|[1521]|DHJPAR0031391|Costa Rica|658[0n]|BOLD:AAM5842
Dolichogenidea Janzen57|[1522]|DHJPAR0031392|Costa Rica|658[0n]|BOLD:AAM5842
Dolichogenidea Janzen57|[1523]|DHJPAR0031386|Costa Rica|658[0n]|BOLD:AAM5842
Dolichogenidea Janzen57|[1524]|DHJPAR0031390|Costa Rica|658[0n]|BOLD:AAM5842
Dolichogenidea Janzen57|[1525]|DHJPAR0031331|Costa Rica|658[0n]|BOLD:AAM5842
Dolichogenidea Janzen57|[1526]|DHJPAR0031333|Costa Rica|658[0n]|BOLD:AAM5842
Dolichogenidea Janzen57|[1527]|DHJPAR0031368|Costa Rica|658[0n]|BOLD:AAM5842
Dolichogenidea Janzen57|[1528]|DHJPAR0031379|Costa Rica|658[0n]|BOLD:AAM5842
Dolichogenidea Janzen57|[1529]|DHJPAR0031317|Costa Rica|658[0n]|BOLD:AAM5842
Dolichogenidea Janzen57|[1530]|DHJPAR0031328|Costa Rica|658[0n]|BOLD:AAM5842
Dolichogenidea Janzen57|[1531]|DHJPAR0031250|Costa Rica|658[0n]|BOLD:AAM5842
Dolichogenidea Janzen57|[1532]|DHJPAR0031311|Costa Rica|658[0n]|BOLD:AAM5842
Dolichogenidea Janzen57|[1533]|DHJPAR0031254|Costa Rica|658[0n]|BOLD:AAM5842
Dolichogenidea Janzen57|[1534]|DHJPAR0031247|Costa Rica|658[0n]|BOLD:AAM5842
Dolichogenidea Janzen57|[1535]|DHJPAR0031260|Costa Rica|658[0n]|BOLD:AAM5842
Dolichogenidea Janzen57|[1536]|DHJPAR0031253|Costa Rica|658[0n]|BOLD:AAM5842
Dolichogenidea Janzen57|[1537]|DHJPAR0033963|Costa Rica|658[0n]|BOLD:AAM5842
Dolichogenidea Janzen57|[1538]|DHJPAR0033956|Costa Rica|658[0n]|BOLD:AAM5842
Dolichogenidea Janzen57|[1539]|DHJPAR0031340|Costa Rica|658[0n]|BOLD:AAM5842
Dolichogenidea Janzen57|[1540]|DHJPAR0031358|Costa Rica|658[0n]|BOLD:AAM5842
Dolichogenidea Janzen57|[1541]|DHJPAR0033978|Costa Rica|658[0n]|BOLD:AAM5842
Dolichogenidea Janzen57|[1542]|DHJPAR0033975|Costa Rica|658[0n]|BOLD:AAM5842
Dolichogenidea Janzen57|[1543]|DHJPAR0031338|Costa Rica|658[0n]|BOLD:AAM5842
Dolichogenidea Janzen57|[1544]|DHJPAR0031339|Costa Rica|658[0n]|BOLD:AAM5842
Dolichogenidea Janzen57|[1545]|DHJPAR0033763|Costa Rica|658[0n]|BOLD:AAM5842
Dolichogenidea Janzen57|[1546]|DHJPAR0033761|Costa Rica|658[0n]|BOLD:AAM5842
Dolichogenidea Janzen57|[1547]|DHJPAR0031262|Costa Rica|658[0n]|BOLD:AAM5842
Dolichogenidea Janzen57|[1548]|DHJPAR0031430|Costa Rica|658[0n]|BOLD:AAM5842
Dolichogenidea Janzen57|[1549]|DHJPAR0033738|Costa Rica|658[0n]|BOLD:AAM5842
Dolichogenidea Janzen57|[1550]|DHJPAR0033733|Costa Rica|658[0n]|BOLD:AAM5842
Dolichogenidea Janzen57|[1551]|DHJPAR0033765|Costa Rica|658[0n]|BOLD:AAM5842
Dolichogenidea Janzen57|[1552]|DHJPAR0033764|Costa Rica|658[0n]|BOLD:AAM5842
Dolichogenidea Janzen57|[1553]|DHJPAR0031354|Costa Rica|658[0n]|BOLD:AAM5842
Dolichogenidea Janzen57|[1554]|DHJPAR0031412|Costa Rica|658[0n]|BOLD:AAM5842
Dolichogenidea Janzen57|[1555]|DHJPAR0033752|Costa Rica|658[0n]|BOLD:AAM5842
Dolichogenidea Janzen57|[1556]|DHJPAR0033741|Costa Rica|658[0n]|BOLD:AAM5842
Dolichogenidea Janzen57|[1557]|DHJPAR0033840|Costa Rica|658[0n]|BOLD:AAM5842
Dolichogenidea Janzen57|[1558]|DHJPAR0033848|Costa Rica|658[0n]|BOLD:AAM5842
Dolichogenidea Janzen57|[1559]|DHJPAR0033846|Costa Rica|658[0n]|BOLD:AAM5842
Dolichogenidea Janzen57|[1560]|DHJPAR0033943|Costa Rica|658[0n]|BOLD:AAM5842
Dolichogenidea Janzen57|[1561]|DHJPAR0033942|Costa Rica|658[0n]|BOLD:AAM5842
Dolichogenidea Janzen57|[1562]|DHJPAR0033947|Costa Rica|658[0n]|BOLD:AAM5842
Dolichogenidea Janzen57|[1563]|DHJPAR0033944|Costa Rica|658[0n]|BOLD:AAM5842
Dolichogenidea Janzen57|[1564]|DHJPAR0033949|Costa Rica|658[0n]|BOLD:AAM5842
Dolichogenidea Janzen57|[1565]|DHJPAR0033948|Costa Rica|658[0n]|BOLD:AAM5842
Hymenoptera|[1566]|CBG-A04315-C04|Costa Rica|656[0n]|BOLD:AAM5842
Dolichogenidea Janzen57|[1567]|CBG-A11789-G05|Costa Rica|656[0n]|BOLD:AAM5842
Dolichogenidea Janzen57|[1568]|CBG-A11791-B05|Costa Rica|655[0n]|BOLD:AAM5842
Dolichogenidea Janzen57|[1569]|DHJPAR0031190|Costa Rica|658[0n]|BOLD:AAM5842
Dolichogenidea Janzen57|[1570]|DHJPAR0031388|Costa Rica|658[1n]|BOLD:AAM5842
Dolichogenidea Janzen57|[1571]|BIOUG58586-A10|Costa Rica|655[0n]|BOLD:AAM5842

Dolichogenidea Janzen57|[1569]|DHJPAR0031190|Costa Rica|658[0n]|BOLD:AAM5842
Dolichogenidea Janzen57|[1570]|DHJPAR0031388|Costa Rica|658[1n]|BOLD:AAM5842
Dolichogenidea Janzen57|[1571]|BIOUG58586-A10|Costa Rica|655[0n]|BOLD:AAM5842
Dolichogenidea Janzen57|[1572]|BIOUG54626-B02|Costa Rica|656[0n]|BOLD:AAM5842
Dolichogenidea Janzen57|[1573]|CBG-A11736-F08|Costa Rica|655[0n]|BOLD:AAM5842
Dolichogenidea Janzen57|[1574]|CBG-A11734-H04|Costa Rica|655[0n]|BOLD:AAM5842
Dolichogenidea Janzen57|[1575]|BIOUG60167-A01|Costa Rica|654[0n]|BOLD:AAM5842
Dolichogenidea Janzen57|[1576]|BIOUG46717-C07|Costa Rica|654[0n]|BOLD:AAM5842
Hymenoptera|[1577]|CBG-A04319-F05|Costa Rica|654[0n]|BOLD:AAM5842
Dolichogenidea Janzen57|[1578]|BIOUG71866-G07|Costa Rica|654[0n]|BOLD:AAM5842
Dolichogenidea Janzen57|[1579]|BIOUG71090-D10|Costa Rica|654[0n]|BOLD:AAM5842
Dolichogenidea Janzen57|[1580]|BIOUG64130-B09|Costa Rica|654[0n]|BOLD:AAM5842
Dolichogenidea Janzen57|[1581]|CBG-A11984-B12|Costa Rica|654[0n]|BOLD:AAM5842
Dolichogenidea Janzen57|[1582]|CBG-A11720-B09|Costa Rica|654[0n]|BOLD:AAM5842
Dolichogenidea Janzen57|[1583]|CBG-A17822-H06|Costa Rica|654[0n]|BOLD:AAM5842
Dolichogenidea Janzen57|[1584]|CBG-A17886-B07|Costa Rica|654[0n]|BOLD:AAM5842
Hymenoptera|[1585]|CBG-A04315-B06|Costa Rica|653[0n]|BOLD:AAM5842
Dolichogenidea Janzen57|[1586]|BIOUG49167-H07|Costa Rica|653[0n]|BOLD:AAM5842
Hymenoptera|[1587]|CBG-A04305-D06|Costa Rica|653[0n]|BOLD:AAM5842
Hymenoptera|[1588]|CBG-A04315-C09|Costa Rica|653[0n]|BOLD:AAM5842
Dolichogenidea Janzen57|[1589]|CBG-A11791-D04|Costa Rica|653[0n]|BOLD:AAM5842
Dolichogenidea Janzen57|[1590]|DHJPAR0033789|Costa Rica|658[0n]|BOLD:AAM5842
Dolichogenidea Janzen57|[1591]|DHJPAR0034045|Costa Rica|658[0n]|BOLD:AAM5842
Hymenoptera|[1592]|CBG-A04312-B07|Costa Rica|653[0n]|BOLD:AAM5842
Hymenoptera|[1593]|CBG-A04320-C11|Costa Rica|653[0n]|BOLD:AAM5842
Dolichogenidea Janzen57|[1594]|CBG-A11743-B07|Costa Rica|653[0n]|BOLD:AAM5842
Dolichogenidea Janzen57|[1595]|DHJPAR0033818|Costa Rica|658[0n]|BOLD:AAM5842
Hymenoptera|[1596]|CBG-A04320-B06|Costa Rica|653[0n]|BOLD:AAM5842
Hymenoptera|[1597]|CBG-A04315-D01|Costa Rica|653[0n]|BOLD:AAM5842
Dolichogenidea Janzen57|[1598]|CBG-A12885-F09|Costa Rica|653[0n]|BOLD:AAM5842
Dolichogenidea Janzen57|[1599]|CBG-A11654-E06|Costa Rica|653[0n]|BOLD:AAM5842
Dolichogenidea Janzen57|[1600]|CBG-A17883-H01|Costa Rica|653[0n]|BOLD:AAM5842
Dolichogenidea Janzen57|[1601]|DHJPAR0033843|Costa Rica|621[0n]|BOLD:AAM5842
Dolichogenidea Janzen57|[1602]|DHJPAR0034028|Costa Rica|618[0n]|BOLD:AAM5842
Dolichogenidea Janzen57|[1603]|DHJPAR0033966|Costa Rica|627[0n]|BOLD:AAM5842
Dolichogenidea Janzen57|[1604]|DHJPAR0034061|Costa Rica|627[0n]|BOLD:AAM5842
Dolichogenidea Janzen57|[1605]|DHJPAR0033786|Costa Rica|614[0n]|BOLD:AAM5842
Dolichogenidea Janzen57|[1606]|BIOUG33179-C04|Costa Rica|603[0n]|BOLD:AAM5842
Dolichogenidea Janzen57|[1607]|DHJPAR0033838|Costa Rica|632[0n]|BOLD:AAM5842
Dolichogenidea Janzen57|[1608]|DHJPAR0033879|Costa Rica|632[0n]|BOLD:AAM5842
Dolichogenidea Janzen57|[1609]|BIOUG33118-D02|Costa Rica|597[0n]|BOLD:AAM5842
Dolichogenidea Janzen57|[1610]|BIOUG36704-E12|Costa Rica|595[0n]|BOLD:AAM5842
Dolichogenidea Janzen57|[1611]|BIOUG36494-H07|Costa Rica|594[0n]|BOLD:AAM5842
Dolichogenidea Janzen57|[1612]|DHJPAR0033992|Costa Rica|540[0n]|BOLD:AAM5842
Dolichogenidea Janzen57|[1613]|DHJPAR0033993|Costa Rica|386[0n]|BOLD:AAM5842
Dolichogenidea Janzen57|[1614]|BIOUG36589-A07|Costa Rica|567[0n]|BOLD:AAM5842
Dolichogenidea Janzen57|[1615]|BIOUG28721-H10|Costa Rica|585[0n]|BOLD:AAM5842
Dolichogenidea Janzen57|[1616]|DHJPAR0033773|Costa Rica|658[0n]|BOLD:AAM5842
Dolichogenidea Janzen57|[1617]|DHJPAR0033829|Costa Rica|658[0n]|BOLD:AAM5842
Dolichogenidea Janzen57|[1618]|CBG-A11790-F09|Costa Rica|653[0n]|BOLD:AAM5842
Dolichogenidea Janzen57|[1619]|DHJPAR0034049|Costa Rica|658[0n]|BOLD:AAM5842
Hymenoptera|[1620]|CBG-A04315-B08|Costa Rica|659[0n]|BOLD:AAM5842
Dolichogenidea Rodriguez197|[1621]|DHJPAR0013101|Costa Rica|657[2n]|BOLD:AAC8392
Dolichogenidea Rodriguez197|[1622]|BIOUG18510-A01|Costa Rica|617[1n]|BOLD:AAC8392
Dolichogenidea Rodriguez197|[1623]|DHJPAR0013663|Costa Rica|593[0n]|BOLD:AAC8392
Dolichogenidea Rodriguez197|[1624]|DHJPAR0013116|Costa Rica|657[2n]|BOLD:AAC8392
Dolichogenidea Rodriguez197|[1625]|DHJPAR0013027|Costa Rica|657[0n]|BOLD:AAC8392
Dolichogenidea Rodriguez197|[1626]|DHJPAR0024691|Costa Rica|657[0n]|BOLD:AAC8392
Dolichogenidea Rodriguez197|[1627]|DHJPAR0024692|Costa Rica|657[0n]|BOLD:AAC8392
Dolichogenidea Rodriguez197|[1628]|DHJPAR0024700|Costa Rica|657[0n]|BOLD:AAC8392
Dolichogenidea Rodriguez197|[1629]|BIOUG05414-B06|Costa Rica|626[0n]|BOLD:AAC8392
Dolichogenidea Rodriguez197|[1630]|BIOUG05195-A07|Costa Rica|609[0n]|BOLD:AAC8392
Dolichogenidea Rodriguez197|[1631]|BIOUG09074-B04|Costa Rica|602[0n]|BOLD:AAC8392
Dolichogenidea Rodriguez197|[1632]|BIOUG05865-F05|Costa Rica|628[0n]|BOLD:AAC8392
Dolichogenidea Rodriguez197|[1633]|BIOUG17842-G03|Costa Rica|555[0n]|BOLD:AAC8392
Dolichogenidea Rodriguez197|[1634]|BIOUG18748-E05|Costa Rica|567[0n]|BOLD:AAC8392
Dolichogenidea Rodriguez197|[1635]|BIOUG05346-B09|Costa Rica|573[0n]|BOLD:AAC8392
Dolichogenidea Rodriguez197|[1636]|BIOUG05346-C03|Costa Rica|591[0n]|BOLD:AAC8392
Dolichogenidea Rodriguez197|[1637]|BIOUG09739-E01|Costa Rica|594[0n]|BOLD:AAC8392
Dolichogenidea Rodriguez197|[1638]|BIOUG05422-H01|Costa Rica|632[0n]|BOLD:AAC8392
Dolichogenidea Rodriguez197|[1639]|BIOUG05865-F06|Costa Rica|632[0n]|BOLD:AAC8392
Dolichogenidea Rodriguez197|[1640]|DHJPAR0031743|Costa Rica|636[0n]|BOLD:AAC8392
Dolichogenidea Rodriguez197|[1641]|BIOUG08911-A09|Costa Rica|638[0n]|BOLD:AAC8392
Dolichogenidea Rodriguez197|[1642]|DHJPAR0013174|Costa Rica|655[0n]|BOLD:AAC8392
Dolichogenidea Rodriguez197|[1643]|DHJPAR0013079|Costa Rica|657[1n]|BOLD:AAC8392
Dolichogenidea Rodriguez197|[1644]|DHJPAR0031646|Costa Rica|658[0n]|BOLD:AAC8392
Dolichogenidea Rodriguez197|[1645]|DHJPAR0031686|Costa Rica|658[0n]|BOLD:AAC8392
Dolichogenidea Rodriguez197|[1646]|DHJPAR0031703|Costa Rica|658[0n]|BOLD:AAC8392
Dolichogenidea Rodriguez197|[1647]|DHJPAR0031707|Costa Rica|658[0n]|BOLD:AAC8392
Dolichogenidea Rodriguez197|[1648]|DHJPAR0031724|Costa Rica|658[0n]|BOLD:AAC8392
Dolichogenidea Rodriguez197|[1649]|DHJPAR0031725|Costa Rica|658[0n]|BOLD:AAC8392
Dolichogenidea Rodriguez197|[1650]|DHJPAR0031756|Costa Rica|658[0n]|BOLD:AAC8392
Dolichogenidea Rodriguez197|[1651]|DHJPAR0031762|Costa Rica|658[0n]|BOLD:AAC8392
Dolichogenidea Rodriguez197|[1652]|DHJPAR0031773|Costa Rica|658[0n]|BOLD:AAC8392
Dolichogenidea Rodriguez197|[1653]|DHJPAR0031824|Costa Rica|658[0n]|BOLD:AAC8392
Dolichogenidea Rodriguez145|[1654]|DHJPAR0013115|Costa Rica|657[1n]|BOLD:AAB9372
Dolichogenidea Rodriguez145|[1655]|BIOUG17841-B01|Costa Rica|537[0n]|BOLD:AAB9372
Dolichogenidea Rodriguez145|[1656]|DHJPAR0013077|Costa Rica|657[5n]|BOLD:AAB9372
Dolichogenidea Rodriguez145|[1657]|DHJPAR0013203|Costa Rica|657[1n]|BOLD:AAB9372
Dolichogenidea Rodriguez145|[1658]|BIOUG09074-C02|Costa Rica|602[0n]|BOLD:AAB9372
Dolichogenidea Rodriguez145|[1659]|DHJPAR0013042|Costa Rica|655[1n]|BOLD:AAB9372
Dolichogenidea Rodriguez145|[1660]|BIOUG09441-F09|Costa Rica|600[0n]|BOLD:AAB9372
Dolichogenidea Rodriguez145|[1661]|BIOUG17841-A06|Costa Rica|585[1n]|BOLD:AAB9372
Dolichogenidea Rodriguez145|[1662]|BIOUG18748-F12|Costa Rica|540[0n]|BOLD:AAB9372
Dolichogenidea Rodriguez145|[1663]|BIOUG17841-F01|Costa Rica|567[0n]|BOLD:AAB9372
Dolichogenidea Rodriguez145|[1664]|BIOUG09434-G06|Costa Rica|594[0n]|BOLD:AAB9372
Dolichogenidea Rodriguez145|[1665]|DHJPAR0031688|Costa Rica|658[0n]|BOLD:AAB9372
Dolichogenidea Rodriguez145|[1666]|BIOUG05414-F05|Costa Rica|631[0n]|BOLD:AAB9372
Dolichogenidea Rodriguez145|[1667]|BIOUG05414-H07|Costa Rica|631[0n]|BOLD:AAB9372
Dolichogenidea Rodriguez145|[1668]|DHJPAR0013050|Costa Rica|657[0n]|BOLD:AAB9372
Dolichogenidea Rodriguez145|[1669]|DHJPAR0013036|Costa Rica|657[0n]|BOLD:AAB9372

Dolichogenidea Rodriguez145|[1667]|BIOUG05414-H07|Costa Rica|631[0n]|BOLD:AAB9372
Dolichogenidea Rodriguez145|[1668]|DHJPAR0013050|Costa Rica|657[0n]|BOLD:AAB9372
Dolichogenidea Rodriguez145|[1669]|DHJPAR0013036|Costa Rica|657[0n]|BOLD:AAB9372
Dolichogenidea Rodriguez145|[1670]|DHJPAR0013037|Costa Rica|657[0n]|BOLD:AAB9372
Dolichogenidea Rodriguez145|[1671]|DHJPAR0013094|Costa Rica|657[0n]|BOLD:AAB9372
Dolichogenidea Rodriguez145|[1672]|DHJPAR0013551|Costa Rica|657[0n]|BOLD:AAB9372
Dolichogenidea Rodriguez145|[1673]|DHJPAR0024694|Costa Rica|657[0n]|BOLD:AAB9372
Dolichogenidea Rodriguez145|[1674]|DHJPAR0024696|Costa Rica|657[0n]|BOLD:AAB9372
Dolichogenidea Rodriguez145|[1675]|DHJPAR0024708|Costa Rica|657[0n]|BOLD:AAB9372
Dolichogenidea Rodriguez145|[1676]|DHJPAR0031566|Costa Rica|658[0n]|BOLD:AAB9372
Dolichogenidea Rodriguez145|[1677]|DHJPAR0031600|Costa Rica|658[0n]|BOLD:AAB9372
Dolichogenidea Rodriguez145|[1678]|DHJPAR0031604|Costa Rica|658[0n]|BOLD:AAB9372
Dolichogenidea Rodriguez145|[1679]|DHJPAR0031628|Costa Rica|658[0n]|BOLD:AAB9372
Dolichogenidea Rodriguez145|[1680]|DHJPAR0031694|Costa Rica|658[0n]|BOLD:AAB9372
Dolichogenidea Rodriguez145|[1681]|DHJPAR0031695|Costa Rica|658[0n]|BOLD:AAB9372
Dolichogenidea Rodriguez145|[1682]|DHJPAR0031706|Costa Rica|658[0n]|BOLD:AAB9372
Dolichogenidea Rodriguez145|[1683]|DHJPAR0031710|Costa Rica|658[0n]|BOLD:AAB9372
Dolichogenidea Rodriguez145|[1684]|DHJPAR0031711|Costa Rica|658[0n]|BOLD:AAB9372
Dolichogenidea Rodriguez145|[1685]|DHJPAR0031734|Costa Rica|658[0n]|BOLD:AAB9372
Dolichogenidea Rodriguez145|[1686]|DHJPAR0031739|Costa Rica|658[0n]|BOLD:AAB9372
Dolichogenidea Rodriguez145|[1687]|DHJPAR0031750|Costa Rica|658[0n]|BOLD:AAB9372
Dolichogenidea Rodriguez145|[1688]|DHJPAR0031755|Costa Rica|658[0n]|BOLD:AAB9372
Dolichogenidea Rodriguez145|[1689]|DHJPAR0031760|Costa Rica|658[0n]|BOLD:AAB9372
Dolichogenidea Rodriguez145|[1690]|DHJPAR0031766|Costa Rica|658[0n]|BOLD:AAB9372
Dolichogenidea Rodriguez145|[1691]|DHJPAR0031767|Costa Rica|658[0n]|BOLD:AAB9372
Dolichogenidea Rodriguez145|[1692]|DHJPAR0031776|Costa Rica|658[0n]|BOLD:AAB9372
Dolichogenidea Rodriguez145|[1693]|DHJPAR0031809|Costa Rica|658[0n]|BOLD:AAB9372
Dolichogenidea Rodriguez145|[1694]|DHJPAR0013089|Costa Rica|657[5n]|BOLD:AAB9372
Dolichogenidea Rodriguez145|[1695]|DHJPAR0012517|Costa Rica|657[0n]|BOLD:AAB9372
Dolichogenidea Rodriguez145|[1696]|DHJPAR0012540|Costa Rica|657[0n]|BOLD:AAB9372
Dolichogenidea Rodriguez145|[1697]|DHJPAR0012554|Costa Rica|657[0n]|BOLD:AAB9372
Dolichogenidea Rodriguez145|[1698]|BIOUG05280-D08|Costa Rica|628[0n]|BOLD:AAB9372
Dolichogenidea Rodriguez145|[1699]|BIOUG61882-F06|Costa Rica|654[0n]|BOLD:AAB9372
Dolichogenidea Rodriguez145|[1700]|DHJPAR0031768|Costa Rica|658[0n]|BOLD:AAB9372
Dolichogenidea Rodriguez145|[1701]|10BZMT-0485|Belize|658[0n]|BOLD:AAB9372
Dolichogenidea Rodriguez195|[1702]|BIOUG68256-F10|Costa Rica|654[0n]|BOLD:AAM5088
Dolichogenidea Rodriguez195|[1703]|BIOUG08265-D05|Costa Rica|586[0n]|BOLD:AAM5088
Dolichogenidea Rodriguez195|[1704]|BIOUG70194-F01|Costa Rica|654[0n]|BOLD:AAM5088
Dolichogenidea Rodriguez195|[1705]|BIOUG07918-G12|Costa Rica|569[0n]|BOLD:AAM5088
Dolichogenidea Rodriguez195|[1706]|BIOUG17843-F03|Costa Rica|546[0n]|BOLD:AAM5088
Dolichogenidea Rodriguez195|[1707]|BIOUG18664-D02|Costa Rica|543[0n]|BOLD:AAM5088
Dolichogenidea Rodriguez195|[1708]|DHJPAR0013122|Costa Rica|657[9n]|BOLD:AAM5088
Dolichogenidea Rodriguez195|[1709]|BIOUG17841-H09|Costa Rica|632[1n]|BOLD:AAM5088
Dolichogenidea Rodriguez195|[1710]|BIOUG07919-F03|Costa Rica|578[1n]|BOLD:AAM5088
Dolichogenidea Rodriguez195|[1711]|BIOUG18510-A11|Costa Rica|617[0n]|BOLD:AAM5088
Dolichogenidea Rodriguez195|[1712]|BIOUG18510-B09|Costa Rica|617[0n]|BOLD:AAM5088
Dolichogenidea Rodriguez195|[1713]|BIOUG17842-B11|Costa Rica|609[0n]|BOLD:AAM5088
Dolichogenidea Rodriguez195|[1714]|BIOUG05422-D08|Costa Rica|658[0n]|BOLD:AAM5088
Dolichogenidea Rodriguez195|[1715]|BIOUG05422-G12|Costa Rica|658[0n]|BOLD:AAM5088
Dolichogenidea Rodriguez195|[1716]|BIOUG07457-G06|Costa Rica|658[0n]|BOLD:AAM5088
Dolichogenidea Rodriguez195|[1717]|BIOUG07617-A03|Costa Rica|658[0n]|BOLD:AAM5088
Dolichogenidea Rodriguez195|[1718]|BIOUG18551-A09|Costa Rica|525[0n]|BOLD:AAM5088
Dolichogenidea Rodriguez195|[1719]|BIOUG70383-A09|Costa Rica|654[0n]|BOLD:AAM5088
Dolichogenidea Rodriguez195|[1720]|BIOUG70435-A11|Costa Rica|654[0n]|BOLD:AAM5088
Dolichogenidea Rodriguez195|[1721]|BIOUG70435-B05|Costa Rica|654[0n]|BOLD:AAM5088
Dolichogenidea Rodriguez195|[1722]|BIOUG70765-E11|Costa Rica|654[0n]|BOLD:AAM5088
Dolichogenidea Rodriguez195|[1723]|BIOUG70804-C08|Costa Rica|654[0n]|BOLD:AAM5088
Dolichogenidea Rodriguez195|[1724]|BIOUG70160-D12|Costa Rica|654[0n]|BOLD:AAM5088
Dolichogenidea Rodriguez195|[1725]|BIOUG68256-B11|Costa Rica|654[0n]|BOLD:AAM5088
Dolichogenidea Rodriguez195|[1726]|BIOUG68256-C08|Costa Rica|654[0n]|BOLD:AAM5088
Dolichogenidea Rodriguez195|[1727]|BIOUG68256-F08|Costa Rica|654[0n]|BOLD:AAM5088
Dolichogenidea Rodriguez195|[1728]|BIOUG62757-D02|Costa Rica|655[0n]|BOLD:AAM5088
Dolichogenidea Rodriguez195|[1729]|BIOUG63124-F05|Costa Rica|654[0n]|BOLD:AAM5088
Dolichogenidea Rodriguez195|[1730]|BIOUG68411-A03|Costa Rica|654[0n]|BOLD:AAM5088
Dolichogenidea Rodriguez195|[1731]|BIOUG60361-C01|Costa Rica|654[0n]|BOLD:AAM5088
Dolichogenidea Rodriguez195|[1732]|BIOUG62904-G01|Costa Rica|654[0n]|BOLD:AAM5088
Dolichogenidea Rodriguez195|[1733]|BIOUG09826-E02|Costa Rica|605[0n]|BOLD:AAM5088
Dolichogenidea Rodriguez195|[1734]|BIOUG09441-H05|Costa Rica|615[0n]|BOLD:AAM5088
Dolichogenidea Rodriguez195|[1735]|BIOUG08267-G11|Costa Rica|602[0n]|BOLD:AAM5088
Dolichogenidea Rodriguez195|[1736]|BIOUG17463-G06|Costa Rica|600[0n]|BOLD:AAM5088
Dolichogenidea Rodriguez195|[1737]|BIOUG17575-A07|Costa Rica|600[0n]|BOLD:AAM5088
Dolichogenidea Rodriguez195|[1738]|BIOUG08074-B11|Costa Rica|597[0n]|BOLD:AAM5088
Dolichogenidea Rodriguez195|[1739]|BIOUG08267-B02|Costa Rica|596[0n]|BOLD:AAM5088
Dolichogenidea Rodriguez195|[1740]|BIOUG17463-E02|Costa Rica|594[0n]|BOLD:AAM5088
Dolichogenidea Rodriguez195|[1741]|BIOUG17530-E05|Costa Rica|588[0n]|BOLD:AAM5088
Dolichogenidea Rodriguez195|[1742]|BIOUG17841-F05|Costa Rica|573[0n]|BOLD:AAM5088
Dolichogenidea Rodriguez195|[1743]|BIOUG13945-C07|Costa Rica|585[0n]|BOLD:AAM5088
Dolichogenidea Rodriguez195|[1744]|BIOUG09826-E06|Costa Rica|592[0n]|BOLD:AAM5088
Dolichogenidea Rodriguez195|[1745]|BIOUG17616-B04|Costa Rica|588[0n]|BOLD:AAM5088
Dolichogenidea Rodriguez195|[1746]|BIOUG18499-F01|Costa Rica|588[0n]|BOLD:AAM5088
Dolichogenidea Rodriguez195|[1747]|BIOUG18661-H08|Costa Rica|588[0n]|BOLD:AAM5088
Dolichogenidea Rodriguez195|[1748]|BIOUG18665-C11|Costa Rica|585[0n]|BOLD:AAM5088
Dolichogenidea Rodriguez195|[1749]|BIOUG13945-F06|Costa Rica|579[0n]|BOLD:AAM5088
Dolichogenidea Rodriguez195|[1750]|BIOUG09738-F12|Costa Rica|549[0n]|BOLD:AAM5088
Dolichogenidea Rodriguez195|[1751]|BIOUG09441-G07|Costa Rica|591[0n]|BOLD:AAM5088
Dolichogenidea Rodriguez195|[1752]|BIOUG17841-B09|Costa Rica|576[0n]|BOLD:AAM5088
Dolichogenidea Rodriguez195|[1753]|BIOUG17841-G03|Costa Rica|576[0n]|BOLD:AAM5088
Dolichogenidea Rodriguez195|[1754]|BIOUG18551-E09|Costa Rica|552[0n]|BOLD:AAM5088
Dolichogenidea Rodriguez195|[1755]|BIOUG07919-B01|Costa Rica|577[0n]|BOLD:AAM5088
Dolichogenidea Rodriguez195|[1756]|BIOUG29810-F04|Costa Rica|576[0n]|BOLD:AAM5088
Dolichogenidea Rodriguez195|[1757]|BIOUG17843-B11|Costa Rica|543[0n]|BOLD:AAM5088
Dolichogenidea Rodriguez195|[1758]|BIOUG17842-C06|Costa Rica|534[3n]|BOLD:AAM5088
Dolichogenidea Rodriguez195|[1759]|BIOUG17462-H07|Costa Rica|567[0n]|BOLD:AAM5088
Dolichogenidea Rodriguez195|[1760]|BIOUG17842-H02|Costa Rica|555[0n]|BOLD:AAM5088
Dolichogenidea Rodriguez195|[1761]|BIOUG13945-A03|Costa Rica|555[0n]|BOLD:AAM5088
Dolichogenidea Rodriguez195|[1762]|BIOUG07918-D04|Costa Rica|596[0n]|BOLD:AAM5088
Dolichogenidea Rodriguez195|[1763]|BIOUG07919-A09|Costa Rica|610[0n]|BOLD:AAM5088
Dolichogenidea Rodriguez195|[1764]|BIOUG07919-C07|Costa Rica|610[0n]|BOLD:AAM5088
Dolichogenidea Rodriguez195|[1765]|BIOUG07918-F05|Costa Rica|617[0n]|BOLD:AAM5088
Dolichogenidea Rodriguez195|[1766]|BIOUG07918-D03|Costa Rica|618[0n]|BOLD:AAM5088
Dolichogenidea Rodriguez195|[1767]|BIOUG18397-A04|Costa Rica|632[0n]|BOLD:AAM5088
Dolichogenidea Rodriguez195|[1768]|BIOUG53421-H07|Costa Rica|[illegible]|BOLD:AAM5088

Dolichogenidea Rodriguez195|[1765]|BIOUG07918-F05|Costa Rica|617[0n]|BOLD:AAM5088
Dolichogenidea Rodriguez195|[1766]|BIOUG07918-D03|Costa Rica|618[0n]|BOLD:AAM5088
Dolichogenidea Rodriguez195|[1767]|BIOUG18397-A04|Costa Rica|632[0n]|BOLD:AAM5088
Dolichogenidea Rodriguez195|[1768]|BIOUG52431-H07|Costa Rica|652[0n]|BOLD:AAM5088
Dolichogenidea Rodriguez195|[1769]|BIOUG68716-D11|Costa Rica|652[0n]|BOLD:AAM5088
Dolichogenidea Rodriguez195|[1770]|BIOUG70762-A01|Costa Rica|652[0n]|BOLD:AAM5088
Dolichogenidea Rodriguez195|[1771]|BIOUG52330-E09|Costa Rica|653[0n]|BOLD:AAM5088
Dolichogenidea Rodriguez195|[1772]|BIOUG59466-C12|Costa Rica|653[0n]|BOLD:AAM5088
Dolichogenidea Rodriguez195|[1773]|BIOUG63126-A08|Costa Rica|653[0n]|BOLD:AAM5088
Dolichogenidea Rodriguez195|[1774]|BIOUG68911-H06|Costa Rica|653[0n]|BOLD:AAM5088
Dolichogenidea Rodriguez195|[1775]|BIOUG70383-E02|Costa Rica|653[0n]|BOLD:AAM5088
Dolichogenidea Rodriguez195|[1776]|BIOUG70765-D07|Costa Rica|653[0n]|BOLD:AAM5088
Dolichogenidea Rodriguez195|[1777]|BIOUG60811-C06|Costa Rica|654[0n]|BOLD:AAM5088
Dolichogenidea Rodriguez195|[1778]|BIOUG70383-D08|Costa Rica|655[0n]|BOLD:AAM5088
Dolichogenidea Rodriguez195|[1779]|BIOUG57075-B01|Costa Rica|656[0n]|BOLD:AAM5088
Dolichogenidea Rodriguez195|[1780]|BIOUG07615-H10|Costa Rica|673[0n]|BOLD:AAM5088
Dolichogenidea Janzen53|[1781]|BIOUG90782-E11|Costa Rica|652[0n]|BOLD:AAC7481
Dolichogenidea Janzen53|[1782]|BIOUG87216-B06|Costa Rica|651[0n]|BOLD:AAC7481
Dolichogenidea Janzen53|[1783]|BIOUG88702-E01|Costa Rica|652[0n]|BOLD:AAC7481
Dolichogenidea Janzen53|[1784]|BIOUG89818-E04|Costa Rica|652[0n]|BOLD:AAC7481
Dolichogenidea Janzen53|[1785]|BIOUG77649-B06|Costa Rica|652[0n]|BOLD:AAC7481
Dolichogenidea Janzen53|[1786]|BIOUG90239-G11|Costa Rica|652[0n]|BOLD:AAC7481
Dolichogenidea Janzen53|[1787]|BIOUG90782-E08|Costa Rica|652[0n]|BOLD:AAC7481
Dolichogenidea Janzen53|[1788]|BIOUG92795-G02|Costa Rica|651[0n]|BOLD:AAC7481
Dolichogenidea Janzen53|[1789]|BIOUG87214-B04|Costa Rica|651[0n]|BOLD:AAC7481
Dolichogenidea Janzen53|[1790]|BIOUG90821-F07|Costa Rica|653[0n]|BOLD:AAC7481
Dolichogenidea Janzen53|[1791]|DHJPAR0025369|Costa Rica|642[1n]|BOLD:AAC7481
Dolichogenidea Janzen53|[1792]|BIOUG31724-A11|Costa Rica|585[0n]|BOLD:AAC7481
Dolichogenidea Janzen53|[1793]|DHJPAR0025767|Costa Rica|631[0n]|BOLD:AAC7481
Dolichogenidea Janzen53|[1794]|BIOUG90942-E04|Costa Rica|646[0n]|BOLD:AAC7481
Dolichogenidea Janzen53|[1795]|BIOUG90823-H02|Costa Rica|651[0n]|BOLD:AAC7481
Dolichogenidea Janzen53|[1796]|DHJPAR0025468|Costa Rica|609[3n]|BOLD:AAC7481
Dolichogenidea Janzen53|[1797]|DHJPAR0025356|Costa Rica|634[2n]|BOLD:AAC7481
Dolichogenidea Janzen53|[1798]|DHJPAR0025502|Costa Rica|635[0n]|BOLD:AAC7481
Dolichogenidea Janzen53|[1799]|BIOUG28376-F12|Costa Rica|591[0n]|BOLD:AAC7481
Dolichogenidea Janzen53|[1800]|DHJPAR0027687|Costa Rica|611[5n]|BOLD:AAC7481
Dolichogenidea Janzen53|[1801]|DHJPAR0025783|Costa Rica|631[0n]|BOLD:AAC7481
Dolichogenidea Janzen53|[1802]|DHJPAR0025424|Costa Rica|657[0n]|BOLD:AAC7481
Dolichogenidea Janzen53|[1803]|DHJPAR0025743|Costa Rica|657[1n]|BOLD:AAC7481
Dolichogenidea Malaise2929|[1804]|BIOUG82444-A07|Costa Rica|654[0n]|BOLD:ACF2929
Dolichogenidea Malaise2929|[1805]|BIOUG82113-B02|Costa Rica|654[0n]|BOLD:ACF2929
Dolichogenidea Malaise2929|[1806]|BIOUG81500-D09|Costa Rica|654[0n]|BOLD:ACF2929
Dolichogenidea Malaise2929|[1807]|BIOUG91192-D05|Costa Rica|654[0n]|BOLD:ACF2929
Dolichogenidea Malaise2929|[1808]|BIOUG84945-B07|Costa Rica|653[0n]|BOLD:ACF2929
Dolichogenidea Malaise2929|[1809]|BIOUG87945-F07|Costa Rica|653[0n]|BOLD:ACF2929
Dolichogenidea Malaise2929|[1810]|BIOUG84565-B01|Costa Rica|652[0n]|BOLD:ACF2929
Dolichogenidea Malaise2929|[1811]|BIOUG83487-C07|Costa Rica|652[0n]|BOLD:ACF2929
Dolichogenidea Janzen255|[1812]|BIOUG82150-G01|Costa Rica|654[0n]|BOLD:ACF2929
Dolichogenidea Janzen255|[1813]|DHJPAR0013641|Costa Rica|381[0n]|BOLD:ACF2929
Dolichogenidea Janzen255|[1814]|BIOUG19826-A11|Costa Rica|573[0n]|BOLD:ACF2929
Dolichogenidea Janzen255|[1815]|BIOUG19940-E07|Costa Rica|576[1n]|BOLD:ACF2929
Dolichogenidea Janzen255|[1816]|DHJPAR0025363|Costa Rica|645[0n]|BOLD:ACF2929
Dolichogenidea|[1817]|CBG-A08882-C11|Costa Rica|654[0n]|BOLD:ACF2929
Microgastrinae|[1818]|CBG-A08652-E09|Costa Rica|655[0n]|BOLD:ACF2929
Dolichogenidea Janzen255|[1819]|DHJPAR0025863|Costa Rica|657[0n]|BOLD:ACF2929
Dolichogenidea Malaise3487|[1820]|BIOUG91002-B07|Costa Rica|642[0n]|BOLD:ACF2929
Dolichogenidea Malaise3487|[1821]|BIOUG88740-B12|Costa Rica|641[0n]|BOLD:ACF2929
Dolichogenidea Malaise3487|[1822]|BIOUG88740-A12|Costa Rica|643[0n]|BOLD:ACF2929
Dolichogenidea Malaise3487|[1823]|BIOUG84585-C02|Costa Rica|652[0n]|BOLD:ACF2929
Dolichogenidea Malaise3487|[1824]|BIOUG78470-G01|Costa Rica|654[0n]|BOLD:ACF2929
Dolichogenidea Janzen56|[1825]|BIOUG77544-F03|Costa Rica|655[0n]|BOLD:ACF2929
Dolichogenidea Janzen56|[1826]|DHJPAR0025364|Costa Rica|645[0n]|BOLD:ACF2929
Microgastrinae|[1827]|CBG-A09171-C11|Costa Rica|652[0n]|BOLD:ACF2929
Dolichogenidea Janzen56|[1828]|DHJPAR0026934|Costa Rica|421[0n]|BOLD:ACF2929
Dolichogenidea Janzen56|[1829]|BIOUG77614-C05|Costa Rica|652[0n]|BOLD:ACF2929
Dolichogenidea Janzen56|[1830]|BIOUG83067-E03|Costa Rica|653[0n]|BOLD:ACF2929
Dolichogenidea Malaise3487|[1831]|BIOUG83487-D12|Costa Rica|653[0n]|BOLD:ACF2929
Dolichogenidea Malaise3487|[1832]|BIOUG86896-E08|Costa Rica|653[0n]|BOLD:ACF2929
Dolichogenidea Janzen56|[1833]|DHJPAR0025828|Costa Rica|656[0n]|BOLD:ACF2929
Dolichogenidea Janzen56|[1834]|DHJPAR0026846|Costa Rica|657[0n]|BOLD:ACF2929
Dolichogenidea Janzen359|[1835]|BIOUG70860-G04|Costa Rica|651[0n]|BOLD:ACE8228
Dolichogenidea Janzen359|[1836]|DHJPAR0031303|Costa Rica|621[0n]|BOLD:ACE8228
Dolichogenidea Janzen359|[1837]|BIOUG91473-F09|Costa Rica|656[0n]|BOLD:ACE8228
Dolichogenidea|[1838]|BIOUG08167-E03|Honduras|658[0n]|BOLD:ACE8228
Hymenoptera|[1839]|BIOUG91473-F09.ONT3|Costa Rica|658[0n]|BOLD:ACE8228
Dolichogenidea Janzen0272|[1840]|DHJPAR0012556|Costa Rica|657[0n]|BOLD:ACF0272
Dolichogenidea Janzen0272|[1841]|DHJPAR0012745|Costa Rica|657[0n]|BOLD:ACF0272
Dolichogenidea Janzen0272|[1842]|DHJPAR0012733|Costa Rica|657[0n]|BOLD:ACF0272
Dolichogenidea Janzen0272|[1843]|DHJPAR0013487|Costa Rica|657[0n]|BOLD:ACF0272
Dolichogenidea Janzen0272|[1844]|DHJPAR0013230|Costa Rica|657[0n]|BOLD:ACF0272
Dolichogenidea Janzen0272|[1845]|DHJPAR0013482|Costa Rica|657[0n]|BOLD:ACF0272
Dolichogenidea Janzen0272|[1846]|DHJPAR0024747|Costa Rica|657[0n]|BOLD:ACF0272
Dolichogenidea Janzen0272|[1847]|BIOUG36967-H08|Costa Rica|537[0n]|BOLD:ACF0272
Dolichogenidea Janzen0272|[1848]|BIOUG32981-D01|Costa Rica|579[0n]|BOLD:ACF0272
Dolichogenidea Janzen0272|[1849]|BIOUG36704-A12|Costa Rica|600[0n]|BOLD:ACF0272
Dolichogenidea Janzen0272|[1850]|BIOUG33119-C02|Costa Rica|603[0n]|BOLD:ACF0272
Dolichogenidea Janzen0272|[1851]|BIOUG33119-F11|Costa Rica|603[0n]|BOLD:ACF0272
Dolichogenidea Janzen0272|[1852]|BIOUG32765-D06|Costa Rica|604[0n]|BOLD:ACF0272
Dolichogenidea Janzen0272|[1853]|DHJPAR0012551|Costa Rica|653[0n]|BOLD:ACF0272
Dolichogenidea Janzen0272|[1854]|BIOUG71864-F08|Costa Rica|653[0n]|BOLD:ACF0272
Dolichogenidea Janzen0272|[1855]|DHJPAR0013224|Costa Rica|657[1n]|BOLD:ACF0272
Dolichogenidea Janzen2930|[1856]|BIOUG92313-B05|Costa Rica|653[0n]|BOLD:ACF2930
Dolichogenidea Janzen2930|[1857]|BIOUG93726-B11|Costa Rica|654[0n]|BOLD:ACF2930
Dolichogenidea Janzen2930|[1858]|BIOUG92313-C01|Costa Rica|654[0n]|BOLD:ACF2930
Dolichogenidea Janzen2930|[1859]|DHJPAR0031509|Costa Rica|658[0n]|BOLD:ACF2930
Dolichogenidea Janzen2930|[1860]|DHJPAR0012559|Costa Rica|657[0n]|BOLD:ACF2930
Dolichogenidea Janzen2930|[1861]|BIOUG33119-D12|Costa Rica|590[0n]|BOLD:ACF2930
Dolichogenidea Janzen2930|[1862]|DHJPAR0012730|Costa Rica|657[0n]|BOLD:ACF2930
Dolichogenidea Janzen2930|[1863]|DHJPAR0013516|Costa Rica|657[0n]|BOLD:ACF2930
Dolichogenidea Janzen2930|[1864]|DHJPAR0013486|Costa Rica|657[0n]|BOLD:ACF2930
Dolichogenidea Janzen2930|[1865]|DHJPAR0013484|Costa Rica|657[0n]|BOLD:ACF2930
Dolichogenidea Janzen2930|[1866]|DHJPAR0031516|Costa Rica|658[0n]|BOLD:ACF2930

Dolichogenidea Janzen2930|[1863]|DHJPAR0013516|Costa Rica|657[0n]|BOLD:ACF2930
Dolichogenidea Janzen2930|[1864]|DHJPAR0013486|Costa Rica|657[0n]|BOLD:ACF2930
Dolichogenidea Janzen2930|[1865]|DHJPAR0013484|Costa Rica|657[0n]|BOLD:ACF2930
Dolichogenidea Janzen2930|[1866]|DHJPAR0031516|Costa Rica|658[0n]|BOLD:ACF2930
Dolichogenidea Janzen459|[1867]|DHJPAR0026913|Costa Rica|620[0n]|BOLD:ACF2930
Dolichogenidea Janzen459|[1868]|DHJPAR0026381|Costa Rica|657[0n]|BOLD:ACF2930
Dolichogenidea Janzen459|[1869]|DHJPAR0026947|Costa Rica|657[0n]|BOLD:ACF2930
Dolichogenidea Janzen457|[1870]|DHJPAR0033777|Costa Rica|658[0n]|BOLD:ACF0267
Hymenoptera|[1871]|CBG-A04624-G05|Costa Rica|653[0n]|BOLD:AAB5549
Dolichogenidea Janzen23|[1872]|DHJPAR0034083|Costa Rica|658[0n]|BOLD:AAB5549
Dolichogenidea Janzen23|[1873]|DHJPAR0034130|Costa Rica|658[0n]|BOLD:AAB5549
Dolichogenidea Janzen23|[1874]|DHJPAR0034133|Costa Rica|658[0n]|BOLD:AAB5549
Dolichogenidea Janzen23|[1875]|DHJPAR0034154|Costa Rica|658[0n]|BOLD:AAB5549
Dolichogenidea Janzen23|[1876]|DHJPAR0034155|Costa Rica|658[0n]|BOLD:AAB5549
Dolichogenidea Janzen23|[1877]|DHJPAR0034157|Costa Rica|658[0n]|BOLD:AAB5549
Dolichogenidea Janzen23|[1878]|DHJPAR0034158|Costa Rica|658[0n]|BOLD:AAB5549
Dolichogenidea Janzen23|[1879]|DHJPAR0034166|Costa Rica|658[0n]|BOLD:AAB5549
Dolichogenidea Janzen23|[1880]|DHJPAR0034167|Costa Rica|658[0n]|BOLD:AAB5549
Dolichogenidea Janzen23|[1881]|DHJPAR0034168|Costa Rica|658[0n]|BOLD:AAB5549
Dolichogenidea Janzen23|[1882]|DHJPAR0031435|Costa Rica|658[0n]|BOLD:AAB5549
Dolichogenidea Janzen23|[1883]|DHJPAR0031441|Costa Rica|658[0n]|BOLD:AAB5549
Dolichogenidea Janzen23|[1884]|DHJPAR0031448|Costa Rica|658[0n]|BOLD:AAB5549
Dolichogenidea Janzen23|[1885]|DHJPAR0031449|Costa Rica|658[0n]|BOLD:AAB5549
Dolichogenidea Janzen23|[1886]|DHJPAR0031450|Costa Rica|658[0n]|BOLD:AAB5549
Dolichogenidea Janzen23|[1887]|DHJPAR0031453|Costa Rica|658[0n]|BOLD:AAB5549
Dolichogenidea Janzen23|[1888]|DHJPAR0031467|Costa Rica|658[0n]|BOLD:AAB5549
Dolichogenidea Janzen23|[1889]|DHJPAR0031468|Costa Rica|658[0n]|BOLD:AAB5549
Dolichogenidea Janzen23|[1890]|DHJPAR0031485|Costa Rica|658[0n]|BOLD:AAB5549
Dolichogenidea Janzen23|[1891]|DHJPAR0031508|Costa Rica|658[0n]|BOLD:AAB5549
Dolichogenidea Janzen23|[1892]|DHJPAR0031521|Costa Rica|658[0n]|BOLD:AAB5549
Dolichogenidea Janzen23|[1893]|DHJPAR0031545|Costa Rica|658[0n]|BOLD:AAB5549
Dolichogenidea Janzen23|[1894]|DHJPAR0031552|Costa Rica|658[0n]|BOLD:AAB5549
Dolichogenidea Janzen23|[1895]|DHJPAR0031553|Costa Rica|658[0n]|BOLD:AAB5549
Dolichogenidea Janzen23|[1896]|DHJPAR0012553|Costa Rica|657[0n]|BOLD:AAB5549
Dolichogenidea Janzen23|[1897]|DHJPAR0012561|Costa Rica|657[0n]|BOLD:AAB5549
Dolichogenidea Janzen23|[1898]|DHJPAR0012723|Costa Rica|657[0n]|BOLD:AAB5549
Dolichogenidea Janzen23|[1899]|DHJPAR0012732|Costa Rica|657[0n]|BOLD:AAB5549
Dolichogenidea Janzen23|[1900]|DHJPAR0012737|Costa Rica|657[0n]|BOLD:AAB5549
Dolichogenidea Janzen23|[1901]|DHJPAR0012752|Costa Rica|657[0n]|BOLD:AAB5549
Dolichogenidea Janzen23|[1902]|DHJPAR0013393|Costa Rica|657[0n]|BOLD:AAB5549
Dolichogenidea Janzen23|[1903]|DHJPAR0013519|Costa Rica|657[0n]|BOLD:AAB5549
Dolichogenidea Janzen23|[1904]|DHJPAR0013524|Costa Rica|657[0n]|BOLD:AAB5549
Dolichogenidea Janzen23|[1905]|DHJPAR0013227|Costa Rica|657[0n]|BOLD:AAB5549
Dolichogenidea Janzen23|[1906]|DHJPAR0013521|Costa Rica|657[0n]|BOLD:AAB5549
Dolichogenidea Janzen23|[1907]|DHJPAR0013508|Costa Rica|657[0n]|BOLD:AAB5549
Dolichogenidea Janzen23|[1908]|DHJPAR0013225|Costa Rica|657[0n]|BOLD:AAB5549
Dolichogenidea Janzen23|[1909]|DHJPAR0013515|Costa Rica|657[0n]|BOLD:AAB5549
Dolichogenidea Janzen23|[1910]|DHJPAR0013471|Costa Rica|657[0n]|BOLD:AAB5549
Dolichogenidea Janzen23|[1911]|DHJPAR0013491|Costa Rica|657[0n]|BOLD:AAB5549
Dolichogenidea Janzen23|[1912]|DHJPAR0013502|Costa Rica|657[0n]|BOLD:AAB5549
Dolichogenidea Janzen23|[1913]|DHJPAR0013507|Costa Rica|657[0n]|BOLD:AAB5549
Hymenoptera|[1914]|CBG-A06120-C01|Costa Rica|655[0n]|BOLD:AAB5549
Dolichogenidea Janzen23|[1915]|BIOUG68481-H01|Costa Rica|654[0n]|BOLD:AAB5549
Dolichogenidea Janzen23|[1916]|BIOUG68382-E06|Costa Rica|653[0n]|BOLD:AAB5549
Dolichogenidea Janzen23|[1917]|BIOUG70648-F06|Costa Rica|653[0n]|BOLD:AAB5549
Hymenoptera|[1918]|CBG-A06069-A07|Costa Rica|653[0n]|BOLD:AAB5549
Hymenoptera|[1919]|CBG-A06082-H09|Costa Rica|653[0n]|BOLD:AAB5549
Hymenoptera|[1920]|CBG-A06096-B11|Costa Rica|653[0n]|BOLD:AAB5549
Dolichogenidea Janzen23|[1921]|BIOUG68382-D04|Costa Rica|652[0n]|BOLD:AAB5549
Dolichogenidea Janzen23|[1922]|BIOUG37575-E07|Costa Rica|595[0n]|BOLD:AAB5549
Dolichogenidea Janzen23|[1923]|BIOUG31570-H04|Costa Rica|567[0n]|BOLD:AAB5549
Dolichogenidea Janzen23|[1924]|BIOUG31518-B10|Costa Rica|588[0n]|BOLD:AAB5549
Dolichogenidea Janzen23|[1925]|DHJPAR0012724|Costa Rica|630[0n]|BOLD:AAB5549
Hymenoptera|[1926]|CBG-A04333-E04|Costa Rica|654[0n]|BOLD:AAB5549
Microgastrinae|[1927]|CBG-A06404-B12|Costa Rica|654[0n]|BOLD:AAB5549
Microgastrinae|[1928]|CBG-A06858-F10|Costa Rica|654[0n]|BOLD:AAB5549
Dolichogenidea Janzen23|[1929]|DHJPAR0013208|Costa Rica|655[0n]|BOLD:AAB5549
Dolichogenidea Janzen23|[1930]|DHJPAR0013226|Costa Rica|657[1n]|BOLD:AAB5549
Hymenoptera|[1931]|CBG-A06040-H01|Costa Rica|669[0n]|BOLD:AAB5549
Apanteles Rodriguez190|[1932]|BIOUG90479-C06|Costa Rica|653[0n]|BOLD:AAF7717
Apanteles Rodriguez190|[1933]|BIOUG82055-F11|Costa Rica|652[0n]|BOLD:AAF7717
Apanteles Rodriguez190|[1934]|DHJPAR0026313|Costa Rica|657[0n]|BOLD:AAF7717
Apanteles Rodriguez190|[1935]|DHJPAR0025511|Costa Rica|657[0n]|BOLD:AAF7717
Apanteles Rodriguez190|[1936]|DHJPAR0025187|Costa Rica|657[1n]|BOLD:AAF7717
Braconidae|[1937]|CBG-A08054-D09|Costa Rica|653[0n]|BOLD:AAJ1396
Dolichogenidea Janzen1396|[1938]|DHJPAR0025961|Costa Rica|657[0n]|BOLD:AAJ1396
Dolichogenidea Janzen1396|[1939]|DHJPAR0026079|Costa Rica|657[0n]|BOLD:AAJ1396
Dolichogenidea Rodriguez187|[1940]|BIOUG88739-D02|Costa Rica|653[0n]|BOLD:AAD6850
Dolichogenidea Rodriguez187|[1941]|DHJPAR0025352|Costa Rica|632[0n]|BOLD:AAD6850
Dolichogenidea Rodriguez187|[1942]|DHJPAR0025581|Costa Rica|657[0n]|BOLD:AAD6850
Dolichogenidea Rodriguez187|[1943]|DHJPAR0026801|Costa Rica|657[0n]|BOLD:AAD6850
Dolichogenidea Rodriguez187|[1944]|DHJPAR0026805|Costa Rica|657[0n]|BOLD:AAD6850
Dolichogenidea Rodriguez187|[1945]|DHJPAR0026946|Costa Rica|657[0n]|BOLD:AAD6850
Dolichogenidea|[1946]|CNCH0621|Venezuela|640[0n]|BOLD:AAF5364
Dolichogenidea Janzen257|[1947]|BIOUG67916-G10|Costa Rica|654[0n]|BOLD:AAF5364
Dolichogenidea Janzen257|[1948]|BIOUG81047-F09|Costa Rica|653[0n]|BOLD:AAF5364
Hymenoptera|[1949]|CBG-A00726-A04|Costa Rica|656[0n]|BOLD:AAF5364
Dolichogenidea Janzen257|[1950]|BIOUG92585-A04|Costa Rica|654[0n]|BOLD:AAF5364
Dolichogenidea Janzen257|[1951]|DHJPAR0058279|Costa Rica|629[0n]|BOLD:AAF5364
Dolichogenidea Janzen257|[1952]|DHJPAR0013660|Costa Rica|583[0n]|BOLD:AAF5364
Dolichogenidea Janzen257|[1953]|DHJPAR0051053|Costa Rica|601[0n]|BOLD:AAF5364
Dolichogenidea Janzen257|[1954]|DHJPAR0012744|Costa Rica|657[0n]|BOLD:AAF5364
Dolichogenidea Janzen257|[1955]|DHJPAR0051056|Costa Rica|658[1n]|BOLD:AAF5364
Apanteles Rodriguez148|[1956]|DHJPAR0033737|Costa Rica|658[0n]|BOLD:AAM5736
Apanteles Rodriguez148|[1957]|DHJPAR0033964|Costa Rica|658[0n]|BOLD:AAM5736
Apanteles Rodriguez148|[1958]|DHJPAR0031312|Costa Rica|658[0n]|BOLD:AAM5736
Apanteles Rodriguez148|[1959]|BIOUG59060-H03|Costa Rica|655[0n]|BOLD:AAM5736
Apanteles Rodriguez148|[1960]|BIOUG49263-G08|Costa Rica|655[0n]|BOLD:AAM5736
Apanteles Rodriguez148|[1961]|CBG-A13507-F06|Costa Rica|654[0n]|BOLD:AAM5736
Apanteles Rodriguez148|[1962]|CBG-A13450-A09|Costa Rica|671[0n]|BOLD:AAM5736
