# Supplementary material for: ﻿A revision of the parasitoid wasp genus Dolichogenidea Viereck (Hymenoptera, Braconidae) in the Neotropical region, with the description of 102 new species
Source: Zookeys. 2025 May 7;1237:1–250. doi: 10.3897/zookeys.1237.141007 (PMC12079117; doi:10.3897/zookeys.1237.141007)
Supplement: ﻿Supplementary material 5 — Additional figures [file zookeys-1237-001_article-141007__-s005.pdf]

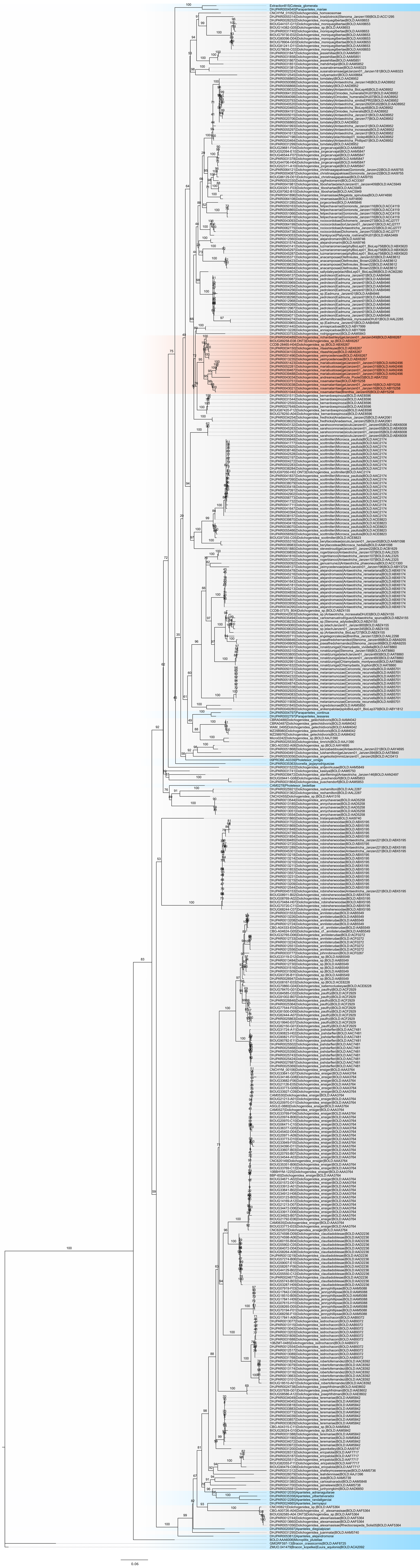
0.06
